# Comprehensive Profiling of Secretome Formulations from Fetal- and Perinatal Human Amniotic Fluid Stem Cells

**DOI:** 10.3390/ijms22073713

**Published:** 2021-04-02

**Authors:** Ambra Costa, Davide Ceresa, Antonella De Palma, Rossana Rossi, Sara Turturo, Sara Santamaria, Carolina Balbi, Federico Villa, Daniele Reverberi, Katia Cortese, Pierangela De Biasio, Dario Paladini, Domenico Coviello, Silvia Ravera, Paolo Malatesta, Pierluigi Mauri, Rodolfo Quarto, Sveva Bollini

**Affiliations:** 1 Experimental Biology Unit, Department of Experimental Medicine (DIMES), University of Genova, 16132 Genova, Italy; costa.ambra93@gmail.com (A.C.); saraturturo96@gmail.com (S.T.); paolo.malatesta@unige.it (P.M.); 2 Cellular Oncology Unit, IRCCS Ospedale Policlinico San Martino, 16132 Genova, Italy; davide.ceresa91@gmail.com; 3 Proteomics and Metabolomics Unit, Institute for Biomedical Technologies (ITB-CNR), 20054 Milan, Italy; antonella.depalma@itb.cnr.it (A.D.P.); rossana.rossi@itb.cnr.it (R.R.); pierluigi.mauri@itb.cnr.it (P.M.); 4 Human Anatomy Unit, Department of Experimental Medicine (DIMES), University of Genova, 16132 Genova, Italy; sara.santamaria1994@gmail.com (S.S.); cortesek@unige.it (K.C.); silvia.ravera@unige.it (S.R.); 5 Laboratory of Cellular and Molecular Cardiology, Cardiocentro Ticino Foundation, 6900 Lugano, Switzerland; carolina.balbi@cardiocentro.org; 6 Center for Molecular Cardiology, University of Zurich, 8952 Zurich, Switzerland; 7 Molecular Oncology and Angiogenesis Unit, IRCCS Ospedale Policlinico San Martino, 16132 Genova, Italy; federico.villa@hsanmartino.it; 8 Molecular Pathology Unit, IRCCS Ospedale Policlinico, San Martino, 16132 Genova, Italy; daniele.reverberi@hsanmartino.it; 9 Prenatal Diagnosis and Perinatal Medicine Unit, IRCCS Ospedale Policlinico San Martino, 16132 Genova, Italy; pierangelaleonilde@gmail.com; 10 Fetal Medicine and Surgery Unit, IRCCS Istituto Giannina Gaslini, 16147 Genova, Italy; dpaladini49@gmail.com; 11 Laboratory of Human Genetics, IRCCS Istituto Giannina Gaslini, 16147 Genova, Italy; covdom56@gmail.com

**Keywords:** amniotic fluid, stem cells, paracrine effects, extracellular vesicles, cell-conditioned medium, chemokine, cytokines, proteomics, RNA sequencing, microRNA

## Abstract

We previously reported that c-KIT+ human amniotic-fluid derived stem cells obtained from leftover samples of routine II trimester prenatal diagnosis (fetal hAFS) are endowed with regenerative paracrine potential driving pro-survival, anti-fibrotic and proliferative effects. hAFS may also be isolated from III trimester clinical waste samples during scheduled C-sections (perinatal hAFS), thus offering a more easily accessible alternative when compared to fetal hAFS. Nonetheless, little is known about the paracrine profile of perinatal hAFS. Here we provide a detailed characterization of the hAFS total secretome (i.e., the entirety of soluble paracrine factors released by cells in the conditioned medium, hAFS-CM) and the extracellular vesicles (hAFS-EVs) within it, from II trimester fetal- versus III trimester perinatal cells. Fetal- and perinatal hAFS were characterized and subject to hypoxic preconditioning to enhance their paracrine potential. hAFS-CM and hAFS-EV formulations were analyzed for protein and chemokine/cytokine content, and the EV cargo was further investigated by RNA sequencing. The phenotype of fetal- and perinatal hAFS, along with their corresponding secretome formulations, overlapped; yet, fetal hAFS showed immature oxidative phosphorylation activity when compared to perinatal ones. The profiling of their paracrine cargo revealed some differences according to gestational stage and hypoxic preconditioning. Both cell sources provided formulations enriched with neurotrophic, immunomodulatory, anti-fibrotic and endothelial stimulating factors, and the immature fetal hAFS secretome was defined by a more pronounced pro-vasculogenic, regenerative, pro-resolving and anti-aging profile. Small RNA profiling showed microRNA enrichment in both fetal- and perinatal hAFS-EV cargo, with a stably- expressed pro-resolving core as a reference molecular signature. Here we confirm that hAFS represents an appealing source of regenerative paracrine factors; the selection of either fetal or perinatal hAFS secretome formulations for future paracrine therapy should be evaluated considering the specific clinical scenario.

## 1. Introduction

Regenerative medicine has recently developed as an emerging field to provide functional restoration of injured tissue by means of several strategies. As tissue engineering approaches have significantly advanced in recent years, the investigation of stem cell paracrine effects has concomitantly increasingly intensified. The therapeutic potential of transplanted stem cells has been broadly shown to be mostly mediated by their secreted soluble factors, which can orchestrate a pro-regenerative microenvironment in the host tissue while triggering the activation of endogenous mechanisms of functional recovery [1,2]. Therefore, the stem cell *secretome* the entirety of cell-released paracrine trophic molecules, as well as membrane-bound extracellular vesicles has been increasingly proposed as an innovative therapy medicinal product by multiple independent preclinical studies targeting cardiovascular, neurological and/or inflammatory disease. Accordingly, stem cells may be envisioned as biological factories for the exploitation of their therapeutic secretome by offering ready-to-use and off-the-shelf regenerative treatments. By applying such a cell-based, yet cell-free strategy, many limiting aspects associated to canonical cell-therapy may be overcome, while still ensuring beneficial effects. In this perspective, mesenchymal stromal cells (MSC) have been extensively tested as putative cell candidates. Indeed, MSC and stem/progenitor cells have been engineered and/or stimulated by different preconditioning strategies to enhance their regenerative capacity and secretory potential [3,4], with explicit interest towards the biological relevance of their secreted extracellular vesicles (EVs).

EVs are nano-sized particles delimited by a lipid bilayer and actively secreted by all cell types. EVs include very small (<200 nm) exosomes, medium-sized (200–500 nm) microvesicles or shedding vesicles, and larger-sized apoptotic bodies (>500 nm); they operate as critical biological conveyors of inter-cellular signaling by delivering their molecular cargo from a parental cell to a responder/target one [5,6]. Given that their peculiar paracrine potential in exerting beneficial effects are comparable to their cells of origin, stem cell-EVs have arisen as appealing therapeutic options in preclinical models of diseases, such as ischemia, inflammation or injury, as extensively reviewed in [7,8,9]. From a translational perspective, on top of cell modulatory potential, isolation feasibility and elevated self-renewal are key aspects for the ideal source of therapeutic EVs and soluble factors. In such a scenario, fetal- and perinatal MSC may offer an interesting option given their proliferative potential, and developmentally immature profile with intermediate features between embryonic and adult somatic progenitors [10,11]. Fetal MSC can be isolated from extra-embryonic annexes during gestation as left-over sampling obtained during prenatal screening (i.e., chorionic villi [12,13,14] and amniotic fluid [15,16]) or obtained as perinatal progenitors at birth, from clinical waste material (i.e., amniotic and placenta membranes [17,18,19,20,21], umbilical cord components [22,23,24] and term amniotic fluid [25,26]).

Notably, human amniotic fluid stem cells (hAFS) have been highlighted as promising therapeutic strategyin regenerative medicine. hAFS have been shown to be broadly multipotent in vitro and in vivo [16,27,28], contribute to the hematopoietic lineage following in utero transplantation [29] and engraft in injured organs while exerting immunomodulatory effects [26,30] and activating endogenous reparative responses, as comprehensively described in [31]. Our team and others have further demonstrated that hAFS release a secretome highly enriched with bioactive trophic molecules able to target different reparative mechanisms. hAFS paracrine factors have been reported to provide pro-survival stimuli with the quenching of inflammation [32], provide cardioprotection against prolonged ischemia [33,34] and cardiotoxicity [35], and stimulate local angiogenesis with cardiomyocyte cell cycle re-entry [34,36]. Since most of these effects have been shown to be recapitulated by hAFS-EV administration alone, independent studies have focused on dissecting their regenerative profile against different pathological backgrounds, including skeletal and cardiac muscle injury, kidney disease, osteoarthritis, osteoporosis, necrotizing enterocolitis and neurodegenerative models [34,37,38,39,40,41,42,43,44].

While evidence may support the clinical translation of hAFS-EVs for future paracrine therapy, it is important to consider that most of these studies have mainly investigated the modulatory potential of fetal hAFS obtained during II trimester prenatal screening. Indeed, complete profile of the secretome from the perinatal hAFS counterpart (i.e., from III trimester c-sections) has not yet been explored in detail. Third trimester perinatal hAFS have shown distinctive immune regulatory properties compared to I- and II-trimester ones [26], while maintaining relevant endothelial regenerative potential [25]. Of note, the recent report of the heterogeneous morphology of fetal hAFS [45] has provided new insights on their stemness and gene expression profile. This has altogether shed new light on the regenerative value of the different cellular fractions of hAFS [46]. Hence, comprehensive characterization of the different subpopulations of hAFS is attracting mounting attention.

We previously reported that a 24 h hypoxic and serum-free stimulation represents an effective strategy to boost the paracrine potential of II trimester fetal hAFS [34,35,37]. Since little is known about the composition of the secretome from III trimester hAFS, here we report the comprehensive comparison of II versus III trimester hAFS and their secretome fractions (including hAFS-EVs), in order to address the influence of gestational stage and hypoxic cell preconditioning on cell and secretome characteristics.

## 2. Results

### 2.1. Perinatal hAFS Present a Close Phenotypic Match to Fetal hAFS

**Figure 1:**
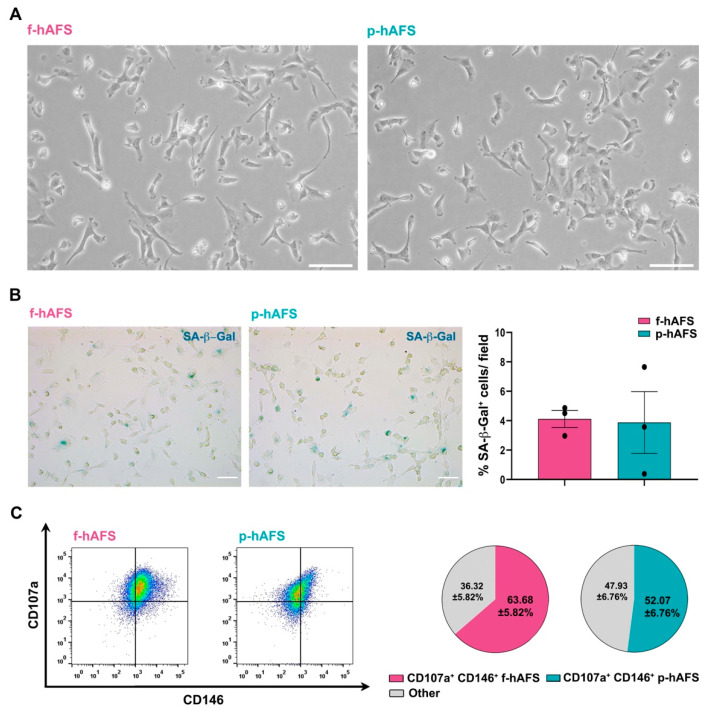
Fetal hAFS and perinatal hAFS phenotypic evaluation. (**A**) Representative images of fetal hAFS (f-hAFS, left panel) and perinatal hAFS (p-hAFS, right panel) cultured in vitro in standard conditions; scale bar: 200 µm. (**B**) Analysis of the senescent marker beta-galactosidase (SA-β-Gal, in blue) via cytochemistry staining on f-hAFS and p-hAFS after 5 passages in culture; representative images are reported in the left panel, scale bar: 200 µm. The corresponding percentage of β-Gal-positive cells/field is reported in the graph in the right panel (f-hAFS: 4.12 ± 0.58% and p-hAFS: 3.88 ± 2.10%; *p* = 0.1424, *n* = 3 experiments). (**C**) Immunophenotype of hAFS expressing CD146 and CD107a mesenchymal markers. Representative flow cytometry plots of f-hAFS and p-hAFS (left panel) and corresponding values referred to double positive CD107a^+^ CD146^+^ cells; CD107a^+^ CD146^+^ f-hAFS: 63.68 ± 5.82%, * *p* = 0.016 compared to remaining 36.32 ± 5.82% f-hAFS (Other); CD107a^+^ CD146^+^ p-hAFS: 52.07 ± 6.76% with remaining 47.93 ± 56.76% p-hAFS (Other); CD107a^+^ CD146^+^ f-hAFS vs CD107a^+^ CD146^+^ p-hAFS *p* = 0.2403, *n* = 4 experiments. *Other*: total amount of remaining CD107a^−^ CD146^−^ hAFS, CD107a^−^ CD146^+^ hAFS and CD107a^+^ CD146^−^ hAFS. All values are expressed as mean ± s.e.m of independent experiments. *SA-β-Gal: Senescence-Associated-β-galactosidase.*

No statistically relevant difference was appreciated in donor age between fetal II trimester and perinatal III trimester amniotic fluid samples. Fetal c-KIT^+^ hAFS (f-hAFS from II trimester amniotic fluid samples) and perinatal c-KIT^+^ hAFS (p-hAFS from III trimester amniotic fluid clinical waste) confirmed similar features with fibroblast-like and oval-round morphology (Figure 1A) and mesenchymal stromal phenotype (data not shown), as previously reported [16,25]. Both f-hAFS and p-hAFS cultured in vitro up to passage 5 showed negligible level of senescence as from senescence-associated-β-galactosidase (SA-β-Gal) activation in about 4% cells (Figure 1B). Both f-hAFS and p-hAFS presented high level of co-expression of the mesenchymal markers CD107a and CD146, which have been recently reported to define a highly secretory phenotype [47]. CD107a^+^ CD146^+^ cells represented the majority of f-hAFS population (approximately 64%, * *p* < 0.05), while p-hAFS showed a lower enrichment for this subpopulation, approximately 52% of total cells, yet this disparity was not statistically significant (Figure 1C).

### 2.2. Fetal hAFS Show a Different Metabolism from Perinatal hAFS

**Figure 2:**
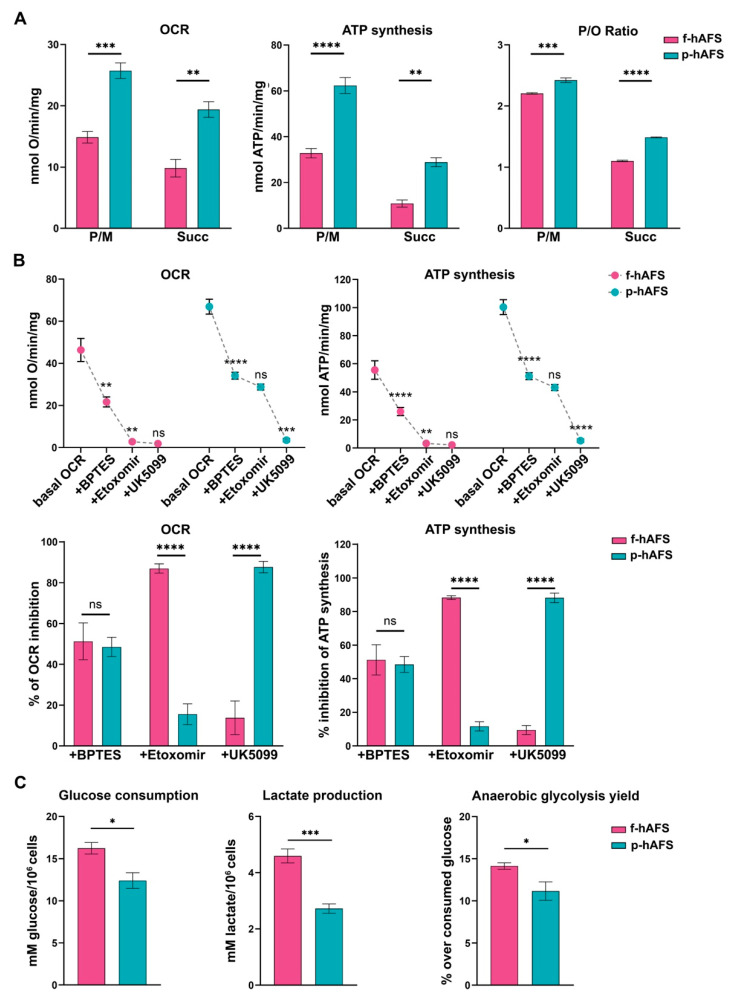
Metabolic characterization of fetal- and perinatal hAFS. (**A**) Oxygen consumption rate (OCR), ATP synthesis through F_1_-F_o_ ATP synthase, and P/O ratio in f-hAFS and p-hAFS in the presence of pyruvate plus malate (P/M) or succinate (Succ); *** *p* = 0.0005, ** *p* = 0.0012, **** *p* < 0.0001, ** *p* = 0.0013, *** *p* = 0.0002, **** *p* < 0.0001. (**B**) OCR and ATP synthesis in presence of BPTES, Etomoxir, and UK5099 (upper panel) was sequentially added during the experiments to evaluate the relative contributions of glutamine, long-chain fatty acid oxidation and glucose in OxPhos metabolism in f-hAFS and p-hAFS. For OCR experiments: f-hAFS + BPTES ** *p* = 0.0014; for f-hAFS + Etomoxir ** *p* = 0.0088; for p-hAFS + BPTES **** *p* < 0.0001; for p-hAFS + UK5099 ** *p* < 0.0001. For ATP experiments: f-hAFS + BPTES **** *p* < 0.0001; for f-hAFS + Etomoxir ** *p* = 0.0013; for p-hAFS + BPTES **** *p* < 0.0001; for p-hAFS + UK5099 ** *p* < 0.0001). The comparison of percentage of inhibition of OCR and ATP synthesis in f- and p-hAFS due to the inhibitors indicated above is reported in the lower panel B. For OCR experiments: hAFS + Etomoxir **** *p* < 0.0001; for hAFS + UK5099 **** *p* < 0.0001. For ATP experiments: hAFS + Etomoxir **** *p* < 0.0001; for hAFS + UK5099 **** *p* < 0.0001). (**C**) Glucose consumption, lactate release and anaerobic glycolysis yield, used as markers of the anaerobic glycolysis, in f-hAFS and p-hAFS. All values are expressed as mean ± s.e.m of *n* = 4 independent experiments; * *p* = 0.016, *** *p* = 0.0008, * *p* = 0.0416, respectively.

To evaluate whether gestational stage may influence hAFS mitochondrial metabolism, f-hAFS and p-hAFS were analyzed in standard in vitro culture conditions by biochemical analyses. Evaluation of aerobic metabolism showed that the oxygen consumption rate (OCR) and ATP synthesis were lower in f-hAFS with respect to p-hAFS, both when stimulated with pyruvate + malate (P/M; *** *p* < 0.001 for OCR, and **** *p* < 0.0001, for ATP synthesis), and with succinate (** *p* < 0.01 for OCR and ATP synthesis, Figure 2A). Moreover, f-hAFS displayed a lower oxidative phosphorylation efficiency when compared to p-hAFS, as shown by the *p*/O values (*** *p* < 0.001 for P/M and **** *p* < 0.0001 for succinate). Values for f-hAFS were lower than those reported in literature [48], and suggest uncoupling between oxygen consumption and ATP production (Figure 2A). By evaluating the relative contributions of glutamine, long-chain fatty acid oxidation, and glucose in oxidative phosphorylation (OxPhos) metabolism, we noticed that f-hAFS were sensitive to the addition of BPTES (glutaminase inhibitor, ** *p* < 0.01) and etomoxir (carnitine palmitoyl-transferase 1A inhibitor, ** *p* < 0.01), but not to UK5099 (mitochondrial pyruvate carrier inhibitor). By contrast, BPTES (**** *p* < 0.0001) and UK5099 (*** *p* < 0.001), but not etomoxir, inhibited the metabolism of p-hAFS (Figure 2B, upper panel). This observation was confirmed by the inhibition percentage of the single inhibitor (Figure 2B, lower panel). Therefore, both cell types similarly rely on glutamine as a respiratory substrate; yet, f-hAFS prefer fatty acids as a second substrate, while p-hAFS are sustained by glucose. Interestingly, f-hAFS showed a higher increment of glucose consumption and lactate release when compared to p-hAFS (* *p* < 0.05 and *** *p* < 0.001 respectively, Figure 2C), which indicates the attempt to balance inefficient aerobic metabolism by lactate fermentation. This difference could also explain the reaction of f-hAFS to the addition of etomoxir and UK5099. Since f-hAFS favor the use of glucose during anaerobic glycolysis (* *p* < 0.05), they are likely forced to use fatty acids and glutamine to supply the aerobic metabolism.

### 2.3. Hypoxic Preconditioning Does Not Affect Fetal- and Perinatal hAFS Viability and Sustains Their Secretory Activity

**Figure 3:**
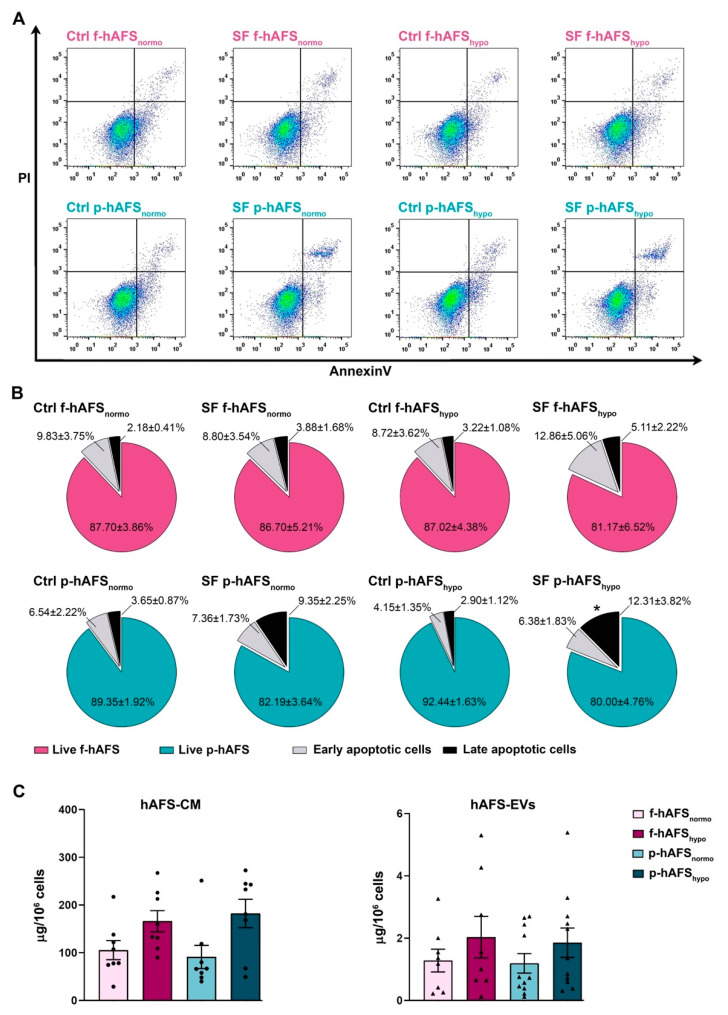
Evaluation of the preconditioning effect on fetal- and perinatal hAFS viability and on secretome yield. (**A**) Apoptosis analysis by flow cytometry evaluation of PI and Annexin V expression on f-hAFS and p-hAFS after 24 h preconditioning in either 20% O_2_ normoxic condition compared to the 1% O_2_ hypoxic condition, and in complete control (Ctrl) medium versus serum-free (SF) medium (Ctrl f-hAFS_normo_; Ctrl f-hAFS_hypo_; SF f-hAFS_normo_; SF f-hAFS_hypo_; Ctrl p-hAFS_normo_; Ctrl p-hAFS_hypo_; SF p-hAFS_normo_; SF p-hAFS_hypo_, respectively). (**B**) Pie charts of live, early apoptotic and late apoptotic f-hAFS (*n* = 4 experiments, upper panel) and corresponding p-hAFS (*n* = 6 experiments, lower panel); *late apoptotic Ctrl p-hAFS_hypo_ vs SF p-hAFS_hypo_* * *p* = 0.0392; no statistically significant differences were detected among all other comparisons. (**C**) Protein concentration of hAFS secretome yield obtained from serum-free culture conditions by BCA assay. Left panel refers to the entire secretome formulation, namely the in vitro hAFS-conditioned medium (hAFS-CM). Right panel refers to hAFS-EV formulations (hAFS-EVs); *n* = 8 experiments for f-hAFS-EVs and *n* = 11 experiments for p-hAS-EVs. All values are expressed as mean ± s.e.m of independent experiments.

In order to define hAFS secretome formulations, cells were cultured in serum-free conditions to avoid any contamination from FBS. We previously showed that 24 h serum-free (SF) and 1% O_2_ hypoxic culture conditions did not significantly alter the viability of II trimester fetal hAFS (f-hAFS), while they supported the release of regenerative paracrine factors in their cell-conditioned medium (hAFS-CM) and in extracellular vesicles (hAFS-EVs) [34,35,37,49]. Herein, in addition to profiling the p-hAFS secretome fractions for the first time, we evaluated whether p-hAFS presented similar behavior under the same preconditioning regime, using the normoxic culture condition as the control. f-hAFS and p-hAFS viability was analyzed after 24 h in the following settings: normoxic (20% O_2_) condition in complete control (Ctrl) culture medium (Ctrl f-hAFS_normo_ and Ctrl p-hAFS_normo_), normoxic condition in SF medium (SF f-hAFS_normo_ and SF p-hAFS_normo_), hypoxic (1% O_2_) condition in complete control medium (Ctrl f-hAFS_hypo_ and Ctrl p-hAFS_hypo_), and hypoxic condition in SF medium (SF f-hAFS_hypo_ and SF p-hAFS_hypo_, Figure 3A). We confirmed that f-hAFS viability was unaltered in both Ctrl and SF conditions and under hypoxic stimulation, with more that 80% (up to almost 88%) of total cells being unaffected. Early and late apoptotic cells ranged from ca. 13% to 18% in SF conditions, without any statistically significant relevance. Likewise, perinatal hAFS viability was in the range of 80–92%, and early and late apoptotic cells represented up to 18% in SF conditions. p-hAFS were marginally influenced only under the combined hypoxic and SF conditions; indeed, while preconditioning did not influence cell survival when p-hAFS were cultured in complete medium, the corresponding SF condition showed an increase by ca. 4-fold (* *p* < 0.05) of late apoptotic cells (Figure 3B).

We then evaluated the yield of secretome fractions obtained from f-hAFS versus p-hAFS based on protein enrichment. The total hAFS secretome, as the entirety of the cell-secreted paracrine factors, is here represented by the hAFS-CM. The protein concentration of f-hAFS-CM and p-hAFS-CM in SF medium following hypoxic cell preconditioning vs control normoxic condition as baseline (namely, f-hAFS-CM_normo_, f-hAFS-CM_hypo_, p-hAFS-CM_normo_, and p-hAFS-CM_hypo,_) was evaluated by BCA assay and measured as per 10^6^ cells. The results acquired suggested that f-hAFS-CM and p-hAFS-CM showed an equal positive trend in protein enrichment following hypoxic priming (f-hAFS-CM_hypo_: 166.10 ± 22.13 µg/10^6^ cells; p-hAFS-CM_hypo_: 182.30 ± 29.71 µg/10^6^ cells) over their normoxic counterparts (f-hAFS-CM_normo_: 105.50 ± 19.89 µg/10^6^ cells; p-hAFS-CM_hypo_: 91.12 ± 24.39 µg/10^6^ cells). Likewise, the surface protein concentration of hAFS-EVs was measured in f-hAFS-EVs_normo_, f-hAFS-EVs_hypo_, p-hAFS-EVs_normo_, and p-hAFS-EVs_hypo._ EVs showed comparable yield when obtained from f-hAFS or p-hAFS. As for hAFS-CM formulations, a positive trend in the increase of protein content on f-hAFS-EVs and p-hAFS-EVs was appreciated after hypoxic stimulation over the corresponding normoxic condition (f-hAFS-EVs_hypo_: 2.03 ± 0.67 µg/10^6^ cells and p-hAFS-EVs_hypo_: 1.85 ± 0.47 µg/10^6^ cells; f-hAFS-EVs_normo_: 1.28 ± 0.36 µg/10^6^ cells and p-hAFS-EVs_normo_: 1.19 ± 0.31 µg/10^6^ cells, Figure 3C).

### 2.4. Fetal- and Perinatal hAFS Release EVs with Analogous Morphology and Size Distribution

**Figure 4:**
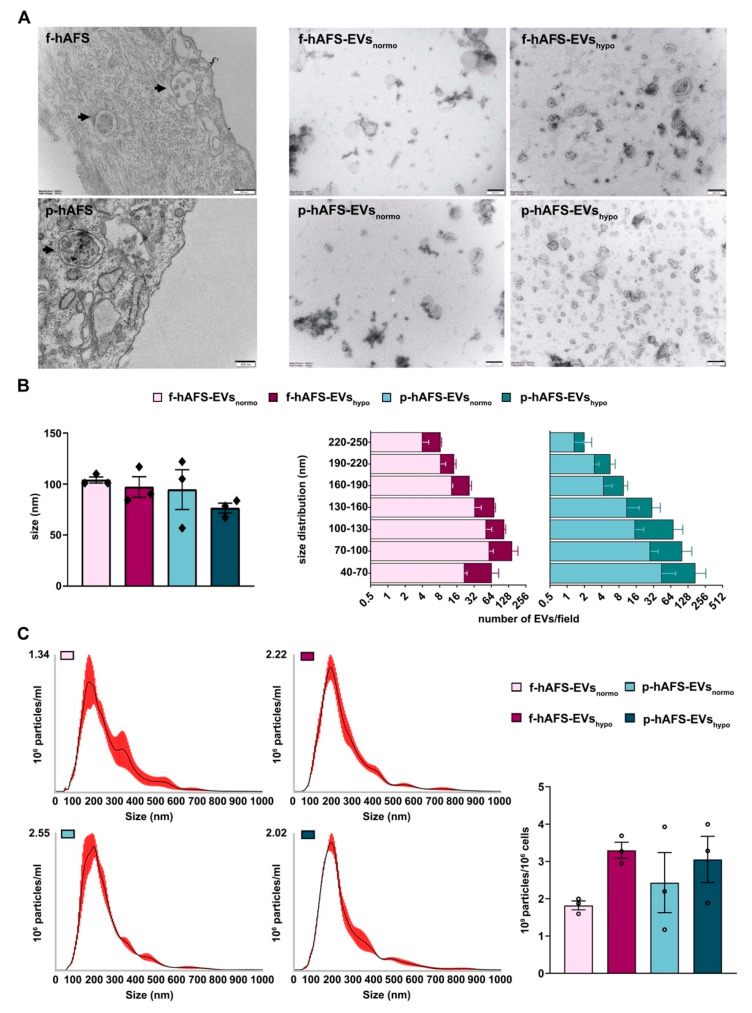
Morphological characterization of fetal- and perinatal hAFS-EVs. (**A**) Representative images of transmission electron microscopy (TEM) of: f-hAFS and p-hAFS (upper and lower left panel, respectively, black arrows indicating intracytoplasmic multi-vesicular bodies with small EVs/exosomes within them), and of f-hAFS-EVs and p-hAFS-EVs (upper and lower right panel, respectively) released in serum-free conditions and under normoxic versus hypoxic preconditioning (f-hAFS-EVs_normo_; f-hAFS-EVs_hypo_; p-hAFS-EVs_normo_; and p-hAFS-EVs_hypo_, respectively), scale bars: 200 nm. (**B**) Left panel: TEM analysis of hAFS-EVs size distribution; right panel: distribution of the number f-hAFS-EVs and p-hAFS-EVs per field size intervals from 40 nm up to 250 nm were considered; values are expressed as mean ± s.e.m of *n* = 3 independent experiments. (**C**) Nanoparticle tracking analysis for hAFS-EVs size and distribution. Left panel: representative image of the graphical output; right panel: hAFS-EVs concentration measured as 10^9^ particles per 10^6^ secreting cells; nm: nanometer; mL: milliliter.

Morphological analysis by transmission electron microscopy (TEM) highlighted the high EV-secretory prolife of both f-hAFS and p-hAFS (Figure 4A). We further investigated the size and area of f-hAFS-EVs and p-hAFS-EVs (Figure 4B) following hypoxic preconditioning compared to normoxic baseline. Fetal- and perinatal hAFS released EVs heterogeneous in size, in the range of 40–250 nm, hence including both exosomes/small EVs and microvesicles/shedding vesicles. The average size of EVs/field in the different groups was comparable, fetal hAFS-EVs measured 90–100 nm (f-hAFS-EVs_normo_: 104.00 ± 3.00 nm; f-hAFS-EVs_hypo_: 97.10 ± 10.10 nm) and perinatal ones measured 70–114 nm (p-hAFS-EVs_normo_: 94.60 ± 19.53 nm; p-hAFS-EVs_hypo_: 76.43 ± 4.86 nm, Figure 4B, left panel). As for the yield, hAFS stimulated under hypoxia showed a positive trend in the increase of the amount of small EVs, although this increase was not statistically significant. f-hAFS-EVs_hypo_ measured 40–70 nm which was almost twice that compared to their normoxic counterpart. Perinatal-hAFS-EVs_hypo_ that measured 40–70 nm, 70–100 nm and 100–130 nm were almost triple the amount of those obtained in normoxic culture (Figure 4B).

Nanoparticle tracking analysis (NTA) showed an elevated number of particles in both f-hAFS-EV and p-hAFS-EV preparations, and confirmed theincrease of EVs in the hypoxic samples, as also observed from the previous analyses (f-hAFS-EVs_normo_: 1.82 ± 0.12 × 10^9^ particles/10^6^ cells; f-hAFS-EVs_hypo_: 3.30 ± 0.22 × 10^9^ particles/10^6^ cells; p-hAFS-EVs_normo_: 2.43 ± 0.80 × 10^9^ particles/10^6^ cells; p-hAFS-EVs_hypo_: 3.05 ± 0.62 × 10^9^ particles/10^6^ cells, Figure 4C).

### 2.5. Proteomic Characterization of Fetal vs. Perinatal hAFS Highlights Differences in Their Secretome Composition According to Gestational Age and Hypoxic Preconditioning

Proteomic characterization of both f-hAFS and p-hAFS secretome formulations was performed by means of a shotgun label-free platform, based on the coupling of nano liquid chromatography and high-resolution mass spectrometry (nLC-hrMS). Forty-eight proteomic profiles were acquired by the duplicate analysis of three biological replicates of hAFS-CM and hAFS-EVs from f-hAFS and p-hAFS undergoing hypoxic cell preconditioning compared to the normoxic condition as a control. A total of 4179 distinct protein groups were identified with at least one unique peptide, and with molecular weights ranging from 2 to 3900 kDa and isoelectric points from 3.6 to 13. A higher average protein expression in hAFS-EVs was observed when compared to hAFS-CM. The alignment of all protein lists obtained was carried out on the basis of identified proteins. For each experimental condition, a unique list was created normalizing and averaging [50] the peptide spectrum match values (aPSMs) attributed to the proteins, which represent the number of mass spectra assigned to each and indirectly represents their abundance in the samples. The complete list of proteins identified in hAFS-CM and hAFS-EV formulations is reported in Table S1.

**Figure 5:**
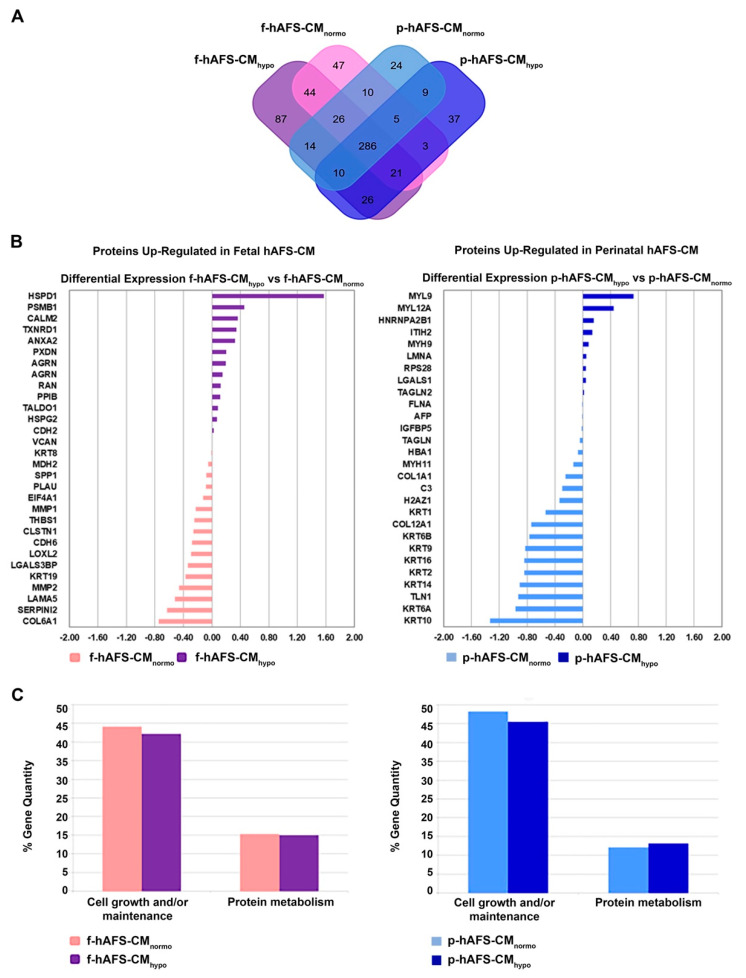
Comparative proteomics analysis of fetal- and perinatal hAFS-CM. (**A**) Venn diagram illustrating the distribution of proteins found with a frequency of at least 2 within f-hAFS-CM_normo_ (pink), f-hAFS-CM_hypo_ (purple), p-hAFS-CM_normo_ (light blue) and p-hAFS-CM_hypo_ (blue). (**B**) Differentially expressed proteins identified in fetal hAFS-CM (left panel) and perinatal hAFS-CM (right panel) by label-free quantification with MAProMa software. Left panel: histogram plot refers to the differential expression between normoxic control (pink bars and negative DAveDAve values) and hypoxic (purple bars and positive DAve values) condition of proteins found up-regulated in f-hAFS-CM over p-hAFS-CM. Right panel: histogram plot reporting the differential expression between normoxic control (light blue bars and negative DAve values) and hypoxic (blue bars and positive DAve values) condition of proteins found upregulated in p-hAFS-CM over f-hAFS-CM. Proteins with DAve (ratio of protein expression) ≥ |0.4| and a DCI (confidence of differential expression) ≥ |5| passed the filters and were considered differentially expressed. For each protein, gene name and related DAve value are reported; see Table S2 for the complete list and detailed parameters of the reported proteins. (**C**) Biological processes enrichment analysis of proteins identified with a frequency of at least 2 in fetal hAFS-CM (left panel) and perinatal hAFS-CM (right panel) according to cell hypoxic preconditioning. Based on FunRich tool, gene ontology terms are shown in bar charts reporting the percentage of genes enriched for each category (pink bars for f-hAFS-CM_normo_, purple bars for f-hAFS-CM_hypo_, light blue bars for p-hAFS-CM_normo_ and blue bars for p-hAFS-CM_hypo_). Only gene ontology terms with Bonferroni corrected with * *p* < 0.05 are reported.

**Figure 6:**
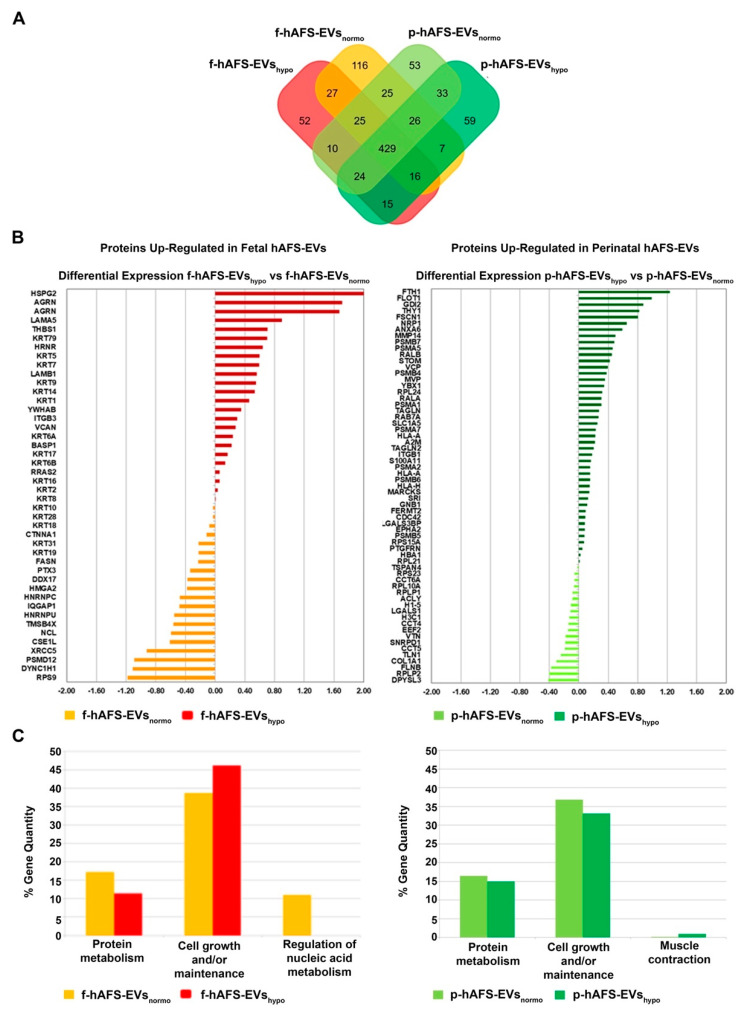
Comparative proteomics analysis of fetal- and perinatal hAFS-EVs. (**A**) Venn diagram illustrating the distribution of proteins identified with a frequency of at least 2 within f-hAFS-EVs_normo_ (dark yellow), f-hAFS-EVs_hypo_ (red), p-hAFS-EVs_normo_ (light green) and p-hAFS-EVs_hypo_ (dark green). (**B**) Differentially expressed proteins identified in fetal hAFS-EVs (left panel) and perinatal hAFS-EVs (right panel) by label-free quantification with MAProMa software. Left panel: histogram reporting the differential expression of proteins found upregulated between normoxic control (dark yellow bars and negative DAve values) and hypoxic preconditioning (red bars and positive DAve values) of f-hAFS-EVs over p-hAFS-EVs. Right panel: histogram reporting the differential expression of proteins found upregulated between control normoxic (light green bars and negative DAve values) and hypoxic preconditioning (dark green bars and positive DAve values) of p-hAFS-EVs over f-hAFS-EVs. Proteins with DAve (ratio of protein expression) ≥ |0.4| and a DCI (confidence of differential expression) ≥ |5| passed the filters and were considered differentially expressed; see Table S3 for the complete list and detailed parameters of the reported proteins. (**C**) Biological processes enrichment analysis of proteins identified with a frequency of at least 2 in f-hAFS-EVs (left panel) and p-hAFS-EVs (right panel) after hypoxic preconditioning. Based on FunRich tool, gene ontology terms are shown in bar charts reporting the percentage of genes enriched for each category (dark yellow bars for f-hAFS-EVs_normo_, red bars for f-hAFS-EVs_hypo_, light green bars for p-hAFS-EVs_normo_ and dark green bars for p-hAFS-EVs_hypo_). Only gene ontology terms with Bonferroni corrected * *p* < 0.05 are reported.

The application of linear discriminant analysis (LDA [51]) on this master list allowed to extract statistically significant proteins (F ratio ≥ 4.5 and ** *p* < 0.001) to be processed by hierarchical clustering. Figure S1A shows a clear separation and different behavior between hAFS-CM and hAFS-EV fractions generating two main branches, as highlighted by the heatmap color code. A further subgrouping was also observed according to the gestational age and the hypoxic preconditioning adopted. The fact that each analyzed condition presented a unique identity is confirmed by the Venn diagrams (Figure 5A and Figure 6A, Tables S1–S3) that report the distribution of proteins identified with a frequency >1 in hAFS-CM and hAFS-EV formulations considered separately. While about 69.5% and 69.9% of proteins was shared among hAFS-EVs and hAFS-CM conditions respectively, the remaining content appeared exclusive in different proportions, ranging from 3.7% to 13.4%, among the formulations.

To quantitatively examine the proteomic changes, a label-free differential analysis was performed by using the home-made MAProMa software and applying two algorithms, DAve (Differential Average) and DCI (Differential Confidence Index, representing the ratio and the confidence in differential expression, respectively), on the aPSMs of each single protein between the two compared terms. Using stringent filters for DAve and DCI to maximize the confidence of identification and to consider proteins with a variation greater than a fold change of 1.5, pairwise comparisons of f-hAFS-CM versus p-hAFS-CM and of f-hAFS-EVs versus p-hAFS-EVs were made according to cell gestational stage. A total of 58 and 109 proteins were found differentially expressed in the aforementioned hAFS-CM and hAFS-EV compartments, respectively, (Figure S1B,C for selected details and Tables S2–S3 in extended form). Among these, 30 proteins resulted up-regulated in f-hAFS-CM and 28 were up-regulated in p-hAFS-CM (Figure S1B); likewise, 44 distinct proteins resulted upregulated in f-hAFS-EVs and 65 were up-regulated in p-hAFS-EVs (Figure S1C). Notably, proteins that resulted up-regulated in f-hAFS are to be considered down-regulated in p-hAFS and vice versa.

Furthermore, as from the bar charts in Figure 5B and Figure 6B, the distinctive protein distribution according to the secreting cell hypoxic preconditioning is appreciable. In this case, for complete information, proteins that do not exceed the threshold of DAve and DCI set were also reported. Fetal hAFS secretome resulted enriched with the 60 kDa heat Shock Protein (HSPD1, Figure 5B, left panel) in its hypoxic formulation, while the perinatal counterpart highly expressed smooth muscle cell contractile myosin regulatory [52] light polypeptide 9 (MYL9, Figure 5B, right panel). An important share of the difference between gestational stage seems to depend more on hypoxic preconditioning in hAFS-EVs; f-hAFS-EVs obtained from hypoxic cell priming were found enriched with factors including Perlecan (HSPG2), Agrin (AGRN), Laminin Subunit α−5 and β-1 (LAMA5 and LAMB1), Thrombospondin-1 (THBS1, Figure 6B, left panel). Hypoxic p-hAFS-EVs contained Ferritin Heavy Chain (FTH1), scaffolding proteins like Flotillin-1 (FLOT1), Fascin (FSCN1), Annexin A6 (ANXA6), Rab GDP dissociation inhibitor beta (GDI2), along with Thy-1 membrane glycoprotein (THY1), Neuropilin-1 (NRP1) and Matrix Metalloprotein 14 (MMP14, Figure 6B, right panel).

Proteins found with a frequency of at least 2 in each examined condition in f-hAFS-EVs and p-hAFS-EVs were further compared to Vesciclepedia database [53]. As expected, the majority of the identified proteins (96%) have been previously described in EVs and exosomes in the reference database (Figure S3A). In this respect, Gene Ontology (GO) enrichment analysis was performed by means of FunRich [54]. The abundance of GO terms in the dataset was compared against their natural amount in the reference database to find statistically over-represented groups of proteins, according to their involvement in biological processes, molecular function and cellular components (for this last aspect data are not shown, but available on request). Regarding the analysis of the molecular functions associated with identified proteins, hAFS-CM fractions indicated an enrichment in structural constituent of extracellular matrix and cytoskeleton, cytoskeletal protein binding and structural molecule activity (Figure S2), while hAFS-EVs were enriched with structural constituent of cytoskeleton and ribosome, DNA and RNA binding and GTPase and chaperone binding factors (Figure S3C).

Biological processes enrichment analysis for both hAFS-CM and hAFS-EVs indicated that the majority of proteins modulated in the fetal- and perinatal hAFS secretome fractions belong to cell growth/maintenance and protein metabolism (Figure 5C and Figure 6C). Within hAFS-EVs we noticed that the term “extracellular matrix structural constituents” exclusively associated to hypoxic f-hAF-EVs; the terms “calcium ion binding” and “structural molecular activity” were mainly enriched in hypoxic f-hAFS and p-hAFS samples (Figure S3B).

### 2.6. The Cytokine and Chemokine Profiling of Fetal vs. Perinatal hAFS-CM and hAFS-EVs Revealed Different Distribution Patterns

**Figure 7:**
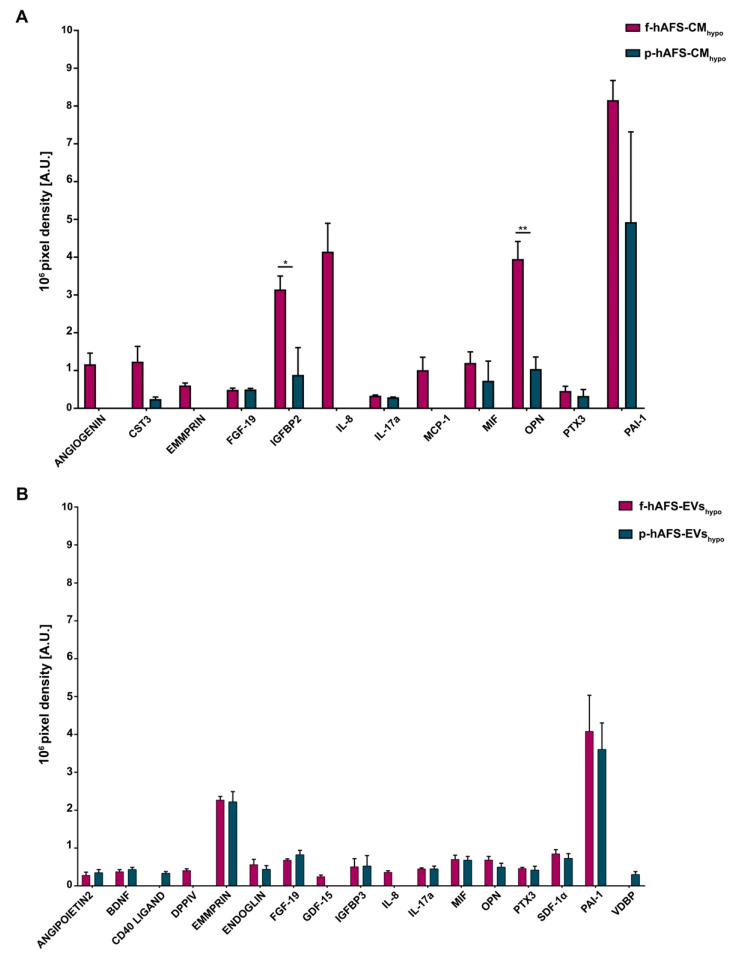
Cytokine and chemokine profiling within fetal- and perinatal hAFS secretome formulations. (**A**) Expression of cytokines and chemokines detected within the hypoxic fetal- versus perinatal hAFS-CM (f-hAFS-CM_hypo_ vs p-hAFS-CM_hypo_) are reported in pixel density by arbitrary unit [A.U.]. Values are expressed as mean ± s.e.m of *n* = 3 independent experiments and reported in Table S4; * *p* = 0.0485; ** *p* = 0.006. (**B**) Cytokine and chemokine content detected in the hypoxic fetal- versus perinatal hAFS-EVs (f-hAFS-EVs_hypo_ vs p-hAFS-EVs_hypo_) and expressed by pixel density in arbitrary units [A.U.]. Values are expressed as mean ± s.e.m of *n* = 3 independent experiments and reported in Table S5. *CST3*: *Cystatin C*; *EMMPRIN*: *Extracellular Matrix MetalloPRoteinase Inducer*; *FGF-19*: *Fibroblast Growth Factor-19*; *IGFBP2*: *Insulin-like Growth Factor (IGF) Binding Protein 2*; *IL-8*: *Interleukin-8*; *IL-17a*: *Interleukin-17a*; *MCP-1*: *Monocyte Chemoattractant Protein-1*; *MIF*: *Macrophage migration Inhibitory Factor*; *PTX3*: *Pentraxin 3*; *PAI-1*: *Plasminogen Activator Inhibitor-1*; *BDNF*: *Brain-Derived Neurotrophic Factor*; *DPPIV*: *DiPeptidyl Peptidase IV*; *GDF-15*: *Growth Differentiation Factor-15*; *IGFBP3*: *Insulin-like Growth Factor (IGF) Binding Protein 3*; *SDF-1α*: *Stromal Derived Factor-1 alpha*; *VDBP*: *Vitamin D-Binding Protein*.

We have previously validated the regenerative capacity of f-hAFS-CM_hypo_ on injured cardiovascular cells via paracrine effects [34,35,49]. Here we compared the cytokine and chemokine content of f-hAFS-CM_hypo_ to the corresponding p-hAFS counterpart (Figure 7A, Figure S4A and Table S4) and found some discriminating factors.

ANGIOGENIN, Extracellular Matrix Metalloproteinase Inducer (EMMPRIN), Interleukin 8 (IL-8) and Monocyte Chemoattractant Protein-1 (MCP-1) were found exclusively enriched inf-hAFS-CM_hypo_ and were not detected in p-hAFS-CM_hypo_. Insulin-like Growth Factor Binding Protein 2 (IGFBP2) and Osteopontin (OPN) were significantly increased in f-hAFS-CM_hypo_ over p-hAFS-CM_hypo_ by 3.5- and 3.8-fold (* *p* < 0.05 and ** *p* < 0.01 respectively, Figure 5A). Plasminogen Activator Inhibitor-1 (PAI-1) was strongly expressed in both f-hAFS-CM_hypo_ and p-hAFS-CM_hypo_ with (Figure 7A). Other cytokines were detectable at low levels, namely Cystatin C (CST3), Fibroblast Growth Factor 19 (FGF-19), Interleukin-17a (IL-17a), Macrophage Migration Inhibitor Factor (MIF), Pentraxin 3 (PTX3).

While fetal- versus perinatal hAFS-CM showed differential expression in their cytokine and chemokine profile, the corresponding fetal versus perinatal EV counterparts were more homogeneously distributed, although with lower expression profiles (Figure 7B, Figure S4B and Table S5). Nonetheless, some differences could be appreciated: DiPeptidyl-Peptidase IV (DPPIV), Growth/differentiation factor 15 (GDF-15) and IL-8 were expressed only by f-hAFS-EVs_hypo_, although at low levels; ANGIOPOIETIN 2, CD40 LIGAND and Vitamin D-Binding Protein (VDBP) were found only in p-hAFS-EVs_hypo_, despite once again detected in low amounts. Other cytokines such as Brain-derived Neurotrophic Factor (BDNF), ENDOGLIN, FGF-19, Insulin-like Growth Factor Binding Protein 3 (IGFBP3), IL-17a, MIF, OPN, PTX3, Stromal Derived Factor-1 alpha (SDF-1α), were found in both f-hAFS-EVs_hypo_ and p-hAFS-EVs_hypo_, with PAI-1 and EMMPRIN being more highly expressed (Figure 7B).

BDNF, ENDOGLIN, IGFBP3 and SDF-1α were exclusively enriched in all hypoxic hAFS-EVs compared to the corresponding hAFS-CM, regardless of gestational stage. Moreover, while EMMPRIN was not detected within p-hAFS-CM_hypo_, it was found enriched in the EV corresponding fraction; conversely OPN was more abundant in f-hAFS-CM_hypo_ than in f-hAFS-EVs_hypo_, while it was comparable among the corresponding p-hAFS secretome fractions. FGF-19, MIF and PTX3 were similarly expressed in both fetal- and perinatal hAFS-CM and in the corresponding hAFS-EVs. PAI-1 was highly enriched in alhypoxic secretome fractions.

### 2.7. Fetal- and Perinatal hAFS-EVs Are Enriched with RNA Information in Their Cargo

**Figure 8:**
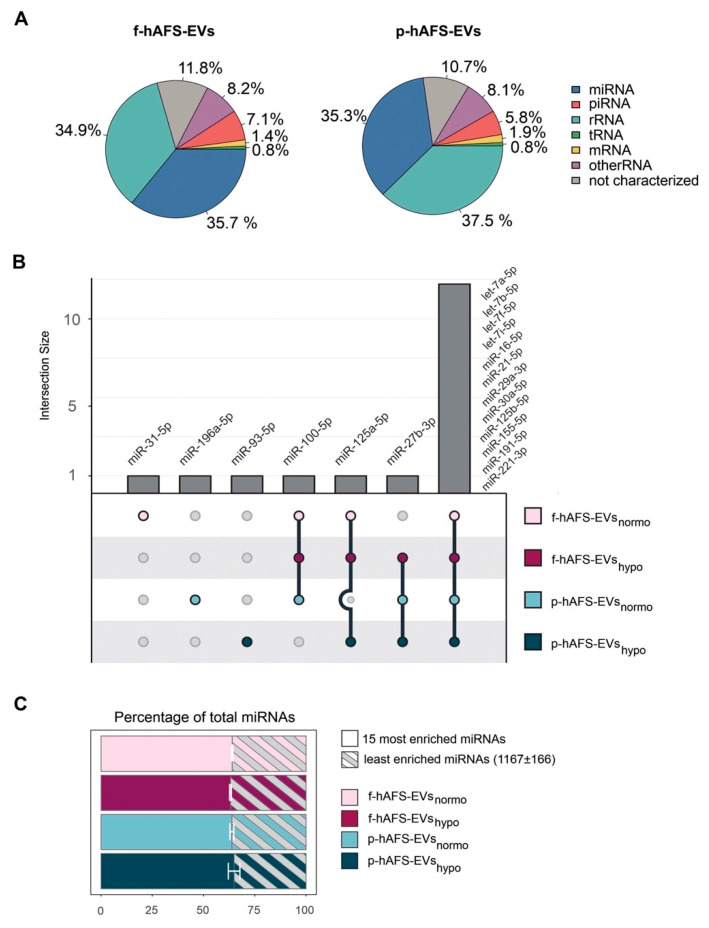
RNA cargo profiling of fetal- and perinatal hAFS-EVs. (**A**) Relative percentage distribution of RNA types detected within fetal- vs perinatal hAFS-EVs (f-hAFS-EVs and p-hAFS-EVs, respectively). Slice of pie chart referring to average percentage of RNA amount in f-hAFS-EVs (left panel) and p-hAFS-EVs (right panel) of *n* = 3 experiments. (**B**) Upset plot of miRNAs enriched in hAFS-EVs according to gestational stage and cell preconditioning. (**C**) The 15 most enriched miRNAs within f-hAFS-EVs_normo_ (64.47 ± 0.28%), f-hAFS-EVs_hypo_ (63.84 ± 0.43%); p-hAFS-EVs_normo_ (64.08 ± 0.92%) and p-hAFS-EVs_hypo_ (65.78 ± 2.82%); values are expressed as mean percentage ± s.e.m of *n* = 3 experiments.

Since small non-coding RNAs have been considered master regulators of EV paracrine influence on target cells [4,55], we mainly focused RNA sequencing analysis on microRNA (miRNA) content within f-hAFS-EVs and p-hAFS-EVs. Small RNA profiling showed enrichment of miRNA in both fetal- and perinatal hAFS-EVs (around 35–36%), when compared to the total small RNA amount (Figure 8A). The miRNA component was indeed among the two most represented RNA species in both EV formulations, together with rRNA (**** *p* < 0.0001). The following miRNAs were the most highly enriched in the EV samples analysed: *miR-31-5p*; *miR-196a-5p*; *miR-93-5p*; *miR-100-5p*; *miR-125a-5p*; *miR-27b-3p*, *let-7a-5p*, *let-7b-5p*, *let-7f-5p*, *let-7i-5p*, *miR-16-5p*, *miR-21-5p*, *miR-29a-3p*, *miR30a-5p*, *miR-125b-5p*, *miR-155-5p*, *miR-191-5p and miR-221-3p*. Of note, fetal- and perinatal hAFS-EVs shared the majority of such miRNAs (namely *let-7a-5p*, *let-7b-5p*, *let-7f-5p*, *let-7i-5p*, *miR-16-5p*, *miR-21-5p*, *miR-29a-3p*, *miR30a-5p*, *miR-125b-5p*, *miR-155-5p*, *miR-191-5p* and *miR-221-3p*, Figure 7B). The 15 mostly enriched miRNAs species covered more than 60% of total miRNA content in each sample. On the other hand, about 1000 miRNAs were found in the remaining 30% of the vesicular miRNA content (Figure 8C).

**Figure 9:**
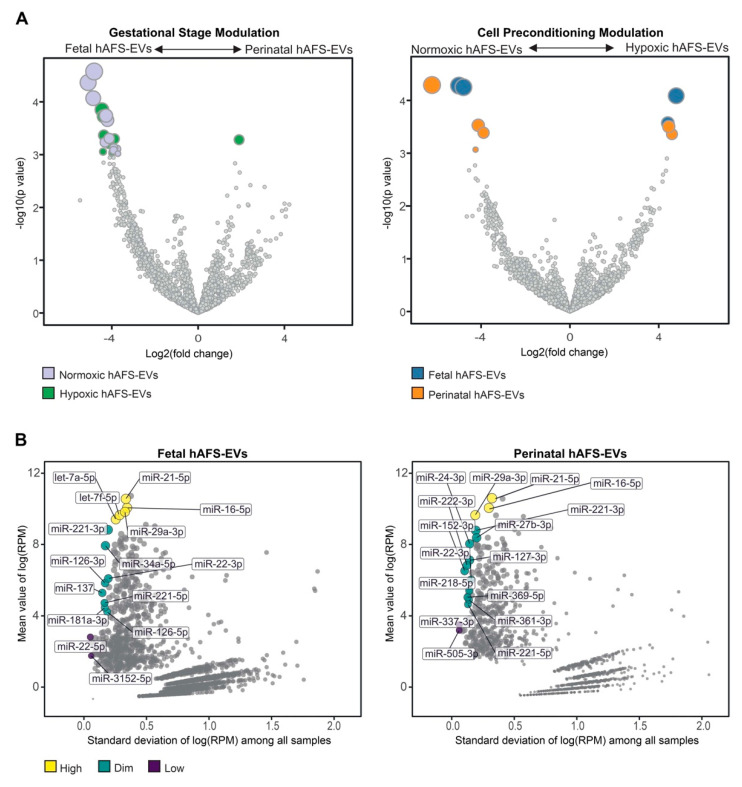
Analysis of differential enrichment of miRNAs in fetal- and perinatal hAFS-EVs. (**A**) Volcano plots for differential enrichment of hAFS-EV miRNA cargo according to gestational stage (left panel) and in vitro hypoxic preconditioning of secreting cells (right panel). For miRNA details, please refer to Table 1. (**B**) Scatter plot of the correlation between variability (X axis) and enrichment level (Y axis) of stable miRNAs within fetal hAFS-EVs (left panel) and perinatal hAFS-EVs (right panel) according to high (yellow dots), dim (green dots) and low (dark purple dots) enrichment. For miRNA details, please refer to Table 2. *RPM: reads per million*.

To further characterize the miRNA content within hAFS-EVs, we investigated whether the hAFS gestational stage or in vitro hypoxic cell preconditioning could influence enrichment of specific miRNAs. The strongest modulation was found between gestational stages, where almost all modulated miRNAs were enriched in f-hAFS-EVs over the perinatal counterpart (Figure 9A and Table 1). Hypoxic preconditioning had a milder effect on miRNA cargo, where in this comparison modulation was in either direction, with some miRNA were enriched in hypoxic- and others in normoxic control conditions (Figure 9A).

As a complementary analysis, we focused on the identification of investigated miRNAs with the lowest variability across the different donors and culture preconditioning, for EVs derived from both investigated gestational stages. miRNAs resulting from this analysis spanned from high to low expression levels (Figure 9B). Within the most stable miRNA core of hAFS-EVs cargo, some shared miRNA between f-hAFS-EVs and p-hAFS-EVs were identified (*miR-21-5p, miR-29a-3p, miR-16-5p* at high level; *miR-221-3p, miR-221-5p and miR-22-3p* at dim level, Table 2).

## 3. Discussion

Left over discarded samples of human amniotic fluid have been identified as a valuable source of stromal cells with promising potential in regenerative medicine and tissue engineering. Ethical concerns associated with their isolation are minimal, since they can be obtained from either left over samples of routine prenatal screening amniocentesis, during the II trimester of gestation (fetal hAFS), or from amniotic fluid discarded as clinical waste in III trimester scheduled C-section procedures (perinatal hAFS). In recent years, hAFS have been proposed as potential therapeutics for human tissue repair and regeneration given the encouraging evidence obtained from experimental disease models. Interestingly, they have been also proposed for in utero therapy of fetal-neonatal neurological diseases; indeed, preclinical studies suggested that hAFS administered prenatally via intra-amniotic delivery safeguarded the spinal cord during gestation via paracrine activity in a rat model of myelomeningocele [56,57,58], and reduced the damage of exposed bowel in experimental rodent gastroschisis [59]. From a translational perspective, in utero transplantation of hAFS could be replaced by administration of the most suitable preparation of their secretome (hAFS-CM o hAFS-EVs). This strategy would allow prompt and timely intervention during gestation by overcoming limitations of canonical cell therapy (i.e., time-consuming in vitro cell expansion), while providing off-the shelf and ready-to-use pharmaceutical formulations.

The recent development of less invasive prenatal diagnostic techniques may result in a decrease in amniocentesis procedures in the near future, thus advocating perinatal hAFS as the more accessible option. Nevertheless, since fetal hAFS are more developmentally immature, they may harbor a more effective paracrine potential. Within this scenario, here we compared fetal- and perinatal c-KIT^+^ hAFS and we focused on profiling their secretome fractions. We highlighted relevant distinctions to be taken into consideration for the possible clinical translation of their paracrine capacity.

In agreement with previous independent studies we show that gestational stage did not influence the heterogeneous hAFS morphology and their mesenchymal antigen profile [25,26]. We then evaluated parameters more likely to impact cell secretory and paracrine activity beyond canonical stromal immunophenotype. Notably the presence of a CD146-positive, CD107a-high subpopulation within bone-marrow mesenchymal progenitors has been recently shown to correlate with remarkable modulatory and therapeutic paracrine activity [47]. Here we revealed that both fetal- and perinatal hAFS are strongly characterized by this molecular signature supporting their secretory potency with relevant translational implications. Moreover, fetal hAFS were characterized by inefficient aerobic metabolism, while more mature perinatal ones showed higher oxygen consumption rate and ATP synthesis. This may suggest a more immature metabolic profile of II trimester hAFS that resembles umbilical cord stromal cells of preterm newborns, which have shown the same trend [60].

In order to trigger paracrine potential, hAFS were exposed to 24 h serum-free hypoxic priming, a strategy we have previously successfully developed [34,35,37] for fetal cells and that here we investigated on their perinatal counterpart for the first time. Preconditioning fetal- and perinatal hAFS under hypoxia resulted in a positive trend in the increase of their secretome concentration and in the amount of EVs released, whereas gestational stage did not exert any effect on the cell secretome yield nor on EV morphology and size distribution.

Notably, characterization of the hAFS paracrine cargo revealed some specific differences, according to the difference conditions we evaluated. The proteomic profiling of the fetal hAFS secretome revealed discernible factor distributions based on gestational stage and cell hypoxic preconditioning. This indicates that the hAFS paracrine potential can acquire a distinct identity during maturation from II to III gestation trimester that in turn can be modulated by stimulating the secreting cells in vitro. Biological processes enrichment analysis of hAFS-CM and hAFS-EVs suggested that most of the modulated proteins may concur to cell growth/maintenance and protein metabolism, thus supporting the cell beneficial paracrine effects reported so far. In particular, the hypoxic fetal hAFS total secretome was found enriched with the heat shock protein HSPD1 (HSP60), which was demonstrated to support wound healing in a diabetic mouse skin injury model and to promote macrophage pro-resolving skewing into M2 phenotype [61]. Likewise, hypoxic fetal hAFS-EVs were enriched for factors promoting neurogenesis (HSPG2 [62]), cell self-renewal and brain and cardiovascular development (LAMA5 [63] and LAMB1 [64]) and migration (THBS1 [2]). The proteoglycan AGRN was also found in EVs following hypoxic priming of fetal hAFS. Our findings are in line with previous evidence of AGRN being upregulated in the proteome of mesenchymal stromal cells under inflammatory and hypoxic instructive stimuli [65]. AGRN has also been shown to be implicated in immune synapse signaling [66] and to concur to neonatal mouse heart regeneration [67], thus supporting a pronounced predisposition of developmentally young fetal hAFS towards regenerative paracrine effects. Moreover, fetal hAFS confirmed to be more responsive to hypoxic preconditioning as shown by enrichment of predictors of vascular regenerative efficacy, such as ANGIOGENIN, EMMPRIM, IL-8 and MCP-1 cytokine [68], in their conditioned medium. This supports previous evidence of the paracrine potency of fetal hAFS-CM in boosting endogenous neo-arteriogenesis in preclinical rodent models of myocardial infarction, hind-limb ischemia, and ischemic fasciocutaneous flap [34,69,70,71]. Furthermore, the fetal hAFS total secretome was found significantly more enriched with IGFBP2 and OPN when compared to the perinatal one, thus suggesting a more pronounced pro-resolving and anti-aging modulatory profile [72,73,74,75]. The perinatal hAFS-CM, while being less enhanced in paracrine factors, was similarly supplemented with neurotrophic and immunomodulatory factors, such as CST3 [76,77] and MIF [78].

Compared to total hAFS-CM, the corresponding hypoxic fetal- and perinatal- EV counterparts showed lower expression of cytokines and chemokines, with the exception of the vascular remodeling mediator EMMPRIN [79], which was mostly enriched in the vesicle compartment. The previously reported cardio-active and pro-regenerative profile of fetal hAFS-EVs [34,37] has been confirmed herein by evidence of their exclusive expression of cardioprotective IL-8 [80] and GDF-15, a key paracrine factor triggering endogenous adult hippocampal neurogenesis [81,82], as well as counteracting anthracycline-induced cardiotoxicity [78]. Both fetal- and perinatal hAFS-EVs showed similar expression for the progenitor/stem cell trafficking regulator SDF-1α [83,84,85]. Proteomic analysis reported increased expression of proteins related to angiogenesis, like NRP1 [86] and MMP14 [87] in hypoxic perinatal hAFS-EVs; such stimulatory profile may explain previous results on the endothelial regenerative properties of III trimester hAFS in a preclinical mouse model of skeletal muscle ischemic injury [25], despite the evidence of their hAFS-CM being less pro-angiogenic than the corresponding fetal one. Notably, the neural growth factor BDNF was found in both fetal- and perinatal EV cargo, although in low amounts, thus suggesting a putative neurotrophic activity for hAFS-EVs in neuronal survival and neurodevelopmental processes, as also observed for extracellular vesicles secreted by human bone marrow- and umbilical cord blood-MSC [88,89]. Both secretome formulations from fetal- and perinatal hAFS undergoing hypoxic stimulation showed to be enriched with PAI-1, a facilitator of endothelial activation [90] that has also been involved in the polarization of M2 macrophages in the heart and endowed with cardioprotective and anti-fibrotic potential [91].

MicroRNAs (miRNAs) have been broadly addressed as crucial regulators of stem cell- and mesenchymal stromal cell-EV paracrine activity [92,93]. Here we found that the 15 mostly enriched miRNA species within the hAFS-EVs cover more than 60% of total miRNA content in each sample. These miRNAs have been reported to characterize the molecular cargo of mesenchymal stromal cell-EVs (*let-7a-5p* [4,94,95]), protect against myocardial ischemia by influencing vascular regeneration and inhibiting fibrosis (*let-7b-5p*, *let-7f-5p*, *miR-21-5p* and *miR-155-5p* [96,97]), promote wound healing by regulating keratinocyte function (*miR-16-5p* [98]) and counteract neuronal death after forebrain ischemia (*miR-29a-3p* [99,100]). On the other hand, about 1000 miRNAs were found in the remaining 30% of vesicular miRNA content. Such unbalanced distribution is consistent with previous studies [4] and highlights the mostly enriched miRNAs as the putative accountable ones for the main biological activity of hAFS-EVs. Interestingly, fetal- and perinatal hAFS-EVs shared the majority of 15 miRNAs. Of note, we also observed that both fetal hAFS-EVs and perinatal ones contained a set of very stable miRNAs across different enrichment levels. Such evidence may suggest a wide range of “housekeeping” candidates to be used as internal reference control in qPCR experiments on hAFS-EVs. Moreover, a consistent subset of such stable miRNAs (*miR-16-5p*, *miR-21-5p*, *miR-22-3p*, *miR-29a-3p*, *miR-221-3p*, *miR-221-5p*) is shared between the two gestational stages and overlaps with the 15 mostly enriched ones, suggesting an even more constant behavior and a reliable pre-resolving molecular signature ([96,98,99,100]). Of note, a couple of candidates within such distinctive core have been recently reported as reference miRNAs within neuroprotective EVs obtained from II trimester amniotic fluid-derived mesenchymal stromal cells (*miR-29a-3p and miR-221-3p* [95]).

Nevertheless, we also noticed that gestational age may modulate the miRNA cargo more than hypoxic preconditioning. Developmentally more juvenile EVs obtained from II trimester fetal hAFS were enriched with miRNAs previously shown to support viability of embryonic stem cells (*miR-302-3p* [101]), cell proliferation and osteogenic differentiation of bone marrow stromal cells (*miR-217* [102]), while also harboring tumor suppressor potential (*miR-302-3p* [103,104]); *miR-383-5p* [105,106]).

Based on our results, here we confirm that fetal- and perinatal hAFS may represent attractive paracrine sources to be exploited for regenerative medicine. While their phenotype and secretory activity were similar, we have highlighted some peculiar aspects in their secretome formulations as useful insights for their future therapeutic translation.

## 4. Materials and Methods

### 4.1. Human Amniotic Fluid Stem Cell Isolation and In Vitro Culture

Human amniotic fluid stem cells (hAFS) were isolated from leftover samples of amniotic fluid (AF) collected by routine prenatal screening via II trimester amniocentesis (fetal hAFS, f-hAFS), or as clinical waste during scheduled cesarean-section delivery during III trimester (perinatal hAFS, p-hAFS) at the Prenatal Diagnosis and Perinatal Medicine Unit, IRCCS San Martino Hospital, at the Fetal- and perinatal Medical and Surgery Unit and Human Genetics Laboratory at IRCCS Istituto Gaslini hospital (Genova, Italy). Informed written consent was obtained from all donors according to local ethical committee authorization (protocol P.R. 428REG2015) and in compliance with Helsinki Declaration guidelines. II trimester fetal AF samples were obtained from female donors with average age of about 37.42 ± 0.32 years old (*n* = 15 ranging from 36- up to 41 years old); III trimester perinatal AF samples were obtained from female donors with average age of 34.25 ± 1.31 years old (*n* = 10 ranging from 26- up to 42 years old). Fetal- and perinatal hAFS were obtained from samples validated for normal karyotype and isolated by immunomagnetic sorting for c-KIT expression (CD117 MicroBead Kit, Miltenyi Biotechnology, Bologna, Italy) from adherent AF mesenchymal stromal cells [16]. c-KIT^+^ hAFS were cultured in Minimal Essential Medium (MEM)-alpha with 15% FBS (Fetal Bovine Serum, Gibco-Thermo Fisher Scientific, Monza, Italy), 18% Chang B and 2% Chang C Medium (Irvine Scientific, Santa Ana, CA, USA) with 1% L-glutamine and 1% penicillin/streptomycin (Gibco-Thermo Fisher Scientific, Monza, Italy), in an incubator at 37 °C with 5% CO_2_ and 20% O_2_ atmosphere and cultured up to 5 passages in vitro before being used to isolate their secretome.

### 4.2. Biochemical Evaluation of hAFS Metabolism

Cell aerobic metabolism was evaluated in terms of oxygen consumption and ATP synthesis through the F_1_-F_o_ ATP synthase. Oxygen consumption rate (OCR) was measured at 37 °C in a closed chamber magnetically stirred using an amperometric electrode (Unisense-Microrespiration, Unisense A/S, Denmark). One hundred thousand (10^5^) cells were used for each experiment. To evaluate basal respiration, hAFS were permeabilized with 0.03 mg/mL digitonin for 10 min and suspended in phosphate buffer saline (PBS). 10 mM pyruvate plus 5 mM malate or 20 mM succinate were added to stimulate the pathways composed by Complexes I, III, and IV or Complexes II, III and IV, respectively [60].

To evaluate the relative contributions to respiration of glutamine, long-chain fatty acid oxidation, and glucose, after the digitonin permeabilization, cells were suspended in growth medium and 4 μM of BPTES, 4 μM Etomoxir, and 4 μM UK5099 were added to inhibit glutaminase, carnitine palmitoyl-transferase 1A (CPT1A), or the mitochondrial pyruvate carrier (MPC), respectively. F_1_-F_o_ ATP synthase (ATP synthase) activity was detected by measuring ATP production by the highly sensitive luciferin/luciferase method. The assays were conducted at 37 °C, for 2 min, and data were collected every 30 s. In a first set of experiments, 10^5^ cells were incubated for 10 min in medium containing 50 mM KCl, 1 mM EGTA, 2 mM EDTA, 5 mM KH2PO4, 2 mM MgCl2, 0.6 mM ouabain, 1 mM P1P5-Di(adenosine-5′) penta- phosphate, 0.040 mg/mL ampicillin, and 10mM Tris-HCl pH7.4. Afterward, ATP synthesis was induced by the addition of the respiratory substrates (10 mM pyruvate + 5 mM malate or 20 mM succinate) and 0.1 mM ADP. The reaction was measured using the luciferin/luciferase ATP bioluminescence assay kit CLSII (Roche, Basel, Switzerland) in a Luminometer (GloMax^®®^ 20/20 Luminometer, Promega, Milan, Italy). ATP standard solutions (Roche, Basel, Switzerland) ranging 10^−10^–10^−7^ M were used for calibration. In the second set of experiments, ATP synthesis was evaluated in the presence of 4 μM BPTES, 4 μM Etomoxir, or 4 μM UK5099. In this case, 10^5^ cells were incubated for 10 min ingrowth medium in the absence or presence of a metabolism inhibitor, and ATP synthesis was induced with 0.1 mM ADP. OxPhos (oxidative phosphorylation) efficiency (P/O ratio) was calculated as the ratio between the concentration of produced ATP and the amount of consumed oxygen in the presence of respiratory substrate and ADP. When oxygen consumption is completely devoted to energy production, the P/O ratio should be approximately 2.5 and 1.5 after pyruvate + malate or succinate addition, respectively [48]. To evaluate the contribution of anaerobic glycolysis to hAFS metabolism, glucose and lactate concentrations were evaluated in the growth medium. Glucose consumption was evaluated by the hexokinase (HK) and glucose-6-phosphate dehydrogenase (G6PD) coupling system, following the reduction of NADP at 340 nm. The assay medium contained 100 mM Tris–HCl, pH 7.4, 2 mM ATP, 10 mM NADP, 2 mM MgCl2, 2 IU of hexokinase, and 2 IU of glucose-6-phosphate dehydrogenase. Lactate release was assayed following the reduction of NAD+ at 340 nm. The assay medium contained 100 mM Tris-HCl (pH 8), 5 mM NAD+, and 1 IU/mL of lactate dehydrogenase. Samples were analyzed before and after the addition of 4 μg of purified lactate dehydrogenase. In both cases, data was normalized to the cell number and expressed as mM glucose/10^6^ cells or mM lactate released/10^6^ cells, respectively [107].

### 4.3. Flow Cytometry Characterization of hAFS

One hundred thousand (10^5^) fetal- and p-hAFS cells were detached and incubated with mouse anti human-CD107a-Alexa Fluor 647- and anti-human CD146-FITC-conjugated antibodies (eBioscience, Thermo Fisher Scientific, Monza, Italy). Cell apoptosis was assessed using a FITC Annexin V Apoptosis Detection Kit (BD Pharmingen, Becton Dickinson, Milan, Italy) following manufacturer’s instructions. Events were acquired on a BD Bioscience FACS Aria II sorter and analyzer, equipped with FACS Diva software (BD Bioscience, Becton Dickinson, Milan, Italy). Data was analyzed using FlowJo V9.0 software (BD Bioscience, Becton Dickinson, Milan, Italy).

### 4.4. Senescence Staining

The senescence phenotype of hAFS cultured up to passage 5 in standard in vitro conditions was evaluated with Senescence β-Galactosidase Staining Kit (Cell Signaling Technology, Danvers, MA, USA): f-hAFS and p-hAFS were fixed with 1x Fixative solution at 70% confluency and stained for SA-β-gal at 37 °C overnight, according to the manufacturer’s instructions. Senescent events were acquired on a Leica DMi1 microscope (equipped with Leica Acquire software V3.4.4, Leica Microsystems, Milan, Italy) and evaluated as a percentage of SA-β-gal-positive cells over total cells per field.

### 4.5. Separation and Concentration of the hAFS Secretome Fractions

f-hAFS and p-hAFS were cultured for 24 h in serum-free medium (SF) in 1% O_2_ hypoxia versus 20% O_2_ normoxia (control), the latter of which was used as as the baseline reference. This preconditioning strategy was used to enhance the release of bio-active paracrine factors, as we previously reported [34,35,37,49]. hAFS were cultured for 24 h in serum-free (SF) medium (high glucose Dulbecco’s Modified Eagle’s Medium, DMEM, with 1% L-glutamine and 1% penicillin/streptomycin, all from Gibco-Thermo Fisher Scientific, Monza, Italy), under normoxic (20% O_2_ and 5% CO_2_ at 37 °C) or hypoxic (1% O_2_ and 5% CO_2_ at 37 °C in CellXpert^®®^ C170i and Galaxy^®®^ 48 R CO_2_ incubators, from Eppendorf, Milan, Italy) conditions.

f-hAFS-CM and p-hAFS-CM were collected and centrifuged at 4 °C at 300× *g* for 10 min and 2000× *g* for 20 min to remove cell debris; hAFS-CM was concentrated using ultrafiltration membranes with a 3kDa selective cut-off (Amicon Ultra-15, Merck Millipore Darmstadt, Germany) at 4 °C at 3000× *g* for 90 min and then further concentrated at 4 °C at 3000× *g* for 30 min. hAFS-EVs were separated and concentrated by serial ultracentrifugation from hAFS-CM. Briefly, hAFS-CM was collected and centrifuged at 4 °C at 300× *g* for 10 min, 2000× *g* for 20 min to remove cell debris. Supernatant was then processed at 10,000× *g* for 40 min. The pellet was discarded and the supernatant was further processed by ultracentrifugation in an Optima L-90K (Beckmann Coulter, Milan, Italy) at 100,000× *g* for 120 min using Beckman Coulter’s swinging-bucket SW55Ti centrifuge rotors. The pellet containing heterogenous hAFS-EVs was washed in PBS with a final centrifugation at 100,000× *g* for 120 min and then resuspended in PBS filtered with a 0,22 µm pore filter membrane. Protein concentrations in hAFS-CM and on the surface of hAFS-EVs was measured using the BiCinchoninic Acid (BCA) assay (Thermo Fisher Scientific, Monza, Italy). Samples were acquired on a Gen5 Microplate Reader at 570 nm to evaluate hAFS-CM and hAFS-EVs yield in terms of µg of solution/10^6^ producing cells.

### 4.6. Characterization of hAFS-EVs by Transmission Electron Microscopy and Nanoparticle Tracking Analysis

Transmission electron microscopy (TEM) analysis was performed on a Hitachi TEM microscope (HT7800 series, Hitachi High Technologies, Monza, Italy). Digital images were taken with a Megaview 3 camera and Radius software (EMSIS, Muenster, Germany). f-hAFS and p-hAFS were fixed in 3.7% paraformaldehyde (PFA) solution diluted 1:1 with hAFS complete medium, washed in 0.1 M cacodylate buffer, and then immediately incubated for 1 h at room temperature in 0.1 M cacodylate buffer containing 2.5% glutaraldehyde (Electron Microscopy Science, Hatfield, PA, USA). Cells pellets were post-fixed in osmium tetroxide for 1h and in a 1% uranyl acetate solution for 1 h. Samples were dehydrated for 24 h at 42 °C and 48 h at 60 °C through a graded ethanol series and embedded in epoxy resin (Poly-Bed; Polysciences Europe GmbH, Minneapolis, Germany). Ultrathin sections (50 nm) were cut with Leica Ultracut microtome (Leica Microsystems, Milan, Italy) and counterstained with a 5% uranyl acetate in 50% ethanol solution. f-hAFS-EVs and p-hAFS-EVs were resuspended in 20 μL PBS solution and fixed by adding an equal volume of 2% paraformaldehyde in 0.1 M phosphate buffer solution (pH 7.4). EVs were then adsorbed for 10 min onto formvar-carbon coated copper grids by floating the grids on 5 μL drops on parafilm. Subsequently, grids with adhering EVs were rinsed in PBS and negatively stained by 2% uranyl acetate solution for 5 min at room temperature. Stained grids were embedded in 2.5% methylcellulose for improved preservation and air dried before examination. Morphometry analysis of hAFS-EVs was measured on 10 randomly taken micrographs at 40.000× *g* magnification. Size was calculated using the arbitrary line function embedded the measurement dialog box of Radius software (EMSIS, Muenster, Germany). To visualize hAFS-EVs size distribution, results were plotted as scatter dot plot and as frequency distribution in which each size is represented as a point along with lines for the median value and the range.

f-hAFS-EVs and p-hAFS-EVs were also analyzed by Nanoparticle Tracking Analysis (NTA) to assess particles released by 10^6^ cells. hAFS-EVs were diluted 1:1000 in PBS solution and acquired on a NanoSight LM10 (Malvern Instruments, Malvern, UK) that recorded at least 3 different frames of 60 s each. Three different acquisitions of each sample were analyzed using the Batch Process option in the software.

### 4.7. LC-MS/MS Analysis of hAFS-CM and hAFS-EVs

#### 4.7.1. In-Solution Digestion

Proteomic analysis was performed on 3 biological replicates of hAFS-CM and hAFS- EVs from f-hAFS and p-hAFS after normoxic or hypoxic preconditioning (*n* = 24 different conditions). hAFS-CM and hAFS-EVs samples were suspended in 0.1M NH_4_HCO_3_ pH 7.9 and treated with Rapigest™ SF reagent (Waters Co, Milford, MA, USA) at the final concentration of 0.25% (*w*/*v*). The resulting suspensions were incubated while stirring at 100 °C for 20 min. The digestion was carried out on each sample by adding Sequencing Grade Modified Trypsin (Promega Inc., Madison, WI, USA) at an enzyme/substrate ratio of 1:50 (*w*/*w*) overnight at 37 °C in 0.1 M NH_4_HCO_3_ pH 7.9 buffer with 10% CH_3_CN. An additional aliquot of trypsin (1:100 *w*/*w*) was added in the morning, and the digestion continued for 4h. Moreover, the addition of 0.5% Trifluoroacetic acid (TFA) (Sigma-Aldrich Inc., St Louis, MO, USA) stopped the enzymatic reaction, and a subsequent incubation at 37 °C for 45 min completed the RapiGest acid hydrolysis [108]. The water immiscible degradation products were removed by centrifugation at 13,000 rpm for 10 min. Finally, the tryptic digest mixtures were desalted using Pierce™ C-18 spin columns (Thermo Fisher Scientific, Monza, Italy), according to the manufacturer protocol, and were resuspended in 0.1% formic acid (Sigma-Aldrich Inc., St. Louis, MO, USA) in water (LC-MS Ultra CHROMASOLV™, Honeywell Riedel-de Haen™, Muskegon, MI, USA) at a concentration of 0.1 µg/µL.

#### 4.7.2. Liquid Chromatography

Trypsin digested mixtures were analyzed by means of a platform consisting of a nano-liquid chromatographic system, Eksigent nanoLC-Ultra^®®^ 2D System (Eksigent, part of AB SCIEX Dublin, Dublin, CA, USA) configured in trap-elute mode, coupled with a high-resolution mass spectrometer. Briefly, samples (0.8 µg injected) were first loaded on a peptide trap (200 µm × 500 µm ChromXP C18-CL, 3 µm, 120 Å) and washed with the loading pump running in isocratic mode with 0.1% formic acid in water for 10 min at a flow of 3 µL/min. The automatic switching of a ten-port valve then eluted the trapped mixture on a nano-reversed phase column (75 µm × 15 cm ChromXP C18-CL, 3 µm, 120 Å) through a 150 min gradient of eluent B (eluent A, 0.1% formic acid in water; eluent B, 0.1% formic acid in acetonitrile) at a flow rate of 300 nL/min. In depth, gradient was: from 5–10% B in 3min, 10–40% B in 130 min, 40–95% B in 10 min and holding at 95% B for 7 min.

#### 4.7.3. Mass Spectrometry

MS/MS analyses were performed on an LTQ-OrbitrapXL mass spectrometer (Thermo Fisher Scientific, Monza, Italy) equipped with a nanospray ion source. The spray capillary voltage was set at 1.7 kV and the ion transfer capillary temperature was held at 220 °C. Full MS spectra were recorded over a 400–1600 m/z range in positive ion mode, with a resolving power of 60000 (full width at half-maximum) and a scan rate of 2 spectra/s. This step was followed by five low-resolution MS/MS events that were sequentially generated in a data-dependent manner on the top five ions selected from the full MS spectrum (at 35% collision energy), using dynamic exclusion of 0.5 min for MS/MS analysis. Mass spectrometer scan functions and high-performance liquid chromatography solvent gradients were controlled by the Xcalibur data system version 1.4 (Thermo Fisher Scientific, Monza, Italy).

#### 4.7.4. Proteomic Data Processing and Data Mining

All generated data were searched using the Sequest HT search engine contained in the Thermo Scientific Proteome Discoverer software, version 2.1. The experimental MS/MS spectra were correlated to tryptic peptide sequences by comparison with the theoretical mass spectra obtained by in silico digestion of the Uniprot Homo Sapiens proteome database (74600 entries), downloaded in January 2020 (www.uniprot.org, accessed on 10 March 2021). The following criteria were used for the identification of peptide sequences and related proteins: trypsin as enzyme, three missed cleavages per peptide, mass tolerances of ±50 ppm for precursor ions and ±0.8 Da for-fragment ions. Percolator node was used with a target-decoy strategy to give a final false discovery rates (FDR) at Peptide Spectrum Match (PSM) level of 0.01 (strict) based on q-values, considering maximum deltaCN of 0.05 [109]. Only peptides with minimum peptide length of six amino acids and rank 1 were considered. Protein grouping and strict parsimony principle were applied. The MS data have been deposited to the ProteomeXchange Consortium via the PRIDE [110] partner repository (ftp://massive.ucsd.edu/MSV000087013/, accessed on 10 March 2021). The 48 proteins obtained from the SEQUEST algorithm were aligned, normalized and label-free compared. An in-house algorithm, namely, the Multidimensional Algorithm Protein Map (MAProMa) was employed to this aim, using the average peptide spectrum matches (aPSM) [111,112] that correspond to the average of all the spectra identified for a protein and, consequently, to its relative abundance, in each analyzed condition. In depth, to select differentially expressed proteins, subgroups (for both fetal- vs perinatal- hAFS-CM and hAFS-EVs, considering also hypoxic cell preconditioning stimulation), were pairwise compared applying a threshold of 0.4 and 5 on the two MAProMa indexes DAve (Differential Average) and DCI (Differential Confidence Index), respectively. DAve, which evaluates changes in protein expression, was defined as (X − Y)/ (X + Y)/0.5, while DCI, that evaluates the confidence of differential expression, was defined as (X + Y) × (X − Y)/2. The X and Y terms represent the PSM of a given protein in two compared samples. In addition, the average protein lists, obtained from each examined condition, were subjected to linear discriminant analysis (LDA) and proteins with the largest F ratio (≥4.5) and smallest *p* value (≤0.001) were retained and processed by hierarchical clustering, applying Ward’s method and the Euclidean’s distance metric using JMP 15.2 software. Specifically, the F ratio represented the model mean square divided by the error mean square, whereas the *p*-value indicated the probability of obtaining an F value greater than that calculated if, in reality, there was no difference between the population group means.

### 4.8. Cytokine and Chemokine Profiling of hAFS-CM and hAFS-EVs

Cytokine and chemokine profiling of hAFS-CM and hAFS-EVs obtained by f-hAFS and p-hAFS after hypoxic preconditioning was assessed by means of Proteome Profiler™ Human XL Cytokine Array kit (R&D System, Minneapolis, MN, USA) according to the manufacturer’s instructions. Twenty μg of hAFS-CM and hAFS-EVs sample were used. Membranes images were acquired by a Chemidoc Mini HD9 Auto (Uvitec Cambridge, UK). Specific cytokine/chemokine content was evaluated by the quantification of positive pixel intensity (by means of arbitrary unit) for each detectable cytokine using ImageJ software (available at https://imagej.nih.gov/ij/, accessed on 10 March 2021 [113]).

### 4.9. RNA Extraction from hAFS-EVs and Next Generation Sequencing

RNA was isolated from f-hAFS-EVs and p-hAFS-EVs with miRNeasy Micro Kit (Qiagen, Milan, Italy) according to the manufacturer’s instructions. RNA integrity and size distribution were evaluated using the Agilent Small RNA Kit with the small noncoding RNA chip in order to assess the content of small RNAs ranging from 6 to 150 nucleotides (nt). The Qubit microRNA Assay Kit (Thermo Fisher Scientific, Monza, Italy) was used to quantify microRNAs (miRNAs) content, following the manufacturer’s instructions. miRNA sequencing libraries were prepared and amplified using QIAseq miRNA Library kit (Qiagen, Milan, Italy) using 18.5 ng of isolated miRNAs as input and following the manufacturer’s instructions. Libraries were pooled after quality check and quantification by TapeStation (Agilent Technologies, Foster City, CA, USA) was performed using Agilent High Sensitivity D1000 ScreenTape. Pooled libraries were assessed for quality control by real-time qPCR following “Sequencing Library qPCR Quantification” Guide (Illumina Inc., San Diego, CA, USA) and sequenced by Illumina NextSeq platform using High Hutput Hit v2.5 (75 cycles) (Illumina Inc., San Diego, CA, USA). Base calling was performed with default Illumina NextSeq500 workflow.

### 4.10. Bioinformatic Data Analysis of miRNA Sequencing

Fastq files were first processed by trimming off the 3′ adapter and low-quality bases using Cutadapt [114]. Following trimming, the insert sequences and UMI sequences were identified. Reads with no adapter sequence, reads with less than 16 bp insert sequences and Reads with less than 10 bp UMI sequences were discarded. To annotate the insert sequences, reads were aligned to GRCh38 human genome assembly using Bowtie [115]. For each sample all reads assigned to a particular miRNA were counted, and the associated UMIs were aggregated to count unique molecules. Secondary analysis was performed by custom R scripts available upon reasonable request. Differential enrichment analysis was performed using Limma [116] and EdgeR Bioconductor packages [117].

### 4.11. Statistical Analyses

Results are presented as mean ± s.e.m of at least three (*n* = 3) independent experiments. Comparisons were drawn by one-way ANOVA followed by post-hoc Tukey’s multiple comparisons test or by Student’s *t*-test. Analyses were performed using Graph-Pad Prism Version 8.0.2 (GraphPad Software, https://www.graphpad.com, accessed on 10 March 2021) with statistical significance set at * *p* < 0.05. For proteomics analysis, the distribution of proteins in the examined conditions, functional enrichment analysis, and comparison of data versus Vesiclepedia database (http://microvesicles.org, accessed on 10 March 2021) were achieved using FunRich (version 3.1.3, http://www.funrich.org, accessed on 10 March 2021 [54]), that uses hypergeometric test and Bonferroni for statistics and allows the graphical visualization of data with Venn and bar charts [118].

## 5. Conclusions

In conclusion, fetal- and perinatal hAFS were found phenotypically equivalent with comparable secretory potency and EV enrichment in size and distribution; yet some distinctions in their secretome profile could be appreciated. Specifically, the developmentally immature profile of fetal hAFS may be recapitulated by their secretome formulations endowed with a more pronounced pro-vasculogenic, pro-regenerative and rejuvenating secretome. However, perinatal hAFS still retain a relevant paracrine profile via the expression of factors related to endothelial cell migration, immune-modulatory, anti-inflammatory and neurotrophic potential similar to fetal hAFS. These findings may provide useful insights supporting a future paracrine therapy of injury-related and inflammatory/ischemic-based disease. Therefore, the selection of either fetal or perinatal hAFS as the most ideal cell source should be evaluated considering the specific clinical scenario.

## Figures and Tables

**Table 1:** miRNAs modulated within hAFS-EVs.

	Fetal hAFS-EVs (f-hAFS-EVs)	Perinatal hAFS-EVs (p-hAFS-EVs)
Hypoxic preconditioning	*miR-26a-1-3p*; *miR-4521*; *miR-302b-3p*; *miR-4787-3p*; *miR-3945*; *miR-6748-5p*; *miR-7155-5p*; *miR-6815-3p*; *miR-3124-5p*; *miR-383-5p*; *miR-1277-3p*; *miR-33b-5p*	*miR-4700-3p*; *miR-3135a*
Normoxic control condition	*miR-504-5p*; *miR-217*; *miR-411-3p*; *miR-585-5p*; *miR-5187-5p*; *miR-6751-5p*; *miR-4433b-3p*; *miR-6733-5p*; *miR-6747-5p*; *miR-6766-3p*; *miR-4787-3p*	*miR-765*; *miR-214-3p*; *miR-199a-3p*; *miR-199b-5p*; *miR-6748-5p*

**Table 2:** miRNA core as steadily expressed in the fetal- and perinatal hAFS-EVs.

	Fetal hAFS-EVs (f-hAFS-EVs)	Perinatal hAFS-EVs (p-hAFS-EVs)
High enrichment	*miR-21-5p*; *miR-16-5p*; *miR-29a-3p*; *let-7f-5p*; *let-7a-5p*	*miR-21-5p; miR-16-5p;* *miR-29a-3p*
Dim enrichment	*miR-221-3p*; *miR-34a-5p*; *miR-22-3p*; *miR-126-3p*; *miR-137*; *miR-221-5p*; *miR-181a-3p*; *miR-126-5p.*	*miR-221-3p; miR-27b-3p;* *miR-24-3p; miR-127-3p;* *miR-222-3p; miR-22-3p;* *miR-152-3p; miR-218-5p;* *miR-369-5p; miR-361-3p;* *miR-221-5p*
Low enrichment	*miR-22-5p and miR-3152-5p*	*miR-337-3p and miR-505-3p*
Shared in common	*miR-16-5p*; *miR-21-5p*; *miR-22-3p*; *miR-29a-3p*; *miR-221-3p*; *miR-221-5p*	

## Data Availability

Proteomic data are stored in the ProteomeXchange Consortium repository and are available on request at the following url: ftp://massive.ucsd.edu/MSV000087013/, accessed on 10 March 2021. RNAsequencing data was deposited in the Gene Expression Omnibus repository (www.ncbi.nlm.nih.gov/geo/, accessed on 10 March 2021) with accession number: GSE168152.

## Supplementary Materials

The following are available online at https://www.mdpi.com/article/10.3390/ijms22073713/s1.
