# Supplementary material for: Comprehensive Profiling of Secretome Formulations from Fetal- and Perinatal Human Amniotic Fluid Stem Cells
Source: Int J Mol Sci. 2021 Apr 2;22(7):3713. doi: 10.3390/ijms22073713 (PMC8038201; doi:10.3390/ijms22073713)
Supplement: Supplementary file 1 [file ijms-22-03713-s001.zip › Costa A et al_Supplementary Files/Costa A et al_Table S1.docx]

**Table S1.** **Complete list of the distinct master proteins detected with at least one unique peptide in the fetal- and perinatal hAFS-CM and hAFS-EV formulations according to in vitro cell hypoxic preconditioning.** For each protein Uniprot Accession, Reference, Gene name, Coverage, Unique Peptides, MW, pI, average Peptide Spectrum Match (aPSM) and Frequency are reported. Frequency indicates how many times a given protein has been identified in the six replicates (n=3 biological replicates in two independent technical runs) under each examined condition, in hAFS-CM and hAFS-EV compartments and in the 8 examined conditions. aPSM indicates that average values are given for each protein of the same condition.

| **Uniprot Accession** | **Description** | **Gene Name** | **Coverage** | **Unique Peptides** | | **MW [kDa]** | | **pI** | | **hAFS-CM** | | | | | | | | | | | | | | | **hAFS-EVs** | | | | | | | | | | | | | | | | **Overall Frequency** | | | | | | | |  |  |  |
| --- | --- | --- | --- | --- | --- | --- | --- | --- | --- | --- | --- | --- | --- | --- | --- | --- | --- | --- | --- | --- | --- | --- | --- | --- | --- | --- | --- | --- | --- | --- | --- | --- | --- | --- | --- | --- | --- | --- | --- | --- | --- | --- | --- | --- | --- | --- | --- | --- | --- | --- | --- |
|  |  |  |  |  |  |  |  |  |  | **f-hAFS-**  **CMnormo** | | | **f-hAFS-**  **CMhypo** | | | | **p-hAFS-CMnormo** | | | | **p-hAFS-**  **CMhypo** | | | | **f-hAFS-**  **EVsnormo** | | | | **f-hAFS-**  **EVshypo** | | | | **p-hAFS-**  **EVsnormo** | | | | **p-hAFS-**  **EVhypo** | | | |  | | | | | | | |  |  |  |
|  |  |  |  |  |  |  |  |  |  | **aPSM** | **Freq** | | **aPSM** | | **Freq** | | **aPSM** | | **Freq** | | **aPSM** | | **Freq** | | **aPSM** | | **Freq** | | **aPSM** | | **Freq** | | **aPSM** | | **Freq** | | **aPSM** | | **Freq** | | **hAFS-CM** | | | **hAFS-EVs** | | **all groups** | |  |  |  |  |
| P02751 | Fibronectin | FN1 | 44.13 | 75 | | 272.2 | | 5.50 | | 183.98 | 6 | | 138.27 | | 6 | | 176.11 | | 6 | | 162.83 | | 6 | | 156.37 | | 6 | | 325.23 | | 6 | | 170.63 | | 6 | | 161.84 | | 6 | | 4 | | | 4 | | 8 | |  |  |  |  |
| P08670 | Vimentin | VIM | 69.10 | 36 | | 53.6 | | 5.12 | | 78.44 | 6 | | 82.23 | | 6 | | 118.82 | | 6 | | 108.82 | | 6 | | 30.25 | | 6 | | 37.63 | | 6 | | 45.71 | | 6 | | 47.46 | | 6 | | 4 | | | 4 | | 8 | |  |  |  |  |
| P60709 | Actin, cytoplasmic 1 | ACTB | 83.73 | 11 | | 41.7 | | 5.48 | | 77.01 | 6 | | 78.58 | | 6 | | 85.87 | | 6 | | 89.34 | | 6 | | 145.31 | | 6 | | 148.33 | | 6 | | 136.30 | | 6 | | 138.34 | | 6 | | 4 | | | 4 | | 8 | |  |  |  |  |
| O43707 | Alpha-actinin-4 | ACTN4 | 57.96 | 26 | | 104.8 | | 5.44 | | 64.44 | 6 | | 57.32 | | 6 | | 67.43 | | 6 | | 62.32 | | 6 | | 5.69 | | 6 | | 8.47 | | 6 | | 8.60 | | 6 | | 8.48 | | 6 | | 4 | | | 4 | | 8 | |  |  |  |  |
| P21333 | Filamin-A | FLNA | 42.54 | 73 | | 280.6 | | 6.06 | | 33.78 | 6 | | 41.57 | | 6 | | 60.48 | | 6 | | 60.00 | | 6 | | 45.94 | | 6 | | 45.05 | | 6 | | 67.02 | | 6 | | 50.83 | | 6 | | 4 | | | 4 | | 8 | |  |  |  |  |
| P06733 | Alpha-enolase | ENO1 | 54.38 | 25 | | 47.1 | | 7.39 | | 62.39 | 6 | | 54.75 | | 6 | | 58.88 | | 6 | | 58.23 | | 6 | | 12.02 | | 6 | | 15.36 | | 6 | | 14.83 | | 6 | | 19.79 | | 6 | | 4 | | | 4 | | 8 | |  |  |  |  |
| P12814 | Alpha-actinin-1 | ACTN1 | 47.87 | 22 | | 103.0 | | 5.41 | | 62.38 | 6 | | 54.89 | | 6 | | 55.28 | | 6 | | 54.37 | | 6 | | 5.38 | | 6 | | 7.73 | | 6 | | 9.79 | | 6 | | 9.49 | | 6 | | 4 | | | 4 | | 8 | |  |  |  |  |
| P68032 | Actin, alpha cardiac muscle 1 | ACTC1 | 65.25 | 1 | | 42.0 | | 5.39 | | 47.01 | 6 | | 48.84 | | 6 | | 52.05 | | 6 | | 55.43 | | 6 | | 89.00 | | 6 | | 92.51 | | 6 | | 89.12 | | 6 | | 91.41 | | 6 | | 4 | | | 4 | | 8 | |  |  |  |  |
| P00338 | L-lactate dehydrogenase A chain | LDHA | 50.60 | 19 | | 36.7 | | 8.27 | | 46.38 | 6 | | 45.88 | | 6 | | 51.73 | | 6 | | 54.33 | | 6 | | 4.61 | | 6 | | 4.66 | | 6 | | 3.65 | | 6 | | 5.11 | | 6 | | 4 | | | 4 | | 8 | |  |  |  |  |
| P14618 | Pyruvate kinase PKM | PKM | 55.18 | 28 | | 57.9 | | 7.84 | | 32.48 | 6 | | 38.88 | | 6 | | 51.41 | | 6 | | 53.28 | | 6 | | 59.39 | | 6 | | 75.20 | | 6 | | 75.98 | | 6 | | 80.59 | | 6 | | 4 | | | 4 | | 8 | |  |  |  |  |
| P62736 | Actin, aortic smooth muscle | ACTA2 | 65.25 | 1 | | 42.0 | | 5.39 | | 42.21 | 6 | | 43.02 | | 6 | | 46.14 | | 6 | | 50.40 | | 6 | | 81.20 | | 6 | | 84.10 | | 6 | | 77.20 | | 6 | | 79.85 | | 6 | | 4 | | | 4 | | 8 | |  |  |  |  |
| P26038 | Moesin | MSN | 52.51 | 30 | | 67.8 | | 6.40 | | 36.75 | 6 | | 38.78 | | 6 | | 36.43 | | 6 | | 37.08 | | 6 | | 19.24 | | 6 | | 21.89 | | 6 | | 20.46 | | 6 | | 24.19 | | 6 | | 4 | | | 4 | | 8 | |  |  |  |  |
| A0A2R8YEA7 | Actin, cytoplasmic 1 (Fragment) | ACTB | 63.69 | 1 | | 17.5 | | 4.73 | | 28.52 | 6 | | 29.18 | | 6 | | 33.27 | | 6 | | 34.92 | | 6 | | 40.98 | | 6 | | 45.01 | | 6 | | 48.46 | | 6 | | 47.76 | | 6 | | 4 | | | 4 | | 8 | |  |  |  |  |
| P11142 | Heat shock coate 71 kDa protein | HSPA8 | 43.96 | 24 | | 70.9 | | 5.52 | | 31.44 | 6 | | 31.41 | | 6 | | 31.83 | | 6 | | 30.62 | | 6 | | 36.77 | | 6 | | 39.02 | | 6 | | 26.55 | | 6 | | 28.92 | | 6 | | 4 | | | 4 | | 8 | |  |  |  |  |
| Q01995 | Transgelin | TAGLN | 68.66 | 14 | | 22.6 | | 8.84 | | 18.28 | 6 | | 19.69 | | 6 | | 30.66 | | 6 | | 29.30 | | 6 | | 3.69 | | 3 | | 4.72 | | 4 | | 5.47 | | 6 | | 7.18 | | 6 | | 4 | | | 4 | | 8 | |  |  |  |  |
| P02545 | Prelamin-A/C | LMNA | 47.74 | 29 | | 74.1 | | 7.02 | | 18.58 | 6 | | 22.83 | | 6 | | 30.21 | | 6 | | 31.77 | | 6 | | 8.10 | | 6 | | 2.67 | | 4 | | 8.37 | | 6 | | 4.17 | | 4 | | 4 | | | 4 | | 8 | |  |  |  |  |
| P18206 | Vinculin | VCL | 35.54 | 30 | | 123.7 | | 5.66 | | 27.95 | 6 | | 31.32 | | 6 | | 30.05 | | 6 | | 18.89 | | 6 | | 4.42 | | 5 | | 6.55 | | 6 | | 2.67 | | 5 | | 4.91 | | 6 | | 4 | | | 4 | | 8 | |  |  |  |  |
| P15311 | Ezrin | EZR | 45.56 | 18 | | 69.4 | | 6.27 | | 32.00 | 6 | | 28.20 | | 6 | | 28.73 | | 6 | | 21.29 | | 6 | | 17.70 | | 6 | | 18.31 | | 6 | | 15.70 | | 6 | | 16.84 | | 6 | | 4 | | | 4 | | 8 | |  |  |  |  |
| P50395 | Rab GDP dissociation inhibitor beta | GDI2 | 42.47 | 16 | | 50.6 | | 6.47 | | 23.03 | 6 | | 24.10 | | 6 | | 28.34 | | 6 | | 24.09 | | 6 | | 3.36 | | 3 | | 2.29 | | 5 | | 4.16 | | 5 | | 10.64 | | 6 | | 4 | | | 4 | | 8 | |  |  |  |  |
| P13639 | Elongation factor 2 | EEF2 | 41.14 | 29 | | 95.3 | | 6.83 | | 22.25 | 6 | | 21.86 | | 6 | | 28.15 | | 6 | | 23.45 | | 6 | | 27.05 | | 6 | | 24.57 | | 6 | | 41.76 | | 6 | | 35.99 | | 6 | | 4 | | | 4 | | 8 | |  |  |  |  |
| P02768 | Serum albumin | ALB | 3.61 | 3 | | 69.3 | | 6.28 | | 29.70 | 6 | | 20.58 | | 6 | | 27.36 | | 6 | | 28.11 | | 6 | | 4.42 | | 4 | | 4.38 | | 4 | | 3.77 | | 6 | | 2.69 | | 6 | | 4 | | | 4 | | 8 | |  |  |  |  |
| P04406 | Glyceraldehyde-3-phosphate dehydrogenase | GAPDH | 74.33 | 19 | | 36.0 | | 8.46 | | 21.16 | 6 | | 22.89 | | 6 | | 25.34 | | 6 | | 35.47 | | 6 | | 54.09 | | 6 | | 74.42 | | 6 | | 68.35 | | 6 | | 79.07 | | 6 | | 4 | | | 4 | | 8 | |  |  |  |  |
| P07737 | Profilin-1 | PFN1 | 56.43 | 8 | | 15.0 | | 8.27 | | 22.43 | 6 | | 21.71 | | 6 | | 24.54 | | 6 | | 24.46 | | 6 | | 4.49 | | 6 | | 7.54 | | 6 | | 6.37 | | 6 | | 9.10 | | 6 | | 4 | | | 4 | | 8 | |  |  |  |  |
| P60174 | Triosephosphate isomerase | TPI1 | 39.51 | 10 | | 30.8 | | 5.92 | | 24.00 | 6 | | 24.48 | | 6 | | 23.98 | | 6 | | 28.89 | | 6 | | 3.00 | | 5 | | 4.02 | | 6 | | 4.19 | | 6 | | 5.61 | | 6 | | 4 | | | 4 | | 8 | |  |  |  |  |
| P29401 | Transketolase | TKT | 47.51 | 20 | | 67.8 | | 7.66 | | 27.22 | 6 | | 29.31 | | 6 | | 22.04 | | 6 | | 22.44 | | 6 | | 0.14 | | 1 | | 0.32 | | 2 | | 0.29 | | 2 | | 0.30 | | 2 | | 4 | | | 4 | | 8 | |  |  |  |  |
| P04264 | Keratin, type II cytoskeletal 1 | KRT1 | 59.94 | 39 | | 66.0 | | 8.12 | | 14.39 | 6 | | 7.28 | | 4 | | 20.76 | | 6 | | 12.03 | | 4 | | 64.88 | | 6 | | 103.48 | | 6 | | 49.51 | | 6 | | 23.26 | | 6 | | 4 | | | 4 | | 8 | |  |  |  |  |
| P63104 | 14-3-3 protein zeta/delta | YWHAZ | 42.04 | 8 | | 27.7 | | 4.79 | | 21.06 | 6 | | 20.30 | | 6 | | 19.92 | | 6 | | 20.97 | | 6 | | 9.93 | | 6 | | 13.72 | | 6 | | 9.66 | | 6 | | 11.48 | | 6 | | 4 | | | 4 | | 8 | |  |  |  |  |
| Q562R1 | Beta-actin-like protein 2 | ACTBL2 | 27.66 | 3 | | 42.0 | | 5.59 | | 19.12 | 6 | | 20.01 | | 6 | | 19.91 | | 6 | | 24.32 | | 6 | | 29.55 | | 6 | | 38.19 | | 6 | | 32.91 | | 6 | | 32.56 | | 6 | | 4 | | | 4 | | 8 | |  |  |  |  |
| P00558 | Phosphoglycerate kinase 1 | PGK1 | 50.36 | 19 | | 44.6 | | 8.10 | | 15.20 | 6 | | 19.24 | | 6 | | 19.76 | | 6 | | 23.95 | | 6 | | 1.86 | | 4 | | 6.55 | | 6 | | 4.27 | | 5 | | 7.15 | | 6 | | 4 | | | 4 | | 8 | |  |  |  |  |
| A5A3E0 | POTE ankyrin domain family member F | POTEF | 8.65 | 1 | | 121.4 | | 6.20 | | 17.11 | 6 | | 21.32 | | 6 | | 18.69 | | 6 | | 23.74 | | 6 | | 32.65 | | 6 | | 35.16 | | 6 | | 32.55 | | 6 | | 33.31 | | 6 | | 4 | | | 4 | | 8 | |  |  |  |  |
| A0A2U3TZU2 | Glucose-6-phosphate isomerase | GPI | 29.82 | 13 | | 67.2 | | 8.97 | | 16.85 | 6 | | 21.00 | | 6 | | 18.46 | | 6 | | 19.96 | | 6 | | 0.65 | | 2 | | 0.82 | | 3 | | 1.34 | | 5 | | 2.39 | | 6 | | 4 | | | 4 | | 8 | |  |  |  |  |
| P62258 | 14-3-3 protein epsilon | YWHAE | 54.12 | 14 | | 29.2 | | 4.74 | | 21.08 | 6 | | 21.77 | | 6 | | 18.19 | | 6 | | 22.81 | | 6 | | 4.73 | | 6 | | 6.41 | | 6 | | 3.35 | | 6 | | 4.78 | | 6 | | 4 | | | 4 | | 8 | |  |  |  |  |
| A0A5F9ZHM4 | L-lactate dehydrogenase | LDHB | 38.42 | 14 | | 37.4 | | 6.25 | | 22.50 | 6 | | 21.55 | | 6 | | 17.70 | | 6 | | 19.96 | | 6 | | 1.06 | | 4 | | 1.16 | | 4 | | 1.01 | | 2 | | 0.33 | | 2 | | 4 | | | 4 | | 8 | |  |  |  |  |
| B7Z6Z4 | Myosin light polypeptide 6 | MYL6 | 25.63 | 6 | | 26.7 | | 5.08 | | 12.31 | 6 | | 17.35 | | 6 | | 16.89 | | 6 | | 17.65 | | 6 | | 8.86 | | 6 | | 8.93 | | 6 | | 11.71 | | 6 | | 10.56 | | 6 | | 4 | | | 4 | | 8 | |  |  |  |  |
| Q32Q12 | Nucleoside diphosphate kinase | NME1-NME2 | 53.77 | 4 | | 32.6 | | 8.48 | | 16.89 | 6 | | 18.37 | | 6 | | 16.33 | | 6 | | 18.98 | | 6 | | 0.93 | | 4 | | 1.65 | | 5 | | 1.85 | | 6 | | 1.60 | | 6 | | 4 | | | 4 | | 8 | |  |  |  |  |
| P62979 | Ubiquitin-40S ribosomal protein S27a | RPS27A | 38.46 | 7 | | 18.0 | | 9.64 | | 16.02 | 6 | | 14.90 | | 6 | | 15.17 | | 6 | | 17.39 | | 6 | | 21.20 | | 6 | | 22.04 | | 6 | | 18.98 | | 6 | | 19.39 | | 6 | | 4 | | | 4 | | 8 | |  |  |  |  |
| P18669 | Phosphoglycerate mutase 1 | PGAM1 | 58.66 | 7 | | 28.8 | | 7.18 | | 15.11 | 6 | | 11.92 | | 6 | | 15.01 | | 6 | | 12.43 | | 6 | | 1.13 | | 3 | | 1.43 | | 2 | | 0.44 | | 2 | | 1.71 | | 5 | | 4 | | | 4 | | 8 | |  |  |  |  |
| P35527 | Keratin, type I cytoskeletal 9 | KRT9 | 85.39 | 40 | | 62.0 | | 5.24 | | 5.92 | 6 | | 1.93 | | 2 | | 14.85 | | 6 | | 6.16 | | 4 | | 52.47 | | 3 | | 92.12 | | 6 | | 42.80 | | 6 | | 16.31 | | 6 | | 4 | | | 4 | | 8 | |  |  |  |  |
| P01023 | Alpha-2-macroglobulin | A2M | 7.53 | 4 | | 163.2 | | 6.46 | | 15.58 | 6 | | 8.13 | | 6 | | 14.55 | | 6 | | 14.94 | | 6 | | 4.42 | | 5 | | 4.24 | | 4 | | 9.02 | | 6 | | 11.21 | | 6 | | 4 | | | 4 | | 8 | |  |  |  |  |
| J3KPS3 | Fructose-bisphosphate aldolase | ALDOA | 41.85 | 14 | | 39.8 | | 8.09 | | 12.38 | 6 | | 12.52 | | 6 | | 14.32 | | 6 | | 14.56 | | 6 | | 5.37 | | 6 | | 7.76 | | 6 | | 7.12 | | 6 | | 7.50 | | 6 | | 4 | | | 4 | | 8 | |  |  |  |  |
| A0A2R8Y5S7 | Radixin | RDX | 18.14 | 3 | | 69.3 | | 6.27 | | 13.74 | 6 | | 12.82 | | 6 | | 12.01 | | 6 | | 11.60 | | 6 | | 5.97 | | 5 | | 7.03 | | 6 | | 7.53 | | 6 | | 8.49 | | 6 | | 4 | | | 4 | | 8 | |  |  |  |  |
| P09382 | Galectin-1 | LGALS1 | 31.11 | 4 | | 14.7 | | 5.50 | | 6.00 | 6 | | 7.40 | | 6 | | 11.36 | | 6 | | 11.86 | | 6 | | 0.85 | | 3 | | 1.19 | | 3 | | 3.62 | | 6 | | 3.22 | | 6 | | 4 | | | 4 | | 8 | |  |  |  |  |
| P61981 | 14-3-3 protein gamma | YWHAG | 38.87 | 6 | | 28.3 | | 4.89 | | 13.74 | 6 | | 12.85 | | 6 | | 11.33 | | 6 | | 11.95 | | 6 | | 5.95 | | 6 | | 8.58 | | 6 | | 4.17 | | 6 | | 5.62 | | 6 | | 4 | | | 4 | | 8 | |  |  |  |  |
| P31946 | 14-3-3 protein beta/alpha | YWHAB | 39.02 | 4 | | 28.1 | | 4.83 | | 12.69 | 6 | | 11.43 | | 6 | | 10.66 | | 6 | | 9.24 | | 6 | | 4.39 | | 5 | | 6.26 | | 6 | | 2.03 | | 5 | | 3.56 | | 6 | | 4 | | | 4 | | 8 | |  |  |  |  |
| Q06830 | Peroxiredoxin-1 | PRDX1 | 48.74 | 10 | | 22.1 | | 8.13 | | 12.93 | 6 | | 13.05 | | 6 | | 10.57 | | 6 | | 9.32 | | 6 | | 12.18 | | 6 | | 10.07 | | 6 | | 13.22 | | 6 | | 9.19 | | 6 | | 4 | | | 4 | | 8 | |  |  |  |  |
| Q08380 | Galectin-3-binding protein | LGALS3BP | 38.12 | 19 | | 65.3 | | 5.27 | | 17.41 | 6 | | 12.41 | | 6 | | 10.19 | | 6 | | 6.61 | | 6 | | 23.08 | | 5 | | 30.15 | | 6 | | 46.01 | | 6 | | 50.01 | | 6 | | 4 | | | 4 | | 8 | |  |  |  |  |
| O75369 | Filamin-B | FLNB | 11.38 | 15 | | 278.0 | | 5.73 | | 9.37 | 6 | | 5.89 | | 6 | | 10.10 | | 6 | | 6.77 | | 6 | | 3.86 | | 4 | | 2.11 | | 4 | | 5.99 | | 6 | | 4.09 | | 6 | | 4 | | | 4 | | 8 | |  |  |  |  |
| Q6FI13 | Histone H2A type 2-A | H2AC18 | 63.08 | 5 | | 14.1 | | 10.90 | | 9.24 | 6 | | 7.75 | | 6 | | 10.02 | | 6 | | 7.80 | | 6 | | 31.92 | | 6 | | 27.67 | | 6 | | 39.07 | | 6 | | 35.13 | | 6 | | 4 | | | 4 | | 8 | |  |  |  |  |
| P04908 | Histone H2A type 1-B/E | H2AC4 | 63.08 | 2 | | 14.1 | | 11.05 | | 9.24 | 6 | | 7.75 | | 6 | | 10.02 | | 6 | | 7.80 | | 6 | | 25.06 | | 6 | | 22.38 | | 6 | | 31.09 | | 6 | | 27.85 | | 6 | | 4 | | | 4 | | 8 | |  |  |  |  |
| Q5TCU3 | Tropomyosin beta chain | TPM2 | 47.54 | 2 | | 32.8 | | 4.68 | | 6.30 | 6 | | 13.72 | | 6 | | 9.97 | | 6 | | 14.84 | | 6 | | 0.16 | | 1 | | 0.62 | | 3 | | 1.16 | | 3 | | 0.46 | | 2 | | 4 | | | 4 | | 8 | |  |  |  |  |
| A7XZE4 | Beta tropomyosin isoform | TPM2 | 42.61 | 2 | | 33.0 | | 4.69 | | 6.17 | 6 | | 11.49 | | 6 | | 9.75 | | 6 | | 14.25 | | 6 | | 0.16 | | 1 | | 0.62 | | 3 | | 1.16 | | 3 | | 0.46 | | 2 | | 4 | | | 4 | | 8 | |  |  |  |  |
| P69905 | Hemoglobin subunit alpha | HBA1 | 28.17 | 3 | | 15.2 | | 8.68 | | 5.30 | 6 | | 4.20 | | 6 | | 9.61 | | 6 | | 8.99 | | 6 | | 4.25 | | 5 | | 6.08 | | 6 | | 9.47 | | 6 | | 9.67 | | 6 | | 4 | | | 4 | | 8 | |  |  |  |  |
| Q14315 | Filamin-C | FLNC | 9.43 | 13 | | 290.8 | | 5.97 | | 7.79 | 6 | | 8.07 | | 6 | | 9.43 | | 6 | | 7.29 | | 6 | | 4.18 | | 6 | | 3.06 | | 5 | | 6.06 | | 6 | | 4.57 | | 6 | | 4 | | | 4 | | 8 | |  |  |  |  |
| P62805 | Histone H4 | H4C1 | 59.22 | 14 | | 11.4 | | 11.36 | | 13.90 | 6 | | 6.15 | | 6 | | 9.42 | | 6 | | 8.09 | | 6 | | 43.06 | | 5 | | 27.40 | | 6 | | 50.39 | | 6 | | 46.36 | | 6 | | 4 | | | 4 | | 8 | |  |  |  |  |
| P62937 | Peptidyl-prolyl cis-trans isomerase A | PPIA | 28.48 | 7 | | 18.0 | | 7.81 | | 7.63 | 6 | | 8.98 | | 6 | | 9.13 | | 6 | | 8.56 | | 6 | | 2.68 | | 5 | | 4.47 | | 6 | | 4.28 | | 6 | | 5.38 | | 6 | | 4 | | | 4 | | 8 | |  |  |  |  |
| P27348 | 14-3-3 protein theta | YWHAQ | 28.16 | 3 | | 27.7 | | 4.78 | | 10.04 | 6 | | 8.56 | | 6 | | 8.20 | | 6 | | 7.98 | | 6 | | 5.87 | | 6 | | 6.91 | | 6 | | 4.20 | | 6 | | 4.87 | | 6 | | 4 | | | 4 | | 8 | |  |  |  |  |
| P09211 | Glutathione S-transferase P | GSTP1 | 47.14 | 7 | | 23.3 | | 5.64 | | 9.34 | 6 | | 12.41 | | 6 | | 7.56 | | 6 | | 10.90 | | 6 | | 0.62 | | 3 | | 2.77 | | 6 | | 1.78 | | 5 | | 2.03 | | 6 | | 4 | | | 4 | | 8 | |  |  |  |  |
| P15531 | Nucleoside diphosphate kinase A | NME1 | 52.63 | 1 | | 17.1 | | 6.19 | | 8.89 | 6 | | 9.11 | | 6 | | 7.41 | | 6 | | 8.59 | | 6 | | 0.93 | | 4 | | 1.65 | | 5 | | 1.85 | | 6 | | 1.60 | | 6 | | 4 | | | 4 | | 8 | |  |  |  |  |
| P09104 | Gamma-enolase | ENO2 | 16.36 | 3 | | 47.2 | | 5.03 | | 6.96 | 6 | | 6.89 | | 6 | | 7.22 | | 6 | | 6.62 | | 6 | | 0.65 | | 2 | | 1.79 | | 6 | | 1.23 | | 5 | | 1.41 | | 5 | | 4 | | | 4 | | 8 | |  |  |  |  |
| P11021 | Endoplasmic reticulum chaperone BiP | HSPA5 | 31.80 | 15 | | 72.3 | | 5.16 | | 9.53 | 6 | | 16.26 | | 6 | | 6.95 | | 6 | | 12.36 | | 6 | | 2.99 | | 5 | | 4.32 | | 6 | | 2.78 | | 6 | | 2.90 | | 6 | | 4 | | | 4 | | 8 | |  |  |  |  |
| P07437 | Tubulin beta chain | TUBB | 70.50 | 5 | | 49.6 | | 4.89 | | 8.50 | 6 | | 13.03 | | 6 | | 6.78 | | 6 | | 14.25 | | 6 | | 64.03 | | 6 | | 54.96 | | 6 | | 56.94 | | 6 | | 51.59 | | 6 | | 4 | | | 4 | | 8 | |  |  |  |  |
| P13929 | Beta-enolase | ENO3 | 11.52 | 1 | | 47.0 | | 7.71 | | 6.71 | 6 | | 6.78 | | 6 | | 6.75 | | 6 | | 5.82 | | 6 | | 0.65 | | 2 | | 1.79 | | 6 | | 1.23 | | 5 | | 1.56 | | 5 | | 4 | | | 4 | | 8 | |  |  |  |  |
| P20742 | Preancy zone protein | PZP | 4.93 | 1 | | 163.8 | | 6.38 | | 6.70 | 6 | | 3.06 | | 6 | | 6.34 | | 6 | | 6.66 | | 6 | | 0.32 | | 1 | | 0.50 | | 2 | | 1.93 | | 4 | | 3.22 | | 2 | | 4 | | | 4 | | 8 | |  |  |  |  |
| P26022 | Pentraxin-related protein PTX3 | PTX3 | 25.20 | 7 | | 41.9 | | 5.01 | | 8.95 | 6 | | 6.68 | | 6 | | 6.34 | | 6 | | 4.93 | | 6 | | 4.61 | | 5 | | 3.29 | | 4 | | 1.76 | | 4 | | 2.31 | | 3 | | 4 | | | 4 | | 8 | |  |  |  |  |
| P61158 | Actin-related protein 3 | ACTR3 | 14.83 | 5 | | 47.3 | | 5.88 | | 5.18 | 6 | | 5.58 | | 6 | | 5.77 | | 6 | | 4.10 | | 6 | | 2.26 | | 3 | | 2.22 | | 2 | | 2.30 | | 6 | | 3.30 | | 6 | | 4 | | | 4 | | 8 | |  |  |  |  |
| F5H5D3 | Tubulin alpha chain | TUBA1C | 52.99 | 4 | | 57.7 | | 5.07 | | 7.31 | 6 | | 8.04 | | 6 | | 5.76 | | 6 | | 7.48 | | 6 | | 32.01 | | 6 | | 25.78 | | 6 | | 22.96 | | 6 | | 21.00 | | 6 | | 4 | | | 4 | | 8 | |  |  |  |  |
| Q71U36 | Tubulin alpha-1A chain | TUBA1A | 56.76 | 2 | | 50.1 | | 5.06 | | 7.31 | 6 | | 8.04 | | 6 | | 5.76 | | 6 | | 7.48 | | 6 | | 31.87 | | 6 | | 25.78 | | 6 | | 22.96 | | 6 | | 21.00 | | 6 | | 4 | | | 4 | | 8 | |  |  |  |  |
| Q04917 | 14-3-3 protein eta | YWHAH | 31.30 | 5 | | 28.2 | | 4.84 | | 9.17 | 6 | | 6.80 | | 6 | | 5.71 | | 6 | | 5.35 | | 6 | | 2.83 | | 4 | | 3.98 | | 6 | | 2.18 | | 5 | | 2.65 | | 6 | | 4 | | | 4 | | 8 | |  |  |  |  |
| Q9BVA1 | Tubulin beta-2B chain | TUBB2B | 55.06 | 1 | | 49.9 | | 4.89 | | 6.76 | 6 | | 10.28 | | 6 | | 5.65 | | 6 | | 11.86 | | 6 | | 48.58 | | 6 | | 39.14 | | 6 | | 42.16 | | 6 | | 36.03 | | 6 | | 4 | | | 4 | | 8 | |  |  |  |  |
| P06703 | Protein S100-A6 | S100A6 | 47.78 | 3 | | 10.2 | | 5.48 | | 4.15 | 6 | | 4.73 | | 6 | | 5.29 | | 6 | | 4.84 | | 6 | | 4.46 | | 6 | | 5.18 | | 6 | | 5.71 | | 6 | | 5.35 | | 6 | | 4 | | | 4 | | 8 | |  |  |  |  |
| P68371 | Tubulin beta-4B chain | TUBB4B | 70.34 | 1 | | 49.8 | | 4.89 | | 7.19 | 6 | | 9.23 | | 6 | | 5.09 | | 6 | | 12.18 | | 6 | | 57.36 | | 6 | | 49.58 | | 6 | | 53.78 | | 6 | | 45.26 | | 6 | | 4 | | | 4 | | 8 | |  |  |  |  |
| P31949 | Protein S100-A11 | S100A11 | 23.81 | 2 | | 11.7 | | 7.12 | | 5.61 | 6 | | 5.34 | | 6 | | 4.54 | | 6 | | 6.43 | | 6 | | 2.28 | | 5 | | 1.75 | | 6 | | 3.41 | | 6 | | 4.02 | | 6 | | 4 | | | 4 | | 8 | |  |  |  |  |
| Q13509 | Tubulin beta-3 chain | TUBB3 | 53.33 | 4 | | 50.4 | | 4.93 | | 6.45 | 6 | | 8.98 | | 6 | | 4.36 | | 6 | | 8.60 | | 6 | | 52.16 | | 6 | | 40.86 | | 6 | | 45.30 | | 6 | | 40.72 | | 6 | | 4 | | | 4 | | 8 | |  |  |  |  |
| A0A2R8Y7X9 | GLOBIN domain-containing protein |  | 5.95 | 1 | | 18.4 | | 6.96 | | 3.71 | 6 | | 3.18 | | 6 | | 4.23 | | 6 | | 4.25 | | 6 | | 1.43 | | 3 | | 2.63 | | 6 | | 3.41 | | 6 | | 3.89 | | 6 | | 4 | | | 4 | | 8 | |  |  |  |  |
| P05787 | Keratin, type II cytoskeletal 8 | KRT8 | 34.16 | 11 | | 53.7 | | 5.59 | | 7.90 | 6 | | 7.85 | | 6 | | 4.19 | | 6 | | 3.59 | | 6 | | 9.16 | | 6 | | 9.20 | | 6 | | 3.31 | | 6 | | 2.75 | | 6 | | 4 | | | 4 | | 8 | |  |  |  |  |
| P08727 | Keratin, type I cytoskeletal 19 | KRT19 | 40.25 | 8 | | 44.1 | | 5.14 | | 6.66 | 6 | | 4.58 | | 6 | | 4.00 | | 6 | | 1.58 | | 4 | | 13.02 | | 6 | | 10.38 | | 6 | | 4.11 | | 4 | | 3.10 | | 6 | | 4 | | | 4 | | 8 | |  |  |  |  |
| P25787 | Proteasome subunit alpha type-2 | PSMA2 | 32.48 | 5 | | 25.9 | | 7.43 | | 3.74 | 6 | | 2.99 | | 5 | | 3.53 | | 6 | | 3.92 | | 6 | | 2.58 | | 5 | | 2.27 | | 4 | | 4.34 | | 6 | | 5.06 | | 6 | | 4 | | | 4 | | 8 | |  |  |  |  |
| E7EMB3 | Calmodulin-2 | CALM2 | 39.80 | 11 | | 21.7 | | 4.56 | | 7.36 | 6 | | 10.62 | | 6 | | 3.42 | | 6 | | 7.31 | | 6 | | 6.76 | | 4 | | 8.50 | | 6 | | 5.47 | | 6 | | 6.47 | | 6 | | 4 | | | 4 | | 8 | |  |  |  |  |
| P62328 | Thymosin beta-4 | TMSB4X | 56.82 | 8 | | 5.1 | | 5.06 | | 3.05 | 6 | | 2.17 | | 6 | | 3.37 | | 6 | | 2.69 | | 6 | | 7.61 | | 3 | | 4.26 | | 4 | | 2.28 | | 6 | | 3.05 | | 6 | | 4 | | | 4 | | 8 | |  |  |  |  |
| P23284 | Peptidyl-prolyl cis-trans isomerase B | PPIB | 44.91 | 11 | | 23.7 | | 9.41 | | 8.43 | 6 | | 9.47 | | 6 | | 3.20 | | 5 | | 6.12 | | 6 | | 2.58 | | 2 | | 1.64 | | 2 | | 1.35 | | 2 | | 1.52 | | 2 | | 4 | | | 4 | | 8 | |  |  |  |  |
| A0A0G2JIW1 | Heat shock 70 kDa protein 1B | HSPA1B | 17.29 | 6 | | 70.1 | | 5.66 | | 5.28 | 6 | | 4.69 | | 6 | | 3.15 | | 6 | | 4.03 | | 6 | | 5.82 | | 6 | | 8.90 | | 6 | | 5.53 | | 6 | | 6.71 | | 6 | | 4 | | | 4 | | 8 | |  |  |  |  |
| U3KQK0 | Histone H2B | H2BC15 | 41.57 | 2 | | 18.8 | | 10.54 | | 5.51 | 6 | | 4.99 | | 6 | | 2.93 | | 6 | | 4.16 | | 6 | | 29.33 | | 6 | | 23.56 | | 6 | | 32.36 | | 6 | | 31.93 | | 6 | | 4 | | | 4 | | 8 | |  |  |  |  |
| P04350 | Tubulin beta-4A chain | TUBB4A | 63.06 | 2 | | 49.6 | | 4.88 | | 4.92 | 6 | | 5.40 | | 6 | | 2.86 | | 6 | | 8.59 | | 6 | | 46.47 | | 6 | | 38.55 | | 6 | | 41.62 | | 6 | | 33.26 | | 6 | | 4 | | | 4 | | 8 | |  |  |  |  |
| P06899 | Histone H2B type 1-J | H2BC11 | 54.76 | 2 | | 13.9 | | 10.32 | | 4.60 | 6 | | 4.51 | | 6 | | 2.71 | | 6 | | 3.79 | | 6 | | 23.63 | | 6 | | 17.61 | | 6 | | 26.51 | | 6 | | 23.89 | | 6 | | 4 | | | 4 | | 8 | |  |  |  |  |
| P08729 | Keratin, type II cytoskeletal 7 | KRT7 | 15.99 | 2 | | 51.4 | | 5.48 | | 2.91 | 6 | | 1.73 | | 6 | | 2.68 | | 6 | | 2.45 | | 6 | | 2.66 | | 3 | | 4.90 | | 5 | | 1.94 | | 6 | | 1.94 | | 6 | | 4 | | | 4 | | 8 | |  |  |  |  |
| Q9BUF5 | Tubulin beta-6 chain | TUBB6 | 63.23 | 7 | | 49.8 | | 4.88 | | 3.65 | 6 | | 4.18 | | 6 | | 2.49 | | 6 | | 5.22 | | 6 | | 32.43 | | 6 | | 21.32 | | 6 | | 25.67 | | 6 | | 20.03 | | 6 | | 4 | | | 4 | | 8 | |  |  |  |  |
| Q9H299 | SH3 domain-binding glutamic acid-rich-like protein 3 | SH3BGRL3 | 31.18 | 3 | | 10.4 | | 4.93 | | 2.41 | 6 | | 2.13 | | 6 | | 2.43 | | 6 | | 2.12 | | 6 | | 1.26 | | 3 | | 1.66 | | 4 | | 0.92 | | 4 | | 1.93 | | 6 | | 4 | | | 4 | | 8 | |  |  |  |  |
| P06748 | Nucleophosmin | NPM1 | 45.58 | 10 | | 32.6 | | 4.78 | | 2.25 | 6 | | 2.57 | | 6 | | 2.40 | | 6 | | 1.90 | | 5 | | 13.82 | | 6 | | 11.80 | | 6 | | 13.02 | | 6 | | 11.33 | | 6 | | 4 | | | 4 | | 8 | |  |  |  |  |
| A6NNZ2 | Tubulin beta 8B | TUBB8B | 25.45 | 1 | | 49.5 | | 4.86 | | 3.02 | 6 | | 3.04 | | 6 | | 2.11 | | 6 | | 3.73 | | 6 | | 19.81 | | 6 | | 16.90 | | 6 | | 16.45 | | 6 | | 13.64 | | 6 | | 4 | | | 4 | | 8 | |  |  |  |  |
| A0A075B6Z2 | T cell receptor alpha joining 56 (Fragment) | TRAJ56 | 38.10 | 1 | | 2.2 | | 10.29 | | 3.30 | 6 | | 2.86 | | 6 | | 2.08 | | 5 | | 2.28 | | 6 | | 4.41 | | 6 | | 4.09 | | 6 | | 4.47 | | 6 | | 4.79 | | 6 | | 4 | | | 4 | | 8 | |  |  |  |  |
| P13611 | Versican core protein | VCAN | 4.86 | 14 | | 372.6 | | 4.51 | | 10.21 | 6 | | 10.21 | | 6 | | 2.05 | | 4 | | 1.75 | | 3 | | 17.01 | | 5 | | 22.39 | | 6 | | 4.99 | | 4 | | 4.48 | | 4 | | 4 | | | 4 | | 8 | |  |  |  |  |
| P05783 | Keratin, type I cytoskeletal 18 | KRT18 | 10.00 | 2 | | 48.0 | | 5.45 | | 3.19 | 6 | | 1.89 | | 4 | | 1.48 | | 6 | | 0.16 | | 1 | | 5.46 | | 6 | | 5.04 | | 6 | | 1.03 | | 4 | | 0.61 | | 3 | | 4 | | | 4 | | 8 | |  |  |  |  |
| Q13162 | Peroxiredoxin-4 | PRDX4 | 8.12 | 1 | | 30.5 | | 6.29 | | 1.97 | 6 | | 1.94 | | 6 | | 1.19 | | 4 | | 1.97 | | 6 | | 2.66 | | 5 | | 2.19 | | 5 | | 2.96 | | 6 | | 2.09 | | 6 | | 4 | | | 4 | | 8 | |  |  |  |  |
| O43854 | EGF-like repeat and discoidin I-like domain-containing protein 3 | EDIL3 | 43.96 | 23 | | 53.7 | | 7.28 | | 1.69 | 6 | | 1.94 | | 5 | | 0.31 | | 2 | | 1.16 | | 4 | | 47.90 | | 6 | | 35.13 | | 6 | | 64.36 | | 6 | | 56.86 | | 6 | | 4 | | | 4 | | 8 | |  |  |  |  |
| P37802 | Transgelin-2 | TAGLN2 | 60.30 | 11 | | 22.4 | | 8.25 | | 12.74 | 5 | | 14.22 | | 6 | | 20.30 | | 6 | | 20.72 | | 6 | | 2.42 | | 5 | | 3.33 | | 6 | | 3.87 | | 6 | | 4.75 | | 6 | | 4 | | | 4 | | 8 | |  |  |  |  |
| P07900 | Heat shock protein HSP 90-alpha | HSP90AA1 | 35.25 | 10 | | 84.6 | | 5.02 | | 15.77 | 5 | | 15.15 | | 6 | | 14.24 | | 6 | | 16.70 | | 6 | | 24.18 | | 6 | | 21.87 | | 6 | | 26.58 | | 6 | | 17.02 | | 6 | | 4 | | | 4 | | 8 | |  |  |  |  |
| P68104 | Elongation factor 1-alpha 1 | EEF1A1 | 36.36 | 14 | | 50.1 | | 9.01 | | 10.71 | 5 | | 13.76 | | 6 | | 14.22 | | 6 | | 14.20 | | 6 | | 33.37 | | 6 | | 26.09 | | 6 | | 34.64 | | 6 | | 24.96 | | 6 | | 4 | | | 4 | | 8 | |  |  |  |  |
| P08238 | Heat shock protein HSP 90-beta | HSP90AB1 | 46.82 | 21 | | 83.2 | | 5.03 | | 13.45 | 5 | | 15.22 | | 6 | | 13.50 | | 6 | | 15.28 | | 6 | | 43.65 | | 6 | | 32.64 | | 6 | | 40.08 | | 6 | | 27.73 | | 6 | | 4 | | | 4 | | 8 | |  |  |  |  |
| E9PK25 | Cofilin-1 | CFL1 | 35.78 | 3 | | 22.7 | | 8.34 | | 7.73 | 5 | | 10.92 | | 6 | | 12.28 | | 6 | | 15.20 | | 6 | | 4.27 | | 6 | | 5.91 | | 6 | | 6.16 | | 6 | | 5.01 | | 6 | | 4 | | | 4 | | 8 | |  |  |  |  |
| P13645 | Keratin, type I cytoskeletal 10 | KRT10 | 47.77 | 27 | | 58.8 | | 5.21 | | 5.93 | 5 | | 2.25 | | 2 | | 12.06 | | 6 | | 2.42 | | 4 | | 55.20 | | 5 | | 53.58 | | 6 | | 32.82 | | 6 | | 25.53 | | 6 | | 4 | | | 4 | | 8 | |  |  |  |  |
| D6R956 | Ubiquitin carboxyl-terminal hydrolase | UCHL1 | 29.46 | 6 | | 26.8 | | 5.81 | | 6.64 | 5 | | 8.01 | | 6 | | 8.59 | | 6 | | 10.01 | | 6 | | 0.79 | | 3 | | 0.36 | | 2 | | 0.59 | | 3 | | 1.40 | | 4 | | 4 | | | 4 | | 8 | |  |  |  |  |
| P02533 | Keratin, type I cytoskeletal 14 | KRT14 | 55.08 | 9 | | 51.5 | | 5.16 | | 3.45 | 5 | | 2.42 | | 5 | | 7.21 | | 6 | | 2.71 | | 4 | | 13.80 | | 6 | | 23.83 | | 6 | | 9.50 | | 4 | | 4.32 | | 6 | | 4 | | | 4 | | 8 | |  |  |  |  |
| P08779 | Keratin, type I cytoskeletal 16 | KRT16 | 49.47 | 11 | | 51.2 | | 5.05 | | 3.45 | 5 | | 2.42 | | 5 | | 6.65 | | 6 | | 2.71 | | 4 | | 12.96 | | 6 | | 13.78 | | 6 | | 11.95 | | 4 | | 4.32 | | 6 | | 4 | | | 4 | | 8 | |  |  |  |  |
| P0C0S5 | Histone H2A.Z | H2AZ1 | 53.91 | 4 | | 13.5 | | 10.58 | | 3.22 | 5 | | 2.70 | | 6 | | 5.25 | | 6 | | 3.75 | | 6 | | 9.27 | | 6 | | 7.17 | | 6 | | 11.90 | | 6 | | 9.17 | | 6 | | 4 | | | 4 | | 8 | |  |  |  |  |
| Q04695 | Keratin, type I cytoskeletal 17 | KRT17 | 21.30 | 1 | | 48.1 | | 5.02 | | 2.98 | 5 | | 1.46 | | 5 | | 4.76 | | 6 | | 1.58 | | 4 | | 8.69 | | 6 | | 10.30 | | 4 | | 6.28 | | 4 | | 3.71 | | 6 | | 4 | | | 4 | | 8 | |  |  |  |  |
| Q7Z3Y7 | Keratin, type I cytoskeletal 28 | KRT28 | 9.70 | 1 | | 50.5 | | 5.47 | | 1.60 | 5 | | 0.97 | | 2 | | 4.35 | | 6 | | 1.13 | | 4 | | 9.27 | | 5 | | 8.98 | | 6 | | 3.84 | | 4 | | 2.59 | | 3 | | 4 | | | 4 | | 8 | |  |  |  |  |
| P32119 | Peroxiredoxin-2 | PRDX2 | 32.32 | 6 | | 21.9 | | 5.97 | | 4.14 | 5 | | 5.17 | | 6 | | 4.34 | | 6 | | 1.92 | | 6 | | 3.58 | | 6 | | 2.06 | | 6 | | 1.85 | | 6 | | 1.58 | | 5 | | 4 | | | 4 | | 8 | |  |  |  |  |
| P29966 | Myristoylated alanine-rich C-kinase substrate | MARCKS | 50.90 | 7 | | 31.5 | | 4.45 | | 1.95 | 5 | | 2.72 | | 6 | | 3.12 | | 6 | | 3.27 | | 6 | | 4.16 | | 6 | | 5.00 | | 6 | | 6.98 | | 6 | | 8.05 | | 6 | | 4 | | | 4 | | 8 | |  |  |  |  |
| P16070 | CD44 antigen | CD44 | 6.20 | 5 | | 81.5 | | 5.33 | | 3.16 | 5 | | 2.54 | | 6 | | 1.86 | | 6 | | 2.12 | | 6 | | 8.19 | | 6 | | 9.32 | | 6 | | 10.61 | | 6 | | 12.34 | | 6 | | 4 | | | 4 | | 8 | |  |  |  |  |
| Q7Z794 | Keratin, type II cytoskeletal 1b | KRT77 | 6.23 | 1 | | 61.9 | | 5.99 | | 2.32 | 5 | | 1.35 | | 4 | | 1.72 | | 4 | | 2.12 | | 6 | | 2.85 | | 3 | | 4.11 | | 6 | | 2.51 | | 4 | | 1.40 | | 5 | | 4 | | | 4 | | 8 | |  |  |  |  |
| P35579 | Myosin-9 | MYH9 | 40.36 | 63 | | 226.4 | | 5.60 | | 12.94 | 4 | | 12.89 | | 6 | | 26.74 | | 6 | | 29.15 | | 6 | | 25.75 | | 6 | | 35.33 | | 6 | | 34.64 | | 6 | | 39.73 | | 6 | | 4 | | | 4 | | 8 | |  |  |  |  |
| P02452 | Collagen alpha-1(I) chain | COL1A1 | 17.62 | 20 | | 138.9 | | 5.80 | | 5.12 | 4 | | 4.09 | | 4 | | 19.26 | | 6 | | 14.98 | | 6 | | 6.67 | | 3 | | 9.45 | | 4 | | 14.83 | | 6 | | 10.88 | | 4 | | 4 | | | 4 | | 8 | |  |  |  |  |
| Q9Y490 | Talin-1 | TLN1 | 11.73 | 15 | | 269.6 | | 6.07 | | 4.45 | 4 | | 4.66 | | 6 | | 10.53 | | 6 | | 3.84 | | 4 | | 2.30 | | 4 | | 2.65 | | 4 | | 4.77 | | 6 | | 3.72 | | 6 | | 4 | | | 4 | | 8 | |  |  |  |  |
| P04259 | Keratin, type II cytoskeletal 6B | KRT6B | 34.93 | 3 | | 60.0 | | 8.00 | | 4.84 | 4 | | 2.27 | | 4 | | 7.67 | | 6 | | 3.41 | | 4 | | 15.49 | | 6 | | 17.75 | | 6 | | 13.06 | | 6 | | 6.13 | | 6 | | 4 | | | 4 | | 8 | |  |  |  |  |
| J3QRS3 | Myosin regulatory light chain 12A | MYL12A | 42.94 | 3 | | 20.4 | | 4.75 | | 1.77 | 4 | | 7.36 | | 6 | | 6.92 | | 6 | | 10.87 | | 6 | | 2.49 | | 5 | | 2.53 | | 6 | | 4.13 | | 6 | | 2.93 | | 6 | | 4 | | | 4 | | 8 | |  |  |  |  |
| P09651 | Heterogeneous nuclear ribonucleoprotein A1 | HNRNPA1 | 39.78 | 13 | | 38.7 | | 9.13 | | 6.36 | 4 | | 4.10 | | 5 | | 6.91 | | 6 | | 7.91 | | 6 | | 6.06 | | 3 | | 3.38 | | 5 | | 5.45 | | 6 | | 1.84 | | 5 | | 4 | | | 4 | | 8 | |  |  |  |  |
| Q15149 | Plectin | PLEC | 8.73 | 30 | | 531.5 | | 5.96 | | 4.12 | 4 | | 4.13 | | 5 | | 6.29 | | 6 | | 2.70 | | 4 | | 3.85 | | 4 | | 3.30 | | 6 | | 3.58 | | 5 | | 2.31 | | 5 | | 4 | | | 4 | | 8 | |  |  |  |  |
| P02538 | Keratin, type II cytoskeletal 6A | KRT6A | 36.88 | 4 | | 60.0 | | 8.00 | | 3.54 | 4 | | 1.63 | | 4 | | 6.10 | | 6 | | 2.12 | | 3 | | 11.90 | | 6 | | 15.14 | | 6 | | 11.25 | | 6 | | 3.74 | | 6 | | 4 | | | 4 | | 8 | |  |  |  |  |
| P35908 | Keratin, type II cytoskeletal 2 epidermal | KRT2 | 61.50 | 22 | | 65.4 | | 8.00 | | 4.30 | 4 | | 1.28 | | 2 | | 5.98 | | 6 | | 2.43 | | 4 | | 33.12 | | 3 | | 34.34 | | 6 | | 14.11 | | 5 | | 10.65 | | 6 | | 4 | | | 4 | | 8 | |  |  |  |  |
| Q9Y281 | Cofilin-2 | CFL2 | 30.72 | 1 | | 18.7 | | 7.88 | | 2.98 | 4 | | 4.48 | | 6 | | 5.21 | | 6 | | 5.45 | | 6 | | 1.91 | | 5 | | 2.97 | | 6 | | 3.10 | | 6 | | 2.44 | | 6 | | 4 | | | 4 | | 8 | |  |  |  |  |
| P13647 | Keratin, type II cytoskeletal 5 | KRT5 | 36.78 | 16 | | 62.3 | | 7.74 | | 2.90 | 4 | | 1.35 | | 4 | | 4.56 | | 6 | | 1.79 | | 3 | | 11.45 | | 6 | | 21.22 | | 6 | | 6.91 | | 5 | | 3.10 | | 6 | | 4 | | | 4 | | 8 | |  |  |  |  |
| Q16658 | Fascin | FSCN1 | 23.94 | 9 | | 54.5 | | 7.24 | | 3.60 | 4 | | 5.59 | | 6 | | 4.48 | | 5 | | 7.67 | | 6 | | 1.93 | | 5 | | 4.16 | | 6 | | 3.03 | | 6 | | 7.10 | | 6 | | 4 | | | 4 | | 8 | |  |  |  |  |
| P26641 | Elongation factor 1-gamma | EEF1G | 24.03 | 8 | | 50.1 | | 6.67 | | 3.71 | 4 | | 3.81 | | 6 | | 4.41 | | 6 | | 4.51 | | 5 | | 2.81 | | 5 | | 4.34 | | 6 | | 4.44 | | 6 | | 4.94 | | 6 | | 4 | | | 4 | | 8 | |  |  |  |  |
| P35749 | Myosin-11 | MYH11 | 5.38 | 1 | | 227.2 | | 5.50 | | 2.31 | 4 | | 0.95 | | 3 | | 3.76 | | 6 | | 3.28 | | 5 | | 2.05 | | 5 | | 1.81 | | 4 | | 1.93 | | 4 | | 2.46 | | 5 | | 4 | | | 4 | | 8 | |  |  |  |  |
| P62314 | Small nuclear ribonucleoprotein Sm D1 | SNRPD1 | 36.97 | 3 | | 13.3 | | 11.56 | | 2.30 | 4 | | 3.25 | | 4 | | 3.38 | | 6 | | 2.24 | | 4 | | 5.46 | | 5 | | 2.22 | | 2 | | 8.22 | | 6 | | 6.82 | | 6 | | 4 | | | 4 | | 8 | |  |  |  |  |
| P20618 | Proteasome subunit beta type-1 | PSMB1 | 30.71 | 7 | | 26.5 | | 8.13 | | 3.49 | 4 | | 5.53 | | 5 | | 2.88 | | 5 | | 2.65 | | 4 | | 0.42 | | 2 | | 0.63 | | 2 | | 1.05 | | 5 | | 2.18 | | 6 | | 4 | | | 4 | | 8 | |  |  |  |  |
| P05386 | 60S acidic ribosomal protein P1 | RPLP1 | 28.95 | 1 | | 11.5 | | 4.32 | | 2.26 | 4 | | 2.65 | | 6 | | 2.72 | | 5 | | 3.23 | | 6 | | 3.75 | | 5 | | 1.61 | | 3 | | 5.16 | | 6 | | 4.74 | | 6 | | 4 | | | 4 | | 8 | |  |  |  |  |
| Q14103 | Heterogeneous nuclear ribonucleoprotein D0 | HNRNPD | 20.28 | 4 | | 38.4 | | 7.81 | | 2.30 | 4 | | 2.49 | | 6 | | 2.59 | | 6 | | 2.45 | | 6 | | 5.84 | | 6 | | 4.47 | | 6 | | 5.74 | | 6 | | 4.84 | | 6 | | 4 | | | 4 | | 8 | |  |  |  |  |
| P07355 | Annexin A2 | ANXA2 | 63.72 | 37 | | 38.6 | | 7.75 | | 7.79 | 4 | | 10.77 | | 5 | | 2.47 | | 4 | | 7.07 | | 4 | | 72.42 | | 6 | | 87.69 | | 6 | | 74.93 | | 6 | | 102.15 | | 6 | | 4 | | | 4 | | 8 | |  |  |  |  |
| P04004 | Vitronectin | VTN | 3.14 | 1 | | 54.3 | | 5.80 | | 1.52 | 4 | | 3.02 | | 6 | | 2.46 | | 4 | | 2.84 | | 6 | | 3.10 | | 5 | | 2.56 | | 4 | | 4.64 | | 6 | | 3.87 | | 6 | | 4 | | | 4 | | 8 | |  |  |  |  |
| O14979 | Heterogeneous nuclear ribonucleoprotein D-like | HNRNPDL | 10.24 | 1 | | 46.4 | | 9.57 | | 1.15 | 4 | | 1.41 | | 6 | | 2.27 | | 6 | | 2.12 | | 6 | | 2.60 | | 5 | | 1.05 | | 4 | | 2.14 | | 6 | | 1.93 | | 6 | | 4 | | | 4 | | 8 | |  |  |  |  |
| P17096 | High mobility group protein HMG-I/HMG-Y | HMGA1 | 41.12 | 4 | | 11.7 | | 10.32 | | 2.19 | 4 | | 2.92 | | 6 | | 2.21 | | 5 | | 2.22 | | 6 | | 11.52 | | 6 | | 6.43 | | 6 | | 11.15 | | 6 | | 7.86 | | 6 | | 4 | | | 4 | | 8 | |  |  |  |  |
| A0A0C4DG17 | 40S ribosomal protein SA | RPSA | 40.67 | 8 | | 33.3 | | 4.87 | | 1.89 | 4 | | 2.06 | | 5 | | 2.12 | | 6 | | 3.02 | | 6 | | 8.22 | | 6 | | 7.91 | | 6 | | 8.32 | | 6 | | 9.25 | | 6 | | 4 | | | 4 | | 8 | |  |  |  |  |
| G3V4C1 | Heterogeneous nuclear ribonucleoproteins C1/C2 | HNRNPC | 33.90 | 9 | | 32.2 | | 5.08 | | 3.82 | 4 | | 1.92 | | 2 | | 2.05 | | 5 | | 0.79 | | 3 | | 10.51 | | 6 | | 6.46 | | 6 | | 6.32 | | 6 | | 4.75 | | 6 | | 4 | | | 4 | | 8 | |  |  |  |  |
| Q14974 | Importin subunit beta-1 | KPNB1 | 8.90 | 6 | | 97.1 | | 4.78 | | 2.41 | 4 | | 2.58 | | 4 | | 1.83 | | 4 | | 0.84 | | 2 | | 1.61 | | 5 | | 1.90 | | 4 | | 1.75 | | 4 | | 1.64 | | 5 | | 4 | | | 4 | | 8 | |  |  |  |  |
| J3KQE5 | GTP-binding nuclear protein Ran (Fragment) | RAN | 31.20 | 7 | | 26.8 | | 9.58 | | 3.50 | 4 | | 3.95 | | 4 | | 1.70 | | 6 | | 2.38 | | 6 | | 7.23 | | 6 | | 10.23 | | 6 | | 10.63 | | 6 | | 11.03 | | 6 | | 4 | | | 4 | | 8 | |  |  |  |  |
| Q99456 | Keratin, type I cytoskeletal 12 | KRT12 | 3.24 | 1 | | 53.5 | | 4.78 | | 1.20 | 4 | | 0.64 | | 2 | | 1.58 | | 5 | | 0.96 | | 4 | | 1.43 | | 3 | | 2.77 | | 4 | | 1.62 | | 4 | | 1.58 | | 6 | | 4 | | | 4 | | 8 | |  |  |  |  |
| P05388 | 60S acidic ribosomal protein P0 | RPLP0 | 29.02 | 7 | | 34.3 | | 5.97 | | 3.34 | 4 | | 2.17 | | 4 | | 1.55 | | 4 | | 1.12 | | 4 | | 6.65 | | 4 | | 2.07 | | 5 | | 6.03 | | 6 | | 5.29 | | 6 | | 4 | | | 4 | | 8 | |  |  |  |  |
| J3KQ32 | Obg-like ATPase 1 | OLA1 | 7.69 | 2 | | 46.9 | | 8.06 | | 0.86 | 4 | | 0.98 | | 4 | | 1.52 | | 4 | | 0.71 | | 3 | | 0.28 | | 1 | | 0.16 | | 1 | | 0.44 | | 3 | | 0.15 | | 1 | | 4 | | | 4 | | 8 | |  |  |  |  |
| J3QQX2 | Rho GDP-dissociation inhibitor 1 | ARHGDIA | 19.57 | 4 | | 25.8 | | 7.44 | | 1.23 | 4 | | 1.60 | | 5 | | 1.49 | | 5 | | 1.41 | | 4 | | 0.32 | | 2 | | 1.25 | | 4 | | 0.43 | | 2 | | 0.31 | | 1 | | 4 | | | 4 | | 8 | |  |  |  |  |
| O15230 | Laminin subunit alpha-5 | LAMA5 | 9.82 | 24 | | 399.5 | | 7.02 | | 5.96 | 4 | | 3.51 | | 6 | | 1.45 | | 4 | | 0.84 | | 2 | | 3.75 | | 5 | | 9.89 | | 6 | | 2.39 | | 4 | | 2.91 | | 5 | | 4 | | | 4 | | 8 | |  |  |  |  |
| P46783 | 40S ribosomal protein S10 | RPS10 | 24.24 | 3 | | 18.9 | | 10.15 | | 1.31 | 4 | | 1.57 | | 6 | | 1.42 | | 4 | | 0.62 | | 2 | | 3.00 | | 6 | | 0.87 | | 3 | | 2.97 | | 5 | | 2.31 | | 5 | | 4 | | | 4 | | 8 | |  |  |  |  |
| P60900 | Proteasome subunit alpha type-6 | PSMA6 | 42.68 | 7 | | 27.4 | | 6.76 | | 1.89 | 4 | | 2.49 | | 4 | | 1.42 | | 2 | | 2.84 | | 4 | | 2.84 | | 5 | | 3.67 | | 4 | | 4.21 | | 6 | | 4.83 | | 6 | | 4 | | | 4 | | 8 | |  |  |  |  |
| P09429 | High mobility group protein B1 | HMGB1 | 26.05 | 4 | | 24.9 | | 5.74 | | 1.02 | 4 | | 1.37 | | 3 | | 1.30 | | 4 | | 1.51 | | 4 | | 0.84 | | 3 | | 1.01 | | 3 | | 0.14 | | 1 | | 0.18 | | 1 | | 4 | | | 4 | | 8 | |  |  |  |  |
| P61978 | Heterogeneous nuclear ribonucleoprotein K | HNRNPK | 25.70 | 11 | | 50.9 | | 5.54 | | 2.21 | 4 | | 2.03 | | 4 | | 0.94 | | 4 | | 1.37 | | 4 | | 10.00 | | 5 | | 3.02 | | 5 | | 8.26 | | 6 | | 4.16 | | 5 | | 4 | | | 4 | | 8 | |  |  |  |  |
| P68431 | Histone H3.1 | H3C1 | 48.53 | 4 | | 15.4 | | 11.12 | | 1.35 | 4 | | 1.27 | | 3 | | 0.91 | | 5 | | 1.26 | | 5 | | 9.79 | | 6 | | 6.53 | | 6 | | 13.53 | | 6 | | 11.87 | | 6 | | 4 | | | 4 | | 8 | |  |  |  |  |
| Q5TEC6 | Histone H3 | H3-2 | 25.00 | 1 | | 15.4 | | 11.27 | | 1.35 | 4 | | 1.27 | | 3 | | 0.91 | | 5 | | 1.10 | | 5 | | 7.35 | | 6 | | 5.20 | | 6 | | 7.98 | | 6 | | 7.06 | | 6 | | 4 | | | 4 | | 8 | |  |  |  |  |
| Q13838 | Spliceosome RNA helicase DDX39B | DDX39B | 13.32 | 5 | | 49.0 | | 5.67 | | 2.27 | 4 | | 2.44 | | 4 | | 0.89 | | 2 | | 0.84 | | 2 | | 1.75 | | 4 | | 0.63 | | 2 | | 1.37 | | 4 | | 0.49 | | 3 | | 4 | | | 4 | | 8 | |  |  |  |  |
| P19338 | Nucleolin | NCL | 21.13 | 12 | | 76.6 | | 4.70 | | 2.08 | 4 | | 3.14 | | 4 | | 0.83 | | 3 | | 1.10 | | 2 | | 9.79 | | 6 | | 5.31 | | 6 | | 4.49 | | 6 | | 4.04 | | 6 | | 4 | | | 4 | | 8 | |  |  |  |  |
| O75083 | WD repeat-containing protein 1 | WDR1 | 36.14 | 14 | | 66.2 | | 6.65 | | 2.23 | 4 | | 3.12 | | 4 | | 0.60 | | 2 | | 1.89 | | 6 | | 5.22 | | 6 | | 10.75 | | 6 | | 8.23 | | 6 | | 9.53 | | 6 | | 4 | | | 4 | | 8 | |  |  |  |  |
| P55060 | Exportin-2 | CSE1L | 5.56 | 3 | | 110.3 | | 5.77 | | 1.22 | 4 | | 0.73 | | 3 | | 0.16 | | 1 | | 0.73 | | 4 | | 4.13 | | 4 | | 2.20 | | 6 | | 1.38 | | 6 | | 0.51 | | 2 | | 4 | | | 4 | | 8 | |  |  |  |  |
| Q09666 | Neuroblast differentiation-associated protein AHNAK | AHNAK | 36.16 | 80 | | 628.7 | | 6.15 | | 2.90 | 3 | | 9.08 | | 5 | | 3.92 | | 5 | | 7.74 | | 6 | | 6.06 | | 3 | | 28.43 | | 4 | | 4.84 | | 6 | | 36.28 | | 6 | | 4 | | | 4 | | 8 | |  |  |  |  |
| Q5XKE5 | Keratin, type II cytoskeletal 79 | KRT79 | 13.27 | 1 | | 57.8 | | 7.20 | | 1.88 | 3 | | 0.32 | | 2 | | 2.86 | | 6 | | 0.82 | | 3 | | 3.50 | | 3 | | 7.27 | | 6 | | 2.19 | | 4 | | 1.68 | | 4 | | 4 | | | 4 | | 8 | |  |  |  |  |
| P40227 | T-complex protein 1 subunit zeta | CCT6A | 34.65 | 14 | | 58.0 | | 6.68 | | 1.72 | 3 | | 0.54 | | 3 | | 2.06 | | 5 | | 2.09 | | 4 | | 13.28 | | 5 | | 10.15 | | 4 | | 18.73 | | 6 | | 17.60 | | 6 | | 4 | | | 4 | | 8 | |  |  |  |  |
| P35580 | Myosin-10 | MYH10 | 8.50 | 3 | | 228.9 | | 5.54 | | 0.61 | 3 | | 0.14 | | 1 | | 1.80 | | 4 | | 1.83 | | 4 | | 2.08 | | 5 | | 3.64 | | 6 | | 2.47 | | 6 | | 2.49 | | 5 | | 4 | | | 4 | | 8 | |  |  |  |  |
| P55072 | Transitional endoplasmic reticulum ATPase | VCP | 26.92 | 16 | | 89.3 | | 5.26 | | 1.11 | 3 | | 4.29 | | 6 | | 1.76 | | 3 | | 4.17 | | 6 | | 3.67 | | 5 | | 6.84 | | 6 | | 7.18 | | 6 | | 10.69 | | 6 | | 4 | | | 4 | | 8 | |  |  |  |  |
| P28066 | Proteasome subunit alpha type-5 | PSMA5 | 18.26 | 3 | | 26.4 | | 4.79 | | 1.46 | 3 | | 1.98 | | 5 | | 1.22 | | 4 | | 1.61 | | 4 | | 0.91 | | 4 | | 1.99 | | 4 | | 2.77 | | 6 | | 4.41 | | 6 | | 4 | | | 4 | | 8 | |  |  |  |  |
| P28070 | Proteasome subunit beta type-4 | PSMB4 | 37.88 | 6 | | 29.2 | | 5.97 | | 1.18 | 3 | | 1.54 | | 4 | | 1.13 | | 3 | | 0.93 | | 2 | | 1.57 | | 4 | | 1.97 | | 4 | | 5.00 | | 6 | | 7.33 | | 6 | | 4 | | | 4 | | 8 | |  |  |  |  |
| Q8IUE6 | Histone H2A type 2-B | H2AC21 | 63.08 | 3 | | 14.0 | | 10.89 | | 2.00 | 3 | | 0.43 | | 3 | | 1.05 | | 2 | | 0.68 | | 4 | | 12.05 | | 6 | | 5.41 | | 6 | | 12.28 | | 6 | | 8.00 | | 6 | | 4 | | | 4 | | 8 | |  |  |  |  |
| Q02952 | A-kinase anchor protein 12 | AKAP12 | 12.51 | 14 | | 191.4 | | 4.41 | | 1.25 | 3 | | 5.07 | | 6 | | 0.97 | | 3 | | 2.05 | | 4 | | 3.01 | | 3 | | 2.53 | | 5 | | 1.02 | | 3 | | 2.12 | | 3 | | 4 | | | 4 | | 8 | |  |  |  |  |
| P22314 | Ubiquitin-like modifier-activating enzyme 1 | UBA1 | 28.17 | 15 | | 117.8 | | 5.76 | | 0.45 | 3 | | 1.00 | | 6 | | 0.85 | | 3 | | 0.49 | | 2 | | 5.68 | | 5 | | 4.18 | | 5 | | 6.78 | | 6 | | 3.14 | | 5 | | 4 | | | 4 | | 8 | |  |  |  |  |
| Q00839 | Heterogeneous nuclear ribonucleoprotein U | HNRNPU | 19.27 | 17 | | 90.5 | | 6.00 | | 0.45 | 3 | | 1.11 | | 4 | | 0.70 | | 3 | | 0.37 | | 2 | | 8.53 | | 5 | | 4.85 | | 4 | | 7.49 | | 6 | | 1.06 | | 4 | | 4 | | | 4 | | 8 | |  |  |  |  |
| P67809 | Y-box-binding protein 1 | YBX1 | 32.72 | 7 | | 35.9 | | 9.88 | | 0.94 | 3 | | 0.80 | | 2 | | 0.57 | | 2 | | 0.62 | | 2 | | 2.25 | | 3 | | 1.63 | | 2 | | 4.64 | | 4 | | 6.57 | | 6 | | 4 | | | 4 | | 8 | |  |  |  |  |
| P28072 | Proteasome subunit beta type-6 | PSMB6 | 37.66 | 6 | | 25.3 | | 4.92 | | 1.34 | 3 | | 0.97 | | 4 | | 0.57 | | 3 | | 0.78 | | 2 | | 1.98 | | 5 | | 3.54 | | 4 | | 5.04 | | 6 | | 5.86 | | 6 | | 4 | | | 4 | | 8 | |  |  |  |  |
| P46777 | 60S ribosomal protein L5 | RPL5 | 12.12 | 3 | | 34.3 | | 9.72 | | 0.76 | 3 | | 1.50 | | 4 | | 0.44 | | 2 | | 1.17 | | 4 | | 1.97 | | 3 | | 0.95 | | 2 | | 1.69 | | 5 | | 1.27 | | 4 | | 4 | | | 4 | | 8 | |  |  |  |  |
| P80723 | Brain acid soluble protein 1 | BASP1 | 76.65 | 9 | | 22.7 | | 4.63 | | 0.93 | 3 | | 1.64 | | 4 | | 0.38 | | 2 | | 0.16 | | 1 | | 11.00 | | 6 | | 13.72 | | 6 | | 5.78 | | 6 | | 7.71 | | 6 | | 4 | | | 4 | | 8 | |  |  |  |  |
| P62081 | 40S ribosomal protein S7 | RPS7 | 50.00 | 8 | | 22.1 | | 10.10 | | 0.45 | 3 | | 0.68 | | 3 | | 0.16 | | 1 | | 0.16 | | 1 | | 5.64 | | 5 | | 1.58 | | 2 | | 5.11 | | 6 | | 3.09 | | 5 | | 4 | | | 4 | | 8 | |  |  |  |  |
| P25789 | Proteasome subunit alpha type-4 | PSMA4 | 15.71 | 4 | | 29.5 | | 7.72 | | 1.18 | 3 | | 1.58 | | 4 | | 0.16 | | 1 | | 2.39 | | 4 | | 0.99 | | 3 | | 1.29 | | 3 | | 1.45 | | 5 | | 3.77 | | 6 | | 4 | | | 4 | | 8 | |  |  |  |  |
| E9PRY8 | Elongation factor 1-delta | EEF1D | 8.61 | 4 | | 76.5 | | 7.05 | | 0.45 | 3 | | 0.68 | | 3 | | 0.16 | | 1 | | 0.42 | | 2 | | 0.57 | | 2 | | 0.87 | | 3 | | 0.30 | | 2 | | 0.46 | | 3 | | 4 | | | 4 | | 8 | |  |  |  |  |
| Q15233 | Non-POU domain-containing octamer-binding protein | NONO | 17.83 | 7 | | 54.2 | | 8.95 | | 0.95 | 2 | | 0.55 | | 2 | | 1.96 | | 6 | | 0.42 | | 1 | | 6.97 | | 5 | | 2.68 | | 5 | | 7.06 | | 6 | | 3.05 | | 6 | | 4 | | | 4 | | 8 | |  |  |  |  |
| Q14195 | Dihydropyrimidinase-related protein 3 | DPYSL3 | 29.30 | 9 | | 61.9 | | 6.49 | | 1.12 | 2 | | 1.09 | | 2 | | 1.48 | | 5 | | 2.53 | | 6 | | 2.90 | | 5 | | 3.34 | | 5 | | 8.54 | | 6 | | 5.60 | | 6 | | 4 | | | 4 | | 8 | |  |  |  |  |
| Q08211 | ATP-dependent RNA helicase A | DHX9 | 15.98 | 13 | | 140.9 | | 6.84 | | 1.12 | 2 | | 0.27 | | 2 | | 1.33 | | 2 | | 0.42 | | 2 | | 6.65 | | 4 | | 1.77 | | 3 | | 4.97 | | 6 | | 1.27 | | 4 | | 4 | | | 4 | | 8 | |  |  |  |  |
| O00299 | Chloride intracellular channel protein 1 | CLIC1 | 35.27 | 6 | | 26.9 | | 5.17 | | 1.59 | 2 | | 2.32 | | 4 | | 1.11 | | 2 | | 2.80 | | 6 | | 0.30 | | 2 | | 0.86 | | 4 | | 1.67 | | 5 | | 0.79 | | 4 | | 4 | | | 4 | | 8 | |  |  |  |  |
| O14818 | Proteasome subunit alpha type-7 | PSMA7 | 44.35 | 9 | | 27.9 | | 8.46 | | 2.44 | 2 | | 2.84 | | 5 | | 0.94 | | 2 | | 1.71 | | 2 | | 2.03 | | 5 | | 2.90 | | 4 | | 5.71 | | 6 | | 7.20 | | 6 | | 4 | | | 4 | | 8 | |  |  |  |  |
| P28074 | Proteasome subunit beta type-5 | PSMB5 | 25.86 | 7 | | 28.5 | | 6.92 | | 1.16 | 2 | | 2.31 | | 4 | | 0.47 | | 2 | | 1.11 | | 4 | | 0.62 | | 3 | | 1.27 | | 2 | | 3.94 | | 6 | | 4.25 | | 6 | | 4 | | | 4 | | 8 | |  |  |  |  |
| Q96M42 | Putative uncharacterized protein encoded by LINC00479 | LINC00479 | 4.93 | 1 | | 15.2 | | 8.00 | | 0.42 | 2 | | 0.70 | | 4 | | 0.34 | | 2 | | 0.53 | | 3 | | 0.89 | | 4 | | 0.41 | | 2 | | 0.14 | | 1 | | 1.12 | | 5 | | 4 | | | 4 | | 8 | |  |  |  |  |
| O15145 | Actin-related protein 2/3 complex subunit 3 | ARPC3 | 13.48 | 2 | | 20.5 | | 8.59 | | 0.51 | 2 | | 1.00 | | 5 | | 0.16 | | 1 | | 0.66 | | 2 | | 0.99 | | 3 | | 0.23 | | 1 | | 0.65 | | 3 | | 0.15 | | 1 | | 4 | | | 4 | | 8 | |  |  |  |  |
| P62318 | Small nuclear ribonucleoprotein Sm D3 | SNRPD3 | 15.08 | 2 | | 13.9 | | 10.32 | | 0.15 | 1 | | 0.55 | | 3 | | 0.53 | | 2 | | 0.83 | | 3 | | 1.97 | | 3 | | 1.11 | | 2 | | 2.99 | | 6 | | 2.47 | | 6 | | 4 | | | 4 | | 8 | |  |  |  |  |
| A0A087WUZ3 | Spectrin beta chain | SPTBN1 | 5.24 | 8 | | 274.7 | | 5.57 | | 0.13 | 1 | | 0.14 | | 1 | | 0.34 | | 2 | | 0.50 | | 2 | | 2.48 | | 5 | | 0.16 | | 1 | | 0.60 | | 3 | | 0.15 | | 1 | | 4 | | | 4 | | 8 | |  |  |  |  |
| P23526 | Adenosylhomocysteinase | AHCY | 19.44 | 6 | | 47.7 | | 6.34 | | 0.31 | 1 | | 0.80 | | 3 | | 0.16 | | 1 | | 0.17 | | 1 | | 0.99 | | 3 | | 2.99 | | 4 | | 2.34 | | 4 | | 2.51 | | 6 | | 4 | | | 4 | | 8 | |  |  |  |  |
| P67936 | Tropomyosin alpha-4 chain | TPM4 | 44.76 | 6 | | 28.5 | | 4.69 | | 15.17 | 6 | | 18.81 | | 6 | | 19.37 | | 6 | | 22.70 | | 6 | | 0.00 | | 0 | | 0.86 | | 3 | | 1.16 | | 3 | | 0.46 | | 2 | | 4 | | | 3 | | 7 | |  |  |  |  |
| H7BYY1 | Tropomyosin 1 (Alpha), isoform CRA_m | TPM1 | 43.55 | 2 | | 28.7 | | 4.82 | | 8.19 | 6 | | 11.07 | | 6 | | 10.14 | | 6 | | 10.91 | | 6 | | 0.00 | | 0 | | 0.39 | | 2 | | 1.16 | | 3 | | 0.46 | | 2 | | 4 | | | 3 | | 7 | |  |  |  |  |
| A0A0S2Z4G6 | Tropomyosin 1 (Alpha), isoform CRA_o (Fragment) | TPM1 | 41.20 | 1 | | 32.7 | | 4.74 | | 7.52 | 6 | | 10.01 | | 6 | | 9.73 | | 6 | | 11.69 | | 6 | | 0.00 | | 0 | | 0.39 | | 2 | | 1.16 | | 3 | | 0.46 | | 2 | | 4 | | | 3 | | 7 | |  |  |  |  |
| Q14019 | Coactosin-like protein | COTL1 | 42.96 | 7 | | 15.9 | | 5.67 | | 12.53 | 6 | | 11.10 | | 6 | | 9.34 | | 6 | | 11.46 | | 6 | | 0.49 | | 2 | | 0.96 | | 3 | | 0.00 | | 0 | | 0.61 | | 2 | | 4 | | | 3 | | 7 | |  |  |  |  |
| Q6ZN40 | Tropomyosin 1 (Alpha), isoform CRA_f | TPM1 | 41.10 | 0 | | 37.4 | | 4.72 | | 7.05 | 6 | | 11.44 | | 6 | | 9.23 | | 6 | | 11.93 | | 6 | | 0.00 | | 0 | | 0.39 | | 2 | | 1.16 | | 3 | | 0.46 | | 2 | | 4 | | | 3 | | 7 | |  |  |  |  |
| A0A087WWU8 | Tropomyosin alpha-3 chain | TPM3 | 49.34 | 3 | | 26.4 | | 4.78 | | 8.07 | 6 | | 12.39 | | 6 | | 8.90 | | 6 | | 13.40 | | 6 | | 0.00 | | 0 | | 0.63 | | 2 | | 1.16 | | 3 | | 0.46 | | 2 | | 4 | | | 3 | | 7 | |  |  |  |  |
| P19823 | Inter-alpha-trypsin inhibitor heavy chain H2 | ITIH2 | 8.46 | 6 | | 106.4 | | 6.86 | | 5.77 | 6 | | 2.65 | | 6 | | 6.62 | | 6 | | 7.61 | | 6 | | 3.11 | | 5 | | 2.58 | | 4 | | 0.00 | | 0 | | 1.43 | | 2 | | 4 | | | 3 | | 7 | |  |  |  |  |
| J3KN67 | Tropomyosin alpha-3 chain | TPM3 | 47.37 | 1 | | 33.2 | | 4.77 | | 6.28 | 6 | | 9.74 | | 6 | | 6.20 | | 6 | | 10.82 | | 6 | | 0.00 | | 0 | | 0.63 | | 2 | | 1.16 | | 3 | | 0.46 | | 2 | | 4 | | | 3 | | 7 | |  |  |  |  |
| P07996 | Thrombospondin-1 | THBS1 | 25.38 | 21 | | 129.3 | | 4.94 | | 32.69 | 6 | | 25.55 | | 6 | | 2.48 | | 5 | | 3.07 | | 3 | | 6.92 | | 3 | | 14.47 | | 6 | | 0.15 | | 1 | | 0.00 | | 0 | | 4 | | | 3 | | 7 | |  |  |  |  |
| A0A0D9SGF6 | Spectrin alpha chain, non-erythrocytic 1 | SPTAN1 | 13.01 | 21 | | 287.4 | | 5.36 | | 5.04 | 5 | | 6.09 | | 6 | | 6.25 | | 6 | | 3.49 | | 5 | | 1.83 | | 3 | | 0.00 | | 0 | | 0.45 | | 2 | | 0.46 | | 2 | | 4 | | | 3 | | 7 | |  |  |  |  |
| Q92626 | Peroxidasin homolog | PXDN | 17.04 | 16 | | 165.2 | | 7.17 | | 6.28 | 5 | | 7.68 | | 6 | | 4.32 | | 6 | | 4.80 | | 5 | | 1.13 | | 2 | | 3.49 | | 4 | | 1.49 | | 4 | | 0.00 | | 0 | | 4 | | | 3 | | 7 | |  |  |  |  |
| P20908 | Collagen alpha-1(V) chain | COL5A1 | 6.86 | 8 | | 183.4 | | 5.06 | | 2.30 | 5 | | 2.08 | | 6 | | 2.09 | | 4 | | 2.62 | | 5 | | 0.00 | | 0 | | 0.91 | | 2 | | 0.48 | | 2 | | 0.87 | | 3 | | 4 | | | 3 | | 7 | |  |  |  |  |
| Q15582 | Transforming growth factor-beta-induced protein ig-h3 | TGFBI | 35.58 | 19 | | 74.6 | | 7.71 | | 9.97 | 4 | | 9.77 | | 6 | | 12.65 | | 6 | | 8.46 | | 5 | | 0.00 | | 0 | | 2.06 | | 3 | | 0.14 | | 1 | | 0.46 | | 3 | | 4 | | | 3 | | 7 | |  |  |  |  |
| Q01518 | Adenylyl cyclase-associated protein 1 | CAP1 | 29.26 | 8 | | 51.9 | | 8.06 | | 3.88 | 4 | | 6.10 | | 4 | | 7.75 | | 6 | | 3.78 | | 6 | | 0.30 | | 2 | | 0.79 | | 2 | | 0.00 | | 0 | | 0.92 | | 3 | | 4 | | | 3 | | 7 | |  |  |  |  |
| P22626 | Heterogeneous nuclear ribonucleoproteins A2/B1 | HNRNPA2B1 | 41.64 | 10 | | 37.4 | | 8.95 | | 4.27 | 4 | | 4.35 | | 4 | | 6.48 | | 6 | | 7.58 | | 6 | | 4.23 | | 3 | | 1.39 | | 4 | | 3.51 | | 4 | | 0.00 | | 0 | | 4 | | | 3 | | 7 | |  |  |  |  |
| P62857 | 40S ribosomal protein S28 | RPS28 | 30.43 | 2 | | 7.8 | | 10.70 | | 1.30 | 4 | | 3.03 | | 6 | | 3.65 | | 6 | | 3.83 | | 6 | | 0.00 | | 0 | | 0.37 | | 1 | | 0.76 | | 4 | | 2.24 | | 6 | | 4 | | | 3 | | 7 | |  |  |  |  |
| Q9ULV4 | Coronin-1C | CORO1C | 18.14 | 5 | | 53.2 | | 7.08 | | 2.79 | 4 | | 1.77 | | 4 | | 2.90 | | 6 | | 1.00 | | 3 | | 0.00 | | 0 | | 0.16 | | 1 | | 0.87 | | 3 | | 1.52 | | 4 | | 4 | | | 3 | | 7 | |  |  |  |  |
| P60981 | Destrin | DSTN | 26.06 | 4 | | 18.5 | | 7.85 | | 1.15 | 4 | | 1.22 | | 4 | | 2.08 | | 6 | | 2.28 | | 6 | | 0.70 | | 3 | | 0.32 | | 2 | | 2.17 | | 4 | | 0.00 | | 0 | | 4 | | | 3 | | 7 | |  |  |  |  |
| Q07666 | KH domain-containing, RNA-binding, sial transduction-associated protein 1 | KHDRBS1 | 11.96 | 4 | | 48.2 | | 8.66 | | 1.15 | 4 | | 1.77 | | 4 | | 1.68 | | 4 | | 0.80 | | 3 | | 1.97 | | 3 | | 0.32 | | 2 | | 0.17 | | 1 | | 0.00 | | 0 | | 4 | | | 3 | | 7 | |  |  |  |  |
| Q58FG0 | Putative heat shock protein HSP 90-alpha A5 | HSP90AA5P | 13.47 | 1 | | 38.7 | | 6.57 | | 1.62 | 4 | | 1.90 | | 4 | | 1.52 | | 4 | | 1.66 | | 5 | | 1.27 | | 3 | | 0.95 | | 2 | | 0.31 | | 2 | | 0.00 | | 0 | | 4 | | | 3 | | 7 | |  |  |  |  |
| I3L504 | Eukaryotic translation initiation factor 5A-1 | EIF5A | 25.81 | 5 | | 20.5 | | 5.25 | | 1.41 | 4 | | 3.68 | | 6 | | 1.52 | | 4 | | 1.65 | | 5 | | 0.14 | | 1 | | 0.00 | | 0 | | 0.50 | | 1 | | 0.18 | | 1 | | 4 | | | 3 | | 7 | |  |  |  |  |
| P05387 | 60S acidic ribosomal protein P2 | RPLP2 | 90.43 | 7 | | 11.7 | | 4.54 | | 1.02 | 4 | | 0.81 | | 3 | | 0.31 | | 2 | | 0.93 | | 2 | | 4.65 | | 3 | | 0.00 | | 0 | | 5.86 | | 4 | | 3.90 | | 4 | | 4 | | | 3 | | 7 | |  |  |  |  |
| P24844 | Myosin regulatory light polypeptide 9 | MYL9 | 44.19 | 2 | | 19.8 | | 4.92 | | 0.81 | 3 | | 4.81 | | 6 | | 4.02 | | 5 | | 8.67 | | 6 | | 0.14 | | 1 | | 0.00 | | 0 | | 0.77 | | 4 | | 0.54 | | 2 | | 4 | | | 3 | | 7 | |  |  |  |  |
| P13797 | Plastin-3 | PLS3 | 7.78 | 4 | | 70.8 | | 5.60 | | 0.64 | 2 | | 0.27 | | 2 | | 0.34 | | 2 | | 0.49 | | 2 | | 0.14 | | 1 | | 0.73 | | 3 | | 0.00 | | 0 | | 0.33 | | 2 | | 4 | | | 3 | | 7 | |  |  |  |  |
| A0A075B6G3 | Dystrophin | DMD | 1.03 | 3 | | 426.5 | | 5.90 | | 0.57 | 2 | | 0.64 | | 2 | | 0.19 | | 1 | | 0.16 | | 1 | | 1.24 | | 4 | | 1.54 | | 3 | | 0.15 | | 1 | | 0.00 | | 0 | | 4 | | | 3 | | 7 | |  |  |  |  |
| Q15019 | Septin-2 | SEPTIN2 | 21.61 | 6 | | 41.5 | | 6.60 | | 0.26 | 1 | | 0.70 | | 3 | | 0.47 | | 2 | | 0.16 | | 1 | | 1.27 | | 3 | | 0.00 | | 0 | | 1.27 | | 3 | | 0.18 | | 1 | | 4 | | | 3 | | 7 | |  |  |  |  |
| H3BPE7 | RNA-binding protein FUS | FUS | 7.59 | 2 | | 53.5 | | 9.36 | | 0.15 | 1 | | 0.55 | | 2 | | 0.16 | | 1 | | 0.62 | | 2 | | 1.55 | | 3 | | 0.48 | | 2 | | 0.14 | | 1 | | 0.00 | | 0 | | 4 | | | 3 | | 7 | |  |  |  |  |
| P05121 | Plasminogen activator inhibitor 1 | SERPINE1 | 66.67 | 19 | | 45.0 | | 7.20 | | 76.48 | 6 | | 50.56 | | 6 | | 54.20 | | 6 | | 41.80 | | 6 | | 0.00 | | 0 | | 0.54 | | 2 | | 0.34 | | 2 | | 0.00 | | 0 | | 4 | | | 2 | | 6 | |  |  |  |  |
| P07237 | Protein disulfide-isomerase | P4HB | 45.67 | 17 | | 57.1 | | 4.87 | | 5.18 | 6 | | 10.80 | | 5 | | 8.99 | | 6 | | 12.48 | | 5 | | 0.00 | | 0 | | 0.24 | | 1 | | 0.00 | | 0 | | 0.15 | | 1 | | 4 | | | 2 | | 6 | |  |  |  |  |
| P24593 | Insulin-like growth factor-binding protein 5 | IGFBP5 | 16.18 | 4 | | 30.6 | | 8.21 | | 3.09 | 6 | | 2.32 | | 5 | | 6.17 | | 6 | | 6.08 | | 6 | | 0.00 | | 0 | | 0.00 | | 0 | | 0.15 | | 1 | | 0.15 | | 1 | | 4 | | | 2 | | 6 | |  |  |  |  |
| P12109 | Collagen alpha-1(VI) chain | COL6A1 | 8.07 | 6 | | 108.5 | | 5.43 | | 9.10 | 6 | | 4.16 | | 4 | | 4.86 | | 6 | | 3.99 | | 5 | | 0.00 | | 0 | | 0.00 | | 0 | | 0.17 | | 1 | | 2.13 | | 3 | | 4 | | | 2 | | 6 | |  |  |  |  |
| P35555 | Fibrillin-1 | FBN1 | 3.00 | 3 | | 312.1 | | 4.93 | | 2.77 | 6 | | 2.66 | | 6 | | 3.09 | | 5 | | 1.42 | | 5 | | 0.28 | | 2 | | 0.00 | | 0 | | 0.00 | | 0 | | 0.15 | | 1 | | 4 | | | 2 | | 6 | |  |  |  |  |
| P15259 | Phosphoglycerate mutase 2 | PGAM2 | 20.95 | 1 | | 28.7 | | 8.88 | | 3.13 | 6 | | 2.79 | | 6 | | 2.74 | | 6 | | 2.28 | | 6 | | 0.00 | | 0 | | 0.00 | | 0 | | 0.44 | | 2 | | 0.97 | | 4 | | 4 | | | 2 | | 6 | |  |  |  |  |
| P19022 | Cadherin-2 | CDH2 | 16.45 | 9 | | 99.7 | | 4.81 | | 5.19 | 6 | | 5.32 | | 6 | | 1.39 | | 4 | | 2.05 | | 4 | | 0.16 | | 1 | | 0.23 | | 1 | | 0.00 | | 0 | | 0.00 | | 0 | | 4 | | | 2 | | 6 | |  |  |  |  |
| P61769 | Beta-2-microglobulin | B2M | 16.81 | 2 | | 13.7 | | 6.52 | | 2.16 | 5 | | 2.08 | | 5 | | 3.63 | | 6 | | 3.60 | | 6 | | 0.00 | | 0 | | 0.00 | | 0 | | 0.17 | | 1 | | 0.54 | | 2 | | 4 | | | 2 | | 6 | |  |  |  |  |
| P38159 | RNA-binding motif protein, X chromosome | RBMX | 14.32 | 5 | | 42.3 | | 10.05 | | 1.73 | 4 | | 2.31 | | 4 | | 3.02 | | 5 | | 1.04 | | 3 | | 0.85 | | 3 | | 0.16 | | 1 | | 0.00 | | 0 | | 0.00 | | 0 | | 4 | | | 2 | | 6 | |  |  |  |  |
| P37837 | Transaldolase | TALDO1 | 20.77 | 7 | | 37.5 | | 6.81 | | 3.12 | 4 | | 3.40 | | 4 | | 1.64 | | 3 | | 0.31 | | 2 | | 0.00 | | 0 | | 1.59 | | 2 | | 0.00 | | 0 | | 1.06 | | 2 | | 4 | | | 2 | | 6 | |  |  |  |  |
| P07093 | Glia-derived nexin | SERPINE2 | 18.84 | 5 | | 44.0 | | 9.29 | | 3.02 | 4 | | 2.32 | | 4 | | 0.82 | | 3 | | 0.63 | | 2 | | 1.13 | | 3 | | 1.27 | | 2 | | 0.00 | | 0 | | 0.00 | | 0 | | 4 | | | 2 | | 6 | |  |  |  |  |
| P10809 | 60 kDa heat shock protein, mitochondrial | HSPD1 | 23.21 | 10 | | 61.0 | | 5.87 | | 1.01 | 4 | | 8.45 | | 3 | | 0.56 | | 2 | | 2.15 | | 6 | | 0.00 | | 0 | | 0.23 | | 1 | | 0.00 | | 0 | | 0.30 | | 2 | | 4 | | | 2 | | 6 | |  |  |  |  |
| P02462 | Collagen alpha-1(IV) chain | COL4A1 | 3.54 | 4 | | 160.5 | | 8.28 | | 1.31 | 3 | | 0.46 | | 3 | | 2.31 | | 4 | | 0.63 | | 3 | | 0.00 | | 0 | | 1.66 | | 4 | | 0.45 | | 2 | | 0.00 | | 0 | | 4 | | | 2 | | 6 | |  |  |  |  |
| P06396 | Gelsolin | GSN | 6.39 | 5 | | 85.6 | | 6.28 | | 0.80 | 3 | | 0.73 | | 5 | | 1.61 | | 5 | | 0.97 | | 4 | | 0.00 | | 0 | | 0.18 | | 1 | | 0.00 | | 0 | | 0.31 | | 1 | | 4 | | | 2 | | 6 | |  |  |  |  |
| P16949 | Stathmin | STMN1 | 34.23 | 2 | | 17.3 | | 5.97 | | 1.01 | 3 | | 2.34 | | 5 | | 1.51 | | 4 | | 1.27 | | 4 | | 0.62 | | 1 | | 0.24 | | 1 | | 0.00 | | 0 | | 0.00 | | 0 | | 4 | | | 2 | | 6 | |  |  |  |  |
| Q96FQ6 | Protein S100-A16 | S100A16 | 30.10 | 2 | | 11.8 | | 6.79 | | 0.42 | 3 | | 0.55 | | 3 | | 0.53 | | 3 | | 1.16 | | 4 | | 0.00 | | 0 | | 0.00 | | 0 | | 0.59 | | 3 | | 1.72 | | 6 | | 4 | | | 2 | | 6 | |  |  |  |  |
| P09972 | Fructose-bisphosphate aldolase C | ALDOC | 7.14 | 2 | | 39.4 | | 6.87 | | 1.03 | 2 | | 1.90 | | 4 | | 2.17 | | 4 | | 1.81 | | 5 | | 0.00 | | 0 | | 0.00 | | 0 | | 0.72 | | 2 | | 0.30 | | 2 | | 4 | | | 2 | | 6 | |  |  |  |  |
| Q9Y696 | Chloride intracellular channel protein 4 | CLIC4 | 7.91 | 2 | | 28.8 | | 5.59 | | 0.42 | 2 | | 0.94 | | 3 | | 0.76 | | 4 | | 1.54 | | 5 | | 0.00 | | 0 | | 0.00 | | 0 | | 1.20 | | 5 | | 1.09 | | 4 | | 4 | | | 2 | | 6 | |  |  |  |  |
| P31629 | Transcription factor HIVEP2 | HIVEP2 | 2.90 | 3 | | 268.9 | | 6.96 | | 0.26 | 2 | | 0.91 | | 4 | | 0.70 | | 2 | | 0.32 | | 2 | | 0.14 | | 1 | | 0.24 | | 1 | | 0.00 | | 0 | | 0.00 | | 0 | | 4 | | | 2 | | 6 | |  |  |  |  |
| P55209 | Nucleosome assembly protein 1-like 1 | NAP1L1 | 19.18 | 4 | | 45.3 | | 4.46 | | 0.47 | 2 | | 0.68 | | 4 | | 0.22 | | 1 | | 0.21 | | 1 | | 1.70 | | 3 | | 0.00 | | 0 | | 0.43 | | 2 | | 0.00 | | 0 | | 4 | | | 2 | | 6 | |  |  |  |  |
| P11413 | Glucose-6-phosphate 1-dehydrogenase | G6PD | 8.93 | 4 | | 59.2 | | 6.84 | | 0.32 | 2 | | 0.14 | | 1 | | 0.22 | | 1 | | 0.21 | | 1 | | 0.14 | | 1 | | 0.00 | | 0 | | 0.87 | | 2 | | 0.00 | | 0 | | 4 | | | 2 | | 6 | |  |  |  |  |
| Q6ZWH5 | Serine/threonine-protein kinase Nek10 | NEK10 | 0.68 | 1 | | 133.2 | | 6.80 | | 0.28 | 2 | | 0.30 | | 2 | | 0.16 | | 1 | | 0.37 | | 2 | | 0.32 | | 1 | | 0.41 | | 2 | | 0.00 | | 0 | | 0.00 | | 0 | | 4 | | | 2 | | 6 | |  |  |  |  |
| P78348 | Acid-sensing ion channel 1 | ASIC1 | 3.98 | 1 | | 59.9 | | 5.73 | | 0.39 | 2 | | 0.14 | | 1 | | 0.16 | | 1 | | 0.16 | | 1 | | 0.28 | | 1 | | 0.16 | | 1 | | 0.00 | | 0 | | 0.00 | | 0 | | 4 | | | 2 | | 6 | |  |  |  |  |
| Q93045 | Stathmin-2 | STMN2 | 31.28 | 1 | | 20.8 | | 8.32 | | 0.33 | 1 | | 1.24 | | 5 | | 0.41 | | 2 | | 1.12 | | 4 | | 0.62 | | 1 | | 0.24 | | 1 | | 0.00 | | 0 | | 0.00 | | 0 | | 4 | | | 2 | | 6 | |  |  |  |  |
| Q12905 | Interleukin enhancer-binding factor 2 | ILF2 | 6.15 | 2 | | 43.0 | | 5.26 | | 0.15 | 1 | | 0.55 | | 2 | | 0.32 | | 1 | | 0.21 | | 1 | | 0.42 | | 3 | | 0.16 | | 1 | | 0.00 | | 0 | | 0.00 | | 0 | | 4 | | | 2 | | 6 | |  |  |  |  |
| P25398 | 40S ribosomal protein S12 | RPS12 | 13.64 | 2 | | 14.5 | | 7.21 | | 0.13 | 1 | | 1.22 | | 4 | | 0.16 | | 1 | | 0.16 | | 1 | | 0.56 | | 3 | | 0.00 | | 0 | | 0.67 | | 2 | | 0.00 | | 0 | | 4 | | | 2 | | 6 | |  |  |  |  |
| P09486 | SPARC | SPARC | 24.75 | 7 | | 34.6 | | 4.84 | | 27.17 | 6 | | 18.51 | | 6 | | 29.54 | | 6 | | 21.73 | | 6 | | 0.32 | | 2 | | 0.00 | | 0 | | 0.00 | | 0 | | 0.00 | | 0 | | 4 | | | 1 | | 5 | |  |  |  |  |
| Q12923 | Tyrosine-protein phosphatase non-receptor type 13 | PTPN13 | 1.13 | 2 | | 276.7 | | 6.42 | | 7.67 | 6 | | 6.94 | | 6 | | 9.78 | | 6 | | 9.05 | | 6 | | 0.16 | | 1 | | 0.00 | | 0 | | 0.00 | | 0 | | 0.00 | | 0 | | 4 | | | 1 | | 5 | |  |  |  |  |
| Q9Y4K0 | Lysyl oxidase homolog 2 | LOXL2 | 20.16 | 12 | | 86.7 | | 6.38 | | 9.68 | 6 | | 7.22 | | 6 | | 7.07 | | 6 | | 3.23 | | 5 | | 0.00 | | 0 | | 1.27 | | 4 | | 0.00 | | 0 | | 0.00 | | 0 | | 4 | | | 1 | | 5 | |  |  |  |  |
| P02788 | Lactotransferrin | LTF | 1.27 | 1 | | 78.1 | | 8.12 | | 3.83 | 6 | | 2.13 | | 6 | | 4.01 | | 6 | | 3.35 | | 6 | | 0.00 | | 0 | | 0.00 | | 0 | | 0.00 | | 0 | | 0.18 | | 1 | | 4 | | | 1 | | 5 | |  |  |  |  |
| P10599 | Thioredoxin | TXN | 22.86 | 2 | | 11.7 | | 4.92 | | 3.71 | 6 | | 3.75 | | 6 | | 3.41 | | 6 | | 3.55 | | 6 | | 0.16 | | 1 | | 0.00 | | 0 | | 0.00 | | 0 | | 0.00 | | 0 | | 4 | | | 1 | | 5 | |  |  |  |  |
| Q9UNN8 | Endothelial protein C receptor | PROCR | 22.69 | 3 | | 26.7 | | 7.18 | | 2.61 | 6 | | 2.67 | | 6 | | 1.01 | | 3 | | 0.97 | | 4 | | 0.14 | | 1 | | 0.00 | | 0 | | 0.00 | | 0 | | 0.00 | | 0 | | 4 | | | 1 | | 5 | |  |  |  |  |
| P30101 | Protein disulfide-isomerase A3 | PDIA3 | 44.55 | 18 | | 56.7 | | 6.35 | | 8.79 | 4 | | 13.33 | | 4 | | 4.67 | | 4 | | 14.06 | | 4 | | 0.00 | | 0 | | 0.00 | | 0 | | 0.00 | | 0 | | 0.31 | | 2 | | 4 | | | 1 | | 5 | |  |  |  |  |
| P21291 | Cysteine and glycine-rich protein 1 | CSRP1 | 16.58 | 2 | | 20.6 | | 8.57 | | 1.63 | 4 | | 3.17 | | 6 | | 3.31 | | 6 | | 2.28 | | 5 | | 0.00 | | 0 | | 0.41 | | 2 | | 0.00 | | 0 | | 0.00 | | 0 | | 4 | | | 1 | | 5 | |  |  |  |  |
| O43852 | Calumenin | CALU | 57.14 | 14 | | 37.1 | | 4.64 | | 5.78 | 4 | | 7.36 | | 4 | | 2.98 | | 4 | | 8.89 | | 6 | | 0.00 | | 0 | | 0.00 | | 0 | | 0.00 | | 0 | | 0.46 | | 1 | | 4 | | | 1 | | 5 | |  |  |  |  |
| P08572 | Collagen alpha-2(IV) chain | COL4A2 | 9.87 | 10 | | 167.4 | | 8.66 | | 2.21 | 4 | | 1.88 | | 3 | | 2.36 | | 2 | | 1.18 | | 5 | | 0.00 | | 0 | | 4.10 | | 4 | | 0.00 | | 0 | | 0.00 | | 0 | | 4 | | | 1 | | 5 | |  |  |  |  |
| Q16881 | Thioredoxin reductase 1, cytoplasmic | TXNRD1 | 32.36 | 12 | | 70.9 | | 7.39 | | 6.26 | 4 | | 8.87 | | 6 | | 2.29 | | 4 | | 2.82 | | 6 | | 0.00 | | 0 | | 0.00 | | 0 | | 0.14 | | 1 | | 0.00 | | 0 | | 4 | | | 1 | | 5 | |  |  |  |  |
| P54819 | Adenylate kinase 2, mitochondrial | AK2 | 21.76 | 3 | | 26.5 | | 7.81 | | 1.66 | 4 | | 0.96 | | 2 | | 1.48 | | 5 | | 0.63 | | 2 | | 0.00 | | 0 | | 0.16 | | 1 | | 0.00 | | 0 | | 0.00 | | 0 | | 4 | | | 1 | | 5 | |  |  |  |  |
| Q15942 | Zyxin | ZYX | 19.93 | 7 | | 61.2 | | 6.67 | | 0.98 | 4 | | 1.24 | | 5 | | 1.00 | | 4 | | 1.89 | | 4 | | 0.00 | | 0 | | 0.23 | | 1 | | 0.00 | | 0 | | 0.00 | | 0 | | 4 | | | 1 | | 5 | |  |  |  |  |
| P49720 | Proteasome subunit beta type-3 | PSMB3 | 32.20 | 4 | | 22.9 | | 6.55 | | 1.22 | 4 | | 2.96 | | 6 | | 0.93 | | 1 | | 1.74 | | 4 | | 0.14 | | 1 | | 0.00 | | 0 | | 0.00 | | 0 | | 0.00 | | 0 | | 4 | | | 1 | | 5 | |  |  |  |  |
| Q9Y6V0 | Protein piccolo | PCLO | 0.72 | 3 | | 560.4 | | 6.47 | | 1.62 | 4 | | 0.41 | | 2 | | 0.79 | | 2 | | 0.69 | | 3 | | 0.14 | | 1 | | 0.00 | | 0 | | 0.00 | | 0 | | 0.00 | | 0 | | 4 | | | 1 | | 5 | |  |  |  |  |
| A8MU27 | Small ubiquitin-related modifier 3 | SUMO3 | 8.16 | 1 | | 16.9 | | 9.67 | | 0.83 | 4 | | 1.08 | | 4 | | 0.63 | | 2 | | 1.08 | | 3 | | 0.62 | | 1 | | 0.00 | | 0 | | 0.00 | | 0 | | 0.00 | | 0 | | 4 | | | 1 | | 5 | |  |  |  |  |
| P31948 | Stress-induced-phosphoprotein 1 | STIP1 | 7.55 | 4 | | 62.6 | | 6.80 | | 1.31 | 4 | | 0.54 | | 3 | | 0.31 | | 1 | | 0.53 | | 3 | | 0.56 | | 3 | | 0.00 | | 0 | | 0.00 | | 0 | | 0.00 | | 0 | | 4 | | | 1 | | 5 | |  |  |  |  |
| P07339 | Cathepsin D | CTSD | 5.10 | 2 | | 44.5 | | 6.54 | | 1.09 | 4 | | 0.27 | | 2 | | 0.19 | | 1 | | 1.09 | | 2 | | 0.00 | | 0 | | 0.00 | | 0 | | 0.00 | | 0 | | 0.30 | | 1 | | 4 | | | 1 | | 5 | |  |  |  |  |
| P30041 | Peroxiredoxin-6 | PRDX6 | 39.73 | 8 | | 25.0 | | 6.38 | | 1.76 | 3 | | 4.21 | | 4 | | 3.62 | | 6 | | 2.39 | | 5 | | 0.00 | | 0 | | 0.16 | | 1 | | 0.00 | | 0 | | 0.00 | | 0 | | 4 | | | 1 | | 5 | |  |  |  |  |
| P28300 | Protein-lysine 6-oxidase | LOX | 11.03 | 3 | | 46.9 | | 8.09 | | 0.67 | 3 | | 1.45 | | 6 | | 2.08 | | 6 | | 1.80 | | 5 | | 0.00 | | 0 | | 0.00 | | 0 | | 0.00 | | 0 | | 0.76 | | 2 | | 4 | | | 1 | | 5 | |  |  |  |  |
| P34932 | Heat shock 70 kDa protein 4 | HSPA4 | 8.81 | 5 | | 94.3 | | 5.19 | | 0.82 | 3 | | 1.68 | | 6 | | 1.60 | | 5 | | 1.75 | | 6 | | 0.00 | | 0 | | 0.00 | | 0 | | 0.17 | | 1 | | 0.00 | | 0 | | 4 | | | 1 | | 5 | |  |  |  |  |
| P20962 | Parathymosin | PTMS | 22.55 | 2 | | 11.5 | | 4.16 | | 0.59 | 3 | | 1.25 | | 5 | | 0.92 | | 4 | | 2.10 | | 5 | | 1.86 | | 1 | | 0.00 | | 0 | | 0.00 | | 0 | | 0.00 | | 0 | | 4 | | | 1 | | 5 | |  |  |  |  |
| P53634 | Dipeptidyl peptidase 1 | CTSC | 3.89 | 2 | | 51.8 | | 6.99 | | 0.54 | 3 | | 0.54 | | 3 | | 0.63 | | 2 | | 0.63 | | 3 | | 0.00 | | 0 | | 0.00 | | 0 | | 0.00 | | 0 | | 0.15 | | 1 | | 4 | | | 1 | | 5 | |  |  |  |  |
| Q02809 | Procollagen-lysine,2-oxoglutarate 5-dioxygenase 1 | PLOD1 | 15.96 | 8 | | 83.5 | | 6.95 | | 1.06 | 3 | | 2.41 | | 2 | | 0.19 | | 1 | | 0.83 | | 2 | | 0.00 | | 0 | | 0.00 | | 0 | | 0.00 | | 0 | | 0.61 | | 3 | | 4 | | | 1 | | 5 | |  |  |  |  |
| O75533 | Splicing factor 3B subunit 1 | SF3B1 | 3.83 | 2 | | 145.7 | | 7.09 | | 0.57 | 3 | | 0.27 | | 2 | | 0.16 | | 1 | | 0.16 | | 1 | | 0.14 | | 1 | | 0.00 | | 0 | | 0.00 | | 0 | | 0.00 | | 0 | | 4 | | | 1 | | 5 | |  |  |  |  |
| A0A0U1RRM4 | Polypyrimidine tract-binding protein 1 | PTBP1 | 19.05 | 5 | | 62.4 | | 9.10 | | 0.47 | 2 | | 2.61 | | 3 | | 1.48 | | 3 | | 1.91 | | 4 | | 0.14 | | 1 | | 0.00 | | 0 | | 0.00 | | 0 | | 0.00 | | 0 | | 4 | | | 1 | | 5 | |  |  |  |  |
| P55786 | Puromycin-sensitive aminopeptidase | NPEPPS | 7.40 | 4 | | 103.2 | | 5.72 | | 0.39 | 2 | | 0.26 | | 1 | | 0.63 | | 2 | | 0.32 | | 2 | | 0.00 | | 0 | | 0.00 | | 0 | | 0.17 | | 1 | | 0.00 | | 0 | | 4 | | | 1 | | 5 | |  |  |  |  |
| P32241 | Vasoactive intestinal polypeptide receptor 1 | VIPR1 | 2.63 | 1 | | 51.5 | | 8.18 | | 0.32 | 2 | | 0.41 | | 2 | | 0.60 | | 2 | | 0.21 | | 1 | | 0.14 | | 1 | | 0.00 | | 0 | | 0.00 | | 0 | | 0.00 | | 0 | | 4 | | | 1 | | 5 | |  |  |  |  |
| A0A3B3ITK7 | Phosphoglucomutase-1 | PGM1 | 4.28 | 1 | | 63.9 | | 6.76 | | 0.47 | 2 | | 0.27 | | 2 | | 0.45 | | 1 | | 0.16 | | 1 | | 0.00 | | 0 | | 0.00 | | 0 | | 0.00 | | 0 | | 0.15 | | 1 | | 4 | | | 1 | | 5 | |  |  |  |  |
| Q01105 | Protein SET | SET | 23.10 | 4 | | 33.5 | | 4.32 | | 1.75 | 2 | | 2.30 | | 4 | | 0.44 | | 1 | | 2.24 | | 4 | | 0.14 | | 1 | | 0.00 | | 0 | | 0.00 | | 0 | | 0.00 | | 0 | | 4 | | | 1 | | 5 | |  |  |  |  |
| E9PMD7 | Serine/threonine-protein phosphatase (Fragment) | PPP1CA | 21.34 | 2 | | 28.9 | | 4.87 | | 0.32 | 2 | | 0.95 | | 4 | | 0.34 | | 2 | | 0.17 | | 1 | | 0.00 | | 0 | | 0.16 | | 1 | | 0.00 | | 0 | | 0.00 | | 0 | | 4 | | | 1 | | 5 | |  |  |  |  |
| E9PPV7 | Disks large homolog 2 | DLG2 | 30.28 | 2 | | 16.3 | | 9.55 | | 0.32 | 2 | | 0.43 | | 3 | | 0.16 | | 1 | | 0.21 | | 1 | | 0.00 | | 0 | | 0.32 | | 1 | | 0.00 | | 0 | | 0.00 | | 0 | | 4 | | | 1 | | 5 | |  |  |  |  |
| P50454 | Serpin H1 | SERPINH1 | 32.54 | 8 | | 46.4 | | 8.69 | | 0.39 | 1 | | 3.08 | | 2 | | 2.27 | | 6 | | 4.56 | | 3 | | 0.00 | | 0 | | 0.00 | | 0 | | 0.00 | | 0 | | 0.45 | | 2 | | 4 | | | 1 | | 5 | |  |  |  |  |
| Q96PJ5 | Fc receptor-like protein 4 | FCRL4 | 7.57 | 2 | | 57.2 | | 6.52 | | 0.13 | 1 | | 0.14 | | 1 | | 0.60 | | 2 | | 0.42 | | 1 | | 0.14 | | 1 | | 0.00 | | 0 | | 0.00 | | 0 | | 0.00 | | 0 | | 4 | | | 1 | | 5 | |  |  |  |  |
| Q15691 | Microtubule-associated protein RP/EB family member 1 | MAPRE1 | 18.66 | 3 | | 30.0 | | 5.14 | | 0.13 | 1 | | 0.55 | | 2 | | 0.45 | | 1 | | 0.63 | | 2 | | 0.16 | | 1 | | 0.00 | | 0 | | 0.00 | | 0 | | 0.00 | | 0 | | 4 | | | 1 | | 5 | |  |  |  |  |
| D6RBZ0 | Heterogeneous nuclear ribonucleoprotein A/B | HNRNPAB | 10.40 | 3 | | 35.7 | | 6.95 | | 0.15 | 1 | | 0.27 | | 1 | | 0.22 | | 1 | | 0.53 | | 3 | | 0.85 | | 3 | | 0.00 | | 0 | | 0.00 | | 0 | | 0.00 | | 0 | | 4 | | | 1 | | 5 | |  |  |  |  |
| Q01469 | Fatty acid-binding protein 5 | FABP5 | 6.67 | 1 | | 15.2 | | 7.01 | | 0.13 | 1 | | 0.62 | | 3 | | 0.16 | | 1 | | 1.17 | | 5 | | 0.00 | | 0 | | 0.24 | | 1 | | 0.00 | | 0 | | 0.00 | | 0 | | 4 | | | 1 | | 5 | |  |  |  |  |
| Q9UI42 | Carboxypeptidase A4 | CPA4 | 61.28 | 16 | | 47.3 | | 6.70 | | 28.08 | 6 | | 19.04 | | 6 | | 36.95 | | 6 | | 25.24 | | 6 | | 0.00 | | 0 | | 0.00 | | 0 | | 0.00 | | 0 | | 0.00 | | 0 | | 4 | | | 0 | | 4 | |  |  |  |  |
| O00391 | Sulfhydryl oxidase 1 | QSOX1 | 19.54 | 11 | | 82.5 | | 8.92 | | 23.39 | 6 | | 14.13 | | 6 | | 20.50 | | 6 | | 10.72 | | 5 | | 0.00 | | 0 | | 0.00 | | 0 | | 0.00 | | 0 | | 0.00 | | 0 | | 4 | | | 0 | | 4 | |  |  |  |  |
| Q16270 | Insulin-like growth factor-binding protein 7 | IGFBP7 | 15.60 | 6 | | 29.1 | | 7.90 | | 17.94 | 6 | | 13.95 | | 6 | | 13.87 | | 6 | | 14.33 | | 6 | | 0.00 | | 0 | | 0.00 | | 0 | | 0.00 | | 0 | | 0.00 | | 0 | | 4 | | | 0 | | 4 | |  |  |  |  |
| Q12841 | Follistatin-related protein 1 | FSTL1 | 16.88 | 5 | | 35.0 | | 5.52 | | 10.60 | 6 | | 6.26 | | 6 | | 6.85 | | 6 | | 5.17 | | 6 | | 0.00 | | 0 | | 0.00 | | 0 | | 0.00 | | 0 | | 0.00 | | 0 | | 4 | | | 0 | | 4 | |  |  |  |  |
| O94985 | Calsyntenin-1 | CLSTN1 | 12.23 | 10 | | 109.7 | | 4.91 | | 9.66 | 6 | | 7.43 | | 6 | | 4.57 | | 6 | | 3.08 | | 6 | | 0.00 | | 0 | | 0.00 | | 0 | | 0.00 | | 0 | | 0.00 | | 0 | | 4 | | | 0 | | 4 | |  |  |  |  |
| P40926 | Malate dehydrogenase, mitochondrial | MDH2 | 28.70 | 7 | | 35.5 | | 8.68 | | 9.32 | 6 | | 8.81 | | 6 | | 4.09 | | 5 | | 5.72 | | 6 | | 0.00 | | 0 | | 0.00 | | 0 | | 0.00 | | 0 | | 0.00 | | 0 | | 4 | | | 0 | | 4 | |  |  |  |  |
| P08476 | Inhibin beta A chain | INHBA | 20.89 | 7 | | 47.4 | | 8.03 | | 3.52 | 6 | | 3.76 | | 6 | | 4.04 | | 6 | | 2.80 | | 6 | | 0.00 | | 0 | | 0.00 | | 0 | | 0.00 | | 0 | | 0.00 | | 0 | | 4 | | | 0 | | 4 | |  |  |  |  |
| P08253 | 72 kDa type IV collagenase | MMP2 | 23.33 | 11 | | 73.8 | | 5.47 | | 5.67 | 6 | | 3.54 | | 4 | | 3.33 | | 5 | | 2.79 | | 5 | | 0.00 | | 0 | | 0.00 | | 0 | | 0.00 | | 0 | | 0.00 | | 0 | | 4 | | | 0 | | 4 | |  |  |  |  |
| A0A5K1VW95 | Malate dehydrogenase | MDH1 | 20.40 | 5 | | 38.6 | | 7.30 | | 2.24 | 6 | | 2.46 | | 6 | | 2.52 | | 6 | | 2.40 | | 6 | | 0.00 | | 0 | | 0.00 | | 0 | | 0.00 | | 0 | | 0.00 | | 0 | | 4 | | | 0 | | 4 | |  |  |  |  |
| P03956 | Interstitial collagenase | MMP1 | 49.25 | 20 | | 54.0 | | 6.96 | | 37.98 | 6 | | 30.22 | | 6 | | 2.48 | | 3 | | 7.71 | | 4 | | 0.00 | | 0 | | 0.00 | | 0 | | 0.00 | | 0 | | 0.00 | | 0 | | 4 | | | 0 | | 4 | |  |  |  |  |
| P01034 | Cystatin-C | CST3 | 19.18 | 3 | | 15.8 | | 8.75 | | 2.54 | 6 | | 2.16 | | 6 | | 2.14 | | 6 | | 1.34 | | 5 | | 0.00 | | 0 | | 0.00 | | 0 | | 0.00 | | 0 | | 0.00 | | 0 | | 4 | | | 0 | | 4 | |  |  |  |  |
| P61604 | 10 kDa heat shock protein, mitochondrial | HSPE1 | 51.96 | 5 | | 10.9 | | 8.92 | | 3.28 | 6 | | 3.54 | | 6 | | 2.10 | | 4 | | 4.25 | | 5 | | 0.00 | | 0 | | 0.00 | | 0 | | 0.00 | | 0 | | 0.00 | | 0 | | 4 | | | 0 | | 4 | |  |  |  |  |
| P02647 | Apolipoprotein A-I | APOA1 | 5.99 | 1 | | 30.8 | | 5.76 | | 3.12 | 6 | | 3.30 | | 6 | | 2.05 | | 5 | | 4.04 | | 6 | | 0.00 | | 0 | | 0.00 | | 0 | | 0.00 | | 0 | | 0.00 | | 0 | | 4 | | | 0 | | 4 | |  |  |  |  |
| P27797 | Calreticulin | CALR | 40.77 | 11 | | 48.1 | | 4.44 | | 2.77 | 6 | | 8.66 | | 4 | | 1.50 | | 2 | | 8.71 | | 4 | | 0.00 | | 0 | | 0.00 | | 0 | | 0.00 | | 0 | | 0.00 | | 0 | | 4 | | | 0 | | 4 | |  |  |  |  |
| A0A0C4DGW9 | Serpin I2 | SERPINI2 | 3.37 | 1 | | 47.4 | | 5.22 | | 4.21 | 6 | | 2.18 | | 5 | | 0.60 | | 2 | | 0.33 | | 1 | | 0.00 | | 0 | | 0.00 | | 0 | | 0.00 | | 0 | | 0.00 | | 0 | | 4 | | | 0 | | 4 | |  |  |  |  |
| P10451 | Osteopontin | SPP1 | 36.62 | 8 | | 35.4 | | 4.58 | | 5.55 | 6 | | 5.14 | | 4 | | 0.56 | | 2 | | 1.32 | | 2 | | 0.00 | | 0 | | 0.00 | | 0 | | 0.00 | | 0 | | 0.00 | | 0 | | 4 | | | 0 | | 4 | |  |  |  |  |
| P55285 | Cadherin-6 | CDH6 | 15.32 | 10 | | 88.3 | | 4.93 | | 11.24 | 6 | | 8.47 | | 6 | | 0.22 | | 1 | | 0.16 | | 1 | | 0.00 | | 0 | | 0.00 | | 0 | | 0.00 | | 0 | | 0.00 | | 0 | | 4 | | | 0 | | 4 | |  |  |  |  |
| P00505 | Aspartate aminotransferase, mitochondrial | GOT2 | 19.07 | 6 | | 47.5 | | 9.01 | | 2.35 | 6 | | 2.83 | | 6 | | 0.16 | | 1 | | 0.62 | | 2 | | 0.00 | | 0 | | 0.00 | | 0 | | 0.00 | | 0 | | 0.00 | | 0 | | 4 | | | 0 | | 4 | |  |  |  |  |
| P01024 | Complement C3 | C3 | 4.21 | 5 | | 187.0 | | 6.40 | | 2.52 | 5 | | 1.58 | | 6 | | 5.93 | | 6 | | 4.41 | | 6 | | 0.00 | | 0 | | 0.00 | | 0 | | 0.00 | | 0 | | 0.00 | | 0 | | 4 | | | 0 | | 4 | |  |  |  |  |
| P68036 | Ubiquitin-conjugating enzyme E2 L3 | UBE2L3 | 35.71 | 4 | | 17.9 | | 8.51 | | 2.33 | 5 | | 3.28 | | 5 | | 2.77 | | 5 | | 2.47 | | 6 | | 0.00 | | 0 | | 0.00 | | 0 | | 0.00 | | 0 | | 0.00 | | 0 | | 4 | | | 0 | | 4 | |  |  |  |  |
| O76061 | Stanniocalcin-2 | STC2 | 7.95 | 2 | | 33.2 | | 7.30 | | 2.08 | 5 | | 2.10 | | 5 | | 1.54 | | 4 | | 1.77 | | 4 | | 0.00 | | 0 | | 0.00 | | 0 | | 0.00 | | 0 | | 0.00 | | 0 | | 4 | | | 0 | | 4 | |  |  |  |  |
| P01033 | Metalloproteinase inhibitor 1 | TIMP1 | 10.14 | 2 | | 23.2 | | 8.10 | | 2.47 | 5 | | 1.26 | | 3 | | 1.45 | | 5 | | 1.18 | | 4 | | 0.00 | | 0 | | 0.00 | | 0 | | 0.00 | | 0 | | 0.00 | | 0 | | 4 | | | 0 | | 4 | |  |  |  |  |
| P07954 | Fumarate hydratase, mitochondrial | FH | 8.63 | 2 | | 54.6 | | 8.76 | | 1.52 | 5 | | 1.64 | | 5 | | 1.00 | | 3 | | 0.94 | | 3 | | 0.00 | | 0 | | 0.00 | | 0 | | 0.00 | | 0 | | 0.00 | | 0 | | 4 | | | 0 | | 4 | |  |  |  |  |
| Q08257 | Quinone oxidoreductase | CRYZ | 10.94 | 3 | | 35.2 | | 8.44 | | 1.95 | 5 | | 0.81 | | 3 | | 0.82 | | 3 | | 0.65 | | 3 | | 0.00 | | 0 | | 0.00 | | 0 | | 0.00 | | 0 | | 0.00 | | 0 | | 4 | | | 0 | | 4 | |  |  |  |  |
| J3KMX3 | Alpha-fetoprotein | AFP | 9.16 | 4 | | 70.4 | | 5.55 | | 2.97 | 4 | | 2.04 | | 6 | | 4.63 | | 6 | | 4.58 | | 6 | | 0.00 | | 0 | | 0.00 | | 0 | | 0.00 | | 0 | | 0.00 | | 0 | | 4 | | | 0 | | 4 | |  |  |  |  |
| P00749 | Urokinase-type plasminogen activator | PLAU | 18.10 | 6 | | 48.5 | | 8.41 | | 5.79 | 4 | | 5.31 | | 5 | | 4.25 | | 6 | | 1.68 | | 2 | | 0.00 | | 0 | | 0.00 | | 0 | | 0.00 | | 0 | | 0.00 | | 0 | | 4 | | | 0 | | 4 | |  |  |  |  |
| O60888 | Protein CutA | CUTA | 22.91 | 2 | | 19.1 | | 5.50 | | 2.05 | 4 | | 1.93 | | 5 | | 2.99 | | 6 | | 0.78 | | 3 | | 0.00 | | 0 | | 0.00 | | 0 | | 0.00 | | 0 | | 0.00 | | 0 | | 4 | | | 0 | | 4 | |  |  |  |  |
| P30086 | Phosphatidylethanolamine-binding protein 1 | PEBP1 | 57.75 | 7 | | 21.0 | | 7.53 | | 2.53 | 4 | | 2.98 | | 6 | | 2.61 | | 5 | | 2.48 | | 6 | | 0.00 | | 0 | | 0.00 | | 0 | | 0.00 | | 0 | | 0.00 | | 0 | | 4 | | | 0 | | 4 | |  |  |  |  |
| P11216 | Glycogen phosphorylase, brain form | PYGB | 7.35 | 3 | | 96.6 | | 6.86 | | 1.89 | 4 | | 1.36 | | 4 | | 2.40 | | 4 | | 0.17 | | 1 | | 0.00 | | 0 | | 0.00 | | 0 | | 0.00 | | 0 | | 0.00 | | 0 | | 4 | | | 0 | | 4 | |  |  |  |  |
| Q04760 | Lactoylglutathione lyase | GLO1 | 29.35 | 5 | | 20.8 | | 5.31 | | 3.11 | 4 | | 3.96 | | 5 | | 2.24 | | 4 | | 1.98 | | 4 | | 0.00 | | 0 | | 0.00 | | 0 | | 0.00 | | 0 | | 0.00 | | 0 | | 4 | | | 0 | | 4 | |  |  |  |  |
| Q8NC51 | Plasminogen activator inhibitor 1 RNA-binding protein | SERBP1 | 13.48 | 4 | | 44.9 | | 8.65 | | 1.11 | 4 | | 2.51 | | 6 | | 2.15 | | 5 | | 2.45 | | 6 | | 0.00 | | 0 | | 0.00 | | 0 | | 0.00 | | 0 | | 0.00 | | 0 | | 4 | | | 0 | | 4 | |  |  |  |  |
| P00441 | Superoxide dismutase [Cu-Zn] | SOD1 | 16.88 | 2 | | 15.9 | | 6.13 | | 2.52 | 4 | | 3.84 | | 6 | | 1.73 | | 2 | | 2.45 | | 5 | | 0.00 | | 0 | | 0.00 | | 0 | | 0.00 | | 0 | | 0.00 | | 0 | | 4 | | | 0 | | 4 | |  |  |  |  |
| P61088 | Ubiquitin-conjugating enzyme E2 N | UBE2N | 23.68 | 3 | | 17.1 | | 6.57 | | 1.82 | 4 | | 1.63 | | 4 | | 1.61 | | 3 | | 0.66 | | 2 | | 0.00 | | 0 | | 0.00 | | 0 | | 0.00 | | 0 | | 0.00 | | 0 | | 4 | | | 0 | | 4 | |  |  |  |  |
| P22692 | Insulin-like growth factor-binding protein 4 | IGFBP4 | 3.10 | 1 | | 27.9 | | 7.15 | | 1.00 | 4 | | 1.19 | | 5 | | 1.04 | | 4 | | 1.32 | | 5 | | 0.00 | | 0 | | 0.00 | | 0 | | 0.00 | | 0 | | 0.00 | | 0 | | 4 | | | 0 | | 4 | |  |  |  |  |
| H7BZJ3 | Protein disulfide-isomerase A3 (Fragment) | PDIA3 | 38.21 | 1 | | 13.5 | | 7.30 | | 3.06 | 4 | | 3.86 | | 4 | | 0.91 | | 3 | | 2.84 | | 4 | | 0.00 | | 0 | | 0.00 | | 0 | | 0.00 | | 0 | | 0.00 | | 0 | | 4 | | | 0 | | 4 | |  |  |  |  |
| P24387 | Corticotropin-releasing factor-binding protein | CRHBP | 4.66 | 2 | | 36.1 | | 6.52 | | 1.47 | 4 | | 1.14 | | 6 | | 0.72 | | 3 | | 0.63 | | 3 | | 0.00 | | 0 | | 0.00 | | 0 | | 0.00 | | 0 | | 0.00 | | 0 | | 4 | | | 0 | | 4 | |  |  |  |  |
| E7EX17 | Eukaryotic translation initiation factor 4B | EIF4B | 4.55 | 2 | | 69.7 | | 5.67 | | 0.57 | 4 | | 0.54 | | 3 | | 0.63 | | 2 | | 0.16 | | 1 | | 0.00 | | 0 | | 0.00 | | 0 | | 0.00 | | 0 | | 0.00 | | 0 | | 4 | | | 0 | | 4 | |  |  |  |  |
| B5MC82 | D-dopachrome decarboxylase | DDT | 7.23 | 1 | | 17.7 | | 8.69 | | 1.28 | 4 | | 1.08 | | 4 | | 0.53 | | 2 | | 0.59 | | 3 | | 0.00 | | 0 | | 0.00 | | 0 | | 0.00 | | 0 | | 0.00 | | 0 | | 4 | | | 0 | | 4 | |  |  |  |  |
| P98179 | RNA-binding protein 3 | RBM3 | 31.85 | 2 | | 17.2 | | 8.91 | | 1.76 | 4 | | 1.50 | | 4 | | 0.47 | | 2 | | 1.09 | | 2 | | 0.00 | | 0 | | 0.00 | | 0 | | 0.00 | | 0 | | 0.00 | | 0 | | 4 | | | 0 | | 4 | |  |  |  |  |
| E5RJD8 | Tubulin-specific chaperone A | TBCA | 24.37 | 3 | | 14.3 | | 5.12 | | 1.11 | 4 | | 1.76 | | 4 | | 0.47 | | 2 | | 0.47 | | 2 | | 0.00 | | 0 | | 0.00 | | 0 | | 0.00 | | 0 | | 0.00 | | 0 | | 4 | | | 0 | | 4 | |  |  |  |  |
| P18065 | Insulin-like growth factor-binding protein 2 | IGFBP2 | 2.77 | 1 | | 34.8 | | 7.50 | | 0.96 | 4 | | 1.46 | | 6 | | 0.41 | | 2 | | 1.22 | | 4 | | 0.00 | | 0 | | 0.00 | | 0 | | 0.00 | | 0 | | 0.00 | | 0 | | 4 | | | 0 | | 4 | |  |  |  |  |
| P61758 | Prefoldin subunit 3 | VBP1 | 9.64 | 1 | | 22.6 | | 7.11 | | 0.57 | 4 | | 0.68 | | 3 | | 0.16 | | 1 | | 0.16 | | 1 | | 0.00 | | 0 | | 0.00 | | 0 | | 0.00 | | 0 | | 0.00 | | 0 | | 4 | | | 0 | | 4 | |  |  |  |  |
| P20700 | Lamin-B1 | LMNB1 | 7.17 | 3 | | 66.4 | | 5.16 | | 1.14 | 3 | | 0.82 | | 3 | | 2.05 | | 6 | | 1.17 | | 5 | | 0.00 | | 0 | | 0.00 | | 0 | | 0.00 | | 0 | | 0.00 | | 0 | | 4 | | | 0 | | 4 | |  |  |  |  |
| Q4G0S7 | Coiled-coil domain-containing protein 152 | CCDC152 | 3.94 | 1 | | 30.0 | | 9.06 | | 1.39 | 3 | | 0.59 | | 4 | | 1.49 | | 3 | | 0.33 | | 2 | | 0.00 | | 0 | | 0.00 | | 0 | | 0.00 | | 0 | | 0.00 | | 0 | | 4 | | | 0 | | 4 | |  |  |  |  |
| A0A0A0MQX1 | Unconventional myosin-X | MYO10 | 1.88 | 2 | | 238.4 | | 6.14 | | 0.69 | 3 | | 0.57 | | 4 | | 1.03 | | 4 | | 1.11 | | 5 | | 0.00 | | 0 | | 0.00 | | 0 | | 0.00 | | 0 | | 0.00 | | 0 | | 4 | | | 0 | | 4 | |  |  |  |  |
| Q5T9B7 | Adenylate kinase isoenzyme 1 | AK1 | 6.67 | 1 | | 23.4 | | 8.60 | | 0.76 | 3 | | 0.96 | | 3 | | 0.95 | | 4 | | 0.58 | | 3 | | 0.00 | | 0 | | 0.00 | | 0 | | 0.00 | | 0 | | 0.00 | | 0 | | 4 | | | 0 | | 4 | |  |  |  |  |
| F5H2P7 | Zinc finger protein 26 (Fragment) | ZNF26 | 5.88 | 1 | | 17.7 | | 5.29 | | 0.54 | 3 | | 0.60 | | 4 | | 0.76 | | 4 | | 1.17 | | 5 | | 0.00 | | 0 | | 0.00 | | 0 | | 0.00 | | 0 | | 0.00 | | 0 | | 4 | | | 0 | | 4 | |  |  |  |  |
| Q15843 | NEDD8 | NEDD8 | 34.57 | 2 | | 9.1 | | 8.43 | | 0.80 | 3 | | 2.44 | | 4 | | 0.69 | | 3 | | 1.59 | | 5 | | 0.00 | | 0 | | 0.00 | | 0 | | 0.00 | | 0 | | 0.00 | | 0 | | 4 | | | 0 | | 4 | |  |  |  |  |
| P04080 | Cystatin-B | CSTB | 33.67 | 2 | | 11.1 | | 7.56 | | 0.67 | 3 | | 1.86 | | 6 | | 0.63 | | 2 | | 0.64 | | 4 | | 0.00 | | 0 | | 0.00 | | 0 | | 0.00 | | 0 | | 0.00 | | 0 | | 4 | | | 0 | | 4 | |  |  |  |  |
| A0A0B4J2C3 | Translationally-controlled tumor protein | TPT1 | 36.04 | 3 | | 22.6 | | 5.24 | | 1.07 | 3 | | 1.15 | | 4 | | 0.60 | | 3 | | 0.80 | | 3 | | 0.00 | | 0 | | 0.00 | | 0 | | 0.00 | | 0 | | 0.00 | | 0 | | 4 | | | 0 | | 4 | |  |  |  |  |
| A0A024R6I7 | Alpha-1-antitrypsin | SERPINA1 | 1.91 | 1 | | 46.7 | | 5.59 | | 0.85 | 3 | | 0.59 | | 2 | | 0.53 | | 2 | | 0.63 | | 2 | | 0.00 | | 0 | | 0.00 | | 0 | | 0.00 | | 0 | | 0.00 | | 0 | | 4 | | | 0 | | 4 | |  |  |  |  |
| I3L0A0 | HCG2044781 | TMEM189-UBE2V1 | 7.30 | 3 | | 42.2 | | 6.71 | | 0.67 | 3 | | 0.98 | | 3 | | 0.47 | | 2 | | 0.16 | | 1 | | 0.00 | | 0 | | 0.00 | | 0 | | 0.00 | | 0 | | 0.00 | | 0 | | 4 | | | 0 | | 4 | |  |  |  |  |
| Q99497 | Parkinson disease protein 7 | PARK7 | 16.93 | 3 | | 19.9 | | 6.79 | | 0.68 | 3 | | 1.55 | | 5 | | 0.32 | | 1 | | 2.17 | | 6 | | 0.00 | | 0 | | 0.00 | | 0 | | 0.00 | | 0 | | 0.00 | | 0 | | 4 | | | 0 | | 4 | |  |  |  |  |
| P58546 | Myotrophin | MTPN | 14.41 | 1 | | 12.9 | | 5.52 | | 0.54 | 3 | | 0.41 | | 2 | | 0.16 | | 1 | | 0.16 | | 1 | | 0.00 | | 0 | | 0.00 | | 0 | | 0.00 | | 0 | | 0.00 | | 0 | | 4 | | | 0 | | 4 | |  |  |  |  |
| E9PGZ1 | Caldesmon | CALD1 | 19.59 | 9 | | 61.7 | | 6.11 | | 0.64 | 2 | | 3.60 | | 6 | | 2.86 | | 5 | | 4.22 | | 6 | | 0.00 | | 0 | | 0.00 | | 0 | | 0.00 | | 0 | | 0.00 | | 0 | | 4 | | | 0 | | 4 | |  |  |  |  |
| P51884 | Lumican | LUM | 8.28 | 2 | | 38.4 | | 6.61 | | 1.26 | 2 | | 0.48 | | 2 | | 2.51 | | 4 | | 2.84 | | 4 | | 0.00 | | 0 | | 0.00 | | 0 | | 0.00 | | 0 | | 0.00 | | 0 | | 4 | | | 0 | | 4 | |  |  |  |  |
| P30085 | UMP-CMP kinase | CMPK1 | 14.80 | 3 | | 22.2 | | 5.57 | | 0.41 | 2 | | 1.63 | | 4 | | 1.82 | | 4 | | 0.16 | | 1 | | 0.00 | | 0 | | 0.00 | | 0 | | 0.00 | | 0 | | 0.00 | | 0 | | 4 | | | 0 | | 4 | |  |  |  |  |
| P62942 | Peptidyl-prolyl cis-trans isomerase FKBP1A | FKBP1A | 12.96 | 1 | | 11.9 | | 8.16 | | 0.51 | 2 | | 1.57 | | 6 | | 1.38 | | 4 | | 1.49 | | 5 | | 0.00 | | 0 | | 0.00 | | 0 | | 0.00 | | 0 | | 0.00 | | 0 | | 4 | | | 0 | | 4 | |  |  |  |  |
| P01008 | Antithrombin-III | SERPINC1 | 11.85 | 5 | | 52.6 | | 6.71 | | 1.70 | 2 | | 0.64 | | 2 | | 1.20 | | 4 | | 2.24 | | 5 | | 0.00 | | 0 | | 0.00 | | 0 | | 0.00 | | 0 | | 0.00 | | 0 | | 4 | | | 0 | | 4 | |  |  |  |  |
| P16035 | Metalloproteinase inhibitor 2 | TIMP2 | 13.64 | 3 | | 24.4 | | 7.49 | | 1.16 | 2 | | 0.33 | | 1 | | 1.13 | | 3 | | 0.48 | | 2 | | 0.00 | | 0 | | 0.00 | | 0 | | 0.00 | | 0 | | 0.00 | | 0 | | 4 | | | 0 | | 4 | |  |  |  |  |
| B4DUT8 | Calponin | CNN2 | 8.48 | 2 | | 35.9 | | 7.30 | | 0.39 | 2 | | 1.92 | | 5 | | 1.04 | | 4 | | 1.18 | | 4 | | 0.00 | | 0 | | 0.00 | | 0 | | 0.00 | | 0 | | 0.00 | | 0 | | 4 | | | 0 | | 4 | |  |  |  |  |
| Q08629 | Testican-1 | SPOCK1 | 7.52 | 3 | | 49.1 | | 6.10 | | 0.26 | 2 | | 0.43 | | 3 | | 1.01 | | 3 | | 0.52 | | 2 | | 0.00 | | 0 | | 0.00 | | 0 | | 0.00 | | 0 | | 0.00 | | 0 | | 4 | | | 0 | | 4 | |  |  |  |  |
| P49321 | Nuclear autoantigenic sperm protein | NASP | 2.92 | 1 | | 85.2 | | 4.30 | | 0.95 | 2 | | 1.09 | | 3 | | 0.95 | | 2 | | 0.42 | | 1 | | 0.00 | | 0 | | 0.00 | | 0 | | 0.00 | | 0 | | 0.00 | | 0 | | 4 | | | 0 | | 4 | |  |  |  |  |
| P29279 | CCN family member 2 | CCN2 | 10.03 | 3 | | 38.1 | | 8.00 | | 0.77 | 2 | | 0.89 | | 4 | | 0.78 | | 2 | | 1.09 | | 2 | | 0.00 | | 0 | | 0.00 | | 0 | | 0.00 | | 0 | | 0.00 | | 0 | | 4 | | | 0 | | 4 | |  |  |  |  |
| Q99598 | Translin-associated protein X | TSNAX | 6.55 | 1 | | 33.1 | | 6.55 | | 0.43 | 2 | | 0.54 | | 2 | | 0.70 | | 2 | | 0.16 | | 1 | | 0.00 | | 0 | | 0.00 | | 0 | | 0.00 | | 0 | | 0.00 | | 0 | | 4 | | | 0 | | 4 | |  |  |  |  |
| P21810 | Biglycan | BGN | 2.99 | 1 | | 41.6 | | 7.52 | | 0.77 | 2 | | 0.59 | | 4 | | 0.47 | | 2 | | 0.16 | | 1 | | 0.00 | | 0 | | 0.00 | | 0 | | 0.00 | | 0 | | 0.00 | | 0 | | 4 | | | 0 | | 4 | |  |  |  |  |
| Q9Y3B8 | Oligoribonuclease, mitochondrial | REXO2 | 7.59 | 1 | | 26.8 | | 6.87 | | 0.26 | 2 | | 0.55 | | 2 | | 0.38 | | 2 | | 0.47 | | 2 | | 0.00 | | 0 | | 0.00 | | 0 | | 0.00 | | 0 | | 0.00 | | 0 | | 4 | | | 0 | | 4 | |  |  |  |  |
| Q15181 | Inorganic pyrophosphatase | PPA1 | 10.38 | 2 | | 32.6 | | 5.86 | | 0.63 | 2 | | 1.08 | | 4 | | 0.31 | | 2 | | 1.33 | | 5 | | 0.00 | | 0 | | 0.00 | | 0 | | 0.00 | | 0 | | 0.00 | | 0 | | 4 | | | 0 | | 4 | |  |  |  |  |
| Q9C0B2 | Cilia- and flagella-associated protein 74 | CFAP74 | 0.63 | 1 | | 178.5 | | 6.42 | | 0.41 | 2 | | 0.87 | | 4 | | 0.22 | | 1 | | 0.16 | | 1 | | 0.00 | | 0 | | 0.00 | | 0 | | 0.00 | | 0 | | 0.00 | | 0 | | 4 | | | 0 | | 4 | |  |  |  |  |
| Q6UWU2 | Beta-galactosidase-1-like protein | GLB1L | 1.38 | 1 | | 74.1 | | 8.92 | | 0.26 | 2 | | 0.46 | | 3 | | 0.22 | | 1 | | 0.42 | | 1 | | 0.00 | | 0 | | 0.00 | | 0 | | 0.00 | | 0 | | 0.00 | | 0 | | 4 | | | 0 | | 4 | |  |  |  |  |
| P04053 | DNA nucleotidylexotransferase | DNTT | 2.16 | 1 | | 58.5 | | 8.43 | | 0.45 | 2 | | 0.13 | | 1 | | 0.19 | | 1 | | 0.37 | | 2 | | 0.00 | | 0 | | 0.00 | | 0 | | 0.00 | | 0 | | 0.00 | | 0 | | 4 | | | 0 | | 4 | |  |  |  |  |
| P30048 | Thioredoxin-dependent peroxide reductase, mitochondrial | PRDX3 | 5.47 | 1 | | 27.7 | | 7.78 | | 0.51 | 2 | | 1.24 | | 5 | | 0.19 | | 1 | | 0.17 | | 1 | | 0.00 | | 0 | | 0.00 | | 0 | | 0.00 | | 0 | | 0.00 | | 0 | | 4 | | | 0 | | 4 | |  |  |  |  |
| O15511 | Actin-related protein 2/3 complex subunit 5 | ARPC5 | 7.95 | 1 | | 16.3 | | 5.67 | | 0.63 | 2 | | 0.54 | | 2 | | 0.16 | | 1 | | 0.16 | | 1 | | 0.00 | | 0 | | 0.00 | | 0 | | 0.00 | | 0 | | 0.00 | | 0 | | 4 | | | 0 | | 4 | |  |  |  |  |
| P62140 | Serine/threonine-protein phosphatase PP1-beta catalytic subunit | PPP1CB | 9.79 | 1 | | 37.2 | | 6.19 | | 0.32 | 2 | | 1.11 | | 5 | | 0.16 | | 1 | | 0.17 | | 1 | | 0.00 | | 0 | | 0.00 | | 0 | | 0.00 | | 0 | | 0.00 | | 0 | | 4 | | | 0 | | 4 | |  |  |  |  |
| Q96HC4 | PDZ and LIM domain protein 5 | PDLIM5 | 4.36 | 2 | | 63.9 | | 8.21 | | 0.16 | 1 | | 0.70 | | 3 | | 1.55 | | 5 | | 0.96 | | 4 | | 0.00 | | 0 | | 0.00 | | 0 | | 0.00 | | 0 | | 0.00 | | 0 | | 4 | | | 0 | | 4 | |  |  |  |  |
| Q8IVI9 | Nostrin | NOSTRIN | 1.58 | 1 | | 57.6 | | 8.97 | | 0.13 | 1 | | 0.30 | | 2 | | 0.88 | | 4 | | 0.16 | | 1 | | 0.00 | | 0 | | 0.00 | | 0 | | 0.00 | | 0 | | 0.00 | | 0 | | 4 | | | 0 | | 4 | |  |  |  |  |
| B7ZKJ8 | ITIH4 protein | ITIH4 | 4.81 | 2 | | 103.8 | | 6.89 | | 0.16 | 1 | | 0.55 | | 3 | | 0.66 | | 1 | | 1.13 | | 4 | | 0.00 | | 0 | | 0.00 | | 0 | | 0.00 | | 0 | | 0.00 | | 0 | | 4 | | | 0 | | 4 | |  |  |  |  |
| Q8NEM0 | Microcephalin | MCPH1 | 1.44 | 1 | | 92.8 | | 8.25 | | 0.16 | 1 | | 0.14 | | 1 | | 0.63 | | 3 | | 0.78 | | 3 | | 0.00 | | 0 | | 0.00 | | 0 | | 0.00 | | 0 | | 0.00 | | 0 | | 4 | | | 0 | | 4 | |  |  |  |  |
| P63220 | 40S ribosomal protein S21 | RPS21 | 28.92 | 2 | | 9.1 | | 8.50 | | 0.15 | 1 | | 1.22 | | 4 | | 0.60 | | 2 | | 0.78 | | 3 | | 0.00 | | 0 | | 0.00 | | 0 | | 0.00 | | 0 | | 0.00 | | 0 | | 4 | | | 0 | | 4 | |  |  |  |  |
| A0A0A0MT84 | T cell receptor gamma joining P2 (Fragment) | TRGJP2 | 35.00 | 1 | | 2.2 | | 9.99 | | 0.13 | 1 | | 0.28 | | 1 | | 0.57 | | 3 | | 0.63 | | 2 | | 0.00 | | 0 | | 0.00 | | 0 | | 0.00 | | 0 | | 0.00 | | 0 | | 4 | | | 0 | | 4 | |  |  |  |  |
| Q15785 | Mitochondrial import receptor subunit TOM34 | TOMM34 | 2.91 | 1 | | 34.5 | | 8.98 | | 0.26 | 1 | | 0.73 | | 4 | | 0.38 | | 2 | | 0.21 | | 1 | | 0.00 | | 0 | | 0.00 | | 0 | | 0.00 | | 0 | | 0.00 | | 0 | | 4 | | | 0 | | 4 | |  |  |  |  |
| O75368 | SH3 domain-binding glutamic acid-rich-like protein | SH3BGRL | 11.40 | 1 | | 12.8 | | 5.25 | | 0.13 | 1 | | 0.14 | | 1 | | 0.32 | | 1 | | 0.47 | | 2 | | 0.00 | | 0 | | 0.00 | | 0 | | 0.00 | | 0 | | 0.00 | | 0 | | 4 | | | 0 | | 4 | |  |  |  |  |
| Q14847 | LIM and SH3 domain protein 1 | LASP1 | 17.62 | 5 | | 29.7 | | 7.05 | | 0.28 | 1 | | 0.55 | | 2 | | 0.31 | | 1 | | 0.94 | | 3 | | 0.00 | | 0 | | 0.00 | | 0 | | 0.00 | | 0 | | 0.00 | | 0 | | 4 | | | 0 | | 4 | |  |  |  |  |
| Q13425 | Beta-2-syntrophin | SNTB2 | 1.85 | 1 | | 57.9 | | 8.82 | | 0.57 | 1 | | 0.40 | | 2 | | 0.22 | | 1 | | 0.63 | | 2 | | 0.00 | | 0 | | 0.00 | | 0 | | 0.00 | | 0 | | 0.00 | | 0 | | 4 | | | 0 | | 4 | |  |  |  |  |
| Q96N76 | Urocanate hydratase | UROC1 | 3.85 | 1 | | 74.8 | | 6.79 | | 0.16 | 1 | | 0.41 | | 2 | | 0.22 | | 1 | | 0.36 | | 2 | | 0.00 | | 0 | | 0.00 | | 0 | | 0.00 | | 0 | | 0.00 | | 0 | | 4 | | | 0 | | 4 | |  |  |  |  |
| Q15084 | Protein disulfide-isomerase A6 | PDIA6 | 12.50 | 3 | | 48.1 | | 5.08 | | 0.13 | 1 | | 1.77 | | 4 | | 0.19 | | 1 | | 0.66 | | 2 | | 0.00 | | 0 | | 0.00 | | 0 | | 0.00 | | 0 | | 0.00 | | 0 | | 4 | | | 0 | | 4 | |  |  |  |  |
| Q12980 | GATOR complex protein NPRL3 | NPRL3 | 2.81 | 1 | | 63.6 | | 6.98 | | 0.28 | 1 | | 0.16 | | 1 | | 0.16 | | 1 | | 0.21 | | 1 | | 0.00 | | 0 | | 0.00 | | 0 | | 0.00 | | 0 | | 0.00 | | 0 | | 4 | | | 0 | | 4 | |  |  |  |  |
| G3XAI2 | Laminin subunit beta-1 | LAMB1 | 6.80 | 7 | | 200.3 | | 4.96 | | 4.05 | 4 | | 1.36 | | 4 | | 1.88 | | 4 | | 0.00 | | 0 | | 2.68 | | 3 | | 4.76 | | 2 | | 0.65 | | 3 | | 0.36 | | 2 | | 3 | | | 4 | | 7 | |  |  |  |  |
| P60842 | Eukaryotic initiation factor 4A-I | EIF4A1 | 47.29 | 8 | | 46.1 | | 5.48 | | 3.38 | 4 | | 2.99 | | 4 | | 1.11 | | 2 | | 0.00 | | 0 | | 7.53 | | 6 | | 9.47 | | 6 | | 10.36 | | 6 | | 7.91 | | 6 | | 3 | | | 4 | | 7 | |  |  |  |  |
| Q14240 | Eukaryotic initiation factor 4A-II | EIF4A2 | 24.82 | 1 | | 46.4 | | 5.48 | | 2.61 | 4 | | 2.58 | | 4 | | 1.11 | | 2 | | 0.00 | | 0 | | 5.37 | | 6 | | 5.59 | | 5 | | 6.11 | | 6 | | 5.03 | | 6 | | 3 | | | 4 | | 7 | |  |  |  |  |
| P46940 | Ras GTPase-activating-like protein IQGAP1 | IQGAP1 | 16.35 | 14 | | 189.1 | | 6.48 | | 1.66 | 4 | | 1.49 | | 4 | | 0.47 | | 1 | | 0.00 | | 0 | | 4.23 | | 3 | | 2.58 | | 3 | | 2.65 | | 4 | | 0.46 | | 2 | | 3 | | | 4 | | 7 | |  |  |  |  |
| P11047 | Laminin subunit gamma-1 | LAMC1 | 7.02 | 8 | | 177.5 | | 5.12 | | 1.98 | 4 | | 0.62 | | 3 | | 0.16 | | 1 | | 0.00 | | 0 | | 0.44 | | 2 | | 5.61 | | 6 | | 0.97 | | 4 | | 0.87 | | 3 | | 3 | | | 4 | | 7 | |  |  |  |  |
| O60506 | Heterogeneous nuclear ribonucleoprotein Q | SYNCRIP | 12.52 | 4 | | 69.6 | | 8.59 | | 0.54 | 3 | | 0.40 | | 2 | | 0.47 | | 2 | | 0.00 | | 0 | | 3.24 | | 3 | | 1.69 | | 4 | | 2.55 | | 6 | | 0.61 | | 2 | | 3 | | | 4 | | 7 | |  |  |  |  |
| A0A087WVQ6 | Clathrin heavy chain | CLTC | 33.41 | 40 | | 191.9 | | 5.69 | | 0.76 | 3 | | 0.00 | | 0 | | 0.44 | | 1 | | 0.16 | | 1 | | 29.92 | | 6 | | 15.74 | | 6 | | 35.22 | | 6 | | 23.61 | | 6 | | 3 | | | 4 | | 7 | |  |  |  |  |
| A0A0D9SF53 | ATP-dependent RNA helicase DDX3X | DDX3X | 13.92 | 7 | | 81.4 | | 8.07 | | 0.42 | 3 | | 0.16 | | 1 | | 0.34 | | 2 | | 0.00 | | 0 | | 4.93 | | 3 | | 0.89 | | 4 | | 0.91 | | 4 | | 0.49 | | 2 | | 3 | | | 4 | | 7 | |  |  |  |  |
| Q99436 | Proteasome subunit beta type-7 | PSMB7 | 21.66 | 4 | | 29.9 | | 7.68 | | 1.87 | 3 | | 1.45 | | 4 | | 0.00 | | 0 | | 3.57 | | 5 | | 0.46 | | 3 | | 1.13 | | 3 | | 2.81 | | 6 | | 4.59 | | 6 | | 3 | | | 4 | | 7 | |  |  |  |  |
| P12956 | X-ray repair cross-complementing protein 6 | XRCC6 | 37.60 | 18 | | 69.8 | | 6.64 | | 0.90 | 3 | | 0.68 | | 4 | | 0.00 | | 0 | | 0.21 | | 1 | | 10.85 | | 3 | | 5.33 | | 4 | | 9.92 | | 6 | | 6.94 | | 6 | | 3 | | | 4 | | 7 | |  |  |  |  |
| O43734 | Adapter protein CIKS | TRAF3IP2 | 2.09 | 1 | | 64.6 | | 6.76 | | 0.26 | 2 | | 1.26 | | 5 | | 0.69 | | 4 | | 0.00 | | 0 | | 2.70 | | 4 | | 1.32 | | 4 | | 2.50 | | 5 | | 2.84 | | 6 | | 3 | | | 4 | | 7 | |  |  |  |  |
| P62249 | 40S ribosomal protein S16 | RPS16 | 27.40 | 5 | | 16.4 | | 10.21 | | 0.32 | 2 | | 0.13 | | 1 | | 0.67 | | 2 | | 0.00 | | 0 | | 8.38 | | 5 | | 4.92 | | 5 | | 8.56 | | 6 | | 8.41 | | 6 | | 3 | | | 4 | | 7 | |  |  |  |  |
| Q9Y678 | Coatomer subunit gamma-1 | COPG1 | 5.03 | 2 | | 97.7 | | 5.47 | | 0.41 | 2 | | 0.40 | | 2 | | 0.60 | | 2 | | 0.00 | | 0 | | 2.12 | | 3 | | 0.48 | | 2 | | 2.43 | | 6 | | 1.55 | | 5 | | 3 | | | 4 | | 7 | |  |  |  |  |
| P23246 | Splicing factor, proline- and glutamine-rich | SFPQ | 21.07 | 10 | | 76.1 | | 9.44 | | 0.47 | 2 | | 1.37 | | 2 | | 0.00 | | 0 | | 1.27 | | 4 | | 5.54 | | 5 | | 1.92 | | 4 | | 5.30 | | 6 | | 1.81 | | 6 | | 3 | | | 4 | | 7 | |  |  |  |  |
| P25786 | Proteasome subunit alpha type-1 | PSMA1 | 52.09 | 12 | | 29.5 | | 6.61 | | 0.90 | 2 | | 1.08 | | 2 | | 0.00 | | 0 | | 0.62 | | 2 | | 0.46 | | 3 | | 0.95 | | 2 | | 3.59 | | 6 | | 4.90 | | 6 | | 3 | | | 4 | | 7 | |  |  |  |  |
| P25788 | Proteasome subunit alpha type-3 | PSMA3 | 17.65 | 4 | | 28.4 | | 5.33 | | 0.77 | 2 | | 0.43 | | 3 | | 0.00 | | 0 | | 0.47 | | 2 | | 0.61 | | 4 | | 1.54 | | 4 | | 1.85 | | 6 | | 2.09 | | 6 | | 3 | | | 4 | | 7 | |  |  |  |  |
| A0A0C4DGQ5 | Calpain small subunit 1 | CAPNS1 | 11.80 | 2 | | 33.8 | | 6.23 | | 0.59 | 2 | | 0.55 | | 3 | | 0.00 | | 0 | | 0.42 | | 2 | | 0.56 | | 3 | | 0.16 | | 1 | | 0.91 | | 3 | | 0.76 | | 3 | | 3 | | | 4 | | 7 | |  |  |  |  |
| P55290 | Cadherin-13 | CDH13 | 11.22 | 6 | | 78.2 | | 4.98 | | 1.03 | 2 | | 0.14 | | 1 | | 0.00 | | 0 | | 0.33 | | 1 | | 0.78 | | 2 | | 1.56 | | 5 | | 2.45 | | 6 | | 2.91 | | 6 | | 3 | | | 4 | | 7 | |  |  |  |  |
| A0A0C4DG49 | Poliovirus receptor | PVR | 15.35 | 5 | | 45.3 | | 6.52 | | 0.26 | 2 | | 0.46 | | 2 | | 0.00 | | 0 | | 0.17 | | 1 | | 0.81 | | 2 | | 1.87 | | 3 | | 0.44 | | 3 | | 1.55 | | 5 | | 3 | | | 4 | | 7 | |  |  |  |  |
| A0A0A6YYG9 | Protein ARPC4-TTLL3 | ARPC4-TTLL3 | 4.80 | 3 | | 71.7 | | 5.88 | | 0.51 | 2 | | 0.14 | | 1 | | 0.00 | | 0 | | 0.16 | | 1 | | 0.14 | | 1 | | 0.32 | | 2 | | 0.17 | | 1 | | 0.18 | | 1 | | 3 | | | 4 | | 7 | |  |  |  |  |
| P20020 | Plasma membrane calcium-transporting ATPase 1 | ATP2B1 | 8.85 | 1 | | 134.6 | | 5.91 | | 0.13 | 1 | | 0.00 | | 0 | | 0.31 | | 2 | | 0.31 | | 2 | | 1.45 | | 2 | | 1.63 | | 2 | | 0.59 | | 3 | | 1.24 | | 5 | | 3 | | | 4 | | 7 | |  |  |  |  |
| P62906 | 60S ribosomal protein L10a | RPL10A | 27.65 | 7 | | 24.8 | | 9.94 | | 0.28 | 1 | | 1.05 | | 5 | | 0.00 | | 0 | | 1.65 | | 5 | | 3.89 | | 5 | | 1.66 | | 4 | | 4.49 | | 6 | | 4.21 | | 6 | | 3 | | | 4 | | 7 | |  |  |  |  |
| P08758 | Annexin A5 | ANXA5 | 59.38 | 17 | | 35.9 | | 5.05 | | 0.28 | 1 | | 1.39 | | 3 | | 0.00 | | 0 | | 1.41 | | 3 | | 21.85 | | 6 | | 28.69 | | 6 | | 27.06 | | 6 | | 38.20 | | 6 | | 3 | | | 4 | | 7 | |  |  |  |  |
| P04083 | Annexin A1 | ANXA1 | 53.47 | 17 | | 38.7 | | 7.02 | | 0.57 | 1 | | 0.48 | | 2 | | 0.00 | | 0 | | 0.78 | | 2 | | 25.94 | | 6 | | 32.58 | | 6 | | 31.15 | | 6 | | 45.81 | | 6 | | 3 | | | 4 | | 7 | |  |  |  |  |
| P62304 | Small nuclear ribonucleoprotein E | SNRPE | 25.00 | 2 | | 10.8 | | 9.44 | | 0.15 | 1 | | 0.40 | | 2 | | 0.00 | | 0 | | 0.31 | | 2 | | 0.99 | | 3 | | 0.48 | | 2 | | 0.34 | | 2 | | 1.02 | | 4 | | 3 | | | 4 | | 7 | |  |  |  |  |
| Q8N4C8 | Misshapen-like kinase 1 | MINK1 | 2.78 | 1 | | 149.7 | | 7.85 | | 0.13 | 1 | | 0.13 | | 1 | | 0.00 | | 0 | | 0.21 | | 1 | | 0.99 | | 3 | | 0.71 | | 3 | | 1.49 | | 5 | | 0.81 | | 4 | | 3 | | | 4 | | 7 | |  |  |  |  |
| Q9UKE5 | TRAF2 and NCK-interacting protein kinase | TNIK | 1.32 | 1 | | 154.8 | | 7.17 | | 0.13 | 1 | | 0.13 | | 1 | | 0.00 | | 0 | | 0.21 | | 1 | | 0.99 | | 3 | | 0.71 | | 3 | | 1.49 | | 5 | | 0.81 | | 4 | | 3 | | | 4 | | 7 | |  |  |  |  |
| P11279 | Lysosome-associated membrane glycoprotein 1 | LAMP1 | 25.42 | 7 | | 44.9 | | 8.75 | | 0.13 | 1 | | 0.97 | | 2 | | 0.00 | | 0 | | 0.16 | | 1 | | 6.99 | | 6 | | 11.45 | | 6 | | 8.40 | | 6 | | 11.54 | | 6 | | 3 | | | 4 | | 7 | |  |  |  |  |
| P63244 | Receptor of activated protein C kinase 1 | RACK1 | 28.08 | 9 | | 35.1 | | 7.69 | | 0.33 | 1 | | 0.95 | | 4 | | 0.00 | | 0 | | 0.16 | | 1 | | 4.34 | | 4 | | 5.82 | | 4 | | 5.93 | | 6 | | 5.06 | | 6 | | 3 | | | 4 | | 7 | |  |  |  |  |
| Q15029 | 116 kDa U5 small nuclear ribonucleoprotein component | EFTUD2 | 8.74 | 5 | | 109.4 | | 5.00 | | 0.00 | 0 | | 0.14 | | 1 | | 0.66 | | 3 | | 0.21 | | 1 | | 3.63 | | 4 | | 1.38 | | 5 | | 0.14 | | 1 | | 0.46 | | 3 | | 3 | | | 4 | | 7 | |  |  |  |  |
| Q8TDR0 | TRAF3-interacting protein 1 | TRAF3IP1 | 1.59 | 1 | | 78.6 | | 7.93 | | 0.00 | 0 | | 0.73 | | 4 | | 0.38 | | 2 | | 0.58 | | 2 | | 1.37 | | 4 | | 0.50 | | 3 | | 0.79 | | 3 | | 2.02 | | 5 | | 3 | | | 4 | | 7 | |  |  |  |  |
| P58107 | Epiplakin | EPPK1 | 7.80 | 5 | | 555.3 | | 5.62 | | 0.57 | 4 | | 0.00 | | 0 | | 0.86 | | 3 | | 0.87 | | 3 | | 0.30 | | 2 | | 0.00 | | 0 | | 0.90 | | 2 | | 0.61 | | 2 | | 3 | | | 3 | | 6 | |  |  |  |  |
| P11217 | Glycogen phosphorylase, muscle form | PYGM | 3.56 | 1 | | 97.0 | | 7.03 | | 1.05 | 3 | | 0.14 | | 1 | | 0.63 | | 2 | | 0.00 | | 0 | | 0.00 | | 0 | | 0.16 | | 1 | | 0.14 | | 1 | | 0.15 | | 1 | | 3 | | | 3 | | 6 | |  |  |  |  |
| O43143 | Pre-mRNA-splicing factor ATP-dependent RNA helicase DHX15 | DHX15 | 10.19 | 6 | | 90.9 | | 7.46 | | 0.76 | 3 | | 0.27 | | 2 | | 0.16 | | 1 | | 0.00 | | 0 | | 1.83 | | 3 | | 0.79 | | 2 | | 0.14 | | 1 | | 0.00 | | 0 | | 3 | | | 3 | | 6 | |  |  |  |  |
| I3L4V6 | Nucleoredoxin (Fragment) | NXN | 16.09 | 2 | | 26.0 | | 4.77 | | 0.28 | 1 | | 0.00 | | 0 | | 0.16 | | 1 | | 0.31 | | 2 | | 0.16 | | 1 | | 0.00 | | 0 | | 0.62 | | 4 | | 0.31 | | 2 | | 3 | | | 3 | | 6 | |  |  |  |  |
| Q86UK7 | E3 ubiquitin-protein ligase ZNF598 | ZNF598 | 7.19 | 3 | | 98.6 | | 8.40 | | 0.00 | 0 | | 0.41 | | 2 | | 0.57 | | 3 | | 0.38 | | 2 | | 0.00 | | 0 | | 0.16 | | 1 | | 0.15 | | 1 | | 0.45 | | 3 | | 3 | | | 3 | | 6 | |  |  |  |  |
| Q9NYQ8 | Protocadherin Fat 2 | FAT2 | 3.47 | 5 | | 479.0 | | 5.16 | | 0.00 | 0 | | 0.13 | | 1 | | 0.16 | | 1 | | 0.36 | | 2 | | 0.28 | | 2 | | 0.16 | | 1 | | 0.30 | | 2 | | 0.00 | | 0 | | 3 | | | 3 | | 6 | |  |  |  |  |
| Q66LE6 | Serine/threonine-protein phosphatase 2A 55 kDa regulatory subunit B delta isoform | PPP2R2D | 3.09 | 1 | | 52.0 | | 6.39 | | 0.00 | 0 | | 0.54 | | 3 | | 0.16 | | 1 | | 0.16 | | 1 | | 0.00 | | 0 | | 0.16 | | 1 | | 0.60 | | 3 | | 1.06 | | 4 | | 3 | | | 3 | | 6 | |  |  |  |  |
| A0A3B3ITK0 | Thrombospondin-2 | THBS2 | 6.54 | 2 | | 130.4 | | 4.83 | | 3.61 | 6 | | 2.27 | | 5 | | 0.00 | | 0 | | 0.16 | | 1 | | 0.32 | | 1 | | 1.51 | | 4 | | 0.00 | | 0 | | 0.00 | | 0 | | 3 | | | 2 | | 5 | |  |  |  |  |
| P14174 | Macrophage migration inhibitory factor | MIF | 47.83 | 1 | | 12.5 | | 7.88 | | 1.06 | 3 | | 0.00 | | 0 | | 0.19 | | 1 | | 0.62 | | 1 | | 0.00 | | 0 | | 0.00 | | 0 | | 0.15 | | 1 | | 0.76 | | 3 | | 3 | | | 2 | | 5 | |  |  |  |  |
| P12111 | Collagen alpha-3(VI) chain | COL6A3 | 3.49 | 6 | | 343.5 | | 6.68 | | 0.64 | 2 | | 0.00 | | 0 | | 1.05 | | 3 | | 1.26 | | 2 | | 0.00 | | 0 | | 0.00 | | 0 | | 0.17 | | 1 | | 1.80 | | 2 | | 3 | | | 2 | | 5 | |  |  |  |  |
| O15020 | Spectrin beta chain, non-erythrocytic 2 | SPTBN2 | 1.97 | 3 | | 271.2 | | 6.11 | | 0.28 | 1 | | 0.32 | | 2 | | 0.98 | | 3 | | 0.00 | | 0 | | 0.14 | | 1 | | 0.18 | | 1 | | 0.00 | | 0 | | 0.00 | | 0 | | 3 | | | 2 | | 5 | |  |  |  |  |
| Q13185 | Chromobox protein homolog 3 | CBX3 | 15.85 | 2 | | 20.8 | | 5.33 | | 0.15 | 1 | | 0.55 | | 2 | | 0.00 | | 0 | | 0.62 | | 2 | | 0.85 | | 3 | | 0.00 | | 0 | | 0.34 | | 2 | | 0.00 | | 0 | | 3 | | | 2 | | 5 | |  |  |  |  |
| Q86TM6 | E3 ubiquitin-protein ligase synoviolin | SYVN1 | 3.73 | 1 | | 67.6 | | 6.95 | | 0.13 | 1 | | 0.27 | | 2 | | 0.00 | | 0 | | 0.16 | | 1 | | 0.16 | | 1 | | 0.00 | | 0 | | 0.00 | | 0 | | 0.15 | | 1 | | 3 | | | 2 | | 5 | |  |  |  |  |
| O60437 | Periplakin | PPL | 5.47 | 5 | | 204.6 | | 5.60 | | 0.00 | 0 | | 0.57 | | 3 | | 0.38 | | 2 | | 0.16 | | 1 | | 0.14 | | 1 | | 0.00 | | 0 | | 0.00 | | 0 | | 0.15 | | 1 | | 3 | | | 2 | | 5 | |  |  |  |  |
| P78417 | Glutathione S-transferase omega-1 | GSTO1 | 11.20 | 2 | | 27.5 | | 6.60 | | 0.73 | 4 | | 0.83 | | 2 | | 0.31 | | 2 | | 0.00 | | 0 | | 0.00 | | 0 | | 0.00 | | 0 | | 0.00 | | 0 | | 0.31 | | 1 | | 3 | | | 1 | | 4 | |  |  |  |  |
| P15121 | Aldo-keto reductase family 1 member B1 | AKR1B1 | 12.03 | 3 | | 35.8 | | 6.98 | | 1.99 | 4 | | 1.36 | | 4 | | 0.22 | | 1 | | 0.00 | | 0 | | 0.00 | | 0 | | 0.32 | | 2 | | 0.00 | | 0 | | 0.00 | | 0 | | 3 | | | 1 | | 4 | |  |  |  |  |
| P98160 | Basement membrane-specific heparan sulfate proteoglycan core protein | HSPG2 | 6.72 | 21 | | 468.5 | | 6.51 | | 4.22 | 4 | | 4.53 | | 5 | | 0.16 | | 1 | | 0.00 | | 0 | | 0.00 | | 0 | | 7.24 | | 4 | | 0.00 | | 0 | | 0.00 | | 0 | | 3 | | | 1 | | 4 | |  |  |  |  |
| F6SYF8 | Dickkopf-related protein 3 | DKK3 | 15.11 | 2 | | 39.9 | | 4.70 | | 0.39 | 2 | | 0.40 | | 2 | | 0.47 | | 2 | | 0.00 | | 0 | | 0.16 | | 1 | | 0.00 | | 0 | | 0.00 | | 0 | | 0.00 | | 0 | | 3 | | | 1 | | 4 | |  |  |  |  |
| Q7Z304 | MAM domain-containing protein 2 | MAMDC2 | 19.53 | 9 | | 77.5 | | 5.16 | | 1.70 | 2 | | 1.94 | | 2 | | 0.32 | | 1 | | 0.00 | | 0 | | 0.00 | | 0 | | 0.00 | | 0 | | 0.14 | | 1 | | 0.00 | | 0 | | 3 | | | 1 | | 4 | |  |  |  |  |
| Q02750 | Dual specificity mitogen-activated protein kinase kinase 1 | MAP2K1 | 5.09 | 2 | | 43.4 | | 6.62 | | 0.47 | 2 | | 0.27 | | 2 | | 0.16 | | 1 | | 0.00 | | 0 | | 0.00 | | 0 | | 0.32 | | 1 | | 0.00 | | 0 | | 0.00 | | 0 | | 3 | | | 1 | | 4 | |  |  |  |  |
| P11940 | Polyadenylate-binding protein 1 | PABPC1 | 15.57 | 3 | | 70.6 | | 9.50 | | 0.48 | 2 | | 0.55 | | 2 | | 0.00 | | 0 | | 0.84 | | 2 | | 1.69 | | 3 | | 0.00 | | 0 | | 0.00 | | 0 | | 0.00 | | 0 | | 3 | | | 1 | | 4 | |  |  |  |  |
| P31689 | DnaJ homolog subfamily A member 1 | DNAJA1 | 8.06 | 2 | | 44.8 | | 7.08 | | 0.41 | 2 | | 0.16 | | 1 | | 0.00 | | 0 | | 0.32 | | 2 | | 0.56 | | 2 | | 0.00 | | 0 | | 0.00 | | 0 | | 0.00 | | 0 | | 3 | | | 1 | | 4 | |  |  |  |  |
| Q8NBP0 | Tetratricopeptide repeat protein 13 | TTC13 | 12.09 | 4 | | 96.8 | | 7.01 | | 0.29 | 2 | | 0.32 | | 1 | | 0.00 | | 0 | | 0.17 | | 1 | | 0.00 | | 0 | | 0.00 | | 0 | | 0.00 | | 0 | | 0.33 | | 2 | | 3 | | | 1 | | 4 | |  |  |  |  |
| O00567 | Nucleolar protein 56 | NOP56 | 14.65 | 6 | | 66.0 | | 9.19 | | 0.39 | 1 | | 0.16 | | 1 | | 0.50 | | 2 | | 0.00 | | 0 | | 2.96 | | 3 | | 0.00 | | 0 | | 0.00 | | 0 | | 0.00 | | 0 | | 3 | | | 1 | | 4 | |  |  |  |  |
| Q8IVL0 | Neuron navigator 3 | NAV3 | 1.76 | 2 | | 255.5 | | 8.76 | | 0.13 | 1 | | 0.27 | | 2 | | 0.38 | | 2 | | 0.00 | | 0 | | 0.00 | | 0 | | 0.00 | | 0 | | 0.00 | | 0 | | 0.15 | | 1 | | 3 | | | 1 | | 4 | |  |  |  |  |
| Q9HCE9 | Anoctamin-8 | ANO8 | 0.73 | 1 | | 135.9 | | 5.82 | | 0.15 | 1 | | 0.55 | | 3 | | 0.31 | | 2 | | 0.00 | | 0 | | 0.00 | | 0 | | 0.23 | | 1 | | 0.00 | | 0 | | 0.00 | | 0 | | 3 | | | 1 | | 4 | |  |  |  |  |
| P54727 | UV excision repair protein RAD23 homolog B | RAD23B | 12.22 | 3 | | 43.1 | | 4.84 | | 0.13 | 1 | | 0.40 | | 3 | | 0.31 | | 1 | | 0.00 | | 0 | | 0.28 | | 2 | | 0.00 | | 0 | | 0.00 | | 0 | | 0.00 | | 0 | | 3 | | | 1 | | 4 | |  |  |  |  |
| A7E2Y1 | Myosin-7B | MYH7B | 0.91 | 2 | | 225.7 | | 6.09 | | 0.13 | 1 | | 0.00 | | 0 | | 0.22 | | 1 | | 0.90 | | 3 | | 0.00 | | 0 | | 0.00 | | 0 | | 0.14 | | 1 | | 0.00 | | 0 | | 3 | | | 1 | | 4 | |  |  |  |  |
| P24534 | Elongation factor 1-beta | EEF1B2 | 20.44 | 3 | | 24.7 | | 4.67 | | 0.13 | 1 | | 1.22 | | 4 | | 0.00 | | 0 | | 0.47 | | 2 | | 0.71 | | 3 | | 0.00 | | 0 | | 0.00 | | 0 | | 0.00 | | 0 | | 3 | | | 1 | | 4 | |  |  |  |  |
| E9PB61 | THO complex subunit 4 | ALYREF | 14.39 | 2 | | 27.5 | | 11.05 | | 0.26 | 1 | | 0.68 | | 3 | | 0.00 | | 0 | | 0.42 | | 1 | | 0.00 | | 0 | | 0.00 | | 0 | | 0.00 | | 0 | | 0.15 | | 1 | | 3 | | | 1 | | 4 | |  |  |  |  |
| P26583 | High mobility group protein B2 | HMGB2 | 18.18 | 2 | | 24.0 | | 7.81 | | 0.13 | 1 | | 0.96 | | 4 | | 0.00 | | 0 | | 0.31 | | 1 | | 0.00 | | 0 | | 0.00 | | 0 | | 0.14 | | 1 | | 0.00 | | 0 | | 3 | | | 1 | | 4 | |  |  |  |  |
| O15091 | Mitochondrial ribonuclease P catalytic subunit | PRORP | 3.43 | 1 | | 67.3 | | 8.78 | | 0.13 | 1 | | 0.14 | | 1 | | 0.00 | | 0 | | 0.21 | | 1 | | 0.00 | | 0 | | 0.00 | | 0 | | 0.30 | | 2 | | 0.00 | | 0 | | 3 | | | 1 | | 4 | |  |  |  |  |
| Q5T3U5 | Multidrug resistance-associated protein 7 | ABCC10 | 3.89 | 2 | | 161.5 | | 7.08 | | 0.28 | 1 | | 0.14 | | 1 | | 0.00 | | 0 | | 0.16 | | 1 | | 0.14 | | 1 | | 0.00 | | 0 | | 0.00 | | 0 | | 0.00 | | 0 | | 3 | | | 1 | | 4 | |  |  |  |  |
| H0Y7Z9 | Receptor-type tyrosine-protein phosphatase F (Fragment) | PTPRF | 1.80 | 1 | | 150.4 | | 6.62 | | 0.00 | 0 | | 0.62 | | 3 | | 0.19 | | 1 | | 0.66 | | 2 | | 0.00 | | 0 | | 0.24 | | 1 | | 0.00 | | 0 | | 0.00 | | 0 | | 3 | | | 1 | | 4 | |  |  |  |  |
| H0Y6Z7 | Receptor-type tyrosine-protein phosphatase F (Fragment) | PTPRF | 2.70 | 1 | | 174.8 | | 6.60 | | 0.00 | 0 | | 0.62 | | 3 | | 0.19 | | 1 | | 0.66 | | 2 | | 0.00 | | 0 | | 0.00 | | 0 | | 0.00 | | 0 | | 0.15 | | 1 | | 3 | | | 1 | | 4 | |  |  |  |  |
| P51511 | Matrix metalloproteinase-15 | MMP15 | 2.69 | 1 | | 75.8 | | 7.46 | | 0.00 | 0 | | 0.32 | | 2 | | 0.19 | | 1 | | 0.49 | | 3 | | 0.00 | | 0 | | 0.00 | | 0 | | 0.00 | | 0 | | 0.36 | | 2 | | 3 | | | 1 | | 4 | |  |  |  |  |
| P30530 | Tyrosine-protein kinase receptor UFO | AXL | 3.24 | 2 | | 98.3 | | 5.39 | | 2.10 | 5 | | 1.89 | | 6 | | 0.63 | | 2 | | 0.00 | | 0 | | 0.00 | | 0 | | 0.00 | | 0 | | 0.00 | | 0 | | 0.00 | | 0 | | 3 | | | 0 | | 3 | |  |  |  |  |
| O75326 | Semaphorin-7A | SEMA7A | 14.86 | 6 | | 74.8 | | 7.64 | | 2.52 | 4 | | 1.67 | | 4 | | 0.16 | | 1 | | 0.00 | | 0 | | 0.00 | | 0 | | 0.00 | | 0 | | 0.00 | | 0 | | 0.00 | | 0 | | 3 | | | 0 | | 3 | |  |  |  |  |
| Q02818 | Nucleobindin-1 | NUCB1 | 20.82 | 7 | | 53.8 | | 5.25 | | 1.97 | 4 | | 3.55 | | 6 | | 0.00 | | 0 | | 0.93 | | 2 | | 0.00 | | 0 | | 0.00 | | 0 | | 0.00 | | 0 | | 0.00 | | 0 | | 3 | | | 0 | | 3 | |  |  |  |  |
| P10124 | Serglycin | SRGN | 8.23 | 1 | | 17.6 | | 4.96 | | 0.85 | 4 | | 0.32 | | 2 | | 0.00 | | 0 | | 0.49 | | 2 | | 0.00 | | 0 | | 0.00 | | 0 | | 0.00 | | 0 | | 0.00 | | 0 | | 3 | | | 0 | | 3 | |  |  |  |  |
| P00491 | Purine nucleoside phosphorylase | PNP | 12.80 | 3 | | 32.1 | | 6.95 | | 0.55 | 3 | | 0.44 | | 3 | | 0.79 | | 2 | | 0.00 | | 0 | | 0.00 | | 0 | | 0.00 | | 0 | | 0.00 | | 0 | | 0.00 | | 0 | | 3 | | | 0 | | 3 | |  |  |  |  |
| P06737 | Glycogen phosphorylase, liver form | PYGL | 3.54 | 1 | | 97.1 | | 7.17 | | 1.05 | 3 | | 0.40 | | 3 | | 0.63 | | 2 | | 0.00 | | 0 | | 0.00 | | 0 | | 0.00 | | 0 | | 0.00 | | 0 | | 0.00 | | 0 | | 3 | | | 0 | | 3 | |  |  |  |  |
| Q5T200 | Zinc finger CCCH domain-containing protein 13 | ZC3H13 | 0.48 | 1 | | 196.5 | | 9.42 | | 0.68 | 3 | | 0.32 | | 2 | | 0.31 | | 2 | | 0.00 | | 0 | | 0.00 | | 0 | | 0.00 | | 0 | | 0.00 | | 0 | | 0.00 | | 0 | | 3 | | | 0 | | 3 | |  |  |  |  |
| O00625 | Pirin | PIR | 15.52 | 3 | | 32.1 | | 6.92 | | 0.80 | 3 | | 0.81 | | 4 | | 0.31 | | 1 | | 0.00 | | 0 | | 0.00 | | 0 | | 0.00 | | 0 | | 0.00 | | 0 | | 0.00 | | 0 | | 3 | | | 0 | | 3 | |  |  |  |  |
| Q9C005 | Protein dpy-30 homolog | DPY30 | 36.36 | 2 | | 11.2 | | 4.88 | | 0.93 | 3 | | 1.64 | | 4 | | 0.00 | | 0 | | 0.99 | | 3 | | 0.00 | | 0 | | 0.00 | | 0 | | 0.00 | | 0 | | 0.00 | | 0 | | 3 | | | 0 | | 3 | |  |  |  |  |
| P61812 | Transforming growth factor beta-2 proprotein | TGFB2 | 10.14 | 3 | | 47.7 | | 8.53 | | 0.64 | 2 | | 0.16 | | 1 | | 1.89 | | 4 | | 0.00 | | 0 | | 0.00 | | 0 | | 0.00 | | 0 | | 0.00 | | 0 | | 0.00 | | 0 | | 3 | | | 0 | | 3 | |  |  |  |  |
| Q6P5Z2 | Serine/threonine-protein kinase N3 | PKN3 | 2.36 | 1 | | 99.4 | | 8.46 | | 0.45 | 2 | | 0.00 | | 0 | | 1.04 | | 3 | | 0.21 | | 1 | | 0.00 | | 0 | | 0.00 | | 0 | | 0.00 | | 0 | | 0.00 | | 0 | | 3 | | | 0 | | 3 | |  |  |  |  |
| P00750 | Tissue-type plasminogen activator | PLAT | 9.25 | 3 | | 62.9 | | 7.80 | | 0.29 | 2 | | 0.16 | | 1 | | 0.85 | | 3 | | 0.00 | | 0 | | 0.00 | | 0 | | 0.00 | | 0 | | 0.00 | | 0 | | 0.00 | | 0 | | 3 | | | 0 | | 3 | |  |  |  |  |
| Q9BRA2 | Thioredoxin domain-containing protein 17 | TXNDC17 | 19.51 | 2 | | 13.9 | | 5.52 | | 0.51 | 2 | | 1.36 | | 4 | | 0.63 | | 2 | | 0.00 | | 0 | | 0.00 | | 0 | | 0.00 | | 0 | | 0.00 | | 0 | | 0.00 | | 0 | | 3 | | | 0 | | 3 | |  |  |  |  |
| A0A5H1ZRS3 | Enolase 4 | ENO4 | 1.43 | 1 | | 68.7 | | 6.00 | | 0.51 | 2 | | 0.16 | | 1 | | 0.19 | | 1 | | 0.00 | | 0 | | 0.00 | | 0 | | 0.00 | | 0 | | 0.00 | | 0 | | 0.00 | | 0 | | 3 | | | 0 | | 3 | |  |  |  |  |
| O43293 | Death-associated protein kinase 3 | DAPK3 | 3.74 | 1 | | 52.5 | | 6.89 | | 0.28 | 2 | | 0.16 | | 1 | | 0.19 | | 1 | | 0.00 | | 0 | | 0.00 | | 0 | | 0.00 | | 0 | | 0.00 | | 0 | | 0.00 | | 0 | | 3 | | | 0 | | 3 | |  |  |  |  |
| Q99797 | Mitochondrial intermediate peptidase | MIPEP | 1.12 | 1 | | 80.6 | | 7.05 | | 0.26 | 2 | | 0.16 | | 1 | | 0.00 | | 0 | | 0.16 | | 1 | | 0.00 | | 0 | | 0.00 | | 0 | | 0.00 | | 0 | | 0.00 | | 0 | | 3 | | | 0 | | 3 | |  |  |  |  |
| Q96G03 | Phosphoglucomutase-2 | PGM2 | 2.94 | 1 | | 68.2 | | 6.73 | | 0.26 | 1 | | 1.22 | | 4 | | 0.95 | | 2 | | 0.00 | | 0 | | 0.00 | | 0 | | 0.00 | | 0 | | 0.00 | | 0 | | 0.00 | | 0 | | 3 | | | 0 | | 3 | |  |  |  |  |
| Q9Y6X8 | Zinc fingers and homeoboxes protein 2 | ZHX2 | 1.08 | 1 | | 92.3 | | 6.86 | | 0.13 | 1 | | 0.14 | | 1 | | 0.50 | | 3 | | 0.00 | | 0 | | 0.00 | | 0 | | 0.00 | | 0 | | 0.00 | | 0 | | 0.00 | | 0 | | 3 | | | 0 | | 3 | |  |  |  |  |
| Q9NR12 | PDZ and LIM domain protein 7 | PDLIM7 | 2.63 | 1 | | 49.8 | | 8.41 | | 0.13 | 1 | | 0.27 | | 2 | | 0.47 | | 2 | | 0.00 | | 0 | | 0.00 | | 0 | | 0.00 | | 0 | | 0.00 | | 0 | | 0.00 | | 0 | | 3 | | | 0 | | 3 | |  |  |  |  |
| Q9H6D7 | HAUS augmin-like complex subunit 4 | HAUS4 | 2.75 | 1 | | 42.4 | | 5.68 | | 0.28 | 1 | | 0.30 | | 2 | | 0.41 | | 2 | | 0.00 | | 0 | | 0.00 | | 0 | | 0.00 | | 0 | | 0.00 | | 0 | | 0.00 | | 0 | | 3 | | | 0 | | 3 | |  |  |  |  |
| O75821 | Eukaryotic translation initiation factor 3 subunit G | EIF3G | 2.81 | 1 | | 35.6 | | 6.13 | | 0.13 | 1 | | 0.27 | | 2 | | 0.32 | | 1 | | 0.00 | | 0 | | 0.00 | | 0 | | 0.00 | | 0 | | 0.00 | | 0 | | 0.00 | | 0 | | 3 | | | 0 | | 3 | |  |  |  |  |
| Q9NZ20 | Group 3 secretory phospholipase A2 | PLA2G3 | 7.86 | 2 | | 57.1 | | 9.07 | | 0.13 | 1 | | 0.14 | | 1 | | 0.32 | | 1 | | 0.00 | | 0 | | 0.00 | | 0 | | 0.00 | | 0 | | 0.00 | | 0 | | 0.00 | | 0 | | 3 | | | 0 | | 3 | |  |  |  |  |
| P36955 | Pigment epithelium-derived factor | SERPINF1 | 6.22 | 2 | | 46.3 | | 6.38 | | 0.57 | 1 | | 0.00 | | 0 | | 0.22 | | 1 | | 0.21 | | 1 | | 0.00 | | 0 | | 0.00 | | 0 | | 0.00 | | 0 | | 0.00 | | 0 | | 3 | | | 0 | | 3 | |  |  |  |  |
| D6REC3 | Phospholipid phosphatase 1 | PLPP1 | 20.00 | 1 | | 12.3 | | 7.85 | | 0.28 | 1 | | 0.27 | | 2 | | 0.16 | | 1 | | 0.00 | | 0 | | 0.00 | | 0 | | 0.00 | | 0 | | 0.00 | | 0 | | 0.00 | | 0 | | 3 | | | 0 | | 3 | |  |  |  |  |
| A0A140T9Z1 | RNA cytosine C(5)-methyltransferase NSUN2 (Fragment) | NSUN2 | 11.76 | 1 | | 11.7 | | 7.40 | | 0.15 | 1 | | 0.14 | | 1 | | 0.16 | | 1 | | 0.00 | | 0 | | 0.00 | | 0 | | 0.00 | | 0 | | 0.00 | | 0 | | 0.00 | | 0 | | 3 | | | 0 | | 3 | |  |  |  |  |
| E9PF18 | Hydroxyacyl-coenzyme A dehydrogenase, mitochondrial | HADH | 7.69 | 1 | | 42.1 | | 9.26 | | 0.15 | 1 | | 0.14 | | 1 | | 0.16 | | 1 | | 0.00 | | 0 | | 0.00 | | 0 | | 0.00 | | 0 | | 0.00 | | 0 | | 0.00 | | 0 | | 3 | | | 0 | | 3 | |  |  |  |  |
| P17174 | Aspartate aminotransferase, cytoplasmic | GOT1 | 4.84 | 1 | | 46.2 | | 7.01 | | 0.13 | 1 | | 0.00 | | 0 | | 0.16 | | 1 | | 0.31 | | 1 | | 0.00 | | 0 | | 0.00 | | 0 | | 0.00 | | 0 | | 0.00 | | 0 | | 3 | | | 0 | | 3 | |  |  |  |  |
| P08397 | Porphobilinogen deaminase | HMBS | 5.54 | 1 | | 39.3 | | 7.18 | | 0.16 | 1 | | 0.41 | | 2 | | 0.16 | | 1 | | 0.00 | | 0 | | 0.00 | | 0 | | 0.00 | | 0 | | 0.00 | | 0 | | 0.00 | | 0 | | 3 | | | 0 | | 3 | |  |  |  |  |
| P40692 | DNA mismatch repair protein Mlh1 | MLH1 | 1.19 | 1 | | 84.5 | | 5.72 | | 0.13 | 1 | | 0.30 | | 2 | | 0.16 | | 1 | | 0.00 | | 0 | | 0.00 | | 0 | | 0.00 | | 0 | | 0.00 | | 0 | | 0.00 | | 0 | | 3 | | | 0 | | 3 | |  |  |  |  |
| A0A499FI48 | Protein disulfide-isomerase | PDIA4 | 5.26 | 3 | | 73.0 | | 5.07 | | 0.13 | 1 | | 1.21 | | 2 | | 0.00 | | 0 | | 1.09 | | 2 | | 0.00 | | 0 | | 0.00 | | 0 | | 0.00 | | 0 | | 0.00 | | 0 | | 3 | | | 0 | | 3 | |  |  |  |  |
| Q9BXB7 | Spermatogenesis-associated protein 16 | SPATA16 | 1.93 | 1 | | 65.2 | | 9.14 | | 0.15 | 1 | | 0.55 | | 2 | | 0.00 | | 0 | | 0.47 | | 2 | | 0.00 | | 0 | | 0.00 | | 0 | | 0.00 | | 0 | | 0.00 | | 0 | | 3 | | | 0 | | 3 | |  |  |  |  |
| K7ENK6 | Interleukin enhancer-binding factor 3 | ILF3 | 31.65 | 1 | | 8.9 | | 4.60 | | 0.16 | 1 | | 0.30 | | 2 | | 0.00 | | 0 | | 0.21 | | 1 | | 0.00 | | 0 | | 0.00 | | 0 | | 0.00 | | 0 | | 0.00 | | 0 | | 3 | | | 0 | | 3 | |  |  |  |  |
| P25054 | Adenomatous polyposis coli protein | APC | 2.22 | 2 | | 311.5 | | 7.80 | | 0.15 | 1 | | 0.27 | | 2 | | 0.00 | | 0 | | 0.21 | | 1 | | 0.00 | | 0 | | 0.00 | | 0 | | 0.00 | | 0 | | 0.00 | | 0 | | 3 | | | 0 | | 3 | |  |  |  |  |
| H7BY58 | Protein-L-isoaspartate O-methyltransferase | PCMT1 | 23.43 | 3 | | 30.3 | | 6.73 | | 0.16 | 1 | | 0.28 | | 1 | | 0.00 | | 0 | | 0.21 | | 1 | | 0.00 | | 0 | | 0.00 | | 0 | | 0.00 | | 0 | | 0.00 | | 0 | | 3 | | | 0 | | 3 | |  |  |  |  |
| P15391 | B-lymphocyte antigen CD19 | CD19 | 9.89 | 2 | | 61.1 | | 4.98 | | 0.13 | 1 | | 0.27 | | 1 | | 0.00 | | 0 | | 0.21 | | 1 | | 0.00 | | 0 | | 0.00 | | 0 | | 0.00 | | 0 | | 0.00 | | 0 | | 3 | | | 0 | | 3 | |  |  |  |  |
| Q13439 | Golgin subfamily A member 4 | GOLGA4 | 1.39 | 3 | | 261.0 | | 5.39 | | 0.13 | 1 | | 0.27 | | 2 | | 0.00 | | 0 | | 0.17 | | 1 | | 0.00 | | 0 | | 0.00 | | 0 | | 0.00 | | 0 | | 0.00 | | 0 | | 3 | | | 0 | | 3 | |  |  |  |  |
| P83110 | Serine protease HTRA3 | HTRA3 | 3.31 | 1 | | 48.6 | | 7.09 | | 0.13 | 1 | | 0.14 | | 1 | | 0.00 | | 0 | | 0.17 | | 1 | | 0.00 | | 0 | | 0.00 | | 0 | | 0.00 | | 0 | | 0.00 | | 0 | | 3 | | | 0 | | 3 | |  |  |  |  |
| O95273 | Cyclin-D1-binding protein 1 | CCNDBP1 | 2.50 | 1 | | 40.2 | | 4.82 | | 0.15 | 1 | | 0.14 | | 1 | | 0.00 | | 0 | | 0.16 | | 1 | | 0.00 | | 0 | | 0.00 | | 0 | | 0.00 | | 0 | | 0.00 | | 0 | | 3 | | | 0 | | 3 | |  |  |  |  |
| Q99715 | Collagen alpha-1(XII) chain | COL12A1 | 8.72 | 20 | | 332.9 | | 5.53 | | 0.00 | 0 | | 1.14 | | 2 | | 7.52 | | 4 | | 3.45 | | 5 | | 0.00 | | 0 | | 0.00 | | 0 | | 0.00 | | 0 | | 0.00 | | 0 | | 3 | | | 0 | | 3 | |  |  |  |  |
| Q8NI77 | Kinesin-like protein KIF18A | KIF18A | 0.89 | 1 | | 102.2 | | 8.91 | | 0.00 | 0 | | 1.32 | | 5 | | 1.20 | | 4 | | 2.33 | | 6 | | 0.00 | | 0 | | 0.00 | | 0 | | 0.00 | | 0 | | 0.00 | | 0 | | 3 | | | 0 | | 3 | |  |  |  |  |
| P19827 | Inter-alpha-trypsin inhibitor heavy chain H1 | ITIH1 | 1.10 | 1 | | 101.3 | | 6.79 | | 0.00 | 0 | | 0.14 | | 1 | | 0.67 | | 2 | | 1.17 | | 4 | | 0.00 | | 0 | | 0.00 | | 0 | | 0.00 | | 0 | | 0.00 | | 0 | | 3 | | | 0 | | 3 | |  |  |  |  |
| Q6DHV7 | Adenosine deaminase-like protein | ADAL | 4.51 | 2 | | 40.2 | | 6.32 | | 0.00 | 0 | | 0.16 | | 1 | | 0.57 | | 2 | | 0.53 | | 3 | | 0.00 | | 0 | | 0.00 | | 0 | | 0.00 | | 0 | | 0.00 | | 0 | | 3 | | | 0 | | 3 | |  |  |  |  |
| Q92766 | Ras-responsive element-binding protein 1 | RREB1 | 1.13 | 1 | | 181.3 | | 6.98 | | 0.00 | 0 | | 0.14 | | 1 | | 0.54 | | 3 | | 0.16 | | 1 | | 0.00 | | 0 | | 0.00 | | 0 | | 0.00 | | 0 | | 0.00 | | 0 | | 3 | | | 0 | | 3 | |  |  |  |  |
| Q8TAD8 | Smad nuclear-interacting protein 1 | SNIP1 | 3.03 | 1 | | 45.8 | | 9.99 | | 0.00 | 0 | | 0.70 | | 3 | | 0.47 | | 2 | | 0.31 | | 2 | | 0.00 | | 0 | | 0.00 | | 0 | | 0.00 | | 0 | | 0.00 | | 0 | | 3 | | | 0 | | 3 | |  |  |  |  |
| Q15637 | Splicing factor 1 | SF1 | 5.95 | 2 | | 68.3 | | 8.98 | | 0.00 | 0 | | 0.71 | | 3 | | 0.44 | | 2 | | 0.16 | | 1 | | 0.00 | | 0 | | 0.00 | | 0 | | 0.00 | | 0 | | 0.00 | | 0 | | 3 | | | 0 | | 3 | |  |  |  |  |
| P23141 | Liver carboxylesterase 1 | CES1 | 14.81 | 2 | | 62.5 | | 6.60 | | 0.00 | 0 | | 0.14 | | 1 | | 0.38 | | 1 | | 0.21 | | 1 | | 0.00 | | 0 | | 0.00 | | 0 | | 0.00 | | 0 | | 0.00 | | 0 | | 3 | | | 0 | | 3 | |  |  |  |  |
| Q96RW7 | Hemicentin-1 | HMCN1 | 0.46 | 2 | | 613.0 | | 6.49 | | 0.00 | 0 | | 0.14 | | 1 | | 0.38 | | 1 | | 0.21 | | 1 | | 0.00 | | 0 | | 0.00 | | 0 | | 0.00 | | 0 | | 0.00 | | 0 | | 3 | | | 0 | | 3 | |  |  |  |  |
| P04217 | Alpha-1B-glycoprotein | A1BG | 1.41 | 1 | | 54.2 | | 5.86 | | 0.00 | 0 | | 0.16 | | 1 | | 0.22 | | 1 | | 0.16 | | 1 | | 0.00 | | 0 | | 0.00 | | 0 | | 0.00 | | 0 | | 0.00 | | 0 | | 3 | | | 0 | | 3 | |  |  |  |  |
| E7EVA0 | Microtubule-associated protein | MAP4 | 2.22 | 3 | | 245.3 | | 6.23 | | 0.00 | 0 | | 0.54 | | 4 | | 0.19 | | 1 | | 0.47 | | 2 | | 0.00 | | 0 | | 0.00 | | 0 | | 0.00 | | 0 | | 0.00 | | 0 | | 3 | | | 0 | | 3 | |  |  |  |  |
| Q96DL1 | NXPE family member 2 | NXPE2 | 1.61 | 1 | | 64.9 | | 8.60 | | 0.00 | 0 | | 0.13 | | 1 | | 0.19 | | 1 | | 0.16 | | 1 | | 0.00 | | 0 | | 0.00 | | 0 | | 0.00 | | 0 | | 0.00 | | 0 | | 3 | | | 0 | | 3 | |  |  |  |  |
| B0QYW2 | PHD finger protein 21B (Fragment) | PHF21B | 4.19 | 1 | | 19.7 | | 10.33 | | 0.00 | 0 | | 0.27 | | 2 | | 0.16 | | 1 | | 0.21 | | 1 | | 0.00 | | 0 | | 0.00 | | 0 | | 0.00 | | 0 | | 0.00 | | 0 | | 3 | | | 0 | | 3 | |  |  |  |  |
| A0A494C0G5 | Agrin | AGRN | 11.75 | 3 | | 203.0 | | 6.55 | | 4.78 | 5 | | 5.55 | | 4 | | 0.00 | | 0 | | 0.00 | | 0 | | 1.85 | | 4 | | 23.84 | | 6 | | 3.94 | | 4 | | 0.87 | | 3 | | 2 | | | 4 | | 6 | |  |  |  |  |
| O00468 | Agrin | AGRN | 10.40 | 1 | | 217.2 | | 6.39 | | 4.64 | 5 | | 5.65 | | 4 | | 0.00 | | 0 | | 0.00 | | 0 | | 1.85 | | 4 | | 20.95 | | 6 | | 3.34 | | 4 | | 0.69 | | 3 | | 2 | | | 4 | | 6 | |  |  |  |  |
| Q15323 | Keratin, type I cuticular Ha1 | KRT31 | 17.31 | 2 | | 47.2 | | 4.88 | | 1.13 | 4 | | 0.00 | | 0 | | 1.48 | | 6 | | 0.00 | | 0 | | 4.19 | | 5 | | 3.35 | | 4 | | 1.18 | | 4 | | 0.76 | | 4 | | 2 | | | 4 | | 6 | |  |  |  |  |
| P16402 | Histone H1.3 | H1-3 | 22.62 | 7 | | 22.3 | | 11.02 | | 1.35 | 4 | | 0.56 | | 3 | | 0.00 | | 0 | | 0.00 | | 0 | | 15.15 | | 5 | | 8.31 | | 4 | | 19.13 | | 6 | | 15.44 | | 6 | | 2 | | | 4 | | 6 | |  |  |  |  |
| P16401 | Histone H1.5 | H1-5 | 18.14 | 5 | | 22.6 | | 10.92 | | 0.89 | 3 | | 0.00 | | 0 | | 0.67 | | 2 | | 0.00 | | 0 | | 10.92 | | 5 | | 6.80 | | 4 | | 15.43 | | 6 | | 13.84 | | 6 | | 2 | | | 4 | | 6 | |  |  |  |  |
| B1AHL2 | Fibulin-1 | FBLN1 | 5.55 | 2 | | 78.3 | | 5.39 | | 0.42 | 3 | | 0.73 | | 3 | | 0.00 | | 0 | | 0.00 | | 0 | | 1.49 | | 5 | | 1.68 | | 4 | | 0.15 | | 1 | | 0.54 | | 2 | | 2 | | | 4 | | 6 | |  |  |  |  |
| Q13443 | Disintegrin and metalloproteinase domain-containing protein 9 | ADAM9 | 5.74 | 5 | | 90.5 | | 7.52 | | 0.81 | 3 | | 0.27 | | 1 | | 0.00 | | 0 | | 0.00 | | 0 | | 0.48 | | 2 | | 0.91 | | 2 | | 0.15 | | 1 | | 0.33 | | 2 | | 2 | | | 4 | | 6 | |  |  |  |  |
| P62917 | 60S ribosomal protein L8 | RPL8 | 31.13 | 7 | | 28.0 | | 11.03 | | 0.47 | 2 | | 0.00 | | 0 | | 0.00 | | 0 | | 0.52 | | 2 | | 4.17 | | 5 | | 4.31 | | 6 | | 4.10 | | 6 | | 4.83 | | 6 | | 2 | | | 4 | | 6 | |  |  |  |  |
| P17655 | Calpain-2 catalytic subunit | CAPN2 | 8.57 | 3 | | 79.9 | | 4.98 | | 0.28 | 2 | | 0.00 | | 0 | | 0.00 | | 0 | | 0.21 | | 1 | | 0.56 | | 2 | | 0.16 | | 1 | | 0.76 | | 4 | | 0.15 | | 1 | | 2 | | | 4 | | 6 | |  |  |  |  |
| Q08431 | Lactadherin | MFGE8 | 44.44 | 15 | | 43.1 | | 8.15 | | 0.57 | 2 | | 1.77 | | 2 | | 0.00 | | 0 | | 0.00 | | 0 | | 19.54 | | 6 | | 16.95 | | 6 | | 20.39 | | 6 | | 21.75 | | 6 | | 2 | | | 4 | | 6 | |  |  |  |  |
| P62269 | 40S ribosomal protein S18 | RPS18 | 48.68 | 11 | | 17.7 | | 10.99 | | 0.48 | 2 | | 0.54 | | 2 | | 0.00 | | 0 | | 0.00 | | 0 | | 11.73 | | 5 | | 7.78 | | 6 | | 13.07 | | 6 | | 14.32 | | 6 | | 2 | | | 4 | | 6 | |  |  |  |  |
| F5H423 | Uncharacterized protein |  | 44.76 | 3 | | 23.3 | | 8.60 | | 0.32 | 2 | | 1.78 | | 2 | | 0.00 | | 0 | | 0.00 | | 0 | | 2.18 | | 4 | | 2.72 | | 3 | | 4.77 | | 6 | | 5.89 | | 6 | | 2 | | | 4 | | 6 | |  |  |  |  |
| P61160 | Actin-related protein 2 | ACTR2 | 17.26 | 4 | | 44.7 | | 6.74 | | 0.47 | 2 | | 0.41 | | 2 | | 0.00 | | 0 | | 0.00 | | 0 | | 2.11 | | 3 | | 1.92 | | 3 | | 1.53 | | 5 | | 1.73 | | 5 | | 2 | | | 4 | | 6 | |  |  |  |  |
| O43390 | Heterogeneous nuclear ribonucleoprotein R | HNRNPR | 5.21 | 1 | | 70.9 | | 8.13 | | 0.32 | 2 | | 0.41 | | 2 | | 0.00 | | 0 | | 0.00 | | 0 | | 1.55 | | 3 | | 0.97 | | 3 | | 1.49 | | 3 | | 0.15 | | 1 | | 2 | | | 4 | | 6 | |  |  |  |  |
| P18124 | 60S ribosomal protein L7 | RPL7 | 31.45 | 11 | | 29.2 | | 10.65 | | 0.15 | 1 | | 0.00 | | 0 | | 0.00 | | 0 | | 0.47 | | 2 | | 8.00 | | 5 | | 4.33 | | 6 | | 4.66 | | 6 | | 6.15 | | 6 | | 2 | | | 4 | | 6 | |  |  |  |  |
| P01893 | Putative HLA class I histocompatibility antigen, alpha chain H | HLA-H | 24.59 | 3 | | 40.9 | | 6.30 | | 0.33 | 1 | | 0.00 | | 0 | | 0.00 | | 0 | | 0.16 | | 1 | | 2.13 | | 4 | | 3.02 | | 4 | | 3.77 | | 6 | | 4.38 | | 6 | | 2 | | | 4 | | 6 | |  |  |  |  |
| P39023 | 60S ribosomal protein L3 | RPL3 | 25.81 | 9 | | 46.1 | | 10.18 | | 0.15 | 1 | | 0.00 | | 0 | | 0.00 | | 0 | | 0.16 | | 1 | | 9.89 | | 4 | | 2.88 | | 3 | | 9.06 | | 6 | | 7.85 | | 6 | | 2 | | | 4 | | 6 | |  |  |  |  |
| P38919 | Eukaryotic initiation factor 4A-III | EIF4A3 | 9.00 | 1 | | 46.8 | | 6.73 | | 0.16 | 1 | | 0.14 | | 1 | | 0.00 | | 0 | | 0.00 | | 0 | | 0.89 | | 5 | | 1.60 | | 5 | | 1.24 | | 6 | | 0.69 | | 3 | | 2 | | | 4 | | 6 | |  |  |  |  |
| Q92499 | ATP-dependent RNA helicase DDX1 | DDX1 | 5.95 | 3 | | 82.4 | | 7.23 | | 0.15 | 1 | | 0.55 | | 2 | | 0.00 | | 0 | | 0.00 | | 0 | | 0.99 | | 3 | | 0.95 | | 2 | | 1.39 | | 6 | | 1.25 | | 5 | | 2 | | | 4 | | 6 | |  |  |  |  |
| P13473 | Lysosome-associated membrane glycoprotein 2 | LAMP2 | 7.07 | 3 | | 44.9 | | 5.63 | | 0.28 | 1 | | 0.48 | | 2 | | 0.00 | | 0 | | 0.00 | | 0 | | 0.65 | | 2 | | 1.41 | | 4 | | 0.93 | | 3 | | 3.46 | | 6 | | 2 | | | 4 | | 6 | |  |  |  |  |
| P31939 | Bifunctional purine biosynthesis protein PURH | ATIC | 21.96 | 8 | | 64.6 | | 6.71 | | 0.15 | 1 | | 0.13 | | 1 | | 0.00 | | 0 | | 0.00 | | 0 | | 1.83 | | 3 | | 1.27 | | 2 | | 0.46 | | 2 | | 0.30 | | 1 | | 2 | | | 4 | | 6 | |  |  |  |  |
| A3KN83 | Protein strawberry notch homolog 1 | SBNO1 | 5.67 | 4 | | 154.2 | | 7.88 | | 0.16 | 1 | | 0.16 | | 1 | | 0.00 | | 0 | | 0.00 | | 0 | | 0.32 | | 1 | | 0.55 | | 2 | | 0.15 | | 1 | | 0.48 | | 3 | | 2 | | | 4 | | 6 | |  |  |  |  |
| Q9Y613 | FH1/FH2 domain-containing protein 1 | FHOD1 | 0.95 | 1 | | 126.5 | | 6.39 | | 0.13 | 1 | | 0.14 | | 1 | | 0.00 | | 0 | | 0.00 | | 0 | | 0.16 | | 1 | | 0.42 | | 2 | | 0.15 | | 1 | | 0.31 | | 2 | | 2 | | | 4 | | 6 | |  |  |  |  |
| P57678 | Gem-associated protein 4 | GEMIN4 | 1.04 | 1 | | 120.0 | | 6.04 | | 0.00 | 0 | | 0.56 | | 3 | | 0.63 | | 2 | | 0.00 | | 0 | | 0.16 | | 1 | | 0.16 | | 1 | | 0.17 | | 1 | | 0.45 | | 3 | | 2 | | | 4 | | 6 | |  |  |  |  |
| Q96AC1 | Fermitin family homolog 2 | FERMT2 | 17.21 | 8 | | 77.8 | | 6.70 | | 0.00 | 0 | | 0.00 | | 0 | | 0.47 | | 2 | | 0.33 | | 1 | | 0.32 | | 2 | | 0.84 | | 3 | | 6.73 | | 6 | | 7.40 | | 6 | | 2 | | | 4 | | 6 | |  |  |  |  |
| P43686 | 26S proteasome regulatory subunit 6B | PSMC4 | 50.00 | 14 | | 47.3 | | 5.21 | | 0.00 | 0 | | 0.00 | | 0 | | 0.22 | | 1 | | 0.16 | | 1 | | 6.49 | | 3 | | 3.01 | | 2 | | 7.40 | | 6 | | 3.67 | | 6 | | 2 | | | 4 | | 6 | |  |  |  |  |
| P30153 | Serine/threonine-protein phosphatase 2A 65 kDa regulatory subunit A alpha isoform | PPP2R1A | 10.36 | 5 | | 65.3 | | 5.11 | | 0.00 | 0 | | 0.13 | | 1 | | 0.19 | | 1 | | 0.00 | | 0 | | 0.85 | | 3 | | 0.79 | | 2 | | 1.81 | | 6 | | 0.76 | | 2 | | 2 | | | 4 | | 6 | |  |  |  |  |
| P53396 | ATP-citrate synthase | ACLY | 21.34 | 17 | | 120.8 | | 7.33 | | 0.00 | 0 | | 0.73 | | 3 | | 0.00 | | 0 | | 0.50 | | 2 | | 5.08 | | 3 | | 4.71 | | 4 | | 9.01 | | 6 | | 8.25 | | 6 | | 2 | | | 4 | | 6 | |  |  |  |  |
| O75531 | Barrier-to-autointegration factor | BANF1 | 29.21 | 2 | | 10.1 | | 6.09 | | 0.00 | 0 | | 0.55 | | 2 | | 0.00 | | 0 | | 0.16 | | 1 | | 0.99 | | 3 | | 0.16 | | 1 | | 1.78 | | 4 | | 1.68 | | 4 | | 2 | | | 4 | | 6 | |  |  |  |  |
| Q15645 | Pachytene checkpoint protein 2 homolog | TRIP13 | 4.17 | 2 | | 48.5 | | 6.09 | | 0.13 | 1 | | 0.00 | | 0 | | 0.19 | | 1 | | 0.00 | | 0 | | 0.78 | | 2 | | 0.42 | | 2 | | 0.59 | | 3 | | 0.00 | | 0 | | 2 | | | 3 | | 5 | |  |  |  |  |
| B1AK88 | F-actin-capping protein subunit beta | CAPZB | 6.31 | 2 | | 33.8 | | 6.43 | | 0.13 | 1 | | 0.00 | | 0 | | 0.16 | | 1 | | 0.00 | | 0 | | 0.42 | | 2 | | 0.48 | | 2 | | 0.29 | | 2 | | 0.00 | | 0 | | 2 | | | 3 | | 5 | |  |  |  |  |
| E9PAV3 | Nascent polypeptide-associated complex subunit alpha, muscle-specific form | NACA | 2.74 | 4 | | 205.3 | | 9.58 | | 0.16 | 1 | | 0.40 | | 2 | | 0.00 | | 0 | | 0.00 | | 0 | | 1.55 | | 3 | | 0.00 | | 0 | | 0.98 | | 3 | | 1.12 | | 3 | | 2 | | | 3 | | 5 | |  |  |  |  |
| Q9UQ80 | Proliferation-associated protein 2G4 | PA2G4 | 9.14 | 3 | | 43.8 | | 6.55 | | 0.13 | 1 | | 0.40 | | 3 | | 0.00 | | 0 | | 0.00 | | 0 | | 0.85 | | 3 | | 0.32 | | 2 | | 0.14 | | 1 | | 0.00 | | 0 | | 2 | | | 3 | | 5 | |  |  |  |  |
| Q14126 | Desmoglein-2 | DSG2 | 10.73 | 6 | | 122.2 | | 5.24 | | 0.13 | 1 | | 0.16 | | 1 | | 0.00 | | 0 | | 0.00 | | 0 | | 1.45 | | 2 | | 1.27 | | 2 | | 0.00 | | 0 | | 0.31 | | 1 | | 2 | | | 3 | | 5 | |  |  |  |  |
| A0A0A0MTH3 | Integrin-linked protein kinase | ILK | 11.59 | 4 | | 54.6 | | 7.97 | | 0.00 | 0 | | 0.14 | | 1 | | 0.76 | | 4 | | 0.00 | | 0 | | 0.56 | | 3 | | 0.00 | | 0 | | 0.46 | | 3 | | 0.15 | | 1 | | 2 | | | 3 | | 5 | |  |  |  |  |
| P05997 | Collagen alpha-2(V) chain | COL5A2 | 4.20 | 1 | | 144.8 | | 6.46 | | 0.00 | 0 | | 0.32 | | 1 | | 0.63 | | 2 | | 0.00 | | 0 | | 0.00 | | 0 | | 2.03 | | 3 | | 1.61 | | 4 | | 1.20 | | 3 | | 2 | | | 3 | | 5 | |  |  |  |  |
| P04792 | Heat shock protein beta-1 | HSPB1 | 33.17 | 5 | | 22.8 | | 6.40 | | 0.00 | 0 | | 0.00 | | 0 | | 0.19 | | 1 | | 0.64 | | 4 | | 0.49 | | 2 | | 0.00 | | 0 | | 1.47 | | 4 | | 2.31 | | 5 | | 2 | | | 3 | | 5 | |  |  |  |  |
| Q8NF91 | Nesprin-1 | SYNE1 | 0.47 | 4 | | 1010.5 | | 5.53 | | 0.00 | 0 | | 0.41 | | 2 | | 0.00 | | 0 | | 0.32 | | 2 | | 0.00 | | 0 | | 0.34 | | 2 | | 0.75 | | 4 | | 0.15 | | 1 | | 2 | | | 3 | | 5 | |  |  |  |  |
| A0A1C7CYX9 | Dihydropyrimidinase-related protein 2 | DPYSL2 | 15.51 | 5 | | 73.5 | | 6.35 | | 0.00 | 0 | | 0.14 | | 1 | | 0.00 | | 0 | | 0.16 | | 1 | | 0.00 | | 0 | | 1.34 | | 3 | | 2.58 | | 6 | | 1.78 | | 6 | | 2 | | | 3 | | 5 | |  |  |  |  |
| P05455 | Lupus La protein | SSB | 20.34 | 6 | | 46.8 | | 7.12 | | 1.78 | 4 | | 2.02 | | 4 | | 0.00 | | 0 | | 0.00 | | 0 | | 0.58 | | 3 | | 0.00 | | 0 | | 0.58 | | 2 | | 0.00 | | 0 | | 2 | | | 2 | | 4 | |  |  |  |  |
| Q86UP2 | Kinectin | KTN1 | 2.87 | 2 | | 156.2 | | 5.64 | | 0.32 | 2 | | 0.27 | | 2 | | 0.00 | | 0 | | 0.00 | | 0 | | 0.56 | | 3 | | 0.00 | | 0 | | 0.17 | | 1 | | 0.00 | | 0 | | 2 | | | 2 | | 4 | |  |  |  |  |
| P31431 | Syndecan-4 | SDC4 | 24.24 | 4 | | 21.6 | | 4.50 | | 1.03 | 2 | | 0.64 | | 2 | | 0.00 | | 0 | | 0.00 | | 0 | | 0.32 | | 1 | | 0.73 | | 2 | | 0.00 | | 0 | | 0.00 | | 0 | | 2 | | | 2 | | 4 | |  |  |  |  |
| Q9UPQ9 | Trinucleotide repeat-containing gene 6B protein | TNRC6B | 1.85 | 2 | | 193.9 | | 6.76 | | 0.28 | 1 | | 0.00 | | 0 | | 0.00 | | 0 | | 0.16 | | 1 | | 0.00 | | 0 | | 0.16 | | 1 | | 0.00 | | 0 | | 0.15 | | 1 | | 2 | | | 2 | | 4 | |  |  |  |  |
| P68402 | Platelet-activating factor acetylhydrolase IB subunit beta | PAFAH1B2 | 12.23 | 2 | | 25.6 | | 5.92 | | 0.15 | 1 | | 0.41 | | 3 | | 0.00 | | 0 | | 0.00 | | 0 | | 0.28 | | 1 | | 0.00 | | 0 | | 0.14 | | 1 | | 0.00 | | 0 | | 2 | | | 2 | | 4 | |  |  |  |  |
| Q96N16 | Janus kinase and microtubule-interacting protein 1 | JAKMIP1 | 7.67 | 3 | | 73.2 | | 6.10 | | 0.15 | 1 | | 0.14 | | 1 | | 0.00 | | 0 | | 0.00 | | 0 | | 0.00 | | 0 | | 0.00 | | 0 | | 0.14 | | 1 | | 0.48 | | 3 | | 2 | | | 2 | | 4 | |  |  |  |  |
| P12004 | Proliferating cell nuclear antigen | PCNA | 5.75 | 2 | | 28.8 | | 4.69 | | 0.15 | 1 | | 0.55 | | 2 | | 0.00 | | 0 | | 0.00 | | 0 | | 0.00 | | 0 | | 0.16 | | 1 | | 0.14 | | 1 | | 0.00 | | 0 | | 2 | | | 2 | | 4 | |  |  |  |  |
| P52907 | F-actin-capping protein subunit alpha-1 | CAPZA1 | 11.54 | 2 | | 32.9 | | 5.69 | | 0.31 | 1 | | 0.27 | | 2 | | 0.00 | | 0 | | 0.00 | | 0 | | 0.14 | | 1 | | 0.00 | | 0 | | 0.14 | | 1 | | 0.00 | | 0 | | 2 | | | 2 | | 4 | |  |  |  |  |
| Q9ULC6 | Protein-arginine deiminase type-1 | PADI1 | 1.36 | 1 | | 74.6 | | 6.49 | | 0.00 | 0 | | 0.14 | | 1 | | 0.31 | | 1 | | 0.00 | | 0 | | 0.14 | | 1 | | 0.00 | | 0 | | 0.00 | | 0 | | 0.15 | | 1 | | 2 | | | 2 | | 4 | |  |  |  |  |
| F8VR36 | Natural resistance-associated macrophage protein 2 (Fragment) | SLC11A2 | 6.54 | 1 | | 16.8 | | 4.37 | | 0.00 | 0 | | 0.00 | | 0 | | 0.16 | | 1 | | 0.37 | | 2 | | 0.00 | | 0 | | 0.18 | | 1 | | 0.15 | | 1 | | 0.00 | | 0 | | 2 | | | 2 | | 4 | |  |  |  |  |
| Q9UHD8 | Septin-9 | SEPTIN9 | 9.22 | 4 | | 65.4 | | 8.97 | | 0.00 | 0 | | 0.14 | | 1 | | 0.16 | | 1 | | 0.00 | | 0 | | 0.28 | | 1 | | 0.32 | | 2 | | 0.00 | | 0 | | 0.00 | | 0 | | 2 | | | 2 | | 4 | |  |  |  |  |
| Q14697 | Neutral alpha-glucosidase AB | GANAB | 16.84 | 11 | | 106.8 | | 6.14 | | 0.00 | 0 | | 0.14 | | 1 | | 0.00 | | 0 | | 0.47 | | 2 | | 0.00 | | 0 | | 0.47 | | 1 | | 0.00 | | 0 | | 2.43 | | 2 | | 2 | | | 2 | | 4 | |  |  |  |  |
| P46939 | Utrophin | UTRN | 1.49 | 4 | | 394.2 | | 5.33 | | 0.00 | 0 | | 0.27 | | 1 | | 0.00 | | 0 | | 0.21 | | 1 | | 0.14 | | 1 | | 0.00 | | 0 | | 0.00 | | 0 | | 0.15 | | 1 | | 2 | | | 2 | | 4 | |  |  |  |  |
| E9PSI1 | Transmembrane 9 superfamily member |  | 2.33 | 1 | | 92.3 | | 6.23 | | 0.00 | 0 | | 0.16 | | 1 | | 0.00 | | 0 | | 0.17 | | 1 | | 0.00 | | 0 | | 0.24 | | 1 | | 0.32 | | 2 | | 0.00 | | 0 | | 2 | | | 2 | | 4 | |  |  |  |  |
| A8MPS7 | Carbohydrate deacetylase | YDJC | 2.48 | 1 | | 34.4 | | 6.39 | | 0.00 | 0 | | 0.14 | | 1 | | 0.00 | | 0 | | 0.16 | | 1 | | 0.14 | | 1 | | 0.00 | | 0 | | 0.14 | | 1 | | 0.00 | | 0 | | 2 | | | 2 | | 4 | |  |  |  |  |
| C9JG08 | Uncharacterized protein C2orf16 | C2orf16 | 1.04 | 3 | | 598.1 | | 9.10 | | 0.00 | 0 | | 0.16 | | 1 | | 0.00 | | 0 | | 0.16 | | 1 | | 0.00 | | 0 | | 0.18 | | 1 | | 0.15 | | 1 | | 0.00 | | 0 | | 2 | | | 2 | | 4 | |  |  |  |  |
| Q8IUG5 | Unconventional myosin-XVIIIb | MYO18B | 0.58 | 1 | | 285.0 | | 6.86 | | 0.00 | 0 | | 0.40 | | 2 | | 0.00 | | 0 | | 0.16 | | 1 | | 0.14 | | 1 | | 0.00 | | 0 | | 0.31 | | 2 | | 0.00 | | 0 | | 2 | | | 2 | | 4 | |  |  |  |  |
| P08123 | Collagen alpha-2(I) chain | COL1A2 | 2.49 | 3 | | 129.2 | | 8.95 | | 0.39 | 2 | | 0.40 | | 2 | | 0.00 | | 0 | | 0.00 | | 0 | | 0.62 | | 1 | | 0.00 | | 0 | | 0.00 | | 0 | | 0.00 | | 0 | | 2 | | | 1 | | 3 | |  |  |  |  |
| H0YMP1 | Deoxyuridine 5'-triphosphate nucleotidohydrolase, mitochondrial (Fragment) | DUT | 29.91 | 1 | | 11.9 | | 5.07 | | 0.51 | 2 | | 0.14 | | 1 | | 0.00 | | 0 | | 0.00 | | 0 | | 0.00 | | 0 | | 0.00 | | 0 | | 0.15 | | 1 | | 0.00 | | 0 | | 2 | | | 1 | | 3 | |  |  |  |  |
| O75112 | LIM domain-binding protein 3 | LDB3 | 7.02 | 2 | | 77.1 | | 8.13 | | 0.48 | 2 | | 0.14 | | 1 | | 0.00 | | 0 | | 0.00 | | 0 | | 0.00 | | 0 | | 0.00 | | 0 | | 0.00 | | 0 | | 0.18 | | 1 | | 2 | | | 1 | | 3 | |  |  |  |  |
| P48058 | Glutamate receptor 4 | GRIA4 | 5.43 | 1 | | 100.8 | | 8.10 | | 0.28 | 1 | | 0.00 | | 0 | | 0.19 | | 1 | | 0.00 | | 0 | | 0.00 | | 0 | | 0.34 | | 2 | | 0.00 | | 0 | | 0.00 | | 0 | | 2 | | | 1 | | 3 | |  |  |  |  |
| Q4G0P3 | Hydrocephalus-inducing protein homolog | HYDIN | 0.92 | 2 | | 575.5 | | 6.06 | | 0.13 | 1 | | 0.00 | | 0 | | 0.19 | | 1 | | 0.00 | | 0 | | 0.14 | | 1 | | 0.00 | | 0 | | 0.00 | | 0 | | 0.00 | | 0 | | 2 | | | 1 | | 3 | |  |  |  |  |
| Q8IX21 | SMC5-SMC6 complex localization factor protein 2 | SLF2 | 1.45 | 1 | | 131.8 | | 8.97 | | 0.16 | 1 | | 0.00 | | 0 | | 0.00 | | 0 | | 0.16 | | 1 | | 0.00 | | 0 | | 0.18 | | 1 | | 0.00 | | 0 | | 0.00 | | 0 | | 2 | | | 1 | | 3 | |  |  |  |  |
| A0A087X250 | Protocadherin-15 | PCDH15 | 5.45 | 3 | | 217.0 | | 5.05 | | 0.13 | 1 | | 0.00 | | 0 | | 0.00 | | 0 | | 0.16 | | 1 | | 0.00 | | 0 | | 0.00 | | 0 | | 0.17 | | 1 | | 0.00 | | 0 | | 2 | | | 1 | | 3 | |  |  |  |  |
| O15144 | Actin-related protein 2/3 complex subunit 2 | ARPC2 | 21.33 | 4 | | 34.3 | | 7.36 | | 0.33 | 1 | | 0.41 | | 2 | | 0.00 | | 0 | | 0.00 | | 0 | | 0.00 | | 0 | | 0.00 | | 0 | | 0.00 | | 0 | | 0.46 | | 3 | | 2 | | | 1 | | 3 | |  |  |  |  |
| Q04446 | 1,4-alpha-glucan-branching enzyme | GBE1 | 11.54 | 3 | | 80.4 | | 6.32 | | 0.13 | 1 | | 0.16 | | 1 | | 0.00 | | 0 | | 0.00 | | 0 | | 0.00 | | 0 | | 0.00 | | 0 | | 0.00 | | 0 | | 0.91 | | 3 | | 2 | | | 1 | | 3 | |  |  |  |  |
| P48163 | NADP-dependent malic enzyme | ME1 | 11.71 | 3 | | 64.1 | | 6.13 | | 0.13 | 1 | | 0.81 | | 2 | | 0.00 | | 0 | | 0.00 | | 0 | | 0.00 | | 0 | | 0.00 | | 0 | | 0.15 | | 1 | | 0.00 | | 0 | | 2 | | | 1 | | 3 | |  |  |  |  |
| Q63HM1 | Kynurenine formamidase | AFMID | 12.21 | 2 | | 34.0 | | 5.91 | | 0.13 | 1 | | 0.13 | | 1 | | 0.00 | | 0 | | 0.00 | | 0 | | 0.00 | | 0 | | 0.16 | | 1 | | 0.00 | | 0 | | 0.00 | | 0 | | 2 | | | 1 | | 3 | |  |  |  |  |
| Q04759 | Protein kinase C theta type | PRKCQ | 6.80 | 3 | | 81.8 | | 7.61 | | 0.13 | 1 | | 0.14 | | 1 | | 0.00 | | 0 | | 0.00 | | 0 | | 0.00 | | 0 | | 0.00 | | 0 | | 0.15 | | 1 | | 0.00 | | 0 | | 2 | | | 1 | | 3 | |  |  |  |  |
| Q13206 | Probable ATP-dependent RNA helicase DDX10 | DDX10 | 3.77 | 2 | | 100.8 | | 8.63 | | 0.28 | 1 | | 0.16 | | 1 | | 0.00 | | 0 | | 0.00 | | 0 | | 0.00 | | 0 | | 0.00 | | 0 | | 0.00 | | 0 | | 0.15 | | 1 | | 2 | | | 1 | | 3 | |  |  |  |  |
| P23142 | Fibulin-1 | FBLN1 | 4.98 | 2 | | 77.2 | | 5.22 | | 0.00 | 0 | | 0.00 | | 0 | | 0.67 | | 2 | | 0.21 | | 1 | | 0.00 | | 0 | | 0.16 | | 1 | | 0.00 | | 0 | | 0.00 | | 0 | | 2 | | | 1 | | 3 | |  |  |  |  |
| P32455 | Guanylate-binding protein 1 | GBP1 | 1.35 | 1 | | 67.9 | | 6.32 | | 0.00 | 0 | | 0.00 | | 0 | | 0.34 | | 2 | | 0.16 | | 1 | | 0.14 | | 1 | | 0.00 | | 0 | | 0.00 | | 0 | | 0.00 | | 0 | | 2 | | | 1 | | 3 | |  |  |  |  |
| P04114 | Apolipoprotein B-100 | APOB | 0.81 | 3 | | 515.3 | | 7.05 | | 0.00 | 0 | | 0.00 | | 0 | | 0.22 | | 1 | | 0.16 | | 1 | | 0.00 | | 0 | | 0.00 | | 0 | | 0.17 | | 1 | | 0.00 | | 0 | | 2 | | | 1 | | 3 | |  |  |  |  |
| Q6UVM3 | Potassium channel subfamily T member 2 | KCNT2 | 2.91 | 2 | | 130.4 | | 7.28 | | 0.00 | 0 | | 0.14 | | 1 | | 0.19 | | 1 | | 0.00 | | 0 | | 0.14 | | 1 | | 0.00 | | 0 | | 0.00 | | 0 | | 0.00 | | 0 | | 2 | | | 1 | | 3 | |  |  |  |  |
| Q8NDV7 | Trinucleotide repeat-containing gene 6A protein | TNRC6A | 4.38 | 3 | | 210.2 | | 7.01 | | 0.00 | 0 | | 0.13 | | 1 | | 0.19 | | 1 | | 0.00 | | 0 | | 0.00 | | 0 | | 0.16 | | 1 | | 0.00 | | 0 | | 0.00 | | 0 | | 2 | | | 1 | | 3 | |  |  |  |  |
| Q8NA31 | Telomere repeats-binding bouquet formation protein 1 | TERB1 | 6.60 | 2 | | 83.0 | | 7.46 | | 0.00 | 0 | | 0.13 | | 1 | | 0.19 | | 1 | | 0.00 | | 0 | | 0.00 | | 0 | | 0.00 | | 0 | | 0.15 | | 1 | | 0.00 | | 0 | | 2 | | | 1 | | 3 | |  |  |  |  |
| P01040 | Cystatin-A | CSTA | 50.00 | 3 | | 11.0 | | 5.50 | | 0.00 | 0 | | 0.16 | | 1 | | 0.19 | | 1 | | 0.00 | | 0 | | 0.00 | | 0 | | 0.70 | | 2 | | 0.00 | | 0 | | 0.00 | | 0 | | 2 | | | 1 | | 3 | |  |  |  |  |
| Q8NBS9 | Thioredoxin domain-containing protein 5 | TXNDC5 | 12.73 | 4 | | 47.6 | | 5.97 | | 0.00 | 0 | | 1.26 | | 3 | | 0.00 | | 0 | | 1.11 | | 4 | | 0.00 | | 0 | | 0.00 | | 0 | | 0.00 | | 0 | | 0.15 | | 1 | | 2 | | | 1 | | 3 | |  |  |  |  |
| Q9H361 | Polyadenylate-binding protein 3 | PABPC3 | 10.46 | 1 | | 70.0 | | 9.67 | | 0.00 | 0 | | 0.14 | | 1 | | 0.00 | | 0 | | 0.38 | | 2 | | 0.99 | | 3 | | 0.00 | | 0 | | 0.00 | | 0 | | 0.00 | | 0 | | 2 | | | 1 | | 3 | |  |  |  |  |
| Q8NFC6 | Biorientation of chromosomes in cell division protein 1-like 1 | BOD1L1 | 2.79 | 3 | | 330.3 | | 5.08 | | 0.00 | 0 | | 0.16 | | 1 | | 0.00 | | 0 | | 0.21 | | 1 | | 0.00 | | 0 | | 0.00 | | 0 | | 0.15 | | 1 | | 0.00 | | 0 | | 2 | | | 1 | | 3 | |  |  |  |  |
| Q9UEW8 | STE20/SPS1-related proline-alanine-rich protein kinase | STK39 | 6.97 | 2 | | 59.4 | | 6.29 | | 0.00 | 0 | | 0.16 | | 1 | | 0.00 | | 0 | | 0.16 | | 1 | | 0.14 | | 1 | | 0.00 | | 0 | | 0.00 | | 0 | | 0.00 | | 0 | | 2 | | | 1 | | 3 | |  |  |  |  |
| Q8WXH0 | Nesprin-2 | SYNE2 | 0.80 | 3 | | 795.9 | | 5.36 | | 0.00 | 0 | | 0.14 | | 1 | | 0.00 | | 0 | | 0.16 | | 1 | | 0.00 | | 0 | | 0.00 | | 0 | | 0.00 | | 0 | | 0.15 | | 1 | | 2 | | | 1 | | 3 | |  |  |  |  |
| Q75MW2 | Protein ZNF767 | ZNF767P | 7.10 | 1 | | 17.2 | | 9.64 | | 0.00 | 0 | | 0.13 | | 1 | | 0.00 | | 0 | | 0.16 | | 1 | | 0.00 | | 0 | | 0.00 | | 0 | | 0.00 | | 0 | | 0.15 | | 1 | | 2 | | | 1 | | 3 | |  |  |  |  |
| P14625 | Endoplasmin | HSP90B1 | 3.99 | 2 | | 92.4 | | 4.84 | | 0.00 | 0 | | 0.67 | | 2 | | 0.00 | | 0 | | 0.16 | | 1 | | 0.00 | | 0 | | 0.46 | | 1 | | 0.00 | | 0 | | 0.00 | | 0 | | 2 | | | 1 | | 3 | |  |  |  |  |
| P02461 | Collagen alpha-1(III) chain | COL3A1 | 1.16 | 1 | | 138.5 | | 6.61 | | 0.00 | 0 | | 0.32 | | 1 | | 0.00 | | 0 | | 0.16 | | 1 | | 0.00 | | 0 | | 0.47 | | 1 | | 0.00 | | 0 | | 0.00 | | 0 | | 2 | | | 1 | | 3 | |  |  |  |  |
| A8MW92 | PHD finger protein 20-like protein 1 | PHF20L1 | 3.54 | 2 | | 114.9 | | 6.83 | | 0.00 | 0 | | 0.14 | | 1 | | 0.00 | | 0 | | 0.16 | | 1 | | 0.16 | | 1 | | 0.00 | | 0 | | 0.00 | | 0 | | 0.00 | | 0 | | 2 | | | 1 | | 3 | |  |  |  |  |
| A4D0S4 | Laminin subunit beta-4 | LAMB4 | 4.77 | 3 | | 193.4 | | 6.35 | | 0.00 | 0 | | 0.14 | | 1 | | 0.00 | | 0 | | 0.16 | | 1 | | 0.14 | | 1 | | 0.00 | | 0 | | 0.00 | | 0 | | 0.00 | | 0 | | 2 | | | 1 | | 3 | |  |  |  |  |
| E5RH11 | Heparan-alpha-glucosaminide N-acetyltransferase | HGSNAT | 39.18 | 1 | | 10.7 | | 8.94 | | 0.00 | 0 | | 0.14 | | 1 | | 0.00 | | 0 | | 0.16 | | 1 | | 0.00 | | 0 | | 0.00 | | 0 | | 0.15 | | 1 | | 0.00 | | 0 | | 2 | | | 1 | | 3 | |  |  |  |  |
| P78363 | Retinal-specific phospholipid-transporting ATPase ABCA4 | ABCA4 | 4.49 | 3 | | 255.8 | | 6.29 | | 0.00 | 0 | | 0.14 | | 1 | | 0.00 | | 0 | | 0.16 | | 1 | | 0.00 | | 0 | | 0.00 | | 0 | | 0.14 | | 1 | | 0.00 | | 0 | | 2 | | | 1 | | 3 | |  |  |  |  |
| P05067 | Amyloid-beta precursor protein | APP | 7.27 | 5 | | 86.9 | | 4.82 | | 1.67 | 5 | | 2.59 | | 4 | | 0.00 | | 0 | | 0.00 | | 0 | | 0.00 | | 0 | | 0.00 | | 0 | | 0.00 | | 0 | | 0.00 | | 0 | | 2 | | | 0 | | 2 | |  |  |  |  |
| Q658N2 | WSC domain-containing protein 1 | WSCD1 | 3.83 | 2 | | 65.7 | | 9.16 | | 0.55 | 3 | | 0.00 | | 0 | | 0.53 | | 2 | | 0.00 | | 0 | | 0.00 | | 0 | | 0.00 | | 0 | | 0.00 | | 0 | | 0.00 | | 0 | | 2 | | | 0 | | 2 | |  |  |  |  |
| P14550 | Aldo-keto reductase family 1 member A1 | AKR1A1 | 6.46 | 1 | | 36.6 | | 6.79 | | 0.41 | 3 | | 0.68 | | 4 | | 0.00 | | 0 | | 0.00 | | 0 | | 0.00 | | 0 | | 0.00 | | 0 | | 0.00 | | 0 | | 0.00 | | 0 | | 2 | | | 0 | | 2 | |  |  |  |  |
| Q09028 | Histone-binding protein RBBP4 | RBBP4 | 1.88 | 1 | | 47.6 | | 4.89 | | 0.42 | 3 | | 0.55 | | 2 | | 0.00 | | 0 | | 0.00 | | 0 | | 0.00 | | 0 | | 0.00 | | 0 | | 0.00 | | 0 | | 0.00 | | 0 | | 2 | | | 0 | | 2 | |  |  |  |  |
| M0R0P8 | Unconventional myosin-IXb | MYO9B | 0.42 | 1 | | 243.2 | | 8.78 | | 0.47 | 2 | | 0.00 | | 0 | | 0.69 | | 2 | | 0.00 | | 0 | | 0.00 | | 0 | | 0.00 | | 0 | | 0.00 | | 0 | | 0.00 | | 0 | | 2 | | | 0 | | 2 | |  |  |  |  |
| B0YIW6 | Coatomer subunit delta | ARCN1 | 1.99 | 1 | | 61.6 | | 5.85 | | 0.56 | 2 | | 0.00 | | 0 | | 0.31 | | 1 | | 0.00 | | 0 | | 0.00 | | 0 | | 0.00 | | 0 | | 0.00 | | 0 | | 0.00 | | 0 | | 2 | | | 0 | | 2 | |  |  |  |  |
| P13497 | Bone morphogenetic protein 1 | BMP1 | 2.74 | 2 | | 111.2 | | 6.90 | | 0.39 | 2 | | 0.00 | | 0 | | 0.22 | | 1 | | 0.00 | | 0 | | 0.00 | | 0 | | 0.00 | | 0 | | 0.00 | | 0 | | 0.00 | | 0 | | 2 | | | 0 | | 2 | |  |  |  |  |
| H0Y7Z4 | Transcription factor RFX3 (Fragment) | RFX3 | 8.33 | 1 | | 16.7 | | 7.14 | | 0.29 | 2 | | 0.00 | | 0 | | 0.19 | | 1 | | 0.00 | | 0 | | 0.00 | | 0 | | 0.00 | | 0 | | 0.00 | | 0 | | 0.00 | | 0 | | 2 | | | 0 | | 2 | |  |  |  |  |
| P61970 | Nuclear transport factor 2 | NUTF2 | 6.30 | 1 | | 14.5 | | 5.38 | | 0.59 | 2 | | 0.00 | | 0 | | 0.16 | | 1 | | 0.00 | | 0 | | 0.00 | | 0 | | 0.00 | | 0 | | 0.00 | | 0 | | 0.00 | | 0 | | 2 | | | 0 | | 2 | |  |  |  |  |
| H0YH15 | Aquarius homolog (Mouse), isoform CRA_a | AQR | 2.32 | 1 | | 75.8 | | 6.25 | | 0.32 | 2 | | 0.00 | | 0 | | 0.16 | | 1 | | 0.00 | | 0 | | 0.00 | | 0 | | 0.00 | | 0 | | 0.00 | | 0 | | 0.00 | | 0 | | 2 | | | 0 | | 2 | |  |  |  |  |
| Q86YC2 | Partner and localizer of BRCA2 | PALB2 | 2.70 | 2 | | 131.2 | | 6.44 | | 0.48 | 2 | | 0.00 | | 0 | | 0.00 | | 0 | | 0.16 | | 1 | | 0.00 | | 0 | | 0.00 | | 0 | | 0.00 | | 0 | | 0.00 | | 0 | | 2 | | | 0 | | 2 | |  |  |  |  |
| Q13740 | CD166 antigen | ALCAM | 8.58 | 4 | | 65.1 | | 6.25 | | 1.03 | 2 | | 0.43 | | 3 | | 0.00 | | 0 | | 0.00 | | 0 | | 0.00 | | 0 | | 0.00 | | 0 | | 0.00 | | 0 | | 0.00 | | 0 | | 2 | | | 0 | | 2 | |  |  |  |  |
| P09237 | Matrilysin | MMP7 | 11.61 | 3 | | 29.7 | | 7.91 | | 1.13 | 2 | | 1.77 | | 2 | | 0.00 | | 0 | | 0.00 | | 0 | | 0.00 | | 0 | | 0.00 | | 0 | | 0.00 | | 0 | | 0.00 | | 0 | | 2 | | | 0 | | 2 | |  |  |  |  |
| H0YNJ9 | Deoxyuridine 5'-triphosphate nucleotidohydrolase, mitochondrial | DUT | 32.87 | 2 | | 15.5 | | 7.90 | | 0.64 | 2 | | 0.27 | | 1 | | 0.00 | | 0 | | 0.00 | | 0 | | 0.00 | | 0 | | 0.00 | | 0 | | 0.00 | | 0 | | 0.00 | | 0 | | 2 | | | 0 | | 2 | |  |  |  |  |
| P61457 | Pterin-4-alpha-carbinolamine dehydratase | PCBD1 | 15.38 | 1 | | 12.0 | | 6.80 | | 0.29 | 2 | | 0.27 | | 1 | | 0.00 | | 0 | | 0.00 | | 0 | | 0.00 | | 0 | | 0.00 | | 0 | | 0.00 | | 0 | | 0.00 | | 0 | | 2 | | | 0 | | 2 | |  |  |  |  |
| Q12849 | G-rich sequence factor 1 | GRSF1 | 7.92 | 2 | | 53.1 | | 6.19 | | 0.29 | 2 | | 0.14 | | 1 | | 0.00 | | 0 | | 0.00 | | 0 | | 0.00 | | 0 | | 0.00 | | 0 | | 0.00 | | 0 | | 0.00 | | 0 | | 2 | | | 0 | | 2 | |  |  |  |  |
| O94955 | Rho-related BTB domain-containing protein 3 | RHOBTB3 | 1.96 | 1 | | 69.4 | | 7.62 | | 0.29 | 2 | | 0.13 | | 1 | | 0.00 | | 0 | | 0.00 | | 0 | | 0.00 | | 0 | | 0.00 | | 0 | | 0.00 | | 0 | | 0.00 | | 0 | | 2 | | | 0 | | 2 | |  |  |  |  |
| P01133 | Pro-epidermal growth factor | EGF | 5.22 | 2 | | 133.9 | | 5.85 | | 0.29 | 2 | | 0.13 | | 1 | | 0.00 | | 0 | | 0.00 | | 0 | | 0.00 | | 0 | | 0.00 | | 0 | | 0.00 | | 0 | | 0.00 | | 0 | | 2 | | | 0 | | 2 | |  |  |  |  |
| Q99633 | Pre-mRNA-splicing factor 18 | PRPF18 | 2.34 | 1 | | 39.8 | | 8.15 | | 0.28 | 2 | | 0.14 | | 1 | | 0.00 | | 0 | | 0.00 | | 0 | | 0.00 | | 0 | | 0.00 | | 0 | | 0.00 | | 0 | | 0.00 | | 0 | | 2 | | | 0 | | 2 | |  |  |  |  |
| I3NI02 | Golgi SNAP receptor complex member 2 | GOSR2 | 7.78 | 1 | | 29.8 | | 7.17 | | 0.57 | 2 | | 0.16 | | 1 | | 0.00 | | 0 | | 0.00 | | 0 | | 0.00 | | 0 | | 0.00 | | 0 | | 0.00 | | 0 | | 0.00 | | 0 | | 2 | | | 0 | | 2 | |  |  |  |  |
| Q8N4F0 | BPI fold-containing family B member 2 | BPIFB2 | 3.93 | 1 | | 49.1 | | 8.72 | | 0.28 | 1 | | 0.00 | | 0 | | 0.51 | | 2 | | 0.00 | | 0 | | 0.00 | | 0 | | 0.00 | | 0 | | 0.00 | | 0 | | 0.00 | | 0 | | 2 | | | 0 | | 2 | |  |  |  |  |
| E5RFV4 | Zinc finger protein 395 (Fragment) | ZNF395 | 15.51 | 1 | | 20.1 | | 5.01 | | 0.13 | 1 | | 0.00 | | 0 | | 0.34 | | 2 | | 0.00 | | 0 | | 0.00 | | 0 | | 0.00 | | 0 | | 0.00 | | 0 | | 0.00 | | 0 | | 2 | | | 0 | | 2 | |  |  |  |  |
| Q96MG7 | Non-structural maintenance of chromosomes element 3 homolog | NSMCE3 | 2.30 | 1 | | 34.3 | | 9.28 | | 0.13 | 1 | | 0.00 | | 0 | | 0.32 | | 1 | | 0.00 | | 0 | | 0.00 | | 0 | | 0.00 | | 0 | | 0.00 | | 0 | | 0.00 | | 0 | | 2 | | | 0 | | 2 | |  |  |  |  |
| Q96P20 | NACHT, LRR and PYD domains-containing protein 3 | NLRP3 | 4.05 | 2 | | 118.1 | | 6.65 | | 0.15 | 1 | | 0.00 | | 0 | | 0.22 | | 1 | | 0.00 | | 0 | | 0.00 | | 0 | | 0.00 | | 0 | | 0.00 | | 0 | | 0.00 | | 0 | | 2 | | | 0 | | 2 | |  |  |  |  |
| Q6EMK4 | Vasorin | VASN | 6.54 | 2 | | 71.7 | | 7.39 | | 0.26 | 1 | | 0.00 | | 0 | | 0.22 | | 1 | | 0.00 | | 0 | | 0.00 | | 0 | | 0.00 | | 0 | | 0.00 | | 0 | | 0.00 | | 0 | | 2 | | | 0 | | 2 | |  |  |  |  |
| Q9UIQ6 | Leucyl-cystinyl aminopeptidase | LNPEP | 7.32 | 2 | | 117.3 | | 5.73 | | 0.13 | 1 | | 0.00 | | 0 | | 0.22 | | 1 | | 0.00 | | 0 | | 0.00 | | 0 | | 0.00 | | 0 | | 0.00 | | 0 | | 0.00 | | 0 | | 2 | | | 0 | | 2 | |  |  |  |  |
| Q9UGM5 | Fetuin-B | FETUB | 8.38 | 1 | | 42.0 | | 6.83 | | 0.15 | 1 | | 0.00 | | 0 | | 0.19 | | 1 | | 0.00 | | 0 | | 0.00 | | 0 | | 0.00 | | 0 | | 0.00 | | 0 | | 0.00 | | 0 | | 2 | | | 0 | | 2 | |  |  |  |  |
| K7ENN9 | Arachidonate 12-lipoxygenase, 12S-type (Fragment) | ALOX12 | 12.24 | 1 | | 16.9 | | 5.11 | | 0.28 | 1 | | 0.00 | | 0 | | 0.19 | | 1 | | 0.00 | | 0 | | 0.00 | | 0 | | 0.00 | | 0 | | 0.00 | | 0 | | 0.00 | | 0 | | 2 | | | 0 | | 2 | |  |  |  |  |
| Q96S21 | Ras-related protein Rab-40C | RAB40C | 2.85 | 1 | | 31.3 | | 9.19 | | 0.57 | 1 | | 0.00 | | 0 | | 0.19 | | 1 | | 0.00 | | 0 | | 0.00 | | 0 | | 0.00 | | 0 | | 0.00 | | 0 | | 0.00 | | 0 | | 2 | | | 0 | | 2 | |  |  |  |  |
| M0QXB4 | Coatomer protein complex, subunit epsilon, isoform CRA_g | COPE | 7.85 | 1 | | 36.9 | | 5.16 | | 0.15 | 1 | | 0.00 | | 0 | | 0.19 | | 1 | | 0.00 | | 0 | | 0.00 | | 0 | | 0.00 | | 0 | | 0.00 | | 0 | | 0.00 | | 0 | | 2 | | | 0 | | 2 | |  |  |  |  |
| A0A087WYK6 | Acetyl-CoA carboxylase 1 | ACACA | 11.40 | 1 | | 12.7 | | 5.19 | | 0.28 | 1 | | 0.00 | | 0 | | 0.19 | | 1 | | 0.00 | | 0 | | 0.00 | | 0 | | 0.00 | | 0 | | 0.00 | | 0 | | 0.00 | | 0 | | 2 | | | 0 | | 2 | |  |  |  |  |
| Q9HBI0 | Gamma-parvin | PARVG | 4.23 | 1 | | 37.5 | | 5.49 | | 0.15 | 1 | | 0.00 | | 0 | | 0.16 | | 1 | | 0.00 | | 0 | | 0.00 | | 0 | | 0.00 | | 0 | | 0.00 | | 0 | | 0.00 | | 0 | | 2 | | | 0 | | 2 | |  |  |  |  |
| Q8N6G6 | ADAMTS-like protein 1 | ADAMTSL1 | 0.68 | 2 | | 193.3 | | 7.80 | | 0.15 | 1 | | 0.00 | | 0 | | 0.16 | | 1 | | 0.00 | | 0 | | 0.00 | | 0 | | 0.00 | | 0 | | 0.00 | | 0 | | 0.00 | | 0 | | 2 | | | 0 | | 2 | |  |  |  |  |
| O00237 | E3 ubiquitin-protein ligase RNF103 | RNF103 | 3.50 | 1 | | 79.4 | | 5.68 | | 0.28 | 1 | | 0.00 | | 0 | | 0.16 | | 1 | | 0.00 | | 0 | | 0.00 | | 0 | | 0.00 | | 0 | | 0.00 | | 0 | | 0.00 | | 0 | | 2 | | | 0 | | 2 | |  |  |  |  |
| O95433 | Activator of 90 kDa heat shock protein ATPase homolog 1 | AHSA1 | 6.51 | 1 | | 38.3 | | 5.53 | | 0.13 | 1 | | 0.00 | | 0 | | 0.16 | | 1 | | 0.00 | | 0 | | 0.00 | | 0 | | 0.00 | | 0 | | 0.00 | | 0 | | 0.00 | | 0 | | 2 | | | 0 | | 2 | |  |  |  |  |
| Q495B1 | Ankyrin repeat and death domain-containing protein 1A | ANKDD1A | 8.81 | 2 | | 57.5 | | 6.71 | | 0.13 | 1 | | 0.00 | | 0 | | 0.16 | | 1 | | 0.00 | | 0 | | 0.00 | | 0 | | 0.00 | | 0 | | 0.00 | | 0 | | 0.00 | | 0 | | 2 | | | 0 | | 2 | |  |  |  |  |
| H0YM30 | Formin-1 | FMN1 | 2.20 | 2 | | 146.4 | | 8.70 | | 0.13 | 1 | | 0.00 | | 0 | | 0.16 | | 1 | | 0.00 | | 0 | | 0.00 | | 0 | | 0.00 | | 0 | | 0.00 | | 0 | | 0.00 | | 0 | | 2 | | | 0 | | 2 | |  |  |  |  |
| H7C3E4 | FERM, ARHGEF and pleckstrin domain-containing protein 2 (Fragment) | FARP2 | 10.13 | 1 | | 9.1 | | 9.41 | | 0.13 | 1 | | 0.00 | | 0 | | 0.00 | | 0 | | 0.53 | | 3 | | 0.00 | | 0 | | 0.00 | | 0 | | 0.00 | | 0 | | 0.00 | | 0 | | 2 | | | 0 | | 2 | |  |  |  |  |
| Q96JK4 | HHIP-like protein 1 | HHIPL1 | 4.09 | 1 | | 86.7 | | 7.58 | | 0.16 | 1 | | 0.00 | | 0 | | 0.00 | | 0 | | 0.21 | | 1 | | 0.00 | | 0 | | 0.00 | | 0 | | 0.00 | | 0 | | 0.00 | | 0 | | 2 | | | 0 | | 2 | |  |  |  |  |
| A2RRP1 | Neuroblastoma-amplified sequence | NBAS | 1.48 | 2 | | 268.4 | | 5.96 | | 0.28 | 1 | | 0.00 | | 0 | | 0.00 | | 0 | | 0.21 | | 1 | | 0.00 | | 0 | | 0.00 | | 0 | | 0.00 | | 0 | | 0.00 | | 0 | | 2 | | | 0 | | 2 | |  |  |  |  |
| O95813 | Cerberus | CER1 | 6.74 | 1 | | 30.1 | | 7.78 | | 0.16 | 1 | | 0.00 | | 0 | | 0.00 | | 0 | | 0.21 | | 1 | | 0.00 | | 0 | | 0.00 | | 0 | | 0.00 | | 0 | | 0.00 | | 0 | | 2 | | | 0 | | 2 | |  |  |  |  |
| Q99471 | Prefoldin subunit 5 | PFDN5 | 12.34 | 1 | | 17.3 | | 6.33 | | 0.13 | 1 | | 0.00 | | 0 | | 0.00 | | 0 | | 0.17 | | 1 | | 0.00 | | 0 | | 0.00 | | 0 | | 0.00 | | 0 | | 0.00 | | 0 | | 2 | | | 0 | | 2 | |  |  |  |  |
| Q5TA82 | Late cornified envelope protein 2D | LCE2D | 22.73 | 1 | | 11.2 | | 8.09 | | 0.13 | 1 | | 0.00 | | 0 | | 0.00 | | 0 | | 0.17 | | 1 | | 0.00 | | 0 | | 0.00 | | 0 | | 0.00 | | 0 | | 0.00 | | 0 | | 2 | | | 0 | | 2 | |  |  |  |  |
| Q6PXP3 | Solute carrier family 2, facilitated glucose transporter member 7 | SLC2A7 | 4.30 | 1 | | 55.7 | | 8.41 | | 0.26 | 1 | | 0.00 | | 0 | | 0.00 | | 0 | | 0.16 | | 1 | | 0.00 | | 0 | | 0.00 | | 0 | | 0.00 | | 0 | | 0.00 | | 0 | | 2 | | | 0 | | 2 | |  |  |  |  |
| Q86VP6 | Cullin-associated NEDD8-dissociated protein 1 | CAND1 | 4.47 | 2 | | 136.3 | | 5.78 | | 0.16 | 1 | | 0.00 | | 0 | | 0.00 | | 0 | | 0.16 | | 1 | | 0.00 | | 0 | | 0.00 | | 0 | | 0.00 | | 0 | | 0.00 | | 0 | | 2 | | | 0 | | 2 | |  |  |  |  |
| Q96PP9 | Guanylate-binding protein 4 | GBP4 | 1.72 | 1 | | 73.1 | | 6.02 | | 0.28 | 1 | | 0.00 | | 0 | | 0.00 | | 0 | | 0.16 | | 1 | | 0.00 | | 0 | | 0.00 | | 0 | | 0.00 | | 0 | | 0.00 | | 0 | | 2 | | | 0 | | 2 | |  |  |  |  |
| Q14094 | Cyclin-I | CCNI | 3.18 | 1 | | 42.5 | | 8.00 | | 0.13 | 1 | | 0.00 | | 0 | | 0.00 | | 0 | | 0.16 | | 1 | | 0.00 | | 0 | | 0.00 | | 0 | | 0.00 | | 0 | | 0.00 | | 0 | | 2 | | | 0 | | 2 | |  |  |  |  |
| P35790 | Choline kinase alpha | CHKA | 8.32 | 2 | | 52.2 | | 6.55 | | 0.13 | 1 | | 0.00 | | 0 | | 0.00 | | 0 | | 0.16 | | 1 | | 0.00 | | 0 | | 0.00 | | 0 | | 0.00 | | 0 | | 0.00 | | 0 | | 2 | | | 0 | | 2 | |  |  |  |  |
| Q5MNZ6 | WD repeat domain phosphoinositide-interacting protein 3 | WDR45B | 3.49 | 1 | | 38.1 | | 7.59 | | 0.28 | 1 | | 0.00 | | 0 | | 0.00 | | 0 | | 0.16 | | 1 | | 0.00 | | 0 | | 0.00 | | 0 | | 0.00 | | 0 | | 0.00 | | 0 | | 2 | | | 0 | | 2 | |  |  |  |  |
| Q92833 | Protein Jumonji | JARID2 | 0.96 | 1 | | 138.6 | | 9.38 | | 0.13 | 1 | | 0.00 | | 0 | | 0.00 | | 0 | | 0.16 | | 1 | | 0.00 | | 0 | | 0.00 | | 0 | | 0.00 | | 0 | | 0.00 | | 0 | | 2 | | | 0 | | 2 | |  |  |  |  |
| H3BQK9 | Microtubule-actin cross-linking factor 1, isoforms 1/2/3/5 | MACF1 | 0.38 | 2 | | 860.5 | | 5.38 | | 0.13 | 1 | | 0.00 | | 0 | | 0.00 | | 0 | | 0.16 | | 1 | | 0.00 | | 0 | | 0.00 | | 0 | | 0.00 | | 0 | | 0.00 | | 0 | | 2 | | | 0 | | 2 | |  |  |  |  |
| Q96KP4 | Cytosolic non-specific dipeptidase | CNDP2 | 12.84 | 5 | | 52.8 | | 5.97 | | 0.13 | 1 | | 1.37 | | 3 | | 0.00 | | 0 | | 0.00 | | 0 | | 0.00 | | 0 | | 0.00 | | 0 | | 0.00 | | 0 | | 0.00 | | 0 | | 2 | | | 0 | | 2 | |  |  |  |  |
| P04179 | Superoxide dismutase [Mn], mitochondrial | SOD2 | 12.61 | 2 | | 24.7 | | 8.25 | | 0.57 | 1 | | 1.27 | | 3 | | 0.00 | | 0 | | 0.00 | | 0 | | 0.00 | | 0 | | 0.00 | | 0 | | 0.00 | | 0 | | 0.00 | | 0 | | 2 | | | 0 | | 2 | |  |  |  |  |
| A0A0C4DGB5 | Calpastatin | CAST | 3.98 | 2 | | 81.0 | | 5.10 | | 0.13 | 1 | | 0.99 | | 3 | | 0.00 | | 0 | | 0.00 | | 0 | | 0.00 | | 0 | | 0.00 | | 0 | | 0.00 | | 0 | | 0.00 | | 0 | | 2 | | | 0 | | 2 | |  |  |  |  |
| P13500 | C-C motif chemokine 2 | CCL2 | 11.11 | 1 | | 11.0 | | 9.25 | | 0.28 | 1 | | 0.48 | | 2 | | 0.00 | | 0 | | 0.00 | | 0 | | 0.00 | | 0 | | 0.00 | | 0 | | 0.00 | | 0 | | 0.00 | | 0 | | 2 | | | 0 | | 2 | |  |  |  |  |
| P13765 | HLA class II histocompatibility antigen, DO beta chain | HLA-DOB | 3.66 | 1 | | 30.8 | | 6.79 | | 0.15 | 1 | | 0.27 | | 2 | | 0.00 | | 0 | | 0.00 | | 0 | | 0.00 | | 0 | | 0.00 | | 0 | | 0.00 | | 0 | | 0.00 | | 0 | | 2 | | | 0 | | 2 | |  |  |  |  |
| P12081 | Histidine--tRNA ligase, cytoplasmic | HARS1 | 4.32 | 1 | | 57.4 | | 5.88 | | 0.13 | 1 | | 0.30 | | 2 | | 0.00 | | 0 | | 0.00 | | 0 | | 0.00 | | 0 | | 0.00 | | 0 | | 0.00 | | 0 | | 0.00 | | 0 | | 2 | | | 0 | | 2 | |  |  |  |  |
| P11766 | Alcohol dehydrogenase class-3 | ADH5 | 10.70 | 3 | | 39.7 | | 7.49 | | 0.15 | 1 | | 0.27 | | 1 | | 0.00 | | 0 | | 0.00 | | 0 | | 0.00 | | 0 | | 0.00 | | 0 | | 0.00 | | 0 | | 0.00 | | 0 | | 2 | | | 0 | | 2 | |  |  |  |  |
| Q9NZL9 | Methionine adenosyltransferase 2 subunit beta | MAT2B | 6.59 | 1 | | 37.5 | | 7.36 | | 0.16 | 1 | | 0.14 | | 1 | | 0.00 | | 0 | | 0.00 | | 0 | | 0.00 | | 0 | | 0.00 | | 0 | | 0.00 | | 0 | | 0.00 | | 0 | | 2 | | | 0 | | 2 | |  |  |  |  |
| Q8TEM1 | Nuclear pore membrane glycoprotein 210 | NUP210 | 0.64 | 1 | | 205.0 | | 6.81 | | 0.16 | 1 | | 0.16 | | 1 | | 0.00 | | 0 | | 0.00 | | 0 | | 0.00 | | 0 | | 0.00 | | 0 | | 0.00 | | 0 | | 0.00 | | 0 | | 2 | | | 0 | | 2 | |  |  |  |  |
| D6RGW2 | Neurexophilin-3 | NXPH3 | 2.82 | 1 | | 38.8 | | 9.09 | | 0.15 | 1 | | 0.14 | | 1 | | 0.00 | | 0 | | 0.00 | | 0 | | 0.00 | | 0 | | 0.00 | | 0 | | 0.00 | | 0 | | 0.00 | | 0 | | 2 | | | 0 | | 2 | |  |  |  |  |
| O43166 | Sial-induced proliferation-associated 1-like protein 1 | SIPA1L1 | 1.22 | 2 | | 199.9 | | 8.19 | | 0.15 | 1 | | 0.14 | | 1 | | 0.00 | | 0 | | 0.00 | | 0 | | 0.00 | | 0 | | 0.00 | | 0 | | 0.00 | | 0 | | 0.00 | | 0 | | 2 | | | 0 | | 2 | |  |  |  |  |
| Q9UQ52 | Contactin-6 | CNTN6 | 2.04 | 2 | | 113.9 | | 6.00 | | 0.28 | 1 | | 0.14 | | 1 | | 0.00 | | 0 | | 0.00 | | 0 | | 0.00 | | 0 | | 0.00 | | 0 | | 0.00 | | 0 | | 0.00 | | 0 | | 2 | | | 0 | | 2 | |  |  |  |  |
| Q96QS1 | Tetraspanin-32 | TSPAN32 | 9.38 | 1 | | 34.6 | | 8.40 | | 0.28 | 1 | | 0.13 | | 1 | | 0.00 | | 0 | | 0.00 | | 0 | | 0.00 | | 0 | | 0.00 | | 0 | | 0.00 | | 0 | | 0.00 | | 0 | | 2 | | | 0 | | 2 | |  |  |  |  |
| O75879 | Glutamyl-tRNA(Gln) amidotransferase subunit B, mitochondrial | GATB | 4.49 | 2 | | 61.8 | | 8.63 | | 0.28 | 1 | | 0.13 | | 1 | | 0.00 | | 0 | | 0.00 | | 0 | | 0.00 | | 0 | | 0.00 | | 0 | | 0.00 | | 0 | | 0.00 | | 0 | | 2 | | | 0 | | 2 | |  |  |  |  |
| A0A0G2JNW7 | Solute carrier family 12 member 7 | SLC12A7 | 0.74 | 1 | | 119.1 | | 6.71 | | 0.13 | 1 | | 0.14 | | 1 | | 0.00 | | 0 | | 0.00 | | 0 | | 0.00 | | 0 | | 0.00 | | 0 | | 0.00 | | 0 | | 0.00 | | 0 | | 2 | | | 0 | | 2 | |  |  |  |  |
| E9PIL3 | CKLF-like MARVEL transmembrane domain-containing protein 1 | CMTM1 | 7.98 | 1 | | 25.5 | | 10.07 | | 0.13 | 1 | | 0.16 | | 1 | | 0.00 | | 0 | | 0.00 | | 0 | | 0.00 | | 0 | | 0.00 | | 0 | | 0.00 | | 0 | | 0.00 | | 0 | | 2 | | | 0 | | 2 | |  |  |  |  |
| E7EX82 | Polyhomeotic-like protein 3 | PHC3 | 10.87 | 2 | | 89.1 | | 6.93 | | 0.13 | 1 | | 0.13 | | 1 | | 0.00 | | 0 | | 0.00 | | 0 | | 0.00 | | 0 | | 0.00 | | 0 | | 0.00 | | 0 | | 0.00 | | 0 | | 2 | | | 0 | | 2 | |  |  |  |  |
| P29374 | AT-rich interactive domain-containing protein 4A | ARID4A | 2.94 | 1 | | 142.7 | | 5.10 | | 0.13 | 1 | | 0.13 | | 1 | | 0.00 | | 0 | | 0.00 | | 0 | | 0.00 | | 0 | | 0.00 | | 0 | | 0.00 | | 0 | | 0.00 | | 0 | | 2 | | | 0 | | 2 | |  |  |  |  |
| Q96DX7 | Tripartite motif-containing protein 44 | TRIM44 | 23.26 | 2 | | 38.4 | | 4.21 | | 0.13 | 1 | | 0.13 | | 1 | | 0.00 | | 0 | | 0.00 | | 0 | | 0.00 | | 0 | | 0.00 | | 0 | | 0.00 | | 0 | | 0.00 | | 0 | | 2 | | | 0 | | 2 | |  |  |  |  |
| Q9ULL5 | Proline-rich protein 12 | PRR12 | 0.59 | 1 | | 210.9 | | 7.87 | | 0.00 | 0 | | 0.00 | | 0 | | 1.05 | | 3 | | 0.36 | | 2 | | 0.00 | | 0 | | 0.00 | | 0 | | 0.00 | | 0 | | 0.00 | | 0 | | 2 | | | 0 | | 2 | |  |  |  |  |
| P17936 | Insulin-like growth factor-binding protein 3 | IGFBP3 | 7.90 | 2 | | 31.7 | | 8.69 | | 0.00 | 0 | | 0.00 | | 0 | | 0.91 | | 4 | | 1.15 | | 3 | | 0.00 | | 0 | | 0.00 | | 0 | | 0.00 | | 0 | | 0.00 | | 0 | | 2 | | | 0 | | 2 | |  |  |  |  |
| H0YJE6 | Spectrin beta chain, erythrocytic (Fragment) | SPTB | 1.85 | 1 | | 117.4 | | 5.20 | | 0.00 | 0 | | 0.00 | | 0 | | 0.63 | | 2 | | 0.42 | | 1 | | 0.00 | | 0 | | 0.00 | | 0 | | 0.00 | | 0 | | 0.00 | | 0 | | 2 | | | 0 | | 2 | |  |  |  |  |
| P43652 | Afamin | AFM | 1.17 | 1 | | 69.0 | | 5.90 | | 0.00 | 0 | | 0.00 | | 0 | | 0.60 | | 2 | | 0.84 | | 2 | | 0.00 | | 0 | | 0.00 | | 0 | | 0.00 | | 0 | | 0.00 | | 0 | | 2 | | | 0 | | 2 | |  |  |  |  |
| Q16222 | UDP-N-acetylhexosamine pyrophosphorylase | UAP1 | 5.56 | 2 | | 58.7 | | 6.33 | | 0.00 | 0 | | 0.57 | | 4 | | 0.54 | | 3 | | 0.00 | | 0 | | 0.00 | | 0 | | 0.00 | | 0 | | 0.00 | | 0 | | 0.00 | | 0 | | 2 | | | 0 | | 2 | |  |  |  |  |
| A0A096LP10 | Putative bifunctional UDP-N-acetylglucosamine transferase and deubiquitinase ALG13 (Fragment) | ALG13 | 14.20 | 1 | | 17.8 | | 6.52 | | 0.00 | 0 | | 0.00 | | 0 | | 0.41 | | 2 | | 0.16 | | 1 | | 0.00 | | 0 | | 0.00 | | 0 | | 0.00 | | 0 | | 0.00 | | 0 | | 2 | | | 0 | | 2 | |  |  |  |  |
| P49758 | Regulator of G-protein sialing 6 | RGS6 | 3.81 | 2 | | 54.4 | | 7.42 | | 0.00 | 0 | | 0.16 | | 1 | | 0.38 | | 2 | | 0.00 | | 0 | | 0.00 | | 0 | | 0.00 | | 0 | | 0.00 | | 0 | | 0.00 | | 0 | | 2 | | | 0 | | 2 | |  |  |  |  |
| Q9Y224 | RNA transcription, translation and transport factor protein | RTRAF | 8.20 | 1 | | 28.1 | | 6.65 | | 0.00 | 0 | | 0.27 | | 2 | | 0.31 | | 2 | | 0.00 | | 0 | | 0.00 | | 0 | | 0.00 | | 0 | | 0.00 | | 0 | | 0.00 | | 0 | | 2 | | | 0 | | 2 | |  |  |  |  |
| Q9H832 | Ubiquitin-conjugating enzyme E2 Z | UBE2Z | 2.82 | 1 | | 38.2 | | 5.62 | | 0.00 | 0 | | 0.14 | | 1 | | 0.31 | | 2 | | 0.00 | | 0 | | 0.00 | | 0 | | 0.00 | | 0 | | 0.00 | | 0 | | 0.00 | | 0 | | 2 | | | 0 | | 2 | |  |  |  |  |
| Q9ULL4 | Plexin-B3 | PLXNB3 | 1.57 | 1 | | 206.7 | | 6.40 | | 0.00 | 0 | | 0.00 | | 0 | | 0.22 | | 1 | | 0.38 | | 2 | | 0.00 | | 0 | | 0.00 | | 0 | | 0.00 | | 0 | | 0.00 | | 0 | | 2 | | | 0 | | 2 | |  |  |  |  |
| H0Y8C6 | Importin-5 (Fragment) | IPO5 | 1.82 | 1 | | 123.8 | | 5.06 | | 0.00 | 0 | | 0.00 | | 0 | | 0.22 | | 1 | | 0.21 | | 1 | | 0.00 | | 0 | | 0.00 | | 0 | | 0.00 | | 0 | | 0.00 | | 0 | | 2 | | | 0 | | 2 | |  |  |  |  |
| Q9H6U6 | Breast carcinoma-amplified sequence 3 | BCAS3 | 4.20 | 2 | | 101.2 | | 6.70 | | 0.00 | 0 | | 0.00 | | 0 | | 0.22 | | 1 | | 0.16 | | 1 | | 0.00 | | 0 | | 0.00 | | 0 | | 0.00 | | 0 | | 0.00 | | 0 | | 2 | | | 0 | | 2 | |  |  |  |  |
| Q99726 | Zinc transporter 3 | SLC30A3 | 2.06 | 1 | | 41.9 | | 6.47 | | 0.00 | 0 | | 0.00 | | 0 | | 0.22 | | 1 | | 0.16 | | 1 | | 0.00 | | 0 | | 0.00 | | 0 | | 0.00 | | 0 | | 0.00 | | 0 | | 2 | | | 0 | | 2 | |  |  |  |  |
| A0A2R8Y4G3 | Uncharacterized protein |  | 13.27 | 1 | | 21.6 | | 6.90 | | 0.00 | 0 | | 0.14 | | 1 | | 0.22 | | 1 | | 0.00 | | 0 | | 0.00 | | 0 | | 0.00 | | 0 | | 0.00 | | 0 | | 0.00 | | 0 | | 2 | | | 0 | | 2 | |  |  |  |  |
| Q9H094 | Neuroblastoma breakpoint family member 3 | NBPF3 | 1.90 | 1 | | 72.9 | | 4.50 | | 0.00 | 0 | | 0.14 | | 1 | | 0.22 | | 1 | | 0.00 | | 0 | | 0.00 | | 0 | | 0.00 | | 0 | | 0.00 | | 0 | | 0.00 | | 0 | | 2 | | | 0 | | 2 | |  |  |  |  |
| Q96EB6 | NAD-dependent protein deacetylase sirtuin-1 | SIRT1 | 4.55 | 1 | | 81.6 | | 4.67 | | 0.00 | 0 | | 0.00 | | 0 | | 0.22 | | 1 | | 0.78 | | 2 | | 0.00 | | 0 | | 0.00 | | 0 | | 0.00 | | 0 | | 0.00 | | 0 | | 2 | | | 0 | | 2 | |  |  |  |  |
| Q66K64 | DDB1- and CUL4-associated factor 15 | DCAF15 | 4.50 | 1 | | 66.4 | | 6.58 | | 0.00 | 0 | | 0.00 | | 0 | | 0.22 | | 1 | | 0.37 | | 2 | | 0.00 | | 0 | | 0.00 | | 0 | | 0.00 | | 0 | | 0.00 | | 0 | | 2 | | | 0 | | 2 | |  |  |  |  |
| Q9Y3B9 | RRP15-like protein | RRP15 | 19.50 | 2 | | 31.5 | | 5.52 | | 0.00 | 0 | | 0.00 | | 0 | | 0.22 | | 1 | | 0.16 | | 1 | | 0.00 | | 0 | | 0.00 | | 0 | | 0.00 | | 0 | | 0.00 | | 0 | | 2 | | | 0 | | 2 | |  |  |  |  |
| A8MVJ9 | Putative histone PARylation factor 1-like |  | 2.31 | 1 | | 39.7 | | 8.10 | | 0.00 | 0 | | 0.29 | | 2 | | 0.22 | | 1 | | 0.00 | | 0 | | 0.00 | | 0 | | 0.00 | | 0 | | 0.00 | | 0 | | 0.00 | | 0 | | 2 | | | 0 | | 2 | |  |  |  |  |
| Q9H426 | Regulating synaptic membrane exocytosis protein 4 | RIMS4 | 6.69 | 1 | | 29.3 | | 5.80 | | 0.00 | 0 | | 0.13 | | 1 | | 0.22 | | 1 | | 0.00 | | 0 | | 0.00 | | 0 | | 0.00 | | 0 | | 0.00 | | 0 | | 0.00 | | 0 | | 2 | | | 0 | | 2 | |  |  |  |  |
| Q8NG68 | Tubulin--tyrosine ligase | TTL | 2.39 | 1 | | 43.2 | | 6.74 | | 0.00 | 0 | | 0.13 | | 1 | | 0.22 | | 1 | | 0.00 | | 0 | | 0.00 | | 0 | | 0.00 | | 0 | | 0.00 | | 0 | | 0.00 | | 0 | | 2 | | | 0 | | 2 | |  |  |  |  |
| D6RDI6 | Claudin domain-containing protein 1 (Fragment) | CLDND1 | 15.90 | 1 | | 26.9 | | 5.19 | | 0.00 | 0 | | 0.00 | | 0 | | 0.19 | | 1 | | 0.16 | | 1 | | 0.00 | | 0 | | 0.00 | | 0 | | 0.00 | | 0 | | 0.00 | | 0 | | 2 | | | 0 | | 2 | |  |  |  |  |
| Q9UK76 | Jupiter microtubule associated homolog 1 | JPT1 | 25.32 | 2 | | 16.0 | | 5.60 | | 0.00 | 0 | | 0.14 | | 1 | | 0.19 | | 1 | | 0.00 | | 0 | | 0.00 | | 0 | | 0.00 | | 0 | | 0.00 | | 0 | | 0.00 | | 0 | | 2 | | | 0 | | 2 | |  |  |  |  |
| Q08EI0 | AOF1 protein | KDM1B | 15.93 | 1 | | 12.5 | | 4.97 | | 0.00 | 0 | | 0.00 | | 0 | | 0.19 | | 1 | | 0.37 | | 2 | | 0.00 | | 0 | | 0.00 | | 0 | | 0.00 | | 0 | | 0.00 | | 0 | | 2 | | | 0 | | 2 | |  |  |  |  |
| H7BZR8 | SEC14 domain and spectrin repeat-containing protein 1 (Fragment) | SESTD1 | 9.33 | 1 | | 26.0 | | 5.24 | | 0.00 | 0 | | 0.00 | | 0 | | 0.19 | | 1 | | 0.21 | | 1 | | 0.00 | | 0 | | 0.00 | | 0 | | 0.00 | | 0 | | 0.00 | | 0 | | 2 | | | 0 | | 2 | |  |  |  |  |
| Q01955 | Collagen alpha-3(IV) chain | COL4A3 | 2.10 | 2 | | 161.7 | | 9.16 | | 0.00 | 0 | | 0.00 | | 0 | | 0.19 | | 1 | | 0.16 | | 1 | | 0.00 | | 0 | | 0.00 | | 0 | | 0.00 | | 0 | | 0.00 | | 0 | | 2 | | | 0 | | 2 | |  |  |  |  |
| O15042 | U2 snRNP-associated SURP motif-containing protein | U2SURP | 1.94 | 1 | | 118.2 | | 8.47 | | 0.00 | 0 | | 0.16 | | 1 | | 0.19 | | 1 | | 0.00 | | 0 | | 0.00 | | 0 | | 0.00 | | 0 | | 0.00 | | 0 | | 0.00 | | 0 | | 2 | | | 0 | | 2 | |  |  |  |  |
| A0A1B0GUN5 | Pleckstrin homology domain-containing family A member 6 | PLEKHA6 | 0.77 | 1 | | 131.2 | | 8.87 | | 0.00 | 0 | | 0.16 | | 1 | | 0.19 | | 1 | | 0.00 | | 0 | | 0.00 | | 0 | | 0.00 | | 0 | | 0.00 | | 0 | | 0.00 | | 0 | | 2 | | | 0 | | 2 | |  |  |  |  |
| Q6ZS30 | Neurobeachin-like protein 1 | NBEAL1 | 0.97 | 1 | | 307.0 | | 6.44 | | 0.00 | 0 | | 0.00 | | 0 | | 0.16 | | 1 | | 0.21 | | 1 | | 0.00 | | 0 | | 0.00 | | 0 | | 0.00 | | 0 | | 0.00 | | 0 | | 2 | | | 0 | | 2 | |  |  |  |  |
| Q8TCU4 | Alstrom syndrome protein 1 | ALMS1 | 0.55 | 1 | | 460.8 | | 6.28 | | 0.00 | 0 | | 0.00 | | 0 | | 0.16 | | 1 | | 0.16 | | 1 | | 0.00 | | 0 | | 0.00 | | 0 | | 0.00 | | 0 | | 0.00 | | 0 | | 2 | | | 0 | | 2 | |  |  |  |  |
| Q1MSJ5 | Centrosome and spindle pole-associated protein 1 | CSPP1 | 1.83 | 2 | | 145.4 | | 6.80 | | 0.00 | 0 | | 0.00 | | 0 | | 0.16 | | 1 | | 0.16 | | 1 | | 0.00 | | 0 | | 0.00 | | 0 | | 0.00 | | 0 | | 0.00 | | 0 | | 2 | | | 0 | | 2 | |  |  |  |  |
| O00764 | Pyridoxal kinase | PDXK | 6.73 | 1 | | 35.1 | | 6.13 | | 0.00 | 0 | | 0.40 | | 3 | | 0.16 | | 1 | | 0.00 | | 0 | | 0.00 | | 0 | | 0.00 | | 0 | | 0.00 | | 0 | | 0.00 | | 0 | | 2 | | | 0 | | 2 | |  |  |  |  |
| P26368 | Splicing factor U2AF 65 kDa subunit | U2AF2 | 7.16 | 2 | | 53.5 | | 9.09 | | 0.00 | 0 | | 0.83 | | 2 | | 0.16 | | 1 | | 0.00 | | 0 | | 0.00 | | 0 | | 0.00 | | 0 | | 0.00 | | 0 | | 0.00 | | 0 | | 2 | | | 0 | | 2 | |  |  |  |  |
| Q9UBD0 | Heat shock transcription factor, X-linked | HSFX1 | 2.36 | 1 | | 46.7 | | 7.11 | | 0.00 | 0 | | 0.14 | | 1 | | 0.16 | | 1 | | 0.00 | | 0 | | 0.00 | | 0 | | 0.00 | | 0 | | 0.00 | | 0 | | 0.00 | | 0 | | 2 | | | 0 | | 2 | |  |  |  |  |
| Q9UKA9 | Polypyrimidine tract-binding protein 2 | PTBP2 | 1.51 | 1 | | 57.5 | | 8.66 | | 0.00 | 0 | | 0.14 | | 1 | | 0.16 | | 1 | | 0.00 | | 0 | | 0.00 | | 0 | | 0.00 | | 0 | | 0.00 | | 0 | | 0.00 | | 0 | | 2 | | | 0 | | 2 | |  |  |  |  |
| Q6QNK2 | Adhesion G-protein coupled receptor D1 | ADGRD1 | 4.69 | 1 | | 96.5 | | 7.88 | | 0.00 | 0 | | 0.14 | | 1 | | 0.16 | | 1 | | 0.00 | | 0 | | 0.00 | | 0 | | 0.00 | | 0 | | 0.00 | | 0 | | 0.00 | | 0 | | 2 | | | 0 | | 2 | |  |  |  |  |
| Q86UU1 | Pleckstrin homology-like domain family B member 1 | PHLDB1 | 0.94 | 1 | | 151.1 | | 8.63 | | 0.00 | 0 | | 0.13 | | 1 | | 0.16 | | 1 | | 0.00 | | 0 | | 0.00 | | 0 | | 0.00 | | 0 | | 0.00 | | 0 | | 0.00 | | 0 | | 2 | | | 0 | | 2 | |  |  |  |  |
| P38646 | Stress-70 protein, mitochondrial | HSPA9 | 3.83 | 2 | | 73.6 | | 6.16 | | 0.00 | 0 | | 0.14 | | 1 | | 0.16 | | 1 | | 0.00 | | 0 | | 0.00 | | 0 | | 0.00 | | 0 | | 0.00 | | 0 | | 0.00 | | 0 | | 2 | | | 0 | | 2 | |  |  |  |  |
| Q9Y3A0 | Ubiquinone biosynthesis protein COQ4 homolog, mitochondrial | COQ4 | 5.28 | 1 | | 29.6 | | 9.17 | | 0.00 | 0 | | 0.14 | | 1 | | 0.16 | | 1 | | 0.00 | | 0 | | 0.00 | | 0 | | 0.00 | | 0 | | 0.00 | | 0 | | 0.00 | | 0 | | 2 | | | 0 | | 2 | |  |  |  |  |
| Q8IUH3 | RNA-binding protein 45 | RBM45 | 2.73 | 1 | | 53.5 | | 7.17 | | 0.00 | 0 | | 0.14 | | 1 | | 0.16 | | 1 | | 0.00 | | 0 | | 0.00 | | 0 | | 0.00 | | 0 | | 0.00 | | 0 | | 0.00 | | 0 | | 2 | | | 0 | | 2 | |  |  |  |  |
| Q13642 | Four and a half LIM domains protein 1 | FHL1 | 3.72 | 1 | | 36.2 | | 8.97 | | 0.00 | 0 | | 0.00 | | 0 | | 0.16 | | 1 | | 0.83 | | 3 | | 0.00 | | 0 | | 0.00 | | 0 | | 0.00 | | 0 | | 0.00 | | 0 | | 2 | | | 0 | | 2 | |  |  |  |  |
| Q96KG7 | Multiple epidermal growth factor-like domains protein 10 | MEGF10 | 4.82 | 2 | | 122.1 | | 6.87 | | 0.00 | 0 | | 0.00 | | 0 | | 0.16 | | 1 | | 0.17 | | 1 | | 0.00 | | 0 | | 0.00 | | 0 | | 0.00 | | 0 | | 0.00 | | 0 | | 2 | | | 0 | | 2 | |  |  |  |  |
| A8MXH5 | Collagen alpha-6(IV) chain | COL4A6 | 5.39 | 3 | | 165.4 | | 9.06 | | 0.00 | 0 | | 0.28 | | 1 | | 0.16 | | 1 | | 0.00 | | 0 | | 0.00 | | 0 | | 0.00 | | 0 | | 0.00 | | 0 | | 0.00 | | 0 | | 2 | | | 0 | | 2 | |  |  |  |  |
| M0QZD8 | Uncharacterized protein | LOC400499 | 0.52 | 2 | | 357.6 | | 7.85 | | 0.00 | 0 | | 0.14 | | 1 | | 0.16 | | 1 | | 0.00 | | 0 | | 0.00 | | 0 | | 0.00 | | 0 | | 0.00 | | 0 | | 0.00 | | 0 | | 2 | | | 0 | | 2 | |  |  |  |  |
| K7ELL7 | Glucosidase 2 subunit beta | PRKCSH | 4.30 | 2 | | 60.2 | | 4.41 | | 0.00 | 0 | | 0.16 | | 1 | | 0.16 | | 1 | | 0.00 | | 0 | | 0.00 | | 0 | | 0.00 | | 0 | | 0.00 | | 0 | | 0.00 | | 0 | | 2 | | | 0 | | 2 | |  |  |  |  |
| O95155 | Ubiquitin conjugation factor E4 B | UBE4B | 0.54 | 1 | | 146.1 | | 6.55 | | 0.00 | 0 | | 0.16 | | 1 | | 0.16 | | 1 | | 0.00 | | 0 | | 0.00 | | 0 | | 0.00 | | 0 | | 0.00 | | 0 | | 0.00 | | 0 | | 2 | | | 0 | | 2 | |  |  |  |  |
| P30044 | Peroxiredoxin-5, mitochondrial | PRDX5 | 16.82 | 3 | | 22.1 | | 8.70 | | 0.00 | 0 | | 2.17 | | 4 | | 0.00 | | 0 | | 0.83 | | 2 | | 0.00 | | 0 | | 0.00 | | 0 | | 0.00 | | 0 | | 0.00 | | 0 | | 2 | | | 0 | | 2 | |  |  |  |  |
| Q96PV0 | Ras/Rap GTPase-activating protein SynGAP | SYNGAP1 | 0.74 | 1 | | 148.2 | | 8.98 | | 0.00 | 0 | | 0.16 | | 1 | | 0.00 | | 0 | | 0.78 | | 3 | | 0.00 | | 0 | | 0.00 | | 0 | | 0.00 | | 0 | | 0.00 | | 0 | | 2 | | | 0 | | 2 | |  |  |  |  |
| P49754 | Vacuolar protein sorting-associated protein 41 homolog | VPS41 | 3.51 | 1 | | 98.5 | | 5.85 | | 0.00 | 0 | | 0.14 | | 1 | | 0.00 | | 0 | | 0.53 | | 3 | | 0.00 | | 0 | | 0.00 | | 0 | | 0.00 | | 0 | | 0.00 | | 0 | | 2 | | | 0 | | 2 | |  |  |  |  |
| O00469 | Procollagen-lysine,2-oxoglutarate 5-dioxygenase 2 | PLOD2 | 1.36 | 1 | | 84.6 | | 6.71 | | 0.00 | 0 | | 0.32 | | 2 | | 0.00 | | 0 | | 0.49 | | 3 | | 0.00 | | 0 | | 0.00 | | 0 | | 0.00 | | 0 | | 0.00 | | 0 | | 2 | | | 0 | | 2 | |  |  |  |  |
| Q86XX4 | Extracellular matrix protein FRAS1 | FRAS1 | 0.35 | 1 | | 442.9 | | 5.57 | | 0.00 | 0 | | 0.13 | | 1 | | 0.00 | | 0 | | 0.47 | | 3 | | 0.00 | | 0 | | 0.00 | | 0 | | 0.00 | | 0 | | 0.00 | | 0 | | 2 | | | 0 | | 2 | |  |  |  |  |
| Q9BWW8 | Apolipoprotein L6 | APOL6 | 6.41 | 1 | | 38.1 | | 8.35 | | 0.00 | 0 | | 0.70 | | 3 | | 0.00 | | 0 | | 0.47 | | 2 | | 0.00 | | 0 | | 0.00 | | 0 | | 0.00 | | 0 | | 0.00 | | 0 | | 2 | | | 0 | | 2 | |  |  |  |  |
| A0A0J9YWL0 | Beta/gamma crystallin domain-containing protein 1 | CRYBG1 | 0.89 | 2 | | 231.6 | | 5.81 | | 0.00 | 0 | | 0.13 | | 1 | | 0.00 | | 0 | | 0.32 | | 2 | | 0.00 | | 0 | | 0.00 | | 0 | | 0.00 | | 0 | | 0.00 | | 0 | | 2 | | | 0 | | 2 | |  |  |  |  |
| Q12907 | Vesicular integral-membrane protein VIP36 | LMAN2 | 7.30 | 1 | | 40.2 | | 6.95 | | 0.00 | 0 | | 0.14 | | 1 | | 0.00 | | 0 | | 0.32 | | 2 | | 0.00 | | 0 | | 0.00 | | 0 | | 0.00 | | 0 | | 0.00 | | 0 | | 2 | | | 0 | | 2 | |  |  |  |  |
| A0A024R1R8 | HCG2014768, isoform CRA_a | hCG_2014768 | 14.06 | 1 | | 7.1 | | 9.99 | | 0.00 | 0 | | 0.13 | | 1 | | 0.00 | | 0 | | 0.31 | | 2 | | 0.00 | | 0 | | 0.00 | | 0 | | 0.00 | | 0 | | 0.00 | | 0 | | 2 | | | 0 | | 2 | |  |  |  |  |
| H7C2N1 | Prothymosin alpha (Fragment) | PTMA | 10.14 | 2 | | 15.8 | | 4.23 | | 0.00 | 0 | | 0.27 | | 1 | | 0.00 | | 0 | | 0.31 | | 1 | | 0.00 | | 0 | | 0.00 | | 0 | | 0.00 | | 0 | | 0.00 | | 0 | | 2 | | | 0 | | 2 | |  |  |  |  |
| O75056 | Syndecan-3 | SDC3 | 2.49 | 1 | | 45.5 | | 4.69 | | 0.00 | 0 | | 0.27 | | 2 | | 0.00 | | 0 | | 0.21 | | 1 | | 0.00 | | 0 | | 0.00 | | 0 | | 0.00 | | 0 | | 0.00 | | 0 | | 2 | | | 0 | | 2 | |  |  |  |  |
| Q96Q89 | Kinesin-like protein KIF20B | KIF20B | 2.14 | 2 | | 210.5 | | 5.67 | | 0.00 | 0 | | 0.13 | | 1 | | 0.00 | | 0 | | 0.21 | | 1 | | 0.00 | | 0 | | 0.00 | | 0 | | 0.00 | | 0 | | 0.00 | | 0 | | 2 | | | 0 | | 2 | |  |  |  |  |
| E7ET48 | Beta/gamma crystallin domain-containing protein 2 | CRYBG2 | 2.42 | 2 | | 212.2 | | 6.18 | | 0.00 | 0 | | 0.14 | | 1 | | 0.00 | | 0 | | 0.21 | | 1 | | 0.00 | | 0 | | 0.00 | | 0 | | 0.00 | | 0 | | 0.00 | | 0 | | 2 | | | 0 | | 2 | |  |  |  |  |
| Q9NWF9 | E3 ubiquitin-protein ligase RNF216 | RNF216 | 9.12 | 2 | | 99.3 | | 4.91 | | 0.00 | 0 | | 0.14 | | 1 | | 0.00 | | 0 | | 0.21 | | 1 | | 0.00 | | 0 | | 0.00 | | 0 | | 0.00 | | 0 | | 0.00 | | 0 | | 2 | | | 0 | | 2 | |  |  |  |  |
| P0DMQ9 | Putative uncharacterized protein C8orf89 | C8orf89 | 9.32 | 1 | | 18.1 | | 9.44 | | 0.00 | 0 | | 0.14 | | 1 | | 0.00 | | 0 | | 0.21 | | 1 | | 0.00 | | 0 | | 0.00 | | 0 | | 0.00 | | 0 | | 0.00 | | 0 | | 2 | | | 0 | | 2 | |  |  |  |  |
| C9K0J5 | Ras association (RalGDS/AF-6) and pleckstrin homology domains 1, isoform CRA_b | RAPH1 | 0.54 | 1 | | 141.1 | | 8.90 | | 0.00 | 0 | | 0.30 | | 2 | | 0.00 | | 0 | | 0.17 | | 1 | | 0.00 | | 0 | | 0.00 | | 0 | | 0.00 | | 0 | | 0.00 | | 0 | | 2 | | | 0 | | 2 | |  |  |  |  |
| Q9NVR0 | Kelch-like protein 11 | KLHL11 | 2.54 | 1 | | 80.1 | | 6.16 | | 0.00 | 0 | | 0.14 | | 1 | | 0.00 | | 0 | | 0.17 | | 1 | | 0.00 | | 0 | | 0.00 | | 0 | | 0.00 | | 0 | | 0.00 | | 0 | | 2 | | | 0 | | 2 | |  |  |  |  |
| A6NHP3 | Speedy protein E2B | SPDYE2B | 9.20 | 2 | | 48.3 | | 9.76 | | 0.00 | 0 | | 0.16 | | 1 | | 0.00 | | 0 | | 0.17 | | 1 | | 0.00 | | 0 | | 0.00 | | 0 | | 0.00 | | 0 | | 0.00 | | 0 | | 2 | | | 0 | | 2 | |  |  |  |  |
| Q13442 | 28 kDa heat- and acid-stable phosphoprotein | PDAP1 | 10.50 | 1 | | 20.6 | | 8.87 | | 0.00 | 0 | | 0.13 | | 1 | | 0.00 | | 0 | | 0.17 | | 1 | | 0.00 | | 0 | | 0.00 | | 0 | | 0.00 | | 0 | | 0.00 | | 0 | | 2 | | | 0 | | 2 | |  |  |  |  |
| P09622 | Dihydrolipoyl dehydrogenase, mitochondrial | DLD | 6.48 | 2 | | 54.1 | | 7.85 | | 0.00 | 0 | | 0.45 | | 2 | | 0.00 | | 0 | | 0.16 | | 1 | | 0.00 | | 0 | | 0.00 | | 0 | | 0.00 | | 0 | | 0.00 | | 0 | | 2 | | | 0 | | 2 | |  |  |  |  |
| Q14693 | Phosphatidate phosphatase LPIN1 | LPIN1 | 8.20 | 2 | | 98.6 | | 6.58 | | 0.00 | 0 | | 0.16 | | 1 | | 0.00 | | 0 | | 0.16 | | 1 | | 0.00 | | 0 | | 0.00 | | 0 | | 0.00 | | 0 | | 0.00 | | 0 | | 2 | | | 0 | | 2 | |  |  |  |  |
| Q9H095 | Dynein regulatory complex protein 9 | IQCG | 2.26 | 1 | | 51.9 | | 6.52 | | 0.00 | 0 | | 0.14 | | 1 | | 0.00 | | 0 | | 0.16 | | 1 | | 0.00 | | 0 | | 0.00 | | 0 | | 0.00 | | 0 | | 0.00 | | 0 | | 2 | | | 0 | | 2 | |  |  |  |  |
| P51858 | Hepatoma-derived growth factor | HDGF | 7.08 | 1 | | 26.8 | | 4.73 | | 0.00 | 0 | | 0.41 | | 2 | | 0.00 | | 0 | | 0.16 | | 1 | | 0.00 | | 0 | | 0.00 | | 0 | | 0.00 | | 0 | | 0.00 | | 0 | | 2 | | | 0 | | 2 | |  |  |  |  |
| Q9NVP4 | Double zinc ribbon and ankyrin repeat-containing protein 1 | DZANK1 | 1.06 | 1 | | 82.1 | | 8.06 | | 0.00 | 0 | | 0.32 | | 2 | | 0.00 | | 0 | | 0.16 | | 1 | | 0.00 | | 0 | | 0.00 | | 0 | | 0.00 | | 0 | | 0.00 | | 0 | | 2 | | | 0 | | 2 | |  |  |  |  |
| Q96JJ3 | Engulfment and cell motility protein 2 | ELMO2 | 2.22 | 1 | | 82.6 | | 5.90 | | 0.00 | 0 | | 0.29 | | 2 | | 0.00 | | 0 | | 0.16 | | 1 | | 0.00 | | 0 | | 0.00 | | 0 | | 0.00 | | 0 | | 0.00 | | 0 | | 2 | | | 0 | | 2 | |  |  |  |  |
| O75937 | DnaJ homolog subfamily C member 8 | DNAJC8 | 22.53 | 2 | | 29.8 | | 9.06 | | 0.00 | 0 | | 0.14 | | 1 | | 0.00 | | 0 | | 0.16 | | 1 | | 0.00 | | 0 | | 0.00 | | 0 | | 0.00 | | 0 | | 0.00 | | 0 | | 2 | | | 0 | | 2 | |  |  |  |  |
| Q14247 | Src substrate cortactin | CTTN | 2.18 | 1 | | 61.5 | | 5.40 | | 0.00 | 0 | | 0.14 | | 1 | | 0.00 | | 0 | | 0.16 | | 1 | | 0.00 | | 0 | | 0.00 | | 0 | | 0.00 | | 0 | | 0.00 | | 0 | | 2 | | | 0 | | 2 | |  |  |  |  |
| A0A590UIU4 | Janus kinase and microtubule-interacting protein 3 | JAKMIP3 | 2.12 | 2 | | 113.9 | | 6.35 | | 0.00 | 0 | | 0.27 | | 2 | | 0.00 | | 0 | | 0.16 | | 1 | | 0.00 | | 0 | | 0.00 | | 0 | | 0.00 | | 0 | | 0.00 | | 0 | | 2 | | | 0 | | 2 | |  |  |  |  |
| P62263 | 40S ribosomal protein S14 | RPS14 | 33.11 | 6 | | 16.3 | | 10.05 | | 0.47 | 2 | | 0.00 | | 0 | | 0.00 | | 0 | | 0.00 | | 0 | | 7.75 | | 6 | | 4.70 | | 6 | | 6.08 | | 6 | | 4.74 | | 6 | | 1 | | | 4 | | 5 | |  |  |  |  |
| P61313 | 60S ribosomal protein L15 | RPL15 | 42.16 | 9 | | 24.1 | | 11.62 | | 0.32 | 2 | | 0.00 | | 0 | | 0.00 | | 0 | | 0.00 | | 0 | | 10.28 | | 5 | | 3.75 | | 5 | | 7.30 | | 6 | | 8.10 | | 6 | | 1 | | | 4 | | 5 | |  |  |  |  |
| A0A2R8YD14 | 40S ribosomal protein S24 | RPS24 | 23.64 | 4 | | 19.2 | | 10.98 | | 0.32 | 2 | | 0.00 | | 0 | | 0.00 | | 0 | | 0.00 | | 0 | | 7.72 | | 5 | | 3.10 | | 4 | | 6.51 | | 6 | | 6.28 | | 6 | | 1 | | | 4 | | 5 | |  |  |  |  |
| P62851 | 40S ribosomal protein S25 | RPS25 | 29.60 | 5 | | 13.7 | | 10.11 | | 0.32 | 2 | | 0.00 | | 0 | | 0.00 | | 0 | | 0.00 | | 0 | | 6.35 | | 5 | | 1.92 | | 3 | | 2.54 | | 6 | | 3.44 | | 6 | | 1 | | | 4 | | 5 | |  |  |  |  |
| P35221 | Catenin alpha-1 | CTNNA1 | 23.84 | 15 | | 100.0 | | 6.29 | | 0.48 | 2 | | 0.00 | | 0 | | 0.00 | | 0 | | 0.00 | | 0 | | 4.80 | | 4 | | 4.27 | | 4 | | 1.47 | | 4 | | 1.58 | | 5 | | 1 | | | 4 | | 5 | |  |  |  |  |
| P21980 | Protein-glutamine gamma-glutamyltransferase 2 | TGM2 | 23.87 | 12 | | 77.3 | | 5.22 | | 0.41 | 2 | | 0.00 | | 0 | | 0.00 | | 0 | | 0.00 | | 0 | | 3.24 | | 3 | | 0.73 | | 3 | | 0.75 | | 2 | | 0.79 | | 4 | | 1 | | | 4 | | 5 | |  |  |  |  |
| P23396 | 40S ribosomal protein S3 | RPS3 | 56.79 | 14 | | 26.7 | | 9.66 | | 0.16 | 1 | | 0.00 | | 0 | | 0.00 | | 0 | | 0.00 | | 0 | | 20.82 | | 6 | | 13.70 | | 6 | | 22.20 | | 6 | | 16.29 | | 6 | | 1 | | | 4 | | 5 | |  |  |  |  |
| Q02878 | 60S ribosomal protein L6 | RPL6 | 41.67 | 14 | | 32.7 | | 10.58 | | 0.13 | 1 | | 0.00 | | 0 | | 0.00 | | 0 | | 0.00 | | 0 | | 18.10 | | 6 | | 9.10 | | 6 | | 10.18 | | 6 | | 13.34 | | 6 | | 1 | | | 4 | | 5 | |  |  |  |  |
| P15880 | 40S ribosomal protein S2 | RPS2 | 29.01 | 8 | | 31.3 | | 10.24 | | 0.15 | 1 | | 0.00 | | 0 | | 0.00 | | 0 | | 0.00 | | 0 | | 14.14 | | 6 | | 7.81 | | 6 | | 9.24 | | 6 | | 8.40 | | 6 | | 1 | | | 4 | | 5 | |  |  |  |  |
| P62701 | 40S ribosomal protein S4, X isoform | RPS4X | 46.01 | 12 | | 29.6 | | 10.15 | | 0.15 | 1 | | 0.00 | | 0 | | 0.00 | | 0 | | 0.00 | | 0 | | 10.68 | | 6 | | 8.65 | | 6 | | 8.84 | | 6 | | 8.44 | | 6 | | 1 | | | 4 | | 5 | |  |  |  |  |
| P62753 | 40S ribosomal protein S6 | RPS6 | 22.49 | 7 | | 28.7 | | 10.84 | | 0.15 | 1 | | 0.00 | | 0 | | 0.00 | | 0 | | 0.00 | | 0 | | 9.42 | | 6 | | 6.65 | | 6 | | 7.83 | | 6 | | 5.39 | | 6 | | 1 | | | 4 | | 5 | |  |  |  |  |
| P62854 | 40S ribosomal protein S26 | RPS26 | 33.91 | 3 | | 13.0 | | 11.00 | | 0.15 | 1 | | 0.00 | | 0 | | 0.00 | | 0 | | 0.00 | | 0 | | 6.66 | | 6 | | 2.77 | | 5 | | 5.92 | | 6 | | 5.80 | | 6 | | 1 | | | 4 | | 5 | |  |  |  |  |
| P62241 | 40S ribosomal protein S8 | RPS8 | 38.46 | 9 | | 24.2 | | 10.32 | | 0.15 | 1 | | 0.00 | | 0 | | 0.00 | | 0 | | 0.00 | | 0 | | 13.19 | | 6 | | 7.30 | | 4 | | 14.34 | | 6 | | 9.06 | | 6 | | 1 | | | 4 | | 5 | |  |  |  |  |
| A0A1W2PQ51 | Probable ATP-dependent RNA helicase DDX17 | DDX17 | 12.04 | 5 | | 80.4 | | 8.37 | | 0.15 | 1 | | 0.00 | | 0 | | 0.00 | | 0 | | 0.00 | | 0 | | 4.55 | | 5 | | 3.11 | | 6 | | 2.48 | | 6 | | 1.63 | | 5 | | 1 | | | 4 | | 5 | |  |  |  |  |
| J3KTA4 | Probable ATP-dependent RNA helicase DDX5 | DDX5 | 12.38 | 4 | | 69.0 | | 8.85 | | 0.15 | 1 | | 0.00 | | 0 | | 0.00 | | 0 | | 0.00 | | 0 | | 3.99 | | 5 | | 2.48 | | 6 | | 2.93 | | 6 | | 1.63 | | 5 | | 1 | | | 4 | | 5 | |  |  |  |  |
| A0A0G2JPD3 | HLA class I histocompatibility antigen, A alpha chain | HLA-A | 24.37 | 2 | | 44.4 | | 6.20 | | 0.26 | 1 | | 0.00 | | 0 | | 0.00 | | 0 | | 0.00 | | 0 | | 2.07 | | 3 | | 3.36 | | 6 | | 4.31 | | 6 | | 5.41 | | 6 | | 1 | | | 4 | | 5 | |  |  |  |  |
| A0A140T913 | HLA class I histocompatibility antigen, A alpha chain | HLA-A | 24.80 | 2 | | 41.4 | | 6.99 | | 0.26 | 1 | | 0.00 | | 0 | | 0.00 | | 0 | | 0.00 | | 0 | | 2.07 | | 3 | | 3.36 | | 6 | | 4.16 | | 6 | | 4.84 | | 6 | | 1 | | | 4 | | 5 | |  |  |  |  |
| A0A140T9B3 | HLA class I histocompatibility antigen, C alpha chain | HLA-C | 13.35 | 1 | | 44.6 | | 7.02 | | 0.13 | 1 | | 0.00 | | 0 | | 0.00 | | 0 | | 0.00 | | 0 | | 1.71 | | 4 | | 2.54 | | 4 | | 2.97 | | 6 | | 4.15 | | 6 | | 1 | | | 4 | | 5 | |  |  |  |  |
| Q14204 | Cytoplasmic dynein 1 heavy chain 1 | DYNC1H1 | 7.68 | 22 | | 532.1 | | 6.40 | | 0.16 | 1 | | 0.00 | | 0 | | 0.00 | | 0 | | 0.00 | | 0 | | 7.76 | | 3 | | 2.22 | | 2 | | 3.22 | | 6 | | 3.25 | | 6 | | 1 | | | 4 | | 5 | |  |  |  |  |
| P46781 | 40S ribosomal protein S9 | RPS9 | 45.36 | 11 | | 22.6 | | 10.65 | | 0.16 | 1 | | 0.00 | | 0 | | 0.00 | | 0 | | 0.00 | | 0 | | 7.99 | | 5 | | 2.06 | | 2 | | 2.23 | | 5 | | 3.67 | | 4 | | 1 | | | 4 | | 5 | |  |  |  |  |
| Q86YZ3 | Hornerin | HRNR | 17.26 | 14 | | 282.2 | | 10.04 | | 0.16 | 1 | | 0.00 | | 0 | | 0.00 | | 0 | | 0.00 | | 0 | | 3.47 | | 3 | | 6.75 | | 4 | | 0.88 | | 4 | | 0.46 | | 3 | | 1 | | | 4 | | 5 | |  |  |  |  |
| O14786 | Neuropilin-1 | NRP1 | 13.43 | 8 | | 103.1 | | 5.88 | | 0.13 | 1 | | 0.00 | | 0 | | 0.00 | | 0 | | 0.00 | | 0 | | 0.16 | | 1 | | 0.16 | | 1 | | 2.49 | | 6 | | 4.89 | | 6 | | 1 | | | 4 | | 5 | |  |  |  |  |
| A0A2R8Y5A3 | Catenin beta-1 | CTNNB1 | 14.30 | 7 | | 85.6 | | 5.97 | | 0.15 | 1 | | 0.00 | | 0 | | 0.00 | | 0 | | 0.00 | | 0 | | 1.35 | | 5 | | 1.78 | | 3 | | 0.88 | | 4 | | 1.52 | | 2 | | 1 | | | 4 | | 5 | |  |  |  |  |
| Q5VY09 | Immediate early response gene 5 protein | IER5 | 3.67 | 1 | | 33.7 | | 4.96 | | 0.13 | 1 | | 0.00 | | 0 | | 0.00 | | 0 | | 0.00 | | 0 | | 0.42 | | 2 | | 0.63 | | 2 | | 0.48 | | 3 | | 1.00 | | 4 | | 1 | | | 4 | | 5 | |  |  |  |  |
| H0Y7R8 | Afadin (Fragment) | AFDN | 3.08 | 1 | | 43.3 | | 7.33 | | 0.00 | 0 | | 0.00 | | 0 | | 0.32 | | 1 | | 0.00 | | 0 | | 0.14 | | 1 | | 0.18 | | 1 | | 0.48 | | 3 | | 0.31 | | 1 | | 1 | | | 4 | | 5 | |  |  |  |  |
| Q96DA2 | Ras-related protein Rab-39B | RAB39B | 16.43 | 1 | | 24.6 | | 7.83 | | 0.00 | 0 | | 0.00 | | 0 | | 0.19 | | 1 | | 0.00 | | 0 | | 0.60 | | 3 | | 0.52 | | 3 | | 0.61 | | 3 | | 0.48 | | 3 | | 1 | | | 4 | | 5 | |  |  |  |  |
| P36578 | 60S ribosomal protein L4 | RPL4 | 29.74 | 14 | | 47.7 | | 11.06 | | 0.00 | 0 | | 0.00 | | 0 | | 0.19 | | 1 | | 0.00 | | 0 | | 13.42 | | 6 | | 5.63 | | 6 | | 6.47 | | 6 | | 7.32 | | 6 | | 1 | | | 4 | | 5 | |  |  |  |  |
| P49327 | Fatty acid synthase | FASN | 16.05 | 26 | | 273.3 | | 6.44 | | 0.00 | 0 | | 0.00 | | 0 | | 0.16 | | 1 | | 0.00 | | 0 | | 9.02 | | 3 | | 7.17 | | 3 | | 2.69 | | 5 | | 4.85 | | 6 | | 1 | | | 4 | | 5 | |  |  |  |  |
| A0A087WT12 | Glutathione peroxidase | GPX4 | 5.58 | 1 | | 26.9 | | 10.07 | | 0.00 | 0 | | 0.00 | | 0 | | 0.00 | | 0 | | 0.32 | | 2 | | 0.16 | | 1 | | 0.52 | | 3 | | 0.46 | | 3 | | 0.36 | | 1 | | 1 | | | 4 | | 5 | |  |  |  |  |
| Q13733 | Sodium/potassium-transporting ATPase subunit alpha-4 | ATP1A4 | 10.01 | 1 | | 114.1 | | 6.64 | | 0.00 | 0 | | 0.00 | | 0 | | 0.00 | | 0 | | 0.21 | | 1 | | 0.32 | | 1 | | 0.18 | | 1 | | 0.29 | | 2 | | 0.30 | | 2 | | 1 | | | 4 | | 5 | |  |  |  |  |
| O14936 | Peripheral plasma membrane protein CASK | CASK | 7.45 | 4 | | 105.1 | | 6.43 | | 0.00 | 0 | | 0.00 | | 0 | | 0.00 | | 0 | | 0.21 | | 1 | | 0.32 | | 2 | | 0.36 | | 2 | | 0.45 | | 2 | | 0.31 | | 1 | | 1 | | | 4 | | 5 | |  |  |  |  |
| P11166 | Solute carrier family 2, facilitated glucose transporter member 1 | SLC2A1 | 18.09 | 4 | | 54.0 | | 8.72 | | 0.00 | 0 | | 0.00 | | 0 | | 0.00 | | 0 | | 0.16 | | 1 | | 3.24 | | 6 | | 3.93 | | 6 | | 1.85 | | 6 | | 2.80 | | 6 | | 1 | | | 4 | | 5 | |  |  |  |  |
| P05556 | Integrin beta-1 | ITGB1 | 24.31 | 21 | | 88.4 | | 5.39 | | 0.00 | 0 | | 0.48 | | 2 | | 0.00 | | 0 | | 0.00 | | 0 | | 39.04 | | 6 | | 45.77 | | 6 | | 65.05 | | 6 | | 78.08 | | 6 | | 1 | | | 4 | | 5 | |  |  |  |  |
| P26006 | Integrin alpha-3 | ITGA3 | 26.64 | 22 | | 116.5 | | 6.77 | | 0.00 | 0 | | 0.16 | | 1 | | 0.00 | | 0 | | 0.00 | | 0 | | 29.16 | | 6 | | 30.23 | | 6 | | 41.87 | | 6 | | 46.52 | | 6 | | 1 | | | 4 | | 5 | |  |  |  |  |
| P48643 | T-complex protein 1 subunit epsilon | CCT5 | 35.86 | 13 | | 59.6 | | 5.66 | | 0.00 | 0 | | 0.32 | | 2 | | 0.00 | | 0 | | 0.00 | | 0 | | 7.47 | | 5 | | 8.29 | | 6 | | 14.37 | | 6 | | 11.89 | | 6 | | 1 | | | 4 | | 5 | |  |  |  |  |
| P35613 | Basigin | BSG | 21.56 | 6 | | 42.2 | | 5.66 | | 0.00 | 0 | | 0.16 | | 1 | | 0.00 | | 0 | | 0.00 | | 0 | | 9.15 | | 6 | | 10.35 | | 6 | | 10.44 | | 6 | | 13.40 | | 6 | | 1 | | | 4 | | 5 | |  |  |  |  |
| Q8WUM4 | Programmed cell death 6-interacting protein | PDCD6IP | 32.95 | 26 | | 96.0 | | 6.52 | | 0.00 | 0 | | 0.14 | | 1 | | 0.00 | | 0 | | 0.00 | | 0 | | 17.23 | | 6 | | 18.57 | | 5 | | 18.13 | | 6 | | 19.32 | | 6 | | 1 | | | 4 | | 5 | |  |  |  |  |
| P30050 | 60S ribosomal protein L12 | RPL12 | 24.24 | 3 | | 17.8 | | 9.42 | | 0.00 | 0 | | 0.13 | | 1 | | 0.00 | | 0 | | 0.00 | | 0 | | 2.94 | | 5 | | 1.77 | | 5 | | 3.77 | | 6 | | 2.11 | | 6 | | 1 | | | 4 | | 5 | |  |  |  |  |
| P17987 | T-complex protein 1 subunit alpha | TCP1 | 46.40 | 17 | | 60.3 | | 6.11 | | 0.00 | 0 | | 0.40 | | 2 | | 0.00 | | 0 | | 0.00 | | 0 | | 8.60 | | 3 | | 5.85 | | 5 | | 9.30 | | 6 | | 8.35 | | 6 | | 1 | | | 4 | | 5 | |  |  |  |  |
| Q9NQC3 | Reticulon-4 | RTN4 | 5.79 | 2 | | 129.9 | | 4.50 | | 0.00 | 0 | | 0.30 | | 2 | | 0.00 | | 0 | | 0.00 | | 0 | | 1.79 | | 5 | | 2.23 | | 5 | | 1.92 | | 5 | | 3.08 | | 5 | | 1 | | | 4 | | 5 | |  |  |  |  |
| F8W914 | Reticulon | RTN4 | 11.88 | 1 | | 37.1 | | 4.77 | | 0.00 | 0 | | 0.30 | | 2 | | 0.00 | | 0 | | 0.00 | | 0 | | 1.79 | | 5 | | 2.23 | | 5 | | 1.76 | | 5 | | 2.63 | | 5 | | 1 | | | 4 | | 5 | |  |  |  |  |
| Q9H4G4 | Golgi-associated plant pathogenesis-related protein 1 | GLIPR2 | 46.75 | 4 | | 17.2 | | 9.41 | | 0.00 | 0 | | 0.16 | | 1 | | 0.00 | | 0 | | 0.00 | | 0 | | 3.50 | | 3 | | 4.30 | | 4 | | 2.50 | | 6 | | 4.17 | | 6 | | 1 | | | 4 | | 5 | |  |  |  |  |
| A0A3B3IRY8 | Ephrin type-B receptor 1 | EPHB1 | 3.32 | 1 | | 117.9 | | 6.05 | | 0.00 | 0 | | 0.13 | | 1 | | 0.00 | | 0 | | 0.00 | | 0 | | 1.13 | | 2 | | 1.41 | | 4 | | 1.85 | | 6 | | 2.57 | | 6 | | 1 | | | 4 | | 5 | |  |  |  |  |
| Q99698 | Lysosomal-trafficking regulator | LYST | 0.26 | 1 | | 428.9 | | 6.61 | | 0.00 | 0 | | 0.32 | | 2 | | 0.00 | | 0 | | 0.00 | | 0 | | 0.88 | | 3 | | 0.81 | | 3 | | 2.45 | | 6 | | 2.09 | | 5 | | 1 | | | 4 | | 5 | |  |  |  |  |
| O00159 | Unconventional myosin-Ic | MYO1C | 8.00 | 6 | | 121.6 | | 9.41 | | 0.00 | 0 | | 0.13 | | 1 | | 0.00 | | 0 | | 0.00 | | 0 | | 1.55 | | 4 | | 2.15 | | 4 | | 1.51 | | 6 | | 1.11 | | 4 | | 1 | | | 4 | | 5 | |  |  |  |  |
| P20073 | Annexin A7 | ANXA7 | 9.63 | 3 | | 52.7 | | 5.68 | | 0.00 | 0 | | 0.14 | | 1 | | 0.00 | | 0 | | 0.00 | | 0 | | 0.16 | | 1 | | 1.91 | | 5 | | 1.68 | | 5 | | 2.24 | | 6 | | 1 | | | 4 | | 5 | |  |  |  |  |
| Q86YS6 | Ras-related protein Rab-43 | RAB43 | 20.28 | 1 | | 23.3 | | 5.64 | | 0.00 | 0 | | 0.16 | | 1 | | 0.00 | | 0 | | 0.00 | | 0 | | 0.92 | | 3 | | 0.93 | | 4 | | 0.78 | | 3 | | 1.15 | | 6 | | 1 | | | 4 | | 5 | |  |  |  |  |
| P17980 | 26S proteasome regulatory subunit 6A | PSMC3 | 36.67 | 11 | | 49.2 | | 5.24 | | 0.00 | 0 | | 0.13 | | 1 | | 0.00 | | 0 | | 0.00 | | 0 | | 4.37 | | 3 | | 0.79 | | 2 | | 3.11 | | 6 | | 1.48 | | 4 | | 1 | | | 4 | | 5 | |  |  |  |  |
| P41250 | Glycine--tRNA ligase | GARS1 | 24.09 | 11 | | 83.1 | | 7.03 | | 0.00 | 0 | | 0.32 | | 2 | | 0.00 | | 0 | | 0.00 | | 0 | | 3.39 | | 3 | | 1.11 | | 2 | | 3.02 | | 6 | | 1.23 | | 3 | | 1 | | | 4 | | 5 | |  |  |  |  |
| P13010 | X-ray repair cross-complementing protein 5 | XRCC5 | 29.37 | 13 | | 82.7 | | 5.81 | | 0.00 | 0 | | 0.13 | | 1 | | 0.00 | | 0 | | 0.00 | | 0 | | 10.30 | | 3 | | 3.80 | | 2 | | 5.79 | | 5 | | 3.04 | | 4 | | 1 | | | 4 | | 5 | |  |  |  |  |
| P02794 | Ferritin heavy chain | FTH1 | 44.81 | 8 | | 21.2 | | 5.55 | | 0.00 | 0 | | 0.27 | | 1 | | 0.00 | | 0 | | 0.00 | | 0 | | 0.16 | | 1 | | 2.00 | | 4 | | 1.93 | | 3 | | 8.12 | | 6 | | 1 | | | 4 | | 5 | |  |  |  |  |
| P18085 | ADP-ribosylation factor 4 | ARF4 | 34.44 | 1 | | 20.5 | | 7.14 | | 0.00 | 0 | | 0.55 | | 2 | | 0.00 | | 0 | | 0.00 | | 0 | | 0.28 | | 2 | | 1.27 | | 2 | | 2.02 | | 4 | | 2.32 | | 4 | | 1 | | | 4 | | 5 | |  |  |  |  |
| P84085 | ADP-ribosylation factor 5 | ARF5 | 34.44 | 1 | | 20.5 | | 6.79 | | 0.00 | 0 | | 0.55 | | 2 | | 0.00 | | 0 | | 0.00 | | 0 | | 0.28 | | 2 | | 1.59 | | 2 | | 1.68 | | 4 | | 2.14 | | 4 | | 1 | | | 4 | | 5 | |  |  |  |  |
| Q9H6S0 | 3'-5' RNA helicase YTHDC2 | YTHDC2 | 4.55 | 2 | | 160.1 | | 8.40 | | 0.00 | 0 | | 0.13 | | 1 | | 0.00 | | 0 | | 0.00 | | 0 | | 0.30 | | 2 | | 0.18 | | 1 | | 1.10 | | 4 | | 1.12 | | 6 | | 1 | | | 4 | | 5 | |  |  |  |  |
| A0A3B3ITT1 | Ras-related protein Rab-12 | RAB12 | 7.94 | 1 | | 36.3 | | 8.43 | | 0.00 | 0 | | 0.14 | | 1 | | 0.00 | | 0 | | 0.00 | | 0 | | 0.60 | | 3 | | 0.52 | | 3 | | 0.61 | | 3 | | 0.48 | | 3 | | 1 | | | 4 | | 5 | |  |  |  |  |
| Q96TA1 | Protein Niban 2 | NIBAN2 | 14.08 | 6 | | 84.1 | | 6.19 | | 0.00 | 0 | | 0.41 | | 2 | | 0.00 | | 0 | | 0.00 | | 0 | | 0.14 | | 1 | | 0.97 | | 3 | | 0.29 | | 2 | | 1.07 | | 3 | | 1 | | | 4 | | 5 | |  |  |  |  |
| Q92743 | Serine protease HTRA1 | HTRA1 | 21.25 | 6 | | 51.3 | | 7.83 | | 0.00 | 0 | | 0.14 | | 1 | | 0.00 | | 0 | | 0.00 | | 0 | | 1.69 | | 3 | | 1.43 | | 2 | | 1.34 | | 2 | | 1.43 | | 2 | | 1 | | | 4 | | 5 | |  |  |  |  |
| A0A0A0MTS7 | Titin | TTN | 0.68 | 12 | | 3992.2 | | 6.39 | | 0.00 | 0 | | 0.14 | | 1 | | 0.00 | | 0 | | 0.00 | | 0 | | 0.14 | | 1 | | 0.79 | | 2 | | 0.29 | | 2 | | 0.85 | | 3 | | 1 | | | 4 | | 5 | |  |  |  |  |
| Q9Y3F4 | Serine-threonine kinase receptor-associated protein | STRAP | 6.57 | 2 | | 38.4 | | 5.12 | | 0.00 | 0 | | 0.14 | | 1 | | 0.00 | | 0 | | 0.00 | | 0 | | 0.56 | | 3 | | 0.47 | | 2 | | 0.14 | | 1 | | 0.15 | | 1 | | 1 | | | 4 | | 5 | |  |  |  |  |
| Q7Z6Z7 | E3 ubiquitin-protein ligase HUWE1 | HUWE1 | 1.12 | 3 | | 481.6 | | 5.22 | | 0.00 | 0 | | 0.16 | | 1 | | 0.00 | | 0 | | 0.00 | | 0 | | 0.32 | | 2 | | 0.18 | | 1 | | 0.17 | | 1 | | 0.87 | | 3 | | 1 | | | 4 | | 5 | |  |  |  |  |
| A0A087WTG3 | Cullin-3 | CUL3 | 5.56 | 1 | | 39.1 | | 9.48 | | 0.00 | 0 | | 0.27 | | 1 | | 0.00 | | 0 | | 0.00 | | 0 | | 0.16 | | 1 | | 0.86 | | 3 | | 0.30 | | 2 | | 0.15 | | 1 | | 1 | | | 4 | | 5 | |  |  |  |  |
| P20648 | Potassium-transporting ATPase alpha chain 1 | ATP4A | 6.09 | 1 | | 114.0 | | 5.81 | | 0.00 | 0 | | 0.14 | | 1 | | 0.00 | | 0 | | 0.00 | | 0 | | 0.32 | | 1 | | 0.34 | | 2 | | 0.15 | | 1 | | 0.30 | | 1 | | 1 | | | 4 | | 5 | |  |  |  |  |
| P51991 | Heterogeneous nuclear ribonucleoprotein A3 | HNRNPA3 | 33.60 | 12 | | 39.6 | | 9.01 | | 0.32 | 2 | | 0.00 | | 0 | | 0.00 | | 0 | | 0.00 | | 0 | | 3.95 | | 3 | | 0.81 | | 3 | | 1.36 | | 6 | | 0.00 | | 0 | | 1 | | | 3 | | 4 | |  |  |  |  |
| Q6P2Q9 | Pre-mRNA-processing-splicing factor 8 | PRPF8 | 6.77 | 6 | | 273.4 | | 8.84 | | 0.47 | 2 | | 0.00 | | 0 | | 0.00 | | 0 | | 0.00 | | 0 | | 4.53 | | 4 | | 0.32 | | 1 | | 0.17 | | 1 | | 0.00 | | 0 | | 1 | | | 3 | | 4 | |  |  |  |  |
| Q96M83 | Coiled-coil domain-containing protein 7 | CCDC7 | 3.32 | 3 | | 157.4 | | 7.58 | | 0.26 | 2 | | 0.00 | | 0 | | 0.00 | | 0 | | 0.00 | | 0 | | 0.14 | | 1 | | 0.00 | | 0 | | 0.15 | | 1 | | 0.15 | | 1 | | 1 | | | 3 | | 4 | |  |  |  |  |
| Q15436 | Protein transport protein Sec23A | SEC23A | 3.66 | 2 | | 86.1 | | 7.08 | | 0.16 | 1 | | 0.00 | | 0 | | 0.00 | | 0 | | 0.00 | | 0 | | 0.00 | | 0 | | 0.47 | | 2 | | 0.60 | | 2 | | 0.92 | | 3 | | 1 | | | 3 | | 4 | |  |  |  |  |
| Q9NRN7 | L-aminoadipate-semialdehyde dehydrogenase-phosphopantetheinyl transferase | AASDHPPT | 4.85 | 1 | | 35.8 | | 6.80 | | 0.15 | 1 | | 0.00 | | 0 | | 0.00 | | 0 | | 0.00 | | 0 | | 0.56 | | 3 | | 0.16 | | 1 | | 0.30 | | 2 | | 0.00 | | 0 | | 1 | | | 3 | | 4 | |  |  |  |  |
| E7ETH6 | Zinc finger protein 587B | ZNF587B | 1.99 | 1 | | 45.5 | | 8.38 | | 0.13 | 1 | | 0.00 | | 0 | | 0.00 | | 0 | | 0.00 | | 0 | | 0.14 | | 1 | | 0.34 | | 2 | | 0.00 | | 0 | | 0.30 | | 2 | | 1 | | | 3 | | 4 | |  |  |  |  |
| Q8WXX0 | Dynein heavy chain 7, axonemal | DNAH7 | 3.58 | 6 | | 460.9 | | 6.00 | | 0.13 | 1 | | 0.00 | | 0 | | 0.00 | | 0 | | 0.00 | | 0 | | 0.00 | | 0 | | 0.23 | | 1 | | 0.46 | | 3 | | 0.18 | | 1 | | 1 | | | 3 | | 4 | |  |  |  |  |
| Q7Z776 | POLR1D protein | POLR1D | 45.45 | 2 | | 3.8 | | 4.96 | | 0.13 | 1 | | 0.00 | | 0 | | 0.00 | | 0 | | 0.00 | | 0 | | 0.16 | | 1 | | 0.54 | | 2 | | 0.15 | | 1 | | 0.00 | | 0 | | 1 | | | 3 | | 4 | |  |  |  |  |
| P06132 | Uroporphyrinogen decarboxylase | UROD | 18.53 | 3 | | 40.8 | | 6.14 | | 0.16 | 1 | | 0.00 | | 0 | | 0.00 | | 0 | | 0.00 | | 0 | | 0.00 | | 0 | | 0.16 | | 1 | | 0.14 | | 1 | | 0.45 | | 2 | | 1 | | | 3 | | 4 | |  |  |  |  |
| P42262 | Glutamate receptor 2 | GRIA2 | 3.96 | 1 | | 98.8 | | 7.58 | | 0.28 | 1 | | 0.00 | | 0 | | 0.00 | | 0 | | 0.00 | | 0 | | 0.14 | | 1 | | 0.34 | | 2 | | 0.14 | | 1 | | 0.00 | | 0 | | 1 | | | 3 | | 4 | |  |  |  |  |
| Q8N961 | Ankyrin repeat and BTB/POZ domain-containing protein 2 | ABTB2 | 3.80 | 2 | | 113.6 | | 6.29 | | 0.16 | 1 | | 0.00 | | 0 | | 0.00 | | 0 | | 0.00 | | 0 | | 0.14 | | 1 | | 0.00 | | 0 | | 0.15 | | 1 | | 0.18 | | 1 | | 1 | | | 3 | | 4 | |  |  |  |  |
| Q8IUX7 | Adipocyte enhancer-binding protein 1 | AEBP1 | 6.99 | 5 | | 130.8 | | 5.11 | | 0.00 | 0 | | 0.00 | | 0 | | 0.44 | | 2 | | 0.00 | | 0 | | 0.16 | | 1 | | 0.34 | | 2 | | 1.50 | | 2 | | 0.00 | | 0 | | 1 | | | 3 | | 4 | |  |  |  |  |
| P78527 | DNA-dependent protein kinase catalytic subunit | PRKDC | 2.91 | 8 | | 468.8 | | 7.12 | | 0.00 | 0 | | 0.00 | | 0 | | 0.38 | | 2 | | 0.00 | | 0 | | 1.97 | | 3 | | 0.18 | | 1 | | 0.15 | | 1 | | 0.00 | | 0 | | 1 | | | 3 | | 4 | |  |  |  |  |
| O43543 | DNA repair protein XRCC2 | XRCC2 | 3.57 | 1 | | 31.9 | | 6.04 | | 0.00 | 0 | | 0.00 | | 0 | | 0.38 | | 1 | | 0.00 | | 0 | | 0.16 | | 1 | | 0.00 | | 0 | | 0.31 | | 2 | | 0.81 | | 5 | | 1 | | | 3 | | 4 | |  |  |  |  |
| A0A0U1RRG4 | UDP-galactose translocator (Fragment) | SLC35A2 | 16.75 | 2 | | 20.0 | | 10.07 | | 0.00 | 0 | | 0.00 | | 0 | | 0.19 | | 1 | | 0.00 | | 0 | | 0.42 | | 2 | | 0.00 | | 0 | | 0.47 | | 3 | | 0.76 | | 4 | | 1 | | | 3 | | 4 | |  |  |  |  |
| Q7KZF4 | Staphylococcal nuclease domain-containing protein 1 | SND1 | 4.51 | 3 | | 101.9 | | 7.17 | | 0.00 | 0 | | 0.00 | | 0 | | 0.19 | | 1 | | 0.00 | | 0 | | 0.85 | | 3 | | 0.16 | | 1 | | 0.14 | | 1 | | 0.00 | | 0 | | 1 | | | 3 | | 4 | |  |  |  |  |
| Q5TCS8 | Adenylate kinase 9 | AK9 | 1.78 | 2 | | 221.3 | | 5.01 | | 0.00 | 0 | | 0.00 | | 0 | | 0.19 | | 1 | | 0.00 | | 0 | | 0.00 | | 0 | | 0.18 | | 1 | | 0.15 | | 1 | | 0.33 | | 2 | | 1 | | | 3 | | 4 | |  |  |  |  |
| Q86SJ2 | Amphoterin-induced protein 2 | AMIGO2 | 5.56 | 2 | | 57.9 | | 8.40 | | 0.00 | 0 | | 0.00 | | 0 | | 0.16 | | 1 | | 0.00 | | 0 | | 0.48 | | 2 | | 0.55 | | 2 | | 0.00 | | 0 | | 0.61 | | 2 | | 1 | | | 3 | | 4 | |  |  |  |  |
| Q9Y2Z9 | Ubiquinone biosynthesis monooxygenase COQ6, mitochondrial | COQ6 | 8.12 | 2 | | 50.8 | | 7.30 | | 0.00 | 0 | | 0.00 | | 0 | | 0.16 | | 1 | | 0.00 | | 0 | | 0.14 | | 1 | | 0.00 | | 0 | | 0.46 | | 3 | | 0.15 | | 1 | | 1 | | | 3 | | 4 | |  |  |  |  |
| P46821 | Microtubule-associated protein 1B | MAP1B | 2.43 | 2 | | 270.5 | | 4.81 | | 0.00 | 0 | | 0.00 | | 0 | | 0.16 | | 1 | | 0.00 | | 0 | | 0.32 | | 2 | | 0.18 | | 1 | | 0.00 | | 0 | | 0.15 | | 1 | | 1 | | | 3 | | 4 | |  |  |  |  |
| J3KQV8 | Synaptojanin-1 | SYNJ1 | 1.99 | 3 | | 177.4 | | 8.22 | | 0.00 | 0 | | 0.00 | | 0 | | 0.16 | | 1 | | 0.00 | | 0 | | 0.00 | | 0 | | 0.18 | | 1 | | 0.14 | | 1 | | 0.15 | | 1 | | 1 | | | 3 | | 4 | |  |  |  |  |
| Q9BV40 | Vesicle-associated membrane protein 8 | VAMP8 | 58.00 | 4 | | 11.4 | | 7.34 | | 0.00 | 0 | | 0.00 | | 0 | | 0.00 | | 0 | | 0.21 | | 1 | | 1.78 | | 2 | | 1.81 | | 2 | | 0.15 | | 1 | | 0.00 | | 0 | | 1 | | | 3 | | 4 | |  |  |  |  |
| A0A087WYX9 | Collagen alpha-2(V) chain | COL5A2 | 7.28 | 1 | | 106.9 | | 6.33 | | 0.00 | 0 | | 0.45 | | 2 | | 0.00 | | 0 | | 0.00 | | 0 | | 0.00 | | 0 | | 2.03 | | 3 | | 1.61 | | 4 | | 1.20 | | 3 | | 1 | | | 3 | | 4 | |  |  |  |  |
| P63313 | Thymosin beta-10 | TMSB10 | 45.45 | 3 | | 5.0 | | 5.36 | | 0.00 | 0 | | 0.14 | | 1 | | 0.00 | | 0 | | 0.00 | | 0 | | 2.50 | | 3 | | 1.43 | | 4 | | 0.29 | | 2 | | 0.00 | | 0 | | 1 | | | 3 | | 4 | |  |  |  |  |
| P20290 | Transcription factor BTF3 | BTF3 | 20.39 | 3 | | 22.2 | | 9.38 | | 0.00 | 0 | | 0.13 | | 1 | | 0.00 | | 0 | | 0.00 | | 0 | | 1.41 | | 3 | | 0.00 | | 0 | | 0.67 | | 2 | | 0.54 | | 2 | | 1 | | | 3 | | 4 | |  |  |  |  |
| O43829 | Zinc finger and BTB domain-containing protein 14 | ZBTB14 | 4.45 | 1 | | 50.9 | | 6.04 | | 0.00 | 0 | | 0.16 | | 1 | | 0.00 | | 0 | | 0.00 | | 0 | | 0.16 | | 1 | | 1.01 | | 4 | | 0.17 | | 1 | | 0.00 | | 0 | | 1 | | | 3 | | 4 | |  |  |  |  |
| A0A1W2PRA1 | Stromal interaction molecule 2 | STIM2 | 1.19 | 1 | | 84.8 | | 6.76 | | 0.00 | 0 | | 0.14 | | 1 | | 0.00 | | 0 | | 0.00 | | 0 | | 0.00 | | 0 | | 0.34 | | 2 | | 0.29 | | 2 | | 0.18 | | 1 | | 1 | | | 3 | | 4 | |  |  |  |  |
| Q8TDC3 | Serine/threonine-protein kinase BRSK1 | BRSK1 | 6.30 | 2 | | 85.0 | | 9.32 | | 0.00 | 0 | | 0.14 | | 1 | | 0.00 | | 0 | | 0.00 | | 0 | | 0.14 | | 1 | | 0.32 | | 1 | | 0.46 | | 2 | | 0.00 | | 0 | | 1 | | | 3 | | 4 | |  |  |  |  |
| Q8IZF6 | Adhesion G-protein coupled receptor G4 | ADGRG4 | 0.23 | 1 | | 333.2 | | 6.21 | | 0.00 | 0 | | 0.14 | | 1 | | 0.00 | | 0 | | 0.00 | | 0 | | 0.00 | | 0 | | 0.16 | | 1 | | 0.17 | | 1 | | 0.72 | | 2 | | 1 | | | 3 | | 4 | |  |  |  |  |
| Q86Y46 | Keratin, type II cytoskeletal 73 | KRT73 | 12.78 | 2 | | 58.9 | | 7.23 | | 0.48 | 2 | | 0.00 | | 0 | | 0.00 | | 0 | | 0.00 | | 0 | | 0.14 | | 1 | | 1.57 | | 3 | | 0.00 | | 0 | | 0.00 | | 0 | | 1 | | | 2 | | 3 | |  |  |  |  |
| Q14CN4 | Keratin, type II cytoskeletal 72 | KRT72 | 9.59 | 1 | | 55.8 | | 6.89 | | 0.48 | 2 | | 0.00 | | 0 | | 0.00 | | 0 | | 0.00 | | 0 | | 0.14 | | 1 | | 1.41 | | 2 | | 0.00 | | 0 | | 0.00 | | 0 | | 1 | | | 2 | | 3 | |  |  |  |  |
| Q16630 | Cleavage and polyadenylation specificity factor subunit 6 | CPSF6 | 5.81 | 2 | | 59.2 | | 7.15 | | 0.43 | 2 | | 0.00 | | 0 | | 0.00 | | 0 | | 0.00 | | 0 | | 0.56 | | 1 | | 0.00 | | 0 | | 0.00 | | 0 | | 0.36 | | 1 | | 1 | | | 2 | | 3 | |  |  |  |  |
| A0A087X097 | Cadherin-23 | CDH23 | 1.34 | 2 | | 369.3 | | 4.67 | | 0.41 | 2 | | 0.00 | | 0 | | 0.00 | | 0 | | 0.00 | | 0 | | 0.16 | | 1 | | 0.00 | | 0 | | 0.00 | | 0 | | 0.18 | | 1 | | 1 | | | 2 | | 3 | |  |  |  |  |
| Q6IAA8 | Ragulator complex protein LAMTOR1 | LAMTOR1 | 18.01 | 2 | | 17.7 | | 5.15 | | 0.28 | 1 | | 0.00 | | 0 | | 0.00 | | 0 | | 0.00 | | 0 | | 0.16 | | 1 | | 1.42 | | 4 | | 0.00 | | 0 | | 0.00 | | 0 | | 1 | | | 2 | | 3 | |  |  |  |  |
| Q06210 | Glutamine--fructose-6-phosphate aminotransferase [isomerizing] 1 | GFPT1 | 9.44 | 4 | | 78.8 | | 7.11 | | 0.28 | 1 | | 0.00 | | 0 | | 0.00 | | 0 | | 0.00 | | 0 | | 1.55 | | 3 | | 0.16 | | 1 | | 0.00 | | 0 | | 0.00 | | 0 | | 1 | | | 2 | | 3 | |  |  |  |  |
| Q96L42 | Potassium voltage-gated channel subfamily H member 8 | KCNH8 | 10.12 | 3 | | 123.7 | | 6.92 | | 0.13 | 1 | | 0.00 | | 0 | | 0.00 | | 0 | | 0.00 | | 0 | | 0.14 | | 1 | | 0.16 | | 1 | | 0.00 | | 0 | | 0.00 | | 0 | | 1 | | | 2 | | 3 | |  |  |  |  |
| Q96JI7 | Spatacsin | SPG11 | 2.42 | 3 | | 278.7 | | 5.97 | | 0.28 | 1 | | 0.00 | | 0 | | 0.00 | | 0 | | 0.00 | | 0 | | 0.16 | | 1 | | 0.16 | | 1 | | 0.00 | | 0 | | 0.00 | | 0 | | 1 | | | 2 | | 3 | |  |  |  |  |
| P42336 | Phosphatidylinositol 4,5-bisphosphate 3-kinase catalytic subunit alpha isoform | PIK3CA | 5.15 | 2 | | 124.2 | | 7.23 | | 0.28 | 1 | | 0.00 | | 0 | | 0.00 | | 0 | | 0.00 | | 0 | | 0.16 | | 1 | | 0.00 | | 0 | | 0.15 | | 1 | | 0.00 | | 0 | | 1 | | | 2 | | 3 | |  |  |  |  |
| A0A0A0MRW5 | Dynein regulatory complex subunit 6 | FBXL13 | 6.00 | 3 | | 78.5 | | 8.87 | | 0.28 | 1 | | 0.00 | | 0 | | 0.00 | | 0 | | 0.00 | | 0 | | 0.16 | | 1 | | 0.00 | | 0 | | 0.00 | | 0 | | 0.15 | | 1 | | 1 | | | 2 | | 3 | |  |  |  |  |
| O14939 | Phospholipase D2 | PLD2 | 0.75 | 1 | | 105.9 | | 7.64 | | 0.15 | 1 | | 0.00 | | 0 | | 0.00 | | 0 | | 0.00 | | 0 | | 0.00 | | 0 | | 0.16 | | 1 | | 0.17 | | 1 | | 0.00 | | 0 | | 1 | | | 2 | | 3 | |  |  |  |  |
| Q8N442 | Translation factor GUF1, mitochondrial | GUF1 | 1.79 | 1 | | 74.3 | | 8.59 | | 0.15 | 1 | | 0.00 | | 0 | | 0.00 | | 0 | | 0.00 | | 0 | | 0.00 | | 0 | | 0.16 | | 1 | | 0.00 | | 0 | | 0.15 | | 1 | | 1 | | | 2 | | 3 | |  |  |  |  |
| Q12851 | Mitogen-activated protein kinase kinase kinase kinase 2 | MAP4K2 | 3.78 | 2 | | 91.5 | | 6.34 | | 0.16 | 1 | | 0.00 | | 0 | | 0.00 | | 0 | | 0.00 | | 0 | | 0.00 | | 0 | | 0.18 | | 1 | | 0.14 | | 1 | | 0.00 | | 0 | | 1 | | | 2 | | 3 | |  |  |  |  |
| A0A1C7CYW8 | Dynein heavy chain 10, axonemal | DNAH10 | 1.72 | 3 | | 521.6 | | 5.80 | | 0.16 | 1 | | 0.00 | | 0 | | 0.00 | | 0 | | 0.00 | | 0 | | 0.00 | | 0 | | 0.24 | | 1 | | 0.14 | | 1 | | 0.00 | | 0 | | 1 | | | 2 | | 3 | |  |  |  |  |
| P49454 | Centromere protein F | CENPF | 1.00 | 2 | | 357.3 | | 5.10 | | 0.13 | 1 | | 0.00 | | 0 | | 0.00 | | 0 | | 0.00 | | 0 | | 0.00 | | 0 | | 0.00 | | 0 | | 0.17 | | 1 | | 0.15 | | 1 | | 1 | | | 2 | | 3 | |  |  |  |  |
| Q96RL7 | Vacuolar protein sorting-associated protein 13A | VPS13A | 2.08 | 3 | | 360.0 | | 6.33 | | 0.13 | 1 | | 0.00 | | 0 | | 0.00 | | 0 | | 0.00 | | 0 | | 0.00 | | 0 | | 0.00 | | 0 | | 0.14 | | 1 | | 0.15 | | 1 | | 1 | | | 2 | | 3 | |  |  |  |  |
| Q9BY76 | Angiopoietin-related protein 4 | ANGPTL4 | 8.13 | 2 | | 45.2 | | 8.85 | | 0.00 | 0 | | 0.00 | | 0 | | 0.34 | | 2 | | 0.00 | | 0 | | 0.00 | | 0 | | 0.48 | | 2 | | 0.00 | | 0 | | 0.94 | | 3 | | 1 | | | 2 | | 3 | |  |  |  |  |
| A0A2R8YH03 | Protein TASOR 2 | TASOR2 | 4.08 | 4 | | 293.4 | | 5.95 | | 0.00 | 0 | | 0.00 | | 0 | | 0.22 | | 1 | | 0.00 | | 0 | | 0.14 | | 1 | | 0.00 | | 0 | | 0.30 | | 2 | | 0.00 | | 0 | | 1 | | | 2 | | 3 | |  |  |  |  |
| Q03001 | Dystonin | DST | 0.65 | 3 | | 860.1 | | 5.25 | | 0.00 | 0 | | 0.00 | | 0 | | 0.22 | | 1 | | 0.00 | | 0 | | 0.00 | | 0 | | 0.18 | | 1 | | 0.00 | | 0 | | 0.15 | | 1 | | 1 | | | 2 | | 3 | |  |  |  |  |
| A0A5F9ZH43 | Xin actin-binding repeat-containing protein 2 | XIRP2 | 1.54 | 3 | | 435.6 | | 6.52 | | 0.00 | 0 | | 0.00 | | 0 | | 0.16 | | 1 | | 0.00 | | 0 | | 0.16 | | 1 | | 0.16 | | 1 | | 0.00 | | 0 | | 0.00 | | 0 | | 1 | | | 2 | | 3 | |  |  |  |  |
| P15813 | Antigen-presenting glycoprotein CD1d | CD1D | 20.30 | 3 | | 37.7 | | 8.16 | | 0.00 | 0 | | 0.00 | | 0 | | 0.16 | | 1 | | 0.00 | | 0 | | 0.14 | | 1 | | 0.23 | | 1 | | 0.00 | | 0 | | 0.00 | | 0 | | 1 | | | 2 | | 3 | |  |  |  |  |
| Q9BYT8 | Neurolysin, mitochondrial | NLN | 8.24 | 4 | | 80.6 | | 6.64 | | 0.00 | 0 | | 0.00 | | 0 | | 0.00 | | 0 | | 0.33 | | 2 | | 0.00 | | 0 | | 0.00 | | 0 | | 0.14 | | 1 | | 0.15 | | 1 | | 1 | | | 2 | | 3 | |  |  |  |  |
| Q15366 | Poly(rC)-binding protein 2 | PCBP2 | 13.15 | 3 | | 38.6 | | 6.79 | | 0.00 | 0 | | 0.00 | | 0 | | 0.00 | | 0 | | 0.21 | | 1 | | 1.27 | | 3 | | 0.00 | | 0 | | 0.17 | | 1 | | 0.00 | | 0 | | 1 | | | 2 | | 3 | |  |  |  |  |
| Q9P265 | Disco-interacting protein 2 homolog B | DIP2B | 2.35 | 2 | | 171.4 | | 8.09 | | 0.00 | 0 | | 0.00 | | 0 | | 0.00 | | 0 | | 0.21 | | 1 | | 0.16 | | 1 | | 0.18 | | 1 | | 0.00 | | 0 | | 0.00 | | 0 | | 1 | | | 2 | | 3 | |  |  |  |  |
| D6RER5 | Septin-11 | SEPTIN11 | 5.32 | 1 | | 49.8 | | 6.68 | | 0.00 | 0 | | 0.00 | | 0 | | 0.00 | | 0 | | 0.17 | | 1 | | 0.14 | | 1 | | 0.00 | | 0 | | 0.00 | | 0 | | 0.18 | | 1 | | 1 | | | 2 | | 3 | |  |  |  |  |
| Q9UII4 | E3 ISG15--protein ligase HERC5 | HERC5 | 3.71 | 2 | | 116.8 | | 7.65 | | 0.00 | 0 | | 0.00 | | 0 | | 0.00 | | 0 | | 0.17 | | 1 | | 0.14 | | 1 | | 0.00 | | 0 | | 0.00 | | 0 | | 0.15 | | 1 | | 1 | | | 2 | | 3 | |  |  |  |  |
| Q92599 | Septin-8 | SEPTIN8 | 4.55 | 1 | | 55.7 | | 6.28 | | 0.00 | 0 | | 0.00 | | 0 | | 0.00 | | 0 | | 0.17 | | 1 | | 0.00 | | 0 | | 0.00 | | 0 | | 0.14 | | 1 | | 0.18 | | 1 | | 1 | | | 2 | | 3 | |  |  |  |  |
| A6NC48 | ADP-ribosyl cyclase/cyclic ADP-ribose hydrolase 2 | BST1 | 13.21 | 2 | | 37.5 | | 7.97 | | 0.00 | 0 | | 0.00 | | 0 | | 0.00 | | 0 | | 0.16 | | 1 | | 0.00 | | 0 | | 0.00 | | 0 | | 0.15 | | 1 | | 0.30 | | 2 | | 1 | | | 2 | | 3 | |  |  |  |  |
| Q99487 | Platelet-activating factor acetylhydrolase 2, cytoplasmic | PAFAH2 | 7.91 | 2 | | 44.0 | | 6.89 | | 0.00 | 0 | | 0.00 | | 0 | | 0.00 | | 0 | | 0.16 | | 1 | | 0.14 | | 1 | | 0.00 | | 0 | | 0.00 | | 0 | | 0.15 | | 1 | | 1 | | | 2 | | 3 | |  |  |  |  |
| Q9P2F8 | Sial-induced proliferation-associated 1-like protein 2 | SIPA1L2 | 1.86 | 2 | | 190.3 | | 6.77 | | 0.00 | 0 | | 0.00 | | 0 | | 0.00 | | 0 | | 0.16 | | 1 | | 0.00 | | 0 | | 0.00 | | 0 | | 0.47 | | 3 | | 0.15 | | 1 | | 1 | | | 2 | | 3 | |  |  |  |  |
| E7ESW6 | WD repeat-containing protein 87 | WDR87 | 2.64 | 2 | | 337.6 | | 7.25 | | 0.00 | 0 | | 0.00 | | 0 | | 0.00 | | 0 | | 0.16 | | 1 | | 0.00 | | 0 | | 0.32 | | 1 | | 0.00 | | 0 | | 0.15 | | 1 | | 1 | | | 2 | | 3 | |  |  |  |  |
| A0A1B0GVU3 | Regulator of G-protein-sialing 9 | RGS9 | 6.82 | 2 | | 77.0 | | 9.39 | | 0.00 | 0 | | 0.00 | | 0 | | 0.00 | | 0 | | 0.16 | | 1 | | 0.00 | | 0 | | 0.18 | | 1 | | 0.00 | | 0 | | 0.15 | | 1 | | 1 | | | 2 | | 3 | |  |  |  |  |
| A0A075B752 | Annexin | ANXA8L1 | 11.51 | 3 | | 40.7 | | 6.04 | | 0.00 | 0 | | 0.00 | | 0 | | 0.00 | | 0 | | 0.16 | | 1 | | 0.00 | | 0 | | 0.00 | | 0 | | 0.34 | | 1 | | 0.15 | | 1 | | 1 | | | 2 | | 3 | |  |  |  |  |
| P26639 | Threonine--tRNA ligase 1, cytoplasmic | TARS1 | 4.43 | 3 | | 83.4 | | 6.67 | | 0.00 | 0 | | 0.00 | | 0 | | 0.00 | | 0 | | 0.16 | | 1 | | 0.00 | | 0 | | 0.00 | | 0 | | 0.14 | | 1 | | 0.15 | | 1 | | 1 | | | 2 | | 3 | |  |  |  |  |
| O43175 | D-3-phosphoglycerate dehydrogenase | PHGDH | 13.13 | 4 | | 56.6 | | 6.71 | | 0.00 | 0 | | 0.16 | | 1 | | 0.00 | | 0 | | 0.00 | | 0 | | 0.14 | | 1 | | 0.00 | | 0 | | 1.71 | | 4 | | 0.00 | | 0 | | 1 | | | 2 | | 3 | |  |  |  |  |
| P25705 | ATP synthase subunit alpha, mitochondrial | ATP5F1A | 13.20 | 5 | | 59.7 | | 9.13 | | 0.00 | 0 | | 0.13 | | 1 | | 0.00 | | 0 | | 0.00 | | 0 | | 0.00 | | 0 | | 0.47 | | 2 | | 0.00 | | 0 | | 1.97 | | 2 | | 1 | | | 2 | | 3 | |  |  |  |  |
| P54136 | Arginine--tRNA ligase, cytoplasmic | RARS1 | 8.03 | 4 | | 75.3 | | 6.68 | | 0.00 | 0 | | 0.13 | | 1 | | 0.00 | | 0 | | 0.00 | | 0 | | 0.57 | | 3 | | 0.00 | | 0 | | 0.17 | | 1 | | 0.00 | | 0 | | 1 | | | 2 | | 3 | |  |  |  |  |
| Q15365 | Poly(rC)-binding protein 1 | PCBP1 | 8.99 | 2 | | 37.5 | | 7.09 | | 0.00 | 0 | | 0.14 | | 1 | | 0.00 | | 0 | | 0.00 | | 0 | | 0.42 | | 3 | | 0.00 | | 0 | | 0.17 | | 1 | | 0.00 | | 0 | | 1 | | | 2 | | 3 | |  |  |  |  |
| A0A0C4DFN2 | Glucokinase (Hexokinase 4) regulator | GCKR | 2.56 | 1 | | 68.7 | | 6.70 | | 0.00 | 0 | | 0.16 | | 1 | | 0.00 | | 0 | | 0.00 | | 0 | | 0.00 | | 0 | | 0.62 | | 3 | | 0.14 | | 1 | | 0.00 | | 0 | | 1 | | | 2 | | 3 | |  |  |  |  |
| Q13085 | Acetyl-CoA carboxylase 1 | ACACA | 2.98 | 3 | | 265.4 | | 6.37 | | 0.00 | 0 | | 0.13 | | 1 | | 0.00 | | 0 | | 0.00 | | 0 | | 0.00 | | 0 | | 0.18 | | 1 | | 0.00 | | 0 | | 0.46 | | 2 | | 1 | | | 2 | | 3 | |  |  |  |  |
| Q6P2I3 | Fumarylacetoacetate hydrolase domain-containing protein 2B | FAHD2B | 2.23 | 1 | | 34.6 | | 7.75 | | 0.00 | 0 | | 0.16 | | 1 | | 0.00 | | 0 | | 0.00 | | 0 | | 0.00 | | 0 | | 0.00 | | 0 | | 0.15 | | 1 | | 0.48 | | 2 | | 1 | | | 2 | | 3 | |  |  |  |  |
| Q86X10 | Ral GTPase-activating protein subunit beta | RALGAPB | 0.60 | 1 | | 166.7 | | 6.79 | | 0.00 | 0 | | 0.14 | | 1 | | 0.00 | | 0 | | 0.00 | | 0 | | 0.14 | | 1 | | 0.32 | | 2 | | 0.00 | | 0 | | 0.00 | | 0 | | 1 | | | 2 | | 3 | |  |  |  |  |
| Q9NTI5 | Sister chromatid cohesion protein PDS5 homolog B | PDS5B | 2.42 | 2 | | 164.6 | | 8.47 | | 0.00 | 0 | | 0.32 | | 2 | | 0.00 | | 0 | | 0.00 | | 0 | | 0.14 | | 1 | | 0.00 | | 0 | | 0.00 | | 0 | | 0.15 | | 1 | | 1 | | | 2 | | 3 | |  |  |  |  |
| Q702N8 | Xin actin-binding repeat-containing protein 1 | XIRP1 | 4.29 | 4 | | 198.4 | | 6.15 | | 0.00 | 0 | | 0.14 | | 1 | | 0.00 | | 0 | | 0.00 | | 0 | | 0.00 | | 0 | | 0.16 | | 1 | | 0.30 | | 2 | | 0.00 | | 0 | | 1 | | | 2 | | 3 | |  |  |  |  |
| A6NHR9 | Structural maintenance of chromosomes flexible hinge domain-containing protein 1 | SMCHD1 | 5.14 | 4 | | 226.2 | | 7.30 | | 0.00 | 0 | | 0.14 | | 1 | | 0.00 | | 0 | | 0.00 | | 0 | | 0.00 | | 0 | | 0.16 | | 1 | | 0.31 | | 2 | | 0.00 | | 0 | | 1 | | | 2 | | 3 | |  |  |  |  |
| Q6T4R5 | Nance-Horan syndrome protein | NHS | 3.51 | 2 | | 179.0 | | 6.86 | | 0.00 | 0 | | 0.14 | | 1 | | 0.00 | | 0 | | 0.00 | | 0 | | 0.28 | | 1 | | 0.00 | | 0 | | 0.00 | | 0 | | 0.15 | | 1 | | 1 | | | 2 | | 3 | |  |  |  |  |
| P06576 | ATP synthase subunit beta, mitochondrial | ATP5F1B | 13.23 | 4 | | 56.5 | | 5.40 | | 0.00 | 0 | | 0.16 | | 1 | | 0.00 | | 0 | | 0.00 | | 0 | | 0.00 | | 0 | | 0.23 | | 1 | | 0.00 | | 0 | | 0.30 | | 1 | | 1 | | | 2 | | 3 | |  |  |  |  |
| Q8N0V4 | Leucine-rich repeat LGI family member 2 | LGI2 | 3.12 | 2 | | 62.3 | | 6.93 | | 0.00 | 0 | | 0.14 | | 1 | | 0.00 | | 0 | | 0.00 | | 0 | | 0.16 | | 1 | | 0.18 | | 1 | | 0.00 | | 0 | | 0.00 | | 0 | | 1 | | | 2 | | 3 | |  |  |  |  |
| Q8NDG6 | ATP-dependent RNA helicase TDRD9 | TDRD9 | 6.87 | 3 | | 155.6 | | 7.05 | | 0.00 | 0 | | 0.13 | | 1 | | 0.00 | | 0 | | 0.00 | | 0 | | 0.16 | | 1 | | 0.00 | | 0 | | 0.00 | | 0 | | 0.15 | | 1 | | 1 | | | 2 | | 3 | |  |  |  |  |
| Q14692 | Ribosome biogenesis protein BMS1 homolog | BMS1 | 2.18 | 2 | | 145.7 | | 6.44 | | 0.00 | 0 | | 0.16 | | 1 | | 0.00 | | 0 | | 0.00 | | 0 | | 0.00 | | 0 | | 0.16 | | 1 | | 0.17 | | 1 | | 0.00 | | 0 | | 1 | | | 2 | | 3 | |  |  |  |  |
| Q9C0G6 | Dynein heavy chain 6, axonemal | DNAH6 | 0.99 | 3 | | 475.7 | | 6.00 | | 0.00 | 0 | | 0.13 | | 1 | | 0.00 | | 0 | | 0.00 | | 0 | | 0.00 | | 0 | | 0.16 | | 1 | | 0.00 | | 0 | | 0.15 | | 1 | | 1 | | | 2 | | 3 | |  |  |  |  |
| Q12955 | Ankyrin-3 | ANK3 | 1.55 | 3 | | 480.1 | | 6.49 | | 0.00 | 0 | | 0.14 | | 1 | | 0.00 | | 0 | | 0.00 | | 0 | | 0.00 | | 0 | | 0.16 | | 1 | | 0.00 | | 0 | | 0.15 | | 1 | | 1 | | | 2 | | 3 | |  |  |  |  |
| A0A087WVP4 | Smoothelin | SMTN | 2.06 | 1 | | 104.8 | | 8.73 | | 0.00 | 0 | | 0.16 | | 1 | | 0.00 | | 0 | | 0.00 | | 0 | | 0.00 | | 0 | | 0.18 | | 1 | | 0.15 | | 1 | | 0.00 | | 0 | | 1 | | | 2 | | 3 | |  |  |  |  |
| Q16531 | DNA damage-binding protein 1 | DDB1 | 5.53 | 3 | | 126.9 | | 5.26 | | 0.00 | 0 | | 0.13 | | 1 | | 0.00 | | 0 | | 0.00 | | 0 | | 0.00 | | 0 | | 0.00 | | 0 | | 0.17 | | 1 | | 0.18 | | 1 | | 1 | | | 2 | | 3 | |  |  |  |  |
| Q07954 | Prolow-density lipoprotein receptor-related protein 1 | LRP1 | 2.00 | 3 | | 504.3 | | 5.39 | | 0.00 | 0 | | 0.14 | | 1 | | 0.00 | | 0 | | 0.00 | | 0 | | 0.00 | | 0 | | 0.00 | | 0 | | 0.15 | | 1 | | 0.15 | | 1 | | 1 | | | 2 | | 3 | |  |  |  |  |
| O94823 | Probable phospholipid-transporting ATPase VB | ATP10B | 4.18 | 3 | | 165.3 | | 6.89 | | 0.32 | 2 | | 0.00 | | 0 | | 0.00 | | 0 | | 0.00 | | 0 | | 0.00 | | 0 | | 0.42 | | 2 | | 0.00 | | 0 | | 0.00 | | 0 | | 1 | | | 1 | | 2 | |  |  |  |  |
| Q96P70 | Importin-9 | IPO9 | 2.69 | 1 | | 115.9 | | 4.81 | | 0.32 | 2 | | 0.00 | | 0 | | 0.00 | | 0 | | 0.00 | | 0 | | 0.14 | | 1 | | 0.00 | | 0 | | 0.00 | | 0 | | 0.00 | | 0 | | 1 | | | 1 | | 2 | |  |  |  |  |
| P61289 | Proteasome activator complex subunit 3 | PSME3 | 18.11 | 3 | | 29.5 | | 5.95 | | 0.13 | 1 | | 0.00 | | 0 | | 0.00 | | 0 | | 0.00 | | 0 | | 0.00 | | 0 | | 1.11 | | 2 | | 0.00 | | 0 | | 0.00 | | 0 | | 1 | | | 1 | | 2 | |  |  |  |  |
| O14980 | Exportin-1 | XPO1 | 5.88 | 3 | | 123.3 | | 6.06 | | 0.15 | 1 | | 0.00 | | 0 | | 0.00 | | 0 | | 0.00 | | 0 | | 0.71 | | 2 | | 0.00 | | 0 | | 0.00 | | 0 | | 0.00 | | 0 | | 1 | | | 1 | | 2 | |  |  |  |  |
| P39060 | Collagen alpha-1(XVIII) chain | COL18A1 | 4.16 | 4 | | 178.1 | | 6.01 | | 0.13 | 1 | | 0.00 | | 0 | | 0.00 | | 0 | | 0.00 | | 0 | | 0.00 | | 0 | | 1.17 | | 2 | | 0.00 | | 0 | | 0.00 | | 0 | | 1 | | | 1 | | 2 | |  |  |  |  |
| P46013 | Proliferation marker protein Ki-67 | MKI67 | 0.80 | 2 | | 358.5 | | 9.45 | | 0.13 | 1 | | 0.00 | | 0 | | 0.00 | | 0 | | 0.00 | | 0 | | 0.28 | | 2 | | 0.00 | | 0 | | 0.00 | | 0 | | 0.00 | | 0 | | 1 | | | 1 | | 2 | |  |  |  |  |
| Q5VT06 | Centrosome-associated protein 350 | CEP350 | 1.35 | 2 | | 350.7 | | 6.33 | | 0.13 | 1 | | 0.00 | | 0 | | 0.00 | | 0 | | 0.00 | | 0 | | 0.00 | | 0 | | 0.00 | | 0 | | 0.00 | | 0 | | 0.30 | | 2 | | 1 | | | 1 | | 2 | |  |  |  |  |
| Q8IV08 | 5'-3' exonuclease PLD3 | PLD3 | 10.00 | 2 | | 54.7 | | 6.47 | | 0.15 | 1 | | 0.00 | | 0 | | 0.00 | | 0 | | 0.00 | | 0 | | 0.00 | | 0 | | 0.36 | | 1 | | 0.00 | | 0 | | 0.00 | | 0 | | 1 | | | 1 | | 2 | |  |  |  |  |
| P12270 | Nucleoprotein TPR | TPR | 1.14 | 2 | | 267.1 | | 5.02 | | 0.15 | 1 | | 0.00 | | 0 | | 0.00 | | 0 | | 0.00 | | 0 | | 0.14 | | 1 | | 0.00 | | 0 | | 0.00 | | 0 | | 0.00 | | 0 | | 1 | | | 1 | | 2 | |  |  |  |  |
| Q9BZ76 | Contactin-associated protein-like 3 | CNTNAP3 | 4.66 | 1 | | 140.6 | | 7.78 | | 0.28 | 1 | | 0.00 | | 0 | | 0.00 | | 0 | | 0.00 | | 0 | | 0.14 | | 1 | | 0.00 | | 0 | | 0.00 | | 0 | | 0.00 | | 0 | | 1 | | | 1 | | 2 | |  |  |  |  |
| Q13515 | Phakinin | BFSP2 | 6.75 | 2 | | 45.9 | | 5.55 | | 0.13 | 1 | | 0.00 | | 0 | | 0.00 | | 0 | | 0.00 | | 0 | | 0.14 | | 1 | | 0.00 | | 0 | | 0.00 | | 0 | | 0.00 | | 0 | | 1 | | | 1 | | 2 | |  |  |  |  |
| P49411 | Elongation factor Tu, mitochondrial | TUFM | 9.73 | 2 | | 49.5 | | 7.61 | | 0.16 | 1 | | 0.00 | | 0 | | 0.00 | | 0 | | 0.00 | | 0 | | 0.14 | | 1 | | 0.00 | | 0 | | 0.00 | | 0 | | 0.00 | | 0 | | 1 | | | 1 | | 2 | |  |  |  |  |
| Q13492 | Phosphatidylinositol-binding clathrin assembly protein | PICALM | 6.44 | 2 | | 70.7 | | 7.90 | | 0.15 | 1 | | 0.00 | | 0 | | 0.00 | | 0 | | 0.00 | | 0 | | 0.14 | | 1 | | 0.00 | | 0 | | 0.00 | | 0 | | 0.00 | | 0 | | 1 | | | 1 | | 2 | |  |  |  |  |
| Q9NPG2 | Neuroglobin | NGB | 15.89 | 2 | | 16.9 | | 5.54 | | 0.15 | 1 | | 0.00 | | 0 | | 0.00 | | 0 | | 0.00 | | 0 | | 0.14 | | 1 | | 0.00 | | 0 | | 0.00 | | 0 | | 0.00 | | 0 | | 1 | | | 1 | | 2 | |  |  |  |  |
| Q8IZF0 | Sodium leak channel non-selective protein | NALCN | 2.07 | 2 | | 200.2 | | 8.68 | | 0.13 | 1 | | 0.00 | | 0 | | 0.00 | | 0 | | 0.00 | | 0 | | 0.14 | | 1 | | 0.00 | | 0 | | 0.00 | | 0 | | 0.00 | | 0 | | 1 | | | 1 | | 2 | |  |  |  |  |
| A0A2R8Y4T1 | Tensin-1 | TNS1 | 3.66 | 2 | | 199.4 | | 8.34 | | 0.13 | 1 | | 0.00 | | 0 | | 0.00 | | 0 | | 0.00 | | 0 | | 0.14 | | 1 | | 0.00 | | 0 | | 0.00 | | 0 | | 0.00 | | 0 | | 1 | | | 1 | | 2 | |  |  |  |  |
| P58170 | Olfactory receptor 1D5 | OR1D5 | 7.37 | 1 | | 35.4 | | 8.44 | | 0.13 | 1 | | 0.00 | | 0 | | 0.00 | | 0 | | 0.00 | | 0 | | 0.16 | | 1 | | 0.00 | | 0 | | 0.00 | | 0 | | 0.00 | | 0 | | 1 | | | 1 | | 2 | |  |  |  |  |
| O43809 | Cleavage and polyadenylation specificity factor subunit 5 | NUDT21 | 12.78 | 1 | | 26.2 | | 8.82 | | 0.15 | 1 | | 0.00 | | 0 | | 0.00 | | 0 | | 0.00 | | 0 | | 0.14 | | 1 | | 0.00 | | 0 | | 0.00 | | 0 | | 0.00 | | 0 | | 1 | | | 1 | | 2 | |  |  |  |  |
| Q8TEP8 | Centrosomal protein of 192 kDa | CEP192 | 0.91 | 2 | | 278.9 | | 5.48 | | 0.16 | 1 | | 0.00 | | 0 | | 0.00 | | 0 | | 0.00 | | 0 | | 0.14 | | 1 | | 0.00 | | 0 | | 0.00 | | 0 | | 0.00 | | 0 | | 1 | | | 1 | | 2 | |  |  |  |  |
| Q96DY2 | Dynein regulatory complex protein 10 | IQCD | 8.46 | 2 | | 52.3 | | 9.25 | | 0.28 | 1 | | 0.00 | | 0 | | 0.00 | | 0 | | 0.00 | | 0 | | 0.14 | | 1 | | 0.00 | | 0 | | 0.00 | | 0 | | 0.00 | | 0 | | 1 | | | 1 | | 2 | |  |  |  |  |
| Q14118 | Dystroglycan | DAG1 | 2.68 | 2 | | 97.4 | | 8.56 | | 0.13 | 1 | | 0.00 | | 0 | | 0.00 | | 0 | | 0.00 | | 0 | | 0.14 | | 1 | | 0.00 | | 0 | | 0.00 | | 0 | | 0.00 | | 0 | | 1 | | | 1 | | 2 | |  |  |  |  |
| Q8IW52 | SLIT and NTRK-like protein 4 | SLITRK4 | 2.15 | 1 | | 94.3 | | 7.80 | | 0.13 | 1 | | 0.00 | | 0 | | 0.00 | | 0 | | 0.00 | | 0 | | 0.14 | | 1 | | 0.00 | | 0 | | 0.00 | | 0 | | 0.00 | | 0 | | 1 | | | 1 | | 2 | |  |  |  |  |
| A0A0G2JNF4 | Killer cell immunoglobulin-like receptor, two Ig domains pseudogene 1 | KIR2DP1 | 17.48 | 2 | | 38.6 | | 7.08 | | 0.13 | 1 | | 0.00 | | 0 | | 0.00 | | 0 | | 0.00 | | 0 | | 0.14 | | 1 | | 0.00 | | 0 | | 0.00 | | 0 | | 0.00 | | 0 | | 1 | | | 1 | | 2 | |  |  |  |  |
| Q12766 | HMG domain-containing protein 3 | HMGXB3 | 4.42 | 2 | | 168.2 | | 8.05 | | 0.13 | 1 | | 0.00 | | 0 | | 0.00 | | 0 | | 0.00 | | 0 | | 0.14 | | 1 | | 0.00 | | 0 | | 0.00 | | 0 | | 0.00 | | 0 | | 1 | | | 1 | | 2 | |  |  |  |  |
| B2RXH4 | BTB/POZ domain-containing protein 18 | BTBD18 | 7.44 | 2 | | 77.9 | | 5.00 | | 0.13 | 1 | | 0.00 | | 0 | | 0.00 | | 0 | | 0.00 | | 0 | | 0.00 | | 0 | | 0.18 | | 1 | | 0.00 | | 0 | | 0.00 | | 0 | | 1 | | | 1 | | 2 | |  |  |  |  |
| S4R3U7 | Zinc finger SWIM domain-containing protein 8 (Fragment) | ZSWIM8 | 3.00 | 2 | | 129.5 | | 6.44 | | 0.16 | 1 | | 0.00 | | 0 | | 0.00 | | 0 | | 0.00 | | 0 | | 0.00 | | 0 | | 0.18 | | 1 | | 0.00 | | 0 | | 0.00 | | 0 | | 1 | | | 1 | | 2 | |  |  |  |  |
| A0A3F2YNY5 | Small conductance calcium-activated potassium channel protein 2 | KCNN2 | 2.10 | 1 | | 93.6 | | 9.28 | | 0.13 | 1 | | 0.00 | | 0 | | 0.00 | | 0 | | 0.00 | | 0 | | 0.00 | | 0 | | 0.18 | | 1 | | 0.00 | | 0 | | 0.00 | | 0 | | 1 | | | 1 | | 2 | |  |  |  |  |
| Q8WZA1 | Protein O-linked-mannose beta-1,2-N-acetylglucosaminyltransferase 1 | POMGNT1 | 11.52 | 2 | | 75.2 | | 6.83 | | 0.13 | 1 | | 0.00 | | 0 | | 0.00 | | 0 | | 0.00 | | 0 | | 0.00 | | 0 | | 0.18 | | 1 | | 0.00 | | 0 | | 0.00 | | 0 | | 1 | | | 1 | | 2 | |  |  |  |  |
| Q6UWX4 | HHIP-like protein 2 | HHIPL2 | 4.83 | 1 | | 80.7 | | 9.01 | | 0.16 | 1 | | 0.00 | | 0 | | 0.00 | | 0 | | 0.00 | | 0 | | 0.00 | | 0 | | 0.23 | | 1 | | 0.00 | | 0 | | 0.00 | | 0 | | 1 | | | 1 | | 2 | |  |  |  |  |
| E7ETV6 | SRSF protein kinase 3 | SRPK3 | 3.47 | 2 | | 69.2 | | 7.23 | | 0.15 | 1 | | 0.00 | | 0 | | 0.00 | | 0 | | 0.00 | | 0 | | 0.00 | | 0 | | 0.24 | | 1 | | 0.00 | | 0 | | 0.00 | | 0 | | 1 | | | 1 | | 2 | |  |  |  |  |
| O15021 | Microtubule-associated serine/threonine-protein kinase 4 | MAST4 | 1.45 | 2 | | 283.9 | | 8.62 | | 0.13 | 1 | | 0.00 | | 0 | | 0.00 | | 0 | | 0.00 | | 0 | | 0.00 | | 0 | | 0.24 | | 1 | | 0.00 | | 0 | | 0.00 | | 0 | | 1 | | | 1 | | 2 | |  |  |  |  |
| Q9H7E2 | Tudor domain-containing protein 3 | TDRD3 | 6.14 | 2 | | 73.1 | | 9.23 | | 0.13 | 1 | | 0.00 | | 0 | | 0.00 | | 0 | | 0.00 | | 0 | | 0.00 | | 0 | | 0.00 | | 0 | | 0.17 | | 1 | | 0.00 | | 0 | | 1 | | | 1 | | 2 | |  |  |  |  |
| Q96PN6 | Adenylate cyclase type 10 | ADCY10 | 0.99 | 2 | | 187.0 | | 7.31 | | 0.28 | 1 | | 0.00 | | 0 | | 0.00 | | 0 | | 0.00 | | 0 | | 0.00 | | 0 | | 0.00 | | 0 | | 0.15 | | 1 | | 0.00 | | 0 | | 1 | | | 1 | | 2 | |  |  |  |  |
| Q86W28 | NACHT, LRR and PYD domains-containing protein 8 | NLRP8 | 1.15 | 1 | | 119.4 | | 7.96 | | 0.15 | 1 | | 0.00 | | 0 | | 0.00 | | 0 | | 0.00 | | 0 | | 0.00 | | 0 | | 0.00 | | 0 | | 0.15 | | 1 | | 0.00 | | 0 | | 1 | | | 1 | | 2 | |  |  |  |  |
| Q03164 | Histone-lysine N-methyltransferase 2A | KMT2A | 0.76 | 2 | | 431.5 | | 9.09 | | 0.13 | 1 | | 0.00 | | 0 | | 0.00 | | 0 | | 0.00 | | 0 | | 0.00 | | 0 | | 0.00 | | 0 | | 0.15 | | 1 | | 0.00 | | 0 | | 1 | | | 1 | | 2 | |  |  |  |  |
| Q06265 | Exosome complex component RRP45 | EXOSC9 | 10.25 | 2 | | 48.9 | | 5.29 | | 0.16 | 1 | | 0.00 | | 0 | | 0.00 | | 0 | | 0.00 | | 0 | | 0.00 | | 0 | | 0.00 | | 0 | | 0.15 | | 1 | | 0.00 | | 0 | | 1 | | | 1 | | 2 | |  |  |  |  |
| Q5THJ4 | Vacuolar protein sorting-associated protein 13D | VPS13D | 0.75 | 2 | | 491.6 | | 6.58 | | 0.28 | 1 | | 0.00 | | 0 | | 0.00 | | 0 | | 0.00 | | 0 | | 0.00 | | 0 | | 0.00 | | 0 | | 0.15 | | 1 | | 0.00 | | 0 | | 1 | | | 1 | | 2 | |  |  |  |  |
| P15822 | Zinc finger protein 40 | HIVEP1 | 1.51 | 2 | | 296.7 | | 7.84 | | 0.16 | 1 | | 0.00 | | 0 | | 0.00 | | 0 | | 0.00 | | 0 | | 0.00 | | 0 | | 0.00 | | 0 | | 0.14 | | 1 | | 0.00 | | 0 | | 1 | | | 1 | | 2 | |  |  |  |  |
| Q86U70 | LIM domain-binding protein 1 | LDB1 | 14.60 | 2 | | 46.5 | | 6.96 | | 0.13 | 1 | | 0.00 | | 0 | | 0.00 | | 0 | | 0.00 | | 0 | | 0.00 | | 0 | | 0.00 | | 0 | | 0.14 | | 1 | | 0.00 | | 0 | | 1 | | | 1 | | 2 | |  |  |  |  |
| Q8N1W2 | Zinc finger protein 710 | ZNF710 | 5.57 | 2 | | 74.4 | | 6.80 | | 0.13 | 1 | | 0.00 | | 0 | | 0.00 | | 0 | | 0.00 | | 0 | | 0.00 | | 0 | | 0.00 | | 0 | | 0.14 | | 1 | | 0.00 | | 0 | | 1 | | | 1 | | 2 | |  |  |  |  |
| Q9Y6L7 | Tolloid-like protein 2 | TLL2 | 3.55 | 2 | | 113.5 | | 5.94 | | 0.13 | 1 | | 0.00 | | 0 | | 0.00 | | 0 | | 0.00 | | 0 | | 0.00 | | 0 | | 0.00 | | 0 | | 0.14 | | 1 | | 0.00 | | 0 | | 1 | | | 1 | | 2 | |  |  |  |  |
| Q6PD74 | Alpha- and gamma-adaptin-binding protein p34 | AAGAB | 15.87 | 1 | | 34.6 | | 4.64 | | 0.13 | 1 | | 0.00 | | 0 | | 0.00 | | 0 | | 0.00 | | 0 | | 0.00 | | 0 | | 0.00 | | 0 | | 0.14 | | 1 | | 0.00 | | 0 | | 1 | | | 1 | | 2 | |  |  |  |  |
| Q6ZPD9 | Probable C-mannosyltransferase DPY19L3 | DPY19L3 | 4.19 | 1 | | 83.1 | | 8.72 | | 0.13 | 1 | | 0.00 | | 0 | | 0.00 | | 0 | | 0.00 | | 0 | | 0.00 | | 0 | | 0.00 | | 0 | | 0.14 | | 1 | | 0.00 | | 0 | | 1 | | | 1 | | 2 | |  |  |  |  |
| O75426 | F-box only protein 24 | FBXO24 | 8.79 | 2 | | 64.8 | | 8.69 | | 0.13 | 1 | | 0.00 | | 0 | | 0.00 | | 0 | | 0.00 | | 0 | | 0.00 | | 0 | | 0.00 | | 0 | | 0.00 | | 0 | | 0.18 | | 1 | | 1 | | | 1 | | 2 | |  |  |  |  |
| H7BXG6 | Zinc finger protein 541 (Fragment) | ZNF541 | 2.95 | 2 | | 73.4 | | 9.39 | | 0.16 | 1 | | 0.00 | | 0 | | 0.00 | | 0 | | 0.00 | | 0 | | 0.00 | | 0 | | 0.00 | | 0 | | 0.00 | | 0 | | 0.15 | | 1 | | 1 | | | 1 | | 2 | |  |  |  |  |
| Q13616 | Cullin-1 | CUL1 | 4.12 | 2 | | 89.6 | | 8.00 | | 0.15 | 1 | | 0.00 | | 0 | | 0.00 | | 0 | | 0.00 | | 0 | | 0.00 | | 0 | | 0.00 | | 0 | | 0.00 | | 0 | | 0.15 | | 1 | | 1 | | | 1 | | 2 | |  |  |  |  |
| Q8N0X7 | Spartin | SPART | 9.16 | 2 | | 72.8 | | 5.91 | | 0.28 | 1 | | 0.00 | | 0 | | 0.00 | | 0 | | 0.00 | | 0 | | 0.00 | | 0 | | 0.00 | | 0 | | 0.00 | | 0 | | 0.15 | | 1 | | 1 | | | 1 | | 2 | |  |  |  |  |
| H0Y9C6 | Acyl-CoA dehydrogenase family member 11 (Fragment) | ACAD11 | 10.97 | 1 | | 17.2 | | 6.40 | | 0.13 | 1 | | 0.00 | | 0 | | 0.00 | | 0 | | 0.00 | | 0 | | 0.00 | | 0 | | 0.00 | | 0 | | 0.00 | | 0 | | 0.15 | | 1 | | 1 | | | 1 | | 2 | |  |  |  |  |
| J3QS41 | Probable helicase with zinc finger domain | HELZ | 2.26 | 2 | | 218.9 | | 7.42 | | 0.15 | 1 | | 0.00 | | 0 | | 0.00 | | 0 | | 0.00 | | 0 | | 0.00 | | 0 | | 0.00 | | 0 | | 0.00 | | 0 | | 0.15 | | 1 | | 1 | | | 1 | | 2 | |  |  |  |  |
| A0A0C4DGV4 | Hepatitis B virus x interacting protein | LAMTOR5 | 39.31 | 2 | | 18.1 | | 5.50 | | 0.13 | 1 | | 0.00 | | 0 | | 0.00 | | 0 | | 0.00 | | 0 | | 0.00 | | 0 | | 0.00 | | 0 | | 0.00 | | 0 | | 0.15 | | 1 | | 1 | | | 1 | | 2 | |  |  |  |  |
| Q6PL18 | ATPase family AAA domain-containing protein 2 | ATAD2 | 3.45 | 2 | | 158.5 | | 6.32 | | 0.16 | 1 | | 0.00 | | 0 | | 0.00 | | 0 | | 0.00 | | 0 | | 0.00 | | 0 | | 0.00 | | 0 | | 0.00 | | 0 | | 0.15 | | 1 | | 1 | | | 1 | | 2 | |  |  |  |  |
| R4GMQ1 | Lysine-specific histone demethylase | KDM1A | 5.59 | 2 | | 93.5 | | 6.51 | | 0.16 | 1 | | 0.00 | | 0 | | 0.00 | | 0 | | 0.00 | | 0 | | 0.00 | | 0 | | 0.00 | | 0 | | 0.00 | | 0 | | 0.15 | | 1 | | 1 | | | 1 | | 2 | |  |  |  |  |
| Q96NU0 | Contactin-associated protein-like 3B | CNTNAP3B | 2.87 | 1 | | 140.3 | | 7.40 | | 0.28 | 1 | | 0.00 | | 0 | | 0.00 | | 0 | | 0.00 | | 0 | | 0.00 | | 0 | | 0.00 | | 0 | | 0.00 | | 0 | | 0.15 | | 1 | | 1 | | | 1 | | 2 | |  |  |  |  |
| A0A494C120 | Zinc finger matrin-type protein 3 | ZMAT3 | 14.88 | 2 | | 42.7 | | 9.17 | | 0.13 | 1 | | 0.00 | | 0 | | 0.00 | | 0 | | 0.00 | | 0 | | 0.00 | | 0 | | 0.00 | | 0 | | 0.00 | | 0 | | 0.15 | | 1 | | 1 | | | 1 | | 2 | |  |  |  |  |
| P08575 | Receptor-type tyrosine-protein phosphatase C | PTPRC | 1.53 | 2 | | 147.4 | | 6.15 | | 0.00 | 0 | | 0.00 | | 0 | | 0.38 | | 2 | | 0.00 | | 0 | | 0.16 | | 1 | | 0.00 | | 0 | | 0.00 | | 0 | | 0.00 | | 0 | | 1 | | | 1 | | 2 | |  |  |  |  |
| Q8NEK5 | Zinc finger protein 548 | ZNF548 | 5.82 | 2 | | 62.7 | | 8.03 | | 0.00 | 0 | | 0.00 | | 0 | | 0.34 | | 2 | | 0.00 | | 0 | | 0.16 | | 1 | | 0.00 | | 0 | | 0.00 | | 0 | | 0.00 | | 0 | | 1 | | | 1 | | 2 | |  |  |  |  |
| P31327 | Carbamoyl-phosphate synthase [ammonia], mitochondrial | CPS1 | 2.60 | 2 | | 164.8 | | 6.74 | | 0.00 | 0 | | 0.00 | | 0 | | 0.32 | | 1 | | 0.00 | | 0 | | 0.00 | | 0 | | 0.18 | | 1 | | 0.00 | | 0 | | 0.00 | | 0 | | 1 | | | 1 | | 2 | |  |  |  |  |
| P51589 | Cytochrome P450 2J2 | CYP2J2 | 9.76 | 2 | | 57.6 | | 8.60 | | 0.00 | 0 | | 0.00 | | 0 | | 0.22 | | 1 | | 0.00 | | 0 | | 0.00 | | 0 | | 0.00 | | 0 | | 0.00 | | 0 | | 0.33 | | 2 | | 1 | | | 1 | | 2 | |  |  |  |  |
| O43747 | AP-1 complex subunit gamma-1 | AP1G1 | 2.43 | 2 | | 91.3 | | 6.80 | | 0.00 | 0 | | 0.00 | | 0 | | 0.22 | | 1 | | 0.00 | | 0 | | 0.14 | | 1 | | 0.00 | | 0 | | 0.00 | | 0 | | 0.00 | | 0 | | 1 | | | 1 | | 2 | |  |  |  |  |
| P00540 | Proto-oncogene serine/threonine-protein kinase mos | MOS | 2.60 | 1 | | 37.8 | | 8.91 | | 0.00 | 0 | | 0.00 | | 0 | | 0.22 | | 1 | | 0.00 | | 0 | | 0.14 | | 1 | | 0.00 | | 0 | | 0.00 | | 0 | | 0.00 | | 0 | | 1 | | | 1 | | 2 | |  |  |  |  |
| Q9HAU0 | Pleckstrin homology domain-containing family A member 5 | PLEKHA5 | 3.58 | 1 | | 127.4 | | 7.53 | | 0.00 | 0 | | 0.00 | | 0 | | 0.22 | | 1 | | 0.00 | | 0 | | 0.00 | | 0 | | 0.00 | | 0 | | 0.17 | | 1 | | 0.00 | | 0 | | 1 | | | 1 | | 2 | |  |  |  |  |
| A0A590UJA9 | Transmembrane and ubiquitin-like domain-containing protein 1 | TMUB1 | 4.09 | 1 | | 37.3 | | 9.54 | | 0.00 | 0 | | 0.00 | | 0 | | 0.22 | | 1 | | 0.00 | | 0 | | 0.14 | | 1 | | 0.00 | | 0 | | 0.00 | | 0 | | 0.00 | | 0 | | 1 | | | 1 | | 2 | |  |  |  |  |
| A0A087X009 | Histo-blood group ABO system transferase | ABO | 4.56 | 2 | | 42.9 | | 9.20 | | 0.00 | 0 | | 0.00 | | 0 | | 0.22 | | 1 | | 0.00 | | 0 | | 0.00 | | 0 | | 0.16 | | 1 | | 0.00 | | 0 | | 0.00 | | 0 | | 1 | | | 1 | | 2 | |  |  |  |  |
| Q9P2E2 | Kinesin-like protein KIF17 | KIF17 | 5.83 | 2 | | 115.0 | | 5.50 | | 0.00 | 0 | | 0.00 | | 0 | | 0.22 | | 1 | | 0.00 | | 0 | | 0.00 | | 0 | | 0.00 | | 0 | | 0.14 | | 1 | | 0.00 | | 0 | | 1 | | | 1 | | 2 | |  |  |  |  |
| Q5VT25 | Serine/threonine-protein kinase MRCK alpha | CDC42BPA | 1.27 | 2 | | 197.2 | | 6.58 | | 0.00 | 0 | | 0.00 | | 0 | | 0.19 | | 1 | | 0.00 | | 0 | | 0.00 | | 0 | | 0.00 | | 0 | | 0.29 | | 2 | | 0.00 | | 0 | | 1 | | | 1 | | 2 | |  |  |  |  |
| O95996 | Adenomatous polyposis coli protein 2 | APC2 | 0.39 | 1 | | 243.8 | | 8.82 | | 0.00 | 0 | | 0.00 | | 0 | | 0.19 | | 1 | | 0.00 | | 0 | | 0.00 | | 0 | | 0.47 | | 1 | | 0.00 | | 0 | | 0.00 | | 0 | | 1 | | | 1 | | 2 | |  |  |  |  |
| Q7Z745 | Maestro heat-like repeat-containing protein family member 2B | MROH2B | 4.16 | 2 | | 180.7 | | 6.28 | | 0.00 | 0 | | 0.00 | | 0 | | 0.19 | | 1 | | 0.00 | | 0 | | 0.00 | | 0 | | 0.16 | | 1 | | 0.00 | | 0 | | 0.00 | | 0 | | 1 | | | 1 | | 2 | |  |  |  |  |
| Q9NR97 | Toll-like receptor 8 | TLR8 | 3.65 | 2 | | 119.8 | | 6.65 | | 0.00 | 0 | | 0.00 | | 0 | | 0.19 | | 1 | | 0.00 | | 0 | | 0.00 | | 0 | | 0.00 | | 0 | | 0.15 | | 1 | | 0.00 | | 0 | | 1 | | | 1 | | 2 | |  |  |  |  |
| Q8IWR0 | Zinc finger CCCH domain-containing protein 7A | ZC3H7A | 4.33 | 2 | | 110.5 | | 7.30 | | 0.00 | 0 | | 0.00 | | 0 | | 0.19 | | 1 | | 0.00 | | 0 | | 0.00 | | 0 | | 0.00 | | 0 | | 0.00 | | 0 | | 0.15 | | 1 | | 1 | | | 1 | | 2 | |  |  |  |  |
| Q9BYX4 | Interferon-induced helicase C domain-containing protein 1 | IFIH1 | 4.68 | 2 | | 116.6 | | 5.52 | | 0.00 | 0 | | 0.00 | | 0 | | 0.19 | | 1 | | 0.00 | | 0 | | 0.00 | | 0 | | 0.00 | | 0 | | 0.00 | | 0 | | 0.15 | | 1 | | 1 | | | 1 | | 2 | |  |  |  |  |
| Q6I9Y2 | THO complex subunit 7 homolog | THOC7 | 11.76 | 2 | | 23.7 | | 5.67 | | 0.00 | 0 | | 0.00 | | 0 | | 0.19 | | 1 | | 0.00 | | 0 | | 0.14 | | 1 | | 0.00 | | 0 | | 0.00 | | 0 | | 0.00 | | 0 | | 1 | | | 1 | | 2 | |  |  |  |  |
| Q9NZJ4 | Sacsin | SACS | 0.52 | 2 | | 520.8 | | 7.05 | | 0.00 | 0 | | 0.00 | | 0 | | 0.19 | | 1 | | 0.00 | | 0 | | 0.00 | | 0 | | 0.16 | | 1 | | 0.00 | | 0 | | 0.00 | | 0 | | 1 | | | 1 | | 2 | |  |  |  |  |
| B5MDU6 | Lipid droplet-associated hydrolase | LDAH | 13.78 | 2 | | 42.6 | | 6.93 | | 0.00 | 0 | | 0.00 | | 0 | | 0.19 | | 1 | | 0.00 | | 0 | | 0.00 | | 0 | | 0.00 | | 0 | | 0.00 | | 0 | | 0.18 | | 1 | | 1 | | | 1 | | 2 | |  |  |  |  |
| O75578 | Integrin alpha-10 | ITGA10 | 2.49 | 2 | | 127.5 | | 6.68 | | 0.00 | 0 | | 0.00 | | 0 | | 0.19 | | 1 | | 0.00 | | 0 | | 0.00 | | 0 | | 0.00 | | 0 | | 0.00 | | 0 | | 0.18 | | 1 | | 1 | | | 1 | | 2 | |  |  |  |  |
| G5E9Q7 | Isocitrate dehydrogenase [NAD] subunit, mitochondrial | IDH3G | 15.22 | 1 | | 35.4 | | 8.51 | | 0.00 | 0 | | 0.00 | | 0 | | 0.19 | | 1 | | 0.00 | | 0 | | 0.00 | | 0 | | 0.00 | | 0 | | 0.00 | | 0 | | 0.15 | | 1 | | 1 | | | 1 | | 2 | |  |  |  |  |
| P51553 | Isocitrate dehydrogenase [NAD] subunit gamma, mitochondrial | IDH3G | 12.47 | 1 | | 42.8 | | 8.50 | | 0.00 | 0 | | 0.00 | | 0 | | 0.19 | | 1 | | 0.00 | | 0 | | 0.00 | | 0 | | 0.00 | | 0 | | 0.00 | | 0 | | 0.15 | | 1 | | 1 | | | 1 | | 2 | |  |  |  |  |
| Q8NFQ8 | Torsin-1A-interacting protein 2 | TOR1AIP2 | 8.94 | 2 | | 51.2 | | 4.96 | | 0.00 | 0 | | 0.00 | | 0 | | 0.19 | | 1 | | 0.00 | | 0 | | 0.00 | | 0 | | 0.00 | | 0 | | 0.00 | | 0 | | 0.15 | | 1 | | 1 | | | 1 | | 2 | |  |  |  |  |
| H0YBK2 | Protocadherin Fat 2 (Fragment) | FAT2 | 3.92 | 1 | | 122.3 | | 5.82 | | 0.00 | 0 | | 0.00 | | 0 | | 0.16 | | 1 | | 0.00 | | 0 | | 0.14 | | 1 | | 0.00 | | 0 | | 0.00 | | 0 | | 0.00 | | 0 | | 1 | | | 1 | | 2 | |  |  |  |  |
| Q9UL16 | Cilia- and flagella-associated protein 45 | CFAP45 | 1.45 | 1 | | 65.7 | | 8.90 | | 0.00 | 0 | | 0.00 | | 0 | | 0.16 | | 1 | | 0.00 | | 0 | | 0.16 | | 1 | | 0.00 | | 0 | | 0.00 | | 0 | | 0.00 | | 0 | | 1 | | | 1 | | 2 | |  |  |  |  |
| Q9HBH9 | MAP kinase-interacting serine/threonine-protein kinase 2 | MKNK2 | 12.69 | 2 | | 51.8 | | 6.19 | | 0.00 | 0 | | 0.00 | | 0 | | 0.16 | | 1 | | 0.00 | | 0 | | 0.00 | | 0 | | 0.18 | | 1 | | 0.00 | | 0 | | 0.00 | | 0 | | 1 | | | 1 | | 2 | |  |  |  |  |
| P42704 | Leucine-rich PPR motif-containing protein, mitochondrial | LRPPRC | 2.94 | 2 | | 157.8 | | 6.13 | | 0.00 | 0 | | 0.00 | | 0 | | 0.16 | | 1 | | 0.00 | | 0 | | 0.00 | | 0 | | 0.00 | | 0 | | 0.15 | | 1 | | 0.00 | | 0 | | 1 | | | 1 | | 2 | |  |  |  |  |
| P00439 | Phenylalanine-4-hydroxylase | PAH | 2.21 | 1 | | 51.8 | | 6.60 | | 0.00 | 0 | | 0.00 | | 0 | | 0.16 | | 1 | | 0.00 | | 0 | | 0.00 | | 0 | | 0.00 | | 0 | | 0.00 | | 0 | | 0.18 | | 1 | | 1 | | | 1 | | 2 | |  |  |  |  |
| Q641Q2 | WASH complex subunit 2A | WASHC2A | 3.13 | 2 | | 147.1 | | 4.81 | | 0.00 | 0 | | 0.00 | | 0 | | 0.16 | | 1 | | 0.00 | | 0 | | 0.00 | | 0 | | 0.00 | | 0 | | 0.00 | | 0 | | 0.18 | | 1 | | 1 | | | 1 | | 2 | |  |  |  |  |
| Q9Y574 | Ankyrin repeat and SOCS box protein 4 | ASB4 | 2.58 | 2 | | 48.2 | | 7.64 | | 0.00 | 0 | | 0.00 | | 0 | | 0.16 | | 1 | | 0.00 | | 0 | | 0.00 | | 0 | | 0.00 | | 0 | | 0.00 | | 0 | | 0.15 | | 1 | | 1 | | | 1 | | 2 | |  |  |  |  |
| H7BZC5 | Exportin-1 (Fragment) | XPO1 | 29.17 | 1 | | 8.7 | | 7.34 | | 0.00 | 0 | | 0.00 | | 0 | | 0.16 | | 1 | | 0.00 | | 0 | | 0.42 | | 2 | | 0.00 | | 0 | | 0.00 | | 0 | | 0.00 | | 0 | | 1 | | | 1 | | 2 | |  |  |  |  |
| Q8NDH2 | Coiled-coil domain-containing protein 168 | CCDC168 | 1.55 | 2 | | 277.8 | | 9.31 | | 0.00 | 0 | | 0.00 | | 0 | | 0.16 | | 1 | | 0.00 | | 0 | | 0.14 | | 1 | | 0.00 | | 0 | | 0.00 | | 0 | | 0.00 | | 0 | | 1 | | | 1 | | 2 | |  |  |  |  |
| Q96G01 | Protein bicaudal D homolog 1 | BICD1 | 2.87 | 1 | | 110.7 | | 5.81 | | 0.00 | 0 | | 0.00 | | 0 | | 0.16 | | 1 | | 0.00 | | 0 | | 0.16 | | 1 | | 0.00 | | 0 | | 0.00 | | 0 | | 0.00 | | 0 | | 1 | | | 1 | | 2 | |  |  |  |  |
| A6NC86 | phospholipase A2 inhibitor and Ly6/PLAUR domain-containing protein | PINLYP | 28.43 | 1 | | 21.9 | | 7.99 | | 0.00 | 0 | | 0.00 | | 0 | | 0.16 | | 1 | | 0.00 | | 0 | | 0.16 | | 1 | | 0.00 | | 0 | | 0.00 | | 0 | | 0.00 | | 0 | | 1 | | | 1 | | 2 | |  |  |  |  |
| A6NGQ3 | Obscurin | OBSCN | 0.71 | 2 | | 972.4 | | 5.99 | | 0.00 | 0 | | 0.00 | | 0 | | 0.16 | | 1 | | 0.00 | | 0 | | 0.14 | | 1 | | 0.00 | | 0 | | 0.00 | | 0 | | 0.00 | | 0 | | 1 | | | 1 | | 2 | |  |  |  |  |
| I3L184 | Vacuolar protein sorting-associated protein 53 homolog (Fragment) | VPS53 | 6.75 | 1 | | 27.1 | | 8.82 | | 0.00 | 0 | | 0.00 | | 0 | | 0.16 | | 1 | | 0.00 | | 0 | | 0.00 | | 0 | | 0.00 | | 0 | | 0.15 | | 1 | | 0.00 | | 0 | | 1 | | | 1 | | 2 | |  |  |  |  |
| Q9NR99 | Matrix-remodeling-associated protein 5 | MXRA5 | 1.17 | 2 | | 312.0 | | 8.32 | | 0.00 | 0 | | 0.00 | | 0 | | 0.16 | | 1 | | 0.00 | | 0 | | 0.00 | | 0 | | 0.00 | | 0 | | 0.00 | | 0 | | 0.15 | | 1 | | 1 | | | 1 | | 2 | |  |  |  |  |
| Q7Z5K2 | Wings apart-like protein homolog | WAPL | 5.13 | 2 | | 132.9 | | 5.44 | | 0.00 | 0 | | 0.00 | | 0 | | 0.16 | | 1 | | 0.00 | | 0 | | 0.00 | | 0 | | 0.00 | | 0 | | 0.00 | | 0 | | 0.15 | | 1 | | 1 | | | 1 | | 2 | |  |  |  |  |
| A0A087X1N7 | Nebulin | NEB | 0.67 | 3 | | 990.2 | | 9.01 | | 0.00 | 0 | | 0.00 | | 0 | | 0.00 | | 0 | | 0.37 | | 2 | | 0.00 | | 0 | | 0.00 | | 0 | | 0.31 | | 2 | | 0.00 | | 0 | | 1 | | | 1 | | 2 | |  |  |  |  |
| O95757 | Heat shock 70 kDa protein 4L | HSPA4L | 8.82 | 3 | | 94.5 | | 5.88 | | 0.00 | 0 | | 0.00 | | 0 | | 0.00 | | 0 | | 0.32 | | 2 | | 0.00 | | 0 | | 0.24 | | 1 | | 0.00 | | 0 | | 0.00 | | 0 | | 1 | | | 1 | | 2 | |  |  |  |  |
| Q6NSI4 | RPA-related protein RADX | RADX | 1.99 | 1 | | 97.5 | | 8.41 | | 0.00 | 0 | | 0.00 | | 0 | | 0.00 | | 0 | | 0.32 | | 2 | | 0.00 | | 0 | | 0.00 | | 0 | | 0.15 | | 1 | | 0.00 | | 0 | | 1 | | | 1 | | 2 | |  |  |  |  |
| Q9BRK5 | 45 kDa calcium-binding protein | SDF4 | 7.73 | 1 | | 41.8 | | 4.86 | | 0.00 | 0 | | 0.00 | | 0 | | 0.00 | | 0 | | 0.31 | | 1 | | 0.28 | | 2 | | 0.00 | | 0 | | 0.00 | | 0 | | 0.00 | | 0 | | 1 | | | 1 | | 2 | |  |  |  |  |
| Q8WWZ4 | ATP-binding cassette sub-family A member 10 | ABCA10 | 2.40 | 2 | | 175.7 | | 6.65 | | 0.00 | 0 | | 0.00 | | 0 | | 0.00 | | 0 | | 0.31 | | 1 | | 0.16 | | 1 | | 0.00 | | 0 | | 0.00 | | 0 | | 0.00 | | 0 | | 1 | | | 1 | | 2 | |  |  |  |  |
| O95714 | E3 ubiquitin-protein ligase HERC2 | HERC2 | 0.97 | 2 | | 526.9 | | 6.28 | | 0.00 | 0 | | 0.00 | | 0 | | 0.00 | | 0 | | 0.31 | | 2 | | 0.14 | | 1 | | 0.00 | | 0 | | 0.00 | | 0 | | 0.00 | | 0 | | 1 | | | 1 | | 2 | |  |  |  |  |
| Q9HC16 | DNA dC->dU-editing enzyme APOBEC-3G | APOBEC3G | 8.33 | 2 | | 46.4 | | 8.00 | | 0.00 | 0 | | 0.00 | | 0 | | 0.00 | | 0 | | 0.31 | | 2 | | 0.00 | | 0 | | 0.00 | | 0 | | 0.17 | | 1 | | 0.00 | | 0 | | 1 | | | 1 | | 2 | |  |  |  |  |
| Q7Z3T1 | Olfactory receptor 2W3 | OR2W3 | 14.01 | 2 | | 34.8 | | 8.60 | | 0.00 | 0 | | 0.00 | | 0 | | 0.00 | | 0 | | 0.21 | | 1 | | 0.14 | | 1 | | 0.00 | | 0 | | 0.00 | | 0 | | 0.00 | | 0 | | 1 | | | 1 | | 2 | |  |  |  |  |
| Q99973 | Telomerase protein component 1 | TEP1 | 1.83 | 2 | | 290.3 | | 7.97 | | 0.00 | 0 | | 0.00 | | 0 | | 0.00 | | 0 | | 0.21 | | 1 | | 0.14 | | 1 | | 0.00 | | 0 | | 0.00 | | 0 | | 0.00 | | 0 | | 1 | | | 1 | | 2 | |  |  |  |  |
| Q6MZM0 | Ferroxidase HEPHL1 | HEPHL1 | 4.83 | 2 | | 131.5 | | 6.74 | | 0.00 | 0 | | 0.00 | | 0 | | 0.00 | | 0 | | 0.21 | | 1 | | 0.00 | | 0 | | 0.16 | | 1 | | 0.00 | | 0 | | 0.00 | | 0 | | 1 | | | 1 | | 2 | |  |  |  |  |
| Q9Y3P9 | Rab GTPase-activating protein 1 | RABGAP1 | 2.99 | 2 | | 121.7 | | 5.25 | | 0.00 | 0 | | 0.00 | | 0 | | 0.00 | | 0 | | 0.21 | | 1 | | 0.00 | | 0 | | 0.00 | | 0 | | 0.00 | | 0 | | 0.15 | | 1 | | 1 | | | 1 | | 2 | |  |  |  |  |
| A0A087WW40 | Endophilin-B1 | SH3GLB1 | 6.60 | 2 | | 44.2 | | 6.44 | | 0.00 | 0 | | 0.00 | | 0 | | 0.00 | | 0 | | 0.21 | | 1 | | 0.00 | | 0 | | 0.00 | | 0 | | 0.00 | | 0 | | 0.15 | | 1 | | 1 | | | 1 | | 2 | |  |  |  |  |
| P54277 | PMS1 protein homolog 1 | PMS1 | 7.19 | 2 | | 105.8 | | 6.67 | | 0.00 | 0 | | 0.00 | | 0 | | 0.00 | | 0 | | 0.21 | | 1 | | 0.00 | | 0 | | 0.00 | | 0 | | 0.00 | | 0 | | 0.15 | | 1 | | 1 | | | 1 | | 2 | |  |  |  |  |
| E5RG17 | Putative deoxyribonuclease TATDN1 (Fragment) | TATDN1 | 11.80 | 1 | | 36.4 | | 7.65 | | 0.00 | 0 | | 0.00 | | 0 | | 0.00 | | 0 | | 0.21 | | 1 | | 0.00 | | 0 | | 0.00 | | 0 | | 0.00 | | 0 | | 0.15 | | 1 | | 1 | | | 1 | | 2 | |  |  |  |  |
| Q6JEL2 | Kelch-like protein 10 | KLHL10 | 6.74 | 2 | | 68.9 | | 5.68 | | 0.00 | 0 | | 0.00 | | 0 | | 0.00 | | 0 | | 0.21 | | 1 | | 0.00 | | 0 | | 0.00 | | 0 | | 0.00 | | 0 | | 0.15 | | 1 | | 1 | | | 1 | | 2 | |  |  |  |  |
| A8K8P3 | Protein SFI1 homolog | SFI1 | 2.33 | 2 | | 147.6 | | 10.81 | | 0.00 | 0 | | 0.00 | | 0 | | 0.00 | | 0 | | 0.21 | | 1 | | 0.00 | | 0 | | 0.00 | | 0 | | 0.34 | | 1 | | 0.00 | | 0 | | 1 | | | 1 | | 2 | |  |  |  |  |
| Q9NZV7 | Zinc finger imprinted 2 | ZIM2 | 5.88 | 2 | | 61.1 | | 6.77 | | 0.00 | 0 | | 0.00 | | 0 | | 0.00 | | 0 | | 0.21 | | 1 | | 0.00 | | 0 | | 0.24 | | 1 | | 0.00 | | 0 | | 0.00 | | 0 | | 1 | | | 1 | | 2 | |  |  |  |  |
| Q96MT7 | Cilia- and flagella-associated protein 44 | CFAP44 | 1.19 | 2 | | 213.7 | | 5.39 | | 0.00 | 0 | | 0.00 | | 0 | | 0.00 | | 0 | | 0.21 | | 1 | | 0.00 | | 0 | | 0.00 | | 0 | | 0.17 | | 1 | | 0.00 | | 0 | | 1 | | | 1 | | 2 | |  |  |  |  |
| E9PGK7 | Transient receptor potential cation channel subfamily M member 2 | TRPM2 | 2.90 | 2 | | 176.4 | | 7.74 | | 0.00 | 0 | | 0.00 | | 0 | | 0.00 | | 0 | | 0.17 | | 1 | | 0.28 | | 2 | | 0.00 | | 0 | | 0.00 | | 0 | | 0.00 | | 0 | | 1 | | | 1 | | 2 | |  |  |  |  |
| Q02224 | Centromere-associated protein E | CENPE | 0.85 | 2 | | 316.2 | | 5.64 | | 0.00 | 0 | | 0.00 | | 0 | | 0.00 | | 0 | | 0.17 | | 1 | | 0.14 | | 1 | | 0.00 | | 0 | | 0.00 | | 0 | | 0.00 | | 0 | | 1 | | | 1 | | 2 | |  |  |  |  |
| Q6ZRS2 | Helicase SRCAP | SRCAP | 1.46 | 2 | | 343.3 | | 5.96 | | 0.00 | 0 | | 0.00 | | 0 | | 0.00 | | 0 | | 0.17 | | 1 | | 0.00 | | 0 | | 0.18 | | 1 | | 0.00 | | 0 | | 0.00 | | 0 | | 1 | | | 1 | | 2 | |  |  |  |  |
| A0A0A0MRC8 | Potassium voltage-gated channel subfamily KQT member 5 | KCNQ5 | 3.47 | 2 | | 104.5 | | 9.58 | | 0.00 | 0 | | 0.00 | | 0 | | 0.00 | | 0 | | 0.17 | | 1 | | 0.00 | | 0 | | 0.24 | | 1 | | 0.00 | | 0 | | 0.00 | | 0 | | 1 | | | 1 | | 2 | |  |  |  |  |
| O75925 | E3 SUMO-protein ligase PIAS1 | PIAS1 | 9.83 | 2 | | 71.8 | | 7.30 | | 0.00 | 0 | | 0.00 | | 0 | | 0.00 | | 0 | | 0.17 | | 1 | | 0.00 | | 0 | | 0.00 | | 0 | | 0.17 | | 1 | | 0.00 | | 0 | | 1 | | | 1 | | 2 | |  |  |  |  |
| Q9UKA1 | F-box/LRR-repeat protein 5 | FBXL5 | 1.01 | 1 | | 78.5 | | 5.50 | | 0.00 | 0 | | 0.00 | | 0 | | 0.00 | | 0 | | 0.17 | | 1 | | 0.00 | | 0 | | 0.00 | | 0 | | 0.17 | | 1 | | 0.00 | | 0 | | 1 | | | 1 | | 2 | |  |  |  |  |
| A0A0G2JNH7 | Ephrin type-B receptor 6 | EPHB6 | 5.09 | 2 | | 110.7 | | 6.65 | | 0.00 | 0 | | 0.00 | | 0 | | 0.00 | | 0 | | 0.17 | | 1 | | 0.00 | | 0 | | 0.00 | | 0 | | 0.17 | | 1 | | 0.00 | | 0 | | 1 | | | 1 | | 2 | |  |  |  |  |
| O15067 | Phosphoribosylformylglycinamidine synthase | PFAS | 3.74 | 2 | | 144.6 | | 5.76 | | 0.00 | 0 | | 0.00 | | 0 | | 0.00 | | 0 | | 0.17 | | 1 | | 0.00 | | 0 | | 0.00 | | 0 | | 0.15 | | 1 | | 0.00 | | 0 | | 1 | | | 1 | | 2 | |  |  |  |  |
| Q56P42 | Pyrin domain-containing protein 2 | PYDC2 | 38.14 | 2 | | 10.8 | | 5.36 | | 0.00 | 0 | | 0.00 | | 0 | | 0.00 | | 0 | | 0.17 | | 1 | | 0.00 | | 0 | | 0.00 | | 0 | | 0.14 | | 1 | | 0.00 | | 0 | | 1 | | | 1 | | 2 | |  |  |  |  |
| A0A087WYX6 | Disrupted in schizophrenia 1 protein | DISC1 | 12.43 | 2 | | 79.7 | | 6.84 | | 0.00 | 0 | | 0.00 | | 0 | | 0.00 | | 0 | | 0.17 | | 1 | | 0.00 | | 0 | | 0.00 | | 0 | | 0.14 | | 1 | | 0.00 | | 0 | | 1 | | | 1 | | 2 | |  |  |  |  |
| O76094 | Sial recoition particle subunit SRP72 | SRP72 | 5.37 | 2 | | 74.6 | | 9.26 | | 0.00 | 0 | | 0.00 | | 0 | | 0.00 | | 0 | | 0.16 | | 1 | | 0.14 | | 1 | | 0.00 | | 0 | | 0.00 | | 0 | | 0.00 | | 0 | | 1 | | | 1 | | 2 | |  |  |  |  |
| Q8N1K5 | Protein THEMIS | THEMIS | 9.20 | 2 | | 73.4 | | 5.90 | | 0.00 | 0 | | 0.00 | | 0 | | 0.00 | | 0 | | 0.16 | | 1 | | 0.14 | | 1 | | 0.00 | | 0 | | 0.00 | | 0 | | 0.00 | | 0 | | 1 | | | 1 | | 2 | |  |  |  |  |
| A0A0J9YXC7 | LIM and senescent cell antigen-like-containing domain protein | LIMS4 | 5.28 | 1 | | 45.7 | | 7.88 | | 0.00 | 0 | | 0.00 | | 0 | | 0.00 | | 0 | | 0.16 | | 1 | | 0.16 | | 1 | | 0.00 | | 0 | | 0.00 | | 0 | | 0.00 | | 0 | | 1 | | | 1 | | 2 | |  |  |  |  |
| Q12893 | Transmembrane protein 115 | TMEM115 | 15.95 | 2 | | 38.2 | | 8.16 | | 0.00 | 0 | | 0.00 | | 0 | | 0.00 | | 0 | | 0.16 | | 1 | | 0.00 | | 0 | | 0.18 | | 1 | | 0.00 | | 0 | | 0.00 | | 0 | | 1 | | | 1 | | 2 | |  |  |  |  |
| P18858 | DNA ligase 1 | LIG1 | 5.44 | 2 | | 101.7 | | 5.62 | | 0.00 | 0 | | 0.00 | | 0 | | 0.00 | | 0 | | 0.16 | | 1 | | 0.00 | | 0 | | 0.00 | | 0 | | 0.14 | | 1 | | 0.00 | | 0 | | 1 | | | 1 | | 2 | |  |  |  |  |
| K7EKI0 | Envoplakin | EVPL | 3.11 | 2 | | 233.7 | | 7.25 | | 0.00 | 0 | | 0.00 | | 0 | | 0.00 | | 0 | | 0.16 | | 1 | | 0.00 | | 0 | | 0.00 | | 0 | | 0.00 | | 0 | | 0.15 | | 1 | | 1 | | | 1 | | 2 | |  |  |  |  |
| P29375 | Lysine-specific demethylase 5A | KDM5A | 1.01 | 1 | | 192.0 | | 6.49 | | 0.00 | 0 | | 0.00 | | 0 | | 0.00 | | 0 | | 0.16 | | 1 | | 0.00 | | 0 | | 0.00 | | 0 | | 0.00 | | 0 | | 0.15 | | 1 | | 1 | | | 1 | | 2 | |  |  |  |  |
| A0A087X255 | E3 ubiquitin-protein ligase Midline-1 | MID1 | 8.33 | 2 | | 61.7 | | 7.68 | | 0.00 | 0 | | 0.00 | | 0 | | 0.00 | | 0 | | 0.16 | | 1 | | 0.00 | | 0 | | 0.00 | | 0 | | 0.00 | | 0 | | 0.15 | | 1 | | 1 | | | 1 | | 2 | |  |  |  |  |
| Q5VT66 | Mitochondrial amidoxime-reducing component 1 | MTARC1 | 8.31 | 2 | | 37.5 | | 8.88 | | 0.00 | 0 | | 0.00 | | 0 | | 0.00 | | 0 | | 0.16 | | 1 | | 0.14 | | 1 | | 0.00 | | 0 | | 0.00 | | 0 | | 0.00 | | 0 | | 1 | | | 1 | | 2 | |  |  |  |  |
| Q5HYK3 | 2-methoxy-6-polyprenyl-1,4-benzoquinol methylase, mitochondrial | COQ5 | 13.46 | 2 | | 37.1 | | 6.95 | | 0.00 | 0 | | 0.00 | | 0 | | 0.00 | | 0 | | 0.16 | | 1 | | 0.14 | | 1 | | 0.00 | | 0 | | 0.00 | | 0 | | 0.00 | | 0 | | 1 | | | 1 | | 2 | |  |  |  |  |
| Q9Y2X7 | ARF GTPase-activating protein GIT1 | GIT1 | 4.86 | 2 | | 84.3 | | 6.80 | | 0.00 | 0 | | 0.00 | | 0 | | 0.00 | | 0 | | 0.16 | | 1 | | 0.16 | | 1 | | 0.00 | | 0 | | 0.00 | | 0 | | 0.00 | | 0 | | 1 | | | 1 | | 2 | |  |  |  |  |
| A6NKB5 | Pecanex-like protein 2 | PCNX2 | 2.11 | 2 | | 237.1 | | 6.76 | | 0.00 | 0 | | 0.00 | | 0 | | 0.00 | | 0 | | 0.16 | | 1 | | 0.00 | | 0 | | 0.16 | | 1 | | 0.00 | | 0 | | 0.00 | | 0 | | 1 | | | 1 | | 2 | |  |  |  |  |
| O14718 | Visual pigment-like receptor peropsin | RRH | 2.97 | 1 | | 37.4 | | 8.48 | | 0.00 | 0 | | 0.00 | | 0 | | 0.00 | | 0 | | 0.16 | | 1 | | 0.00 | | 0 | | 0.18 | | 1 | | 0.00 | | 0 | | 0.00 | | 0 | | 1 | | | 1 | | 2 | |  |  |  |  |
| Q14790 | Caspase-8 | CASP8 | 5.85 | 2 | | 55.4 | | 5.10 | | 0.00 | 0 | | 0.00 | | 0 | | 0.00 | | 0 | | 0.16 | | 1 | | 0.00 | | 0 | | 0.00 | | 0 | | 0.17 | | 1 | | 0.00 | | 0 | | 1 | | | 1 | | 2 | |  |  |  |  |
| O75486 | Transcription initiation protein SPT3 homolog | SUPT3H | 16.09 | 2 | | 35.8 | | 6.87 | | 0.00 | 0 | | 0.00 | | 0 | | 0.00 | | 0 | | 0.16 | | 1 | | 0.00 | | 0 | | 0.00 | | 0 | | 0.15 | | 1 | | 0.00 | | 0 | | 1 | | | 1 | | 2 | |  |  |  |  |
| Q2TAZ0 | Autophagy-related protein 2 homolog A | ATG2A | 2.43 | 2 | | 212.7 | | 5.88 | | 0.00 | 0 | | 0.00 | | 0 | | 0.00 | | 0 | | 0.16 | | 1 | | 0.00 | | 0 | | 0.00 | | 0 | | 0.00 | | 0 | | 0.15 | | 1 | | 1 | | | 1 | | 2 | |  |  |  |  |
| Q13936 | Voltage-dependent L-type calcium channel subunit alpha-1C | CACNA1C | 3.74 | 2 | | 248.8 | | 6.76 | | 0.00 | 0 | | 0.00 | | 0 | | 0.00 | | 0 | | 0.16 | | 1 | | 0.16 | | 1 | | 0.00 | | 0 | | 0.00 | | 0 | | 0.00 | | 0 | | 1 | | | 1 | | 2 | |  |  |  |  |
| Q9P2G1 | Ankyrin repeat and IBR domain-containing protein 1 | ANKIB1 | 4.22 | 2 | | 121.9 | | 5.11 | | 0.00 | 0 | | 0.00 | | 0 | | 0.00 | | 0 | | 0.16 | | 1 | | 0.00 | | 0 | | 0.16 | | 1 | | 0.00 | | 0 | | 0.00 | | 0 | | 1 | | | 1 | | 2 | |  |  |  |  |
| Q8WXI7 | Mucin-16 | MUC16 | 0.21 | 2 | | 1518.2 | | 5.26 | | 0.00 | 0 | | 0.00 | | 0 | | 0.00 | | 0 | | 0.16 | | 1 | | 0.00 | | 0 | | 0.24 | | 1 | | 0.00 | | 0 | | 0.00 | | 0 | | 1 | | | 1 | | 2 | |  |  |  |  |
| Q8TDZ2 | [F-actin]-monooxygenase MICAL1 | MICAL1 | 3.47 | 2 | | 117.8 | | 6.40 | | 0.00 | 0 | | 0.00 | | 0 | | 0.00 | | 0 | | 0.16 | | 1 | | 0.00 | | 0 | | 0.00 | | 0 | | 0.17 | | 1 | | 0.00 | | 0 | | 1 | | | 1 | | 2 | |  |  |  |  |
| Q8NBJ5 | Procollagen galactosyltransferase 1 | COLGALT1 | 4.02 | 2 | | 71.6 | | 7.31 | | 0.00 | 0 | | 0.00 | | 0 | | 0.00 | | 0 | | 0.16 | | 1 | | 0.00 | | 0 | | 0.00 | | 0 | | 0.00 | | 0 | | 0.15 | | 1 | | 1 | | | 1 | | 2 | |  |  |  |  |
| P52209 | 6-phosphogluconate dehydrogenase, decarboxylating | PGD | 18.43 | 2 | | 53.1 | | 7.23 | | 0.00 | 0 | | 1.07 | | 2 | | 0.00 | | 0 | | 0.00 | | 0 | | 0.00 | | 0 | | 0.00 | | 0 | | 0.73 | | 3 | | 0.00 | | 0 | | 1 | | | 1 | | 2 | |  |  |  |  |
| Q99584 | Protein S100-A13 | S100A13 | 27.55 | 2 | | 11.5 | | 6.16 | | 0.00 | 0 | | 0.27 | | 2 | | 0.00 | | 0 | | 0.00 | | 0 | | 0.00 | | 0 | | 0.00 | | 0 | | 0.00 | | 0 | | 0.76 | | 3 | | 1 | | | 1 | | 2 | |  |  |  |  |
| K7EM49 | 6-phosphogluconate dehydrogenase, decarboxylating (Fragment) | PGD | 44.39 | 1 | | 22.2 | | 6.39 | | 0.00 | 0 | | 0.13 | | 1 | | 0.00 | | 0 | | 0.00 | | 0 | | 0.00 | | 0 | | 0.00 | | 0 | | 0.73 | | 3 | | 0.00 | | 0 | | 1 | | | 1 | | 2 | |  |  |  |  |
| O60292 | Sial-induced proliferation-associated 1-like protein 3 | SIPA1L3 | 2.19 | 2 | | 194.5 | | 8.32 | | 0.00 | 0 | | 0.27 | | 2 | | 0.00 | | 0 | | 0.00 | | 0 | | 0.00 | | 0 | | 0.23 | | 1 | | 0.00 | | 0 | | 0.00 | | 0 | | 1 | | | 1 | | 2 | |  |  |  |  |
| Q96T58 | Msx2-interacting protein | SPEN | 1.53 | 3 | | 402.0 | | 7.64 | | 0.00 | 0 | | 0.14 | | 1 | | 0.00 | | 0 | | 0.00 | | 0 | | 0.00 | | 0 | | 0.00 | | 0 | | 0.29 | | 2 | | 0.00 | | 0 | | 1 | | | 1 | | 2 | |  |  |  |  |
| Q92918 | Mitogen-activated protein kinase kinase kinase kinase 1 | MAP4K1 | 1.44 | 1 | | 91.2 | | 8.34 | | 0.00 | 0 | | 0.30 | | 2 | | 0.00 | | 0 | | 0.00 | | 0 | | 0.00 | | 0 | | 0.00 | | 0 | | 0.15 | | 1 | | 0.00 | | 0 | | 1 | | | 1 | | 2 | |  |  |  |  |
| P16591 | Tyrosine-protein kinase Fer | FER | 3.16 | 1 | | 94.6 | | 7.14 | | 0.00 | 0 | | 0.27 | | 2 | | 0.00 | | 0 | | 0.00 | | 0 | | 0.00 | | 0 | | 0.00 | | 0 | | 0.00 | | 0 | | 0.18 | | 1 | | 1 | | | 1 | | 2 | |  |  |  |  |
| Q9Y6J8 | Serine/threonine/tyrosine-interacting-like protein 1 | STYXL1 | 14.38 | 2 | | 35.8 | | 6.14 | | 0.00 | 0 | | 0.27 | | 2 | | 0.00 | | 0 | | 0.00 | | 0 | | 0.00 | | 0 | | 0.00 | | 0 | | 0.00 | | 0 | | 0.15 | | 1 | | 1 | | | 1 | | 2 | |  |  |  |  |
| Q9HBR0 | Putative sodium-coupled neutral amino acid transporter 10 | SLC38A10 | 0.98 | 1 | | 119.7 | | 5.73 | | 0.00 | 0 | | 0.14 | | 1 | | 0.00 | | 0 | | 0.00 | | 0 | | 0.00 | | 0 | | 0.00 | | 0 | | 0.00 | | 0 | | 0.30 | | 2 | | 1 | | | 1 | | 2 | |  |  |  |  |
| Q92736 | Ryanodine receptor 2 | RYR2 | 1.39 | 3 | | 564.2 | | 6.07 | | 0.00 | 0 | | 0.14 | | 1 | | 0.00 | | 0 | | 0.00 | | 0 | | 0.00 | | 0 | | 0.47 | | 1 | | 0.00 | | 0 | | 0.00 | | 0 | | 1 | | | 1 | | 2 | |  |  |  |  |
| Q9UFH2 | Dynein heavy chain 17, axonemal | DNAH17 | 0.94 | 2 | | 509.0 | | 5.72 | | 0.00 | 0 | | 0.13 | | 1 | | 0.00 | | 0 | | 0.00 | | 0 | | 0.14 | | 1 | | 0.00 | | 0 | | 0.00 | | 0 | | 0.00 | | 0 | | 1 | | | 1 | | 2 | |  |  |  |  |
| O14744 | Protein arginine N-methyltransferase 5 | PRMT5 | 8.95 | 2 | | 72.6 | | 6.29 | | 0.00 | 0 | | 0.16 | | 1 | | 0.00 | | 0 | | 0.00 | | 0 | | 0.14 | | 1 | | 0.00 | | 0 | | 0.00 | | 0 | | 0.00 | | 0 | | 1 | | | 1 | | 2 | |  |  |  |  |
| P19021 | Peptidyl-glycine alpha-amidating monooxygenase | PAM | 6.37 | 2 | | 108.3 | | 6.42 | | 0.00 | 0 | | 0.14 | | 1 | | 0.00 | | 0 | | 0.00 | | 0 | | 0.14 | | 1 | | 0.00 | | 0 | | 0.00 | | 0 | | 0.00 | | 0 | | 1 | | | 1 | | 2 | |  |  |  |  |
| Q9Y4D1 | Disheveled-associated activator of morphogenesis 1 | DAAM1 | 2.97 | 2 | | 123.4 | | 7.23 | | 0.00 | 0 | | 0.14 | | 1 | | 0.00 | | 0 | | 0.00 | | 0 | | 0.14 | | 1 | | 0.00 | | 0 | | 0.00 | | 0 | | 0.00 | | 0 | | 1 | | | 1 | | 2 | |  |  |  |  |
| H3BNR1 | BORCS8-MEF2B readthrough | BORCS8-MEF2B | 14.40 | 2 | | 40.4 | | 9.47 | | 0.00 | 0 | | 0.14 | | 1 | | 0.00 | | 0 | | 0.00 | | 0 | | 0.14 | | 1 | | 0.00 | | 0 | | 0.00 | | 0 | | 0.00 | | 0 | | 1 | | | 1 | | 2 | |  |  |  |  |
| Q0VDD8 | Dynein heavy chain 14, axonemal | DNAH14 | 1.80 | 2 | | 399.6 | | 6.93 | | 0.00 | 0 | | 0.14 | | 1 | | 0.00 | | 0 | | 0.00 | | 0 | | 0.14 | | 1 | | 0.00 | | 0 | | 0.00 | | 0 | | 0.00 | | 0 | | 1 | | | 1 | | 2 | |  |  |  |  |
| Q5W149 | Nucleolar protein 4-like (Fragment) | NOL4L | 13.65 | 1 | | 27.3 | | 8.28 | | 0.00 | 0 | | 0.13 | | 1 | | 0.00 | | 0 | | 0.00 | | 0 | | 0.14 | | 1 | | 0.00 | | 0 | | 0.00 | | 0 | | 0.00 | | 0 | | 1 | | | 1 | | 2 | |  |  |  |  |
| J3KPF0 | Probable E3 ubiquitin-protein ligase HECTD4 | HECTD4 | 0.68 | 1 | | 484.0 | | 6.02 | | 0.00 | 0 | | 0.14 | | 1 | | 0.00 | | 0 | | 0.00 | | 0 | | 0.14 | | 1 | | 0.00 | | 0 | | 0.00 | | 0 | | 0.00 | | 0 | | 1 | | | 1 | | 2 | |  |  |  |  |
| A0A494C150 | Probable E3 ubiquitin-protein ligase HECTD4 (Fragment) | HECTD4 | 1.70 | 1 | | 199.2 | | 6.38 | | 0.00 | 0 | | 0.14 | | 1 | | 0.00 | | 0 | | 0.00 | | 0 | | 0.14 | | 1 | | 0.00 | | 0 | | 0.00 | | 0 | | 0.00 | | 0 | | 1 | | | 1 | | 2 | |  |  |  |  |
| Q9UIY3 | RWD domain-containing protein 2A | RWDD2A | 14.73 | 2 | | 33.9 | | 6.47 | | 0.00 | 0 | | 0.14 | | 1 | | 0.00 | | 0 | | 0.00 | | 0 | | 0.16 | | 1 | | 0.00 | | 0 | | 0.00 | | 0 | | 0.00 | | 0 | | 1 | | | 1 | | 2 | |  |  |  |  |
| Q4LE39 | AT-rich interactive domain-containing protein 4B | ARID4B | 2.90 | 2 | | 147.7 | | 5.12 | | 0.00 | 0 | | 0.13 | | 1 | | 0.00 | | 0 | | 0.00 | | 0 | | 0.16 | | 1 | | 0.00 | | 0 | | 0.00 | | 0 | | 0.00 | | 0 | | 1 | | | 1 | | 2 | |  |  |  |  |
| Q7Z5J4 | Retinoic acid-induced protein 1 | RAI1 | 1.47 | 1 | | 203.2 | | 8.79 | | 0.00 | 0 | | 0.14 | | 1 | | 0.00 | | 0 | | 0.00 | | 0 | | 0.16 | | 1 | | 0.00 | | 0 | | 0.00 | | 0 | | 0.00 | | 0 | | 1 | | | 1 | | 2 | |  |  |  |  |
| P08311 | Cathepsin G | CTSG | 5.10 | 1 | | 28.8 | | 11.19 | | 0.00 | 0 | | 0.14 | | 1 | | 0.00 | | 0 | | 0.00 | | 0 | | 0.14 | | 1 | | 0.00 | | 0 | | 0.00 | | 0 | | 0.00 | | 0 | | 1 | | | 1 | | 2 | |  |  |  |  |
| Q86YW9 | Mediator of RNA polymerase II transcription subunit 12-like protein | MED12L | 2.19 | 2 | | 240.0 | | 7.77 | | 0.00 | 0 | | 0.13 | | 1 | | 0.00 | | 0 | | 0.00 | | 0 | | 0.14 | | 1 | | 0.00 | | 0 | | 0.00 | | 0 | | 0.00 | | 0 | | 1 | | | 1 | | 2 | |  |  |  |  |
| P32926 | Desmoglein-3 | DSG3 | 3.10 | 2 | | 107.5 | | 5.00 | | 0.00 | 0 | | 0.13 | | 1 | | 0.00 | | 0 | | 0.00 | | 0 | | 0.14 | | 1 | | 0.00 | | 0 | | 0.00 | | 0 | | 0.00 | | 0 | | 1 | | | 1 | | 2 | |  |  |  |  |
| Q99645 | Epiphycan | EPYC | 8.70 | 2 | | 36.6 | | 4.81 | | 0.00 | 0 | | 0.13 | | 1 | | 0.00 | | 0 | | 0.00 | | 0 | | 0.00 | | 0 | | 0.16 | | 1 | | 0.00 | | 0 | | 0.00 | | 0 | | 1 | | | 1 | | 2 | |  |  |  |  |
| A0A1W2PS05 | Phosphatidylinositol 3-kinase catalytic subunit type 3 | PIK3C3 | 2.25 | 1 | | 101.6 | | 6.90 | | 0.00 | 0 | | 0.14 | | 1 | | 0.00 | | 0 | | 0.00 | | 0 | | 0.00 | | 0 | | 0.16 | | 1 | | 0.00 | | 0 | | 0.00 | | 0 | | 1 | | | 1 | | 2 | |  |  |  |  |
| O75874 | Isocitrate dehydrogenase [NADP] cytoplasmic | IDH1 | 3.14 | 1 | | 46.6 | | 7.01 | | 0.00 | 0 | | 0.14 | | 1 | | 0.00 | | 0 | | 0.00 | | 0 | | 0.00 | | 0 | | 0.16 | | 1 | | 0.00 | | 0 | | 0.00 | | 0 | | 1 | | | 1 | | 2 | |  |  |  |  |
| Q7Z2Y8 | Interferon-induced very large GTPase 1 | GVINP1 | 2.31 | 2 | | 278.9 | | 6.55 | | 0.00 | 0 | | 0.16 | | 1 | | 0.00 | | 0 | | 0.00 | | 0 | | 0.00 | | 0 | | 0.16 | | 1 | | 0.00 | | 0 | | 0.00 | | 0 | | 1 | | | 1 | | 2 | |  |  |  |  |
| Q5HYW3 | Retrotransposon Gag-like protein 5 | RTL5 | 2.11 | 1 | | 64.7 | | 4.75 | | 0.00 | 0 | | 0.16 | | 1 | | 0.00 | | 0 | | 0.00 | | 0 | | 0.00 | | 0 | | 0.16 | | 1 | | 0.00 | | 0 | | 0.00 | | 0 | | 1 | | | 1 | | 2 | |  |  |  |  |
| Q9H221 | ATP-binding cassette sub-family G member 8 | ABCG8 | 2.82 | 2 | | 75.6 | | 8.22 | | 0.00 | 0 | | 0.13 | | 1 | | 0.00 | | 0 | | 0.00 | | 0 | | 0.00 | | 0 | | 0.16 | | 1 | | 0.00 | | 0 | | 0.00 | | 0 | | 1 | | | 1 | | 2 | |  |  |  |  |
| P48742 | LIM/homeobox protein Lhx1 | LHX1 | 6.40 | 2 | | 44.8 | | 7.72 | | 0.00 | 0 | | 0.14 | | 1 | | 0.00 | | 0 | | 0.00 | | 0 | | 0.00 | | 0 | | 0.16 | | 1 | | 0.00 | | 0 | | 0.00 | | 0 | | 1 | | | 1 | | 2 | |  |  |  |  |
| Q9UJX6 | Anaphase-promoting complex subunit 2 | ANAPC2 | 3.65 | 2 | | 93.8 | | 5.22 | | 0.00 | 0 | | 0.14 | | 1 | | 0.00 | | 0 | | 0.00 | | 0 | | 0.00 | | 0 | | 0.18 | | 1 | | 0.00 | | 0 | | 0.00 | | 0 | | 1 | | | 1 | | 2 | |  |  |  |  |
| O94964 | Protein SOGA1 | SOGA1 | 1.41 | 2 | | 159.7 | | 6.46 | | 0.00 | 0 | | 0.13 | | 1 | | 0.00 | | 0 | | 0.00 | | 0 | | 0.00 | | 0 | | 0.18 | | 1 | | 0.00 | | 0 | | 0.00 | | 0 | | 1 | | | 1 | | 2 | |  |  |  |  |
| A0A2R8YD71 | Natural resistance-associated macrophage protein 2 | SLC11A2 | 5.24 | 1 | | 62.7 | | 6.61 | | 0.00 | 0 | | 0.14 | | 1 | | 0.00 | | 0 | | 0.00 | | 0 | | 0.00 | | 0 | | 0.18 | | 1 | | 0.00 | | 0 | | 0.00 | | 0 | | 1 | | | 1 | | 2 | |  |  |  |  |
| P14410 | Sucrase-isomaltase, intestinal | SI | 1.70 | 2 | | 209.3 | | 5.64 | | 0.00 | 0 | | 0.14 | | 1 | | 0.00 | | 0 | | 0.00 | | 0 | | 0.00 | | 0 | | 0.23 | | 1 | | 0.00 | | 0 | | 0.00 | | 0 | | 1 | | | 1 | | 2 | |  |  |  |  |
| O75746 | Calcium-binding mitochondrial carrier protein Aralar1 | SLC25A12 | 2.51 | 1 | | 74.7 | | 8.38 | | 0.00 | 0 | | 0.14 | | 1 | | 0.00 | | 0 | | 0.00 | | 0 | | 0.00 | | 0 | | 0.23 | | 1 | | 0.00 | | 0 | | 0.00 | | 0 | | 1 | | | 1 | | 2 | |  |  |  |  |
| Q5TAH2 | Sodium/hydrogen exchanger 11 | SLC9C2 | 4.45 | 2 | | 129.0 | | 6.92 | | 0.00 | 0 | | 0.16 | | 1 | | 0.00 | | 0 | | 0.00 | | 0 | | 0.00 | | 0 | | 0.23 | | 1 | | 0.00 | | 0 | | 0.00 | | 0 | | 1 | | | 1 | | 2 | |  |  |  |  |
| A0A0A0MR25 | Fibroblast growth factor receptor | FGFR2 | 4.14 | 2 | | 92.0 | | 5.88 | | 0.00 | 0 | | 0.16 | | 1 | | 0.00 | | 0 | | 0.00 | | 0 | | 0.00 | | 0 | | 0.23 | | 1 | | 0.00 | | 0 | | 0.00 | | 0 | | 1 | | | 1 | | 2 | |  |  |  |  |
| F8W1F2 | Natural resistance-associated macrophage protein 2 (Fragment) | SLC11A2 | 29.33 | 1 | | 16.5 | | 6.54 | | 0.00 | 0 | | 0.14 | | 1 | | 0.00 | | 0 | | 0.00 | | 0 | | 0.00 | | 0 | | 0.23 | | 1 | | 0.00 | | 0 | | 0.00 | | 0 | | 1 | | | 1 | | 2 | |  |  |  |  |
| Q709C8 | Vacuolar protein sorting-associated protein 13C | VPS13C | 0.53 | 1 | | 422.1 | | 6.83 | | 0.00 | 0 | | 0.14 | | 1 | | 0.00 | | 0 | | 0.00 | | 0 | | 0.00 | | 0 | | 0.23 | | 1 | | 0.00 | | 0 | | 0.00 | | 0 | | 1 | | | 1 | | 2 | |  |  |  |  |
| Q9UF83 | Uncharacterized protein DKFZp434B061 |  | 7.98 | 2 | | 59.4 | | 13.07 | | 0.00 | 0 | | 0.16 | | 1 | | 0.00 | | 0 | | 0.00 | | 0 | | 0.00 | | 0 | | 0.24 | | 1 | | 0.00 | | 0 | | 0.00 | | 0 | | 1 | | | 1 | | 2 | |  |  |  |  |
| Q8IWY7 | Tau-tubulin kinase | TTBK2 | 1.52 | 1 | | 182.4 | | 7.02 | | 0.00 | 0 | | 0.14 | | 1 | | 0.00 | | 0 | | 0.00 | | 0 | | 0.00 | | 0 | | 0.00 | | 0 | | 0.15 | | 1 | | 0.00 | | 0 | | 1 | | | 1 | | 2 | |  |  |  |  |
| P52948 | Nuclear pore complex protein Nup98-Nup96 | NUP98 | 1.87 | 2 | | 197.5 | | 6.40 | | 0.00 | 0 | | 0.14 | | 1 | | 0.00 | | 0 | | 0.00 | | 0 | | 0.00 | | 0 | | 0.00 | | 0 | | 0.15 | | 1 | | 0.00 | | 0 | | 1 | | | 1 | | 2 | |  |  |  |  |
| Q15293 | Reticulocalbin-1 | RCN1 | 15.41 | 2 | | 38.9 | | 5.00 | | 0.00 | 0 | | 0.16 | | 1 | | 0.00 | | 0 | | 0.00 | | 0 | | 0.00 | | 0 | | 0.00 | | 0 | | 0.15 | | 1 | | 0.00 | | 0 | | 1 | | | 1 | | 2 | |  |  |  |  |
| Q8IYB4 | PEX5-related protein | PEX5L | 10.22 | 2 | | 69.7 | | 5.29 | | 0.00 | 0 | | 0.14 | | 1 | | 0.00 | | 0 | | 0.00 | | 0 | | 0.00 | | 0 | | 0.00 | | 0 | | 0.15 | | 1 | | 0.00 | | 0 | | 1 | | | 1 | | 2 | |  |  |  |  |
| Q504Y3 | Zinc finger CW-type PWWP domain protein 2 | ZCWPW2 | 10.96 | 2 | | 41.3 | | 5.78 | | 0.00 | 0 | | 0.14 | | 1 | | 0.00 | | 0 | | 0.00 | | 0 | | 0.00 | | 0 | | 0.00 | | 0 | | 0.15 | | 1 | | 0.00 | | 0 | | 1 | | | 1 | | 2 | |  |  |  |  |
| P52701 | DNA mismatch repair protein Msh6 | MSH6 | 1.40 | 2 | | 152.7 | | 6.90 | | 0.00 | 0 | | 0.14 | | 1 | | 0.00 | | 0 | | 0.00 | | 0 | | 0.00 | | 0 | | 0.00 | | 0 | | 0.15 | | 1 | | 0.00 | | 0 | | 1 | | | 1 | | 2 | |  |  |  |  |
| Q8N6I1 | EP300-interacting inhibitor of differentiation 2 | EID2 | 16.95 | 2 | | 25.2 | | 7.46 | | 0.00 | 0 | | 0.14 | | 1 | | 0.00 | | 0 | | 0.00 | | 0 | | 0.00 | | 0 | | 0.00 | | 0 | | 0.14 | | 1 | | 0.00 | | 0 | | 1 | | | 1 | | 2 | |  |  |  |  |
| P46063 | ATP-dependent DNA helicase Q1 | RECQL | 3.70 | 1 | | 73.4 | | 7.88 | | 0.00 | 0 | | 0.14 | | 1 | | 0.00 | | 0 | | 0.00 | | 0 | | 0.00 | | 0 | | 0.00 | | 0 | | 0.14 | | 1 | | 0.00 | | 0 | | 1 | | | 1 | | 2 | |  |  |  |  |
| Q5HYW2 | NHS-like protein 2 | NHSL2 | 6.53 | 2 | | 133.2 | | 7.77 | | 0.00 | 0 | | 0.14 | | 1 | | 0.00 | | 0 | | 0.00 | | 0 | | 0.00 | | 0 | | 0.00 | | 0 | | 0.14 | | 1 | | 0.00 | | 0 | | 1 | | | 1 | | 2 | |  |  |  |  |
| O00219 | Hyaluronan synthase 3 | HAS3 | 6.51 | 2 | | 63.0 | | 8.48 | | 0.00 | 0 | | 0.16 | | 1 | | 0.00 | | 0 | | 0.00 | | 0 | | 0.00 | | 0 | | 0.00 | | 0 | | 0.14 | | 1 | | 0.00 | | 0 | | 1 | | | 1 | | 2 | |  |  |  |  |
| Q5VWM4 | PRAME family member 8 | PRAMEF8 | 8.86 | 2 | | 53.6 | | 6.38 | | 0.00 | 0 | | 0.14 | | 1 | | 0.00 | | 0 | | 0.00 | | 0 | | 0.00 | | 0 | | 0.00 | | 0 | | 0.14 | | 1 | | 0.00 | | 0 | | 1 | | | 1 | | 2 | |  |  |  |  |
| P30622 | CAP-Gly domain-containing linker protein 1 | CLIP1 | 1.95 | 2 | | 162.1 | | 5.36 | | 0.00 | 0 | | 0.14 | | 1 | | 0.00 | | 0 | | 0.00 | | 0 | | 0.00 | | 0 | | 0.00 | | 0 | | 0.14 | | 1 | | 0.00 | | 0 | | 1 | | | 1 | | 2 | |  |  |  |  |
| O75179 | Ankyrin repeat domain-containing protein 17 | ANKRD17 | 1.96 | 2 | | 274.1 | | 6.52 | | 0.00 | 0 | | 0.16 | | 1 | | 0.00 | | 0 | | 0.00 | | 0 | | 0.00 | | 0 | | 0.00 | | 0 | | 0.14 | | 1 | | 0.00 | | 0 | | 1 | | | 1 | | 2 | |  |  |  |  |
| Q8TDW7 | Protocadherin Fat 3 | FAT3 | 0.90 | 2 | | 501.7 | | 4.87 | | 0.00 | 0 | | 0.13 | | 1 | | 0.00 | | 0 | | 0.00 | | 0 | | 0.00 | | 0 | | 0.00 | | 0 | | 0.14 | | 1 | | 0.00 | | 0 | | 1 | | | 1 | | 2 | |  |  |  |  |
| Q6YHK3 | CD109 antigen | CD109 | 1.04 | 1 | | 161.6 | | 5.85 | | 0.00 | 0 | | 0.14 | | 1 | | 0.00 | | 0 | | 0.00 | | 0 | | 0.00 | | 0 | | 0.00 | | 0 | | 0.14 | | 1 | | 0.00 | | 0 | | 1 | | | 1 | | 2 | |  |  |  |  |
| Q9UPT8 | Zinc finger CCCH domain-containing protein 4 | ZC3H4 | 4.07 | 2 | | 140.2 | | 6.27 | | 0.00 | 0 | | 0.14 | | 1 | | 0.00 | | 0 | | 0.00 | | 0 | | 0.00 | | 0 | | 0.00 | | 0 | | 0.14 | | 1 | | 0.00 | | 0 | | 1 | | | 1 | | 2 | |  |  |  |  |
| Q8IX30 | Sial peptide, CUB and EGF-like domain-containing protein 3 | SCUBE3 | 5.04 | 2 | | 109.2 | | 7.56 | | 0.00 | 0 | | 0.14 | | 1 | | 0.00 | | 0 | | 0.00 | | 0 | | 0.00 | | 0 | | 0.00 | | 0 | | 0.14 | | 1 | | 0.00 | | 0 | | 1 | | | 1 | | 2 | |  |  |  |  |
| Q8NDV3 | Structural maintenance of chromosomes protein 1B | SMC1B | 5.34 | 2 | | 143.8 | | 7.66 | | 0.00 | 0 | | 0.14 | | 1 | | 0.00 | | 0 | | 0.00 | | 0 | | 0.00 | | 0 | | 0.00 | | 0 | | 0.00 | | 0 | | 0.15 | | 1 | | 1 | | | 1 | | 2 | |  |  |  |  |
| Q5VYJ5 | MAM and LDL-receptor class A domain-containing protein 1 | MALRD1 | 1.99 | 2 | | 240.9 | | 5.22 | | 0.00 | 0 | | 0.16 | | 1 | | 0.00 | | 0 | | 0.00 | | 0 | | 0.00 | | 0 | | 0.00 | | 0 | | 0.00 | | 0 | | 0.15 | | 1 | | 1 | | | 1 | | 2 | |  |  |  |  |
| A0A2R8YF49 | Rho guanine nucleotide exchange factor 5 | ARHGEF5 | 2.25 | 2 | | 176.6 | | 5.55 | | 0.00 | 0 | | 0.14 | | 1 | | 0.00 | | 0 | | 0.00 | | 0 | | 0.00 | | 0 | | 0.00 | | 0 | | 0.00 | | 0 | | 0.15 | | 1 | | 1 | | | 1 | | 2 | |  |  |  |  |
| Q9H5I5 | Piezo-type mechanosensitive ion channel component 2 | PIEZO2 | 2.14 | 2 | | 317.9 | | 6.15 | | 0.00 | 0 | | 0.14 | | 1 | | 0.00 | | 0 | | 0.00 | | 0 | | 0.00 | | 0 | | 0.00 | | 0 | | 0.00 | | 0 | | 0.15 | | 1 | | 1 | | | 1 | | 2 | |  |  |  |  |
| A0A2R8Y5B3 | Band 4.1-like protein 2 (Fragment) | EPB41L2 | 4.61 | 2 | | 92.1 | | 5.68 | | 0.00 | 0 | | 0.14 | | 1 | | 0.00 | | 0 | | 0.00 | | 0 | | 0.00 | | 0 | | 0.00 | | 0 | | 0.00 | | 0 | | 0.15 | | 1 | | 1 | | | 1 | | 2 | |  |  |  |  |
| O60518 | Ran-binding protein 6 | RANBP6 | 3.98 | 2 | | 124.6 | | 5.01 | | 0.00 | 0 | | 0.14 | | 1 | | 0.00 | | 0 | | 0.00 | | 0 | | 0.00 | | 0 | | 0.00 | | 0 | | 0.00 | | 0 | | 0.15 | | 1 | | 1 | | | 1 | | 2 | |  |  |  |  |
| Q9Y2W1 | Thyroid hormone receptor-associated protein 3 | THRAP3 | 1.36 | 1 | | 108.6 | | 10.15 | | 0.00 | 0 | | 0.14 | | 1 | | 0.00 | | 0 | | 0.00 | | 0 | | 0.00 | | 0 | | 0.00 | | 0 | | 0.00 | | 0 | | 0.15 | | 1 | | 1 | | | 1 | | 2 | |  |  |  |  |
| D6RE77 | Rap guanine nucleotide exchange factor 6 | RAPGEF6 | 2.51 | 1 | | 126.3 | | 8.95 | | 0.00 | 0 | | 0.13 | | 1 | | 0.00 | | 0 | | 0.00 | | 0 | | 0.00 | | 0 | | 0.00 | | 0 | | 0.00 | | 0 | | 0.15 | | 1 | | 1 | | | 1 | | 2 | |  |  |  |  |
| Q96MW7 | Tigger transposable element-derived protein 1 | TIGD1 | 5.92 | 2 | | 67.3 | | 8.46 | | 0.00 | 0 | | 0.14 | | 1 | | 0.00 | | 0 | | 0.00 | | 0 | | 0.00 | | 0 | | 0.00 | | 0 | | 0.00 | | 0 | | 0.15 | | 1 | | 1 | | | 1 | | 2 | |  |  |  |  |
| A4FU69 | EF-hand calcium-binding domain-containing protein 5 | EFCAB5 | 6.05 | 2 | | 173.3 | | 5.82 | | 0.00 | 0 | | 0.13 | | 1 | | 0.00 | | 0 | | 0.00 | | 0 | | 0.00 | | 0 | | 0.00 | | 0 | | 0.00 | | 0 | | 0.15 | | 1 | | 1 | | | 1 | | 2 | |  |  |  |  |
| Q9BV73 | Centrosome-associated protein CEP250 | CEP250 | 0.70 | 2 | | 281.0 | | 5.02 | | 0.56 | 3 | | 0.00 | | 0 | | 0.00 | | 0 | | 0.00 | | 0 | | 0.00 | | 0 | | 0.00 | | 0 | | 0.00 | | 0 | | 0.00 | | 0 | | 1 | | | 0 | | 1 | |  |  |  |  |
| Q15063 | Periostin | POSTN | 3.35 | 2 | | 93.3 | | 7.53 | | 0.64 | 2 | | 0.00 | | 0 | | 0.00 | | 0 | | 0.00 | | 0 | | 0.00 | | 0 | | 0.00 | | 0 | | 0.00 | | 0 | | 0.00 | | 0 | | 1 | | | 0 | | 1 | |  |  |  |  |
| O60673 | DNA polymerase zeta catalytic subunit | REV3L | 1.47 | 2 | | 352.6 | | 8.47 | | 0.29 | 2 | | 0.00 | | 0 | | 0.00 | | 0 | | 0.00 | | 0 | | 0.00 | | 0 | | 0.00 | | 0 | | 0.00 | | 0 | | 0.00 | | 0 | | 1 | | | 0 | | 1 | |  |  |  |  |
| Q9NZP5 | Olfactory receptor 5AC2 | OR5AC2 | 2.27 | 1 | | 35.3 | | 8.94 | | 0.41 | 2 | | 0.00 | | 0 | | 0.00 | | 0 | | 0.00 | | 0 | | 0.00 | | 0 | | 0.00 | | 0 | | 0.00 | | 0 | | 0.00 | | 0 | | 1 | | | 0 | | 1 | |  |  |  |  |
| P84090 | Enhancer of rudimentary homolog | ERH | 10.58 | 1 | | 12.3 | | 5.92 | | 0.26 | 2 | | 0.00 | | 0 | | 0.00 | | 0 | | 0.00 | | 0 | | 0.00 | | 0 | | 0.00 | | 0 | | 0.00 | | 0 | | 0.00 | | 0 | | 1 | | | 0 | | 1 | |  |  |  |  |
| E7EUW2 | Adhesion G protein-coupled receptor L3 | ADGRL3 | 0.57 | 1 | | 175.9 | | 6.86 | | 0.31 | 1 | | 0.00 | | 0 | | 0.00 | | 0 | | 0.00 | | 0 | | 0.00 | | 0 | | 0.00 | | 0 | | 0.00 | | 0 | | 0.00 | | 0 | | 1 | | | 0 | | 1 | |  |  |  |  |
| Q13308 | Inactive tyrosine-protein kinase 7 | PTK7 | 1.12 | 1 | | 118.3 | | 7.09 | | 0.26 | 1 | | 0.00 | | 0 | | 0.00 | | 0 | | 0.00 | | 0 | | 0.00 | | 0 | | 0.00 | | 0 | | 0.00 | | 0 | | 0.00 | | 0 | | 1 | | | 0 | | 1 | |  |  |  |  |
| Q8WW12 | PEST proteolytic sial-containing nuclear protein | PCNP | 8.99 | 1 | | 18.9 | | 7.49 | | 0.16 | 1 | | 0.00 | | 0 | | 0.00 | | 0 | | 0.00 | | 0 | | 0.00 | | 0 | | 0.00 | | 0 | | 0.00 | | 0 | | 0.00 | | 0 | | 1 | | | 0 | | 1 | |  |  |  |  |
| P49619 | Diacylglycerol kinase gamma | DGKG | 2.40 | 1 | | 89.1 | | 6.80 | | 0.16 | 1 | | 0.00 | | 0 | | 0.00 | | 0 | | 0.00 | | 0 | | 0.00 | | 0 | | 0.00 | | 0 | | 0.00 | | 0 | | 0.00 | | 0 | | 1 | | | 0 | | 1 | |  |  |  |  |
| Q9C0D2 | Centrosomal protein of 295 kDa | CEP295 | 1.08 | 1 | | 295.0 | | 6.00 | | 0.16 | 1 | | 0.00 | | 0 | | 0.00 | | 0 | | 0.00 | | 0 | | 0.00 | | 0 | | 0.00 | | 0 | | 0.00 | | 0 | | 0.00 | | 0 | | 1 | | | 0 | | 1 | |  |  |  |  |
| F5H527 | Protein phosphatase Slingshot homolog 2 | SSH2 | 0.97 | 1 | | 161.0 | | 5.30 | | 0.16 | 1 | | 0.00 | | 0 | | 0.00 | | 0 | | 0.00 | | 0 | | 0.00 | | 0 | | 0.00 | | 0 | | 0.00 | | 0 | | 0.00 | | 0 | | 1 | | | 0 | | 1 | |  |  |  |  |
| P12882 | Myosin-1 | MYH1 | 1.29 | 1 | | 223.0 | | 5.74 | | 0.16 | 1 | | 0.00 | | 0 | | 0.00 | | 0 | | 0.00 | | 0 | | 0.00 | | 0 | | 0.00 | | 0 | | 0.00 | | 0 | | 0.00 | | 0 | | 1 | | | 0 | | 1 | |  |  |  |  |
| Q5R3C7 | Uncharacterized protein C1orf105 | C1orf105 | 15.03 | 1 | | 19.5 | | 8.92 | | 0.16 | 1 | | 0.00 | | 0 | | 0.00 | | 0 | | 0.00 | | 0 | | 0.00 | | 0 | | 0.00 | | 0 | | 0.00 | | 0 | | 0.00 | | 0 | | 1 | | | 0 | | 1 | |  |  |  |  |
| J3QSU1 | Leucine-rich repeat-containing protein 37B | LRRC37B | 1.44 | 1 | | 108.4 | | 4.98 | | 0.16 | 1 | | 0.00 | | 0 | | 0.00 | | 0 | | 0.00 | | 0 | | 0.00 | | 0 | | 0.00 | | 0 | | 0.00 | | 0 | | 0.00 | | 0 | | 1 | | | 0 | | 1 | |  |  |  |  |
| Q9H2X9 | Solute carrier family 12 member 5 | SLC12A5 | 1.05 | 1 | | 126.1 | | 6.73 | | 0.16 | 1 | | 0.00 | | 0 | | 0.00 | | 0 | | 0.00 | | 0 | | 0.00 | | 0 | | 0.00 | | 0 | | 0.00 | | 0 | | 0.00 | | 0 | | 1 | | | 0 | | 1 | |  |  |  |  |
| P10620 | Microsomal glutathione S-transferase 1 | MGST1 | 7.74 | 1 | | 17.6 | | 9.39 | | 0.16 | 1 | | 0.00 | | 0 | | 0.00 | | 0 | | 0.00 | | 0 | | 0.00 | | 0 | | 0.00 | | 0 | | 0.00 | | 0 | | 0.00 | | 0 | | 1 | | | 0 | | 1 | |  |  |  |  |
| F5H5Q0 | ATP-binding cassette sub-family B member 9 (Fragment) | ABCB9 | 47.83 | 1 | | 13.0 | | 7.96 | | 0.16 | 1 | | 0.00 | | 0 | | 0.00 | | 0 | | 0.00 | | 0 | | 0.00 | | 0 | | 0.00 | | 0 | | 0.00 | | 0 | | 0.00 | | 0 | | 1 | | | 0 | | 1 | |  |  |  |  |
| P51681 | C-C chemokine receptor type 5 | CCR5 | 9.09 | 1 | | 40.5 | | 9.00 | | 0.16 | 1 | | 0.00 | | 0 | | 0.00 | | 0 | | 0.00 | | 0 | | 0.00 | | 0 | | 0.00 | | 0 | | 0.00 | | 0 | | 0.00 | | 0 | | 1 | | | 0 | | 1 | |  |  |  |  |
| Q8N4B1 | Sesquipedalian-1 | PHETA1 | 4.02 | 1 | | 27.2 | | 8.98 | | 0.16 | 1 | | 0.00 | | 0 | | 0.00 | | 0 | | 0.00 | | 0 | | 0.00 | | 0 | | 0.00 | | 0 | | 0.00 | | 0 | | 0.00 | | 0 | | 1 | | | 0 | | 1 | |  |  |  |  |
| A0A1B0GTF3 | Heat shock 70 kDa protein 12A | HSPA12A | 4.34 | 1 | | 77.0 | | 7.17 | | 0.16 | 1 | | 0.00 | | 0 | | 0.00 | | 0 | | 0.00 | | 0 | | 0.00 | | 0 | | 0.00 | | 0 | | 0.00 | | 0 | | 0.00 | | 0 | | 1 | | | 0 | | 1 | |  |  |  |  |
| Q9HCM1 | Retroelement silencing factor 1 | RESF1 | 1.26 | 1 | | 194.7 | | 8.78 | | 0.16 | 1 | | 0.00 | | 0 | | 0.00 | | 0 | | 0.00 | | 0 | | 0.00 | | 0 | | 0.00 | | 0 | | 0.00 | | 0 | | 0.00 | | 0 | | 1 | | | 0 | | 1 | |  |  |  |  |
| Q9P2I0 | Cleavage and polyadenylation specificity factor subunit 2 | CPSF2 | 1.53 | 1 | | 88.4 | | 5.11 | | 0.16 | 1 | | 0.00 | | 0 | | 0.00 | | 0 | | 0.00 | | 0 | | 0.00 | | 0 | | 0.00 | | 0 | | 0.00 | | 0 | | 0.00 | | 0 | | 1 | | | 0 | | 1 | |  |  |  |  |
| Q9BRR0 | Zinc finger protein with KRAB and SCAN domains 3 | ZKSCAN3 | 2.60 | 1 | | 60.6 | | 6.40 | | 0.16 | 1 | | 0.00 | | 0 | | 0.00 | | 0 | | 0.00 | | 0 | | 0.00 | | 0 | | 0.00 | | 0 | | 0.00 | | 0 | | 0.00 | | 0 | | 1 | | | 0 | | 1 | |  |  |  |  |
| E9PGQ0 | Solute carrier family 25 member 44 | SLC25A44 | 2.80 | 1 | | 36.3 | | 9.63 | | 0.16 | 1 | | 0.00 | | 0 | | 0.00 | | 0 | | 0.00 | | 0 | | 0.00 | | 0 | | 0.00 | | 0 | | 0.00 | | 0 | | 0.00 | | 0 | | 1 | | | 0 | | 1 | |  |  |  |  |
| Q5TCQ9 | Membrane-associated guanylate kinase, WW and PDZ domain-containing protein 3 | MAGI3 | 0.61 | 1 | | 162.8 | | 8.09 | | 0.16 | 1 | | 0.00 | | 0 | | 0.00 | | 0 | | 0.00 | | 0 | | 0.00 | | 0 | | 0.00 | | 0 | | 0.00 | | 0 | | 0.00 | | 0 | | 1 | | | 0 | | 1 | |  |  |  |  |
| O95197 | Reticulon-3 | RTN3 | 0.87 | 1 | | 112.5 | | 4.96 | | 0.16 | 1 | | 0.00 | | 0 | | 0.00 | | 0 | | 0.00 | | 0 | | 0.00 | | 0 | | 0.00 | | 0 | | 0.00 | | 0 | | 0.00 | | 0 | | 1 | | | 0 | | 1 | |  |  |  |  |
| Q9NPA3 | Mid1-interacting protein 1 | MID1IP1 | 6.01 | 1 | | 20.2 | | 5.50 | | 0.16 | 1 | | 0.00 | | 0 | | 0.00 | | 0 | | 0.00 | | 0 | | 0.00 | | 0 | | 0.00 | | 0 | | 0.00 | | 0 | | 0.00 | | 0 | | 1 | | | 0 | | 1 | |  |  |  |  |
| A0A2R8YGD3 | Rap guanine nucleotide exchange factor 2 | RAPGEF2 | 0.77 | 1 | | 188.2 | | 6.68 | | 0.16 | 1 | | 0.00 | | 0 | | 0.00 | | 0 | | 0.00 | | 0 | | 0.00 | | 0 | | 0.00 | | 0 | | 0.00 | | 0 | | 0.00 | | 0 | | 1 | | | 0 | | 1 | |  |  |  |  |
| P51857 | Aldo-keto reductase family 1 member D1 | AKR1D1 | 2.76 | 1 | | 37.4 | | 7.50 | | 0.16 | 1 | | 0.00 | | 0 | | 0.00 | | 0 | | 0.00 | | 0 | | 0.00 | | 0 | | 0.00 | | 0 | | 0.00 | | 0 | | 0.00 | | 0 | | 1 | | | 0 | | 1 | |  |  |  |  |
| C9JWG6 | Schwannomin interacting protein 1, isoform CRA_c | SCHIP1 | 10.00 | 1 | | 29.2 | | 4.97 | | 0.16 | 1 | | 0.00 | | 0 | | 0.00 | | 0 | | 0.00 | | 0 | | 0.00 | | 0 | | 0.00 | | 0 | | 0.00 | | 0 | | 0.00 | | 0 | | 1 | | | 0 | | 1 | |  |  |  |  |
| Q96CS2 | HAUS augmin-like complex subunit 1 | HAUS1 | 3.96 | 1 | | 31.8 | | 5.53 | | 0.16 | 1 | | 0.00 | | 0 | | 0.00 | | 0 | | 0.00 | | 0 | | 0.00 | | 0 | | 0.00 | | 0 | | 0.00 | | 0 | | 0.00 | | 0 | | 1 | | | 0 | | 1 | |  |  |  |  |
| Q8IYT1 | Protein FAM71A | FAM71A | 1.35 | 1 | | 63.1 | | 9.64 | | 0.16 | 1 | | 0.00 | | 0 | | 0.00 | | 0 | | 0.00 | | 0 | | 0.00 | | 0 | | 0.00 | | 0 | | 0.00 | | 0 | | 0.00 | | 0 | | 1 | | | 0 | | 1 | |  |  |  |  |
| Q15404 | Ras suppressor protein 1 | RSU1 | 8.66 | 1 | | 31.5 | | 8.65 | | 0.15 | 1 | | 0.00 | | 0 | | 0.00 | | 0 | | 0.00 | | 0 | | 0.00 | | 0 | | 0.00 | | 0 | | 0.00 | | 0 | | 0.00 | | 0 | | 1 | | | 0 | | 1 | |  |  |  |  |
| A0A096LNH6 | Dedicator of cytokinesis protein 1 | DOCK1 | 0.37 | 1 | | 217.6 | | 7.71 | | 0.15 | 1 | | 0.00 | | 0 | | 0.00 | | 0 | | 0.00 | | 0 | | 0.00 | | 0 | | 0.00 | | 0 | | 0.00 | | 0 | | 0.00 | | 0 | | 1 | | | 0 | | 1 | |  |  |  |  |
| F2Z3N3 | Olfactomedin-like protein 2B | OLFML2B | 2.40 | 1 | | 84.1 | | 5.20 | | 0.15 | 1 | | 0.00 | | 0 | | 0.00 | | 0 | | 0.00 | | 0 | | 0.00 | | 0 | | 0.00 | | 0 | | 0.00 | | 0 | | 0.00 | | 0 | | 1 | | | 0 | | 1 | |  |  |  |  |
| Q5JY77 | G-protein coupled receptor-associated sorting protein 1 | GPRASP1 | 0.72 | 1 | | 156.8 | | 4.68 | | 0.15 | 1 | | 0.00 | | 0 | | 0.00 | | 0 | | 0.00 | | 0 | | 0.00 | | 0 | | 0.00 | | 0 | | 0.00 | | 0 | | 0.00 | | 0 | | 1 | | | 0 | | 1 | |  |  |  |  |
| A0A2R8Y7X1 | Chromodomain-helicase-DNA-binding protein 4 (Fragment) | CHD4 | 24.66 | 1 | | 8.4 | | 4.74 | | 0.15 | 1 | | 0.00 | | 0 | | 0.00 | | 0 | | 0.00 | | 0 | | 0.00 | | 0 | | 0.00 | | 0 | | 0.00 | | 0 | | 0.00 | | 0 | | 1 | | | 0 | | 1 | |  |  |  |  |
| Q53EU6 | Glycerol-3-phosphate acyltransferase 3 | GPAT3 | 2.07 | 1 | | 48.7 | | 8.87 | | 0.15 | 1 | | 0.00 | | 0 | | 0.00 | | 0 | | 0.00 | | 0 | | 0.00 | | 0 | | 0.00 | | 0 | | 0.00 | | 0 | | 0.00 | | 0 | | 1 | | | 0 | | 1 | |  |  |  |  |
| Q6DN72 | Fc receptor-like protein 6 | FCRL6 | 4.61 | 1 | | 47.7 | | 7.58 | | 0.15 | 1 | | 0.00 | | 0 | | 0.00 | | 0 | | 0.00 | | 0 | | 0.00 | | 0 | | 0.00 | | 0 | | 0.00 | | 0 | | 0.00 | | 0 | | 1 | | | 0 | | 1 | |  |  |  |  |
| H7BY59 | Beta-taxilin (Fragment) | TXLNB | 20.30 | 1 | | 15.3 | | 8.73 | | 0.15 | 1 | | 0.00 | | 0 | | 0.00 | | 0 | | 0.00 | | 0 | | 0.00 | | 0 | | 0.00 | | 0 | | 0.00 | | 0 | | 0.00 | | 0 | | 1 | | | 0 | | 1 | |  |  |  |  |
| P19801 | Amiloride-sensitive amine oxidase [copper-containing] | AOC1 | 6.66 | 1 | | 85.3 | | 7.09 | | 0.15 | 1 | | 0.00 | | 0 | | 0.00 | | 0 | | 0.00 | | 0 | | 0.00 | | 0 | | 0.00 | | 0 | | 0.00 | | 0 | | 0.00 | | 0 | | 1 | | | 0 | | 1 | |  |  |  |  |
| Q14258 | E3 ubiquitin/ISG15 ligase TRIM25 | TRIM25 | 2.70 | 1 | | 70.9 | | 8.09 | | 0.15 | 1 | | 0.00 | | 0 | | 0.00 | | 0 | | 0.00 | | 0 | | 0.00 | | 0 | | 0.00 | | 0 | | 0.00 | | 0 | | 0.00 | | 0 | | 1 | | | 0 | | 1 | |  |  |  |  |
| Q9BYG3 | MKI67 FHA domain-interacting nucleolar phosphoprotein | NIFK | 1.37 | 1 | | 34.2 | | 9.88 | | 0.15 | 1 | | 0.00 | | 0 | | 0.00 | | 0 | | 0.00 | | 0 | | 0.00 | | 0 | | 0.00 | | 0 | | 0.00 | | 0 | | 0.00 | | 0 | | 1 | | | 0 | | 1 | |  |  |  |  |
| Q86UU5 | Gametogenetin | GGN | 4.60 | 1 | | 66.7 | | 10.29 | | 0.15 | 1 | | 0.00 | | 0 | | 0.00 | | 0 | | 0.00 | | 0 | | 0.00 | | 0 | | 0.00 | | 0 | | 0.00 | | 0 | | 0.00 | | 0 | | 1 | | | 0 | | 1 | |  |  |  |  |
| Q13315 | Serine-protein kinase ATM | ATM | 0.29 | 1 | | 350.5 | | 6.81 | | 0.15 | 1 | | 0.00 | | 0 | | 0.00 | | 0 | | 0.00 | | 0 | | 0.00 | | 0 | | 0.00 | | 0 | | 0.00 | | 0 | | 0.00 | | 0 | | 1 | | | 0 | | 1 | |  |  |  |  |
| E9PQB0 | Nuclear factor-related to kappa-B-binding protein | NFRKB | 3.28 | 1 | | 13.7 | | 4.37 | | 0.15 | 1 | | 0.00 | | 0 | | 0.00 | | 0 | | 0.00 | | 0 | | 0.00 | | 0 | | 0.00 | | 0 | | 0.00 | | 0 | | 0.00 | | 0 | | 1 | | | 0 | | 1 | |  |  |  |  |
| H7C008 | Transmembrane protein 214 (Fragment) | TMEM214 | 8.17 | 1 | | 34.2 | | 8.76 | | 0.15 | 1 | | 0.00 | | 0 | | 0.00 | | 0 | | 0.00 | | 0 | | 0.00 | | 0 | | 0.00 | | 0 | | 0.00 | | 0 | | 0.00 | | 0 | | 1 | | | 0 | | 1 | |  |  |  |  |
| O00629 | Importin subunit alpha-3 | KPNA4 | 3.26 | 1 | | 57.9 | | 4.96 | | 0.15 | 1 | | 0.00 | | 0 | | 0.00 | | 0 | | 0.00 | | 0 | | 0.00 | | 0 | | 0.00 | | 0 | | 0.00 | | 0 | | 0.00 | | 0 | | 1 | | | 0 | | 1 | |  |  |  |  |
| O75175 | CCR4-NOT transcription complex subunit 3 | CNOT3 | 1.06 | 1 | | 81.8 | | 6.20 | | 0.15 | 1 | | 0.00 | | 0 | | 0.00 | | 0 | | 0.00 | | 0 | | 0.00 | | 0 | | 0.00 | | 0 | | 0.00 | | 0 | | 0.00 | | 0 | | 1 | | | 0 | | 1 | |  |  |  |  |
| Q8TE82 | SH3 domain and tetratricopeptide repeat-containing protein 1 | SH3TC1 | 0.67 | 1 | | 146.9 | | 6.25 | | 0.15 | 1 | | 0.00 | | 0 | | 0.00 | | 0 | | 0.00 | | 0 | | 0.00 | | 0 | | 0.00 | | 0 | | 0.00 | | 0 | | 0.00 | | 0 | | 1 | | | 0 | | 1 | |  |  |  |  |
| Q8TDV0 | G-protein coupled receptor 151 | GPR151 | 3.82 | 1 | | 46.6 | | 6.60 | | 0.15 | 1 | | 0.00 | | 0 | | 0.00 | | 0 | | 0.00 | | 0 | | 0.00 | | 0 | | 0.00 | | 0 | | 0.00 | | 0 | | 0.00 | | 0 | | 1 | | | 0 | | 1 | |  |  |  |  |
| P11388 | DNA topoisomerase 2-alpha | TOP2A | 0.85 | 1 | | 174.3 | | 8.72 | | 0.15 | 1 | | 0.00 | | 0 | | 0.00 | | 0 | | 0.00 | | 0 | | 0.00 | | 0 | | 0.00 | | 0 | | 0.00 | | 0 | | 0.00 | | 0 | | 1 | | | 0 | | 1 | |  |  |  |  |
| P43250 | G protein-coupled receptor kinase 6 | GRK6 | 2.95 | 1 | | 65.9 | | 8.00 | | 0.15 | 1 | | 0.00 | | 0 | | 0.00 | | 0 | | 0.00 | | 0 | | 0.00 | | 0 | | 0.00 | | 0 | | 0.00 | | 0 | | 0.00 | | 0 | | 1 | | | 0 | | 1 | |  |  |  |  |
| Q96DA0 | Zymogen granule protein 16 homolog B | ZG16B | 7.21 | 1 | | 22.7 | | 7.39 | | 0.15 | 1 | | 0.00 | | 0 | | 0.00 | | 0 | | 0.00 | | 0 | | 0.00 | | 0 | | 0.00 | | 0 | | 0.00 | | 0 | | 0.00 | | 0 | | 1 | | | 0 | | 1 | |  |  |  |  |
| B0QYC7 | Cdc42 effector protein 1 (Fragment) | CDC42EP1 | 11.67 | 1 | | 6.3 | | 10.78 | | 0.15 | 1 | | 0.00 | | 0 | | 0.00 | | 0 | | 0.00 | | 0 | | 0.00 | | 0 | | 0.00 | | 0 | | 0.00 | | 0 | | 0.00 | | 0 | | 1 | | | 0 | | 1 | |  |  |  |  |
| P53004 | Biliverdin reductase A | BLVRA | 8.45 | 1 | | 33.4 | | 6.44 | | 0.15 | 1 | | 0.00 | | 0 | | 0.00 | | 0 | | 0.00 | | 0 | | 0.00 | | 0 | | 0.00 | | 0 | | 0.00 | | 0 | | 0.00 | | 0 | | 1 | | | 0 | | 1 | |  |  |  |  |
| G3V292 | Serine/threonine-protein phosphatase 2A 56 kDa regulatory subunit gamma isoform | PPP2R5C | 6.45 | 1 | | 13.9 | | 9.92 | | 0.15 | 1 | | 0.00 | | 0 | | 0.00 | | 0 | | 0.00 | | 0 | | 0.00 | | 0 | | 0.00 | | 0 | | 0.00 | | 0 | | 0.00 | | 0 | | 1 | | | 0 | | 1 | |  |  |  |  |
| Q8IZW8 | Tensin-4 | TNS4 | 1.12 | 1 | | 76.7 | | 7.34 | | 0.15 | 1 | | 0.00 | | 0 | | 0.00 | | 0 | | 0.00 | | 0 | | 0.00 | | 0 | | 0.00 | | 0 | | 0.00 | | 0 | | 0.00 | | 0 | | 1 | | | 0 | | 1 | |  |  |  |  |
| E5RHT7 | MAPK/MAK/MRK overlapping kinase | MOK | 19.20 | 1 | | 13.4 | | 7.27 | | 0.15 | 1 | | 0.00 | | 0 | | 0.00 | | 0 | | 0.00 | | 0 | | 0.00 | | 0 | | 0.00 | | 0 | | 0.00 | | 0 | | 0.00 | | 0 | | 1 | | | 0 | | 1 | |  |  |  |  |
| F5H619 | HEAT repeat-containing protein 5A | HEATR5A | 0.24 | 1 | | 222.6 | | 6.55 | | 0.15 | 1 | | 0.00 | | 0 | | 0.00 | | 0 | | 0.00 | | 0 | | 0.00 | | 0 | | 0.00 | | 0 | | 0.00 | | 0 | | 0.00 | | 0 | | 1 | | | 0 | | 1 | |  |  |  |  |
| Q32MH5 | Protein FAM214A | FAM214A | 0.74 | 1 | | 121.6 | | 7.90 | | 0.15 | 1 | | 0.00 | | 0 | | 0.00 | | 0 | | 0.00 | | 0 | | 0.00 | | 0 | | 0.00 | | 0 | | 0.00 | | 0 | | 0.00 | | 0 | | 1 | | | 0 | | 1 | |  |  |  |  |
| Q8N5Z0 | Kynurenine/alpha-aminoadipate aminotransferase, mitochondrial | AADAT | 2.12 | 1 | | 47.3 | | 6.96 | | 0.15 | 1 | | 0.00 | | 0 | | 0.00 | | 0 | | 0.00 | | 0 | | 0.00 | | 0 | | 0.00 | | 0 | | 0.00 | | 0 | | 0.00 | | 0 | | 1 | | | 0 | | 1 | |  |  |  |  |
| E7ERK9 | Translation initiation factor eIF-2B subunit delta | EIF2B4 | 1.84 | 1 | | 59.7 | | 9.42 | | 0.15 | 1 | | 0.00 | | 0 | | 0.00 | | 0 | | 0.00 | | 0 | | 0.00 | | 0 | | 0.00 | | 0 | | 0.00 | | 0 | | 0.00 | | 0 | | 1 | | | 0 | | 1 | |  |  |  |  |
| Q9HAQ2 | Kinesin-like protein KIF9 | KIF9 | 0.89 | 1 | | 89.9 | | 7.17 | | 0.15 | 1 | | 0.00 | | 0 | | 0.00 | | 0 | | 0.00 | | 0 | | 0.00 | | 0 | | 0.00 | | 0 | | 0.00 | | 0 | | 0.00 | | 0 | | 1 | | | 0 | | 1 | |  |  |  |  |
| Q96PV4 | Paraneoplastic antigen-like protein 5 | PNMA5 | 3.35 | 1 | | 49.9 | | 6.73 | | 0.15 | 1 | | 0.00 | | 0 | | 0.00 | | 0 | | 0.00 | | 0 | | 0.00 | | 0 | | 0.00 | | 0 | | 0.00 | | 0 | | 0.00 | | 0 | | 1 | | | 0 | | 1 | |  |  |  |  |
| Q6ZVF9 | G protein-regulated inducer of neurite outgrowth 3 | GPRIN3 | 0.77 | 1 | | 82.4 | | 7.56 | | 0.15 | 1 | | 0.00 | | 0 | | 0.00 | | 0 | | 0.00 | | 0 | | 0.00 | | 0 | | 0.00 | | 0 | | 0.00 | | 0 | | 0.00 | | 0 | | 1 | | | 0 | | 1 | |  |  |  |  |
| Q96A98 | Tuberoinfundibular peptide of 39 residues | PTH2 | 16.00 | 1 | | 11.2 | | 11.82 | | 0.28 | 1 | | 0.00 | | 0 | | 0.00 | | 0 | | 0.00 | | 0 | | 0.00 | | 0 | | 0.00 | | 0 | | 0.00 | | 0 | | 0.00 | | 0 | | 1 | | | 0 | | 1 | |  |  |  |  |
| P49768 | Presenilin-1 | PSEN1 | 4.93 | 1 | | 52.6 | | 5.31 | | 0.28 | 1 | | 0.00 | | 0 | | 0.00 | | 0 | | 0.00 | | 0 | | 0.00 | | 0 | | 0.00 | | 0 | | 0.00 | | 0 | | 0.00 | | 0 | | 1 | | | 0 | | 1 | |  |  |  |  |
| Q9HCH0 | Nck-associated protein 5-like | NCKAP5L | 3.98 | 1 | | 138.9 | | 8.13 | | 0.28 | 1 | | 0.00 | | 0 | | 0.00 | | 0 | | 0.00 | | 0 | | 0.00 | | 0 | | 0.00 | | 0 | | 0.00 | | 0 | | 0.00 | | 0 | | 1 | | | 0 | | 1 | |  |  |  |  |
| Q8N434 | Putative transporter SVOPL | SVOPL | 2.24 | 1 | | 54.0 | | 8.81 | | 0.28 | 1 | | 0.00 | | 0 | | 0.00 | | 0 | | 0.00 | | 0 | | 0.00 | | 0 | | 0.00 | | 0 | | 0.00 | | 0 | | 0.00 | | 0 | | 1 | | | 0 | | 1 | |  |  |  |  |
| Q86VR2 | Reticulophagy regulator 3 | RETREG3 | 4.51 | 1 | | 51.4 | | 4.97 | | 0.28 | 1 | | 0.00 | | 0 | | 0.00 | | 0 | | 0.00 | | 0 | | 0.00 | | 0 | | 0.00 | | 0 | | 0.00 | | 0 | | 0.00 | | 0 | | 1 | | | 0 | | 1 | |  |  |  |  |
| A0A087WUC4 | Alcohol dehydrogenase 1C (Fragment) | ADH1C | 8.82 | 1 | | 17.5 | | 8.06 | | 0.28 | 1 | | 0.00 | | 0 | | 0.00 | | 0 | | 0.00 | | 0 | | 0.00 | | 0 | | 0.00 | | 0 | | 0.00 | | 0 | | 0.00 | | 0 | | 1 | | | 0 | | 1 | |  |  |  |  |
| Q9ULP9 | TBC1 domain family member 24 | TBC1D24 | 8.23 | 1 | | 62.9 | | 7.36 | | 0.28 | 1 | | 0.00 | | 0 | | 0.00 | | 0 | | 0.00 | | 0 | | 0.00 | | 0 | | 0.00 | | 0 | | 0.00 | | 0 | | 0.00 | | 0 | | 1 | | | 0 | | 1 | |  |  |  |  |
| O75529 | TAF5-like RNA polymerase II p300/CBP-associated factor-associated factor 65 kDa subunit 5L | TAF5L | 2.04 | 1 | | 66.1 | | 5.88 | | 0.28 | 1 | | 0.00 | | 0 | | 0.00 | | 0 | | 0.00 | | 0 | | 0.00 | | 0 | | 0.00 | | 0 | | 0.00 | | 0 | | 0.00 | | 0 | | 1 | | | 0 | | 1 | |  |  |  |  |
| H7BXS7 | Dynein heavy chain 14, axonemal (Fragment) | DNAH14 | 2.23 | 1 | | 169.1 | | 7.68 | | 0.28 | 1 | | 0.00 | | 0 | | 0.00 | | 0 | | 0.00 | | 0 | | 0.00 | | 0 | | 0.00 | | 0 | | 0.00 | | 0 | | 0.00 | | 0 | | 1 | | | 0 | | 1 | |  |  |  |  |
| Q8NFW1 | Collagen alpha-1(XXII) chain | COL22A1 | 2.46 | 1 | | 161.0 | | 7.23 | | 0.28 | 1 | | 0.00 | | 0 | | 0.00 | | 0 | | 0.00 | | 0 | | 0.00 | | 0 | | 0.00 | | 0 | | 0.00 | | 0 | | 0.00 | | 0 | | 1 | | | 0 | | 1 | |  |  |  |  |
| P13056 | Nuclear receptor subfamily 2 group C member 1 | NR2C1 | 8.62 | 1 | | 67.3 | | 6.32 | | 0.28 | 1 | | 0.00 | | 0 | | 0.00 | | 0 | | 0.00 | | 0 | | 0.00 | | 0 | | 0.00 | | 0 | | 0.00 | | 0 | | 0.00 | | 0 | | 1 | | | 0 | | 1 | |  |  |  |  |
| O14830 | Serine/threonine-protein phosphatase with EF-hands 2 | PPEF2 | 2.39 | 1 | | 86.5 | | 7.14 | | 0.28 | 1 | | 0.00 | | 0 | | 0.00 | | 0 | | 0.00 | | 0 | | 0.00 | | 0 | | 0.00 | | 0 | | 0.00 | | 0 | | 0.00 | | 0 | | 1 | | | 0 | | 1 | |  |  |  |  |
| A0A494C1K3 | General transcription factor II-I | GTF2I | 1.64 | 1 | | 131.0 | | 8.69 | | 0.28 | 1 | | 0.00 | | 0 | | 0.00 | | 0 | | 0.00 | | 0 | | 0.00 | | 0 | | 0.00 | | 0 | | 0.00 | | 0 | | 0.00 | | 0 | | 1 | | | 0 | | 1 | |  |  |  |  |
| Q76NI1 | Kinase non-catalytic C-lobe domain-containing protein 1 | KNDC1 | 0.57 | 1 | | 191.3 | | 6.16 | | 0.28 | 1 | | 0.00 | | 0 | | 0.00 | | 0 | | 0.00 | | 0 | | 0.00 | | 0 | | 0.00 | | 0 | | 0.00 | | 0 | | 0.00 | | 0 | | 1 | | | 0 | | 1 | |  |  |  |  |
| O95633 | Follistatin-related protein 3 | FSTL3 | 6.08 | 1 | | 27.6 | | 6.77 | | 0.28 | 1 | | 0.00 | | 0 | | 0.00 | | 0 | | 0.00 | | 0 | | 0.00 | | 0 | | 0.00 | | 0 | | 0.00 | | 0 | | 0.00 | | 0 | | 1 | | | 0 | | 1 | |  |  |  |  |
| Q5W197 | Solute carrier family 17 member 9 (Fragment) | SLC17A9 | 4.61 | 1 | | 16.7 | | 8.63 | | 0.28 | 1 | | 0.00 | | 0 | | 0.00 | | 0 | | 0.00 | | 0 | | 0.00 | | 0 | | 0.00 | | 0 | | 0.00 | | 0 | | 0.00 | | 0 | | 1 | | | 0 | | 1 | |  |  |  |  |
| Q99502 | Eyes absent homolog 1 | EYA1 | 4.05 | 1 | | 64.6 | | 6.21 | | 0.28 | 1 | | 0.00 | | 0 | | 0.00 | | 0 | | 0.00 | | 0 | | 0.00 | | 0 | | 0.00 | | 0 | | 0.00 | | 0 | | 0.00 | | 0 | | 1 | | | 0 | | 1 | |  |  |  |  |
| H0YL89 | BTB/POZ domain-containing protein 1 (Fragment) | BTBD1 | 43.24 | 1 | | 4.3 | | 7.44 | | 0.28 | 1 | | 0.00 | | 0 | | 0.00 | | 0 | | 0.00 | | 0 | | 0.00 | | 0 | | 0.00 | | 0 | | 0.00 | | 0 | | 0.00 | | 0 | | 1 | | | 0 | | 1 | |  |  |  |  |
| Q6PJT7 | Zinc finger CCCH domain-containing protein 14 | ZC3H14 | 6.79 | 1 | | 82.8 | | 7.31 | | 0.28 | 1 | | 0.00 | | 0 | | 0.00 | | 0 | | 0.00 | | 0 | | 0.00 | | 0 | | 0.00 | | 0 | | 0.00 | | 0 | | 0.00 | | 0 | | 1 | | | 0 | | 1 | |  |  |  |  |
| P41743 | Protein kinase C iota type | PRKCI | 2.01 | 1 | | 68.2 | | 5.85 | | 0.28 | 1 | | 0.00 | | 0 | | 0.00 | | 0 | | 0.00 | | 0 | | 0.00 | | 0 | | 0.00 | | 0 | | 0.00 | | 0 | | 0.00 | | 0 | | 1 | | | 0 | | 1 | |  |  |  |  |
| Q8IYB1 | Protein MB21D2 | MB21D2 | 1.43 | 1 | | 55.8 | | 7.03 | | 0.28 | 1 | | 0.00 | | 0 | | 0.00 | | 0 | | 0.00 | | 0 | | 0.00 | | 0 | | 0.00 | | 0 | | 0.00 | | 0 | | 0.00 | | 0 | | 1 | | | 0 | | 1 | |  |  |  |  |
| H0YCE2 | Acidic leucine-rich nuclear phosphoprotein 32 family member E (Fragment) | ANP32E | 24.00 | 1 | | 11.7 | | 3.68 | | 0.28 | 1 | | 0.00 | | 0 | | 0.00 | | 0 | | 0.00 | | 0 | | 0.00 | | 0 | | 0.00 | | 0 | | 0.00 | | 0 | | 0.00 | | 0 | | 1 | | | 0 | | 1 | |  |  |  |  |
| Q9H4D5 | Nuclear RNA export factor 3 | NXF3 | 1.51 | 1 | | 60.1 | | 6.76 | | 0.28 | 1 | | 0.00 | | 0 | | 0.00 | | 0 | | 0.00 | | 0 | | 0.00 | | 0 | | 0.00 | | 0 | | 0.00 | | 0 | | 0.00 | | 0 | | 1 | | | 0 | | 1 | |  |  |  |  |
| Q9UN76 | Sodium- and chloride-dependent neutral and basic amino acid transporter B(0+) | SLC6A14 | 2.96 | 1 | | 72.1 | | 8.19 | | 0.28 | 1 | | 0.00 | | 0 | | 0.00 | | 0 | | 0.00 | | 0 | | 0.00 | | 0 | | 0.00 | | 0 | | 0.00 | | 0 | | 0.00 | | 0 | | 1 | | | 0 | | 1 | |  |  |  |  |
| F8VXR6 | 60S ribosomal protein L18 | RPL18 | 51.19 | 1 | | 9.5 | | 6.92 | | 0.28 | 1 | | 0.00 | | 0 | | 0.00 | | 0 | | 0.00 | | 0 | | 0.00 | | 0 | | 0.00 | | 0 | | 0.00 | | 0 | | 0.00 | | 0 | | 1 | | | 0 | | 1 | |  |  |  |  |
| Q9BSA4 | Protein tweety homolog 2 | TTYH2 | 3.75 | 1 | | 58.7 | | 5.83 | | 0.28 | 1 | | 0.00 | | 0 | | 0.00 | | 0 | | 0.00 | | 0 | | 0.00 | | 0 | | 0.00 | | 0 | | 0.00 | | 0 | | 0.00 | | 0 | | 1 | | | 0 | | 1 | |  |  |  |  |
| A0A1X7SBZ8 | Gamma-aminobutyric acid receptor subunit gamma-2 | GABRG2 | 3.16 | 1 | | 58.3 | | 8.53 | | 0.28 | 1 | | 0.00 | | 0 | | 0.00 | | 0 | | 0.00 | | 0 | | 0.00 | | 0 | | 0.00 | | 0 | | 0.00 | | 0 | | 0.00 | | 0 | | 1 | | | 0 | | 1 | |  |  |  |  |
| Q6NUI6 | Chondroadherin-like protein | CHADL | 1.71 | 1 | | 82.3 | | 8.84 | | 0.28 | 1 | | 0.00 | | 0 | | 0.00 | | 0 | | 0.00 | | 0 | | 0.00 | | 0 | | 0.00 | | 0 | | 0.00 | | 0 | | 0.00 | | 0 | | 1 | | | 0 | | 1 | |  |  |  |  |
| K7EME9 | Chromatin assembly factor 1 subunit A (Fragment) | CHAF1A | 68.09 | 1 | | 5.2 | | 3.70 | | 0.28 | 1 | | 0.00 | | 0 | | 0.00 | | 0 | | 0.00 | | 0 | | 0.00 | | 0 | | 0.00 | | 0 | | 0.00 | | 0 | | 0.00 | | 0 | | 1 | | | 0 | | 1 | |  |  |  |  |
| Q7L576 | Cytoplasmic FMR1-interacting protein 1 | CYFIP1 | 1.76 | 1 | | 145.1 | | 6.90 | | 0.28 | 1 | | 0.00 | | 0 | | 0.00 | | 0 | | 0.00 | | 0 | | 0.00 | | 0 | | 0.00 | | 0 | | 0.00 | | 0 | | 0.00 | | 0 | | 1 | | | 0 | | 1 | |  |  |  |  |
| P18084 | Integrin beta-5 | ITGB5 | 7.63 | 1 | | 88.0 | | 6.06 | | 0.28 | 1 | | 0.00 | | 0 | | 0.00 | | 0 | | 0.00 | | 0 | | 0.00 | | 0 | | 0.00 | | 0 | | 0.00 | | 0 | | 0.00 | | 0 | | 1 | | | 0 | | 1 | |  |  |  |  |
| Q9NUQ7 | Ufm1-specific protease 2 | UFSP2 | 4.26 | 1 | | 53.2 | | 7.01 | | 0.28 | 1 | | 0.00 | | 0 | | 0.00 | | 0 | | 0.00 | | 0 | | 0.00 | | 0 | | 0.00 | | 0 | | 0.00 | | 0 | | 0.00 | | 0 | | 1 | | | 0 | | 1 | |  |  |  |  |
| O60603 | Toll-like receptor 2 | TLR2 | 1.02 | 1 | | 89.8 | | 6.61 | | 0.28 | 1 | | 0.00 | | 0 | | 0.00 | | 0 | | 0.00 | | 0 | | 0.00 | | 0 | | 0.00 | | 0 | | 0.00 | | 0 | | 0.00 | | 0 | | 1 | | | 0 | | 1 | |  |  |  |  |
| A0A494C1J1 | Cytospin-A | SPECC1L | 0.62 | 1 | | 126.2 | | 5.57 | | 0.28 | 1 | | 0.00 | | 0 | | 0.00 | | 0 | | 0.00 | | 0 | | 0.00 | | 0 | | 0.00 | | 0 | | 0.00 | | 0 | | 0.00 | | 0 | | 1 | | | 0 | | 1 | |  |  |  |  |
| Q9GZL7 | Ribosome biogenesis protein WDR12 | WDR12 | 9.22 | 1 | | 47.7 | | 5.90 | | 0.28 | 1 | | 0.00 | | 0 | | 0.00 | | 0 | | 0.00 | | 0 | | 0.00 | | 0 | | 0.00 | | 0 | | 0.00 | | 0 | | 0.00 | | 0 | | 1 | | | 0 | | 1 | |  |  |  |  |
| F8WET1 | Vasopressin V2 receptor | AVPR2 | 72.92 | 1 | | 4.5 | | 9.32 | | 0.28 | 1 | | 0.00 | | 0 | | 0.00 | | 0 | | 0.00 | | 0 | | 0.00 | | 0 | | 0.00 | | 0 | | 0.00 | | 0 | | 0.00 | | 0 | | 1 | | | 0 | | 1 | |  |  |  |  |
| Q8NH08 | Olfactory receptor 10AC1 | OR10AC1 | 2.46 | 1 | | 35.1 | | 9.00 | | 0.28 | 1 | | 0.00 | | 0 | | 0.00 | | 0 | | 0.00 | | 0 | | 0.00 | | 0 | | 0.00 | | 0 | | 0.00 | | 0 | | 0.00 | | 0 | | 1 | | | 0 | | 1 | |  |  |  |  |
| Q6ZWB5 | C3orf57 protein | SPTSSB | 16.52 | 1 | | 24.9 | | 11.36 | | 0.28 | 1 | | 0.00 | | 0 | | 0.00 | | 0 | | 0.00 | | 0 | | 0.00 | | 0 | | 0.00 | | 0 | | 0.00 | | 0 | | 0.00 | | 0 | | 1 | | | 0 | | 1 | |  |  |  |  |
| A0A1W2PNR4 | Lactosylceramide alpha-2,3-sialyltransferase (Fragment) | ST3GAL5 | 23.29 | 1 | | 8.6 | | 8.95 | | 0.28 | 1 | | 0.00 | | 0 | | 0.00 | | 0 | | 0.00 | | 0 | | 0.00 | | 0 | | 0.00 | | 0 | | 0.00 | | 0 | | 0.00 | | 0 | | 1 | | | 0 | | 1 | |  |  |  |  |
| Q9ULJ1 | Protein BCAP | ODF2L | 1.42 | 1 | | 73.7 | | 6.48 | | 0.28 | 1 | | 0.00 | | 0 | | 0.00 | | 0 | | 0.00 | | 0 | | 0.00 | | 0 | | 0.00 | | 0 | | 0.00 | | 0 | | 0.00 | | 0 | | 1 | | | 0 | | 1 | |  |  |  |  |
| B4DR61 | Protein transport protein Sec61 subunit alpha isoform 1 | SEC61A1 | 10.79 | 1 | | 52.9 | | 8.24 | | 0.28 | 1 | | 0.00 | | 0 | | 0.00 | | 0 | | 0.00 | | 0 | | 0.00 | | 0 | | 0.00 | | 0 | | 0.00 | | 0 | | 0.00 | | 0 | | 1 | | | 0 | | 1 | |  |  |  |  |
| A0A3F2YP85 | Myeloid differentiation primary response protein MyD88 | MYD88 | 25.00 | 1 | | 22.1 | | 5.14 | | 0.28 | 1 | | 0.00 | | 0 | | 0.00 | | 0 | | 0.00 | | 0 | | 0.00 | | 0 | | 0.00 | | 0 | | 0.00 | | 0 | | 0.00 | | 0 | | 1 | | | 0 | | 1 | |  |  |  |  |
| Q8TF21 | Ankyrin repeat domain-containing protein 24 | ANKRD24 | 2.79 | 1 | | 124.1 | | 5.01 | | 0.28 | 1 | | 0.00 | | 0 | | 0.00 | | 0 | | 0.00 | | 0 | | 0.00 | | 0 | | 0.00 | | 0 | | 0.00 | | 0 | | 0.00 | | 0 | | 1 | | | 0 | | 1 | |  |  |  |  |
| Q9UGU0 | Transcription factor 20 | TCF20 | 1.43 | 1 | | 211.6 | | 9.04 | | 0.28 | 1 | | 0.00 | | 0 | | 0.00 | | 0 | | 0.00 | | 0 | | 0.00 | | 0 | | 0.00 | | 0 | | 0.00 | | 0 | | 0.00 | | 0 | | 1 | | | 0 | | 1 | |  |  |  |  |
| Q96RJ0 | Trace amine-associated receptor 1 | TAAR1 | 8.55 | 1 | | 39.1 | | 8.70 | | 0.28 | 1 | | 0.00 | | 0 | | 0.00 | | 0 | | 0.00 | | 0 | | 0.00 | | 0 | | 0.00 | | 0 | | 0.00 | | 0 | | 0.00 | | 0 | | 1 | | | 0 | | 1 | |  |  |  |  |
| Q8IZC6 | Collagen alpha-1(XXVII) chain | COL27A1 | 3.23 | 1 | | 186.8 | | 9.82 | | 0.28 | 1 | | 0.00 | | 0 | | 0.00 | | 0 | | 0.00 | | 0 | | 0.00 | | 0 | | 0.00 | | 0 | | 0.00 | | 0 | | 0.00 | | 0 | | 1 | | | 0 | | 1 | |  |  |  |  |
| P01350 | Gastrin | GAST | 17.82 | 1 | | 11.4 | | 5.19 | | 0.28 | 1 | | 0.00 | | 0 | | 0.00 | | 0 | | 0.00 | | 0 | | 0.00 | | 0 | | 0.00 | | 0 | | 0.00 | | 0 | | 0.00 | | 0 | | 1 | | | 0 | | 1 | |  |  |  |  |
| G3V1W8 | Serine/threonine-protein kinase receptor | ACVRL1 | 4.26 | 1 | | 57.5 | | 7.61 | | 0.28 | 1 | | 0.00 | | 0 | | 0.00 | | 0 | | 0.00 | | 0 | | 0.00 | | 0 | | 0.00 | | 0 | | 0.00 | | 0 | | 0.00 | | 0 | | 1 | | | 0 | | 1 | |  |  |  |  |
| P55089 | Urocortin | UCN | 6.45 | 1 | | 13.5 | | 11.71 | | 0.28 | 1 | | 0.00 | | 0 | | 0.00 | | 0 | | 0.00 | | 0 | | 0.00 | | 0 | | 0.00 | | 0 | | 0.00 | | 0 | | 0.00 | | 0 | | 1 | | | 0 | | 1 | |  |  |  |  |
| Q9H7F4 | Transmembrane protein 185B | TMEM185B | 7.71 | 1 | | 40.6 | | 7.75 | | 0.28 | 1 | | 0.00 | | 0 | | 0.00 | | 0 | | 0.00 | | 0 | | 0.00 | | 0 | | 0.00 | | 0 | | 0.00 | | 0 | | 0.00 | | 0 | | 1 | | | 0 | | 1 | |  |  |  |  |
| Q14739 | Delta(14)-sterol reductase LBR | LBR | 3.09 | 1 | | 70.7 | | 9.36 | | 0.28 | 1 | | 0.00 | | 0 | | 0.00 | | 0 | | 0.00 | | 0 | | 0.00 | | 0 | | 0.00 | | 0 | | 0.00 | | 0 | | 0.00 | | 0 | | 1 | | | 0 | | 1 | |  |  |  |  |
| K7EJ65 | Protein Hook homolog 2 (Fragment) | HOOK2 | 3.83 | 1 | | 23.3 | | 4.97 | | 0.28 | 1 | | 0.00 | | 0 | | 0.00 | | 0 | | 0.00 | | 0 | | 0.00 | | 0 | | 0.00 | | 0 | | 0.00 | | 0 | | 0.00 | | 0 | | 1 | | | 0 | | 1 | |  |  |  |  |
| Q9Y4A5 | Transformation/transcription domain-associated protein | TRRAP | 0.73 | 1 | | 437.3 | | 8.19 | | 0.28 | 1 | | 0.00 | | 0 | | 0.00 | | 0 | | 0.00 | | 0 | | 0.00 | | 0 | | 0.00 | | 0 | | 0.00 | | 0 | | 0.00 | | 0 | | 1 | | | 0 | | 1 | |  |  |  |  |
| Q6UY11 | Protein delta homolog 2 | DLK2 | 8.88 | 1 | | 40.5 | | 6.54 | | 0.28 | 1 | | 0.00 | | 0 | | 0.00 | | 0 | | 0.00 | | 0 | | 0.00 | | 0 | | 0.00 | | 0 | | 0.00 | | 0 | | 0.00 | | 0 | | 1 | | | 0 | | 1 | |  |  |  |  |
| Q6P4D5 | Protein FAM122C | FAM122C | 13.33 | 1 | | 22.5 | | 9.88 | | 0.28 | 1 | | 0.00 | | 0 | | 0.00 | | 0 | | 0.00 | | 0 | | 0.00 | | 0 | | 0.00 | | 0 | | 0.00 | | 0 | | 0.00 | | 0 | | 1 | | | 0 | | 1 | |  |  |  |  |
| Q8ND56 | Protein LSM14 homolog A | LSM14A | 10.37 | 1 | | 50.5 | | 9.52 | | 0.28 | 1 | | 0.00 | | 0 | | 0.00 | | 0 | | 0.00 | | 0 | | 0.00 | | 0 | | 0.00 | | 0 | | 0.00 | | 0 | | 0.00 | | 0 | | 1 | | | 0 | | 1 | |  |  |  |  |
| J3KNE8 | Cyclin-dependent kinase-like 2 | CDKL2 | 7.54 | 1 | | 64.8 | | 8.72 | | 0.28 | 1 | | 0.00 | | 0 | | 0.00 | | 0 | | 0.00 | | 0 | | 0.00 | | 0 | | 0.00 | | 0 | | 0.00 | | 0 | | 0.00 | | 0 | | 1 | | | 0 | | 1 | |  |  |  |  |
| P51805 | Plexin-A3 | PLXNA3 | 1.60 | 1 | | 207.6 | | 7.31 | | 0.28 | 1 | | 0.00 | | 0 | | 0.00 | | 0 | | 0.00 | | 0 | | 0.00 | | 0 | | 0.00 | | 0 | | 0.00 | | 0 | | 0.00 | | 0 | | 1 | | | 0 | | 1 | |  |  |  |  |
| H3BP20 | Beta-hexosaminidase | HEXA | 3.52 | 1 | | 62.0 | | 5.21 | | 0.13 | 1 | | 0.00 | | 0 | | 0.00 | | 0 | | 0.00 | | 0 | | 0.00 | | 0 | | 0.00 | | 0 | | 0.00 | | 0 | | 0.00 | | 0 | | 1 | | | 0 | | 1 | |  |  |  |  |
| Q7Z2K6 | Endoplasmic reticulum metallopeptidase 1 | ERMP1 | 2.43 | 1 | | 100.2 | | 7.52 | | 0.13 | 1 | | 0.00 | | 0 | | 0.00 | | 0 | | 0.00 | | 0 | | 0.00 | | 0 | | 0.00 | | 0 | | 0.00 | | 0 | | 0.00 | | 0 | | 1 | | | 0 | | 1 | |  |  |  |  |
| A0A087X1Z3 | Proteasome activator complex subunit 2 | PSME2 | 8.66 | 1 | | 29.1 | | 6.71 | | 0.13 | 1 | | 0.00 | | 0 | | 0.00 | | 0 | | 0.00 | | 0 | | 0.00 | | 0 | | 0.00 | | 0 | | 0.00 | | 0 | | 0.00 | | 0 | | 1 | | | 0 | | 1 | |  |  |  |  |
| Q12805 | EGF-containing fibulin-like extracellular matrix protein 1 | EFEMP1 | 4.67 | 1 | | 54.6 | | 5.07 | | 0.13 | 1 | | 0.00 | | 0 | | 0.00 | | 0 | | 0.00 | | 0 | | 0.00 | | 0 | | 0.00 | | 0 | | 0.00 | | 0 | | 0.00 | | 0 | | 1 | | | 0 | | 1 | |  |  |  |  |
| F5H1U9 | Multiple PDZ domain protein | MPDZ | 0.86 | 1 | | 223.0 | | 5.06 | | 0.13 | 1 | | 0.00 | | 0 | | 0.00 | | 0 | | 0.00 | | 0 | | 0.00 | | 0 | | 0.00 | | 0 | | 0.00 | | 0 | | 0.00 | | 0 | | 1 | | | 0 | | 1 | |  |  |  |  |
| Q9Y2G1 | Myelin regulatory factor | MYRF | 2.26 | 1 | | 124.3 | | 7.44 | | 0.13 | 1 | | 0.00 | | 0 | | 0.00 | | 0 | | 0.00 | | 0 | | 0.00 | | 0 | | 0.00 | | 0 | | 0.00 | | 0 | | 0.00 | | 0 | | 1 | | | 0 | | 1 | |  |  |  |  |
| A0A5K1VW80 | Aprataxin | APTX | 35.00 | 1 | | 6.9 | | 9.99 | | 0.13 | 1 | | 0.00 | | 0 | | 0.00 | | 0 | | 0.00 | | 0 | | 0.00 | | 0 | | 0.00 | | 0 | | 0.00 | | 0 | | 0.00 | | 0 | | 1 | | | 0 | | 1 | |  |  |  |  |
| Q9Y484 | WD repeat domain phosphoinositide-interacting protein 4 | WDR45 | 4.72 | 1 | | 39.8 | | 7.14 | | 0.13 | 1 | | 0.00 | | 0 | | 0.00 | | 0 | | 0.00 | | 0 | | 0.00 | | 0 | | 0.00 | | 0 | | 0.00 | | 0 | | 0.00 | | 0 | | 1 | | | 0 | | 1 | |  |  |  |  |
| O14813 | Paired mesoderm homeobox protein 2A | PHOX2A | 8.45 | 1 | | 29.6 | | 8.76 | | 0.13 | 1 | | 0.00 | | 0 | | 0.00 | | 0 | | 0.00 | | 0 | | 0.00 | | 0 | | 0.00 | | 0 | | 0.00 | | 0 | | 0.00 | | 0 | | 1 | | | 0 | | 1 | |  |  |  |  |
| K7EJP9 | Probable phospholipid-transporting ATPase IIB (Fragment) | ATP9B | 13.57 | 1 | | 15.9 | | 10.05 | | 0.13 | 1 | | 0.00 | | 0 | | 0.00 | | 0 | | 0.00 | | 0 | | 0.00 | | 0 | | 0.00 | | 0 | | 0.00 | | 0 | | 0.00 | | 0 | | 1 | | | 0 | | 1 | |  |  |  |  |
| P42785 | Lysosomal Pro-X carboxypeptidase | PRCP | 3.83 | 1 | | 55.8 | | 7.21 | | 0.13 | 1 | | 0.00 | | 0 | | 0.00 | | 0 | | 0.00 | | 0 | | 0.00 | | 0 | | 0.00 | | 0 | | 0.00 | | 0 | | 0.00 | | 0 | | 1 | | | 0 | | 1 | |  |  |  |  |
| Q99856 | AT-rich interactive domain-containing protein 3A | ARID3A | 2.19 | 1 | | 62.9 | | 4.91 | | 0.13 | 1 | | 0.00 | | 0 | | 0.00 | | 0 | | 0.00 | | 0 | | 0.00 | | 0 | | 0.00 | | 0 | | 0.00 | | 0 | | 0.00 | | 0 | | 1 | | | 0 | | 1 | |  |  |  |  |
| O75165 | DnaJ homolog subfamily C member 13 | DNAJC13 | 2.27 | 1 | | 254.3 | | 6.74 | | 0.13 | 1 | | 0.00 | | 0 | | 0.00 | | 0 | | 0.00 | | 0 | | 0.00 | | 0 | | 0.00 | | 0 | | 0.00 | | 0 | | 0.00 | | 0 | | 1 | | | 0 | | 1 | |  |  |  |  |
| Q9H8X3 | Putative uncharacterized protein LINC00574 | LINC00574 | 32.03 | 1 | | 13.6 | | 7.94 | | 0.13 | 1 | | 0.00 | | 0 | | 0.00 | | 0 | | 0.00 | | 0 | | 0.00 | | 0 | | 0.00 | | 0 | | 0.00 | | 0 | | 0.00 | | 0 | | 1 | | | 0 | | 1 | |  |  |  |  |
| P07738 | Bisphosphoglycerate mutase | BPGM | 8.11 | 1 | | 30.0 | | 6.54 | | 0.13 | 1 | | 0.00 | | 0 | | 0.00 | | 0 | | 0.00 | | 0 | | 0.00 | | 0 | | 0.00 | | 0 | | 0.00 | | 0 | | 0.00 | | 0 | | 1 | | | 0 | | 1 | |  |  |  |  |
| P22732 | Solute carrier family 2, facilitated glucose transporter member 5 | SLC2A5 | 1.80 | 1 | | 54.9 | | 6.04 | | 0.13 | 1 | | 0.00 | | 0 | | 0.00 | | 0 | | 0.00 | | 0 | | 0.00 | | 0 | | 0.00 | | 0 | | 0.00 | | 0 | | 0.00 | | 0 | | 1 | | | 0 | | 1 | |  |  |  |  |
| Q9ULZ1 | Apelin | APLN | 46.75 | 1 | | 8.6 | | 11.82 | | 0.13 | 1 | | 0.00 | | 0 | | 0.00 | | 0 | | 0.00 | | 0 | | 0.00 | | 0 | | 0.00 | | 0 | | 0.00 | | 0 | | 0.00 | | 0 | | 1 | | | 0 | | 1 | |  |  |  |  |
| Q58EX7 | Puratrophin-1 | PLEKHG4 | 0.92 | 1 | | 130.7 | | 5.64 | | 0.13 | 1 | | 0.00 | | 0 | | 0.00 | | 0 | | 0.00 | | 0 | | 0.00 | | 0 | | 0.00 | | 0 | | 0.00 | | 0 | | 0.00 | | 0 | | 1 | | | 0 | | 1 | |  |  |  |  |
| O75391 | Sperm-associated antigen 7 | SPAG7 | 8.81 | 1 | | 26.0 | | 7.91 | | 0.13 | 1 | | 0.00 | | 0 | | 0.00 | | 0 | | 0.00 | | 0 | | 0.00 | | 0 | | 0.00 | | 0 | | 0.00 | | 0 | | 0.00 | | 0 | | 1 | | | 0 | | 1 | |  |  |  |  |
| Q9P127 | Leucine zipper protein 4 | LUZP4 | 8.31 | 1 | | 35.9 | | 9.45 | | 0.13 | 1 | | 0.00 | | 0 | | 0.00 | | 0 | | 0.00 | | 0 | | 0.00 | | 0 | | 0.00 | | 0 | | 0.00 | | 0 | | 0.00 | | 0 | | 1 | | | 0 | | 1 | |  |  |  |  |
| Q8NC56 | LEM domain-containing protein 2 | LEMD2 | 4.57 | 1 | | 56.9 | | 9.00 | | 0.13 | 1 | | 0.00 | | 0 | | 0.00 | | 0 | | 0.00 | | 0 | | 0.00 | | 0 | | 0.00 | | 0 | | 0.00 | | 0 | | 0.00 | | 0 | | 1 | | | 0 | | 1 | |  |  |  |  |
| Q15811 | Intersectin-1 | ITSN1 | 1.28 | 1 | | 195.3 | | 7.77 | | 0.13 | 1 | | 0.00 | | 0 | | 0.00 | | 0 | | 0.00 | | 0 | | 0.00 | | 0 | | 0.00 | | 0 | | 0.00 | | 0 | | 0.00 | | 0 | | 1 | | | 0 | | 1 | |  |  |  |  |
| P07451 | Carbonic anhydrase 3 | CA3 | 6.54 | 1 | | 29.5 | | 7.34 | | 0.13 | 1 | | 0.00 | | 0 | | 0.00 | | 0 | | 0.00 | | 0 | | 0.00 | | 0 | | 0.00 | | 0 | | 0.00 | | 0 | | 0.00 | | 0 | | 1 | | | 0 | | 1 | |  |  |  |  |
| A6NGG8 | Photoreceptor cilium actin regulator | PCARE | 0.78 | 1 | | 139.6 | | 8.07 | | 0.13 | 1 | | 0.00 | | 0 | | 0.00 | | 0 | | 0.00 | | 0 | | 0.00 | | 0 | | 0.00 | | 0 | | 0.00 | | 0 | | 0.00 | | 0 | | 1 | | | 0 | | 1 | |  |  |  |  |
| P47944 | Metallothionein-4 | MT4 | 27.42 | 1 | | 6.5 | | 7.85 | | 0.13 | 1 | | 0.00 | | 0 | | 0.00 | | 0 | | 0.00 | | 0 | | 0.00 | | 0 | | 0.00 | | 0 | | 0.00 | | 0 | | 0.00 | | 0 | | 1 | | | 0 | | 1 | |  |  |  |  |
| A0A087WY85 | Ubiquitin-conjugating enzyme E2 D3 | UBE2D3 | 4.73 | 1 | | 16.8 | | 8.44 | | 0.13 | 1 | | 0.00 | | 0 | | 0.00 | | 0 | | 0.00 | | 0 | | 0.00 | | 0 | | 0.00 | | 0 | | 0.00 | | 0 | | 0.00 | | 0 | | 1 | | | 0 | | 1 | |  |  |  |  |
| Q8N349 | Olfactory receptor 2L13 | OR2L13 | 10.26 | 1 | | 35.6 | | 8.87 | | 0.13 | 1 | | 0.00 | | 0 | | 0.00 | | 0 | | 0.00 | | 0 | | 0.00 | | 0 | | 0.00 | | 0 | | 0.00 | | 0 | | 0.00 | | 0 | | 1 | | | 0 | | 1 | |  |  |  |  |
| A0A590UJR0 | Rap guanine nucleotide exchange factor 5 | RAPGEF5 | 4.08 | 1 | | 101.5 | | 6.73 | | 0.13 | 1 | | 0.00 | | 0 | | 0.00 | | 0 | | 0.00 | | 0 | | 0.00 | | 0 | | 0.00 | | 0 | | 0.00 | | 0 | | 0.00 | | 0 | | 1 | | | 0 | | 1 | |  |  |  |  |
| Q3KNW5 | Solute carrier family 10 member 6 | SLC10A6 | 5.84 | 1 | | 41.2 | | 7.85 | | 0.13 | 1 | | 0.00 | | 0 | | 0.00 | | 0 | | 0.00 | | 0 | | 0.00 | | 0 | | 0.00 | | 0 | | 0.00 | | 0 | | 0.00 | | 0 | | 1 | | | 0 | | 1 | |  |  |  |  |
| A0A0A0MTT5 | Cancer/testis antigen 1 | CTAG1A | 8.57 | 1 | | 20.6 | | 7.39 | | 0.13 | 1 | | 0.00 | | 0 | | 0.00 | | 0 | | 0.00 | | 0 | | 0.00 | | 0 | | 0.00 | | 0 | | 0.00 | | 0 | | 0.00 | | 0 | | 1 | | | 0 | | 1 | |  |  |  |  |
| A0A0A0MQR0 | Cytochrome P450 4F2 | CYP4F2 | 4.23 | 1 | | 59.9 | | 6.90 | | 0.13 | 1 | | 0.00 | | 0 | | 0.00 | | 0 | | 0.00 | | 0 | | 0.00 | | 0 | | 0.00 | | 0 | | 0.00 | | 0 | | 0.00 | | 0 | | 1 | | | 0 | | 1 | |  |  |  |  |
| Q9UMS6 | Synaptopodin-2 | SYNPO2 | 3.11 | 1 | | 117.4 | | 8.57 | | 0.13 | 1 | | 0.00 | | 0 | | 0.00 | | 0 | | 0.00 | | 0 | | 0.00 | | 0 | | 0.00 | | 0 | | 0.00 | | 0 | | 0.00 | | 0 | | 1 | | | 0 | | 1 | |  |  |  |  |
| Q86WK7 | Amphoterin-induced protein 3 | AMIGO3 | 3.97 | 1 | | 55.2 | | 7.87 | | 0.13 | 1 | | 0.00 | | 0 | | 0.00 | | 0 | | 0.00 | | 0 | | 0.00 | | 0 | | 0.00 | | 0 | | 0.00 | | 0 | | 0.00 | | 0 | | 1 | | | 0 | | 1 | |  |  |  |  |
| Q9Y266 | Nuclear migration protein nudC | NUDC | 2.72 | 1 | | 38.2 | | 5.38 | | 0.13 | 1 | | 0.00 | | 0 | | 0.00 | | 0 | | 0.00 | | 0 | | 0.00 | | 0 | | 0.00 | | 0 | | 0.00 | | 0 | | 0.00 | | 0 | | 1 | | | 0 | | 1 | |  |  |  |  |
| Q96NA2 | Rab-interacting lysosomal protein | RILP | 7.23 | 1 | | 44.2 | | 5.59 | | 0.13 | 1 | | 0.00 | | 0 | | 0.00 | | 0 | | 0.00 | | 0 | | 0.00 | | 0 | | 0.00 | | 0 | | 0.00 | | 0 | | 0.00 | | 0 | | 1 | | | 0 | | 1 | |  |  |  |  |
| H0YC20 | PH and SEC7 domain-containing protein 3 (Fragment) | PSD3 | 6.06 | 1 | | 19.1 | | 7.77 | | 0.13 | 1 | | 0.00 | | 0 | | 0.00 | | 0 | | 0.00 | | 0 | | 0.00 | | 0 | | 0.00 | | 0 | | 0.00 | | 0 | | 0.00 | | 0 | | 1 | | | 0 | | 1 | |  |  |  |  |
| B4E0Y9 | Serine/threonine-protein kinase 26 | STK26 | 4.11 | 1 | | 49.2 | | 5.68 | | 0.13 | 1 | | 0.00 | | 0 | | 0.00 | | 0 | | 0.00 | | 0 | | 0.00 | | 0 | | 0.00 | | 0 | | 0.00 | | 0 | | 0.00 | | 0 | | 1 | | | 0 | | 1 | |  |  |  |  |
| P10600 | Transforming growth factor beta-3 proprotein | TGFB3 | 2.43 | 1 | | 47.3 | | 8.03 | | 0.13 | 1 | | 0.00 | | 0 | | 0.00 | | 0 | | 0.00 | | 0 | | 0.00 | | 0 | | 0.00 | | 0 | | 0.00 | | 0 | | 0.00 | | 0 | | 1 | | | 0 | | 1 | |  |  |  |  |
| Q969H0 | F-box/WD repeat-containing protein 7 | FBXW7 | 2.97 | 1 | | 79.6 | | 5.80 | | 0.13 | 1 | | 0.00 | | 0 | | 0.00 | | 0 | | 0.00 | | 0 | | 0.00 | | 0 | | 0.00 | | 0 | | 0.00 | | 0 | | 0.00 | | 0 | | 1 | | | 0 | | 1 | |  |  |  |  |
| Q8WWW8 | GRB2-associated-binding protein 3 | GAB3 | 1.88 | 1 | | 65.5 | | 7.23 | | 0.13 | 1 | | 0.00 | | 0 | | 0.00 | | 0 | | 0.00 | | 0 | | 0.00 | | 0 | | 0.00 | | 0 | | 0.00 | | 0 | | 0.00 | | 0 | | 1 | | | 0 | | 1 | |  |  |  |  |
| Q5JPI9 | EEF1A lysine methyltransferase 2 | EEF1AKMT2 | 6.87 | 1 | | 31.8 | | 6.16 | | 0.13 | 1 | | 0.00 | | 0 | | 0.00 | | 0 | | 0.00 | | 0 | | 0.00 | | 0 | | 0.00 | | 0 | | 0.00 | | 0 | | 0.00 | | 0 | | 1 | | | 0 | | 1 | |  |  |  |  |
| O94933 | SLIT and NTRK-like protein 3 | SLITRK3 | 2.76 | 1 | | 108.9 | | 7.37 | | 0.13 | 1 | | 0.00 | | 0 | | 0.00 | | 0 | | 0.00 | | 0 | | 0.00 | | 0 | | 0.00 | | 0 | | 0.00 | | 0 | | 0.00 | | 0 | | 1 | | | 0 | | 1 | |  |  |  |  |
| Q9NW82 | WD repeat-containing protein 70 | WDR70 | 1.68 | 1 | | 73.2 | | 6.33 | | 0.13 | 1 | | 0.00 | | 0 | | 0.00 | | 0 | | 0.00 | | 0 | | 0.00 | | 0 | | 0.00 | | 0 | | 0.00 | | 0 | | 0.00 | | 0 | | 1 | | | 0 | | 1 | |  |  |  |  |
| B0QYN7 | SUMO-conjugating enzyme UBC9 | UBE2I | 7.07 | 1 | | 20.4 | | 8.46 | | 0.13 | 1 | | 0.00 | | 0 | | 0.00 | | 0 | | 0.00 | | 0 | | 0.00 | | 0 | | 0.00 | | 0 | | 0.00 | | 0 | | 0.00 | | 0 | | 1 | | | 0 | | 1 | |  |  |  |  |
| Q96T17 | MAP7 domain-containing protein 2 | MAP7D2 | 1.78 | 1 | | 81.9 | | 8.84 | | 0.13 | 1 | | 0.00 | | 0 | | 0.00 | | 0 | | 0.00 | | 0 | | 0.00 | | 0 | | 0.00 | | 0 | | 0.00 | | 0 | | 0.00 | | 0 | | 1 | | | 0 | | 1 | |  |  |  |  |
| H0YHJ4 | Liprin-alpha-2 (Fragment) | PPFIA2 | 17.55 | 1 | | 21.5 | | 8.94 | | 0.13 | 1 | | 0.00 | | 0 | | 0.00 | | 0 | | 0.00 | | 0 | | 0.00 | | 0 | | 0.00 | | 0 | | 0.00 | | 0 | | 0.00 | | 0 | | 1 | | | 0 | | 1 | |  |  |  |  |
| Q8NE71 | ATP-binding cassette sub-family F member 1 | ABCF1 | 5.56 | 1 | | 95.9 | | 6.80 | | 0.13 | 1 | | 0.00 | | 0 | | 0.00 | | 0 | | 0.00 | | 0 | | 0.00 | | 0 | | 0.00 | | 0 | | 0.00 | | 0 | | 0.00 | | 0 | | 1 | | | 0 | | 1 | |  |  |  |  |
| H3BLT9 | NLR family CARD domain-containing protein 3 | NLRC3 | 4.77 | 1 | | 119.5 | | 8.24 | | 0.13 | 1 | | 0.00 | | 0 | | 0.00 | | 0 | | 0.00 | | 0 | | 0.00 | | 0 | | 0.00 | | 0 | | 0.00 | | 0 | | 0.00 | | 0 | | 1 | | | 0 | | 1 | |  |  |  |  |
| P08069 | Insulin-like growth factor 1 receptor | IGF1R | 2.78 | 1 | | 154.7 | | 5.80 | | 0.13 | 1 | | 0.00 | | 0 | | 0.00 | | 0 | | 0.00 | | 0 | | 0.00 | | 0 | | 0.00 | | 0 | | 0.00 | | 0 | | 0.00 | | 0 | | 1 | | | 0 | | 1 | |  |  |  |  |
| Q03181 | Peroxisome proliferator-activated receptor delta | PPARD | 13.15 | 1 | | 49.9 | | 7.59 | | 0.13 | 1 | | 0.00 | | 0 | | 0.00 | | 0 | | 0.00 | | 0 | | 0.00 | | 0 | | 0.00 | | 0 | | 0.00 | | 0 | | 0.00 | | 0 | | 1 | | | 0 | | 1 | |  |  |  |  |
| Q9H2U1 | ATP-dependent DNA/RNA helicase DHX36 | DHX36 | 3.08 | 1 | | 114.7 | | 7.68 | | 0.13 | 1 | | 0.00 | | 0 | | 0.00 | | 0 | | 0.00 | | 0 | | 0.00 | | 0 | | 0.00 | | 0 | | 0.00 | | 0 | | 0.00 | | 0 | | 1 | | | 0 | | 1 | |  |  |  |  |
| O15061 | Synemin | SYNM | 1.09 | 1 | | 172.7 | | 5.16 | | 0.13 | 1 | | 0.00 | | 0 | | 0.00 | | 0 | | 0.00 | | 0 | | 0.00 | | 0 | | 0.00 | | 0 | | 0.00 | | 0 | | 0.00 | | 0 | | 1 | | | 0 | | 1 | |  |  |  |  |
| F5GYJ8 | Ubiquitin thioesterase OTUB1 | OTUB1 | 5.24 | 1 | | 32.3 | | 4.96 | | 0.13 | 1 | | 0.00 | | 0 | | 0.00 | | 0 | | 0.00 | | 0 | | 0.00 | | 0 | | 0.00 | | 0 | | 0.00 | | 0 | | 0.00 | | 0 | | 1 | | | 0 | | 1 | |  |  |  |  |
| Q15572 | TATA box-binding protein-associated factor RNA polymerase I subunit C | TAF1C | 2.53 | 1 | | 95.2 | | 8.59 | | 0.13 | 1 | | 0.00 | | 0 | | 0.00 | | 0 | | 0.00 | | 0 | | 0.00 | | 0 | | 0.00 | | 0 | | 0.00 | | 0 | | 0.00 | | 0 | | 1 | | | 0 | | 1 | |  |  |  |  |
| A0A0G2JNW1 | Testis-expressed basic protein 1 | TSBP1 | 2.47 | 1 | | 62.7 | | 9.33 | | 0.13 | 1 | | 0.00 | | 0 | | 0.00 | | 0 | | 0.00 | | 0 | | 0.00 | | 0 | | 0.00 | | 0 | | 0.00 | | 0 | | 0.00 | | 0 | | 1 | | | 0 | | 1 | |  |  |  |  |
| Q14680 | Maternal embryonic leucine zipper kinase | MELK | 3.99 | 1 | | 74.6 | | 8.72 | | 0.13 | 1 | | 0.00 | | 0 | | 0.00 | | 0 | | 0.00 | | 0 | | 0.00 | | 0 | | 0.00 | | 0 | | 0.00 | | 0 | | 0.00 | | 0 | | 1 | | | 0 | | 1 | |  |  |  |  |
| Q9NZY2 | Putative uncharacterized protein FAM30A | FAM30A | 6.72 | 1 | | 14.6 | | 7.30 | | 0.13 | 1 | | 0.00 | | 0 | | 0.00 | | 0 | | 0.00 | | 0 | | 0.00 | | 0 | | 0.00 | | 0 | | 0.00 | | 0 | | 0.00 | | 0 | | 1 | | | 0 | | 1 | |  |  |  |  |
| Q9Y2V7 | Conserved oligomeric Golgi complex subunit 6 | COG6 | 3.35 | 1 | | 73.2 | | 5.76 | | 0.13 | 1 | | 0.00 | | 0 | | 0.00 | | 0 | | 0.00 | | 0 | | 0.00 | | 0 | | 0.00 | | 0 | | 0.00 | | 0 | | 0.00 | | 0 | | 1 | | | 0 | | 1 | |  |  |  |  |
| Q8WW59 | SPRY domain-containing protein 4 | SPRYD4 | 8.21 | 1 | | 23.1 | | 6.93 | | 0.13 | 1 | | 0.00 | | 0 | | 0.00 | | 0 | | 0.00 | | 0 | | 0.00 | | 0 | | 0.00 | | 0 | | 0.00 | | 0 | | 0.00 | | 0 | | 1 | | | 0 | | 1 | |  |  |  |  |
| Q8IVD9 | NudC domain-containing protein 3 | NUDCD3 | 8.31 | 1 | | 40.8 | | 5.25 | | 0.13 | 1 | | 0.00 | | 0 | | 0.00 | | 0 | | 0.00 | | 0 | | 0.00 | | 0 | | 0.00 | | 0 | | 0.00 | | 0 | | 0.00 | | 0 | | 1 | | | 0 | | 1 | |  |  |  |  |
| E9PFE2 | General transcription factor II-I repeat domain-containing protein 1 | GTF2IRD1 | 0.83 | 1 | | 106.0 | | 7.58 | | 0.13 | 1 | | 0.00 | | 0 | | 0.00 | | 0 | | 0.00 | | 0 | | 0.00 | | 0 | | 0.00 | | 0 | | 0.00 | | 0 | | 0.00 | | 0 | | 1 | | | 0 | | 1 | |  |  |  |  |
| Q12884 | Prolyl endopeptidase FAP | FAP | 2.24 | 1 | | 87.7 | | 6.65 | | 0.13 | 1 | | 0.00 | | 0 | | 0.00 | | 0 | | 0.00 | | 0 | | 0.00 | | 0 | | 0.00 | | 0 | | 0.00 | | 0 | | 0.00 | | 0 | | 1 | | | 0 | | 1 | |  |  |  |  |
| Q9NPF2 | Carbohydrate sulfotransferase 11 | CHST11 | 7.39 | 1 | | 41.5 | | 8.85 | | 0.13 | 1 | | 0.00 | | 0 | | 0.00 | | 0 | | 0.00 | | 0 | | 0.00 | | 0 | | 0.00 | | 0 | | 0.00 | | 0 | | 0.00 | | 0 | | 1 | | | 0 | | 1 | |  |  |  |  |
| Q9Y3T9 | Nucleolar complex protein 2 homolog | NOC2L | 1.74 | 1 | | 84.9 | | 5.62 | | 0.13 | 1 | | 0.00 | | 0 | | 0.00 | | 0 | | 0.00 | | 0 | | 0.00 | | 0 | | 0.00 | | 0 | | 0.00 | | 0 | | 0.00 | | 0 | | 1 | | | 0 | | 1 | |  |  |  |  |
| Q96A46 | Mitoferrin-2 | SLC25A28 | 7.42 | 1 | | 39.2 | | 8.59 | | 0.13 | 1 | | 0.00 | | 0 | | 0.00 | | 0 | | 0.00 | | 0 | | 0.00 | | 0 | | 0.00 | | 0 | | 0.00 | | 0 | | 0.00 | | 0 | | 1 | | | 0 | | 1 | |  |  |  |  |
| F8VXK0 | Peptidyl-prolyl cis-trans isomerase FKBP11 | FKBP11 | 12.50 | 1 | | 8.2 | | 7.36 | | 0.13 | 1 | | 0.00 | | 0 | | 0.00 | | 0 | | 0.00 | | 0 | | 0.00 | | 0 | | 0.00 | | 0 | | 0.00 | | 0 | | 0.00 | | 0 | | 1 | | | 0 | | 1 | |  |  |  |  |
| Q8IZS5 | Orofacial cleft 1 candidate gene 1 protein | OFCC1 | 11.26 | 1 | | 26.7 | | 6.25 | | 0.13 | 1 | | 0.00 | | 0 | | 0.00 | | 0 | | 0.00 | | 0 | | 0.00 | | 0 | | 0.00 | | 0 | | 0.00 | | 0 | | 0.00 | | 0 | | 1 | | | 0 | | 1 | |  |  |  |  |
| Q9HC10 | Otoferlin | OTOF | 0.35 | 1 | | 226.6 | | 5.69 | | 0.13 | 1 | | 0.00 | | 0 | | 0.00 | | 0 | | 0.00 | | 0 | | 0.00 | | 0 | | 0.00 | | 0 | | 0.00 | | 0 | | 0.00 | | 0 | | 1 | | | 0 | | 1 | |  |  |  |  |
| Q9Y619 | Mitochondrial ornithine transporter 1 | SLC25A15 | 5.32 | 1 | | 32.7 | | 9.13 | | 0.13 | 1 | | 0.00 | | 0 | | 0.00 | | 0 | | 0.00 | | 0 | | 0.00 | | 0 | | 0.00 | | 0 | | 0.00 | | 0 | | 0.00 | | 0 | | 1 | | | 0 | | 1 | |  |  |  |  |
| Q13363 | C-terminal-binding protein 1 | CTBP1 | 10.00 | 1 | | 47.5 | | 6.77 | | 0.13 | 1 | | 0.00 | | 0 | | 0.00 | | 0 | | 0.00 | | 0 | | 0.00 | | 0 | | 0.00 | | 0 | | 0.00 | | 0 | | 0.00 | | 0 | | 1 | | | 0 | | 1 | |  |  |  |  |
| J3QLE2 | Dual-specificity mitogen-activated protein kinase kinase 4 | MAP2K4 | 100.00 | 1 | | 3.9 | | 5.48 | | 0.13 | 1 | | 0.00 | | 0 | | 0.00 | | 0 | | 0.00 | | 0 | | 0.00 | | 0 | | 0.00 | | 0 | | 0.00 | | 0 | | 0.00 | | 0 | | 1 | | | 0 | | 1 | |  |  |  |  |
| A0A087WZ85 | Roundabout homolog 1 | ROBO1 | 3.34 | 1 | | 166.6 | | 6.13 | | 0.13 | 1 | | 0.00 | | 0 | | 0.00 | | 0 | | 0.00 | | 0 | | 0.00 | | 0 | | 0.00 | | 0 | | 0.00 | | 0 | | 0.00 | | 0 | | 1 | | | 0 | | 1 | |  |  |  |  |
| Q9BTE7 | DCN1-like protein 5 | DCUN1D5 | 8.44 | 1 | | 27.5 | | 5.58 | | 0.13 | 1 | | 0.00 | | 0 | | 0.00 | | 0 | | 0.00 | | 0 | | 0.00 | | 0 | | 0.00 | | 0 | | 0.00 | | 0 | | 0.00 | | 0 | | 1 | | | 0 | | 1 | |  |  |  |  |
| B4E2M5 | Ankyrin repeat domain-containing protein 66 | ANKRD66 | 7.97 | 1 | | 27.9 | | 9.16 | | 0.13 | 1 | | 0.00 | | 0 | | 0.00 | | 0 | | 0.00 | | 0 | | 0.00 | | 0 | | 0.00 | | 0 | | 0.00 | | 0 | | 0.00 | | 0 | | 1 | | | 0 | | 1 | |  |  |  |  |
| O14657 | Torsin-1B | TOR1B | 6.25 | 1 | | 38.0 | | 8.54 | | 0.13 | 1 | | 0.00 | | 0 | | 0.00 | | 0 | | 0.00 | | 0 | | 0.00 | | 0 | | 0.00 | | 0 | | 0.00 | | 0 | | 0.00 | | 0 | | 1 | | | 0 | | 1 | |  |  |  |  |
| Q8NGR2 | Olfactory receptor 1L6 | OR1L6 | 14.12 | 1 | | 39.5 | | 9.52 | | 0.13 | 1 | | 0.00 | | 0 | | 0.00 | | 0 | | 0.00 | | 0 | | 0.00 | | 0 | | 0.00 | | 0 | | 0.00 | | 0 | | 0.00 | | 0 | | 1 | | | 0 | | 1 | |  |  |  |  |
| P11686 | Pulmonary surfactant-associated protein C | SFTPC | 14.21 | 1 | | 21.0 | | 6.65 | | 0.13 | 1 | | 0.00 | | 0 | | 0.00 | | 0 | | 0.00 | | 0 | | 0.00 | | 0 | | 0.00 | | 0 | | 0.00 | | 0 | | 0.00 | | 0 | | 1 | | | 0 | | 1 | |  |  |  |  |
| Q32MZ4 | Leucine-rich repeat flightless-interacting protein 1 | LRRFIP1 | 1.49 | 1 | | 89.2 | | 4.65 | | 0.13 | 1 | | 0.00 | | 0 | | 0.00 | | 0 | | 0.00 | | 0 | | 0.00 | | 0 | | 0.00 | | 0 | | 0.00 | | 0 | | 0.00 | | 0 | | 1 | | | 0 | | 1 | |  |  |  |  |
| Q8NC67 | Neuropilin and tolloid-like protein 2 | NETO2 | 7.24 | 1 | | 59.4 | | 6.81 | | 0.13 | 1 | | 0.00 | | 0 | | 0.00 | | 0 | | 0.00 | | 0 | | 0.00 | | 0 | | 0.00 | | 0 | | 0.00 | | 0 | | 0.00 | | 0 | | 1 | | | 0 | | 1 | |  |  |  |  |
| Q6IA86 | Elongator complex protein 2 | ELP2 | 2.54 | 1 | | 92.4 | | 5.96 | | 0.13 | 1 | | 0.00 | | 0 | | 0.00 | | 0 | | 0.00 | | 0 | | 0.00 | | 0 | | 0.00 | | 0 | | 0.00 | | 0 | | 0.00 | | 0 | | 1 | | | 0 | | 1 | |  |  |  |  |
| E7EW31 | Proline-rich basic protein 1 | PROB1 | 5.52 | 1 | | 106.9 | | 9.63 | | 0.13 | 1 | | 0.00 | | 0 | | 0.00 | | 0 | | 0.00 | | 0 | | 0.00 | | 0 | | 0.00 | | 0 | | 0.00 | | 0 | | 0.00 | | 0 | | 1 | | | 0 | | 1 | |  |  |  |  |
| Q01831 | DNA repair protein complementing XP-C cells | XPC | 3.72 | 1 | | 105.9 | | 8.90 | | 0.13 | 1 | | 0.00 | | 0 | | 0.00 | | 0 | | 0.00 | | 0 | | 0.00 | | 0 | | 0.00 | | 0 | | 0.00 | | 0 | | 0.00 | | 0 | | 1 | | | 0 | | 1 | |  |  |  |  |
| Q5JRC9 | Protein FAM47A | FAM47A | 1.26 | 1 | | 90.5 | | 9.11 | | 0.13 | 1 | | 0.00 | | 0 | | 0.00 | | 0 | | 0.00 | | 0 | | 0.00 | | 0 | | 0.00 | | 0 | | 0.00 | | 0 | | 0.00 | | 0 | | 1 | | | 0 | | 1 | |  |  |  |  |
| B1AKR6 | Dynein light chain roadblock-type 1 | DYNLRB1 | 18.24 | 1 | | 16.2 | | 7.02 | | 0.13 | 1 | | 0.00 | | 0 | | 0.00 | | 0 | | 0.00 | | 0 | | 0.00 | | 0 | | 0.00 | | 0 | | 0.00 | | 0 | | 0.00 | | 0 | | 1 | | | 0 | | 1 | |  |  |  |  |
| P13349 | Myogenic factor 5 | MYF5 | 5.88 | 1 | | 28.3 | | 6.30 | | 0.13 | 1 | | 0.00 | | 0 | | 0.00 | | 0 | | 0.00 | | 0 | | 0.00 | | 0 | | 0.00 | | 0 | | 0.00 | | 0 | | 0.00 | | 0 | | 1 | | | 0 | | 1 | |  |  |  |  |
| H0YJI1 | SRA stem-loop-interacting RNA-binding protein, mitochondrial (Fragment) | SLIRP | 20.00 | 1 | | 4.3 | | 12.70 | | 0.13 | 1 | | 0.00 | | 0 | | 0.00 | | 0 | | 0.00 | | 0 | | 0.00 | | 0 | | 0.00 | | 0 | | 0.00 | | 0 | | 0.00 | | 0 | | 1 | | | 0 | | 1 | |  |  |  |  |
| A0A1B0GW21 | KIF-binding protein (Fragment) | KIFBP | 14.83 | 1 | | 24.1 | | 5.43 | | 0.13 | 1 | | 0.00 | | 0 | | 0.00 | | 0 | | 0.00 | | 0 | | 0.00 | | 0 | | 0.00 | | 0 | | 0.00 | | 0 | | 0.00 | | 0 | | 1 | | | 0 | | 1 | |  |  |  |  |
| P11464 | Preancy-specific beta-1-glycoprotein 1 | PSG1 | 6.92 | 1 | | 47.2 | | 8.12 | | 0.13 | 1 | | 0.00 | | 0 | | 0.00 | | 0 | | 0.00 | | 0 | | 0.00 | | 0 | | 0.00 | | 0 | | 0.00 | | 0 | | 0.00 | | 0 | | 1 | | | 0 | | 1 | |  |  |  |  |
| P35318 | ADM | ADM | 17.84 | 1 | | 20.4 | | 10.84 | | 0.13 | 1 | | 0.00 | | 0 | | 0.00 | | 0 | | 0.00 | | 0 | | 0.00 | | 0 | | 0.00 | | 0 | | 0.00 | | 0 | | 0.00 | | 0 | | 1 | | | 0 | | 1 | |  |  |  |  |
| Q8N239 | Kelch-like protein 34 | KLHL34 | 4.97 | 1 | | 70.6 | | 5.60 | | 0.13 | 1 | | 0.00 | | 0 | | 0.00 | | 0 | | 0.00 | | 0 | | 0.00 | | 0 | | 0.00 | | 0 | | 0.00 | | 0 | | 0.00 | | 0 | | 1 | | | 0 | | 1 | |  |  |  |  |
| Q6ZW61 | Bardet-Biedl syndrome 12 protein | BBS12 | 6.20 | 1 | | 79.0 | | 6.20 | | 0.13 | 1 | | 0.00 | | 0 | | 0.00 | | 0 | | 0.00 | | 0 | | 0.00 | | 0 | | 0.00 | | 0 | | 0.00 | | 0 | | 0.00 | | 0 | | 1 | | | 0 | | 1 | |  |  |  |  |
| R4GN21 | UbiA prenyltransferase domain-containing protein 1 (Fragment) | UBIAD1 | 38.96 | 1 | | 8.2 | | 7.24 | | 0.13 | 1 | | 0.00 | | 0 | | 0.00 | | 0 | | 0.00 | | 0 | | 0.00 | | 0 | | 0.00 | | 0 | | 0.00 | | 0 | | 0.00 | | 0 | | 1 | | | 0 | | 1 | |  |  |  |  |
| P28223 | 5-hydroxytryptamine receptor 2A | HTR2A | 1.91 | 1 | | 52.6 | | 7.66 | | 0.13 | 1 | | 0.00 | | 0 | | 0.00 | | 0 | | 0.00 | | 0 | | 0.00 | | 0 | | 0.00 | | 0 | | 0.00 | | 0 | | 0.00 | | 0 | | 1 | | | 0 | | 1 | |  |  |  |  |
| O43149 | Zinc finger ZZ-type and EF-hand domain-containing protein 1 | ZZEF1 | 0.34 | 1 | | 330.9 | | 5.95 | | 0.13 | 1 | | 0.00 | | 0 | | 0.00 | | 0 | | 0.00 | | 0 | | 0.00 | | 0 | | 0.00 | | 0 | | 0.00 | | 0 | | 0.00 | | 0 | | 1 | | | 0 | | 1 | |  |  |  |  |
| Q8NG81 | Olfactory receptor 2M7 | OR2M7 | 13.14 | 1 | | 34.9 | | 7.58 | | 0.13 | 1 | | 0.00 | | 0 | | 0.00 | | 0 | | 0.00 | | 0 | | 0.00 | | 0 | | 0.00 | | 0 | | 0.00 | | 0 | | 0.00 | | 0 | | 1 | | | 0 | | 1 | |  |  |  |  |
| Q8TDS5 | Oxoeicosanoid receptor 1 | OXER1 | 9.22 | 1 | | 45.8 | | 9.66 | | 0.13 | 1 | | 0.00 | | 0 | | 0.00 | | 0 | | 0.00 | | 0 | | 0.00 | | 0 | | 0.00 | | 0 | | 0.00 | | 0 | | 0.00 | | 0 | | 1 | | | 0 | | 1 | |  |  |  |  |
| Q92508 | Piezo-type mechanosensitive ion channel component 1 | PIEZO1 | 0.52 | 1 | | 286.6 | | 7.47 | | 0.13 | 1 | | 0.00 | | 0 | | 0.00 | | 0 | | 0.00 | | 0 | | 0.00 | | 0 | | 0.00 | | 0 | | 0.00 | | 0 | | 0.00 | | 0 | | 1 | | | 0 | | 1 | |  |  |  |  |
| Q8NGE5 | Olfactory receptor 10A7 | OR10A7 | 9.18 | 1 | | 35.7 | | 8.46 | | 0.13 | 1 | | 0.00 | | 0 | | 0.00 | | 0 | | 0.00 | | 0 | | 0.00 | | 0 | | 0.00 | | 0 | | 0.00 | | 0 | | 0.00 | | 0 | | 1 | | | 0 | | 1 | |  |  |  |  |
| A0A0G2JN34 | Transient receptor potential cation channel subfamily M member 1 | TRPM1 | 1.46 | 1 | | 186.4 | | 7.14 | | 0.13 | 1 | | 0.00 | | 0 | | 0.00 | | 0 | | 0.00 | | 0 | | 0.00 | | 0 | | 0.00 | | 0 | | 0.00 | | 0 | | 0.00 | | 0 | | 1 | | | 0 | | 1 | |  |  |  |  |
| A5PLK6 | Regulator of G-protein sialing protein-like | RGSL1 | 4.55 | 1 | | 125.6 | | 9.01 | | 0.13 | 1 | | 0.00 | | 0 | | 0.00 | | 0 | | 0.00 | | 0 | | 0.00 | | 0 | | 0.00 | | 0 | | 0.00 | | 0 | | 0.00 | | 0 | | 1 | | | 0 | | 1 | |  |  |  |  |
| C9JXA7 | Ragulator complex protein LAMTOR4 | LAMTOR4 | 38.69 | 1 | | 14.3 | | 7.36 | | 0.13 | 1 | | 0.00 | | 0 | | 0.00 | | 0 | | 0.00 | | 0 | | 0.00 | | 0 | | 0.00 | | 0 | | 0.00 | | 0 | | 0.00 | | 0 | | 1 | | | 0 | | 1 | |  |  |  |  |
| U3KPS9 | Adenylate cyclase type 10 | ADCY10 | 63.01 | 1 | | 8.7 | | 5.83 | | 0.13 | 1 | | 0.00 | | 0 | | 0.00 | | 0 | | 0.00 | | 0 | | 0.00 | | 0 | | 0.00 | | 0 | | 0.00 | | 0 | | 0.00 | | 0 | | 1 | | | 0 | | 1 | |  |  |  |  |
| P38398 | Breast cancer type 1 susceptibility protein | BRCA1 | 0.86 | 1 | | 207.6 | | 5.41 | | 0.13 | 1 | | 0.00 | | 0 | | 0.00 | | 0 | | 0.00 | | 0 | | 0.00 | | 0 | | 0.00 | | 0 | | 0.00 | | 0 | | 0.00 | | 0 | | 1 | | | 0 | | 1 | |  |  |  |  |
| Q8IYU8 | Calcium uptake protein 2, mitochondrial | MICU2 | 5.99 | 1 | | 49.6 | | 9.09 | | 0.13 | 1 | | 0.00 | | 0 | | 0.00 | | 0 | | 0.00 | | 0 | | 0.00 | | 0 | | 0.00 | | 0 | | 0.00 | | 0 | | 0.00 | | 0 | | 1 | | | 0 | | 1 | |  |  |  |  |
| Q3KQV3 | Zinc finger protein 792 | ZNF792 | 4.43 | 1 | | 71.5 | | 8.02 | | 0.13 | 1 | | 0.00 | | 0 | | 0.00 | | 0 | | 0.00 | | 0 | | 0.00 | | 0 | | 0.00 | | 0 | | 0.00 | | 0 | | 0.00 | | 0 | | 1 | | | 0 | | 1 | |  |  |  |  |
| H0YMP6 | Ubiquitin carboxyl-terminal hydrolase 3 (Fragment) | USP3 | 15.91 | 1 | | 14.6 | | 7.11 | | 0.13 | 1 | | 0.00 | | 0 | | 0.00 | | 0 | | 0.00 | | 0 | | 0.00 | | 0 | | 0.00 | | 0 | | 0.00 | | 0 | | 0.00 | | 0 | | 1 | | | 0 | | 1 | |  |  |  |  |
| Q9ULJ8 | Neurabin-1 | PPP1R9A | 5.19 | 1 | | 123.3 | | 5.10 | | 0.13 | 1 | | 0.00 | | 0 | | 0.00 | | 0 | | 0.00 | | 0 | | 0.00 | | 0 | | 0.00 | | 0 | | 0.00 | | 0 | | 0.00 | | 0 | | 1 | | | 0 | | 1 | |  |  |  |  |
| A0A494C087 | Receptor-type tyrosine-protein phosphatase zeta | PTPRZ1 | 1.36 | 1 | | 258.5 | | 4.91 | | 0.13 | 1 | | 0.00 | | 0 | | 0.00 | | 0 | | 0.00 | | 0 | | 0.00 | | 0 | | 0.00 | | 0 | | 0.00 | | 0 | | 0.00 | | 0 | | 1 | | | 0 | | 1 | |  |  |  |  |
| O75908 | Sterol O-acyltransferase 2 | SOAT2 | 8.24 | 1 | | 59.9 | | 8.63 | | 0.13 | 1 | | 0.00 | | 0 | | 0.00 | | 0 | | 0.00 | | 0 | | 0.00 | | 0 | | 0.00 | | 0 | | 0.00 | | 0 | | 0.00 | | 0 | | 1 | | | 0 | | 1 | |  |  |  |  |
| Q8N392 | Rho GTPase-activating protein 18 | ARHGAP18 | 7.24 | 1 | | 74.9 | | 6.44 | | 0.13 | 1 | | 0.00 | | 0 | | 0.00 | | 0 | | 0.00 | | 0 | | 0.00 | | 0 | | 0.00 | | 0 | | 0.00 | | 0 | | 0.00 | | 0 | | 1 | | | 0 | | 1 | |  |  |  |  |
| P00450 | Ceruloplasmin | CP | 0.75 | 1 | | 122.1 | | 5.72 | | 0.13 | 1 | | 0.00 | | 0 | | 0.00 | | 0 | | 0.00 | | 0 | | 0.00 | | 0 | | 0.00 | | 0 | | 0.00 | | 0 | | 0.00 | | 0 | | 1 | | | 0 | | 1 | |  |  |  |  |
| Q14393 | Growth arrest-specific protein 6 | GAS6 | 3.39 | 1 | | 74.9 | | 5.69 | | 0.13 | 1 | | 0.00 | | 0 | | 0.00 | | 0 | | 0.00 | | 0 | | 0.00 | | 0 | | 0.00 | | 0 | | 0.00 | | 0 | | 0.00 | | 0 | | 1 | | | 0 | | 1 | |  |  |  |  |
| A0A087WZK0 | Deoxyhypusine synthase | DHPS | 4.86 | 1 | | 41.1 | | 5.47 | | 0.13 | 1 | | 0.00 | | 0 | | 0.00 | | 0 | | 0.00 | | 0 | | 0.00 | | 0 | | 0.00 | | 0 | | 0.00 | | 0 | | 0.00 | | 0 | | 1 | | | 0 | | 1 | |  |  |  |  |
| Q99965 | Disintegrin and metalloproteinase domain-containing protein 2 | ADAM2 | 2.31 | 1 | | 82.4 | | 6.15 | | 0.13 | 1 | | 0.00 | | 0 | | 0.00 | | 0 | | 0.00 | | 0 | | 0.00 | | 0 | | 0.00 | | 0 | | 0.00 | | 0 | | 0.00 | | 0 | | 1 | | | 0 | | 1 | |  |  |  |  |
| A0A087WV75 | Neural cell adhesion molecule 1 | NCAM1 | 3.17 | 1 | | 97.3 | | 4.93 | | 0.13 | 1 | | 0.00 | | 0 | | 0.00 | | 0 | | 0.00 | | 0 | | 0.00 | | 0 | | 0.00 | | 0 | | 0.00 | | 0 | | 0.00 | | 0 | | 1 | | | 0 | | 1 | |  |  |  |  |
| O14782 | Kinesin-like protein KIF3C | KIF3C | 1.89 | 1 | | 89.4 | | 8.22 | | 0.13 | 1 | | 0.00 | | 0 | | 0.00 | | 0 | | 0.00 | | 0 | | 0.00 | | 0 | | 0.00 | | 0 | | 0.00 | | 0 | | 0.00 | | 0 | | 1 | | | 0 | | 1 | |  |  |  |  |
| P36021 | Monocarboxylate transporter 8 | SLC16A2 | 4.27 | 1 | | 59.5 | | 5.59 | | 0.13 | 1 | | 0.00 | | 0 | | 0.00 | | 0 | | 0.00 | | 0 | | 0.00 | | 0 | | 0.00 | | 0 | | 0.00 | | 0 | | 0.00 | | 0 | | 1 | | | 0 | | 1 | |  |  |  |  |
| Q9NS39 | Double-stranded RNA-specific editase B2 | ADARB2 | 3.11 | 1 | | 80.6 | | 10.17 | | 0.13 | 1 | | 0.00 | | 0 | | 0.00 | | 0 | | 0.00 | | 0 | | 0.00 | | 0 | | 0.00 | | 0 | | 0.00 | | 0 | | 0.00 | | 0 | | 1 | | | 0 | | 1 | |  |  |  |  |
| A0A087WWT4 | Tetraspanin | TSPAN10 | 6.11 | 1 | | 40.8 | | 6.92 | | 0.13 | 1 | | 0.00 | | 0 | | 0.00 | | 0 | | 0.00 | | 0 | | 0.00 | | 0 | | 0.00 | | 0 | | 0.00 | | 0 | | 0.00 | | 0 | | 1 | | | 0 | | 1 | |  |  |  |  |
| Q8TCX5 | Rhophilin-1 | RHPN1 | 1.19 | 1 | | 73.5 | | 7.53 | | 0.13 | 1 | | 0.00 | | 0 | | 0.00 | | 0 | | 0.00 | | 0 | | 0.00 | | 0 | | 0.00 | | 0 | | 0.00 | | 0 | | 0.00 | | 0 | | 1 | | | 0 | | 1 | |  |  |  |  |
| Q49AR2 | UPF0489 protein C5orf22 | C5orf22 | 6.79 | 1 | | 49.9 | | 4.78 | | 0.13 | 1 | | 0.00 | | 0 | | 0.00 | | 0 | | 0.00 | | 0 | | 0.00 | | 0 | | 0.00 | | 0 | | 0.00 | | 0 | | 0.00 | | 0 | | 1 | | | 0 | | 1 | |  |  |  |  |
| P07585 | Decorin | DCN | 6.96 | 2 | | 39.7 | | 8.54 | | 0.00 | 0 | | 0.00 | | 0 | | 0.48 | | 1 | | 0.00 | | 0 | | 0.00 | | 0 | | 0.00 | | 0 | | 0.00 | | 0 | | 0.00 | | 0 | | 1 | | | 0 | | 1 | |  |  |  |  |
| Q14114 | Low-density lipoprotein receptor-related protein 8 | LRP8 | 1.77 | 1 | | 105.6 | | 5.05 | | 0.00 | 0 | | 0.00 | | 0 | | 0.44 | | 2 | | 0.00 | | 0 | | 0.00 | | 0 | | 0.00 | | 0 | | 0.00 | | 0 | | 0.00 | | 0 | | 1 | | | 0 | | 1 | |  |  |  |  |
| Q6P1J6 | Phospholipase B1, membrane-associated | PLB1 | 0.62 | 1 | | 163.0 | | 5.88 | | 0.00 | 0 | | 0.00 | | 0 | | 0.44 | | 1 | | 0.00 | | 0 | | 0.00 | | 0 | | 0.00 | | 0 | | 0.00 | | 0 | | 0.00 | | 0 | | 1 | | | 0 | | 1 | |  |  |  |  |
| A0A087WTB0 | DNA repair and recombination protein RAD54-like (Fragment) | RAD54L | 13.28 | 2 | | 28.5 | | 8.72 | | 0.00 | 0 | | 0.00 | | 0 | | 0.38 | | 2 | | 0.00 | | 0 | | 0.00 | | 0 | | 0.00 | | 0 | | 0.00 | | 0 | | 0.00 | | 0 | | 1 | | | 0 | | 1 | |  |  |  |  |
| O60229 | Kalirin | KALRN | 1.11 | 2 | | 340.0 | | 6.07 | | 0.00 | 0 | | 0.00 | | 0 | | 0.35 | | 2 | | 0.00 | | 0 | | 0.00 | | 0 | | 0.00 | | 0 | | 0.00 | | 0 | | 0.00 | | 0 | | 1 | | | 0 | | 1 | |  |  |  |  |
| Q8NGR3 | Olfactory receptor 1K1 | OR1K1 | 8.54 | 1 | | 34.2 | | 7.93 | | 0.00 | 0 | | 0.00 | | 0 | | 0.22 | | 1 | | 0.00 | | 0 | | 0.00 | | 0 | | 0.00 | | 0 | | 0.00 | | 0 | | 0.00 | | 0 | | 1 | | | 0 | | 1 | |  |  |  |  |
| H0Y5J9 | TRIO and F-actin-binding protein (Fragment) | TRIOBP | 6.75 | 1 | | 34.4 | | 8.91 | | 0.00 | 0 | | 0.00 | | 0 | | 0.22 | | 1 | | 0.00 | | 0 | | 0.00 | | 0 | | 0.00 | | 0 | | 0.00 | | 0 | | 0.00 | | 0 | | 1 | | | 0 | | 1 | |  |  |  |  |
| E9PBC5 | Plasma kallikrein | KLKB1 | 2.33 | 1 | | 57.6 | | 8.35 | | 0.00 | 0 | | 0.00 | | 0 | | 0.22 | | 1 | | 0.00 | | 0 | | 0.00 | | 0 | | 0.00 | | 0 | | 0.00 | | 0 | | 0.00 | | 0 | | 1 | | | 0 | | 1 | |  |  |  |  |
| J3QK89 | Calcium homeostasis endoplasmic reticulum protein | CHERP | 2.27 | 1 | | 104.9 | | 9.19 | | 0.00 | 0 | | 0.00 | | 0 | | 0.22 | | 1 | | 0.00 | | 0 | | 0.00 | | 0 | | 0.00 | | 0 | | 0.00 | | 0 | | 0.00 | | 0 | | 1 | | | 0 | | 1 | |  |  |  |  |
| B2RC85 | Radial spoke head 10 homolog B2 | RSPH10B2 | 1.61 | 1 | | 100.5 | | 7.49 | | 0.00 | 0 | | 0.00 | | 0 | | 0.22 | | 1 | | 0.00 | | 0 | | 0.00 | | 0 | | 0.00 | | 0 | | 0.00 | | 0 | | 0.00 | | 0 | | 1 | | | 0 | | 1 | |  |  |  |  |
| Q8WXA8 | 5-hydroxytryptamine receptor 3C | HTR3C | 4.25 | 1 | | 50.2 | | 5.88 | | 0.00 | 0 | | 0.00 | | 0 | | 0.22 | | 1 | | 0.00 | | 0 | | 0.00 | | 0 | | 0.00 | | 0 | | 0.00 | | 0 | | 0.00 | | 0 | | 1 | | | 0 | | 1 | |  |  |  |  |
| Q9NVU0 | DNA-directed RNA polymerase III subunit RPC5 | POLR3E | 2.26 | 1 | | 79.8 | | 6.47 | | 0.00 | 0 | | 0.00 | | 0 | | 0.22 | | 1 | | 0.00 | | 0 | | 0.00 | | 0 | | 0.00 | | 0 | | 0.00 | | 0 | | 0.00 | | 0 | | 1 | | | 0 | | 1 | |  |  |  |  |
| A0A087X1N8 | Serpin B6 | SERPINB6 | 4.05 | 1 | | 44.8 | | 5.68 | | 0.00 | 0 | | 0.00 | | 0 | | 0.22 | | 1 | | 0.00 | | 0 | | 0.00 | | 0 | | 0.00 | | 0 | | 0.00 | | 0 | | 0.00 | | 0 | | 1 | | | 0 | | 1 | |  |  |  |  |
| Q13451 | Peptidyl-prolyl cis-trans isomerase FKBP5 | FKBP5 | 4.38 | 1 | | 51.2 | | 5.90 | | 0.00 | 0 | | 0.00 | | 0 | | 0.22 | | 1 | | 0.00 | | 0 | | 0.00 | | 0 | | 0.00 | | 0 | | 0.00 | | 0 | | 0.00 | | 0 | | 1 | | | 0 | | 1 | |  |  |  |  |
| H3BUP8 | Exportin-6 (Fragment) | XPO6 | 66.67 | 1 | | 2.5 | | 6.23 | | 0.00 | 0 | | 0.00 | | 0 | | 0.22 | | 1 | | 0.00 | | 0 | | 0.00 | | 0 | | 0.00 | | 0 | | 0.00 | | 0 | | 0.00 | | 0 | | 1 | | | 0 | | 1 | |  |  |  |  |
| Q96F07 | Cytoplasmic FMR1-interacting protein 2 | CYFIP2 | 0.94 | 1 | | 148.3 | | 7.31 | | 0.00 | 0 | | 0.00 | | 0 | | 0.22 | | 1 | | 0.00 | | 0 | | 0.00 | | 0 | | 0.00 | | 0 | | 0.00 | | 0 | | 0.00 | | 0 | | 1 | | | 0 | | 1 | |  |  |  |  |
| Q8IY22 | C-Maf-inducing protein | CMIP | 1.16 | 1 | | 86.3 | | 6.70 | | 0.00 | 0 | | 0.00 | | 0 | | 0.22 | | 1 | | 0.00 | | 0 | | 0.00 | | 0 | | 0.00 | | 0 | | 0.00 | | 0 | | 0.00 | | 0 | | 1 | | | 0 | | 1 | |  |  |  |  |
| P80108 | Phosphatidylinositol-glycan-specific phospholipase D | GPLD1 | 1.07 | 1 | | 92.3 | | 6.37 | | 0.00 | 0 | | 0.00 | | 0 | | 0.22 | | 1 | | 0.00 | | 0 | | 0.00 | | 0 | | 0.00 | | 0 | | 0.00 | | 0 | | 0.00 | | 0 | | 1 | | | 0 | | 1 | |  |  |  |  |
| A0A5F9ZHB4 | Dipeptidase | DPEP3 | 2.14 | 1 | | 56.4 | | 8.75 | | 0.00 | 0 | | 0.00 | | 0 | | 0.22 | | 1 | | 0.00 | | 0 | | 0.00 | | 0 | | 0.00 | | 0 | | 0.00 | | 0 | | 0.00 | | 0 | | 1 | | | 0 | | 1 | |  |  |  |  |
| Q8IWW8 | Hydroxyacid-oxoacid transhydrogenase, mitochondrial | ADHFE1 | 3.64 | 1 | | 50.3 | | 7.77 | | 0.00 | 0 | | 0.00 | | 0 | | 0.22 | | 1 | | 0.00 | | 0 | | 0.00 | | 0 | | 0.00 | | 0 | | 0.00 | | 0 | | 0.00 | | 0 | | 1 | | | 0 | | 1 | |  |  |  |  |
| Q658Y4 | Protein FAM91A1 | FAM91A1 | 3.46 | 1 | | 93.9 | | 6.39 | | 0.00 | 0 | | 0.00 | | 0 | | 0.22 | | 1 | | 0.00 | | 0 | | 0.00 | | 0 | | 0.00 | | 0 | | 0.00 | | 0 | | 0.00 | | 0 | | 1 | | | 0 | | 1 | |  |  |  |  |
| Q5SRH9 | Tetratricopeptide repeat protein 39A | TTC39A | 2.61 | 1 | | 69.7 | | 7.47 | | 0.00 | 0 | | 0.00 | | 0 | | 0.22 | | 1 | | 0.00 | | 0 | | 0.00 | | 0 | | 0.00 | | 0 | | 0.00 | | 0 | | 0.00 | | 0 | | 1 | | | 0 | | 1 | |  |  |  |  |
| I3L0N7 | Serine/arginine repetitive matrix protein 2 (Fragment) | SRRM2 | 17.39 | 1 | | 13.4 | | 6.19 | | 0.00 | 0 | | 0.00 | | 0 | | 0.22 | | 1 | | 0.00 | | 0 | | 0.00 | | 0 | | 0.00 | | 0 | | 0.00 | | 0 | | 0.00 | | 0 | | 1 | | | 0 | | 1 | |  |  |  |  |
| Q9BZI7 | Regulator of nonsense transcripts 3B | UPF3B | 3.93 | 1 | | 57.7 | | 9.48 | | 0.00 | 0 | | 0.00 | | 0 | | 0.22 | | 1 | | 0.00 | | 0 | | 0.00 | | 0 | | 0.00 | | 0 | | 0.00 | | 0 | | 0.00 | | 0 | | 1 | | | 0 | | 1 | |  |  |  |  |
| Q14999 | Cullin-7 | CUL7 | 1.65 | 1 | | 191.0 | | 5.87 | | 0.00 | 0 | | 0.00 | | 0 | | 0.22 | | 1 | | 0.00 | | 0 | | 0.00 | | 0 | | 0.00 | | 0 | | 0.00 | | 0 | | 0.00 | | 0 | | 1 | | | 0 | | 1 | |  |  |  |  |
| E9PIF5 | Phospholipase DDHD2 | DDHD2 | 12.68 | 1 | | 16.7 | | 5.22 | | 0.00 | 0 | | 0.00 | | 0 | | 0.22 | | 1 | | 0.00 | | 0 | | 0.00 | | 0 | | 0.00 | | 0 | | 0.00 | | 0 | | 0.00 | | 0 | | 1 | | | 0 | | 1 | |  |  |  |  |
| Q8N9N5 | Protein BANP | BANP | 1.73 | 1 | | 56.5 | | 5.49 | | 0.00 | 0 | | 0.00 | | 0 | | 0.22 | | 1 | | 0.00 | | 0 | | 0.00 | | 0 | | 0.00 | | 0 | | 0.00 | | 0 | | 0.00 | | 0 | | 1 | | | 0 | | 1 | |  |  |  |  |
| Q96QF7 | Acidic repeat-containing protein | GCNA | 0.72 | 1 | | 76.1 | | 4.60 | | 0.00 | 0 | | 0.00 | | 0 | | 0.22 | | 1 | | 0.00 | | 0 | | 0.00 | | 0 | | 0.00 | | 0 | | 0.00 | | 0 | | 0.00 | | 0 | | 1 | | | 0 | | 1 | |  |  |  |  |
| M0QX63 | Zinc finger protein 547 (Fragment) | ZNF547 | 9.76 | 1 | | 4.8 | | 4.42 | | 0.00 | 0 | | 0.00 | | 0 | | 0.22 | | 1 | | 0.00 | | 0 | | 0.00 | | 0 | | 0.00 | | 0 | | 0.00 | | 0 | | 0.00 | | 0 | | 1 | | | 0 | | 1 | |  |  |  |  |
| A0A0A0MTN6 | Biogenesis of lysosome-related organelles complex 1 subunit 5 | BLOC1S5 | 17.78 | 1 | | 9.8 | | 8.53 | | 0.00 | 0 | | 0.00 | | 0 | | 0.22 | | 1 | | 0.00 | | 0 | | 0.00 | | 0 | | 0.00 | | 0 | | 0.00 | | 0 | | 0.00 | | 0 | | 1 | | | 0 | | 1 | |  |  |  |  |
| Q9Y2M5 | Kelch-like protein 20 | KLHL20 | 1.64 | 1 | | 67.9 | | 6.87 | | 0.00 | 0 | | 0.00 | | 0 | | 0.22 | | 1 | | 0.00 | | 0 | | 0.00 | | 0 | | 0.00 | | 0 | | 0.00 | | 0 | | 0.00 | | 0 | | 1 | | | 0 | | 1 | |  |  |  |  |
| A0A087WX23 | Retrotransposon-derived protein PEG10 | PEG10 | 2.94 | 1 | | 88.2 | | 7.08 | | 0.00 | 0 | | 0.00 | | 0 | | 0.22 | | 1 | | 0.00 | | 0 | | 0.00 | | 0 | | 0.00 | | 0 | | 0.00 | | 0 | | 0.00 | | 0 | | 1 | | | 0 | | 1 | |  |  |  |  |
| Q92953 | Potassium voltage-gated channel subfamily B member 2 | KCNB2 | 1.65 | 1 | | 102.5 | | 6.14 | | 0.00 | 0 | | 0.00 | | 0 | | 0.22 | | 1 | | 0.00 | | 0 | | 0.00 | | 0 | | 0.00 | | 0 | | 0.00 | | 0 | | 0.00 | | 0 | | 1 | | | 0 | | 1 | |  |  |  |  |
| Q13324 | Corticotropin-releasing factor receptor 2 | CRHR2 | 5.35 | 1 | | 47.7 | | 7.72 | | 0.00 | 0 | | 0.00 | | 0 | | 0.22 | | 1 | | 0.00 | | 0 | | 0.00 | | 0 | | 0.00 | | 0 | | 0.00 | | 0 | | 0.00 | | 0 | | 1 | | | 0 | | 1 | |  |  |  |  |
| Q9NQ38 | Serine protease inhibitor Kazal-type 5 | SPINK5 | 1.50 | 1 | | 120.6 | | 8.06 | | 0.00 | 0 | | 0.00 | | 0 | | 0.22 | | 1 | | 0.00 | | 0 | | 0.00 | | 0 | | 0.00 | | 0 | | 0.00 | | 0 | | 0.00 | | 0 | | 1 | | | 0 | | 1 | |  |  |  |  |
| E5RG85 | Protein CBFA2T1 (Fragment) | RUNX1T1 | 2.34 | 1 | | 18.3 | | 9.99 | | 0.00 | 0 | | 0.00 | | 0 | | 0.22 | | 1 | | 0.00 | | 0 | | 0.00 | | 0 | | 0.00 | | 0 | | 0.00 | | 0 | | 0.00 | | 0 | | 1 | | | 0 | | 1 | |  |  |  |  |
| Q14324 | Myosin-binding protein C, fast-type | MYBPC2 | 1.14 | 1 | | 128.0 | | 7.52 | | 0.00 | 0 | | 0.00 | | 0 | | 0.22 | | 1 | | 0.00 | | 0 | | 0.00 | | 0 | | 0.00 | | 0 | | 0.00 | | 0 | | 0.00 | | 0 | | 1 | | | 0 | | 1 | |  |  |  |  |
| Q3ZLR7 | Transcription factor SPT20 homolog-like 1 | SUPT20HL1 | 3.40 | 1 | | 89.4 | | 8.78 | | 0.00 | 0 | | 0.00 | | 0 | | 0.22 | | 1 | | 0.00 | | 0 | | 0.00 | | 0 | | 0.00 | | 0 | | 0.00 | | 0 | | 0.00 | | 0 | | 1 | | | 0 | | 1 | |  |  |  |  |
| Q8TAE8 | Growth arrest and DNA damage-inducible proteins-interacting protein 1 | GADD45GIP1 | 10.36 | 1 | | 25.4 | | 10.02 | | 0.00 | 0 | | 0.00 | | 0 | | 0.22 | | 1 | | 0.00 | | 0 | | 0.00 | | 0 | | 0.00 | | 0 | | 0.00 | | 0 | | 0.00 | | 0 | | 1 | | | 0 | | 1 | |  |  |  |  |
| P52789 | Hexokinase-2 | HK2 | 2.18 | 1 | | 102.3 | | 6.05 | | 0.00 | 0 | | 0.00 | | 0 | | 0.22 | | 1 | | 0.00 | | 0 | | 0.00 | | 0 | | 0.00 | | 0 | | 0.00 | | 0 | | 0.00 | | 0 | | 1 | | | 0 | | 1 | |  |  |  |  |
| H3BSC9 | Protein ERGIC-53-like (Fragment) | LMAN1L | 22.02 | 1 | | 11.7 | | 7.27 | | 0.00 | 0 | | 0.00 | | 0 | | 0.22 | | 1 | | 0.00 | | 0 | | 0.00 | | 0 | | 0.00 | | 0 | | 0.00 | | 0 | | 0.00 | | 0 | | 1 | | | 0 | | 1 | |  |  |  |  |
| A0A3B3ISW9 | Phosphoinositide phospholipase C | PLCZ1 | 2.62 | 1 | | 75.3 | | 6.61 | | 0.00 | 0 | | 0.00 | | 0 | | 0.22 | | 1 | | 0.00 | | 0 | | 0.00 | | 0 | | 0.00 | | 0 | | 0.00 | | 0 | | 0.00 | | 0 | | 1 | | | 0 | | 1 | |  |  |  |  |
| Q8NGY0 | Olfactory receptor 10X1 | OR10X1 | 9.82 | 1 | | 36.4 | | 8.15 | | 0.00 | 0 | | 0.00 | | 0 | | 0.22 | | 1 | | 0.00 | | 0 | | 0.00 | | 0 | | 0.00 | | 0 | | 0.00 | | 0 | | 0.00 | | 0 | | 1 | | | 0 | | 1 | |  |  |  |  |
| P78539 | Sushi repeat-containing protein SRPX | SRPX | 4.53 | 1 | | 51.5 | | 8.66 | | 0.00 | 0 | | 0.00 | | 0 | | 0.22 | | 1 | | 0.00 | | 0 | | 0.00 | | 0 | | 0.00 | | 0 | | 0.00 | | 0 | | 0.00 | | 0 | | 1 | | | 0 | | 1 | |  |  |  |  |
| Q96KN7 | X-linked retinitis pigmentosa GTPase regulator-interacting protein 1 | RPGRIP1 | 0.54 | 1 | | 146.6 | | 5.68 | | 0.00 | 0 | | 0.00 | | 0 | | 0.22 | | 1 | | 0.00 | | 0 | | 0.00 | | 0 | | 0.00 | | 0 | | 0.00 | | 0 | | 0.00 | | 0 | | 1 | | | 0 | | 1 | |  |  |  |  |
| Q9P0B6 | Coiled-coil domain-containing protein 167 | CCDC167 | 7.22 | 1 | | 11.5 | | 9.50 | | 0.00 | 0 | | 0.00 | | 0 | | 0.22 | | 1 | | 0.00 | | 0 | | 0.00 | | 0 | | 0.00 | | 0 | | 0.00 | | 0 | | 0.00 | | 0 | | 1 | | | 0 | | 1 | |  |  |  |  |
| Q9UKT8 | F-box/WD repeat-containing protein 2 | FBXW2 | 4.85 | 1 | | 51.5 | | 6.58 | | 0.00 | 0 | | 0.00 | | 0 | | 0.22 | | 1 | | 0.00 | | 0 | | 0.00 | | 0 | | 0.00 | | 0 | | 0.00 | | 0 | | 0.00 | | 0 | | 1 | | | 0 | | 1 | |  |  |  |  |
| Q86UW1 | Organic solute transporter subunit alpha | SLC51A | 7.35 | 1 | | 37.7 | | 8.54 | | 0.00 | 0 | | 0.00 | | 0 | | 0.22 | | 1 | | 0.00 | | 0 | | 0.00 | | 0 | | 0.00 | | 0 | | 0.00 | | 0 | | 0.00 | | 0 | | 1 | | | 0 | | 1 | |  |  |  |  |
| Q5VZN3 | Zinc finger protein 483 | ZNF483 | 8.99 | 1 | | 20.8 | | 5.00 | | 0.00 | 0 | | 0.00 | | 0 | | 0.19 | | 1 | | 0.00 | | 0 | | 0.00 | | 0 | | 0.00 | | 0 | | 0.00 | | 0 | | 0.00 | | 0 | | 1 | | | 0 | | 1 | |  |  |  |  |
| Q9Y471 | Inactive cytidine monophosphate-N-acetylneuraminic acid hydroxylase | CMAHP | 5.79 | 1 | | 58.3 | | 6.39 | | 0.00 | 0 | | 0.00 | | 0 | | 0.19 | | 1 | | 0.00 | | 0 | | 0.00 | | 0 | | 0.00 | | 0 | | 0.00 | | 0 | | 0.00 | | 0 | | 1 | | | 0 | | 1 | |  |  |  |  |
| Q8N4L8 | Coiled-coil domain-containing protein 24 | CCDC24 | 7.82 | 1 | | 34.3 | | 7.02 | | 0.00 | 0 | | 0.00 | | 0 | | 0.19 | | 1 | | 0.00 | | 0 | | 0.00 | | 0 | | 0.00 | | 0 | | 0.00 | | 0 | | 0.00 | | 0 | | 1 | | | 0 | | 1 | |  |  |  |  |
| Q7Z4H8 | Protein O-glucosyltransferase 3 | POGLUT3 | 2.96 | 1 | | 58.5 | | 8.24 | | 0.00 | 0 | | 0.00 | | 0 | | 0.19 | | 1 | | 0.00 | | 0 | | 0.00 | | 0 | | 0.00 | | 0 | | 0.00 | | 0 | | 0.00 | | 0 | | 1 | | | 0 | | 1 | |  |  |  |  |
| Q14833 | Metabotropic glutamate receptor 4 | GRM4 | 2.41 | 1 | | 101.8 | | 8.82 | | 0.00 | 0 | | 0.00 | | 0 | | 0.19 | | 1 | | 0.00 | | 0 | | 0.00 | | 0 | | 0.00 | | 0 | | 0.00 | | 0 | | 0.00 | | 0 | | 1 | | | 0 | | 1 | |  |  |  |  |
| Q8TDM6 | Disks large homolog 5 | DLG5 | 1.35 | 1 | | 213.7 | | 7.42 | | 0.00 | 0 | | 0.00 | | 0 | | 0.19 | | 1 | | 0.00 | | 0 | | 0.00 | | 0 | | 0.00 | | 0 | | 0.00 | | 0 | | 0.00 | | 0 | | 1 | | | 0 | | 1 | |  |  |  |  |
| P98161 | Polycystin-1 | PKD1 | 0.28 | 1 | | 462.2 | | 6.73 | | 0.00 | 0 | | 0.00 | | 0 | | 0.19 | | 1 | | 0.00 | | 0 | | 0.00 | | 0 | | 0.00 | | 0 | | 0.00 | | 0 | | 0.00 | | 0 | | 1 | | | 0 | | 1 | |  |  |  |  |
| Q9H0M0 | NEDD4-like E3 ubiquitin-protein ligase WWP1 | WWP1 | 2.06 | 1 | | 105.1 | | 5.90 | | 0.00 | 0 | | 0.00 | | 0 | | 0.19 | | 1 | | 0.00 | | 0 | | 0.00 | | 0 | | 0.00 | | 0 | | 0.00 | | 0 | | 0.00 | | 0 | | 1 | | | 0 | | 1 | |  |  |  |  |
| O15438 | Canalicular multispecific organic anion transporter 2 | ABCC3 | 1.57 | 1 | | 169.2 | | 7.20 | | 0.00 | 0 | | 0.00 | | 0 | | 0.19 | | 1 | | 0.00 | | 0 | | 0.00 | | 0 | | 0.00 | | 0 | | 0.00 | | 0 | | 0.00 | | 0 | | 1 | | | 0 | | 1 | |  |  |  |  |
| Q9NVV4 | Poly(A) RNA polymerase, mitochondrial | MTPAP | 2.06 | 1 | | 66.1 | | 9.04 | | 0.00 | 0 | | 0.00 | | 0 | | 0.19 | | 1 | | 0.00 | | 0 | | 0.00 | | 0 | | 0.00 | | 0 | | 0.00 | | 0 | | 0.00 | | 0 | | 1 | | | 0 | | 1 | |  |  |  |  |
| Q8N1G4 | Leucine-rich repeat-containing protein 47 | LRRC47 | 3.26 | 1 | | 63.4 | | 8.28 | | 0.00 | 0 | | 0.00 | | 0 | | 0.19 | | 1 | | 0.00 | | 0 | | 0.00 | | 0 | | 0.00 | | 0 | | 0.00 | | 0 | | 0.00 | | 0 | | 1 | | | 0 | | 1 | |  |  |  |  |
| Q9UPW6 | DNA-binding protein SATB2 | SATB2 | 3.41 | 1 | | 82.5 | | 6.90 | | 0.00 | 0 | | 0.00 | | 0 | | 0.19 | | 1 | | 0.00 | | 0 | | 0.00 | | 0 | | 0.00 | | 0 | | 0.00 | | 0 | | 0.00 | | 0 | | 1 | | | 0 | | 1 | |  |  |  |  |
| K7EKB9 | Transcription factor E2-alpha (Fragment) | TCF3 | 6.70 | 1 | | 20.1 | | 6.09 | | 0.00 | 0 | | 0.00 | | 0 | | 0.19 | | 1 | | 0.00 | | 0 | | 0.00 | | 0 | | 0.00 | | 0 | | 0.00 | | 0 | | 0.00 | | 0 | | 1 | | | 0 | | 1 | |  |  |  |  |
| Q6UXY8 | Transmembrane channel-like protein 5 | TMC5 | 1.89 | 1 | | 114.7 | | 8.21 | | 0.00 | 0 | | 0.00 | | 0 | | 0.19 | | 1 | | 0.00 | | 0 | | 0.00 | | 0 | | 0.00 | | 0 | | 0.00 | | 0 | | 0.00 | | 0 | | 1 | | | 0 | | 1 | |  |  |  |  |
| F8WD09 | GPN-loop GTPase | GPN1 | 47.95 | 1 | | 7.5 | | 6.49 | | 0.00 | 0 | | 0.00 | | 0 | | 0.19 | | 1 | | 0.00 | | 0 | | 0.00 | | 0 | | 0.00 | | 0 | | 0.00 | | 0 | | 0.00 | | 0 | | 1 | | | 0 | | 1 | |  |  |  |  |
| Q9UQR1 | Zinc finger protein 148 | ZNF148 | 1.64 | 1 | | 88.9 | | 6.48 | | 0.00 | 0 | | 0.00 | | 0 | | 0.19 | | 1 | | 0.00 | | 0 | | 0.00 | | 0 | | 0.00 | | 0 | | 0.00 | | 0 | | 0.00 | | 0 | | 1 | | | 0 | | 1 | |  |  |  |  |
| H3BT02 | Integrator complex subunit 14 (Fragment) | INTS14 | 38.46 | 1 | | 4.3 | | 4.53 | | 0.00 | 0 | | 0.00 | | 0 | | 0.19 | | 1 | | 0.00 | | 0 | | 0.00 | | 0 | | 0.00 | | 0 | | 0.00 | | 0 | | 0.00 | | 0 | | 1 | | | 0 | | 1 | |  |  |  |  |
| Q9BYB0 | SH3 and multiple ankyrin repeat domains protein 3 | SHANK3 | 2.08 | 1 | | 184.6 | | 8.88 | | 0.00 | 0 | | 0.00 | | 0 | | 0.19 | | 1 | | 0.00 | | 0 | | 0.00 | | 0 | | 0.00 | | 0 | | 0.00 | | 0 | | 0.00 | | 0 | | 1 | | | 0 | | 1 | |  |  |  |  |
| A0A2R8YFA9 | [F-actin]-monooxygenase MICAL2 | MICAL2 | 2.25 | 1 | | 218.9 | | 8.34 | | 0.00 | 0 | | 0.00 | | 0 | | 0.19 | | 1 | | 0.00 | | 0 | | 0.00 | | 0 | | 0.00 | | 0 | | 0.00 | | 0 | | 0.00 | | 0 | | 1 | | | 0 | | 1 | |  |  |  |  |
| Q5VV63 | Attractin-like protein 1 | ATRNL1 | 1.74 | 1 | | 152.5 | | 7.31 | | 0.00 | 0 | | 0.00 | | 0 | | 0.19 | | 1 | | 0.00 | | 0 | | 0.00 | | 0 | | 0.00 | | 0 | | 0.00 | | 0 | | 0.00 | | 0 | | 1 | | | 0 | | 1 | |  |  |  |  |
| P48145 | Neuropeptides B/W receptor type 1 | NPBWR1 | 8.54 | 1 | | 36.1 | | 8.73 | | 0.00 | 0 | | 0.00 | | 0 | | 0.19 | | 1 | | 0.00 | | 0 | | 0.00 | | 0 | | 0.00 | | 0 | | 0.00 | | 0 | | 0.00 | | 0 | | 1 | | | 0 | | 1 | |  |  |  |  |
| Q71RC2 | La-related protein 4 | LARP4 | 0.69 | 1 | | 80.5 | | 6.61 | | 0.00 | 0 | | 0.00 | | 0 | | 0.19 | | 1 | | 0.00 | | 0 | | 0.00 | | 0 | | 0.00 | | 0 | | 0.00 | | 0 | | 0.00 | | 0 | | 1 | | | 0 | | 1 | |  |  |  |  |
| Q15274 | Nicotinate-nucleotide pyrophosphorylase [carboxylating] | QPRT | 17.17 | 1 | | 30.8 | | 6.21 | | 0.00 | 0 | | 0.00 | | 0 | | 0.19 | | 1 | | 0.00 | | 0 | | 0.00 | | 0 | | 0.00 | | 0 | | 0.00 | | 0 | | 0.00 | | 0 | | 1 | | | 0 | | 1 | |  |  |  |  |
| K7ER97 | E3 SUMO-protein ligase PIAS2 (Fragment) | PIAS2 | 14.77 | 1 | | 9.8 | | 10.62 | | 0.00 | 0 | | 0.00 | | 0 | | 0.19 | | 1 | | 0.00 | | 0 | | 0.00 | | 0 | | 0.00 | | 0 | | 0.00 | | 0 | | 0.00 | | 0 | | 1 | | | 0 | | 1 | |  |  |  |  |
| P53794 | Sodium/myo-inositol cotransporter | SLC5A3 | 1.11 | 1 | | 79.6 | | 7.27 | | 0.00 | 0 | | 0.00 | | 0 | | 0.19 | | 1 | | 0.00 | | 0 | | 0.00 | | 0 | | 0.00 | | 0 | | 0.00 | | 0 | | 0.00 | | 0 | | 1 | | | 0 | | 1 | |  |  |  |  |
| Q9NXF1 | Testis-expressed protein 10 | TEX10 | 3.66 | 1 | | 105.6 | | 9.36 | | 0.00 | 0 | | 0.00 | | 0 | | 0.19 | | 1 | | 0.00 | | 0 | | 0.00 | | 0 | | 0.00 | | 0 | | 0.00 | | 0 | | 0.00 | | 0 | | 1 | | | 0 | | 1 | |  |  |  |  |
| O14494 | Phospholipid phosphatase 1 | PLPP1 | 10.21 | 1 | | 32.1 | | 7.97 | | 0.00 | 0 | | 0.00 | | 0 | | 0.19 | | 1 | | 0.00 | | 0 | | 0.00 | | 0 | | 0.00 | | 0 | | 0.00 | | 0 | | 0.00 | | 0 | | 1 | | | 0 | | 1 | |  |  |  |  |
| H3BUX4 | Mitochondrial Rho GTPase 2 | RHOT2 | 22.96 | 1 | | 14.9 | | 4.89 | | 0.00 | 0 | | 0.00 | | 0 | | 0.19 | | 1 | | 0.00 | | 0 | | 0.00 | | 0 | | 0.00 | | 0 | | 0.00 | | 0 | | 0.00 | | 0 | | 1 | | | 0 | | 1 | |  |  |  |  |
| O94967 | WD repeat-containing protein 47 | WDR47 | 0.76 | 1 | | 101.9 | | 5.94 | | 0.00 | 0 | | 0.00 | | 0 | | 0.19 | | 1 | | 0.00 | | 0 | | 0.00 | | 0 | | 0.00 | | 0 | | 0.00 | | 0 | | 0.00 | | 0 | | 1 | | | 0 | | 1 | |  |  |  |  |
| A2A2Z9 | Ankyrin repeat domain-containing protein 18B | ANKRD18B | 1.09 | 1 | | 118.2 | | 8.15 | | 0.00 | 0 | | 0.00 | | 0 | | 0.19 | | 1 | | 0.00 | | 0 | | 0.00 | | 0 | | 0.00 | | 0 | | 0.00 | | 0 | | 0.00 | | 0 | | 1 | | | 0 | | 1 | |  |  |  |  |
| Q86YQ8 | Copine-8 | CPNE8 | 4.08 | 1 | | 63.1 | | 5.96 | | 0.00 | 0 | | 0.00 | | 0 | | 0.19 | | 1 | | 0.00 | | 0 | | 0.00 | | 0 | | 0.00 | | 0 | | 0.00 | | 0 | | 0.00 | | 0 | | 1 | | | 0 | | 1 | |  |  |  |  |
| Q8N4C7 | Syntaxin-19 | STX19 | 5.10 | 1 | | 34.3 | | 7.39 | | 0.00 | 0 | | 0.00 | | 0 | | 0.19 | | 1 | | 0.00 | | 0 | | 0.00 | | 0 | | 0.00 | | 0 | | 0.00 | | 0 | | 0.00 | | 0 | | 1 | | | 0 | | 1 | |  |  |  |  |
| Q8TCY5 | Melanocortin-2 receptor accessory protein | MRAP | 19.77 | 1 | | 19.1 | | 7.88 | | 0.00 | 0 | | 0.00 | | 0 | | 0.19 | | 1 | | 0.00 | | 0 | | 0.00 | | 0 | | 0.00 | | 0 | | 0.00 | | 0 | | 0.00 | | 0 | | 1 | | | 0 | | 1 | |  |  |  |  |
| K7EII4 | Ubiquitin carboxyl-terminal hydrolase 32 (Fragment) | USP32 | 9.76 | 1 | | 28.3 | | 7.23 | | 0.00 | 0 | | 0.00 | | 0 | | 0.19 | | 1 | | 0.00 | | 0 | | 0.00 | | 0 | | 0.00 | | 0 | | 0.00 | | 0 | | 0.00 | | 0 | | 1 | | | 0 | | 1 | |  |  |  |  |
| Q96JM7 | Lethal(3)maliant brain tumor-like protein 3 | L3MBTL3 | 2.82 | 1 | | 88.3 | | 6.46 | | 0.00 | 0 | | 0.00 | | 0 | | 0.19 | | 1 | | 0.00 | | 0 | | 0.00 | | 0 | | 0.00 | | 0 | | 0.00 | | 0 | | 0.00 | | 0 | | 1 | | | 0 | | 1 | |  |  |  |  |
| O00429 | Dynamin-1-like protein | DNM1L | 2.58 | 1 | | 81.8 | | 6.81 | | 0.00 | 0 | | 0.00 | | 0 | | 0.19 | | 1 | | 0.00 | | 0 | | 0.00 | | 0 | | 0.00 | | 0 | | 0.00 | | 0 | | 0.00 | | 0 | | 1 | | | 0 | | 1 | |  |  |  |  |
| Q8N807 | Protein disulfide-isomerase-like protein of the testis | PDILT | 2.57 | 1 | | 66.6 | | 6.86 | | 0.00 | 0 | | 0.00 | | 0 | | 0.19 | | 1 | | 0.00 | | 0 | | 0.00 | | 0 | | 0.00 | | 0 | | 0.00 | | 0 | | 0.00 | | 0 | | 1 | | | 0 | | 1 | |  |  |  |  |
| Q8TC21 | Zinc finger protein 596 | ZNF596 | 1.79 | 1 | | 58.5 | | 8.92 | | 0.00 | 0 | | 0.00 | | 0 | | 0.19 | | 1 | | 0.00 | | 0 | | 0.00 | | 0 | | 0.00 | | 0 | | 0.00 | | 0 | | 0.00 | | 0 | | 1 | | | 0 | | 1 | |  |  |  |  |
| O75208 | Ubiquinone biosynthesis protein COQ9, mitochondrial | COQ9 | 12.89 | 1 | | 35.5 | | 5.94 | | 0.00 | 0 | | 0.00 | | 0 | | 0.19 | | 1 | | 0.00 | | 0 | | 0.00 | | 0 | | 0.00 | | 0 | | 0.00 | | 0 | | 0.00 | | 0 | | 1 | | | 0 | | 1 | |  |  |  |  |
| A0A1B0GWG4 | Serine-rich and transmembrane domain-containing 2 | SERTM2 | 17.78 | 1 | | 10.2 | | 6.51 | | 0.00 | 0 | | 0.00 | | 0 | | 0.19 | | 1 | | 0.00 | | 0 | | 0.00 | | 0 | | 0.00 | | 0 | | 0.00 | | 0 | | 0.00 | | 0 | | 1 | | | 0 | | 1 | |  |  |  |  |
| Q8IWZ8 | SURP and G-patch domain-containing protein 1 | SUGP1 | 4.34 | 1 | | 72.4 | | 7.61 | | 0.00 | 0 | | 0.00 | | 0 | | 0.19 | | 1 | | 0.00 | | 0 | | 0.00 | | 0 | | 0.00 | | 0 | | 0.00 | | 0 | | 0.00 | | 0 | | 1 | | | 0 | | 1 | |  |  |  |  |
| Q92922 | SWI/SNF complex subunit SMARCC1 | SMARCC1 | 1.27 | 1 | | 122.8 | | 5.76 | | 0.00 | 0 | | 0.00 | | 0 | | 0.19 | | 1 | | 0.00 | | 0 | | 0.00 | | 0 | | 0.00 | | 0 | | 0.00 | | 0 | | 0.00 | | 0 | | 1 | | | 0 | | 1 | |  |  |  |  |
| O43298 | Zinc finger and BTB domain-containing protein 43 | ZBTB43 | 3.21 | 1 | | 52.6 | | 5.76 | | 0.00 | 0 | | 0.00 | | 0 | | 0.19 | | 1 | | 0.00 | | 0 | | 0.00 | | 0 | | 0.00 | | 0 | | 0.00 | | 0 | | 0.00 | | 0 | | 1 | | | 0 | | 1 | |  |  |  |  |
| Q9BV94 | ER degradation-enhancing alpha-mannosidase-like protein 2 | EDEM2 | 7.27 | 1 | | 64.7 | | 5.31 | | 0.00 | 0 | | 0.00 | | 0 | | 0.19 | | 1 | | 0.00 | | 0 | | 0.00 | | 0 | | 0.00 | | 0 | | 0.00 | | 0 | | 0.00 | | 0 | | 1 | | | 0 | | 1 | |  |  |  |  |
| J3KR12 | Uncharacterized protein |  | 5.01 | 1 | | 36.7 | | 11.37 | | 0.00 | 0 | | 0.00 | | 0 | | 0.19 | | 1 | | 0.00 | | 0 | | 0.00 | | 0 | | 0.00 | | 0 | | 0.00 | | 0 | | 0.00 | | 0 | | 1 | | | 0 | | 1 | |  |  |  |  |
| Q9NZ08 | Endoplasmic reticulum aminopeptidase 1 | ERAP1 | 3.51 | 1 | | 107.2 | | 6.46 | | 0.00 | 0 | | 0.00 | | 0 | | 0.19 | | 1 | | 0.00 | | 0 | | 0.00 | | 0 | | 0.00 | | 0 | | 0.00 | | 0 | | 0.00 | | 0 | | 1 | | | 0 | | 1 | |  |  |  |  |
| Q3T8J9 | GON-4-like protein | GON4L | 0.62 | 1 | | 248.5 | | 5.01 | | 0.00 | 0 | | 0.00 | | 0 | | 0.19 | | 1 | | 0.00 | | 0 | | 0.00 | | 0 | | 0.00 | | 0 | | 0.00 | | 0 | | 0.00 | | 0 | | 1 | | | 0 | | 1 | |  |  |  |  |
| Q9ULL0 | Acrosomal protein KIAA1210 | KIAA1210 | 1.64 | 1 | | 186.9 | | 8.51 | | 0.00 | 0 | | 0.00 | | 0 | | 0.19 | | 1 | | 0.00 | | 0 | | 0.00 | | 0 | | 0.00 | | 0 | | 0.00 | | 0 | | 0.00 | | 0 | | 1 | | | 0 | | 1 | |  |  |  |  |
| F5H2B5 | 5'-3' exonuclease PLD4 | PLD4 | 1.95 | 1 | | 56.3 | | 8.28 | | 0.00 | 0 | | 0.00 | | 0 | | 0.19 | | 1 | | 0.00 | | 0 | | 0.00 | | 0 | | 0.00 | | 0 | | 0.00 | | 0 | | 0.00 | | 0 | | 1 | | | 0 | | 1 | |  |  |  |  |
| Q9BT25 | HAUS augmin-like complex subunit 8 | HAUS8 | 6.10 | 1 | | 44.8 | | 7.06 | | 0.00 | 0 | | 0.00 | | 0 | | 0.19 | | 1 | | 0.00 | | 0 | | 0.00 | | 0 | | 0.00 | | 0 | | 0.00 | | 0 | | 0.00 | | 0 | | 1 | | | 0 | | 1 | |  |  |  |  |
| Q96L73 | Histone-lysine N-methyltransferase, H3 lysine-36 specific | NSD1 | 0.56 | 1 | | 296.5 | | 8.03 | | 0.00 | 0 | | 0.00 | | 0 | | 0.19 | | 1 | | 0.00 | | 0 | | 0.00 | | 0 | | 0.00 | | 0 | | 0.00 | | 0 | | 0.00 | | 0 | | 1 | | | 0 | | 1 | |  |  |  |  |
| A4D1U4 | DENN domain-containing protein 11 | DENND11 | 2.86 | 1 | | 51.4 | | 5.31 | | 0.00 | 0 | | 0.00 | | 0 | | 0.19 | | 1 | | 0.00 | | 0 | | 0.00 | | 0 | | 0.00 | | 0 | | 0.00 | | 0 | | 0.00 | | 0 | | 1 | | | 0 | | 1 | |  |  |  |  |
| Q8IZL2 | Mastermind-like protein 2 | MAML2 | 0.87 | 1 | | 125.1 | | 9.42 | | 0.00 | 0 | | 0.00 | | 0 | | 0.19 | | 1 | | 0.00 | | 0 | | 0.00 | | 0 | | 0.00 | | 0 | | 0.00 | | 0 | | 0.00 | | 0 | | 1 | | | 0 | | 1 | |  |  |  |  |
| O14841 | 5-oxoprolinase | OPLAH | 1.71 | 1 | | 137.4 | | 6.58 | | 0.00 | 0 | | 0.00 | | 0 | | 0.19 | | 1 | | 0.00 | | 0 | | 0.00 | | 0 | | 0.00 | | 0 | | 0.00 | | 0 | | 0.00 | | 0 | | 1 | | | 0 | | 1 | |  |  |  |  |
| H7C588 | E3 ubiquitin-protein ligase makorin-1 (Fragment) | MKRN1 | 20.51 | 1 | | 4.4 | | 10.92 | | 0.00 | 0 | | 0.00 | | 0 | | 0.19 | | 1 | | 0.00 | | 0 | | 0.00 | | 0 | | 0.00 | | 0 | | 0.00 | | 0 | | 0.00 | | 0 | | 1 | | | 0 | | 1 | |  |  |  |  |
| Q5VV67 | Peroxisome proliferator-activated receptor gamma coactivator-related protein 1 | PPRC1 | 1.14 | 1 | | 177.4 | | 6.51 | | 0.00 | 0 | | 0.00 | | 0 | | 0.19 | | 1 | | 0.00 | | 0 | | 0.00 | | 0 | | 0.00 | | 0 | | 0.00 | | 0 | | 0.00 | | 0 | | 1 | | | 0 | | 1 | |  |  |  |  |
| Q4VX62 | Putative uncharacterized protein C6orf99 | C6orf99 | 5.94 | 1 | | 22.8 | | 8.21 | | 0.00 | 0 | | 0.00 | | 0 | | 0.19 | | 1 | | 0.00 | | 0 | | 0.00 | | 0 | | 0.00 | | 0 | | 0.00 | | 0 | | 0.00 | | 0 | | 1 | | | 0 | | 1 | |  |  |  |  |
| Q8NFK1 | Gap junction gamma-3 protein | GJC3 | 5.73 | 1 | | 31.3 | | 9.17 | | 0.00 | 0 | | 0.00 | | 0 | | 0.16 | | 1 | | 0.00 | | 0 | | 0.00 | | 0 | | 0.00 | | 0 | | 0.00 | | 0 | | 0.00 | | 0 | | 1 | | | 0 | | 1 | |  |  |  |  |
| Q8N0Z3 | Spindle and centriole-associated protein 1 | SPICE1 | 2.69 | 1 | | 96.2 | | 7.44 | | 0.00 | 0 | | 0.00 | | 0 | | 0.16 | | 1 | | 0.00 | | 0 | | 0.00 | | 0 | | 0.00 | | 0 | | 0.00 | | 0 | | 0.00 | | 0 | | 1 | | | 0 | | 1 | |  |  |  |  |
| Q8NG76 | Olfactory receptor 2T33 | OR2T33 | 8.13 | 1 | | 35.9 | | 8.57 | | 0.00 | 0 | | 0.00 | | 0 | | 0.16 | | 1 | | 0.00 | | 0 | | 0.00 | | 0 | | 0.00 | | 0 | | 0.00 | | 0 | | 0.00 | | 0 | | 1 | | | 0 | | 1 | |  |  |  |  |
| A0A2R8YF01 | Vacuolar protein sorting-associated protein 45 | VPS45 | 5.53 | 1 | | 50.0 | | 7.24 | | 0.00 | 0 | | 0.00 | | 0 | | 0.16 | | 1 | | 0.00 | | 0 | | 0.00 | | 0 | | 0.00 | | 0 | | 0.00 | | 0 | | 0.00 | | 0 | | 1 | | | 0 | | 1 | |  |  |  |  |
| O60938 | Keratocan | KERA | 5.40 | 1 | | 40.5 | | 7.47 | | 0.00 | 0 | | 0.00 | | 0 | | 0.16 | | 1 | | 0.00 | | 0 | | 0.00 | | 0 | | 0.00 | | 0 | | 0.00 | | 0 | | 0.00 | | 0 | | 1 | | | 0 | | 1 | |  |  |  |  |
| Q9NVH2 | Integrator complex subunit 7 | INTS7 | 1.98 | 1 | | 106.8 | | 8.02 | | 0.00 | 0 | | 0.00 | | 0 | | 0.16 | | 1 | | 0.00 | | 0 | | 0.00 | | 0 | | 0.00 | | 0 | | 0.00 | | 0 | | 0.00 | | 0 | | 1 | | | 0 | | 1 | |  |  |  |  |
| Q5T0Z8 | Uncharacterized protein C6orf132 | C6orf132 | 0.84 | 1 | | 124.0 | | 9.45 | | 0.00 | 0 | | 0.00 | | 0 | | 0.16 | | 1 | | 0.00 | | 0 | | 0.00 | | 0 | | 0.00 | | 0 | | 0.00 | | 0 | | 0.00 | | 0 | | 1 | | | 0 | | 1 | |  |  |  |  |
| P41236 | Protein phosphatase inhibitor 2 | PPP1R2 | 5.37 | 1 | | 23.0 | | 4.74 | | 0.00 | 0 | | 0.00 | | 0 | | 0.16 | | 1 | | 0.00 | | 0 | | 0.00 | | 0 | | 0.00 | | 0 | | 0.00 | | 0 | | 0.00 | | 0 | | 1 | | | 0 | | 1 | |  |  |  |  |
| A6NI87 | Protein chibby homolog 3 | CBY3 | 7.44 | 1 | | 27.3 | | 10.64 | | 0.00 | 0 | | 0.00 | | 0 | | 0.16 | | 1 | | 0.00 | | 0 | | 0.00 | | 0 | | 0.00 | | 0 | | 0.00 | | 0 | | 0.00 | | 0 | | 1 | | | 0 | | 1 | |  |  |  |  |
| Q9UBH6 | Xenotropic and polytropic retrovirus receptor 1 | XPR1 | 3.16 | 1 | | 81.5 | | 8.44 | | 0.00 | 0 | | 0.00 | | 0 | | 0.16 | | 1 | | 0.00 | | 0 | | 0.00 | | 0 | | 0.00 | | 0 | | 0.00 | | 0 | | 0.00 | | 0 | | 1 | | | 0 | | 1 | |  |  |  |  |
| Q9UL01 | Dermatan-sulfate epimerase | DSE | 0.73 | 1 | | 109.7 | | 8.09 | | 0.00 | 0 | | 0.00 | | 0 | | 0.16 | | 1 | | 0.00 | | 0 | | 0.00 | | 0 | | 0.00 | | 0 | | 0.00 | | 0 | | 0.00 | | 0 | | 1 | | | 0 | | 1 | |  |  |  |  |
| Q86Y56 | Dynein assembly factor 5, axonemal | DNAAF5 | 2.69 | 1 | | 93.5 | | 6.42 | | 0.00 | 0 | | 0.00 | | 0 | | 0.16 | | 1 | | 0.00 | | 0 | | 0.00 | | 0 | | 0.00 | | 0 | | 0.00 | | 0 | | 0.00 | | 0 | | 1 | | | 0 | | 1 | |  |  |  |  |
| H9KV53 | Cytosolic carboxypeptidase 2 | AGBL2 | 3.43 | 1 | | 104.4 | | 9.03 | | 0.00 | 0 | | 0.00 | | 0 | | 0.16 | | 1 | | 0.00 | | 0 | | 0.00 | | 0 | | 0.00 | | 0 | | 0.00 | | 0 | | 0.00 | | 0 | | 1 | | | 0 | | 1 | |  |  |  |  |
| Q13268 | Dehydrogenase/reductase SDR family member 2, mitochondrial | DHRS2 | 8.21 | 1 | | 29.9 | | 9.01 | | 0.00 | 0 | | 0.00 | | 0 | | 0.16 | | 1 | | 0.00 | | 0 | | 0.00 | | 0 | | 0.00 | | 0 | | 0.00 | | 0 | | 0.00 | | 0 | | 1 | | | 0 | | 1 | |  |  |  |  |
| Q2LD37 | Transmembrane protein KIAA1109 | KIAA1109 | 0.44 | 1 | | 555.1 | | 6.58 | | 0.00 | 0 | | 0.00 | | 0 | | 0.16 | | 1 | | 0.00 | | 0 | | 0.00 | | 0 | | 0.00 | | 0 | | 0.00 | | 0 | | 0.00 | | 0 | | 1 | | | 0 | | 1 | |  |  |  |  |
| Q8WUP2 | Filamin-binding LIM protein 1 | FBLIM1 | 2.14 | 1 | | 40.6 | | 6.02 | | 0.00 | 0 | | 0.00 | | 0 | | 0.16 | | 1 | | 0.00 | | 0 | | 0.00 | | 0 | | 0.00 | | 0 | | 0.00 | | 0 | | 0.00 | | 0 | | 1 | | | 0 | | 1 | |  |  |  |  |
| Q9BZQ2 | Testicular spindle-associated protein SHCBP1L | SHCBP1L | 6.28 | 1 | | 72.6 | | 5.31 | | 0.00 | 0 | | 0.00 | | 0 | | 0.16 | | 1 | | 0.00 | | 0 | | 0.00 | | 0 | | 0.00 | | 0 | | 0.00 | | 0 | | 0.00 | | 0 | | 1 | | | 0 | | 1 | |  |  |  |  |
| Q8WXG6 | MAP kinase-activating death domain protein | MADD | 0.55 | 1 | | 183.2 | | 6.04 | | 0.00 | 0 | | 0.00 | | 0 | | 0.16 | | 1 | | 0.00 | | 0 | | 0.00 | | 0 | | 0.00 | | 0 | | 0.00 | | 0 | | 0.00 | | 0 | | 1 | | | 0 | | 1 | |  |  |  |  |
| Q9Y2D8 | Afadin- and alpha-actinin-binding protein | SSX2IP | 1.30 | 1 | | 71.2 | | 6.40 | | 0.00 | 0 | | 0.00 | | 0 | | 0.16 | | 1 | | 0.00 | | 0 | | 0.00 | | 0 | | 0.00 | | 0 | | 0.00 | | 0 | | 0.00 | | 0 | | 1 | | | 0 | | 1 | |  |  |  |  |
| P10071 | Transcriptional activator GLI3 | GLI3 | 0.63 | 1 | | 169.8 | | 7.40 | | 0.00 | 0 | | 0.00 | | 0 | | 0.16 | | 1 | | 0.00 | | 0 | | 0.00 | | 0 | | 0.00 | | 0 | | 0.00 | | 0 | | 0.00 | | 0 | | 1 | | | 0 | | 1 | |  |  |  |  |
| Q5VZE5 | N-alpha-acetyltransferase 35, NatC auxiliary subunit | NAA35 | 3.31 | 1 | | 83.6 | | 7.05 | | 0.00 | 0 | | 0.00 | | 0 | | 0.16 | | 1 | | 0.00 | | 0 | | 0.00 | | 0 | | 0.00 | | 0 | | 0.00 | | 0 | | 0.00 | | 0 | | 1 | | | 0 | | 1 | |  |  |  |  |
| Q14766 | Latent-transforming growth factor beta-binding protein 1 | LTBP1 | 0.81 | 1 | | 186.7 | | 5.96 | | 0.00 | 0 | | 0.00 | | 0 | | 0.16 | | 1 | | 0.00 | | 0 | | 0.00 | | 0 | | 0.00 | | 0 | | 0.00 | | 0 | | 0.00 | | 0 | | 1 | | | 0 | | 1 | |  |  |  |  |
| Q9Y6X0 | SET-binding protein | SETBP1 | 0.81 | 1 | | 174.9 | | 9.74 | | 0.00 | 0 | | 0.00 | | 0 | | 0.16 | | 1 | | 0.00 | | 0 | | 0.00 | | 0 | | 0.00 | | 0 | | 0.00 | | 0 | | 0.00 | | 0 | | 1 | | | 0 | | 1 | |  |  |  |  |
| A0A2U3TZM3 | Chromosome 1 open reading frame 19, isoform CRA_a | TSEN15 | 11.43 | 1 | | 19.1 | | 4.54 | | 0.00 | 0 | | 0.00 | | 0 | | 0.16 | | 1 | | 0.00 | | 0 | | 0.00 | | 0 | | 0.00 | | 0 | | 0.00 | | 0 | | 0.00 | | 0 | | 1 | | | 0 | | 1 | |  |  |  |  |
| A0A0A0MR85 | Glutathione S-transferase Mu 4 | GSTM4 | 13.76 | 1 | | 25.5 | | 5.90 | | 0.00 | 0 | | 0.00 | | 0 | | 0.16 | | 1 | | 0.00 | | 0 | | 0.00 | | 0 | | 0.00 | | 0 | | 0.00 | | 0 | | 0.00 | | 0 | | 1 | | | 0 | | 1 | |  |  |  |  |
| A0A590UJL7 | FERM and PDZ domain-containing protein 4 | FRMPD4 | 0.34 | 1 | | 195.2 | | 5.43 | | 0.00 | 0 | | 0.00 | | 0 | | 0.16 | | 1 | | 0.00 | | 0 | | 0.00 | | 0 | | 0.00 | | 0 | | 0.00 | | 0 | | 0.00 | | 0 | | 1 | | | 0 | | 1 | |  |  |  |  |
| A0A087X0Z1 | Arf-GAP with GTPase, ANK repeat and PH domain-containing protein 4 | AGAP4 | 2.77 | 1 | | 75.6 | | 7.12 | | 0.00 | 0 | | 0.00 | | 0 | | 0.16 | | 1 | | 0.00 | | 0 | | 0.00 | | 0 | | 0.00 | | 0 | | 0.00 | | 0 | | 0.00 | | 0 | | 1 | | | 0 | | 1 | |  |  |  |  |
| Q96M27 | Protein PRRC1 | PRRC1 | 2.92 | 1 | | 46.7 | | 5.83 | | 0.00 | 0 | | 0.00 | | 0 | | 0.16 | | 1 | | 0.00 | | 0 | | 0.00 | | 0 | | 0.00 | | 0 | | 0.00 | | 0 | | 0.00 | | 0 | | 1 | | | 0 | | 1 | |  |  |  |  |
| U3KQK5 | Uncharacterized protein |  | 10.37 | 1 | | 17.0 | | 10.80 | | 0.00 | 0 | | 0.00 | | 0 | | 0.16 | | 1 | | 0.00 | | 0 | | 0.00 | | 0 | | 0.00 | | 0 | | 0.00 | | 0 | | 0.00 | | 0 | | 1 | | | 0 | | 1 | |  |  |  |  |
| O43379 | WD repeat-containing protein 62 | WDR62 | 1.05 | 1 | | 165.8 | | 5.91 | | 0.00 | 0 | | 0.00 | | 0 | | 0.16 | | 1 | | 0.00 | | 0 | | 0.00 | | 0 | | 0.00 | | 0 | | 0.00 | | 0 | | 0.00 | | 0 | | 1 | | | 0 | | 1 | |  |  |  |  |
| A0A1B0GUH0 | Oxygen-regulated protein 1 | RP1 | 2.19 | 1 | | 158.4 | | 7.91 | | 0.00 | 0 | | 0.00 | | 0 | | 0.16 | | 1 | | 0.00 | | 0 | | 0.00 | | 0 | | 0.00 | | 0 | | 0.00 | | 0 | | 0.00 | | 0 | | 1 | | | 0 | | 1 | |  |  |  |  |
| A0A5F9ZHW8 | Pleckstrin homology domain-containing family G member 5 | PLEKHG5 | 1.72 | 1 | | 122.3 | | 6.04 | | 0.00 | 0 | | 0.00 | | 0 | | 0.16 | | 1 | | 0.00 | | 0 | | 0.00 | | 0 | | 0.00 | | 0 | | 0.00 | | 0 | | 0.00 | | 0 | | 1 | | | 0 | | 1 | |  |  |  |  |
| Q9NZB8 | Molybdenum cofactor biosynthesis protein 1 | MOCS1 | 1.89 | 1 | | 70.1 | | 9.26 | | 0.00 | 0 | | 0.00 | | 0 | | 0.16 | | 1 | | 0.00 | | 0 | | 0.00 | | 0 | | 0.00 | | 0 | | 0.00 | | 0 | | 0.00 | | 0 | | 1 | | | 0 | | 1 | |  |  |  |  |
| Q8IYD2 | Kelch domain-containing protein 8A | KLHDC8A | 3.14 | 1 | | 38.8 | | 9.26 | | 0.00 | 0 | | 0.00 | | 0 | | 0.16 | | 1 | | 0.00 | | 0 | | 0.00 | | 0 | | 0.00 | | 0 | | 0.00 | | 0 | | 0.00 | | 0 | | 1 | | | 0 | | 1 | |  |  |  |  |
| Q96SE7 | Zinc finger protein 347 | ZNF347 | 1.79 | 1 | | 95.7 | | 9.17 | | 0.00 | 0 | | 0.00 | | 0 | | 0.16 | | 1 | | 0.00 | | 0 | | 0.00 | | 0 | | 0.00 | | 0 | | 0.00 | | 0 | | 0.00 | | 0 | | 1 | | | 0 | | 1 | |  |  |  |  |
| O00300 | Tumor necrosis factor receptor superfamily member 11B | TNFRSF11B | 4.24 | 1 | | 46.0 | | 8.29 | | 0.00 | 0 | | 0.00 | | 0 | | 0.16 | | 1 | | 0.00 | | 0 | | 0.00 | | 0 | | 0.00 | | 0 | | 0.00 | | 0 | | 0.00 | | 0 | | 1 | | | 0 | | 1 | |  |  |  |  |
| P0C7T3 | Olfactory receptor 56A5 | OR56A5 | 12.14 | 1 | | 35.3 | | 8.72 | | 0.00 | 0 | | 0.00 | | 0 | | 0.16 | | 1 | | 0.00 | | 0 | | 0.00 | | 0 | | 0.00 | | 0 | | 0.00 | | 0 | | 0.00 | | 0 | | 1 | | | 0 | | 1 | |  |  |  |  |
| Q8N7B6 | PACRG-like protein | PACRGL | 3.63 | 1 | | 27.1 | | 9.67 | | 0.00 | 0 | | 0.00 | | 0 | | 0.16 | | 1 | | 0.00 | | 0 | | 0.00 | | 0 | | 0.00 | | 0 | | 0.00 | | 0 | | 0.00 | | 0 | | 1 | | | 0 | | 1 | |  |  |  |  |
| H0Y4R2 | NADPH--cytochrome P450 reductase (Fragment) | POR | 2.76 | 1 | | 81.6 | | 6.54 | | 0.00 | 0 | | 0.00 | | 0 | | 0.16 | | 1 | | 0.00 | | 0 | | 0.00 | | 0 | | 0.00 | | 0 | | 0.00 | | 0 | | 0.00 | | 0 | | 1 | | | 0 | | 1 | |  |  |  |  |
| P16157 | Ankyrin-1 | ANK1 | 1.06 | 1 | | 206.1 | | 6.01 | | 0.00 | 0 | | 0.00 | | 0 | | 0.16 | | 1 | | 0.00 | | 0 | | 0.00 | | 0 | | 0.00 | | 0 | | 0.00 | | 0 | | 0.00 | | 0 | | 1 | | | 0 | | 1 | |  |  |  |  |
| Q6ICG6 | Uncharacterized protein KIAA0930 | KIAA0930 | 4.95 | 1 | | 45.8 | | 7.94 | | 0.00 | 0 | | 0.00 | | 0 | | 0.16 | | 1 | | 0.00 | | 0 | | 0.00 | | 0 | | 0.00 | | 0 | | 0.00 | | 0 | | 0.00 | | 0 | | 1 | | | 0 | | 1 | |  |  |  |  |
| O60239 | SH3 domain-binding protein 5 | SH3BP5 | 5.05 | 1 | | 50.4 | | 4.97 | | 0.00 | 0 | | 0.00 | | 0 | | 0.16 | | 1 | | 0.00 | | 0 | | 0.00 | | 0 | | 0.00 | | 0 | | 0.00 | | 0 | | 0.00 | | 0 | | 1 | | | 0 | | 1 | |  |  |  |  |
| Q8N4H0 | Spermatogenesis associated 6-like protein | SPATA6L | 9.44 | 1 | | 45.0 | | 9.13 | | 0.00 | 0 | | 0.00 | | 0 | | 0.16 | | 1 | | 0.00 | | 0 | | 0.00 | | 0 | | 0.00 | | 0 | | 0.00 | | 0 | | 0.00 | | 0 | | 1 | | | 0 | | 1 | |  |  |  |  |
| Q03403 | Trefoil factor 2 | TFF2 | 22.48 | 1 | | 14.3 | | 5.81 | | 0.00 | 0 | | 0.00 | | 0 | | 0.16 | | 1 | | 0.00 | | 0 | | 0.00 | | 0 | | 0.00 | | 0 | | 0.00 | | 0 | | 0.00 | | 0 | | 1 | | | 0 | | 1 | |  |  |  |  |
| K7EN42 | Glycylpeptide N-tetradecanoyltransferase 1 (Fragment) | NMT1 | 47.73 | 1 | | 9.6 | | 6.77 | | 0.00 | 0 | | 0.00 | | 0 | | 0.16 | | 1 | | 0.00 | | 0 | | 0.00 | | 0 | | 0.00 | | 0 | | 0.00 | | 0 | | 0.00 | | 0 | | 1 | | | 0 | | 1 | |  |  |  |  |
| Q6AI14 | Sodium/hydrogen exchanger 4 | SLC9A4 | 1.38 | 1 | | 89.8 | | 8.54 | | 0.00 | 0 | | 0.00 | | 0 | | 0.16 | | 1 | | 0.00 | | 0 | | 0.00 | | 0 | | 0.00 | | 0 | | 0.00 | | 0 | | 0.00 | | 0 | | 1 | | | 0 | | 1 | |  |  |  |  |
| Q96KP1 | Exocyst complex component 2 | EXOC2 | 0.87 | 1 | | 104.0 | | 6.90 | | 0.00 | 0 | | 0.00 | | 0 | | 0.16 | | 1 | | 0.00 | | 0 | | 0.00 | | 0 | | 0.00 | | 0 | | 0.00 | | 0 | | 0.00 | | 0 | | 1 | | | 0 | | 1 | |  |  |  |  |
| A0A0A0MSY7 | Thymidine kinase 2, mitochondrial | TK2 | 5.54 | 1 | | 33.3 | | 9.32 | | 0.00 | 0 | | 0.00 | | 0 | | 0.16 | | 1 | | 0.00 | | 0 | | 0.00 | | 0 | | 0.00 | | 0 | | 0.00 | | 0 | | 0.00 | | 0 | | 1 | | | 0 | | 1 | |  |  |  |  |
| Q14767 | Latent-transforming growth factor beta-binding protein 2 | LTBP2 | 0.71 | 1 | | 194.9 | | 5.19 | | 0.00 | 0 | | 0.00 | | 0 | | 0.16 | | 1 | | 0.00 | | 0 | | 0.00 | | 0 | | 0.00 | | 0 | | 0.00 | | 0 | | 0.00 | | 0 | | 1 | | | 0 | | 1 | |  |  |  |  |
| Q96CN5 | Leucine-rich repeat-containing protein 45 | LRRC45 | 1.79 | 1 | | 75.9 | | 6.23 | | 0.00 | 0 | | 0.00 | | 0 | | 0.16 | | 1 | | 0.00 | | 0 | | 0.00 | | 0 | | 0.00 | | 0 | | 0.00 | | 0 | | 0.00 | | 0 | | 1 | | | 0 | | 1 | |  |  |  |  |
| Q5TC82 | Roquin-1 | RC3H1 | 1.06 | 1 | | 125.7 | | 7.27 | | 0.00 | 0 | | 0.00 | | 0 | | 0.16 | | 1 | | 0.00 | | 0 | | 0.00 | | 0 | | 0.00 | | 0 | | 0.00 | | 0 | | 0.00 | | 0 | | 1 | | | 0 | | 1 | |  |  |  |  |
| Q8NEF3 | Coiled-coil domain-containing protein 112 | CCDC112 | 1.79 | 1 | | 53.5 | | 9.48 | | 0.00 | 0 | | 0.00 | | 0 | | 0.16 | | 1 | | 0.00 | | 0 | | 0.00 | | 0 | | 0.00 | | 0 | | 0.00 | | 0 | | 0.00 | | 0 | | 1 | | | 0 | | 1 | |  |  |  |  |
| Q16520 | Basic leucine zipper transcriptional factor ATF-like | BATF | 11.20 | 1 | | 14.1 | | 8.75 | | 0.00 | 0 | | 0.00 | | 0 | | 0.16 | | 1 | | 0.00 | | 0 | | 0.00 | | 0 | | 0.00 | | 0 | | 0.00 | | 0 | | 0.00 | | 0 | | 1 | | | 0 | | 1 | |  |  |  |  |
| E9PLP8 | Cleavage stimulation factor subunit 3 | CSTF3 | 0.93 | 1 | | 86.2 | | 7.65 | | 0.00 | 0 | | 0.00 | | 0 | | 0.16 | | 1 | | 0.00 | | 0 | | 0.00 | | 0 | | 0.00 | | 0 | | 0.00 | | 0 | | 0.00 | | 0 | | 1 | | | 0 | | 1 | |  |  |  |  |
| Q9UPZ3 | Hermansky-Pudlak syndrome 5 protein | HPS5 | 2.83 | 1 | | 127.4 | | 5.54 | | 0.00 | 0 | | 0.00 | | 0 | | 0.16 | | 1 | | 0.00 | | 0 | | 0.00 | | 0 | | 0.00 | | 0 | | 0.00 | | 0 | | 0.00 | | 0 | | 1 | | | 0 | | 1 | |  |  |  |  |
| A0A2R8Y7A4 | Centrosomal protein of 78 kDa | CEP78 | 0.69 | 1 | | 80.8 | | 8.46 | | 0.00 | 0 | | 0.00 | | 0 | | 0.16 | | 1 | | 0.00 | | 0 | | 0.00 | | 0 | | 0.00 | | 0 | | 0.00 | | 0 | | 0.00 | | 0 | | 1 | | | 0 | | 1 | |  |  |  |  |
| Q99999 | Galactosylceramide sulfotransferase | GAL3ST1 | 7.57 | 1 | | 48.7 | | 8.62 | | 0.00 | 0 | | 0.00 | | 0 | | 0.16 | | 1 | | 0.00 | | 0 | | 0.00 | | 0 | | 0.00 | | 0 | | 0.00 | | 0 | | 0.00 | | 0 | | 1 | | | 0 | | 1 | |  |  |  |  |
| A0A5F9ZHQ6 | EF-hand calcium-binding domain-containing protein 14 | EFCAB14 | 1.52 | 1 | | 58.1 | | 6.58 | | 0.00 | 0 | | 0.00 | | 0 | | 0.16 | | 1 | | 0.00 | | 0 | | 0.00 | | 0 | | 0.00 | | 0 | | 0.00 | | 0 | | 0.00 | | 0 | | 1 | | | 0 | | 1 | |  |  |  |  |
| H0Y911 | Transforming acidic coiled-coil-containing protein 2 (Fragment) | TACC2 | 5.62 | 1 | | 44.9 | | 5.12 | | 0.00 | 0 | | 0.00 | | 0 | | 0.16 | | 1 | | 0.00 | | 0 | | 0.00 | | 0 | | 0.00 | | 0 | | 0.00 | | 0 | | 0.00 | | 0 | | 1 | | | 0 | | 1 | |  |  |  |  |
| E5KLJ9 | Dynamin-like 120 kDa protein, mitochondrial | OPA1 | 2.86 | 1 | | 113.6 | | 7.88 | | 0.00 | 0 | | 0.00 | | 0 | | 0.16 | | 1 | | 0.00 | | 0 | | 0.00 | | 0 | | 0.00 | | 0 | | 0.00 | | 0 | | 0.00 | | 0 | | 1 | | | 0 | | 1 | |  |  |  |  |
| Q9NTK1 | Protein DEPP1 | DEPP1 | 5.66 | 1 | | 23.4 | | 10.67 | | 0.00 | 0 | | 0.00 | | 0 | | 0.16 | | 1 | | 0.00 | | 0 | | 0.00 | | 0 | | 0.00 | | 0 | | 0.00 | | 0 | | 0.00 | | 0 | | 1 | | | 0 | | 1 | |  |  |  |  |
| Q8N7E2 | E3 ubiquitin-protein ligase CBLL2 | CBLL2 | 4.71 | 1 | | 48.8 | | 9.50 | | 0.00 | 0 | | 0.00 | | 0 | | 0.16 | | 1 | | 0.00 | | 0 | | 0.00 | | 0 | | 0.00 | | 0 | | 0.00 | | 0 | | 0.00 | | 0 | | 1 | | | 0 | | 1 | |  |  |  |  |
| Q96ED9 | Protein Hook homolog 2 | HOOK2 | 1.11 | 1 | | 83.2 | | 5.47 | | 0.00 | 0 | | 0.00 | | 0 | | 0.16 | | 1 | | 0.00 | | 0 | | 0.00 | | 0 | | 0.00 | | 0 | | 0.00 | | 0 | | 0.00 | | 0 | | 1 | | | 0 | | 1 | |  |  |  |  |
| A0A1P0AZG4 | Ligand-dependent corepressor | LCOR | 1.92 | 1 | | 137.1 | | 8.24 | | 0.00 | 0 | | 0.00 | | 0 | | 0.16 | | 1 | | 0.00 | | 0 | | 0.00 | | 0 | | 0.00 | | 0 | | 0.00 | | 0 | | 0.00 | | 0 | | 1 | | | 0 | | 1 | |  |  |  |  |
| A5D8W1 | Cilia- and flagella-associated protein 69 | CFAP69 | 2.44 | 1 | | 105.8 | | 7.17 | | 0.00 | 0 | | 0.00 | | 0 | | 0.16 | | 1 | | 0.00 | | 0 | | 0.00 | | 0 | | 0.00 | | 0 | | 0.00 | | 0 | | 0.00 | | 0 | | 1 | | | 0 | | 1 | |  |  |  |  |
| Q9UHD1 | Cysteine and histidine-rich domain-containing protein 1 | CHORDC1 | 4.52 | 1 | | 37.5 | | 7.87 | | 0.00 | 0 | | 0.00 | | 0 | | 0.16 | | 1 | | 0.00 | | 0 | | 0.00 | | 0 | | 0.00 | | 0 | | 0.00 | | 0 | | 0.00 | | 0 | | 1 | | | 0 | | 1 | |  |  |  |  |
| P04198 | N-myc proto-oncogene protein | MYCN | 11.21 | 1 | | 49.5 | | 5.66 | | 0.00 | 0 | | 0.00 | | 0 | | 0.16 | | 1 | | 0.00 | | 0 | | 0.00 | | 0 | | 0.00 | | 0 | | 0.00 | | 0 | | 0.00 | | 0 | | 1 | | | 0 | | 1 | |  |  |  |  |
| Q9H9J4 | Ubiquitin carboxyl-terminal hydrolase 42 | USP42 | 0.83 | 1 | | 145.3 | | 8.63 | | 0.00 | 0 | | 0.00 | | 0 | | 0.16 | | 1 | | 0.00 | | 0 | | 0.00 | | 0 | | 0.00 | | 0 | | 0.00 | | 0 | | 0.00 | | 0 | | 1 | | | 0 | | 1 | |  |  |  |  |
| Q16880 | 2-hydroxyacylsphingosine 1-beta-galactosyltransferase | UGT8 | 3.14 | 1 | | 61.4 | | 9.52 | | 0.00 | 0 | | 0.00 | | 0 | | 0.16 | | 1 | | 0.00 | | 0 | | 0.00 | | 0 | | 0.00 | | 0 | | 0.00 | | 0 | | 0.00 | | 0 | | 1 | | | 0 | | 1 | |  |  |  |  |
| Q7Z2Z2 | Elongation factor-like GTPase 1 | EFL1 | 2.50 | 1 | | 125.4 | | 5.91 | | 0.00 | 0 | | 0.00 | | 0 | | 0.16 | | 1 | | 0.00 | | 0 | | 0.00 | | 0 | | 0.00 | | 0 | | 0.00 | | 0 | | 0.00 | | 0 | | 1 | | | 0 | | 1 | |  |  |  |  |
| Q6GQQ9 | OTU domain-containing protein 7B | OTUD7B | 2.25 | 1 | | 92.5 | | 6.71 | | 0.00 | 0 | | 0.00 | | 0 | | 0.16 | | 1 | | 0.00 | | 0 | | 0.00 | | 0 | | 0.00 | | 0 | | 0.00 | | 0 | | 0.00 | | 0 | | 1 | | | 0 | | 1 | |  |  |  |  |
| P38935 | DNA-binding protein SMUBP-2 | IGHMBP2 | 0.91 | 1 | | 109.1 | | 8.97 | | 0.00 | 0 | | 0.00 | | 0 | | 0.16 | | 1 | | 0.00 | | 0 | | 0.00 | | 0 | | 0.00 | | 0 | | 0.00 | | 0 | | 0.00 | | 0 | | 1 | | | 0 | | 1 | |  |  |  |  |
| B4DX56 | Doublesex- and mab-3-related transcription factor C2 | DMRTC2 | 2.39 | 1 | | 44.7 | | 8.63 | | 0.00 | 0 | | 0.00 | | 0 | | 0.16 | | 1 | | 0.00 | | 0 | | 0.00 | | 0 | | 0.00 | | 0 | | 0.00 | | 0 | | 0.00 | | 0 | | 1 | | | 0 | | 1 | |  |  |  |  |
| Q5JUK9 | P antigen family member 3 | PAGE3 | 18.58 | 1 | | 12.5 | | 4.70 | | 0.00 | 0 | | 0.00 | | 0 | | 0.16 | | 1 | | 0.00 | | 0 | | 0.00 | | 0 | | 0.00 | | 0 | | 0.00 | | 0 | | 0.00 | | 0 | | 1 | | | 0 | | 1 | |  |  |  |  |
| P28331 | NADH-ubiquinone oxidoreductase 75 kDa subunit, mitochondrial | NDUFS1 | 1.10 | 1 | | 79.4 | | 6.23 | | 0.00 | 0 | | 0.00 | | 0 | | 0.16 | | 1 | | 0.00 | | 0 | | 0.00 | | 0 | | 0.00 | | 0 | | 0.00 | | 0 | | 0.00 | | 0 | | 1 | | | 0 | | 1 | |  |  |  |  |
| O43148 | mRNA cap guanine-N7 methyltransferase | RNMT | 6.30 | 1 | | 54.8 | | 6.61 | | 0.00 | 0 | | 0.00 | | 0 | | 0.16 | | 1 | | 0.00 | | 0 | | 0.00 | | 0 | | 0.00 | | 0 | | 0.00 | | 0 | | 0.00 | | 0 | | 1 | | | 0 | | 1 | |  |  |  |  |
| Q8N3E9 | 1-phosphatidylinositol 4,5-bisphosphate phosphodiesterase delta-3 | PLCD3 | 2.92 | 1 | | 89.2 | | 6.98 | | 0.00 | 0 | | 0.00 | | 0 | | 0.16 | | 1 | | 0.00 | | 0 | | 0.00 | | 0 | | 0.00 | | 0 | | 0.00 | | 0 | | 0.00 | | 0 | | 1 | | | 0 | | 1 | |  |  |  |  |
| Q6ZNL6 | FYVE, RhoGEF and PH domain-containing protein 5 | FGD5 | 1.92 | 1 | | 159.8 | | 5.01 | | 0.00 | 0 | | 0.00 | | 0 | | 0.16 | | 1 | | 0.00 | | 0 | | 0.00 | | 0 | | 0.00 | | 0 | | 0.00 | | 0 | | 0.00 | | 0 | | 1 | | | 0 | | 1 | |  |  |  |  |
| O60343 | TBC1 domain family member 4 | TBC1D4 | 1.16 | 1 | | 146.5 | | 7.01 | | 0.00 | 0 | | 0.00 | | 0 | | 0.16 | | 1 | | 0.00 | | 0 | | 0.00 | | 0 | | 0.00 | | 0 | | 0.00 | | 0 | | 0.00 | | 0 | | 1 | | | 0 | | 1 | |  |  |  |  |
| O60934 | Nibrin | NBN | 1.46 | 1 | | 84.9 | | 6.90 | | 0.00 | 0 | | 0.00 | | 0 | | 0.16 | | 1 | | 0.00 | | 0 | | 0.00 | | 0 | | 0.00 | | 0 | | 0.00 | | 0 | | 0.00 | | 0 | | 1 | | | 0 | | 1 | |  |  |  |  |
| Q5VZL5 | Zinc finger MYM-type protein 4 | ZMYM4 | 1.10 | 1 | | 172.7 | | 6.84 | | 0.00 | 0 | | 0.00 | | 0 | | 0.16 | | 1 | | 0.00 | | 0 | | 0.00 | | 0 | | 0.00 | | 0 | | 0.00 | | 0 | | 0.00 | | 0 | | 1 | | | 0 | | 1 | |  |  |  |  |
| Q8N565 | Melanoregulin | MREG | 6.54 | 1 | | 24.9 | | 5.73 | | 0.00 | 0 | | 0.00 | | 0 | | 0.16 | | 1 | | 0.00 | | 0 | | 0.00 | | 0 | | 0.00 | | 0 | | 0.00 | | 0 | | 0.00 | | 0 | | 1 | | | 0 | | 1 | |  |  |  |  |
| H0YI54 | Keratin, type II cytoskeletal 78 (Fragment) | KRT78 | 21.95 | 1 | | 5.0 | | 8.05 | | 0.00 | 0 | | 0.00 | | 0 | | 0.16 | | 1 | | 0.00 | | 0 | | 0.00 | | 0 | | 0.00 | | 0 | | 0.00 | | 0 | | 0.00 | | 0 | | 1 | | | 0 | | 1 | |  |  |  |  |
| Q9H813 | Proton-activated chloride channel | PACC1 | 8.57 | 1 | | 40.0 | | 8.88 | | 0.00 | 0 | | 0.00 | | 0 | | 0.16 | | 1 | | 0.00 | | 0 | | 0.00 | | 0 | | 0.00 | | 0 | | 0.00 | | 0 | | 0.00 | | 0 | | 1 | | | 0 | | 1 | |  |  |  |  |
| P61073 | C-X-C chemokine receptor type 4 | CXCR4 | 7.10 | 1 | | 39.7 | | 8.21 | | 0.00 | 0 | | 0.00 | | 0 | | 0.16 | | 1 | | 0.00 | | 0 | | 0.00 | | 0 | | 0.00 | | 0 | | 0.00 | | 0 | | 0.00 | | 0 | | 1 | | | 0 | | 1 | |  |  |  |  |
| P48443 | Retinoic acid receptor RXR-gamma | RXRG | 12.10 | 1 | | 50.8 | | 7.62 | | 0.00 | 0 | | 0.00 | | 0 | | 0.16 | | 1 | | 0.00 | | 0 | | 0.00 | | 0 | | 0.00 | | 0 | | 0.00 | | 0 | | 0.00 | | 0 | | 1 | | | 0 | | 1 | |  |  |  |  |
| A0A3F2YP58 | Phosphodiesterase | PDE10A | 0.66 | 1 | | 114.9 | | 7.55 | | 0.00 | 0 | | 0.00 | | 0 | | 0.16 | | 1 | | 0.00 | | 0 | | 0.00 | | 0 | | 0.00 | | 0 | | 0.00 | | 0 | | 0.00 | | 0 | | 1 | | | 0 | | 1 | |  |  |  |  |
| Q96AA8 | Janus kinase and microtubule-interacting protein 2 | JAKMIP2 | 1.23 | 1 | | 94.9 | | 6.16 | | 0.00 | 0 | | 0.00 | | 0 | | 0.16 | | 1 | | 0.00 | | 0 | | 0.00 | | 0 | | 0.00 | | 0 | | 0.00 | | 0 | | 0.00 | | 0 | | 1 | | | 0 | | 1 | |  |  |  |  |
| G3XAE9 | KIAA0423, isoform CRA_a | TOGARAM1 | 1.24 | 1 | | 195.1 | | 8.40 | | 0.00 | 0 | | 0.00 | | 0 | | 0.16 | | 1 | | 0.00 | | 0 | | 0.00 | | 0 | | 0.00 | | 0 | | 0.00 | | 0 | | 0.00 | | 0 | | 1 | | | 0 | | 1 | |  |  |  |  |
| Q9BXP2 | Solute carrier family 12 member 9 | SLC12A9 | 1.09 | 1 | | 96.0 | | 8.07 | | 0.00 | 0 | | 0.00 | | 0 | | 0.16 | | 1 | | 0.00 | | 0 | | 0.00 | | 0 | | 0.00 | | 0 | | 0.00 | | 0 | | 0.00 | | 0 | | 1 | | | 0 | | 1 | |  |  |  |  |
| Q86VY9 | Transmembrane protein 200A | TMEM200A | 6.52 | 1 | | 54.3 | | 9.17 | | 0.00 | 0 | | 0.00 | | 0 | | 0.16 | | 1 | | 0.00 | | 0 | | 0.00 | | 0 | | 0.00 | | 0 | | 0.00 | | 0 | | 0.00 | | 0 | | 1 | | | 0 | | 1 | |  |  |  |  |
| Q9H008 | Phospholysine phosphohistidine inorganic pyrophosphate phosphatase | LHPP | 7.78 | 1 | | 29.1 | | 6.15 | | 0.00 | 0 | | 0.00 | | 0 | | 0.16 | | 1 | | 0.00 | | 0 | | 0.00 | | 0 | | 0.00 | | 0 | | 0.00 | | 0 | | 0.00 | | 0 | | 1 | | | 0 | | 1 | |  |  |  |  |
| Q8N7U6 | EF-hand domain-containing family member B | EFHB | 1.92 | 1 | | 93.7 | | 7.58 | | 0.00 | 0 | | 0.00 | | 0 | | 0.16 | | 1 | | 0.00 | | 0 | | 0.00 | | 0 | | 0.00 | | 0 | | 0.00 | | 0 | | 0.00 | | 0 | | 1 | | | 0 | | 1 | |  |  |  |  |
| O43290 | U4/U6.U5 tri-snRNP-associated protein 1 | SART1 | 1.25 | 1 | | 90.2 | | 6.13 | | 0.00 | 0 | | 0.00 | | 0 | | 0.16 | | 1 | | 0.00 | | 0 | | 0.00 | | 0 | | 0.00 | | 0 | | 0.00 | | 0 | | 0.00 | | 0 | | 1 | | | 0 | | 1 | |  |  |  |  |
| Q8WZA9 | Immunity-related GTPase family Q protein | IRGQ | 1.77 | 1 | | 62.7 | | 4.88 | | 0.00 | 0 | | 0.00 | | 0 | | 0.16 | | 1 | | 0.00 | | 0 | | 0.00 | | 0 | | 0.00 | | 0 | | 0.00 | | 0 | | 0.00 | | 0 | | 1 | | | 0 | | 1 | |  |  |  |  |
| P20023 | Complement receptor type 2 | CR2 | 3.10 | 1 | | 112.8 | | 7.52 | | 0.00 | 0 | | 0.00 | | 0 | | 0.16 | | 1 | | 0.00 | | 0 | | 0.00 | | 0 | | 0.00 | | 0 | | 0.00 | | 0 | | 0.00 | | 0 | | 1 | | | 0 | | 1 | |  |  |  |  |
| Q9Y2J2 | Band 4.1-like protein 3 | EPB41L3 | 1.38 | 1 | | 120.6 | | 5.19 | | 0.00 | 0 | | 0.00 | | 0 | | 0.16 | | 1 | | 0.00 | | 0 | | 0.00 | | 0 | | 0.00 | | 0 | | 0.00 | | 0 | | 0.00 | | 0 | | 1 | | | 0 | | 1 | |  |  |  |  |
| Q96HN2 | Adenosylhomocysteinase 3 | AHCYL2 | 1.31 | 1 | | 66.7 | | 7.36 | | 0.00 | 0 | | 0.00 | | 0 | | 0.16 | | 1 | | 0.00 | | 0 | | 0.00 | | 0 | | 0.00 | | 0 | | 0.00 | | 0 | | 0.00 | | 0 | | 1 | | | 0 | | 1 | |  |  |  |  |
| Q9P2G9 | Kelch-like protein 8 | KLHL8 | 4.03 | 1 | | 68.8 | | 6.58 | | 0.00 | 0 | | 0.00 | | 0 | | 0.16 | | 1 | | 0.00 | | 0 | | 0.00 | | 0 | | 0.00 | | 0 | | 0.00 | | 0 | | 0.00 | | 0 | | 1 | | | 0 | | 1 | |  |  |  |  |
| A6NL08 | Olfactory receptor 6C75 | OR6C75 | 2.24 | 1 | | 35.3 | | 9.07 | | 0.00 | 0 | | 0.00 | | 0 | | 0.16 | | 1 | | 0.00 | | 0 | | 0.00 | | 0 | | 0.00 | | 0 | | 0.00 | | 0 | | 0.00 | | 0 | | 1 | | | 0 | | 1 | |  |  |  |  |
| A2RUR9 | Coiled-coil domain-containing protein 144A | CCDC144A | 0.70 | 1 | | 165.0 | | 5.36 | | 0.00 | 0 | | 0.00 | | 0 | | 0.16 | | 1 | | 0.00 | | 0 | | 0.00 | | 0 | | 0.00 | | 0 | | 0.00 | | 0 | | 0.00 | | 0 | | 1 | | | 0 | | 1 | |  |  |  |  |
| A6NE01 | Protein FAM186A | FAM186A | 0.68 | 1 | | 262.6 | | 8.60 | | 0.00 | 0 | | 0.00 | | 0 | | 0.16 | | 1 | | 0.00 | | 0 | | 0.00 | | 0 | | 0.00 | | 0 | | 0.00 | | 0 | | 0.00 | | 0 | | 1 | | | 0 | | 1 | |  |  |  |  |
| O60268 | Uncharacterized protein KIAA0513 | KIAA0513 | 5.84 | 1 | | 46.6 | | 5.06 | | 0.00 | 0 | | 0.00 | | 0 | | 0.16 | | 1 | | 0.00 | | 0 | | 0.00 | | 0 | | 0.00 | | 0 | | 0.00 | | 0 | | 0.00 | | 0 | | 1 | | | 0 | | 1 | |  |  |  |  |
| Q92545 | Transmembrane protein 131 | TMEM131 | 0.64 | 1 | | 205.0 | | 8.59 | | 0.00 | 0 | | 0.00 | | 0 | | 0.16 | | 1 | | 0.00 | | 0 | | 0.00 | | 0 | | 0.00 | | 0 | | 0.00 | | 0 | | 0.00 | | 0 | | 1 | | | 0 | | 1 | |  |  |  |  |
| A6NHT5 | Homeobox protein HMX3 | HMX3 | 7.28 | 1 | | 37.8 | | 8.12 | | 0.00 | 0 | | 0.00 | | 0 | | 0.16 | | 1 | | 0.00 | | 0 | | 0.00 | | 0 | | 0.00 | | 0 | | 0.00 | | 0 | | 0.00 | | 0 | | 1 | | | 0 | | 1 | |  |  |  |  |
| P20936 | Ras GTPase-activating protein 1 | RASA1 | 0.96 | 1 | | 116.3 | | 6.54 | | 0.00 | 0 | | 0.00 | | 0 | | 0.16 | | 1 | | 0.00 | | 0 | | 0.00 | | 0 | | 0.00 | | 0 | | 0.00 | | 0 | | 0.00 | | 0 | | 1 | | | 0 | | 1 | |  |  |  |  |
| Q68E01 | Integrator complex subunit 3 | INTS3 | 2.40 | 1 | | 118.0 | | 5.80 | | 0.00 | 0 | | 0.00 | | 0 | | 0.16 | | 1 | | 0.00 | | 0 | | 0.00 | | 0 | | 0.00 | | 0 | | 0.00 | | 0 | | 0.00 | | 0 | | 1 | | | 0 | | 1 | |  |  |  |  |
| A0A494C0S1 | GTP-binding protein Rit1 | RIT1 | 3.64 | 1 | | 25.3 | | 9.10 | | 0.00 | 0 | | 0.00 | | 0 | | 0.16 | | 1 | | 0.00 | | 0 | | 0.00 | | 0 | | 0.00 | | 0 | | 0.00 | | 0 | | 0.00 | | 0 | | 1 | | | 0 | | 1 | |  |  |  |  |
| P0DO97 | Coiled-coil domain-containing protein 192 | CCDC192 | 5.82 | 1 | | 32.5 | | 5.02 | | 0.00 | 0 | | 0.00 | | 0 | | 0.16 | | 1 | | 0.00 | | 0 | | 0.00 | | 0 | | 0.00 | | 0 | | 0.00 | | 0 | | 0.00 | | 0 | | 1 | | | 0 | | 1 | |  |  |  |  |
| Q96NJ5 | Kelch-like protein 32 | KLHL32 | 4.19 | 1 | | 70.3 | | 6.42 | | 0.00 | 0 | | 0.00 | | 0 | | 0.16 | | 1 | | 0.00 | | 0 | | 0.00 | | 0 | | 0.00 | | 0 | | 0.00 | | 0 | | 0.00 | | 0 | | 1 | | | 0 | | 1 | |  |  |  |  |
| E7EVL8 | Cytochrome P450 4A11 | CYP4A11 | 6.35 | 1 | | 59.4 | | 8.76 | | 0.00 | 0 | | 0.00 | | 0 | | 0.16 | | 1 | | 0.00 | | 0 | | 0.00 | | 0 | | 0.00 | | 0 | | 0.00 | | 0 | | 0.00 | | 0 | | 1 | | | 0 | | 1 | |  |  |  |  |
| Q9Y2A7 | Nck-associated protein 1 | NCKAP1 | 0.80 | 1 | | 128.7 | | 6.62 | | 0.00 | 0 | | 0.00 | | 0 | | 0.16 | | 1 | | 0.00 | | 0 | | 0.00 | | 0 | | 0.00 | | 0 | | 0.00 | | 0 | | 0.00 | | 0 | | 1 | | | 0 | | 1 | |  |  |  |  |
| Q3V6T2 | Girdin | CCDC88A | 2.99 | 2 | | 215.9 | | 6.21 | | 0.00 | 0 | | 0.00 | | 0 | | 0.00 | | 0 | | 0.42 | | 1 | | 0.00 | | 0 | | 0.00 | | 0 | | 0.00 | | 0 | | 0.00 | | 0 | | 1 | | | 0 | | 1 | |  |  |  |  |
| P24592 | Insulin-like growth factor-binding protein 6 | IGFBP6 | 4.17 | 1 | | 25.3 | | 7.81 | | 0.00 | 0 | | 0.00 | | 0 | | 0.00 | | 0 | | 0.37 | | 2 | | 0.00 | | 0 | | 0.00 | | 0 | | 0.00 | | 0 | | 0.00 | | 0 | | 1 | | | 0 | | 1 | |  |  |  |  |
| Q6ZT12 | E3 ubiquitin-protein ligase UBR3 | UBR3 | 0.42 | 1 | | 212.3 | | 6.10 | | 0.00 | 0 | | 0.00 | | 0 | | 0.00 | | 0 | | 0.37 | | 2 | | 0.00 | | 0 | | 0.00 | | 0 | | 0.00 | | 0 | | 0.00 | | 0 | | 1 | | | 0 | | 1 | |  |  |  |  |
| P27815 | cAMP-specific 3',5'-cyclic phosphodiesterase 4A | PDE4A | 1.13 | 1 | | 98.1 | | 5.21 | | 0.00 | 0 | | 0.00 | | 0 | | 0.00 | | 0 | | 0.37 | | 2 | | 0.00 | | 0 | | 0.00 | | 0 | | 0.00 | | 0 | | 0.00 | | 0 | | 1 | | | 0 | | 1 | |  |  |  |  |
| O14490 | Disks large-associated protein 1 | DLGAP1 | 4.61 | 1 | | 108.8 | | 7.08 | | 0.00 | 0 | | 0.00 | | 0 | | 0.00 | | 0 | | 0.36 | | 2 | | 0.00 | | 0 | | 0.00 | | 0 | | 0.00 | | 0 | | 0.00 | | 0 | | 1 | | | 0 | | 1 | |  |  |  |  |
| H7BZF5 | Uncharacterized protein C2orf80 (Fragment) | C2orf80 | 5.76 | 1 | | 15.7 | | 9.83 | | 0.00 | 0 | | 0.00 | | 0 | | 0.00 | | 0 | | 0.32 | | 2 | | 0.00 | | 0 | | 0.00 | | 0 | | 0.00 | | 0 | | 0.00 | | 0 | | 1 | | | 0 | | 1 | |  |  |  |  |
| Q8IXS2 | Dynein regulatory complex subunit 2 | CCDC65 | 1.65 | 1 | | 57.3 | | 7.43 | | 0.00 | 0 | | 0.00 | | 0 | | 0.00 | | 0 | | 0.32 | | 2 | | 0.00 | | 0 | | 0.00 | | 0 | | 0.00 | | 0 | | 0.00 | | 0 | | 1 | | | 0 | | 1 | |  |  |  |  |
| J3QTJ5 | Leucine-rich repeat-containing protein 37A3 | LRRC37A3 | 7.58 | 2 | | 84.1 | | 8.32 | | 0.00 | 0 | | 0.00 | | 0 | | 0.00 | | 0 | | 0.32 | | 2 | | 0.00 | | 0 | | 0.00 | | 0 | | 0.00 | | 0 | | 0.00 | | 0 | | 1 | | | 0 | | 1 | |  |  |  |  |
| Q9Y5Z4 | Heme-binding protein 2 | HEBP2 | 7.32 | 1 | | 22.9 | | 4.63 | | 0.00 | 0 | | 0.00 | | 0 | | 0.00 | | 0 | | 0.31 | | 2 | | 0.00 | | 0 | | 0.00 | | 0 | | 0.00 | | 0 | | 0.00 | | 0 | | 1 | | | 0 | | 1 | |  |  |  |  |
| Q9Y227 | Ectonucleoside triphosphate diphosphohydrolase 4 | ENTPD4 | 3.25 | 1 | | 70.2 | | 8.29 | | 0.00 | 0 | | 0.00 | | 0 | | 0.00 | | 0 | | 0.21 | | 1 | | 0.00 | | 0 | | 0.00 | | 0 | | 0.00 | | 0 | | 0.00 | | 0 | | 1 | | | 0 | | 1 | |  |  |  |  |
| E7EWN3 | Histone-lysine N-methyltransferase SETD5 | SETD5 | 0.75 | 1 | | 159.3 | | 8.43 | | 0.00 | 0 | | 0.00 | | 0 | | 0.00 | | 0 | | 0.21 | | 1 | | 0.00 | | 0 | | 0.00 | | 0 | | 0.00 | | 0 | | 0.00 | | 0 | | 1 | | | 0 | | 1 | |  |  |  |  |
| A0A1W2PQH5 | Lactosylceramide alpha-2,3-sialyltransferase | ST3GAL5 | 4.39 | 1 | | 13.2 | | 9.79 | | 0.00 | 0 | | 0.00 | | 0 | | 0.00 | | 0 | | 0.21 | | 1 | | 0.00 | | 0 | | 0.00 | | 0 | | 0.00 | | 0 | | 0.00 | | 0 | | 1 | | | 0 | | 1 | |  |  |  |  |
| C1ID54 | Cytochrome P450 2D6 | CYP2D6 | 6.04 | 1 | | 55.8 | | 7.42 | | 0.00 | 0 | | 0.00 | | 0 | | 0.00 | | 0 | | 0.21 | | 1 | | 0.00 | | 0 | | 0.00 | | 0 | | 0.00 | | 0 | | 0.00 | | 0 | | 1 | | | 0 | | 1 | |  |  |  |  |
| Q5VWI1 | Transcription elongation regulator 1-like protein | TCERG1L | 3.41 | 1 | | 65.6 | | 9.83 | | 0.00 | 0 | | 0.00 | | 0 | | 0.00 | | 0 | | 0.21 | | 1 | | 0.00 | | 0 | | 0.00 | | 0 | | 0.00 | | 0 | | 0.00 | | 0 | | 1 | | | 0 | | 1 | |  |  |  |  |
| Q8TF76 | Serine/threonine-protein kinase haspin | HASPIN | 0.88 | 1 | | 88.4 | | 9.16 | | 0.00 | 0 | | 0.00 | | 0 | | 0.00 | | 0 | | 0.21 | | 1 | | 0.00 | | 0 | | 0.00 | | 0 | | 0.00 | | 0 | | 0.00 | | 0 | | 1 | | | 0 | | 1 | |  |  |  |  |
| Q70Z35 | Phosphatidylinositol 3,4,5-trisphosphate-dependent Rac exchanger 2 protein | PREX2 | 1.93 | 1 | | 182.5 | | 7.44 | | 0.00 | 0 | | 0.00 | | 0 | | 0.00 | | 0 | | 0.21 | | 1 | | 0.00 | | 0 | | 0.00 | | 0 | | 0.00 | | 0 | | 0.00 | | 0 | | 1 | | | 0 | | 1 | |  |  |  |  |
| Q9NRP7 | Serine/threonine-protein kinase 36 | STK36 | 0.61 | 1 | | 143.9 | | 5.90 | | 0.00 | 0 | | 0.00 | | 0 | | 0.00 | | 0 | | 0.21 | | 1 | | 0.00 | | 0 | | 0.00 | | 0 | | 0.00 | | 0 | | 0.00 | | 0 | | 1 | | | 0 | | 1 | |  |  |  |  |
| B5ME97 | Septin 10, isoform CRA_c | SEPTIN10 | 4.96 | 1 | | 62.9 | | 6.83 | | 0.00 | 0 | | 0.00 | | 0 | | 0.00 | | 0 | | 0.21 | | 1 | | 0.00 | | 0 | | 0.00 | | 0 | | 0.00 | | 0 | | 0.00 | | 0 | | 1 | | | 0 | | 1 | |  |  |  |  |
| Q9P232 | Contactin-3 | CNTN3 | 1.56 | 1 | | 112.8 | | 6.30 | | 0.00 | 0 | | 0.00 | | 0 | | 0.00 | | 0 | | 0.21 | | 1 | | 0.00 | | 0 | | 0.00 | | 0 | | 0.00 | | 0 | | 0.00 | | 0 | | 1 | | | 0 | | 1 | |  |  |  |  |
| Q8N2S1 | Latent-transforming growth factor beta-binding protein 4 | LTBP4 | 3.20 | 1 | | 173.3 | | 5.43 | | 0.00 | 0 | | 0.00 | | 0 | | 0.00 | | 0 | | 0.21 | | 1 | | 0.00 | | 0 | | 0.00 | | 0 | | 0.00 | | 0 | | 0.00 | | 0 | | 1 | | | 0 | | 1 | |  |  |  |  |
| H0YD55 | Chromosome 11 open reading frame 80 (Fragment) | C11orf80 | 7.14 | 1 | | 23.0 | | 8.98 | | 0.00 | 0 | | 0.00 | | 0 | | 0.00 | | 0 | | 0.21 | | 1 | | 0.00 | | 0 | | 0.00 | | 0 | | 0.00 | | 0 | | 0.00 | | 0 | | 1 | | | 0 | | 1 | |  |  |  |  |
| Q13938 | Calcyphosin | CAPS | 2.55 | 1 | | 30.2 | | 6.04 | | 0.00 | 0 | | 0.00 | | 0 | | 0.00 | | 0 | | 0.21 | | 1 | | 0.00 | | 0 | | 0.00 | | 0 | | 0.00 | | 0 | | 0.00 | | 0 | | 1 | | | 0 | | 1 | |  |  |  |  |
| A0A494C1N2 | Caspase recruitment domain-containing protein 14 | CARD14 | 1.09 | 1 | | 114.1 | | 5.87 | | 0.00 | 0 | | 0.00 | | 0 | | 0.00 | | 0 | | 0.21 | | 1 | | 0.00 | | 0 | | 0.00 | | 0 | | 0.00 | | 0 | | 0.00 | | 0 | | 1 | | | 0 | | 1 | |  |  |  |  |
| Q8WVE0 | EEF1A lysine methyltransferase 1 | EEF1AKMT1 | 5.61 | 1 | | 24.5 | | 4.55 | | 0.00 | 0 | | 0.00 | | 0 | | 0.00 | | 0 | | 0.21 | | 1 | | 0.00 | | 0 | | 0.00 | | 0 | | 0.00 | | 0 | | 0.00 | | 0 | | 1 | | | 0 | | 1 | |  |  |  |  |
| A0A494C0N0 | Poly(A)-specific ribonuclease PARN (Fragment) | PARN | 12.24 | 1 | | 5.7 | | 7.34 | | 0.00 | 0 | | 0.00 | | 0 | | 0.00 | | 0 | | 0.21 | | 1 | | 0.00 | | 0 | | 0.00 | | 0 | | 0.00 | | 0 | | 0.00 | | 0 | | 1 | | | 0 | | 1 | |  |  |  |  |
| J3KR90 | Atrial natriuretic peptide-converting enzyme | CORIN | 3.38 | 1 | | 112.3 | | 5.08 | | 0.00 | 0 | | 0.00 | | 0 | | 0.00 | | 0 | | 0.21 | | 1 | | 0.00 | | 0 | | 0.00 | | 0 | | 0.00 | | 0 | | 0.00 | | 0 | | 1 | | | 0 | | 1 | |  |  |  |  |
| P83111 | Serine beta-lactamase-like protein LACTB, mitochondrial | LACTB | 3.11 | 1 | | 60.7 | | 8.53 | | 0.00 | 0 | | 0.00 | | 0 | | 0.00 | | 0 | | 0.21 | | 1 | | 0.00 | | 0 | | 0.00 | | 0 | | 0.00 | | 0 | | 0.00 | | 0 | | 1 | | | 0 | | 1 | |  |  |  |  |
| Q8WY64 | E3 ubiquitin-protein ligase MYLIP | MYLIP | 2.47 | 1 | | 49.9 | | 7.24 | | 0.00 | 0 | | 0.00 | | 0 | | 0.00 | | 0 | | 0.21 | | 1 | | 0.00 | | 0 | | 0.00 | | 0 | | 0.00 | | 0 | | 0.00 | | 0 | | 1 | | | 0 | | 1 | |  |  |  |  |
| Q12802 | A-kinase anchor protein 13 | AKAP13 | 0.64 | 1 | | 307.4 | | 5.24 | | 0.00 | 0 | | 0.00 | | 0 | | 0.00 | | 0 | | 0.21 | | 1 | | 0.00 | | 0 | | 0.00 | | 0 | | 0.00 | | 0 | | 0.00 | | 0 | | 1 | | | 0 | | 1 | |  |  |  |  |
| Q8IX15 | Homeobox and leucine zipper protein Homez | HOMEZ | 3.09 | 1 | | 61.2 | | 4.93 | | 0.00 | 0 | | 0.00 | | 0 | | 0.00 | | 0 | | 0.21 | | 1 | | 0.00 | | 0 | | 0.00 | | 0 | | 0.00 | | 0 | | 0.00 | | 0 | | 1 | | | 0 | | 1 | |  |  |  |  |
| A0A0R4J2E6 | ELAV-like protein | ELAVL4 | 5.22 | 1 | | 44.5 | | 9.38 | | 0.00 | 0 | | 0.00 | | 0 | | 0.00 | | 0 | | 0.21 | | 1 | | 0.00 | | 0 | | 0.00 | | 0 | | 0.00 | | 0 | | 0.00 | | 0 | | 1 | | | 0 | | 1 | |  |  |  |  |
| A0A1W2PQB3 | Thiamin pyrophosphokinase 1 | TPK1 | 6.67 | 1 | | 15.3 | | 8.76 | | 0.00 | 0 | | 0.00 | | 0 | | 0.00 | | 0 | | 0.21 | | 1 | | 0.00 | | 0 | | 0.00 | | 0 | | 0.00 | | 0 | | 0.00 | | 0 | | 1 | | | 0 | | 1 | |  |  |  |  |
| P02753 | Retinol-binding protein 4 | RBP4 | 4.98 | 1 | | 23.0 | | 6.07 | | 0.00 | 0 | | 0.00 | | 0 | | 0.00 | | 0 | | 0.21 | | 1 | | 0.00 | | 0 | | 0.00 | | 0 | | 0.00 | | 0 | | 0.00 | | 0 | | 1 | | | 0 | | 1 | |  |  |  |  |
| Q14980 | Nuclear mitotic apparatus protein 1 | NUMA1 | 0.76 | 1 | | 238.1 | | 5.78 | | 0.00 | 0 | | 0.00 | | 0 | | 0.00 | | 0 | | 0.21 | | 1 | | 0.00 | | 0 | | 0.00 | | 0 | | 0.00 | | 0 | | 0.00 | | 0 | | 1 | | | 0 | | 1 | |  |  |  |  |
| O00534 | von Willebrand factor A domain-containing protein 5A | VWA5A | 5.34 | 1 | | 86.4 | | 6.58 | | 0.00 | 0 | | 0.00 | | 0 | | 0.00 | | 0 | | 0.21 | | 1 | | 0.00 | | 0 | | 0.00 | | 0 | | 0.00 | | 0 | | 0.00 | | 0 | | 1 | | | 0 | | 1 | |  |  |  |  |
| O60551 | Glycylpeptide N-tetradecanoyltransferase 2 | NMT2 | 3.82 | 1 | | 56.9 | | 7.58 | | 0.00 | 0 | | 0.00 | | 0 | | 0.00 | | 0 | | 0.21 | | 1 | | 0.00 | | 0 | | 0.00 | | 0 | | 0.00 | | 0 | | 0.00 | | 0 | | 1 | | | 0 | | 1 | |  |  |  |  |
| Q8N3D4 | EH domain-binding protein 1-like protein 1 | EHBP1L1 | 4.20 | 1 | | 161.8 | | 4.83 | | 0.00 | 0 | | 0.00 | | 0 | | 0.00 | | 0 | | 0.21 | | 1 | | 0.00 | | 0 | | 0.00 | | 0 | | 0.00 | | 0 | | 0.00 | | 0 | | 1 | | | 0 | | 1 | |  |  |  |  |
| Q8WXK3 | Ankyrin repeat and SOCS box protein 13 | ASB13 | 6.83 | 1 | | 30.0 | | 6.81 | | 0.00 | 0 | | 0.00 | | 0 | | 0.00 | | 0 | | 0.21 | | 1 | | 0.00 | | 0 | | 0.00 | | 0 | | 0.00 | | 0 | | 0.00 | | 0 | | 1 | | | 0 | | 1 | |  |  |  |  |
| Q2NL82 | Pre-rRNA-processing protein TSR1 homolog | TSR1 | 1.12 | 1 | | 91.8 | | 7.42 | | 0.00 | 0 | | 0.00 | | 0 | | 0.00 | | 0 | | 0.21 | | 1 | | 0.00 | | 0 | | 0.00 | | 0 | | 0.00 | | 0 | | 0.00 | | 0 | | 1 | | | 0 | | 1 | |  |  |  |  |
| A0A5F9ZHW2 | DNA polymerase alpha catalytic subunit | POLA1 | 7.29 | 1 | | 11.0 | | 10.14 | | 0.00 | 0 | | 0.00 | | 0 | | 0.00 | | 0 | | 0.21 | | 1 | | 0.00 | | 0 | | 0.00 | | 0 | | 0.00 | | 0 | | 0.00 | | 0 | | 1 | | | 0 | | 1 | |  |  |  |  |
| I6L8A6 | DNA endonuclease RBBP8 | RBBP8 | 0.78 | 1 | | 102.5 | | 6.24 | | 0.00 | 0 | | 0.00 | | 0 | | 0.00 | | 0 | | 0.21 | | 1 | | 0.00 | | 0 | | 0.00 | | 0 | | 0.00 | | 0 | | 0.00 | | 0 | | 1 | | | 0 | | 1 | |  |  |  |  |
| Q8NHU3 | Phosphatidylcholine:ceramide cholinephosphotransferase 2 | SGMS2 | 9.86 | 1 | | 42.3 | | 8.81 | | 0.00 | 0 | | 0.00 | | 0 | | 0.00 | | 0 | | 0.21 | | 1 | | 0.00 | | 0 | | 0.00 | | 0 | | 0.00 | | 0 | | 0.00 | | 0 | | 1 | | | 0 | | 1 | |  |  |  |  |
| P51654 | Glypican-3 | GPC3 | 5.00 | 1 | | 65.5 | | 6.37 | | 0.00 | 0 | | 0.00 | | 0 | | 0.00 | | 0 | | 0.21 | | 1 | | 0.00 | | 0 | | 0.00 | | 0 | | 0.00 | | 0 | | 0.00 | | 0 | | 1 | | | 0 | | 1 | |  |  |  |  |
| Q9Y6N6 | Laminin subunit gamma-3 | LAMC3 | 1.84 | 1 | | 171.1 | | 6.58 | | 0.00 | 0 | | 0.00 | | 0 | | 0.00 | | 0 | | 0.21 | | 1 | | 0.00 | | 0 | | 0.00 | | 0 | | 0.00 | | 0 | | 0.00 | | 0 | | 1 | | | 0 | | 1 | |  |  |  |  |
| Q92667 | A-kinase anchor protein 1, mitochondrial | AKAP1 | 2.88 | 1 | | 97.3 | | 4.94 | | 0.00 | 0 | | 0.00 | | 0 | | 0.00 | | 0 | | 0.21 | | 1 | | 0.00 | | 0 | | 0.00 | | 0 | | 0.00 | | 0 | | 0.00 | | 0 | | 1 | | | 0 | | 1 | |  |  |  |  |
| Q9P2E7 | Protocadherin-10 | PCDH10 | 1.25 | 1 | | 112.9 | | 4.87 | | 0.00 | 0 | | 0.00 | | 0 | | 0.00 | | 0 | | 0.21 | | 1 | | 0.00 | | 0 | | 0.00 | | 0 | | 0.00 | | 0 | | 0.00 | | 0 | | 1 | | | 0 | | 1 | |  |  |  |  |
| H7C5V5 | Mitochondrial fission regulator 1 (Fragment) | MTFR1 | 2.78 | 1 | | 15.9 | | 9.57 | | 0.00 | 0 | | 0.00 | | 0 | | 0.00 | | 0 | | 0.21 | | 1 | | 0.00 | | 0 | | 0.00 | | 0 | | 0.00 | | 0 | | 0.00 | | 0 | | 1 | | | 0 | | 1 | |  |  |  |  |
| Q8TF39 | Zinc finger protein 483 | ZNF483 | 3.76 | 1 | | 85.0 | | 8.57 | | 0.00 | 0 | | 0.00 | | 0 | | 0.00 | | 0 | | 0.17 | | 1 | | 0.00 | | 0 | | 0.00 | | 0 | | 0.00 | | 0 | | 0.00 | | 0 | | 1 | | | 0 | | 1 | |  |  |  |  |
| E7EQ45 | AP-5 complex subunit mu-1 | AP5M1 | 11.51 | 1 | | 56.4 | | 6.32 | | 0.00 | 0 | | 0.00 | | 0 | | 0.00 | | 0 | | 0.17 | | 1 | | 0.00 | | 0 | | 0.00 | | 0 | | 0.00 | | 0 | | 0.00 | | 0 | | 1 | | | 0 | | 1 | |  |  |  |  |
| Q1L5Z9 | LON peptidase N-terminal domain and RING finger protein 2 | LONRF2 | 3.58 | 1 | | 83.6 | | 5.90 | | 0.00 | 0 | | 0.00 | | 0 | | 0.00 | | 0 | | 0.17 | | 1 | | 0.00 | | 0 | | 0.00 | | 0 | | 0.00 | | 0 | | 0.00 | | 0 | | 1 | | | 0 | | 1 | |  |  |  |  |
| Q7RTU9 | Stereocilin | STRC | 3.49 | 1 | | 192.8 | | 5.41 | | 0.00 | 0 | | 0.00 | | 0 | | 0.00 | | 0 | | 0.17 | | 1 | | 0.00 | | 0 | | 0.00 | | 0 | | 0.00 | | 0 | | 0.00 | | 0 | | 1 | | | 0 | | 1 | |  |  |  |  |
| O95071 | E3 ubiquitin-protein ligase UBR5 | UBR5 | 0.57 | 1 | | 309.2 | | 5.85 | | 0.00 | 0 | | 0.00 | | 0 | | 0.00 | | 0 | | 0.17 | | 1 | | 0.00 | | 0 | | 0.00 | | 0 | | 0.00 | | 0 | | 0.00 | | 0 | | 1 | | | 0 | | 1 | |  |  |  |  |
| Q7LBE3 | Solute carrier family 26 member 9 | SLC26A9 | 3.67 | 1 | | 86.9 | | 8.22 | | 0.00 | 0 | | 0.00 | | 0 | | 0.00 | | 0 | | 0.17 | | 1 | | 0.00 | | 0 | | 0.00 | | 0 | | 0.00 | | 0 | | 0.00 | | 0 | | 1 | | | 0 | | 1 | |  |  |  |  |
| R4GN13 | Choline O-acetyltransferase | CHAT | 13.39 | 1 | | 11.7 | | 11.00 | | 0.00 | 0 | | 0.00 | | 0 | | 0.00 | | 0 | | 0.17 | | 1 | | 0.00 | | 0 | | 0.00 | | 0 | | 0.00 | | 0 | | 0.00 | | 0 | | 1 | | | 0 | | 1 | |  |  |  |  |
| Q9H0C1 | Zinc finger MYND domain-containing protein 12 | ZMYND12 | 3.29 | 1 | | 41.8 | | 6.23 | | 0.00 | 0 | | 0.00 | | 0 | | 0.00 | | 0 | | 0.17 | | 1 | | 0.00 | | 0 | | 0.00 | | 0 | | 0.00 | | 0 | | 0.00 | | 0 | | 1 | | | 0 | | 1 | |  |  |  |  |
| F8W8M9 | Protein unc-13 homolog B | UNC13B | 1.42 | 1 | | 136.4 | | 7.24 | | 0.00 | 0 | | 0.00 | | 0 | | 0.00 | | 0 | | 0.17 | | 1 | | 0.00 | | 0 | | 0.00 | | 0 | | 0.00 | | 0 | | 0.00 | | 0 | | 1 | | | 0 | | 1 | |  |  |  |  |
| O96028 | Histone-lysine N-methyltransferase NSD2 | NSD2 | 0.88 | 1 | | 152.2 | | 8.69 | | 0.00 | 0 | | 0.00 | | 0 | | 0.00 | | 0 | | 0.17 | | 1 | | 0.00 | | 0 | | 0.00 | | 0 | | 0.00 | | 0 | | 0.00 | | 0 | | 1 | | | 0 | | 1 | |  |  |  |  |
| H7BYJ1 | E3 ubiquitin-protein ligase RNF34 | RNF34 | 7.30 | 1 | | 56.8 | | 5.29 | | 0.00 | 0 | | 0.00 | | 0 | | 0.00 | | 0 | | 0.17 | | 1 | | 0.00 | | 0 | | 0.00 | | 0 | | 0.00 | | 0 | | 0.00 | | 0 | | 1 | | | 0 | | 1 | |  |  |  |  |
| Q4FAT2 | Endothelin-3 | EDN3 | 7.29 | 1 | | 20.6 | | 8.03 | | 0.00 | 0 | | 0.00 | | 0 | | 0.00 | | 0 | | 0.17 | | 1 | | 0.00 | | 0 | | 0.00 | | 0 | | 0.00 | | 0 | | 0.00 | | 0 | | 1 | | | 0 | | 1 | |  |  |  |  |
| Q12767 | Transmembrane protein 94 | TMEM94 | 1.55 | 1 | | 151.1 | | 6.44 | | 0.00 | 0 | | 0.00 | | 0 | | 0.00 | | 0 | | 0.17 | | 1 | | 0.00 | | 0 | | 0.00 | | 0 | | 0.00 | | 0 | | 0.00 | | 0 | | 1 | | | 0 | | 1 | |  |  |  |  |
| Q6UX27 | V-set and transmembrane domain-containing protein 1 | VSTM1 | 8.90 | 1 | | 26.1 | | 5.06 | | 0.00 | 0 | | 0.00 | | 0 | | 0.00 | | 0 | | 0.17 | | 1 | | 0.00 | | 0 | | 0.00 | | 0 | | 0.00 | | 0 | | 0.00 | | 0 | | 1 | | | 0 | | 1 | |  |  |  |  |
| Q2TAY7 | WD40 repeat-containing protein SMU1 | SMU1 | 6.63 | 1 | | 57.5 | | 7.18 | | 0.00 | 0 | | 0.00 | | 0 | | 0.00 | | 0 | | 0.17 | | 1 | | 0.00 | | 0 | | 0.00 | | 0 | | 0.00 | | 0 | | 0.00 | | 0 | | 1 | | | 0 | | 1 | |  |  |  |  |
| A0A087WYG2 | C-Jun-amino-terminal kinase-interacting protein 3 | MAPK8IP3 | 1.72 | 1 | | 147.5 | | 5.40 | | 0.00 | 0 | | 0.00 | | 0 | | 0.00 | | 0 | | 0.17 | | 1 | | 0.00 | | 0 | | 0.00 | | 0 | | 0.00 | | 0 | | 0.00 | | 0 | | 1 | | | 0 | | 1 | |  |  |  |  |
| Q9NNX6 | CD209 antigen | CD209 | 5.69 | 1 | | 45.7 | | 5.47 | | 0.00 | 0 | | 0.00 | | 0 | | 0.00 | | 0 | | 0.17 | | 1 | | 0.00 | | 0 | | 0.00 | | 0 | | 0.00 | | 0 | | 0.00 | | 0 | | 1 | | | 0 | | 1 | |  |  |  |  |
| P56199 | Integrin alpha-1 | ITGA1 | 2.21 | 1 | | 130.8 | | 6.29 | | 0.00 | 0 | | 0.00 | | 0 | | 0.00 | | 0 | | 0.17 | | 1 | | 0.00 | | 0 | | 0.00 | | 0 | | 0.00 | | 0 | | 0.00 | | 0 | | 1 | | | 0 | | 1 | |  |  |  |  |
| Q5VZP5 | Inactive dual specificity phosphatase 27 | DUSP27 | 1.99 | 1 | | 130.1 | | 5.17 | | 0.00 | 0 | | 0.00 | | 0 | | 0.00 | | 0 | | 0.17 | | 1 | | 0.00 | | 0 | | 0.00 | | 0 | | 0.00 | | 0 | | 0.00 | | 0 | | 1 | | | 0 | | 1 | |  |  |  |  |
| Q4VY12 | Phosphatase and actin regulator | PHACTR1 | 9.03 | 1 | | 17.6 | | 9.80 | | 0.00 | 0 | | 0.00 | | 0 | | 0.00 | | 0 | | 0.17 | | 1 | | 0.00 | | 0 | | 0.00 | | 0 | | 0.00 | | 0 | | 0.00 | | 0 | | 1 | | | 0 | | 1 | |  |  |  |  |
| Q9Y2Z4 | Tyrosine--tRNA ligase, mitochondrial | YARS2 | 3.77 | 1 | | 53.2 | | 8.98 | | 0.00 | 0 | | 0.00 | | 0 | | 0.00 | | 0 | | 0.17 | | 1 | | 0.00 | | 0 | | 0.00 | | 0 | | 0.00 | | 0 | | 0.00 | | 0 | | 1 | | | 0 | | 1 | |  |  |  |  |
| A2RTX5 | Threonine--tRNA ligase 2, cytoplasmic | TARS3 | 2.87 | 1 | | 92.6 | | 6.05 | | 0.00 | 0 | | 0.00 | | 0 | | 0.00 | | 0 | | 0.16 | | 1 | | 0.00 | | 0 | | 0.00 | | 0 | | 0.00 | | 0 | | 0.00 | | 0 | | 1 | | | 0 | | 1 | |  |  |  |  |
| Q5T2E6 | Armadillo-like helical domain-containing protein 3 | ARMH3 | 2.32 | 1 | | 78.7 | | 6.60 | | 0.00 | 0 | | 0.00 | | 0 | | 0.00 | | 0 | | 0.16 | | 1 | | 0.00 | | 0 | | 0.00 | | 0 | | 0.00 | | 0 | | 0.00 | | 0 | | 1 | | | 0 | | 1 | |  |  |  |  |
| Q9C0D5 | Protein TANC1 | TANC1 | 1.02 | 1 | | 202.1 | | 8.32 | | 0.00 | 0 | | 0.00 | | 0 | | 0.00 | | 0 | | 0.16 | | 1 | | 0.00 | | 0 | | 0.00 | | 0 | | 0.00 | | 0 | | 0.00 | | 0 | | 1 | | | 0 | | 1 | |  |  |  |  |
| A0A3B3IU55 | Stonin-2 | STON2 | 0.92 | 1 | | 108.9 | | 5.64 | | 0.00 | 0 | | 0.00 | | 0 | | 0.00 | | 0 | | 0.16 | | 1 | | 0.00 | | 0 | | 0.00 | | 0 | | 0.00 | | 0 | | 0.00 | | 0 | | 1 | | | 0 | | 1 | |  |  |  |  |
| B4E1Z4 | cDNA FLJ55673, highly similar to Complement factor B |  | 2.45 | 1 | | 140.9 | | 7.18 | | 0.00 | 0 | | 0.00 | | 0 | | 0.00 | | 0 | | 0.16 | | 1 | | 0.00 | | 0 | | 0.00 | | 0 | | 0.00 | | 0 | | 0.00 | | 0 | | 1 | | | 0 | | 1 | |  |  |  |  |
| Q9ULX6 | A-kinase anchor protein 8-like | AKAP8L | 8.36 | 1 | | 71.6 | | 5.05 | | 0.00 | 0 | | 0.00 | | 0 | | 0.00 | | 0 | | 0.16 | | 1 | | 0.00 | | 0 | | 0.00 | | 0 | | 0.00 | | 0 | | 0.00 | | 0 | | 1 | | | 0 | | 1 | |  |  |  |  |
| E7EW32 | Vacuolar fusion protein MON1 homolog B | MON1B | 12.10 | 1 | | 47.8 | | 6.54 | | 0.00 | 0 | | 0.00 | | 0 | | 0.00 | | 0 | | 0.16 | | 1 | | 0.00 | | 0 | | 0.00 | | 0 | | 0.00 | | 0 | | 0.00 | | 0 | | 1 | | | 0 | | 1 | |  |  |  |  |
| Q9Y5H1 | Protocadherin gamma-A2 | PCDHGA2 | 2.04 | 1 | | 101.4 | | 5.01 | | 0.00 | 0 | | 0.00 | | 0 | | 0.00 | | 0 | | 0.16 | | 1 | | 0.00 | | 0 | | 0.00 | | 0 | | 0.00 | | 0 | | 0.00 | | 0 | | 1 | | | 0 | | 1 | |  |  |  |  |
| B8ZZ84 | Leucine-rich repeat transmembrane neuronal protein 4 | LRRTM4 | 5.75 | 1 | | 67.3 | | 8.95 | | 0.00 | 0 | | 0.00 | | 0 | | 0.00 | | 0 | | 0.16 | | 1 | | 0.00 | | 0 | | 0.00 | | 0 | | 0.00 | | 0 | | 0.00 | | 0 | | 1 | | | 0 | | 1 | |  |  |  |  |
| A0A338VFP5 | Golgin subfamily A member 6-like protein 2 | GOLGA6L2 | 4.59 | 1 | | 48.1 | | 8.03 | | 0.00 | 0 | | 0.00 | | 0 | | 0.00 | | 0 | | 0.16 | | 1 | | 0.00 | | 0 | | 0.00 | | 0 | | 0.00 | | 0 | | 0.00 | | 0 | | 1 | | | 0 | | 1 | |  |  |  |  |
| O43150 | Arf-GAP with SH3 domain, ANK repeat and PH domain-containing protein 2 | ASAP2 | 3.18 | 1 | | 111.6 | | 6.68 | | 0.00 | 0 | | 0.00 | | 0 | | 0.00 | | 0 | | 0.16 | | 1 | | 0.00 | | 0 | | 0.00 | | 0 | | 0.00 | | 0 | | 0.00 | | 0 | | 1 | | | 0 | | 1 | |  |  |  |  |
| Q5SYB0 | FERM and PDZ domain-containing protein 1 | FRMPD1 | 1.46 | 1 | | 173.3 | | 5.25 | | 0.00 | 0 | | 0.00 | | 0 | | 0.00 | | 0 | | 0.16 | | 1 | | 0.00 | | 0 | | 0.00 | | 0 | | 0.00 | | 0 | | 0.00 | | 0 | | 1 | | | 0 | | 1 | |  |  |  |  |
| A0A2C9F2M1 | Olfactory receptor | OR7G2 | 7.54 | 1 | | 38.4 | | 8.15 | | 0.00 | 0 | | 0.00 | | 0 | | 0.00 | | 0 | | 0.16 | | 1 | | 0.00 | | 0 | | 0.00 | | 0 | | 0.00 | | 0 | | 0.00 | | 0 | | 1 | | | 0 | | 1 | |  |  |  |  |
| Q6Q759 | Sperm-associated antigen 17 | SPAG17 | 3.15 | 1 | | 251.6 | | 6.24 | | 0.00 | 0 | | 0.00 | | 0 | | 0.00 | | 0 | | 0.16 | | 1 | | 0.00 | | 0 | | 0.00 | | 0 | | 0.00 | | 0 | | 0.00 | | 0 | | 1 | | | 0 | | 1 | |  |  |  |  |
| Q9Y3D3 | 28S ribosomal protein S16, mitochondrial | MRPS16 | 16.79 | 1 | | 15.3 | | 9.50 | | 0.00 | 0 | | 0.00 | | 0 | | 0.00 | | 0 | | 0.16 | | 1 | | 0.00 | | 0 | | 0.00 | | 0 | | 0.00 | | 0 | | 0.00 | | 0 | | 1 | | | 0 | | 1 | |  |  |  |  |
| A6XGL3 | Protease serine 1 | PRSS1 | 5.49 | 1 | | 25.4 | | 7.55 | | 0.00 | 0 | | 0.00 | | 0 | | 0.00 | | 0 | | 0.16 | | 1 | | 0.00 | | 0 | | 0.00 | | 0 | | 0.00 | | 0 | | 0.00 | | 0 | | 1 | | | 0 | | 1 | |  |  |  |  |
| Q6JQN1 | Acyl-CoA dehydrogenase family member 10 | ACAD10 | 1.79 | 1 | | 118.8 | | 8.06 | | 0.00 | 0 | | 0.00 | | 0 | | 0.00 | | 0 | | 0.16 | | 1 | | 0.00 | | 0 | | 0.00 | | 0 | | 0.00 | | 0 | | 0.00 | | 0 | | 1 | | | 0 | | 1 | |  |  |  |  |
| Q149M9 | NACHT domain- and WD repeat-containing protein 1 | NWD1 | 2.17 | 1 | | 174.4 | | 6.74 | | 0.00 | 0 | | 0.00 | | 0 | | 0.00 | | 0 | | 0.16 | | 1 | | 0.00 | | 0 | | 0.00 | | 0 | | 0.00 | | 0 | | 0.00 | | 0 | | 1 | | | 0 | | 1 | |  |  |  |  |
| Q8TBZ5 | Zinc finger protein 502 | ZNF502 | 6.43 | 1 | | 62.9 | | 8.69 | | 0.00 | 0 | | 0.00 | | 0 | | 0.00 | | 0 | | 0.16 | | 1 | | 0.00 | | 0 | | 0.00 | | 0 | | 0.00 | | 0 | | 0.00 | | 0 | | 1 | | | 0 | | 1 | |  |  |  |  |
| E7ESC7 | E3 ubiquitin-protein transferase MAEA | MAEA | 5.30 | 1 | | 49.4 | | 8.98 | | 0.00 | 0 | | 0.00 | | 0 | | 0.00 | | 0 | | 0.16 | | 1 | | 0.00 | | 0 | | 0.00 | | 0 | | 0.00 | | 0 | | 0.00 | | 0 | | 1 | | | 0 | | 1 | |  |  |  |  |
| B2RV13 | Uncharacterized protein CFAP97D1 | CFAP97D1 | 11.59 | 1 | | 19.5 | | 10.07 | | 0.00 | 0 | | 0.00 | | 0 | | 0.00 | | 0 | | 0.16 | | 1 | | 0.00 | | 0 | | 0.00 | | 0 | | 0.00 | | 0 | | 0.00 | | 0 | | 1 | | | 0 | | 1 | |  |  |  |  |
| E9PP44 | Protein phosphatase 1A | PPM1A | 20.12 | 1 | | 19.0 | | 10.17 | | 0.00 | 0 | | 0.00 | | 0 | | 0.00 | | 0 | | 0.16 | | 1 | | 0.00 | | 0 | | 0.00 | | 0 | | 0.00 | | 0 | | 0.00 | | 0 | | 1 | | | 0 | | 1 | |  |  |  |  |
| P43897 | Elongation factor Ts, mitochondrial | TSFM | 15.38 | 1 | | 35.4 | | 8.38 | | 0.00 | 0 | | 0.00 | | 0 | | 0.00 | | 0 | | 0.16 | | 1 | | 0.00 | | 0 | | 0.00 | | 0 | | 0.00 | | 0 | | 0.00 | | 0 | | 1 | | | 0 | | 1 | |  |  |  |  |
| Q6ZMT4 | Lysine-specific demethylase 7A | KDM7A | 3.29 | 1 | | 106.5 | | 8.06 | | 0.00 | 0 | | 0.00 | | 0 | | 0.00 | | 0 | | 0.16 | | 1 | | 0.00 | | 0 | | 0.00 | | 0 | | 0.00 | | 0 | | 0.00 | | 0 | | 1 | | | 0 | | 1 | |  |  |  |  |
| H0Y3Z0 | Potassium voltage-gated channel subfamily KQT member 5 (Fragment) | KCNQ5 | 11.34 | 1 | | 10.9 | | 11.00 | | 0.00 | 0 | | 0.00 | | 0 | | 0.00 | | 0 | | 0.16 | | 1 | | 0.00 | | 0 | | 0.00 | | 0 | | 0.00 | | 0 | | 0.00 | | 0 | | 1 | | | 0 | | 1 | |  |  |  |  |
| A6PVC2 | Protein monoglycylase TTLL8 | TTLL8 | 2.71 | 1 | | 94.6 | | 8.21 | | 0.00 | 0 | | 0.00 | | 0 | | 0.00 | | 0 | | 0.16 | | 1 | | 0.00 | | 0 | | 0.00 | | 0 | | 0.00 | | 0 | | 0.00 | | 0 | | 1 | | | 0 | | 1 | |  |  |  |  |
| P16219 | Short-chain specific acyl-CoA dehydrogenase, mitochondrial | ACADS | 1.70 | 1 | | 44.3 | | 7.99 | | 0.00 | 0 | | 0.00 | | 0 | | 0.00 | | 0 | | 0.16 | | 1 | | 0.00 | | 0 | | 0.00 | | 0 | | 0.00 | | 0 | | 0.00 | | 0 | | 1 | | | 0 | | 1 | |  |  |  |  |
| Q5SV97 | PGC-1 and ERR-induced regulator in muscle protein 1 | PERM1 | 6.96 | 1 | | 81.3 | | 5.58 | | 0.00 | 0 | | 0.00 | | 0 | | 0.00 | | 0 | | 0.16 | | 1 | | 0.00 | | 0 | | 0.00 | | 0 | | 0.00 | | 0 | | 0.00 | | 0 | | 1 | | | 0 | | 1 | |  |  |  |  |
| Q8NEB7 | Acrosin-binding protein | ACRBP | 2.03 | 1 | | 61.3 | | 5.16 | | 0.00 | 0 | | 0.00 | | 0 | | 0.00 | | 0 | | 0.16 | | 1 | | 0.00 | | 0 | | 0.00 | | 0 | | 0.00 | | 0 | | 0.00 | | 0 | | 1 | | | 0 | | 1 | |  |  |  |  |
| A0A0G2JJQ8 | HLA class II histocompatibility antigen, DR beta 4 chain | HLA-DRB4 | 10.53 | 1 | | 30.0 | | 7.33 | | 0.00 | 0 | | 0.00 | | 0 | | 0.00 | | 0 | | 0.16 | | 1 | | 0.00 | | 0 | | 0.00 | | 0 | | 0.00 | | 0 | | 0.00 | | 0 | | 1 | | | 0 | | 1 | |  |  |  |  |
| H0YAS1 | Fatty acid-binding protein 12 (Fragment) | FABP12 | 14.44 | 1 | | 10.3 | | 10.05 | | 0.00 | 0 | | 0.00 | | 0 | | 0.00 | | 0 | | 0.16 | | 1 | | 0.00 | | 0 | | 0.00 | | 0 | | 0.00 | | 0 | | 0.00 | | 0 | | 1 | | | 0 | | 1 | |  |  |  |  |
| B4DV59 | REST corepressor 3 | RCOR3 | 9.89 | 1 | | 41.8 | | 7.02 | | 0.00 | 0 | | 0.00 | | 0 | | 0.00 | | 0 | | 0.16 | | 1 | | 0.00 | | 0 | | 0.00 | | 0 | | 0.00 | | 0 | | 0.00 | | 0 | | 1 | | | 0 | | 1 | |  |  |  |  |
| P27469 | G0/G1 switch protein 2 | G0S2 | 8.74 | 1 | | 11.3 | | 9.72 | | 0.00 | 0 | | 0.00 | | 0 | | 0.00 | | 0 | | 0.16 | | 1 | | 0.00 | | 0 | | 0.00 | | 0 | | 0.00 | | 0 | | 0.00 | | 0 | | 1 | | | 0 | | 1 | |  |  |  |  |
| Q13434 | Putative E3 ubiquitin-protein ligase makorin-4 | MKRN4P | 2.06 | 1 | | 52.9 | | 5.16 | | 0.00 | 0 | | 0.00 | | 0 | | 0.00 | | 0 | | 0.16 | | 1 | | 0.00 | | 0 | | 0.00 | | 0 | | 0.00 | | 0 | | 0.00 | | 0 | | 1 | | | 0 | | 1 | |  |  |  |  |
| Q9NSD7 | Relaxin-3 receptor 1 | RXFP3 | 8.32 | 1 | | 51.1 | | 8.79 | | 0.00 | 0 | | 0.00 | | 0 | | 0.00 | | 0 | | 0.16 | | 1 | | 0.00 | | 0 | | 0.00 | | 0 | | 0.00 | | 0 | | 0.00 | | 0 | | 1 | | | 0 | | 1 | |  |  |  |  |
| Q9UKX5 | Integrin alpha-11 | ITGA11 | 2.69 | 1 | | 133.4 | | 6.70 | | 0.00 | 0 | | 0.00 | | 0 | | 0.00 | | 0 | | 0.16 | | 1 | | 0.00 | | 0 | | 0.00 | | 0 | | 0.00 | | 0 | | 0.00 | | 0 | | 1 | | | 0 | | 1 | |  |  |  |  |
| O14874 | [3-methyl-2-oxobutanoate dehydrogenase [lipoamide]] kinase, mitochondrial | BCKDK | 4.13 | 1 | | 46.3 | | 8.82 | | 0.00 | 0 | | 0.00 | | 0 | | 0.00 | | 0 | | 0.16 | | 1 | | 0.00 | | 0 | | 0.00 | | 0 | | 0.00 | | 0 | | 0.00 | | 0 | | 1 | | | 0 | | 1 | |  |  |  |  |
| Q9GZY8 | Mitochondrial fission factor | MFF | 10.53 | 1 | | 38.4 | | 8.95 | | 0.00 | 0 | | 0.00 | | 0 | | 0.00 | | 0 | | 0.16 | | 1 | | 0.00 | | 0 | | 0.00 | | 0 | | 0.00 | | 0 | | 0.00 | | 0 | | 1 | | | 0 | | 1 | |  |  |  |  |
| K7EQZ0 | Protein phosphatase Slingshot homolog 2 | SSH2 | 59.52 | 1 | | 4.5 | | 11.55 | | 0.00 | 0 | | 0.00 | | 0 | | 0.00 | | 0 | | 0.16 | | 1 | | 0.00 | | 0 | | 0.00 | | 0 | | 0.00 | | 0 | | 0.00 | | 0 | | 1 | | | 0 | | 1 | |  |  |  |  |
| Q9NZN5 | Rho guanine nucleotide exchange factor 12 | ARHGEF12 | 3.63 | 1 | | 173.1 | | 5.74 | | 0.00 | 0 | | 0.00 | | 0 | | 0.00 | | 0 | | 0.16 | | 1 | | 0.00 | | 0 | | 0.00 | | 0 | | 0.00 | | 0 | | 0.00 | | 0 | | 1 | | | 0 | | 1 | |  |  |  |  |
| Q8NEP3 | Dynein assembly factor 1, axonemal | DNAAF1 | 2.48 | 1 | | 80.0 | | 4.67 | | 0.00 | 0 | | 0.00 | | 0 | | 0.00 | | 0 | | 0.16 | | 1 | | 0.00 | | 0 | | 0.00 | | 0 | | 0.00 | | 0 | | 0.00 | | 0 | | 1 | | | 0 | | 1 | |  |  |  |  |
| Q15013 | MAD2L1-binding protein | MAD2L1BP | 20.44 | 1 | | 31.0 | | 6.30 | | 0.00 | 0 | | 0.00 | | 0 | | 0.00 | | 0 | | 0.16 | | 1 | | 0.00 | | 0 | | 0.00 | | 0 | | 0.00 | | 0 | | 0.00 | | 0 | | 1 | | | 0 | | 1 | |  |  |  |  |
| G3V3J7 | F-box only protein 33 | FBXO33 | 19.10 | 1 | | 9.6 | | 6.27 | | 0.00 | 0 | | 0.00 | | 0 | | 0.00 | | 0 | | 0.16 | | 1 | | 0.00 | | 0 | | 0.00 | | 0 | | 0.00 | | 0 | | 0.00 | | 0 | | 1 | | | 0 | | 1 | |  |  |  |  |
| F5GWR3 | Matrix metalloproteinase-17 (Fragment) | MMP17 | 19.13 | 1 | | 13.0 | | 9.25 | | 0.00 | 0 | | 0.00 | | 0 | | 0.00 | | 0 | | 0.16 | | 1 | | 0.00 | | 0 | | 0.00 | | 0 | | 0.00 | | 0 | | 0.00 | | 0 | | 1 | | | 0 | | 1 | |  |  |  |  |
| Q5XPI4 | E3 ubiquitin-protein ligase RNF123 | RNF123 | 5.02 | 1 | | 148.4 | | 6.74 | | 0.00 | 0 | | 0.00 | | 0 | | 0.00 | | 0 | | 0.16 | | 1 | | 0.00 | | 0 | | 0.00 | | 0 | | 0.00 | | 0 | | 0.00 | | 0 | | 1 | | | 0 | | 1 | |  |  |  |  |
| O60662 | Kelch-like protein 41 | KLHL41 | 4.13 | 1 | | 68.0 | | 5.29 | | 0.00 | 0 | | 0.00 | | 0 | | 0.00 | | 0 | | 0.16 | | 1 | | 0.00 | | 0 | | 0.00 | | 0 | | 0.00 | | 0 | | 0.00 | | 0 | | 1 | | | 0 | | 1 | |  |  |  |  |
| Q96RG2 | PAS domain-containing serine/threonine-protein kinase | PASK | 4.46 | 1 | | 142.8 | | 4.86 | | 0.00 | 0 | | 0.00 | | 0 | | 0.00 | | 0 | | 0.16 | | 1 | | 0.00 | | 0 | | 0.00 | | 0 | | 0.00 | | 0 | | 0.00 | | 0 | | 1 | | | 0 | | 1 | |  |  |  |  |
| A0A3B3IRZ4 | IQ motif and SEC7 domain-containing protein 1 | IQSEC1 | 2.05 | 1 | | 136.0 | | 8.47 | | 0.00 | 0 | | 0.00 | | 0 | | 0.00 | | 0 | | 0.16 | | 1 | | 0.00 | | 0 | | 0.00 | | 0 | | 0.00 | | 0 | | 0.00 | | 0 | | 1 | | | 0 | | 1 | |  |  |  |  |
| Q9NWL6 | Asparagine synthetase domain-containing protein 1 | ASNSD1 | 2.49 | 1 | | 72.0 | | 6.83 | | 0.00 | 0 | | 0.00 | | 0 | | 0.00 | | 0 | | 0.16 | | 1 | | 0.00 | | 0 | | 0.00 | | 0 | | 0.00 | | 0 | | 0.00 | | 0 | | 1 | | | 0 | | 1 | |  |  |  |  |
| P22681 | E3 ubiquitin-protein ligase CBL | CBL | 3.64 | 1 | | 99.6 | | 6.54 | | 0.00 | 0 | | 0.00 | | 0 | | 0.00 | | 0 | | 0.16 | | 1 | | 0.00 | | 0 | | 0.00 | | 0 | | 0.00 | | 0 | | 0.00 | | 0 | | 1 | | | 0 | | 1 | |  |  |  |  |
| P30281 | G1/S-specific cyclin-D3 | CCND3 | 12.67 | 1 | | 32.5 | | 7.06 | | 0.00 | 0 | | 0.00 | | 0 | | 0.00 | | 0 | | 0.16 | | 1 | | 0.00 | | 0 | | 0.00 | | 0 | | 0.00 | | 0 | | 0.00 | | 0 | | 1 | | | 0 | | 1 | |  |  |  |  |
| E5RJ46 | Uncharacterized protein C8orf87 | C8orf87 | 13.86 | 1 | | 11.4 | | 11.37 | | 0.00 | 0 | | 0.00 | | 0 | | 0.00 | | 0 | | 0.16 | | 1 | | 0.00 | | 0 | | 0.00 | | 0 | | 0.00 | | 0 | | 0.00 | | 0 | | 1 | | | 0 | | 1 | |  |  |  |  |
| F2Z3C9 | Granulocyte-macrophage colony-stimulating factor receptor subunit alpha | CSF2RA | 9.12 | 1 | | 32.8 | | 8.47 | | 0.00 | 0 | | 0.00 | | 0 | | 0.00 | | 0 | | 0.16 | | 1 | | 0.00 | | 0 | | 0.00 | | 0 | | 0.00 | | 0 | | 0.00 | | 0 | | 1 | | | 0 | | 1 | |  |  |  |  |
| O95373 | Importin-7 | IPO7 | 3.85 | 1 | | 119.4 | | 4.82 | | 0.00 | 0 | | 0.00 | | 0 | | 0.00 | | 0 | | 0.16 | | 1 | | 0.00 | | 0 | | 0.00 | | 0 | | 0.00 | | 0 | | 0.00 | | 0 | | 1 | | | 0 | | 1 | |  |  |  |  |
| P54132 | Bloom syndrome protein | BLM | 3.03 | 1 | | 158.9 | | 7.49 | | 0.00 | 0 | | 0.00 | | 0 | | 0.00 | | 0 | | 0.16 | | 1 | | 0.00 | | 0 | | 0.00 | | 0 | | 0.00 | | 0 | | 0.00 | | 0 | | 1 | | | 0 | | 1 | |  |  |  |  |
| O00548 | Delta-like protein 1 | DLL1 | 5.53 | 1 | | 78.0 | | 6.24 | | 0.00 | 0 | | 0.00 | | 0 | | 0.00 | | 0 | | 0.16 | | 1 | | 0.00 | | 0 | | 0.00 | | 0 | | 0.00 | | 0 | | 0.00 | | 0 | | 1 | | | 0 | | 1 | |  |  |  |  |
| Q12889 | Oviduct-specific glycoprotein | OVGP1 | 5.31 | 1 | | 75.4 | | 8.78 | | 0.00 | 0 | | 0.00 | | 0 | | 0.00 | | 0 | | 0.16 | | 1 | | 0.00 | | 0 | | 0.00 | | 0 | | 0.00 | | 0 | | 0.00 | | 0 | | 1 | | | 0 | | 1 | |  |  |  |  |
| Q92917 | G-patch domain and KOW motifs-containing protein | GPKOW | 7.56 | 1 | | 52.2 | | 6.15 | | 0.00 | 0 | | 0.00 | | 0 | | 0.00 | | 0 | | 0.16 | | 1 | | 0.00 | | 0 | | 0.00 | | 0 | | 0.00 | | 0 | | 0.00 | | 0 | | 1 | | | 0 | | 1 | |  |  |  |  |
| P57060 | RWD domain-containing protein 2B | RWDD2B | 2.19 | 1 | | 36.3 | | 5.87 | | 0.00 | 0 | | 0.00 | | 0 | | 0.00 | | 0 | | 0.16 | | 1 | | 0.00 | | 0 | | 0.00 | | 0 | | 0.00 | | 0 | | 0.00 | | 0 | | 1 | | | 0 | | 1 | |  |  |  |  |
| Q5W026 | Ankyrin repeat domain-containing protein 30A | ANKRD30A | 1.85 | 1 | | 171.9 | | 6.33 | | 0.00 | 0 | | 0.00 | | 0 | | 0.00 | | 0 | | 0.16 | | 1 | | 0.00 | | 0 | | 0.00 | | 0 | | 0.00 | | 0 | | 0.00 | | 0 | | 1 | | | 0 | | 1 | |  |  |  |  |
| P51523 | Zinc finger protein 84 | ZNF84 | 3.93 | 1 | | 85.4 | | 8.78 | | 0.00 | 0 | | 0.00 | | 0 | | 0.00 | | 0 | | 0.16 | | 1 | | 0.00 | | 0 | | 0.00 | | 0 | | 0.00 | | 0 | | 0.00 | | 0 | | 1 | | | 0 | | 1 | |  |  |  |  |
| E9PMC9 | BRCA2-interacting transcriptional repressor EMSY | EMSY | 2.12 | 1 | | 130.6 | | 9.28 | | 0.00 | 0 | | 0.00 | | 0 | | 0.00 | | 0 | | 0.16 | | 1 | | 0.00 | | 0 | | 0.00 | | 0 | | 0.00 | | 0 | | 0.00 | | 0 | | 1 | | | 0 | | 1 | |  |  |  |  |
| Q96BZ9 | TBC1 domain family member 20 | TBC1D20 | 4.22 | 1 | | 45.8 | | 6.86 | | 0.00 | 0 | | 0.00 | | 0 | | 0.00 | | 0 | | 0.16 | | 1 | | 0.00 | | 0 | | 0.00 | | 0 | | 0.00 | | 0 | | 0.00 | | 0 | | 1 | | | 0 | | 1 | |  |  |  |  |
| Q86UU0 | B-cell CLL/lymphoma 9-like protein | BCL9L | 3.80 | 1 | | 157.0 | | 8.63 | | 0.00 | 0 | | 0.00 | | 0 | | 0.00 | | 0 | | 0.16 | | 1 | | 0.00 | | 0 | | 0.00 | | 0 | | 0.00 | | 0 | | 0.00 | | 0 | | 1 | | | 0 | | 1 | |  |  |  |  |
| A6NDU8 | UPF0600 protein C5orf51 | C5orf51 | 9.18 | 1 | | 33.6 | | 5.26 | | 0.00 | 0 | | 0.00 | | 0 | | 0.00 | | 0 | | 0.16 | | 1 | | 0.00 | | 0 | | 0.00 | | 0 | | 0.00 | | 0 | | 0.00 | | 0 | | 1 | | | 0 | | 1 | |  |  |  |  |
| Q86XS5 | Angiopoietin-related protein 5 | ANGPTL5 | 12.11 | 1 | | 44.1 | | 6.60 | | 0.00 | 0 | | 0.00 | | 0 | | 0.00 | | 0 | | 0.16 | | 1 | | 0.00 | | 0 | | 0.00 | | 0 | | 0.00 | | 0 | | 0.00 | | 0 | | 1 | | | 0 | | 1 | |  |  |  |  |
| Q8NG77 | Olfactory receptor 2T12 | OR2T12 | 15.00 | 1 | | 35.9 | | 8.65 | | 0.00 | 0 | | 0.00 | | 0 | | 0.00 | | 0 | | 0.16 | | 1 | | 0.00 | | 0 | | 0.00 | | 0 | | 0.00 | | 0 | | 0.00 | | 0 | | 1 | | | 0 | | 1 | |  |  |  |  |
| Q8TBE0 | Bromo adjacent homology domain-containing 1 protein | BAHD1 | 3.72 | 1 | | 84.6 | | 9.07 | | 0.00 | 0 | | 0.00 | | 0 | | 0.00 | | 0 | | 0.16 | | 1 | | 0.00 | | 0 | | 0.00 | | 0 | | 0.00 | | 0 | | 0.00 | | 0 | | 1 | | | 0 | | 1 | |  |  |  |  |
| Q8N5F7 | NF-kappa-B-activating protein | NKAP | 5.06 | 1 | | 47.1 | | 10.11 | | 0.00 | 0 | | 0.00 | | 0 | | 0.00 | | 0 | | 0.16 | | 1 | | 0.00 | | 0 | | 0.00 | | 0 | | 0.00 | | 0 | | 0.00 | | 0 | | 1 | | | 0 | | 1 | |  |  |  |  |
| Q5VTL8 | Pre-mRNA-splicing factor 38B | PRPF38B | 1.28 | 1 | | 64.4 | | 10.54 | | 0.00 | 0 | | 0.00 | | 0 | | 0.00 | | 0 | | 0.16 | | 1 | | 0.00 | | 0 | | 0.00 | | 0 | | 0.00 | | 0 | | 0.00 | | 0 | | 1 | | | 0 | | 1 | |  |  |  |  |
| D6REB7 | Succinate dehydrogenase [ubiquinone] flavoprotein subunit, mitochondrial | SDHA | 21.35 | 1 | | 10.1 | | 10.21 | | 0.00 | 0 | | 0.00 | | 0 | | 0.00 | | 0 | | 0.16 | | 1 | | 0.00 | | 0 | | 0.00 | | 0 | | 0.00 | | 0 | | 0.00 | | 0 | | 1 | | | 0 | | 1 | |  |  |  |  |
| Q9Y4B6 | DDB1- and CUL4-associated factor 1 | DCAF1 | 1.92 | 1 | | 168.9 | | 5.06 | | 0.00 | 0 | | 0.00 | | 0 | | 0.00 | | 0 | | 0.16 | | 1 | | 0.00 | | 0 | | 0.00 | | 0 | | 0.00 | | 0 | | 0.00 | | 0 | | 1 | | | 0 | | 1 | |  |  |  |  |
| O60296 | Trafficking kinesin-binding protein 2 | TRAK2 | 3.39 | 1 | | 101.4 | | 5.24 | | 0.00 | 0 | | 0.00 | | 0 | | 0.00 | | 0 | | 0.16 | | 1 | | 0.00 | | 0 | | 0.00 | | 0 | | 0.00 | | 0 | | 0.00 | | 0 | | 1 | | | 0 | | 1 | |  |  |  |  |
| Q64LD2 | WD repeat-containing protein 25 | WDR25 | 6.07 | 1 | | 60.1 | | 9.00 | | 0.00 | 0 | | 0.00 | | 0 | | 0.00 | | 0 | | 0.16 | | 1 | | 0.00 | | 0 | | 0.00 | | 0 | | 0.00 | | 0 | | 0.00 | | 0 | | 1 | | | 0 | | 1 | |  |  |  |  |
| Q9UJW2 | Tubulointerstitial nephritis antigen | TINAG | 2.94 | 1 | | 54.6 | | 7.97 | | 0.00 | 0 | | 0.00 | | 0 | | 0.00 | | 0 | | 0.16 | | 1 | | 0.00 | | 0 | | 0.00 | | 0 | | 0.00 | | 0 | | 0.00 | | 0 | | 1 | | | 0 | | 1 | |  |  |  |  |
| P07307 | Asialoglycoprotein receptor 2 | ASGR2 | 3.54 | 1 | | 35.1 | | 6.25 | | 0.00 | 0 | | 0.00 | | 0 | | 0.00 | | 0 | | 0.16 | | 1 | | 0.00 | | 0 | | 0.00 | | 0 | | 0.00 | | 0 | | 0.00 | | 0 | | 1 | | | 0 | | 1 | |  |  |  |  |
| Q9Y5A6 | Zinc finger and SCAN domain-containing protein 21 | ZSCAN21 | 4.86 | 1 | | 53.6 | | 7.72 | | 0.00 | 0 | | 0.00 | | 0 | | 0.00 | | 0 | | 0.16 | | 1 | | 0.00 | | 0 | | 0.00 | | 0 | | 0.00 | | 0 | | 0.00 | | 0 | | 1 | | | 0 | | 1 | |  |  |  |  |
| Q9H4I0 | Double-strand-break repair protein rad21-like protein 1 | RAD21L1 | 1.80 | 1 | | 63.3 | | 5.16 | | 0.00 | 0 | | 0.00 | | 0 | | 0.00 | | 0 | | 0.16 | | 1 | | 0.00 | | 0 | | 0.00 | | 0 | | 0.00 | | 0 | | 0.00 | | 0 | | 1 | | | 0 | | 1 | |  |  |  |  |
| H0Y858 | Uncharacterized protein (Fragment) |  | 1.26 | 1 | | 133.5 | | 8.09 | | 0.00 | 0 | | 0.00 | | 0 | | 0.00 | | 0 | | 0.16 | | 1 | | 0.00 | | 0 | | 0.00 | | 0 | | 0.00 | | 0 | | 0.00 | | 0 | | 1 | | | 0 | | 1 | |  |  |  |  |
| P30414 | NK-tumor recoition protein | NKTR | 2.46 | 1 | | 165.6 | | 9.99 | | 0.00 | 0 | | 0.00 | | 0 | | 0.00 | | 0 | | 0.16 | | 1 | | 0.00 | | 0 | | 0.00 | | 0 | | 0.00 | | 0 | | 0.00 | | 0 | | 1 | | | 0 | | 1 | |  |  |  |  |
| Q12888 | TP53-binding protein 1 | TP53BP1 | 1.37 | 1 | | 213.4 | | 4.70 | | 0.00 | 0 | | 0.00 | | 0 | | 0.00 | | 0 | | 0.16 | | 1 | | 0.00 | | 0 | | 0.00 | | 0 | | 0.00 | | 0 | | 0.00 | | 0 | | 1 | | | 0 | | 1 | |  |  |  |  |
| Q9BWT1 | Cell division cycle-associated protein 7 | CDCA7 | 8.63 | 1 | | 42.5 | | 9.45 | | 0.00 | 0 | | 0.00 | | 0 | | 0.00 | | 0 | | 0.16 | | 1 | | 0.00 | | 0 | | 0.00 | | 0 | | 0.00 | | 0 | | 0.00 | | 0 | | 1 | | | 0 | | 1 | |  |  |  |  |
| A4D263 | Spermatogenesis-associated protein 48 | SPATA48 | 5.94 | 1 | | 49.6 | | 8.48 | | 0.00 | 0 | | 0.00 | | 0 | | 0.00 | | 0 | | 0.16 | | 1 | | 0.00 | | 0 | | 0.00 | | 0 | | 0.00 | | 0 | | 0.00 | | 0 | | 1 | | | 0 | | 1 | |  |  |  |  |
| Q9BX69 | Caspase recruitment domain-containing protein 6 | CARD6 | 2.60 | 1 | | 116.4 | | 6.37 | | 0.00 | 0 | | 0.00 | | 0 | | 0.00 | | 0 | | 0.16 | | 1 | | 0.00 | | 0 | | 0.00 | | 0 | | 0.00 | | 0 | | 0.00 | | 0 | | 1 | | | 0 | | 1 | |  |  |  |  |
| Q6GPH4 | XIAP-associated factor 1 | XAF1 | 6.98 | 1 | | 34.6 | | 8.19 | | 0.00 | 0 | | 0.00 | | 0 | | 0.00 | | 0 | | 0.16 | | 1 | | 0.00 | | 0 | | 0.00 | | 0 | | 0.00 | | 0 | | 0.00 | | 0 | | 1 | | | 0 | | 1 | |  |  |  |  |
| C9JQI7 | Transmembrane protein 232 | TMEM232 | 1.07 | 1 | | 76.4 | | 8.06 | | 0.00 | 0 | | 0.00 | | 0 | | 0.00 | | 0 | | 0.16 | | 1 | | 0.00 | | 0 | | 0.00 | | 0 | | 0.00 | | 0 | | 0.00 | | 0 | | 1 | | | 0 | | 1 | |  |  |  |  |
| A6NDG6 | Glycerol-3-phosphate phosphatase | PGP | 3.12 | 1 | | 34.0 | | 6.14 | | 0.00 | 0 | | 0.00 | | 0 | | 0.00 | | 0 | | 0.16 | | 1 | | 0.00 | | 0 | | 0.00 | | 0 | | 0.00 | | 0 | | 0.00 | | 0 | | 1 | | | 0 | | 1 | |  |  |  |  |
| Q9NTQ9 | Gap junction beta-4 protein | GJB4 | 10.90 | 1 | | 30.4 | | 8.09 | | 0.00 | 0 | | 0.00 | | 0 | | 0.00 | | 0 | | 0.16 | | 1 | | 0.00 | | 0 | | 0.00 | | 0 | | 0.00 | | 0 | | 0.00 | | 0 | | 1 | | | 0 | | 1 | |  |  |  |  |
| Q96JN2 | Coiled-coil domain-containing protein 136 | CCDC136 | 1.99 | 1 | | 134.0 | | 4.65 | | 0.00 | 0 | | 0.00 | | 0 | | 0.00 | | 0 | | 0.16 | | 1 | | 0.00 | | 0 | | 0.00 | | 0 | | 0.00 | | 0 | | 0.00 | | 0 | | 1 | | | 0 | | 1 | |  |  |  |  |
| Q2TAL5 | Smoothelin-like protein 2 | SMTNL2 | 9.54 | 1 | | 50.2 | | 8.56 | | 0.00 | 0 | | 0.00 | | 0 | | 0.00 | | 0 | | 0.16 | | 1 | | 0.00 | | 0 | | 0.00 | | 0 | | 0.00 | | 0 | | 0.00 | | 0 | | 1 | | | 0 | | 1 | |  |  |  |  |
| B1ALY0 | PALM2 and AKAP2 fusion (Fragment) | PALM2AKAP2 | 12.70 | 1 | | 47.6 | | 4.89 | | 0.00 | 0 | | 0.00 | | 0 | | 0.00 | | 0 | | 0.16 | | 1 | | 0.00 | | 0 | | 0.00 | | 0 | | 0.00 | | 0 | | 0.00 | | 0 | | 1 | | | 0 | | 1 | |  |  |  |  |
| H7C4T8 | Tyrosine-protein phosphatase non-receptor type 7 (Fragment) | | | | PTPN7 | | 19.67 | | 1 | | | 14.3 | | 4.98 | | 0.00 | | 0 | | 0.00 | | 0 | | 0.00 | | 0 | | 0.16 | | 1 | | 0.00 | | 0 | | 0.00 | | 0 | | 0.00 | | 0 | 0.00 | | 0 | | 1 | | | 0 | 1 |
| E7ESC6 | Exportin-7 | | | | XPO7 | | 0.83 | | 1 | | | 124.0 | | 6.48 | | 0.00 | | 0 | | 0.00 | | 0 | | 0.00 | | 0 | | 0.16 | | 1 | | 0.00 | | 0 | | 0.00 | | 0 | | 0.00 | | 0 | 0.00 | | 0 | | 1 | | | 0 | 1 |
| P29376 | Leukocyte tyrosine kinase receptor | | | | LTK | | 4.05 | | 1 | | | 91.6 | | 6.49 | | 0.00 | | 0 | | 0.00 | | 0 | | 0.00 | | 0 | | 0.16 | | 1 | | 0.00 | | 0 | | 0.00 | | 0 | | 0.00 | | 0 | 0.00 | | 0 | | 1 | | | 0 | 1 |
| Q86W25 | NACHT, LRR and PYD domains-containing protein 13 | | | | NLRP13 | | 2.49 | | 1 | | | 118.8 | | 5.66 | | 0.00 | | 0 | | 0.00 | | 0 | | 0.00 | | 0 | | 0.16 | | 1 | | 0.00 | | 0 | | 0.00 | | 0 | | 0.00 | | 0 | 0.00 | | 0 | | 1 | | | 0 | 1 |
| O75051 | Plexin-A2 | | | | PLXNA2 | | 0.53 | | 1 | | | 211.0 | | 6.48 | | 0.00 | | 0 | | 0.00 | | 0 | | 0.00 | | 0 | | 0.16 | | 1 | | 0.00 | | 0 | | 0.00 | | 0 | | 0.00 | | 0 | 0.00 | | 0 | | 1 | | | 0 | 1 |
| Q5T653 | 39S ribosomal protein L2, mitochondrial | | | | MRPL2 | | 9.18 | | 1 | | | 33.3 | | 11.30 | | 0.00 | | 0 | | 0.00 | | 0 | | 0.00 | | 0 | | 0.16 | | 1 | | 0.00 | | 0 | | 0.00 | | 0 | | 0.00 | | 0 | 0.00 | | 0 | | 1 | | | 0 | 1 |
| Q9UMN6 | Histone-lysine N-methyltransferase 2B | | | | KMT2B | | 1.25 | | 1 | | | 293.3 | | 8.22 | | 0.00 | | 0 | | 0.00 | | 0 | | 0.00 | | 0 | | 0.16 | | 1 | | 0.00 | | 0 | | 0.00 | | 0 | | 0.00 | | 0 | 0.00 | | 0 | | 1 | | | 0 | 1 |
| Q38SD2 | Leucine-rich repeat serine/threonine-protein kinase 1 | | | | LRRK1 | | 1.04 | | 1 | | | 225.2 | | 6.68 | | 0.00 | | 0 | | 0.00 | | 0 | | 0.00 | | 0 | | 0.16 | | 1 | | 0.00 | | 0 | | 0.00 | | 0 | | 0.00 | | 0 | 0.00 | | 0 | | 1 | | | 0 | 1 |
| Q6AI08 | HEAT repeat-containing protein 6 | | | | HEATR6 | | 0.59 | | 1 | | | 128.7 | | 7.03 | | 0.00 | | 0 | | 0.00 | | 0 | | 0.00 | | 0 | | 0.16 | | 1 | | 0.00 | | 0 | | 0.00 | | 0 | | 0.00 | | 0 | 0.00 | | 0 | | 1 | | | 0 | 1 |
| Q8NFI4 | Putative protein FAM10A5 | | | | ST13P5 | | 14.63 | | 1 | | | 41.4 | | 5.05 | | 0.00 | | 0 | | 0.00 | | 0 | | 0.00 | | 0 | | 0.16 | | 1 | | 0.00 | | 0 | | 0.00 | | 0 | | 0.00 | | 0 | 0.00 | | 0 | | 1 | | | 0 | 1 |
| H0YCG4 | Target of Nesh-SH3 (Fragment) | | | | ABI3BP | | 23.18 | | 1 | | | 16.5 | | 7.25 | | 0.00 | | 0 | | 0.00 | | 0 | | 0.00 | | 0 | | 0.16 | | 1 | | 0.00 | | 0 | | 0.00 | | 0 | | 0.00 | | 0 | 0.00 | | 0 | | 1 | | | 0 | 1 |
| G5E9Z5 | NYD-SP14 protein, isoform CRA_c | | | | TTC29 | | 2.40 | | 1 | | | 57.9 | | 5.55 | | 0.00 | | 0 | | 0.00 | | 0 | | 0.00 | | 0 | | 0.16 | | 1 | | 0.00 | | 0 | | 0.00 | | 0 | | 0.00 | | 0 | 0.00 | | 0 | | 1 | | | 0 | 1 |
| H9KVD2 | Sodium channel protein | | | | SCN5A | | 2.28 | | 1 | | | 226.8 | | 5.53 | | 0.00 | | 0 | | 0.00 | | 0 | | 0.00 | | 0 | | 0.16 | | 1 | | 0.00 | | 0 | | 0.00 | | 0 | | 0.00 | | 0 | 0.00 | | 0 | | 1 | | | 0 | 1 |
| Q8NAP1 | Putative protein CASTOR 3 | | | | CASTOR3 | | 12.88 | | 1 | | | 17.8 | | 8.50 | | 0.00 | | 0 | | 0.00 | | 0 | | 0.00 | | 0 | | 0.16 | | 1 | | 0.00 | | 0 | | 0.00 | | 0 | | 0.00 | | 0 | 0.00 | | 0 | | 1 | | | 0 | 1 |
| Q86UT6 | NLR family member X1 | | | | NLRX1 | | 2.26 | | 1 | | | 107.5 | | 7.37 | | 0.00 | | 0 | | 0.00 | | 0 | | 0.00 | | 0 | | 0.16 | | 1 | | 0.00 | | 0 | | 0.00 | | 0 | | 0.00 | | 0 | 0.00 | | 0 | | 1 | | | 0 | 1 |
| Q9NYU2 | UDP-glucose:glycoprotein glucosyltransferase 1 | | | | UGGT1 | | 2.32 | | 1 | | | 177.1 | | 5.63 | | 0.00 | | 0 | | 0.00 | | 0 | | 0.00 | | 0 | | 0.16 | | 1 | | 0.00 | | 0 | | 0.00 | | 0 | | 0.00 | | 0 | 0.00 | | 0 | | 1 | | | 0 | 1 |
| P02765 | Alpha-2-HS-glycoprotein | | | | AHSG | | 3.54 | | 1 | | | 39.3 | | 5.72 | | 0.00 | | 0 | | 0.00 | | 0 | | 0.00 | | 0 | | 0.16 | | 1 | | 0.00 | | 0 | | 0.00 | | 0 | | 0.00 | | 0 | 0.00 | | 0 | | 1 | | | 0 | 1 |
| O00445 | Synaptotagmin-5 | | | | SYT5 | | 9.84 | | 1 | | | 42.9 | | 9.17 | | 0.00 | | 0 | | 0.00 | | 0 | | 0.00 | | 0 | | 0.16 | | 1 | | 0.00 | | 0 | | 0.00 | | 0 | | 0.00 | | 0 | 0.00 | | 0 | | 1 | | | 0 | 1 |
| P30838 | Aldehyde dehydrogenase, dimeric NADP-preferring | | | | ALDH3A1 | | 5.96 | | 1 | | | 50.4 | | 6.54 | | 0.00 | | 0 | | 0.00 | | 0 | | 0.00 | | 0 | | 0.16 | | 1 | | 0.00 | | 0 | | 0.00 | | 0 | | 0.00 | | 0 | 0.00 | | 0 | | 1 | | | 0 | 1 |
| Q12882 | Dihydropyrimidine dehydrogenase [NADP(+)] | | | | DPYD | | 0.88 | | 1 | | | 111.3 | | 7.05 | | 0.00 | | 0 | | 0.00 | | 0 | | 0.00 | | 0 | | 0.16 | | 1 | | 0.00 | | 0 | | 0.00 | | 0 | | 0.00 | | 0 | 0.00 | | 0 | | 1 | | | 0 | 1 |
| Q6UWN0 | Ly6/PLAUR domain-containing protein 4 | | | | LYPD4 | | 9.35 | | 1 | | | 26.7 | | 8.38 | | 0.00 | | 0 | | 0.00 | | 0 | | 0.00 | | 0 | | 0.16 | | 1 | | 0.00 | | 0 | | 0.00 | | 0 | | 0.00 | | 0 | 0.00 | | 0 | | 1 | | | 0 | 1 |
| B4DJ23 | Myotubularin-related protein 14 | | | | MTMR14 | | 11.70 | | 1 | | | 20.9 | | 8.75 | | 0.00 | | 0 | | 0.00 | | 0 | | 0.00 | | 0 | | 0.16 | | 1 | | 0.00 | | 0 | | 0.00 | | 0 | | 0.00 | | 0 | 0.00 | | 0 | | 1 | | | 0 | 1 |
| A0A2R8YFX0 | Uncharacterized protein | | | |  | | 3.24 | | 1 | | | 38.0 | | 9.76 | | 0.00 | | 0 | | 0.00 | | 0 | | 0.00 | | 0 | | 0.16 | | 1 | | 0.00 | | 0 | | 0.00 | | 0 | | 0.00 | | 0 | 0.00 | | 0 | | 1 | | | 0 | 1 |
| E7EQB3 | tRNA-splicing endonuclease subunit Sen34 | | | | TSEN34 | | 7.30 | | 1 | | | 34.3 | | 8.84 | | 0.00 | | 0 | | 0.00 | | 0 | | 0.00 | | 0 | | 0.16 | | 1 | | 0.00 | | 0 | | 0.00 | | 0 | | 0.00 | | 0 | 0.00 | | 0 | | 1 | | | 0 | 1 |
| H3BP25 | Putative sodium-coupled neutral amino acid transporter 7 | | | | SLC38A7 | | 15.00 | | 1 | | | 27.9 | | 5.01 | | 0.00 | | 0 | | 0.00 | | 0 | | 0.00 | | 0 | | 0.16 | | 1 | | 0.00 | | 0 | | 0.00 | | 0 | | 0.00 | | 0 | 0.00 | | 0 | | 1 | | | 0 | 1 |
| Q99547 | M-phase phosphoprotein 6 | | | | MPHOSPH6 | | 18.75 | | 1 | | | 19.0 | | 5.20 | | 0.00 | | 0 | | 0.00 | | 0 | | 0.00 | | 0 | | 0.16 | | 1 | | 0.00 | | 0 | | 0.00 | | 0 | | 0.00 | | 0 | 0.00 | | 0 | | 1 | | | 0 | 1 |
| Q92824 | Proprotein convertase subtilisin/kexin type 5 | | | | PCSK5 | | 1.88 | | 1 | | | 206.8 | | 6.10 | | 0.00 | | 0 | | 0.00 | | 0 | | 0.00 | | 0 | | 0.16 | | 1 | | 0.00 | | 0 | | 0.00 | | 0 | | 0.00 | | 0 | 0.00 | | 0 | | 1 | | | 0 | 1 |
| Q13332 | Receptor-type tyrosine-protein phosphatase S | | | | PTPRS | | 2.31 | | 1 | | | 216.9 | | 6.46 | | 0.00 | | 0 | | 0.00 | | 0 | | 0.00 | | 0 | | 0.16 | | 1 | | 0.00 | | 0 | | 0.00 | | 0 | | 0.00 | | 0 | 0.00 | | 0 | | 1 | | | 0 | 1 |
| A0A087WX97 | Bcl-2-like protein 13 | | | | BCL2L13 | | 5.11 | | 1 | | | 54.4 | | 4.54 | | 0.00 | | 0 | | 0.00 | | 0 | | 0.00 | | 0 | | 0.16 | | 1 | | 0.00 | | 0 | | 0.00 | | 0 | | 0.00 | | 0 | 0.00 | | 0 | | 1 | | | 0 | 1 |
| Q9Y5H4 | Protocadherin gamma-A1 | | | | PCDHGA1 | | 2.04 | | 1 | | | 101.2 | | 5.03 | | 0.00 | | 0 | | 0.00 | | 0 | | 0.00 | | 0 | | 0.16 | | 1 | | 0.00 | | 0 | | 0.00 | | 0 | | 0.00 | | 0 | 0.00 | | 0 | | 1 | | | 0 | 1 |
| C9JXK9 | Lipoma-preferred partner (Fragment) | | | | LPP | | 6.35 | | 1 | | | 26.7 | | 7.85 | | 0.00 | | 0 | | 0.00 | | 0 | | 0.00 | | 0 | | 0.16 | | 1 | | 0.00 | | 0 | | 0.00 | | 0 | | 0.00 | | 0 | 0.00 | | 0 | | 1 | | | 0 | 1 |
| R4GNG2 | E3 ubiquitin-protein ligase AMFR (Fragment) | | | | AMFR | | 5.69 | | 1 | | | 33.9 | | 5.43 | | 0.00 | | 0 | | 0.00 | | 0 | | 0.00 | | 0 | | 0.16 | | 1 | | 0.00 | | 0 | | 0.00 | | 0 | | 0.00 | | 0 | 0.00 | | 0 | | 1 | | | 0 | 1 |
| Q6DT37 | Serine/threonine-protein kinase MRCK gamma | | | | CDC42BPG | | 2.39 | | 1 | | | 172.4 | | 6.28 | | 0.00 | | 0 | | 0.00 | | 0 | | 0.00 | | 0 | | 0.16 | | 1 | | 0.00 | | 0 | | 0.00 | | 0 | | 0.00 | | 0 | 0.00 | | 0 | | 1 | | | 0 | 1 |
| O43156 | TELO2-interacting protein 1 homolog | | | | TTI1 | | 3.31 | | 1 | | | 122.0 | | 5.97 | | 0.00 | | 0 | | 0.00 | | 0 | | 0.00 | | 0 | | 0.16 | | 1 | | 0.00 | | 0 | | 0.00 | | 0 | | 0.00 | | 0 | 0.00 | | 0 | | 1 | | | 0 | 1 |
| Q14164 | Inhibitor of nuclear factor kappa-B kinase subunit epsilon | | | | IKBKE | | 6.01 | | 1 | | | 80.4 | | 7.84 | | 0.00 | | 0 | | 0.00 | | 0 | | 0.00 | | 0 | | 0.16 | | 1 | | 0.00 | | 0 | | 0.00 | | 0 | | 0.00 | | 0 | 0.00 | | 0 | | 1 | | | 0 | 1 |
| Q8N0V3 | Putative ribosome-binding factor A, mitochondrial | | | | RBFA | | 12.83 | | 1 | | | 38.3 | | 7.85 | | 0.00 | | 0 | | 0.00 | | 0 | | 0.00 | | 0 | | 0.16 | | 1 | | 0.00 | | 0 | | 0.00 | | 0 | | 0.00 | | 0 | 0.00 | | 0 | | 1 | | | 0 | 1 |
| F8VU56 | Receptor-type tyrosine-protein phosphatase beta | | | | PTPRB | | 1.08 | | 1 | | | 239.4 | | 7.74 | | 0.00 | | 0 | | 0.00 | | 0 | | 0.00 | | 0 | | 0.16 | | 1 | | 0.00 | | 0 | | 0.00 | | 0 | | 0.00 | | 0 | 0.00 | | 0 | | 1 | | | 0 | 1 |
| Q14416 | Metabotropic glutamate receptor 2 | | | | GRM2 | | 3.33 | | 1 | | | 95.5 | | 8.12 | | 0.00 | | 0 | | 0.00 | | 0 | | 0.00 | | 0 | | 0.16 | | 1 | | 0.00 | | 0 | | 0.00 | | 0 | | 0.00 | | 0 | 0.00 | | 0 | | 1 | | | 0 | 1 |
| P16383 | GC-rich sequence DNA-binding factor 2 | | | | GCFC2 | | 6.15 | | 1 | | | 89.3 | | 5.99 | | 0.00 | | 0 | | 0.00 | | 0 | | 0.00 | | 0 | | 0.16 | | 1 | | 0.00 | | 0 | | 0.00 | | 0 | | 0.00 | | 0 | 0.00 | | 0 | | 1 | | | 0 | 1 |
| O60784 | Target of Myb protein 1 | | | | TOM1 | | 6.71 | | 1 | | | 53.8 | | 4.70 | | 0.00 | | 0 | | 0.00 | | 0 | | 0.00 | | 0 | | 0.16 | | 1 | | 0.00 | | 0 | | 0.00 | | 0 | | 0.00 | | 0 | 0.00 | | 0 | | 1 | | | 0 | 1 |
| K7EIK4 | Thimet oligopeptidase (Fragment) | | | | THOP1 | | 21.89 | | 1 | | | 19.1 | | 5.34 | | 0.00 | | 0 | | 0.00 | | 0 | | 0.00 | | 0 | | 0.16 | | 1 | | 0.00 | | 0 | | 0.00 | | 0 | | 0.00 | | 0 | 0.00 | | 0 | | 1 | | | 0 | 1 |
| Q6ZMP0 | Thrombospondin type-1 domain-containing protein 4 | | | | THSD4 | | 3.73 | | 1 | | | 112.4 | | 7.65 | | 0.00 | | 0 | | 0.00 | | 0 | | 0.00 | | 0 | | 0.16 | | 1 | | 0.00 | | 0 | | 0.00 | | 0 | | 0.00 | | 0 | 0.00 | | 0 | | 1 | | | 0 | 1 |
| P11511 | Aromatase | | | | CYP19A1 | | 4.37 | | 1 | | | 57.8 | | 7.50 | | 0.00 | | 0 | | 0.00 | | 0 | | 0.00 | | 0 | | 0.16 | | 1 | | 0.00 | | 0 | | 0.00 | | 0 | | 0.00 | | 0 | 0.00 | | 0 | | 1 | | | 0 | 1 |
| Q8NGV7 | Olfactory receptor 5H2 | | | | OR5H2 | | 9.24 | | 1 | | | 36.0 | | 7.65 | | 0.00 | | 0 | | 0.00 | | 0 | | 0.00 | | 0 | | 0.16 | | 1 | | 0.00 | | 0 | | 0.00 | | 0 | | 0.00 | | 0 | 0.00 | | 0 | | 1 | | | 0 | 1 |
| O14559 | Rho GTPase-activating protein 33 | | | | ARHGAP33 | | 1.48 | | 1 | | | 137.1 | | 8.75 | | 0.00 | | 0 | | 0.00 | | 0 | | 0.00 | | 0 | | 0.16 | | 1 | | 0.00 | | 0 | | 0.00 | | 0 | | 0.00 | | 0 | 0.00 | | 0 | | 1 | | | 0 | 1 |
| Q6VAB6 | Kinase suppressor of Ras 2 | | | | KSR2 | | 1.79 | | 1 | | | 107.6 | | 8.69 | | 0.00 | | 0 | | 0.00 | | 0 | | 0.00 | | 0 | | 0.16 | | 1 | | 0.00 | | 0 | | 0.00 | | 0 | | 0.00 | | 0 | 0.00 | | 0 | | 1 | | | 0 | 1 |
| A0A494C1V2 | Zinc finger protein 891 | | | | ZNF891 | | 8.39 | | 1 | | | 64.0 | | 8.73 | | 0.00 | | 0 | | 0.00 | | 0 | | 0.00 | | 0 | | 0.16 | | 1 | | 0.00 | | 0 | | 0.00 | | 0 | | 0.00 | | 0 | 0.00 | | 0 | | 1 | | | 0 | 1 |
| B5MEB3 | Calcineurin binding protein 1, isoform CRA_c | | | | CABIN1 | | 4.07 | | 1 | | | 63.3 | | 6.77 | | 0.00 | | 0 | | 0.00 | | 0 | | 0.00 | | 0 | | 0.16 | | 1 | | 0.00 | | 0 | | 0.00 | | 0 | | 0.00 | | 0 | 0.00 | | 0 | | 1 | | | 0 | 1 |
| Q8IZ16 | Uncharacterized protein C7orf61 | | | | C7orf61 | | 16.02 | | 1 | | | 23.8 | | 10.40 | | 0.00 | | 0 | | 0.00 | | 0 | | 0.00 | | 0 | | 0.16 | | 1 | | 0.00 | | 0 | | 0.00 | | 0 | | 0.00 | | 0 | 0.00 | | 0 | | 1 | | | 0 | 1 |
| A0A0U1RQC5 | Neurexin-3-beta | | | | NRXN3 | | 1.68 | | 1 | | | 183.2 | | 5.74 | | 0.00 | | 0 | | 0.00 | | 0 | | 0.00 | | 0 | | 0.16 | | 1 | | 0.00 | | 0 | | 0.00 | | 0 | | 0.00 | | 0 | 0.00 | | 0 | | 1 | | | 0 | 1 |
| Q6ZRQ5 | Protein MMS22-like | | | | MMS22L | | 1.45 | | 1 | | | 142.2 | | 7.12 | | 0.00 | | 0 | | 0.00 | | 0 | | 0.00 | | 0 | | 0.16 | | 1 | | 0.00 | | 0 | | 0.00 | | 0 | | 0.00 | | 0 | 0.00 | | 0 | | 1 | | | 0 | 1 |
| H7C4F4 | Major facilitator superfamily domain-containing protein 1 (Fragment) | | | | MFSD1 | | 30.05 | | 1 | | | 21.0 | | 7.94 | | 0.00 | | 0 | | 0.00 | | 0 | | 0.00 | | 0 | | 0.16 | | 1 | | 0.00 | | 0 | | 0.00 | | 0 | | 0.00 | | 0 | 0.00 | | 0 | | 1 | | | 0 | 1 |
| Q7L1I2 | Synaptic vesicle glycoprotein 2B | | | | SV2B | | 7.17 | | 1 | | | 77.4 | | 5.44 | | 0.00 | | 0 | | 0.00 | | 0 | | 0.00 | | 0 | | 0.16 | | 1 | | 0.00 | | 0 | | 0.00 | | 0 | | 0.00 | | 0 | 0.00 | | 0 | | 1 | | | 0 | 1 |
| E5RHC4 | Arylsulfatase B (Fragment) | | | | ARSB | | 62.22 | | 1 | | | 4.5 | | 12.60 | | 0.00 | | 0 | | 0.00 | | 0 | | 0.00 | | 0 | | 0.16 | | 1 | | 0.00 | | 0 | | 0.00 | | 0 | | 0.00 | | 0 | 0.00 | | 0 | | 1 | | | 0 | 1 |
| P28062 | Proteasome subunit beta type-8 | | | | PSMB8 | | 8.70 | | 1 | | | 30.3 | | 7.43 | | 0.00 | | 0 | | 0.00 | | 0 | | 0.00 | | 0 | | 0.16 | | 1 | | 0.00 | | 0 | | 0.00 | | 0 | | 0.00 | | 0 | 0.00 | | 0 | | 1 | | | 0 | 1 |
| A0A0A0MTI5 | Acyl-CoA-binding protein | | | | DBI | | 19.58 | | 2 | | | 15.9 | | 5.05 | | 0.00 | | 0 | | 0.95 | | 4 | | 0.00 | | 0 | | 0.00 | | 0 | | 0.00 | | 0 | | 0.00 | | 0 | | 0.00 | | 0 | 0.00 | | 0 | | 1 | | | 0 | 1 |
| B2RTY4 | Unconventional myosin-IXa | | | | MYO9A | | 0.31 | | 1 | | | 292.5 | | 8.88 | | 0.00 | | 0 | | 0.41 | | 3 | | 0.00 | | 0 | | 0.00 | | 0 | | 0.00 | | 0 | | 0.00 | | 0 | | 0.00 | | 0 | 0.00 | | 0 | | 1 | | | 0 | 1 |
| Q9UPA5 | Protein bassoon | | | | BSN | | 0.82 | | 2 | | | 416.2 | | 7.55 | | 0.00 | | 0 | | 0.43 | | 3 | | 0.00 | | 0 | | 0.00 | | 0 | | 0.00 | | 0 | | 0.00 | | 0 | | 0.00 | | 0 | 0.00 | | 0 | | 1 | | | 0 | 1 |
| Q9UBG0 | C-type mannose receptor 2 | | | | MRC2 | | 1.83 | | 2 | | | 166.6 | | 5.83 | | 0.00 | | 0 | | 0.64 | | 2 | | 0.00 | | 0 | | 0.00 | | 0 | | 0.00 | | 0 | | 0.00 | | 0 | | 0.00 | | 0 | 0.00 | | 0 | | 1 | | | 0 | 1 |
| P55263 | Adenosine kinase | | | | ADK | | 4.42 | | 1 | | | 40.5 | | 6.70 | | 0.00 | | 0 | | 0.41 | | 2 | | 0.00 | | 0 | | 0.00 | | 0 | | 0.00 | | 0 | | 0.00 | | 0 | | 0.00 | | 0 | 0.00 | | 0 | | 1 | | | 0 | 1 |
| E5RJR5 | S-phase kinase-associated protein 1 | | | | SKP1 | | 7.36 | | 1 | | | 18.7 | | 4.70 | | 0.00 | | 0 | | 0.41 | | 2 | | 0.00 | | 0 | | 0.00 | | 0 | | 0.00 | | 0 | | 0.00 | | 0 | | 0.00 | | 0 | 0.00 | | 0 | | 1 | | | 0 | 1 |
| Q92820 | Gamma-glutamyl hydrolase | | | | GGH | | 6.92 | | 2 | | | 35.9 | | 7.11 | | 0.00 | | 0 | | 0.48 | | 2 | | 0.00 | | 0 | | 0.00 | | 0 | | 0.00 | | 0 | | 0.00 | | 0 | | 0.00 | | 0 | 0.00 | | 0 | | 1 | | | 0 | 1 |
| P49721 | Proteasome subunit beta type-2 | | | | PSMB2 | | 8.96 | | 2 | | | 22.8 | | 7.02 | | 0.00 | | 0 | | 0.40 | | 2 | | 0.00 | | 0 | | 0.00 | | 0 | | 0.00 | | 0 | | 0.00 | | 0 | | 0.00 | | 0 | 0.00 | | 0 | | 1 | | | 0 | 1 |
| Q00688 | Peptidyl-prolyl cis-trans isomerase FKBP3 | | | | FKBP3 | | 6.70 | | 1 | | | 25.2 | | 9.28 | | 0.00 | | 0 | | 0.27 | | 2 | | 0.00 | | 0 | | 0.00 | | 0 | | 0.00 | | 0 | | 0.00 | | 0 | | 0.00 | | 0 | 0.00 | | 0 | | 1 | | | 0 | 1 |
| P27695 | DNA-(apurinic or apyrimidinic site) lyase | | | | APEX1 | | 11.64 | | 2 | | | 35.5 | | 8.12 | | 0.00 | | 0 | | 0.27 | | 2 | | 0.00 | | 0 | | 0.00 | | 0 | | 0.00 | | 0 | | 0.00 | | 0 | | 0.00 | | 0 | 0.00 | | 0 | | 1 | | | 0 | 1 |
| Q9NQZ7 | Ectonucleoside triphosphate diphosphohydrolase 7 | | | | ENTPD7 | | 4.64 | | 1 | | | 68.9 | | 7.56 | | 0.00 | | 0 | | 0.27 | | 2 | | 0.00 | | 0 | | 0.00 | | 0 | | 0.00 | | 0 | | 0.00 | | 0 | | 0.00 | | 0 | 0.00 | | 0 | | 1 | | | 0 | 1 |
| D6RAR4 | Hepatocyte growth factor activator | | | | HGFAC | | 5.29 | | 2 | | | 71.4 | | 7.05 | | 0.00 | | 0 | | 0.27 | | 2 | | 0.00 | | 0 | | 0.00 | | 0 | | 0.00 | | 0 | | 0.00 | | 0 | | 0.00 | | 0 | 0.00 | | 0 | | 1 | | | 0 | 1 |
| Q99733 | Nucleosome assembly protein 1-like 4 | | | | NAP1L4 | | 2.93 | | 1 | | | 42.8 | | 4.69 | | 0.00 | | 0 | | 0.27 | | 2 | | 0.00 | | 0 | | 0.00 | | 0 | | 0.00 | | 0 | | 0.00 | | 0 | | 0.00 | | 0 | 0.00 | | 0 | | 1 | | | 0 | 1 |
| Q9H7P6 | Multivesicular body subunit 12B | | | | MVB12B | | 10.66 | | 2 | | | 35.6 | | 8.15 | | 0.00 | | 0 | | 0.27 | | 2 | | 0.00 | | 0 | | 0.00 | | 0 | | 0.00 | | 0 | | 0.00 | | 0 | | 0.00 | | 0 | 0.00 | | 0 | | 1 | | | 0 | 1 |
| Q96BJ8 | Engulfment and cell motility protein 3 | | | | ELMO3 | | 7.92 | | 2 | | | 81.4 | | 6.30 | | 0.00 | | 0 | | 0.27 | | 2 | | 0.00 | | 0 | | 0.00 | | 0 | | 0.00 | | 0 | | 0.00 | | 0 | | 0.00 | | 0 | 0.00 | | 0 | | 1 | | | 0 | 1 |
| Q9UBT6 | DNA polymerase kappa | | | | POLK | | 4.37 | | 2 | | | 98.7 | | 8.13 | | 0.00 | | 0 | | 0.27 | | 2 | | 0.00 | | 0 | | 0.00 | | 0 | | 0.00 | | 0 | | 0.00 | | 0 | | 0.00 | | 0 | 0.00 | | 0 | | 1 | | | 0 | 1 |
| Q96GR2 | Long-chain-fatty-acid--CoA ligase ACSBG1 | | | | ACSBG1 | | 8.15 | | 2 | | | 81.2 | | 6.02 | | 0.00 | | 0 | | 0.30 | | 2 | | 0.00 | | 0 | | 0.00 | | 0 | | 0.00 | | 0 | | 0.00 | | 0 | | 0.00 | | 0 | 0.00 | | 0 | | 1 | | | 0 | 1 |
| P46663 | B1 bradykinin receptor | | | | BDKRB1 | | 3.12 | | 1 | | | 40.5 | | 9.31 | | 0.00 | | 0 | | 0.30 | | 2 | | 0.00 | | 0 | | 0.00 | | 0 | | 0.00 | | 0 | | 0.00 | | 0 | | 0.00 | | 0 | 0.00 | | 0 | | 1 | | | 0 | 1 |
| Q13630 | GDP-L-fucose synthase | | | | TSTA3 | | 3.74 | | 1 | | | 35.9 | | 6.60 | | 0.00 | | 0 | | 0.29 | | 2 | | 0.00 | | 0 | | 0.00 | | 0 | | 0.00 | | 0 | | 0.00 | | 0 | | 0.00 | | 0 | 0.00 | | 0 | | 1 | | | 0 | 1 |
| Q8WW22 | DnaJ homolog subfamily A member 4 | | | | DNAJA4 | | 2.02 | | 1 | | | 44.8 | | 7.59 | | 0.00 | | 0 | | 0.30 | | 2 | | 0.00 | | 0 | | 0.00 | | 0 | | 0.00 | | 0 | | 0.00 | | 0 | | 0.00 | | 0 | 0.00 | | 0 | | 1 | | | 0 | 1 |
| Q14376 | UDP-glucose 4-epimerase | | | | GALE | | 6.03 | | 1 | | | 38.3 | | 6.73 | | 0.00 | | 0 | | 0.27 | | 2 | | 0.00 | | 0 | | 0.00 | | 0 | | 0.00 | | 0 | | 0.00 | | 0 | | 0.00 | | 0 | 0.00 | | 0 | | 1 | | | 0 | 1 |
| Q8TE56 | A disintegrin and metalloproteinase with thrombospondin motifs 17 | | | | ADAMTS17 | | 1.00 | | 1 | | | 121.0 | | 8.06 | | 0.00 | | 0 | | 0.27 | | 2 | | 0.00 | | 0 | | 0.00 | | 0 | | 0.00 | | 0 | | 0.00 | | 0 | | 0.00 | | 0 | 0.00 | | 0 | | 1 | | | 0 | 1 |
| A0A2R8YFN3 | ADP-ribose glycohydrolase MACROD2 | | | | MACROD2 | | 11.92 | | 2 | | | 50.4 | | 4.74 | | 0.00 | | 0 | | 0.27 | | 2 | | 0.00 | | 0 | | 0.00 | | 0 | | 0.00 | | 0 | | 0.00 | | 0 | | 0.00 | | 0 | 0.00 | | 0 | | 1 | | | 0 | 1 |
| O95372 | Acyl-protein thioesterase 2 | | | | LYPLA2 | | 11.26 | | 1 | | | 24.7 | | 7.23 | | 0.00 | | 0 | | 0.28 | | 1 | | 0.00 | | 0 | | 0.00 | | 0 | | 0.00 | | 0 | | 0.00 | | 0 | | 0.00 | | 0 | 0.00 | | 0 | | 1 | | | 0 | 1 |
| O60664 | Perilipin-3 | | | | PLIN3 | | 9.45 | | 2 | | | 47.0 | | 5.44 | | 0.00 | | 0 | | 0.27 | | 1 | | 0.00 | | 0 | | 0.00 | | 0 | | 0.00 | | 0 | | 0.00 | | 0 | | 0.00 | | 0 | 0.00 | | 0 | | 1 | | | 0 | 1 |
| Q99683 | Mitogen-activated protein kinase kinase kinase 5 | | | | MAP3K5 | | 0.87 | | 1 | | | 154.4 | | 5.78 | | 0.00 | | 0 | | 0.26 | | 1 | | 0.00 | | 0 | | 0.00 | | 0 | | 0.00 | | 0 | | 0.00 | | 0 | | 0.00 | | 0 | 0.00 | | 0 | | 1 | | | 0 | 1 |
| P46108 | Adapter molecule crk | | | | CRK | | 5.59 | | 1 | | | 33.8 | | 5.55 | | 0.00 | | 0 | | 0.27 | | 1 | | 0.00 | | 0 | | 0.00 | | 0 | | 0.00 | | 0 | | 0.00 | | 0 | | 0.00 | | 0 | 0.00 | | 0 | | 1 | | | 0 | 1 |
| Q96PZ0 | Pseudouridylate synthase 7 homolog | | | | PUS7 | | 3.03 | | 1 | | | 75.0 | | 6.37 | | 0.00 | | 0 | | 0.27 | | 1 | | 0.00 | | 0 | | 0.00 | | 0 | | 0.00 | | 0 | | 0.00 | | 0 | | 0.00 | | 0 | 0.00 | | 0 | | 1 | | | 0 | 1 |
| Q92805 | Golgin subfamily A member 1 | | | | GOLGA1 | | 2.74 | | 1 | | | 88.1 | | 5.27 | | 0.00 | | 0 | | 0.14 | | 1 | | 0.00 | | 0 | | 0.00 | | 0 | | 0.00 | | 0 | | 0.00 | | 0 | | 0.00 | | 0 | 0.00 | | 0 | | 1 | | | 0 | 1 |
| P04843 | Dolichyl-diphosphooligosaccharide--protein glycosyltransferase subunit 1 | | | | RPN1 | | 1.98 | | 1 | | | 68.5 | | 6.38 | | 0.00 | | 0 | | 0.14 | | 1 | | 0.00 | | 0 | | 0.00 | | 0 | | 0.00 | | 0 | | 0.00 | | 0 | | 0.00 | | 0 | 0.00 | | 0 | | 1 | | | 0 | 1 |
| A5PLL3 | Histone acetyltransferase | | | | KAT6A | | 0.98 | | 1 | | | 93.2 | | 8.94 | | 0.00 | | 0 | | 0.14 | | 1 | | 0.00 | | 0 | | 0.00 | | 0 | | 0.00 | | 0 | | 0.00 | | 0 | | 0.00 | | 0 | 0.00 | | 0 | | 1 | | | 0 | 1 |
[truncated: 562,698 more chars]
