# Supplementary material for: Comprehensive Profiling of Secretome Formulations from Fetal- and Perinatal Human Amniotic Fluid Stem Cells
Source: Int J Mol Sci. 2021 Apr 2;22(7):3713. doi: 10.3390/ijms22073713 (PMC8038201; doi:10.3390/ijms22073713)
Supplement: Supplementary file 1 [file ijms-22-03713-s001.zip › Costa A et al_Supplementary Files/Costa A et al_Table S2.docx]

**Table S2. Complete list of differentially expressed proteins extracted from MAProMa comparison of fetal hAFS-CM versus perinatal hAFS-CM formulations.** For each protein Uniprot Accession, Gene name, Reference, Frequency in hAFS-CM, aPSM are reported in fetal hAFS-CM (f-hAFS-CM) and perinatal hAFS-CM (p-hAFS-CM) according to in vitro cell hypoxic preconditioning (f-hAFS-CM_normo_; f-hAFS-CM_hypo;_ p-hAFS-CM_normo_ and p-hAFS-CM_hypo,_ respectively). Table shows DAve and DCI value calculated by MAProMa software for: comparison of f-hAFS-CM versus p-hAFS-CM; for f-hAFS-CM_normo_ vs f-hAFS-CM_hypo_ within up-regulated f-hAFS-CM proteins resulting from the comparison with p-hAFS-CM, and for p-hAFS-CM_normo_ vs p-hAFS-CM_hypo_ of up-regulated p-hAFS-CM proteins resulting from the comparison with f-hAFS-CM. Frequency indicates how many times a given protein has been identified under the examined conditions (total n=24 runs for hAFS-CM). The aPSM indicates that average values are given for each protein of the same condition. Positive values for DAve and DCI indicate that the corresponding protein is more abundant in the first compared condition, while negative values in the second ones. DAve and DCI values are reported in bold if they exceed the imposed thresholds. For further details regarding the meaning and the confidence range applied to DAve and DCI see Materials & Methods section.

| **Uniprot Accession** | **Reference** | **Gene**  **Name** | **Frequency hAFS-CM** | **aPSM hAFS-CM** | | | | | | **MAProMa** | | | | | | | |
| --- | --- | --- | --- | --- | --- | --- | --- | --- | --- | --- | --- | --- | --- | --- | --- | --- | --- |
|  |  |  |  |  |  |  |  |  |  | **f-hAFS-CM vs p-hAFS-CM** | | | **f-hAFS-CM_normo_ vs f-hAFS-CM_hypo_** | | | **p-hAFS-CM_normo_ vs p-hAFS-CM_hypo_** | |
|  |  |  |  | **f-hAFS-**  **CM_normo_** | **f-hAFS-CM_hypo_** | **f-hAFS-CM** | **p-hAFS-**  **CM_normo_** | **p-hAFS-CM_hypo_** | **p-hAFS-CM** | **DAve** | **DCI** | **DAve** | | **DCI** | **DAve** | | **DCI** |
| A0A494C0G5 | Agrin | AGRN | 9 | 4.78 | 5.55 | 5.17 | 0.00 | 0.00 | 0.00 | **2.00** | **13** | -0.15 | | -4 |  | |  |
| O00468 | Agrin | AGRN | 9 | 4.64 | 5.65 | 5.14 | 0.00 | 0.00 | 0.00 | **2.00** | **13** | -0.20 | | -5 |  | |  |
| P98160 | Basement membrane-specific heparan sulfate proteoglycan core protein | HSPG2 | 10 | 4.22 | 4.53 | 4.38 | 0.16 | 0.00 | 0.08 | **1.93** | **10** | -0.07 | | -1 |  | |  |
| P55285 | Cadherin-6 | CDH6 | 14 | 11.24 | 8.47 | 9.85 | 0.22 | 0.16 | 0.19 | **1.92** | **49** | 0.28 | | 27 |  | |  |
| P07996 | Thrombospondin-1 | THBS1 | 20 | 32.69 | 25.55 | 29.12 | 2.48 | 3.07 | 2.78 | **1.65** | **420** | 0.25 | | 208 |  | |  |
| A0A0C4DGW9 | Serpin I2 | SERPINI2 | 14 | 4.21 | 2.18 | 3.20 | 0.60 | 0.33 | 0.46 | **1.49** | **5** | **0.63** | | **6** |  | |  |
| P03956 | Interstitial collagenase | MMP1 | 19 | 37.98 | 30.22 | 34.10 | 2.48 | 7.71 | 5.10 | **1.48** | **568** | 0.23 | | 265 |  | |  |
| P60842 | Eukaryotic initiation factor 4A-I | EIF4A1 | 10 | 3.38 | 2.99 | 3.19 | 1.11 | 0.00 | 0.56 | **1.41** | **5** | 0.12 | | 1 |  | |  |
| P10451 | Osteopontin | SPP1 | 14 | 5.55 | 5.14 | 5.34 | 0.56 | 1.32 | 0.94 | **1.40** | **14** | 0.08 | | 2 |  | |  |
| P13611 | Versican core protein | VCAN | 19 | 10.21 | 10.21 | 10.21 | 2.05 | 1.75 | 1.90 | **1.37** | **50** | 0.00 | | 0 |  | |  |
| O15230 | Laminin subunit alpha-5 | LAMA5 | 16 | 5.96 | 3.51 | 4.74 | 1.45 | 0.84 | 1.15 | **1.22** | **11** | **0.52** | | **12** |  | |  |
| P10809 | 60 kDa heat shock protein, mitochondrial | HSPD1 | 15 | 1.01 | 8.45 | 4.73 | 0.56 | 2.15 | 1.36 | **1.11** | **10** | **-1.57** | | **-35** |  | |  |
| P37837 | Transaldolase | TALDO1 | 13 | 3.12 | 3.40 | 3.26 | 1.64 | 0.31 | 0.98 | **1.08** | **5** | -0.09 | | -1 |  | |  |
| P19022 | Cadherin-2 | CDH2 | 20 | 5.19 | 5.32 | 5.26 | 1.39 | 2.05 | 1.72 | **1.01** | **12** | -0.03 | | -1 |  | |  |
| Q16881 | Thioredoxin reductase 1, cytoplasmic | TXNRD1 | 20 | 6.26 | 8.87 | 7.56 | 2.29 | 2.82 | 2.55 | **0.99** | **25** | -0.34 | | -20 |  | |  |
| O94985 | Calsyntenin-1 | CLSTN1 | 24 | 9.66 | 7.43 | 8.54 | 4.57 | 3.08 | 3.83 | **0.76** | **29** | 0.26 | | 19 |  | |  |
| P05787 | Keratin, type II cytoskeletal 8 | KRT8 | 24 | 7.90 | 7.85 | 7.88 | 4.19 | 3.59 | 3.89 | **0.68** | **23** | 0.01 | | 0 |  | |  |
| P08727 | Keratin, type I cytoskeletal 19 | KRT19 | 22 | 6.66 | 4.58 | 5.62 | 4.00 | 1.58 | 2.79 | **0.67** | **12** | 0.37 | | 12 |  | |  |
| P07355 | Annexin A2 | ANXA2 | 17 | 7.79 | 10.77 | 9.28 | 2.47 | 7.07 | 4.77 | **0.64** | **32** | -0.32 | | -28 |  | |  |
| P23284 | Peptidyl-prolyl cis-trans isomerase B | PPIB | 23 | 8.43 | 9.47 | 8.95 | 3.20 | 6.12 | 4.66 | **0.63** | **29** | -0.12 | | -9 |  | |  |
| P00749 | Urokinase-type plasminogen activator | PLAU | 17 | 5.79 | 5.31 | 5.55 | 4.25 | 1.68 | 2.97 | **0.61** | **11** | 0.09 | | 3 |  | |  |
| P40926 | Malate dehydrogenase, mitochondrial | MDH2 | 23 | 9.32 | 8.81 | 9.07 | 4.09 | 5.72 | 4.90 | **0.60** | **29** | 0.06 | | 5 |  | |  |
| J3KQE5 | GTP-binding nuclear protein Ran (Fragment) | RAN | 20 | 3.50 | 3.95 | 3.72 | 1.70 | 2.38 | 2.04 | **0.58** | **5** | -0.12 | | -2 |  | |  |
| Q08380 | Galectin-3-binding protein | LGALS3BP | 24 | 17.41 | 12.41 | 14.91 | 10.19 | 6.61 | 8.40 | **0.56** | **76** | 0.34 | | 75 |  | |  |
| E7EMB3 | Calmodulin-2 | CALM2 | 24 | 7.36 | 10.62 | 8.99 | 3.42 | 7.31 | 5.37 | **0.50** | **26** | -0.36 | | -29 |  | |  |
| Q9Y4K0 | Lysyl oxidase homolog 2 | LOXL2 | 23 | 9.68 | 7.22 | 8.45 | 7.07 | 3.23 | 5.15 | **0.48** | **22** | 0.29 | | 21 |  | |  |
| P20618 | Proteasome subunit beta type-1 | PSMB1 | 18 | 3.49 | 5.53 | 4.51 | 2.88 | 2.65 | 2.77 | **0.48** | **6** | **-0.45** | | **-9** |  | |  |
| Q92626 | Peroxidasin homolog | PXDN | 22 | 6.28 | 7.68 | 6.98 | 4.32 | 4.80 | 4.56 | **0.42** | **14** | -0.20 | | -10 |  | |  |
| P08253 | 72 kDa type IV collagenase | MMP2 | 20 | 5.67 | 3.54 | 4.60 | 3.33 | 2.79 | 3.06 | **0.40** | **6** | **0.46** | | **10** |  | |  |
| P12109 | Collagen alpha-1(VI) chain | COL6A1 | 21 | 9.10 | 4.16 | 6.63 | 4.86 | 3.99 | 4.43 | **0.40** | **12** | **0.75** | | **33** |  | |  |
| P02545 | Prelamin-A/C | LMNA | 24 | 18.58 | 22.83 | 20.70 | 30.21 | 31.77 | 30.99 | **-0.40** | **-266** |  | |  | -0.05 | | -48 |
| P35908 | Keratin, type II cytoskeletal 2 epidermal | KRT2 | 16 | 4.30 | 1.28 | 2.79 | 5.98 | 2.43 | 4.20 | **-0.40** | **-5** |  | |  | **0.84** | | **15** |
| P04264 | Keratin, type II cytoskeletal 1 | KRT1 | 20 | 14.39 | 7.28 | 10.83 | 20.76 | 12.03 | 16.39 | **-0.41** | **-76** |  | |  | **0.53** | | **143** |
| P0C0S5 | Histone H2A.Z | H2AZ1 | 23 | 3.22 | 2.70 | 2.96 | 5.25 | 3.75 | 4.50 | **-0.41** | **-6** |  | |  | 0.33 | | 7 |
| P37802 | Transgelin-2 | TAGLN2 | 23 | 12.74 | 14.22 | 13.48 | 20.30 | 20.72 | 20.51 | **-0.41** | **-119** |  | |  | -0.02 | | -9 |
| P04259 | Keratin, type II cytoskeletal 6B | KRT6B | 18 | 4.84 | 2.27 | 3.55 | 7.67 | 3.41 | 5.54 | **-0.44** | **-9** |  | |  | **0.77** | | **24** |
| Q9Y490 | Talin-1 | TLN1 | 20 | 4.45 | 4.66 | 4.55 | 10.53 | 3.84 | 7.19 | **-0.45** | **-15** |  | |  | **0.93** | | **48** |
| Q01995 | Transgelin | TAGLN | 24 | 18.28 | 19.69 | 18.99 | 30.66 | 29.30 | 29.98 | **-0.45** | **-269** |  | |  | 0.05 | | 41 |
| P02538 | Keratin, type II cytoskeletal 6A | KRT6A | 17 | 3.54 | 1.63 | 2.58 | 6.10 | 2.12 | 4.11 | **-0.46** | **-5** |  | |  | **0.97** | | **16** |
| P08779 | Keratin, type I cytoskeletal 16 | KRT16 | 20 | 3.45 | 2.42 | 2.93 | 6.65 | 2.71 | 4.68 | **-0.46** | **-7** |  | |  | **0.84** | | **18** |
| P21333 | Filamin-A | FLNA | 24 | 33.78 | 41.57 | 37.68 | 60.48 | 60.00 | 60.24 | **-0.46** | **-1105** |  | |  | 0.01 | | 29 |
| P22626 | Heterogeneous nuclear ribonucleoproteins A2/B1 | HNRNPA2B1 | 20 | 4.27 | 4.35 | 4.31 | 6.48 | 7.58 | 7.03 | **-0.48** | **-15** |  | |  | -0.16 | | -8 |
| P19823 | Inter-alpha-trypsin inhibitor heavy chain H2 | ITIH2 | 24 | 5.77 | 2.65 | 4.21 | 6.62 | 7.61 | 7.12 | **-0.51** | **-16** |  | |  | -0.14 | | -7 |
| P02533 | Keratin, type I cytoskeletal 14 | KRT14 | 20 | 3.45 | 2.42 | 2.93 | 7.21 | 2.71 | 4.96 | **-0.51** | **-8** |  | |  | **0.91** | | **22** |
| P62857 | 40S ribosomal protein S28 | RPS28 | 22 | 1.30 | 3.03 | 2.17 | 3.65 | 3.83 | 3.74 | **-0.53** | **-5** |  | |  | -0.05 | | -1 |
| P09382 | Galectin-1 | LGALS1 | 24 | 6.00 | 7.40 | 6.70 | 11.36 | 11.86 | 11.61 | **-0.54** | **-45** |  | |  | -0.04 | | -6 |
| P13645 | Keratin, type I cytoskeletal 10 | KRT10 | 17 | 5.93 | 2.25 | 4.09 | 12.06 | 2.42 | 7.24 | **-0.56** | **-18** |  | |  | **1.33** | | **70** |
| J3KMX3 | Alpha-fetoprotein | AFP | 22 | 2.97 | 2.04 | 2.51 | 4.63 | 4.58 | 4.61 | **-0.59** | **-7** |  | |  | 0.01 | | 0 |
| J3QRS3 | Myosin regulatory light chain 12A | MYL12A | 22 | 1.77 | 7.36 | 4.56 | 6.92 | 10.87 | 8.90 | **-0.64** | **-29** |  | |  | **-0.44** | | **-35** |
| P69905 | Hemoglobin subunit alpha | HBA1 | 24 | 5.30 | 4.20 | 4.75 | 9.61 | 8.99 | 9.30 | **-0.65** | **-32** |  | |  | 0.07 | | 6 |
| P35749 | Myosin-11 | MYH11 | 18 | 2.31 | 0.95 | 1.63 | 3.76 | 3.28 | 3.52 | **-0.73** | **-5** |  | |  | 0.14 | | 2 |
| P35579 | Myosin-9 | MYH9 | 22 | 12.94 | 12.89 | 12.92 | 26.74 | 29.15 | 27.95 | **-0.74** | **-307** |  | |  | -0.09 | | -68 |
| P24844 | Myosin regulatory light polypeptide 9 | MYL9 | 20 | 0.81 | 4.81 | 2.81 | 4.02 | 8.67 | 6.35 | **-0.77** | **-16** |  | |  | **-0.73** | | **-30** |
| P24593 | Insulin-like growth factor-binding protein 5 | IGFBP5 | 23 | 3.09 | 2.32 | 2.71 | 6.17 | 6.08 | 6.12 | **-0.77** | **-15** |  | |  | 0.01 | | 1 |
| P01024 | Complement C3 | C3 | 23 | 2.52 | 1.58 | 2.05 | 5.93 | 4.41 | 5.17 | **-0.87** | **-11** |  | |  | 0.30 | | 8 |
| P35527 | Keratin, type I cytoskeletal 9 | KRT9 | 18 | 5.92 | 1.93 | 3.93 | 14.85 | 6.16 | 10.50 | **-0.91** | **-47** |  | |  | **0.83** | | **91** |
| P02452 | Collagen alpha-1(I) chain | COL1A1 | 20 | 5.12 | 4.09 | 4.61 | 19.26 | 14.98 | 17.12 | **-1.15** | **-136** |  | |  | 0.25 | | 73 |
| Q99715 | Collagen alpha-1(XII) chain | COL12A1 | 11 | 0.00 | 1.14 | 0.57 | 7.52 | 3.45 | 5.48 | **-1.62** | **-15** |  | |  | **0.74** | | **22** |
