# Supplementary material for: Comprehensive Profiling of Secretome Formulations from Fetal- and Perinatal Human Amniotic Fluid Stem Cells
Source: Int J Mol Sci. 2021 Apr 2;22(7):3713. doi: 10.3390/ijms22073713 (PMC8038201; doi:10.3390/ijms22073713)
Supplement: Supplementary file 1 [file ijms-22-03713-s001.zip › Costa A et al_Supplementary Files/Costa A et al_Table S3.docx]

**Table S3. Complete list of differentially expressed proteins extracted from MAProMa comparison of fetal hAFS-EVs versus perinatal hAFS-EVs.** For each protein Uniprot Accession, Gene name, Reference, Frequency in hAFS-CM, aPSM are reported in fetal hAFS-EVs (f-hAFS-EVs) and perinatal hAFS-EVs (p-hAFS-EVs) according to in vitro cell hypoxic preconditioning (f-hAFS-EVs_normo_; f-hAFS-EVs_hypo;_ p-hAFS-EVs_normo_ and p-hAFS-EVs_hypo,_ respectively). Table shows DAve and DCI value calculated by MAProMa software for: comparison of f-hAFS-EVs versus p-hAFS-EVs; for f-hAFS-EVs_normo_ vs f-hAFS-EVs_hypo_ within up-regulated f-hAFS-EV proteins resulting from the comparison with p-hAFS-EVs, and for p-hAFS-EVs_normo_ vs p-hAFS-EVs_hypo_ of up-regulated p-hAFS-EV proteins resulting from the comparison with f-hAFS-EVs. Frequency indicates how many times a given protein has been identified under the examined conditions (total n=24 runs for hAFS-CM). The aPSM indicates that average values are given for each protein of the same condition. Positive values for DAve and DCI indicate that the corresponding protein is more abundant in the first compared condition, while negative values in the second ones. DAve and DCI values are reported in bold if they exceed the imposed thresholds. For further details regarding the meaning and the confidence range applied to DAve and DCI see Materials & Methods section.

| **Uniprot Accession** | **Reference** | **Gene Name** | **Frequency**  **hAFS-EVs** | **aPSMs hAFS-EVs** | | | | | | **MAProMa** | | | | | |
| --- | --- | --- | --- | --- | --- | --- | --- | --- | --- | --- | --- | --- | --- | --- | --- |
|  |  |  |  |  |  |  |  |  |  | **f-hAFS-EVs vs p-hAFS-EVs** | | **f-hAFS-EVs_normo_ vs f-hAFS-EVs_hypo_** | | **p-hAFS-Evs_normo_ vs p-hAFS-EVs_hypo_** | |
|  |  |  |  | **f-hAFS-**  **EVs_normo_** | **f-hAFS-**  **EVs_hypo_** | **f-hAFS-**  **EVs** | **p-hAFS-**  **EVs_normo_** | **p-hAFS-**  **EVs_hypo_** | **p-hAFS-**  **EVs** | **DAve** | **DCI** | **DAve** | **DCI** | **DAve** | **DCI** |
| P98160 | Basement membrane-specific heparan sulfate proteoglycan core protein | HSPG2 | 4 | 0.00 | 7.24 | 3.62 | 0.00 | 0.00 | 0.00 | **2.00** | **7** | **-2.00** | **-26** |  |  |
| P07996 | Thrombospondin-1 | THBS1 | 10 | 6.92 | 14.47 | 10.69 | 0.15 | 0.00 | 0.08 | **1.97** | **57** | **-0.71** | **-81** |  |  |
| Q86YZ3 | Hornerin | HRNR | 14 | 3.47 | 6.75 | 5.11 | 0.88 | 0.46 | 0.67 | **1.54** | **13** | **-0.64** | **-17** |  |  |
| G3XAI2 | Laminin subunit beta-1 | LAMB1 | 10 | 2.68 | 4.76 | 3.72 | 0.65 | 0.36 | 0.51 | **1.52** | **7** | **-0.56** | **-8** |  |  |
| P05783 | Keratin, type I cytoskeletal 18 | KRT18 | 19 | 5.46 | 5.04 | 5.25 | 1.03 | 0.61 | 0.82 | **1.46** | **13** | 0.08 | 2 |  |  |
| O00468 | Agrin | AGRN | 17 | 1.85 | 20.95 | 11.40 | 3.34 | 0.69 | 2.01 | **1.40** | **63** | **-1.68** | **-218** |  |  |
| A0A494C0G5 | Agrin | AGRN | 17 | 1.85 | 23.84 | 12.84 | 3.94 | 0.87 | 2.40 | **1.37** | **80** | **-1.71** | **-282** |  |  |
| P13611 | Versican core protein | VCAN | 19 | 17.01 | 22.39 | 19.70 | 4.99 | 4.48 | 4.73 | **1.22** | **183** | -0.27 | -106 |  |  |
| Q15323 | Keratin, type I cuticular Ha1 | KRT31 | 17 | 4.19 | 3.35 | 3.77 | 1.18 | 0.76 | 0.97 | **1.18** | **7** | 0.22 | 3 |  |  |
| P55060 | Exportin-2 | CSE1L | 18 | 4.13 | 2.20 | 3.16 | 1.38 | 0.51 | 0.95 | **1.08** | **5** | **0.61** | **6** |  |  |
| P52926 | High mobility group protein HMGI-C | HMGA2 | 23 | 9.85 | 6.69 | 8.27 | 2.52 | 2.55 | 2.53 | **1.06** | **31** | 0.38 | 26 |  |  |
| P13647 | Keratin, type II cytoskeletal 5 | KRT5 | 23 | 11.45 | 21.22 | 16.33 | 6.91 | 3.10 | 5.00 | **1.06** | **121** | **-0.60** | **-160** |  |  |
| P08727 | Keratin, type I cytoskeletal 19 | KRT19 | 22 | 13.02 | 10.38 | 11.70 | 4.11 | 3.10 | 3.61 | **1.06** | **62** | 0.23 | 31 |  |  |
| O00232 | 26S proteasome non-ATPase regulatory subunit 12 | PSMD12 | 11 | 5.92 | 1.74 | 3.83 | 2.02 | 0.48 | 1.25 | **1.02** | **7** | **1.09** | **16** |  |  |
| P05787 | Keratin, type II cytoskeletal 8 | KRT8 | 24 | 9.16 | 9.20 | 9.18 | 3.31 | 2.75 | 3.03 | **1.01** | **38** | 0.00 | 0 |  |  |
| P35221 | Catenin alpha-1 | CTNNA1 | 17 | 4.80 | 4.27 | 4.53 | 1.47 | 1.58 | 1.52 | **0.99** | **9** | 0.12 | 2 |  |  |
| Q7Z3Y7 | Keratin, type I cytoskeletal 28 | KRT28 | 18 | 9.27 | 8.98 | 9.13 | 3.84 | 2.59 | 3.22 | **0.96** | **36** | 0.03 | 3 |  |  |
| Q5XKE5 | Keratin, type II cytoskeletal 79 | KRT79 | 17 | 3.50 | 7.27 | 5.38 | 2.19 | 1.68 | 1.93 | **0.94** | **13** | **-0.70** | **-20** |  |  |
| P35908 | Keratin, type II cytoskeletal 2 epidermal | KRT2 | 20 | 33.12 | 34.34 | 33.73 | 14.11 | 10.65 | 12.38 | **0.93** | **492** | -0.04 | -41 |  |  |
| P02533 | Keratin, type I cytoskeletal 14 | KRT14 | 22 | 13.80 | 23.83 | 18.81 | 9.50 | 4.32 | 6.91 | **0.93** | **153** | **-0.53** | **-189** |  |  |
| O15230 | Laminin subunit alpha-5 | LAMA5 | 20 | 3.75 | 9.89 | 6.82 | 2.39 | 2.91 | 2.65 | **0.88** | **20** | **-0.90** | **-42** |  |  |
| P35527 | Keratin, type I cytoskeletal 9 | KRT9 | 21 | 52.47 | 92.12 | 72.29 | 42.80 | 16.31 | 29.56 | **0.84** | **2176** | **-0.55** | **-2867** |  |  |
| P05106 | Integrin beta-3 | ITGB3 | 17 | 8.34 | 11.28 | 9.81 | 2.71 | 5.49 | 4.10 | **0.82** | **40** | -0.30 | -29 |  |  |
| P04264 | Keratin, type II cytoskeletal 1 | KRT1 | 24 | 64.88 | 103.48 | 84.18 | 49.51 | 23.26 | 36.38 | **0.79** | **2881** | **-0.46** | **-3249** |  |  |
| P62328 | Thymosin beta-4 | TMSB4X | 19 | 7.61 | 4.26 | 5.93 | 2.28 | 3.05 | 2.67 | **0.76** | **14** | **0.57** | **20** |  |  |
| P46940 | Ras GTPase-activating-like protein IQGAP1 | IQGAP1 | 12 | 4.23 | 2.58 | 3.41 | 2.65 | 0.46 | 1.55 | **0.75** | **5** | **0.48** | **6** |  |  |
| P49327 | Fatty acid synthase | FASN | 17 | 9.02 | 7.17 | 8.09 | 2.69 | 4.85 | 3.77 | **0.73** | **26** | 0.23 | 15 |  |  |
| P08729 | Keratin, type II cytoskeletal 7 | KRT7 | 20 | 2.66 | 4.90 | 3.78 | 1.94 | 1.94 | 1.94 | **0.64** | **5** | **-0.59** | **-8** |  |  |
| P26022 | Pentraxin-related protein PTX3 | PTX3 | 16 | 4.61 | 3.29 | 3.95 | 1.76 | 2.31 | 2.03 | **0.64** | **6** | 0.34 | 5 |  |  |
| P31946 | 14-3-3 protein beta/alpha | YWHAB | 22 | 4.39 | 6.26 | 5.32 | 2.03 | 3.56 | 2.80 | **0.62** | **10** | -0.35 | -10 |  |  |
| Q04695 | Keratin, type I cytoskeletal 17 | KRT17 | 20 | 8.69 | 10.30 | 9.50 | 6.28 | 3.71 | 4.99 | **0.62** | **33** | -0.17 | -15 |  |  |
| A0A1W2PQ51 | Probable ATP-dependent RNA helicase DDX17 | DDX17 | 22 | 4.55 | 3.11 | 3.83 | 2.48 | 1.63 | 2.05 | **0.61** | **5** | 0.38 | 6 |  |  |
| P13645 | Keratin, type I cytoskeletal 10 | KRT10 | 23 | 55.20 | 53.58 | 54.39 | 32.82 | 25.53 | 29.18 | **0.60** | **1054** | 0.03 | 88 |  |  |
| P80723 | Brain acid soluble protein 1 | BASP1 | 24 | 11.00 | 13.72 | 12.36 | 5.78 | 7.71 | 6.74 | **0.59** | **54** | -0.22 | -34 |  |  |
| P02538 | Keratin, type II cytoskeletal 6A | KRT6A | 24 | 11.90 | 15.14 | 13.52 | 11.25 | 3.74 | 7.50 | **0.57** | **63** | -0.24 | -44 |  |  |
| P19338 | Nucleolin | NCL | 24 | 9.79 | 5.31 | 7.55 | 4.49 | 4.04 | 4.27 | **0.56** | **19** | **0.59** | **34** |  |  |
| P04259 | Keratin, type II cytoskeletal 6B | KRT6B | 24 | 15.49 | 17.75 | 16.62 | 13.06 | 6.13 | 9.59 | **0.54** | **92** | -0.14 | -38 |  |  |
| P62070 | Ras-related protein R-Ras2 | RRAS2 | 21 | 3.64 | 3.87 | 3.75 | 2.30 | 2.09 | 2.20 | **0.52** | **5** | -0.06 | -1 |  |  |
| P46781 | 40S ribosomal protein S9 | RPS9 | 16 | 7.99 | 2.06 | 5.02 | 2.23 | 3.67 | 2.95 | **0.52** | **8** | **1.18** | **30** |  |  |
| P08779 | Keratin, type I cytoskeletal 16 | KRT16 | 22 | 12.96 | 13.78 | 13.37 | 11.95 | 4.32 | 8.13 | **0.49** | **56** | -0.06 | -11 |  |  |
| P13010 | X-ray repair cross-complementing protein 5 | XRCC5 | 14 | 10.30 | 3.80 | 7.05 | 5.79 | 3.04 | 4.41 | **0.46** | **15** | **0.92** | **46** |  |  |
| Q00839 | Heterogeneous nuclear ribonucleoprotein U | HNRNPU | 19 | 8.53 | 4.85 | 6.69 | 7.49 | 1.06 | 4.28 | **0.44** | **13** | **0.55** | **25** |  |  |
| Q14204 | Cytoplasmic dynein 1 heavy chain 1 | DYNC1H1 | 17 | 7.76 | 2.22 | 4.99 | 3.22 | 3.25 | 3.23 | **0.43** | **7** | **1.11** | **28** |  |  |
| G3V4C1 | Heterogeneous nuclear ribonucleoproteins C1/C2 | HNRNPC | 24 | 10.51 | 6.46 | 8.49 | 6.32 | 4.75 | 5.53 | **0.42** | **21** | **0.48** | **34** |  |  |
| P37802 | Transgelin-2 | TAGLN2 | 23 | 2.42 | 3.33 | 2.87 | 3.87 | 4.75 | 4.31 | **-0.40** | **-5** |  |  | -0.20 | -4 |
| P04004 | Vitronectin | VTN | 21 | 3.10 | 2.56 | 2.83 | 4.64 | 3.87 | 4.25 | **-0.40** | **-5** |  |  | 0.18 | 3 |
| Q01995 | Transgelin | TAGLN | 19 | 3.69 | 4.72 | 4.21 | 5.47 | 7.18 | 6.32 | **-0.40** | **-11** |  |  | -0.27 | -11 |
| P13639 | Elongation factor 2 | EEF2 | 24 | 27.05 | 24.57 | 25.81 | 41.76 | 35.99 | 38.87 | **-0.40** | **-423** |  |  | 0.15 | 224 |
| P11233 | Ras-related protein Ral-A | RALA | 24 | 5.91 | 3.94 | 4.93 | 6.45 | 8.80 | 7.62 | **-0.43** | **-17** |  |  | -0.31 | -18 |
| P40227 | T-complex protein 1 subunit zeta | CCT6A | 21 | 13.28 | 10.15 | 11.71 | 18.73 | 17.60 | 18.16 | **-0.43** | **-96** |  |  | 0.06 | 21 |
| P68431 | Histone H3.1 | H3C1 | 24 | 9.79 | 6.53 | 8.16 | 13.53 | 11.87 | 12.70 | **-0.44** | **-47** |  |  | 0.13 | 21 |
| P62906 | 60S ribosomal protein L10a | RPL10A | 21 | 3.89 | 1.66 | 2.77 | 4.49 | 4.21 | 4.35 | **-0.44** | **-6** |  |  | 0.07 | 1 |
| P01893 | Putative HLA class I histocompatibility antigen, alpha chain H | HLA-H | 20 | 2.13 | 3.02 | 2.58 | 3.77 | 4.38 | 4.07 | **-0.45** | **-5** |  |  | -0.15 | -2 |
| P02452 | Collagen alpha-1(I) chain | COL1A1 | 17 | 6.67 | 9.45 | 8.06 | 14.83 | 10.88 | 12.86 | **-0.46** | **-50** |  |  | 0.31 | 51 |
| P62266 | 40S ribosomal protein S23 | RPS23 | 21 | 3.04 | 2.47 | 2.76 | 4.63 | 4.35 | 4.49 | **-0.48** | **-6** |  |  | 0.06 | 1 |
| P50991 | T-complex protein 1 subunit delta | CCT4 | 18 | 2.12 | 4.19 | 3.15 | 5.53 | 4.80 | 5.16 | **-0.48** | **-8** |  |  | 0.14 | 4 |
| P29966 | Myristoylated alanine-rich C-kinase substrate | MARCKS | 24 | 4.16 | 5.00 | 4.58 | 6.98 | 8.05 | 7.51 | **-0.48** | **-18** |  |  | -0.14 | -8 |
| P16401 | Histone H1.5 | H1-5 | 21 | 10.92 | 6.80 | 8.86 | 15.43 | 13.84 | 14.64 | **-0.49** | **-68** |  |  | 0.11 | 23 |
| A0A140T913 | HLA class I histocompatibility antigen, A alpha chain | HLA-A | 21 | 2.07 | 3.36 | 2.72 | 4.16 | 4.84 | 4.50 | **-0.50** | **-6** |  |  | -0.15 | -3 |
| Q16658 | Fascin | FSCN1 | 23 | 1.93 | 4.16 | 3.05 | 3.03 | 7.10 | 5.06 | **-0.50** | **-8** |  |  | **-0.80** | **-21** |
| P11234 | Ras-related protein Ral-B | RALB | 24 | 4.46 | 2.92 | 3.69 | 4.76 | 7.50 | 6.13 | **-0.50** | **-12** |  |  | **-0.45** | **-17** |
| P48643 | T-complex protein 1 subunit epsilon | CCT5 | 23 | 7.47 | 8.29 | 7.88 | 14.37 | 11.89 | 13.13 | **-0.50** | **-55** |  |  | 0.19 | 33 |
| P60953 | Cell division control protein 42 homolog | CDC42 | 21 | 3.06 | 3.08 | 3.07 | 4.92 | 5.37 | 5.14 | **-0.50** | **-9** |  |  | -0.09 | -2 |
| P05556 | Integrin beta-1 | ITGB1 | 24 | 39.04 | 45.77 | 42.41 | 65.05 | 78.08 | 71.56 | **-0.51** | **-1661** |  |  | -0.18 | -932 |
| O75369 | Filamin-B | FLNB | 20 | 3.86 | 2.11 | 2.98 | 5.99 | 4.09 | 5.04 | **-0.51** | **-8** |  |  | 0.38 | 10 |
| P55072 | Transitional endoplasmic reticulum ATPase | VCP | 23 | 3.67 | 6.84 | 5.25 | 7.18 | 10.69 | 8.94 | **-0.52** | **-26** |  |  | -0.39 | -31 |
| C9J4V0 | Ras-related protein Rab-7a | RAB7A | 20 | 3.37 | 4.82 | 4.09 | 6.08 | 7.96 | 7.02 | **-0.53** | **-16** |  |  | -0.27 | -13 |
| Q9Y490 | Talin-1 | TLN1 | 20 | 2.30 | 2.65 | 2.47 | 4.77 | 3.72 | 4.24 | **-0.53** | **-6** |  |  | 0.25 | 4 |
| P53396 | ATP-citrate synthase | ACLY | 19 | 5.08 | 4.71 | 4.89 | 9.01 | 8.25 | 8.63 | **-0.55** | **-25** |  |  | 0.09 | 7 |
| P83731 | 60S ribosomal protein L24 | RPL24 | 22 | 3.85 | 2.34 | 3.10 | 4.64 | 6.38 | 5.51 | **-0.56** | **-10** |  |  | -0.32 | -10 |
| P46778 | 60S ribosomal protein L21 | RPL21 | 17 | 3.81 | 1.11 | 2.46 | 4.35 | 4.40 | 4.38 | **-0.56** | **-7** |  |  | -0.01 | 0 |
| A0A0G2JPD3 | HLA class I histocompatibility antigen, A alpha chain | HLA-A | 21 | 2.07 | 3.36 | 2.72 | 4.31 | 5.41 | 4.86 | **-0.57** | **-8** |  |  | -0.23 | -5 |
| Q15758 | Neutral amino acid transporter B(0) | SLC1A5 | 19 | 3.01 | 2.84 | 2.92 | 4.58 | 5.89 | 5.24 | **-0.57** | **-9** |  |  | -0.25 | -7 |
| A0A2R8Y7X9 | GLOBIN domain-containing protein |  | 21 | 1.43 | 2.63 | 2.03 | 3.41 | 3.89 | 3.65 | **-0.57** | **-5** |  |  | -0.13 | -2 |
| Q08380 | Galectin-3-binding protein | LGALS3BP | 23 | 23.08 | 30.15 | 26.62 | 46.01 | 50.01 | 48.01 | **-0.57** | **-798** |  |  | -0.08 | -192 |
| Q9P2B2 | Prostaglandin F2 receptor negative regulator | PTGFRN | 21 | 14.48 | 21.36 | 17.92 | 31.70 | 33.31 | 32.51 | **-0.58** | **-368** |  |  | -0.05 | -52 |
| P31949 | Protein S100-A11 | S100A11 | 23 | 2.28 | 1.75 | 2.01 | 3.41 | 4.02 | 3.72 | **-0.60** | **-5** |  |  | -0.16 | -2 |
| P05386 | 60S acidic ribosomal protein P1 | RPLP1 | 20 | 3.75 | 1.61 | 2.68 | 5.16 | 4.74 | 4.95 | **-0.60** | **-9** |  |  | 0.09 | 2 |
| P69905 | Hemoglobin subunit alpha | HBA1 | 23 | 4.25 | 6.08 | 5.16 | 9.47 | 9.67 | 9.57 | **-0.60** | **-32** |  |  | -0.02 | -2 |
| P25787 | Proteasome subunit alpha type-2 | PSMA2 | 21 | 2.58 | 2.27 | 2.42 | 4.34 | 5.06 | 4.70 | **-0.64** | **-8** |  |  | -0.15 | -3 |
| P62314 | Small nuclear ribonucleoprotein Sm D1 | SNRPD1 | 19 | 5.46 | 2.22 | 3.84 | 8.22 | 6.82 | 7.52 | **-0.65** | **-21** |  |  | 0.19 | 10 |
| P62244 | 40S ribosomal protein S15a | RPS15A | 18 | 3.97 | 0.95 | 2.46 | 4.67 | 5.01 | 4.84 | **-0.65** | **-9** |  |  | -0.07 | -2 |
| P28072 | Proteasome subunit beta type-6 | PSMB6 | 21 | 1.98 | 3.54 | 2.76 | 5.04 | 5.86 | 5.45 | **-0.66** | **-11** |  |  | -0.15 | -4 |
| Q14764 | Major vault protein | MVP | 23 | 9.43 | 14.11 | 11.77 | 19.78 | 28.32 | 24.05 | **-0.69** | **-220** |  |  | -0.36 | -205 |
| P05387 | 60S acidic ribosomal protein P2 | RPLP2 | 11 | 4.65 | 0.00 | 2.33 | 5.86 | 3.90 | 4.88 | **-0.71** | **-9** |  |  | **0.40** | **10** |
| F5H423 | Uncharacterized protein |  | 19 | 2.18 | 2.72 | 2.45 | 4.77 | 5.89 | 5.33 | **-0.74** | **-11** |  |  | -0.21 | -6 |
| P50281 | Matrix metalloproteinase-14 | MMP14 | 17 | 2.42 | 2.50 | 2.46 | 4.04 | 6.70 | 5.37 | **-0.74** | **-11** |  |  | **-0.50** | **-14** |
| P08133 | Annexin A6 | ANXA6 | 24 | 14.50 | 18.31 | 16.41 | 25.34 | 46.50 | 35.92 | **-0.75** | **-511** |  |  | **-0.59** | **-760** |
| P29317 | Ephrin type-A receptor 2 | EPHA2 | 18 | 6.46 | 4.79 | 5.63 | 11.90 | 12.89 | 12.40 | **-0.75** | **-61** |  |  | -0.08 | -12 |
| Q14195 | Dihydropyrimidinase-related protein 3 | DPYSL3 | 22 | 2.90 | 3.34 | 3.12 | 8.54 | 5.60 | 7.07 | **-0.77** | **-20** |  |  | **0.42** | **21** |
| P01023 | Alpha-2-macroglobulin | A2M | 21 | 4.42 | 4.24 | 4.33 | 9.02 | 11.21 | 10.11 | **-0.80** | **-42** |  |  | -0.22 | -22 |
| P62873 | Guanine nucleotide-binding protein G(I)/G(S)/G(T) subunit beta-1 | GNB1 | 21 | 2.12 | 1.52 | 1.82 | 4.03 | 4.53 | 4.28 | **-0.81** | **-8** |  |  | -0.12 | -2 |
| P28066 | Proteasome subunit alpha type-5 | PSMA5 | 20 | 0.91 | 1.99 | 1.45 | 2.77 | 4.41 | 3.59 | **-0.85** | **-5** |  |  | **-0.46** | **-6** |
| O14818 | Proteasome subunit alpha type-7 | PSMA7 | 21 | 2.03 | 2.90 | 2.47 | 5.71 | 7.20 | 6.45 | **-0.89** | **-18** |  |  | -0.23 | -10 |
| P50395 | Rab GDP dissociation inhibitor beta | GDI2 | 19 | 3.36 | 2.29 | 2.83 | 4.16 | 10.64 | 7.40 | **-0.89** | **-23** |  |  | **-0.88** | **-48** |
| P67809 | Y-box-binding protein 1 | YBX1 | 15 | 2.25 | 1.63 | 1.94 | 4.64 | 6.57 | 5.60 | **-0.97** | **-14** |  |  | -0.34 | -11 |
| P27105 | Erythrocyte band 7 integral membrane protein | STOM | 22 | 2.86 | 4.45 | 3.65 | 8.88 | 13.61 | 11.25 | **-1.02** | **-57** |  |  | **-0.42** | **-53** |
| J3KQ42 | Tetraspanin | TSPAN4 | 20 | 1.19 | 0.84 | 1.01 | 3.25 | 3.18 | 3.21 | **-1.04** | **-5** |  |  | 0.02 | 0 |
| P30626 | Sorcin | SRI | 20 | 0.89 | 1.29 | 1.09 | 3.39 | 3.84 | 3.61 | **-1.07** | **-6** |  |  | -0.12 | -2 |
| P09382 | Galectin-1 | LGALS1 | 18 | 0.85 | 1.19 | 1.02 | 3.62 | 3.22 | 3.42 | **-1.08** | **-5** |  |  | 0.12 | 1 |
| O75955 | Flotillin-1 | FLOT1 | 16 | 0.14 | 2.13 | 1.14 | 1.94 | 5.73 | 3.84 | **-1.09** | **-7** |  |  | **-0.99** | **-15** |
| P28070 | Proteasome subunit beta type-4 | PSMB4 | 20 | 1.57 | 1.97 | 1.77 | 5.00 | 7.33 | 6.17 | **-1.11** | **-17** |  |  | -0.38 | -14 |
| P28074 | Proteasome subunit beta type-5 | PSMB5 | 17 | 0.62 | 1.27 | 0.95 | 3.94 | 4.25 | 4.10 | **-1.25** | **-8** |  |  | -0.07 | -1 |
| Q99436 | Proteasome subunit beta type-7 | PSMB7 | 18 | 0.46 | 1.13 | 0.80 | 2.81 | 4.59 | 3.70 | **-1.29** | **-7** |  |  | **-0.48** | **-7** |
| P02794 | Ferritin heavy chain | FTH1 | 14 | 0.16 | 2.00 | 1.08 | 1.93 | 8.12 | 5.03 | **-1.29** | **-12** |  |  | **-1.23** | **-31** |
| P25786 | Proteasome subunit alpha type-1 | PSMA1 | 17 | 0.46 | 0.95 | 0.71 | 3.59 | 4.90 | 4.24 | **-1.43** | **-9** |  |  | -0.31 | -6 |
| E9PNQ8 | Thy-1 membrane glycoprotein (Fragment) | THY1 | 16 | 1.10 | 0.18 | 0.64 | 2.29 | 5.47 | 3.88 | **-1.43** | **-7** |  |  | **-0.82** | **-12** |
| Q96AC1 | Fermitin family homolog 2 | FERMT2 | 17 | 0.32 | 0.84 | 0.58 | 6.73 | 7.40 | 7.06 | **-1.70** | **-25** |  |  | -0.10 | -5 |
| O14786 | Neuropilin-1 | NRP1 | 14 | 0.16 | 0.16 | 0.16 | 2.49 | 4.89 | 3.69 | **-1.83** | **-7** |  |  | **-0.65** | **-9** |
