# Supplementary material for: Comprehensive Profiling of Secretome Formulations from Fetal- and Perinatal Human Amniotic Fluid Stem Cells
Source: Int J Mol Sci. 2021 Apr 2;22(7):3713. doi: 10.3390/ijms22073713 (PMC8038201; doi:10.3390/ijms22073713)
Supplement: Supplementary file 1 [file ijms-22-03713-s001.zip › Costa A et al_Supplementary Files/Costa A et al_ Supplementary Materials.docx]

**Costa A et al. Supplementary Figures**

**
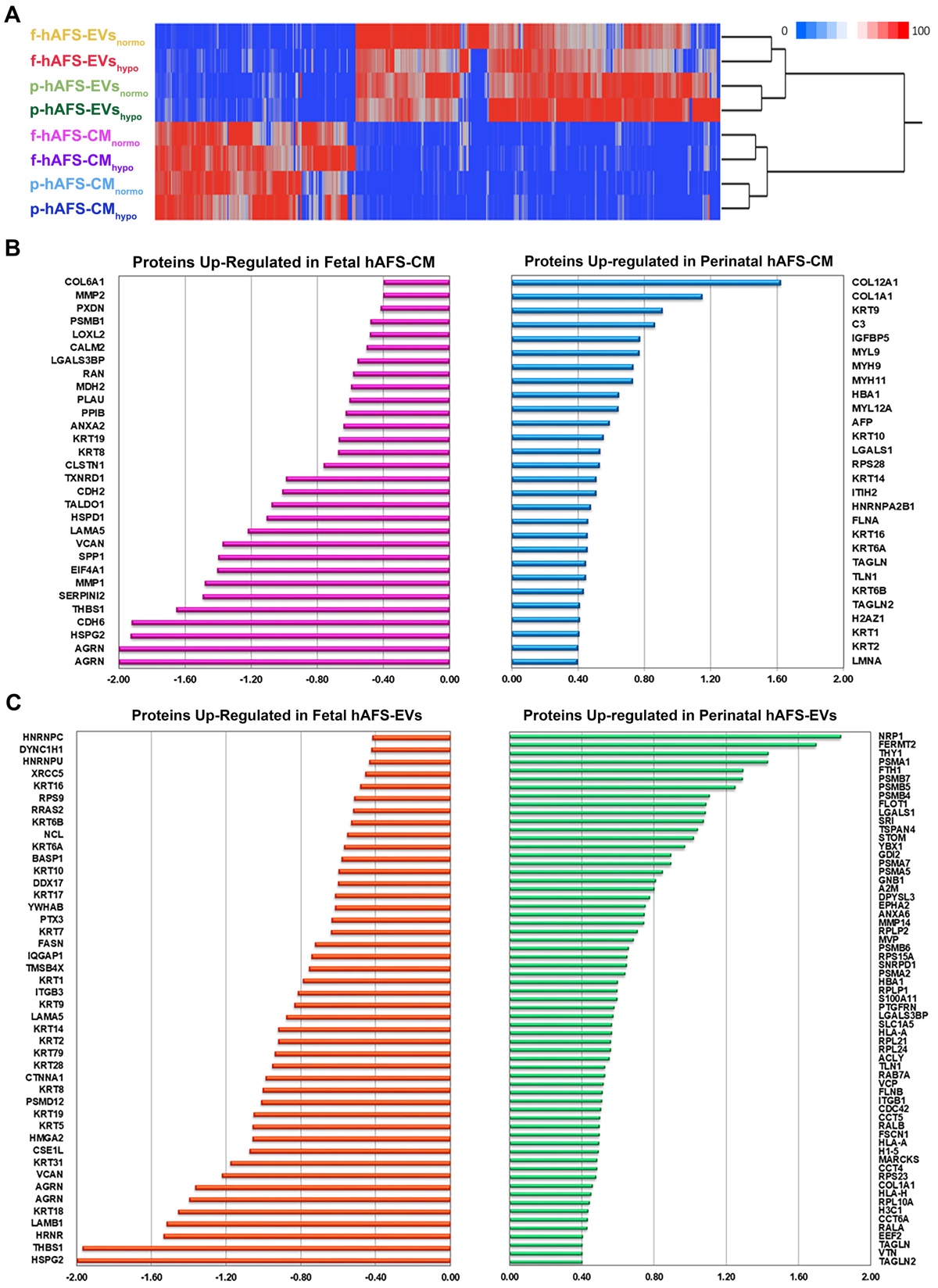
**

**Figure S1. Supplementary data related to proteomic analysis of fetal- and perinatal hAFS secretome fractions. (A)** Dendogram of average protein lists from fetal- versus perinatal hAFS-CM (f-hAFS-CM and p-hAFS-CM, respectively) and hAFS-EVs (f-hAFS-EVs and p-hAFS-EVs, respectively), according to normoxic control versus hypoxic cell preconditioning. Clustering was performed by computing the average peptide spectrum matches (aPSM) of proteins selected by Linear Discriminant Analysis (LDA); Euclidean’s distance metric and Ward’s methods were applied (JMP15.2 software). (**B**) Histogram plots of the fetal hAFS-CM versus perinatal hAFS-CM comparison, with negative DAve values corresponding to up-regulated proteins in f-hAFS-CM (left panel, pink bars) and positive DAve values corresponding to up-regulated proteins in p-hAFS-CM (right panel, blue bars). **(C)** Histogram plots of the fetal hAFS-EVs versus perinatal hAFS-EVs comparison, with negative DAve values corresponding to up-regulated proteins in fetal hAFS-EVs (left panel, red bars) and positive DAve values corresponding to up-regulated ones in perinatal hAFS-EVs (right panel, green bars). In the above pairwise comparisons obtained by MAProMa software, negative DAve values in the left panels indicate that a specific protein is more abundant in the first compared condition, while positive DAve values in the right panels indicate proteins upregulated in the second condition.


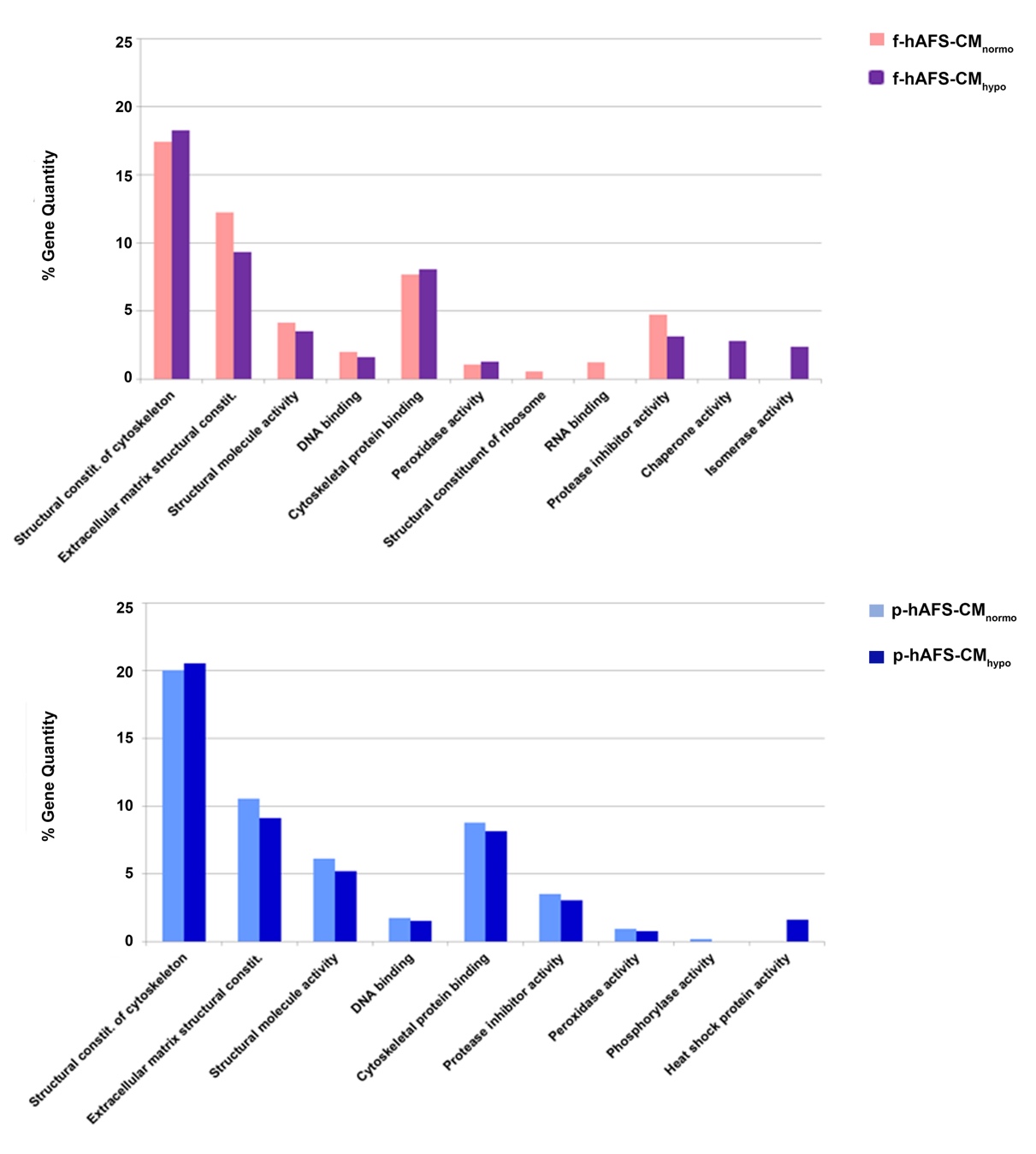


**Figure S2. Supplementary data related to proteomic enrichment analysis of fetal- and perinatal hAFS-CM.** Molecular function enrichment analysis of proteins identified with a frequency of at least 2 in fetal hAFS-CM (upper panel) and in perinatal hAFS-CM (lower panel), according to normoxic (f-hAFS-CM_normo_ and p-hAFS-CM_normo_) versus hypoxic (f-hAFS-CM_hypo_ and p-hAFS-CM_hypo_) cell preconditioning in vitro. Using FunRich tool, gene ontology terms are shown in bar charts reporting the percentage of genes enriched for each category (pink bars for f-hAFS-CM_normo_, purple bars for f-hAFS-CM_hypo_, light blue bars for p-hAFS-CM_normo_ and blue bars for p-hAFS-CM_hypo_). Only gene ontology terms with Bonferroni corrected *p<0.05 for molecular function are reported.

**
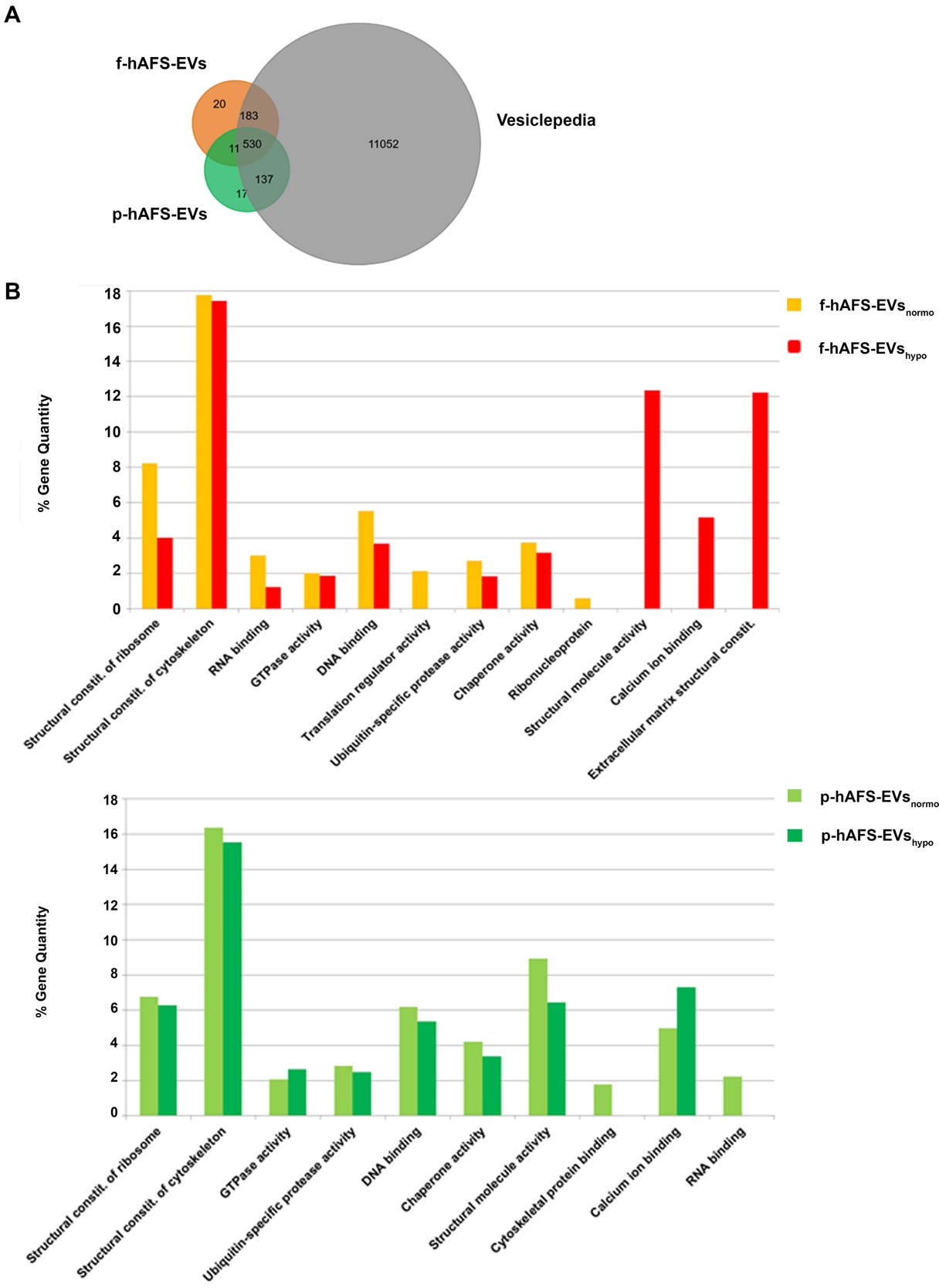
**

**Figure S3. Supplementary data related to proteomic enrichment analysis of fetal- and perinatal hAFS-EVs and comparison against Vesiclepedia database. (A)** Venn diagrams illustrating the comparison of proteins detected with a frequency at least of 2 in fetal hAFS-EVs (f-hAFS-EVs: 744 gene entries) and in perinatal hAFS-EVs (p-hAFS-EVs: 695 gene entries) against the Vesiclepedia database; for the overlay analysis, all gene entries from Vesiclepedia were downloaded, imported in Funrich software and filtered for “exosomes” and “extracellular vesicles” (11902 entries on 612 experiments selected from the entire database). **(B)** Molecular function enrichment analysis of proteins identified with a frequency of at least 2 in fetal hAFS-EVs (upper panel) and perinatal hAFS-EVs (lower panel) according to normoxic control (f-hAFS-EVs_normo_ and p-hAFS-EVs_normo_) versus hypoxic (f-hAFS-EVs_hypo_ and p-hAFS-EVs_hypo_) cell preconditioning in vitro. Using FunRich tool, gene ontology terms are shown in bar charts reporting the percentage of genes enriched for each category (dark yellow bars for f-hAFS-EVs_normo_, red bars for f-hAFS-EVs_hypo_, light green bars for p-hAFS-EVs_normo_ and dark green bars for p-hAFS-EVs_hypo_). Only gene ontology terms with Bonferroni corrected *p<0.05 for molecular function are reported.


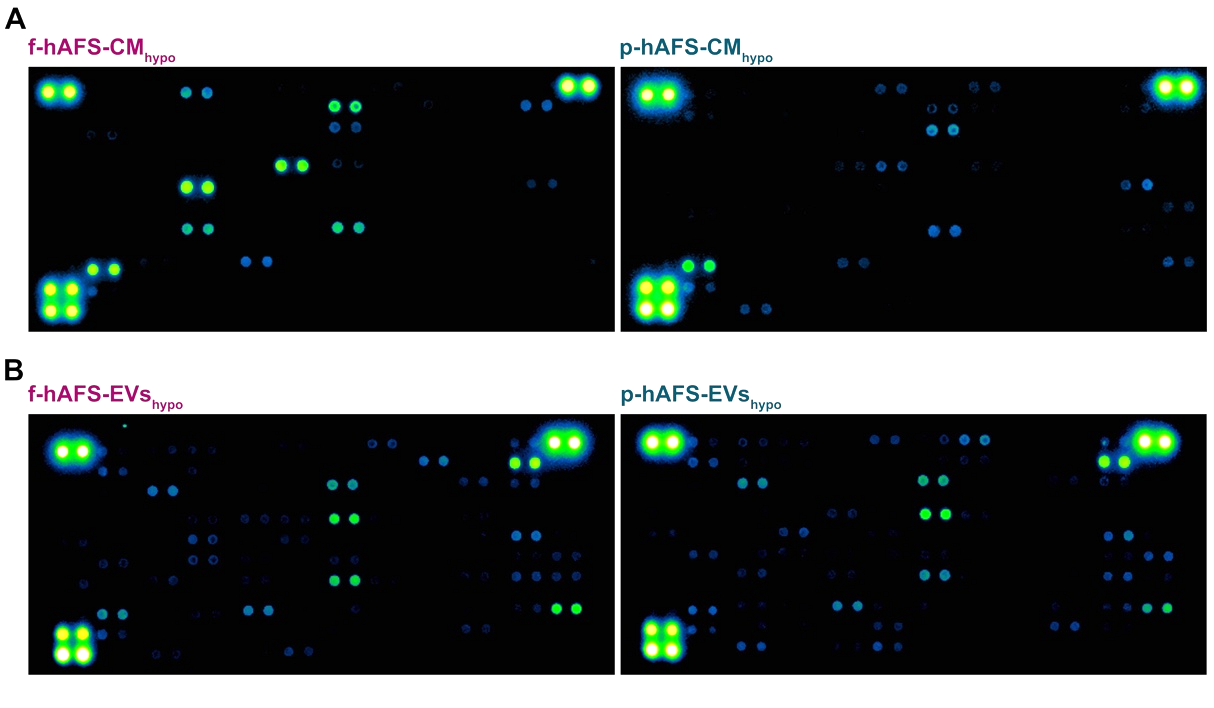


**Figure S4. Cytokine/chemokine array membranes of fetal- versus perinatal hAFS secretome fractions. (A)** Representative images of cytokine/chemokine array membranes of fetal hAFS-CM versus perinatal hAFS-CM following hypoxic cell preconditioning (f-hAFS-CM_hypo_ vs p-hAFS-CM_hypo_). **(B)** Representative images of cytokine/chemokine array spot membranes of fetal- versus perinatal hAFS-EVs following hypoxic cell preconditioning in vitro (f-hAFS-EVs_hypo_ vs p-hAFS-EVs_hypo_).

**Costa A et al. Supplementary Tables**

**Table S1. Complete list of distinct master proteins detected with at least one unique peptide in the fetal- and perinatal hAFS-CM and hAFS-EV formulations.** For each protein Uniprot Accession, Reference, Gene name, Coverage, Unique Peptides, MW, pI, average Peptide Spectrum Match (aPSM) and Frequency are reported. aPSM indicates that average values are given for each protein of the same condition. See the supplementary table file attached (Costa A et al_Table S1).

**Table S2. Complete list of differentially expressed proteins in fetal hAFS-CM versus perinatal hAFS-CM.** For each protein Uniprot Accession, Gene name, Reference, Frequency in hAFS-CM, aPSM are reported in fetal hAFS-CM (f-hAFS-CM) and perinatal hAFS-CM (p-hAFS-CM) according to in vitro cell hypoxic preconditioning (f-hAFS-CM_normo_; f-hAFS-CM_hypo_; p-hAFS-CM_normo_ and p-hAFS-CM_hypo_, respectively). For further details regarding the meaning and the confidence range applied to DAve and DCI see Materials & Methods section. See the supplementary table file attached (Costa A et al_Table S2).

**Table S3. Complete list of differentially expressed proteins in fetal hAFS-EVs versus perinatal hAFS-EVs.** For each protein Uniprot Accession, Gene name, Reference, Frequency in hAFS-CM, aPSM are reported in fetal hAFS-EVs (f-hAFS-EVs) and perinatal hAFS-EVs (p-hAFS-EVs) according to in vitro cell hypoxic preconditioning (f-hAFS-EVs_normo_; f-hAFS-EVs_hypo_; p-hAFS-EVs_normo_ and p-hAFS-EVs_hypo_, respectively). For further details regarding the meaning and the confidence range applied to DAve and DCI see Materials & Methods section. See the supplementary table file attached (Costa A et al_Table S3).

**Table S4. Quantification of detectable chemokines/cytokines within fetal- and perinatal hAFS-CM formulation from hAFS undergoing hypoxic preconditioning.** Mean ± s.e.m values refer to 106 pixel intensity by means of arbitrary unit (n=3 experiments); *n.d.: not determined*. *CST3: Cystatin C; EMMPRIN: Extracellular Matrix Metalloproteinase Inducer; FGF19: Fibroblast Growth Factor 19; IGFBP2: Insulin-like growth factor binding protein 2; IL-8: Interleukin 8; IL-17a: Interleukin 17a; OPN: Osteoponti; PTX3: Pentraxin 3; PAI-1: Plasminogen Activator Inhibitor-1.*

|  | **Fetal hAFS-CM**  **(f-hAFS-CM_hypo_)** | **Perinatal hAFS-CM**  **(p-hAFS-CM_hypo_)** |
| --- | --- | --- |
| **ANGIOGENIN** | 1.17 ± 0.30 | n.d. |
| **CST3** | 1.24 ± 0.41 | 0.25 ± 0.05 |
| **EMMPRIN** | 0.61 ± 0.06 | n.d. |
| **FGF-19** | 0.50 ± 0.04 | 0.50 ± 0.03 |
| **IGFBP-2** | 3.15 ± 0.36 | 0.88 ± 0.72 |
| **IL-8** | 4.15 ± 0.75 | n.d. |
| **IL-17a** | 0.34 ± 0.02 | 0.29 ± 0.01 |
| **MCP-1** | 1.01 ± 0.34 | n.d. |
| **MIF** | 1.20 ± 0.30 | 0.73 ± 0.52 |
| **OPN** | 3.95 ± 0.46 | 1.04 ± 0.32 |
| **PTX3** | 0.46 ± 0.12 | 0.33 ± 0.17 |
| **PAI-1** | 8.16 ± 0.52 | 4.93 ± 2.20 |

**Table S5.** Cytokine and chemokine profiling of fetal- versus perinatal hAFS-EV formulations from hAFS undergoing hypoxic preconditioning. Mean ± s.e.m values refer to pixel intensity by means of arbitrary unit; n= 3 experiments; *n.d.: not determined*. *BDNF: Brain-derived neurotrophic factor; CD40L: CD40 LIGAND; DPPIV: Dipeptidyl-peptidase IV; EMMPRIN: Extracellular Matrix Metalloproteinase Inducer; FGF19: Fibroblast Growth Factor 19; GDF-15: Growth/differentiation factor 15; IGFBP-3: IGFBP3: Insulin-like growth factor binding protein 3; IL-8; Interleukin 8; IL-17a: Interleukin 17a; OPN: Osteopontin; PTX3: Pentraxin 3; SFD-1 α: Stromal Derived Factor-1alpha; PAI-1: Plasminogen Activator Inhibitor-1; VDBP: Vitamin D binding protein.*

|  | **Fetal hAFS-EVs**  **(f-hAFS-EVs_hypo_)** | **Perinatal hAFS-EVs**  **(p-hAFS-EVs_hypo_)** |
| --- | --- | --- |
| **ANGIOPOIETIN2** | n.d. | 0.35 ± 0.08 |
| **BDNF** | 0.38 ± 0.01 | 0.44 ± 0.01 |
| **CD40L** | n.d. | 0.34 ± 0.04 |
| **DPPIV** | 0.41 ± 0.01 | n.d. |
| **EMMPRIN** | 2.27 ± 0.09 | 2.22 ± 0.27 |
| **ENDOGLIN** | 0.57 ± 0.13 | 0.44 ± 0.10 |
| **FGF-19** | 0.69 ± 0.03 | 0.83 ± 0.12 |
| **GDF-15** | 0.25 ± 0.04 | n.d. |
| **IGFBP-3** | 0.51 ± 0.21 | 0.53 ± 0.27 |
| **IL-8** | 0.37 ± 0.03 | n.d. |
| **IL-17a** | 0.46 ± 0.02 | 0.45 ± 0.07 |
| **MIF** | 0.71 ± 0.11 | 0.68 ± 0.10 |
| **OPN** | 0.69 ± 0.09 | 0.50 ± 0.10 |
| **PTX3** | 0.35 ± 0.03 | 0.42 ± 0.10 |
| **SDF-1α** | 0.85 ± 0.10 | 0.73 ± 0.12 |
| **PAI-1** | 4.09 ± 0.95 | 3.60 ± 0.70 |
| **VDBP** | n.d. | 0.30 ± 0.08 |
