# Supplementary figures and images for: Comprehensive Profiling of Secretome Formulations from Fetal- and Perinatal Human Amniotic Fluid Stem Cells
Source: Int J Mol Sci. 2021 Apr 2;22(7):3713. doi: 10.3390/ijms22073713 (PMC8038201; doi:10.3390/ijms22073713)

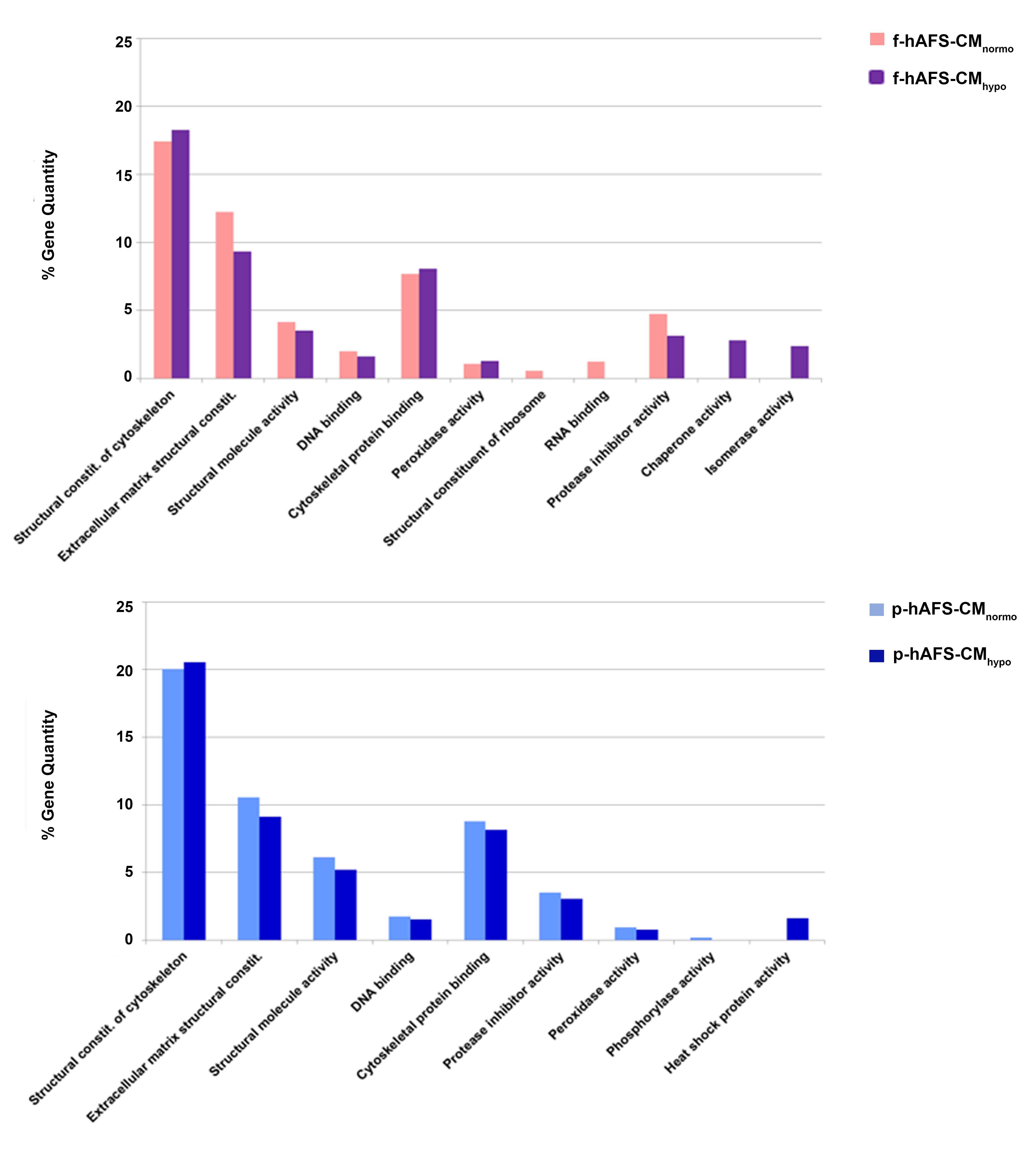

Supplement: Supplementary file 1 [file ijms-22-03713-s001.zip › Costa A et al_Supplementary Files/Costa A et al_Figure S2.tif]

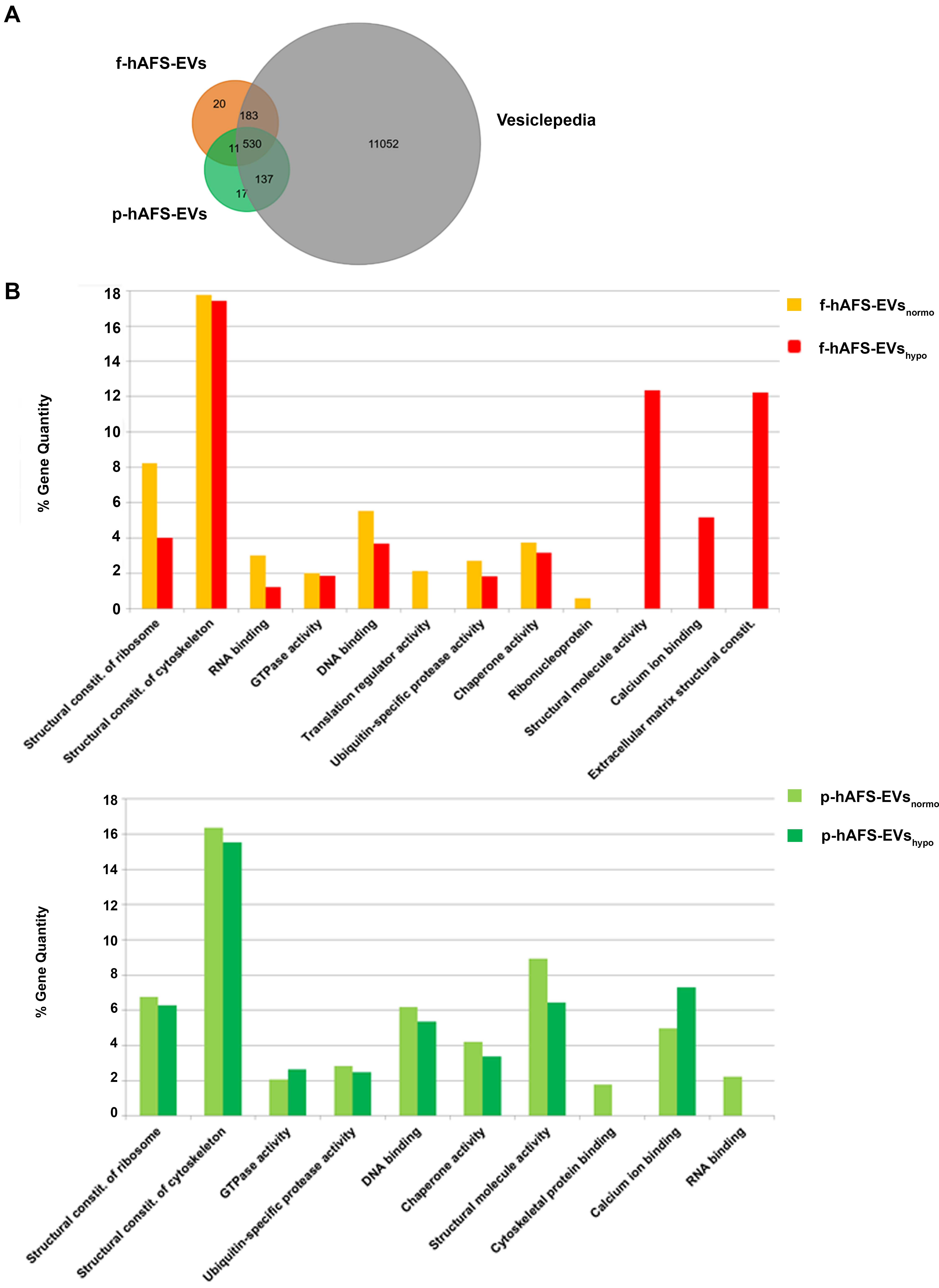

Supplement: Supplementary file 1 [file ijms-22-03713-s001.zip › Costa A et al_Supplementary Files/Costa A et al_Figure S3.tif]

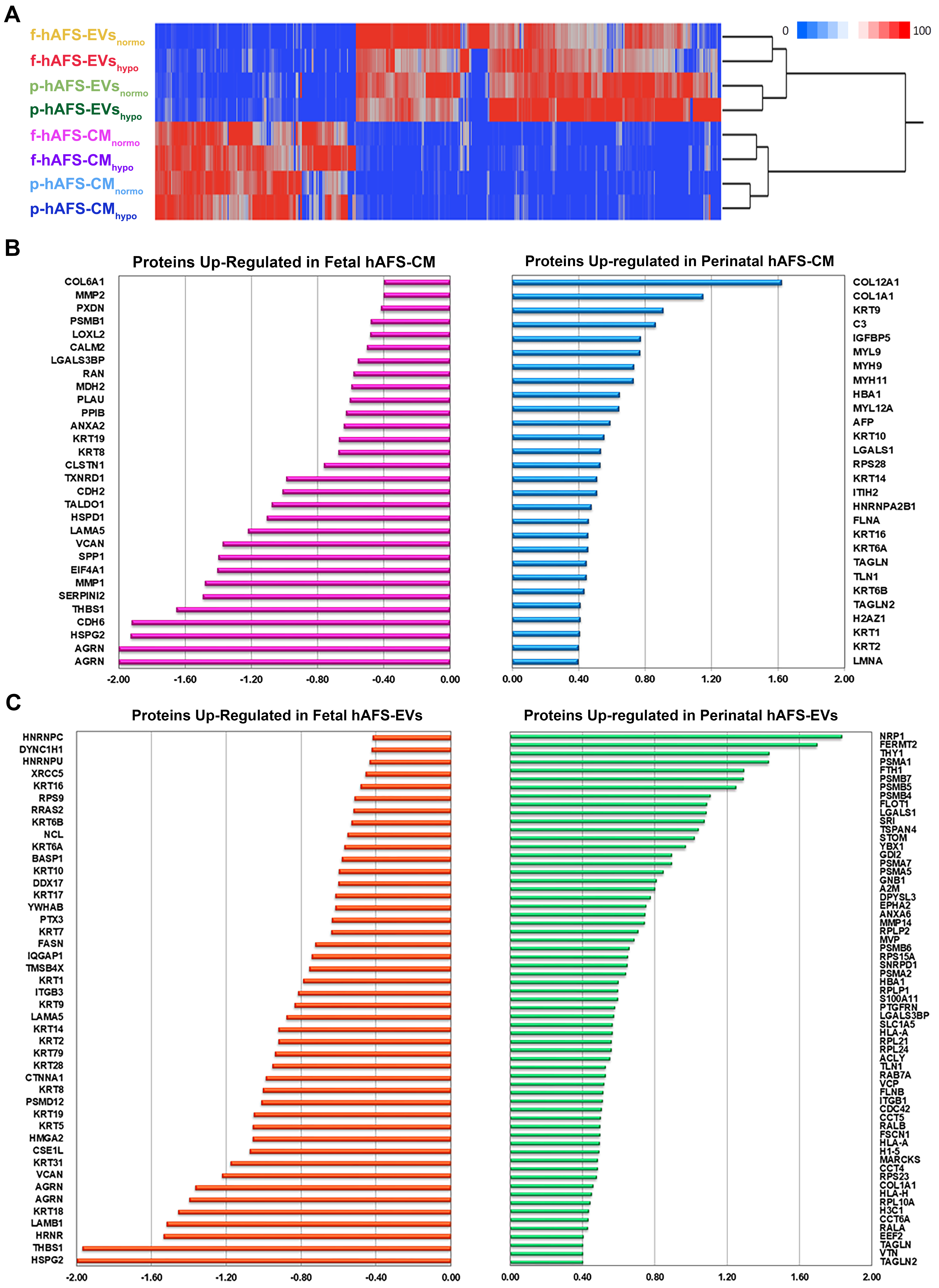

Supplement: Supplementary file 1 [file ijms-22-03713-s001.zip › Costa A et al_Supplementary Files/Costa A et al_Figure S1.tif]

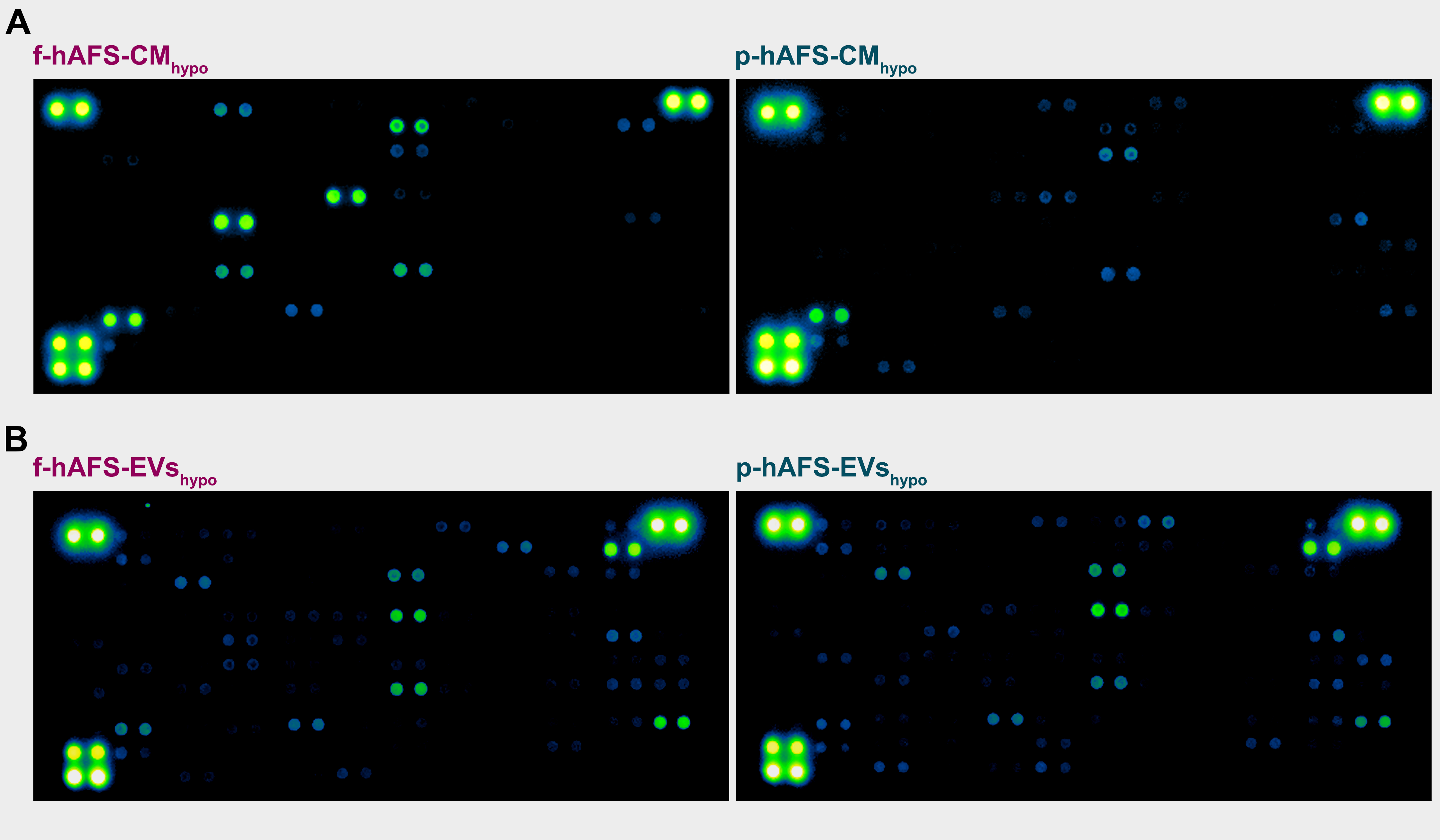

Supplement: Supplementary file 1 [file ijms-22-03713-s001.zip › Costa A et al_Supplementary Files/Costa et al_Figure S4.tif]
